# Chemoselective Oxyfunctionalization of Functionalized Benzylic Compounds with a Manganese Catalyst

**DOI:** 10.1002/anie.202205983

**Published:** 2022-06-08

**Authors:** Jimei Zhou, Minxian Jia, Menghui Song, Zhiliang Huang, Alexander Steiner, Qidong An, Jianwei Ma, Zhiyin Guo, Qianqian Zhang, Huaming Sun, Craig Robertson, John Bacsa, Jianliang Xiao, Chaoqun Li

**Affiliations:** ^1^ Key Laboratory of Applied Surface and Colloid Chemistry Ministry of Education and School of Chemistry and Chemical Engineering Shaanxi Normal University Xi'an 710119 China; ^2^ Department of Chemistry University of Liverpool Liverpool L69 7ZD UK; ^3^ Department of Chemistry Emory University 1515 Dickey Dr. Atlanta GA 30322 USA

**Keywords:** Benzylic Oxidation, Cyclic Imines, Ketones, Manganese Catalysts, Selective Oxidation

## Abstract

Whilst allowing for easy access to synthetically versatile motifs and for modification of bioactive molecules, the chemoselective benzylic oxidation reactions of functionalized alkyl arenes remain challenging. Reported in this study is a new non‐heme Mn catalyst stabilized by a bipiperidine‐based tetradentate ligand, which enables methylene oxidation of benzylic compounds by H_2_O_2_, showing high activity and excellent chemoselectivity under mild conditions. The protocol tolerates an unprecedentedly wide range of functional groups, including carboxylic acid and derivatives, ketone, cyano, azide, acetate, sulfonate, alkyne, amino acid, and amine units, thus providing a low‐cost, more sustainable and robust pathway for the facile synthesis of ketones, increase of complexity of organic molecules, and late‐stage modification of drugs.

## Graphical abstract

A new bipiperidine‐based manganese catalyst is introduced, which catalyzes the chemoselective benzylic oxidation of a wide range of diverse functionalized alkyl arenes with H_2_O_2_, affording various functionalized aryl ketones, cyclic imines, and bioactive molecules under mild conditions in a short time.

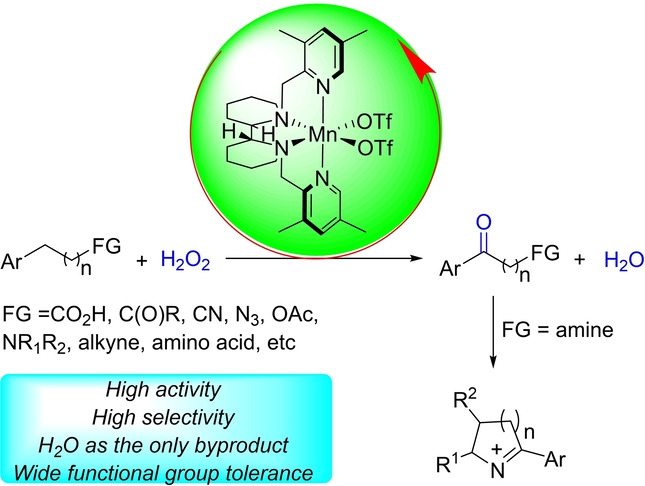

## Introduction

**Figure 1:**
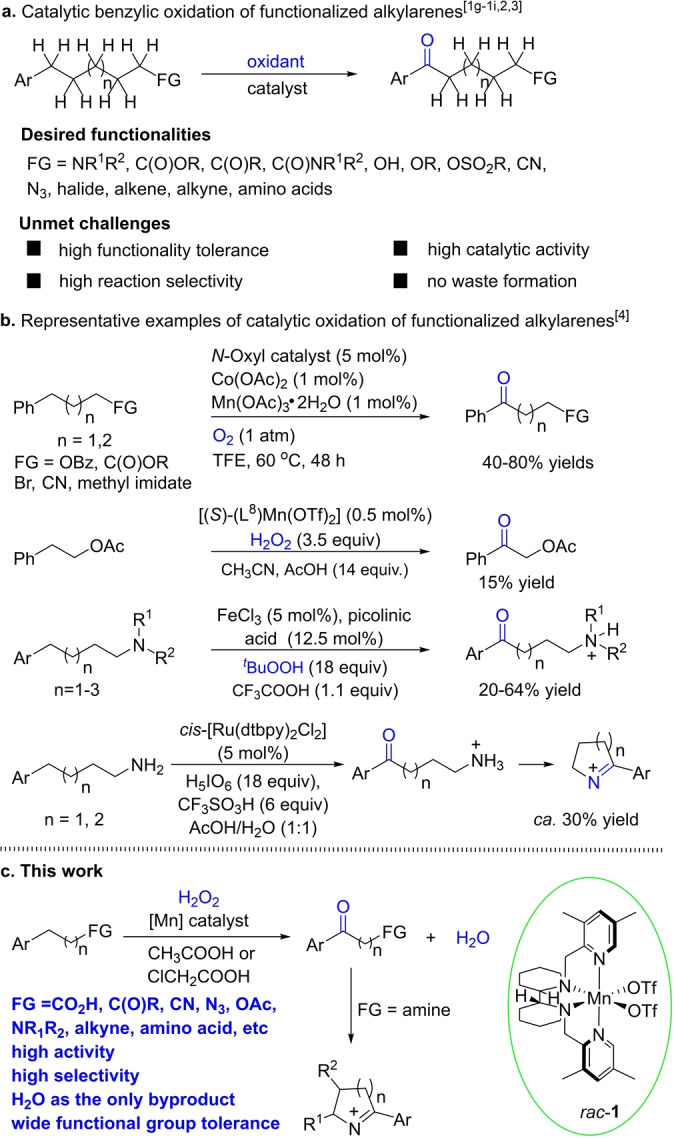
Catalytic benzylic methylene oxidation of alkyl chains bearing functional groups.

Catalytic oxidation of C−H bonds is a one‐step transformation to access highly value‐added hydroxyl and carbonyl compounds.^[1]^ Great strides have been made in understanding, controlling and expanding the scope of the reaction in recent years, thanks to the contributions of a number of research groups.^[2]^ Selective benzylic C−H oxidation would allow direct access to alkyl aryl ketones, such as those bearing functional groups on the alkyl unit, which are ubiquitous in fine chemicals, natural products and pharmaceuticals. They are also used in the synthesis of a wide range of essential organic building blocks, such as amines, amino acids, lactones, and heterocyclic compounds (see Figures S‐1, S‐2 and S‐3 in the Supporting Information). Indeed, various oxidation methods have been developed to enable benzylic oxidation.[^1b^, ^3^] However, there remains a significant issue, i.e. low compatibility with functional groups, with few methods known that tolerate some of the most common functional groups in organic synthesis (Figure 1a).[^1g^, ^1h^, ^1i^, ^2^, ^3^] Benzylic oxidation is traditionally performed with stoichiometric strong oxidants, such as CrO_3_, Na_2_CrO_4_, KMnO_4_, 2‐iodoxybenzoic acid and ^*t*^BuOOH.[^1g^, ^1h^, ^1i^] Apart from generating at least stoichiometric amounts of waste, these reagents are tarnished by the low tolerance of functional groups and they are mainly restricted to nonfunctionalized alkyl arenes.[^1g^, ^1h^, ^1i^, ^2^, ^3^] In response to these challenges, benzylic oxidation with transition metal catalysis,[^2b^, ^2d^, ^2e^, ^2f^, ^2g^, ^3^] and more recently, organocatalysis,[^2c^, ^4a^] electrochemistry,[^2j^, ^4b^] and photoredox chemistry[^2a^, ^4c^] has been actively pursued. Whilst these catalytic systems have advanced selective benzylic oxidation considerably, few of them have been demonstrated to tolerate a wide variety of functionalities on the alkyl side chains in benzylic compounds.^[4]^ Notably, the group of Oisaki and Kanai reported a *N*‐oxyl radical‐catalyzed benzylic oxidation of side chains bearing various functional groups,^[4d]^ and Sun and co‐workers reported a manganese catalyst that promotes benzylic oxidation of phenethyl acetate (Figure 1b).^[4h]^ However, no catalytic systems are known of capable of benzylic oxidation of side chains bearing such important functionalities as alkyne, alkene, azide, amino acid, or free amine moieties.

Being the most widely seen functionality in natural products and pharmaceuticals, amines draw particular attention. However, the nitrogen lone pair lowers the bond dissociation energy (BDE) of the neighboring C−H bond (c.f. BDE 98 for MeC**H_2_** Me and 90 for MeC**H_2_** NH_2_ kcal mol^−1^)^[5]^ and is prone to coordinate to metal centers, complicating the chemoselectivity of oxidation while poisoning metal‐based catalysts.[^4j^, ^4k^, ^6^] Indeed, very few examples have been reported of benzylic oxidation of aryl aliphatic amines. By virtue of Brönsted acid protonation of or Lewis acid coordination to nitrogen, Sanford and co‐workers achieved an iron‐catalyzed remote benzylic oxidation with ^*t*^BuOOH of a range of aliphatic tertiary amines, in which CF_3_CO_2_H was used to protonate the amines (Figure 1b).^[4j]^ In a similar fashion, the groups of Du Bois and Sigman disclosed a ruthenium‐catalyzed benzylic oxidation of aliphatic primary amines which led to cyclic imines, with H_5_IO_6_ being the oxidant and CF_3_SO_3_H as an acid additive (Figure 1b).^[4k]^ However, the use of a strong acid such as CF_3_SO_3_H and a strong oxidant like H_5_IO_6_ may limit the application of these methods. For instance, functionalities, such as nitriles, alkynes, esters, amides and the common protecting groups for amines, alcohols and amino acids, could undergo acid‐promoted decomposition. Aiming to expand the boundary of functional groups in benzylic oxidation, we have become interested in developing a more versatile, enabling catalytic system that is desirably also cheaper and cleaner.

In biological systems, selective C−H bond oxidation is primarily performed by high‐valent metal‐oxo species formed by oxygenases reacting with O_2_.^[7]^ In pioneering studies, Que and co‐workers reported the non‐heme biomimetic Fe complex [Fe(TPA)(CH_3_CN)_2_]^2+^ (TPA=tris(2‐pyridylmethyl)amine), which catalyzes selective oxidation by H_2_O_2_, with water as the only byproduct.^[8]^ This is followed by the advent of a series of highly efficient oxygenation catalysts based on Fe and Mn complexes bearing tetradentate amino ligands,[^2d^, ^2f^, ^7d^, ^9^] culminated by the novel bipyrrolidine‐bipyridine (BPBP) ligands discovered by the White group and further developed by the groups of White, Costas, and Bryliakov.^[10]^ In 2012, Rybak‐Akimova and co‐workers reported a similar Fe‐PYBP complex, with the PYBP ligand featuring two pyridines bridged by a bipiperidine. The complex was shown to be highly active and selective in catalyzing olefin epoxidation with H_2_O_2_.^[11]^ In our search for competent catalysts for selective C−H oxidation, we have found that the Mn complex of a similar ligand allows for highly selective benzylic C−H oxidation of arene side chains with H_2_O_2_, tolerating an unprecedentedly wide variety of functional groups and thus providing a more general method for benzylic oxidation (Figure 1c).

## Results and Discussion

### Reaction Development

In continuing our study of selective oxidation with catalysts and oxidants that respond to the Green Chemistry agenda,^[12]^ we concentrated on searching for an able catalyst based on the cheap, biocompatible Fe or Mn for the selective oxidation of functionalized alkylarenes with H_2_O_2_ as oxidant, which would generate water as the only byproduct. Considering the importance of alkyl aryl ketones functionalized with a carboxylate group in synthesis (see Figure S‐3 for examples), we set out to investigate the oxidation of 4‐phenylbutanoic acid (**2**) as the model substrate to identify a possible catalyst. The results of examining the effect of various metal salts and ligands on the oxidation are summarized Table 1 (for more details, see Table S‐1 in the Supporting Information). As can be seen, the simple manganese salt, Mn(OTf)_2_ (2 mol %), showed no activity for the catalytic oxidation. Following on from the study of Rybak‐Akimova,^[11]^ we envisaged that bipiperidine, which is readily accessible from the hydrogenation of bipyridine, could provide a rigid ligand backbone, and therefore synthesized the PYBP type ligand 1,1′‐bis((3,5‐dimethylpyridin‐2‐yl)methyl)‐2,2′‐bipiperidine **L^1^**. The ligand exists as racemic and *meso* isomers, *rac*‐**L^1^** and *meso*‐**L^1^**, which could be separated into analytically pure forms by flash column chromatography (see Supporting Information for details). Delightfully, combining *rac*‐**L^1^** with Mn(OTf)_2_ (1 : 1 ratio) afforded the ketone **2 a** in an excellent yield of 90 %. In stark contrast, the analogous *meso*‐**L^1^** was much less effective, as can be seen from Table 1. This is reminiscent of the observations made with other *meso*‐PYBP and *meso*‐BPBP ligands in Fe and Mn catalyzed oxidation reactions.^[13]^

**Table 1:**
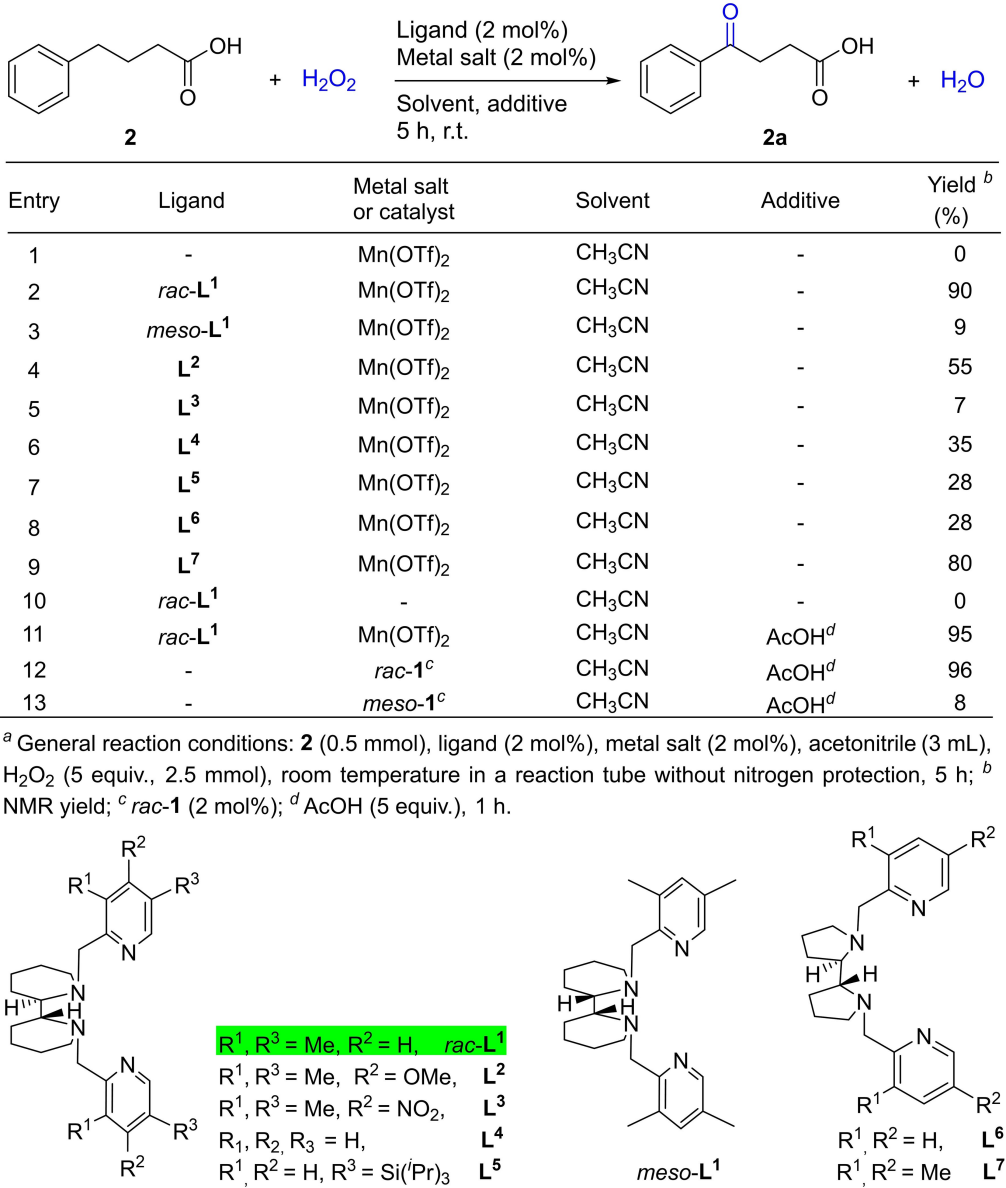
Identification of catalyst for benzylic oxidation.

Aimed at increasing the catalyst activity further, we prepared the electronically and sterically varied analogues of *rac*‐**L^1^**, i.e. *R*,*R*‐**L^2^**, *R*,*R*‐**L^3^**, *R*,*R*‐**L^4^**, and *R*,*R*‐**L^5^**, and examined their effect on the model oxidation, in addition to that of the BPBP ligands **L^6^** and **L^7^**.^[14]^ As can be seen, none of them outperformed *rac*‐**L^1^**, and in particular, whilst the electron‐donating methoxy substituent reduces the catalytic activity (entry 4), the electron‐withdrawing nitro group renders the catalyst much less active (entry 5). The reason as to why both substituents deactivate the catalyst is not immediately clear. Further screening of other ligands as well as the combination of *rac*‐**L^1^** with other manganese or iron salts demonstrated that *rac*‐**L^1^** combined with Mn(OTf)_2_ exhibits the best catalytic activity in the oxidation of **2** with H_2_O_2_ (see Table S‐1 in the Supporting Information). The reaction time could be shortened from 5 h to 1 h by adding 5 equivalents of acetic acid without affecting the yield of **2 a** (entry 11). Carboxylic acids are known to promote manganese‐catalyzed oxidation.[^7d^, ^9^, ^10^, ^11^]

**Figure 2:**
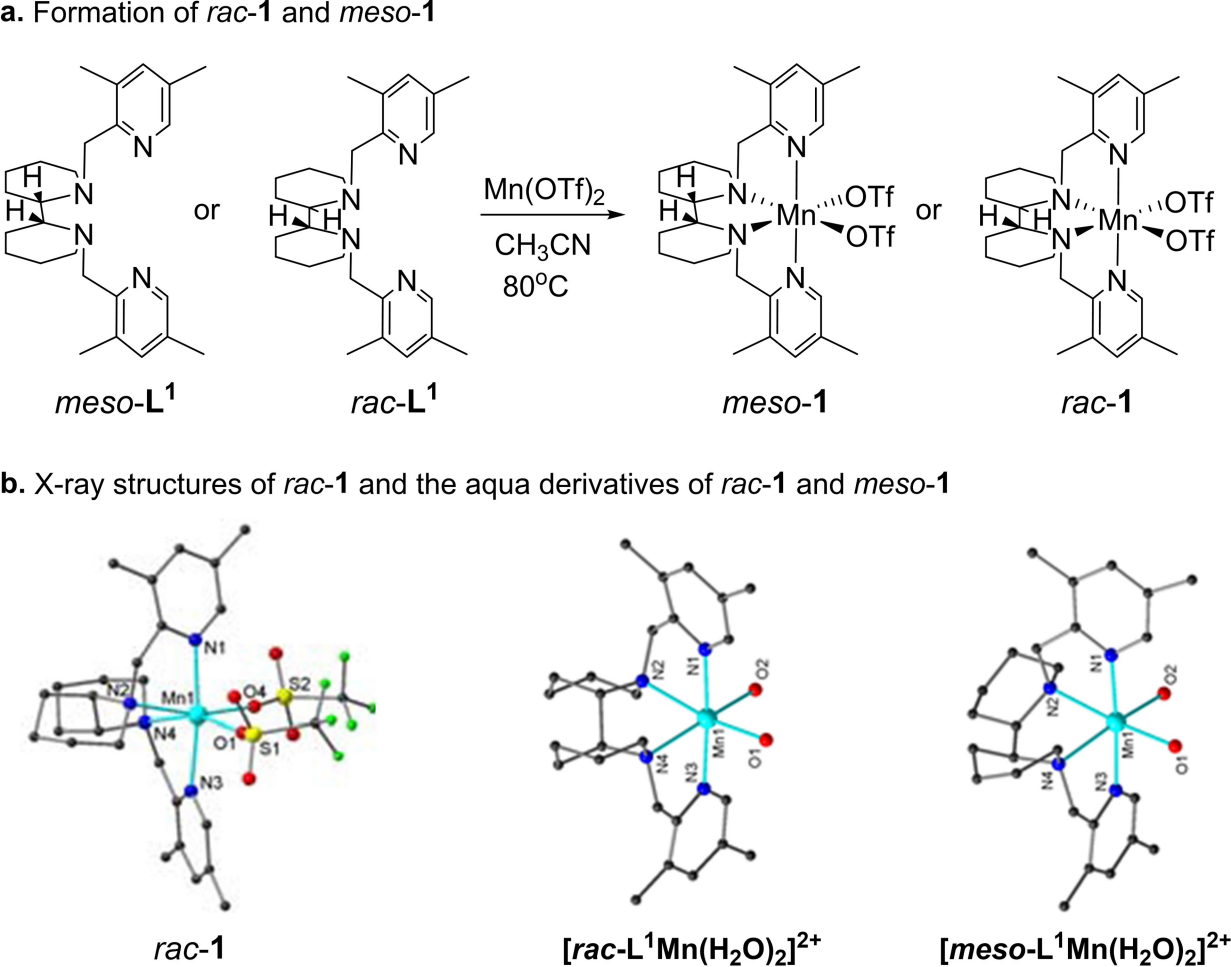
Formation of *rac*‐**1** and *meso*‐**1** and the X‐ray structures of *rac*‐**1** and the cations [**L^1^** Mn(H_2_O)_2_]^2+^ of *rac*‐**1** and *meso*‐**1**. Selected bond lengths [Å] for *rac*‐**1**: Mn1−N1 2.237(2), Mn1−N3 2.260(2), Mn1−N2 2.284(2), Mn1−N4 2.299(2), Mn1−O1 2.131(2), Mn1−O4 2.140(2); for [*rac*‐**L^1^** Mn(H_2_O)_2_]^2+^: Mn1−N1 2.2528(19), Mn1−N2 2.2979(18), Mn1−N3 2.2412(19), Mn1−N4 2.2999(18), Mn1−O1 2.1624(17), Mn1−O2 2.1644(18); for [*meso*‐**L^1^** Mn(H_2_O)_2_]^2+^: Mn1−N1 2.244(12), Mn1−N2 2.281(15), Mn1−N3 2.243(12), Mn1−N4 2.309(12), Mn1−O1 2.13(2), Mn1−O2 2.222(18). Hydrogen atoms have been omitted for clarity. See the Supporting Information for more details.

Reacting *rac*‐**L^1^** and *meso*‐**L^1^** with Mn(OTf)_2_ led to the complexes *rac*‐**1** and *meso*‐**1**, respectively (Figure 2a). The structures of *rac*‐**1** and the aqua derivatives of *rac*‐**1** and *meso*‐**1** resulting from substitution of the triflate anion with water have been determined by X‐ray diffraction (see Supporting Information for details).^[15]^ The latter two exhibit very similar M−L bond lengths and coordination geometries, and both show a distorted octahedral *cis*‐α geometry (Figure 2b). The complex [*rac*‐**L^1^** Mn(H_2_O)_2_]^2+^ is approximately *C*_2_ symmetric, in which both piperidine rings exhibit chair conformation, while in the *meso*‐analogue one piperidine ring has a chair and the other a boat conformation. The axial Mn−N distances are very similar in both aqua complexes measuring between 2.24 and 2.26 Å. The equatorial Mn−N distances in [*rac*‐**L^1^** Mn(H_2_O)_2_]^2+^ are virtually identical, while in [*meso*‐**L^1^** Mn(H_2_O)_2_]^2+^ they are slightly longer to the ligand N‐atom that is part of the boat‐configured piperidine ring. The boat conformation indicates that the *meso*‐ligand is under some strain. The metal coordination forces the C−N bond that is both part of the ligand backbone and the piperidine ring into an eclipsed conformation which imposes the boat shape.

The isolated *rac*‐**1** and *meso*‐**1** displayed a similar activity to that prepared in situ (Table 1, entries 2 vs 12, and entries 3 vs 13). Further screening led to the optimized oxidation conditions as: *rac*‐**1** (2 mol %) being the catalyst, H_2_O_2_ (5 equiv) as the oxidant, acetic acid (5 equiv) as an additive in CH_3_CN at room temperature, and 1 h reaction time (see Table S‐2). Under the optimized conditions, **2** was oxidized to **2 a** with a high yield of 96 % (entry 12).

**Figure 3:**
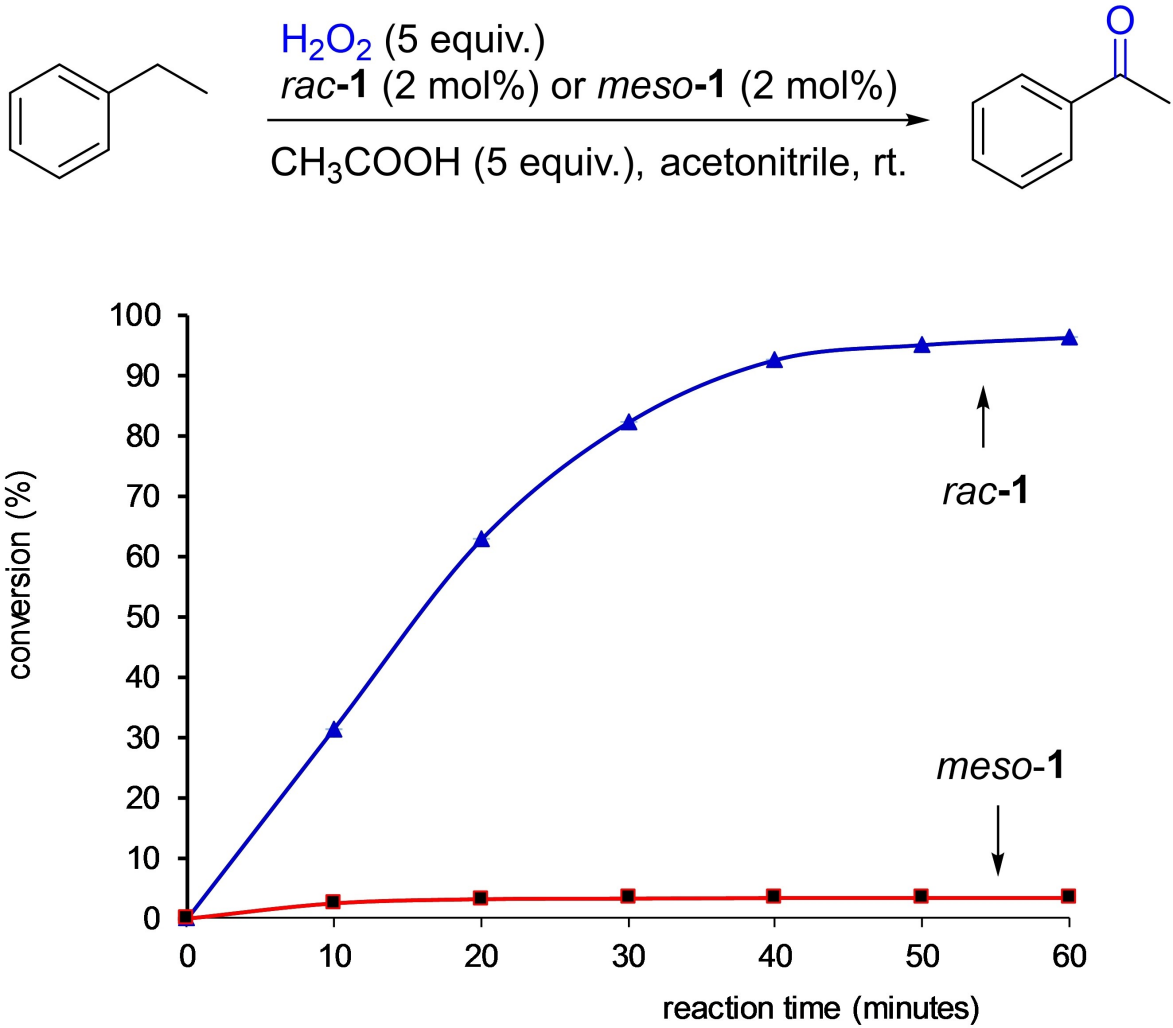
The time course of *rac*‐**1** and *meso*‐**1** catalyzed benzylic oxidation of ethyl benzene under the standard conditions.

The superior activity of *rac*‐**1** to *meso*‐**1** is further illustrated by the kinetic profiles observed in the oxidation of ethyl benzene under the optimized conditions (Figure 3). While *rac*‐**1** catalyzed fast conversion of ethyl benzene to acetophenone with *t*_1/2_<20 min, *meso*‐**1** showed only insignificant activity throughout the course of the reaction. The color change is also indicative. The *rac*‐**1** mediated reaction turned to reddish almost immediately upon introduction of H_2_O_2_, whereas the *meso*‐**1** reaction remained almost colorless throughout, suggesting inability to activate H_2_O_2_. This activity difference is in stark contrast to the structural similarity seen in the two complexes. We also compared the activity of *rac*‐**1** with two well‐known manganese oxidation catalysts[^4h^, ^9b^, ^10^] in the benzylic oxidation of alkyl chains with a range of functional groups. It is remarkable that in all the cases examined, *rac*‐**1** afforded significantly higher product yields (see Table S‐6 in Supporting Information).

### Scope of Reaction

#### Benzylic Oxidation to Access Functionalized Arene Ketones

**Figure 4:**
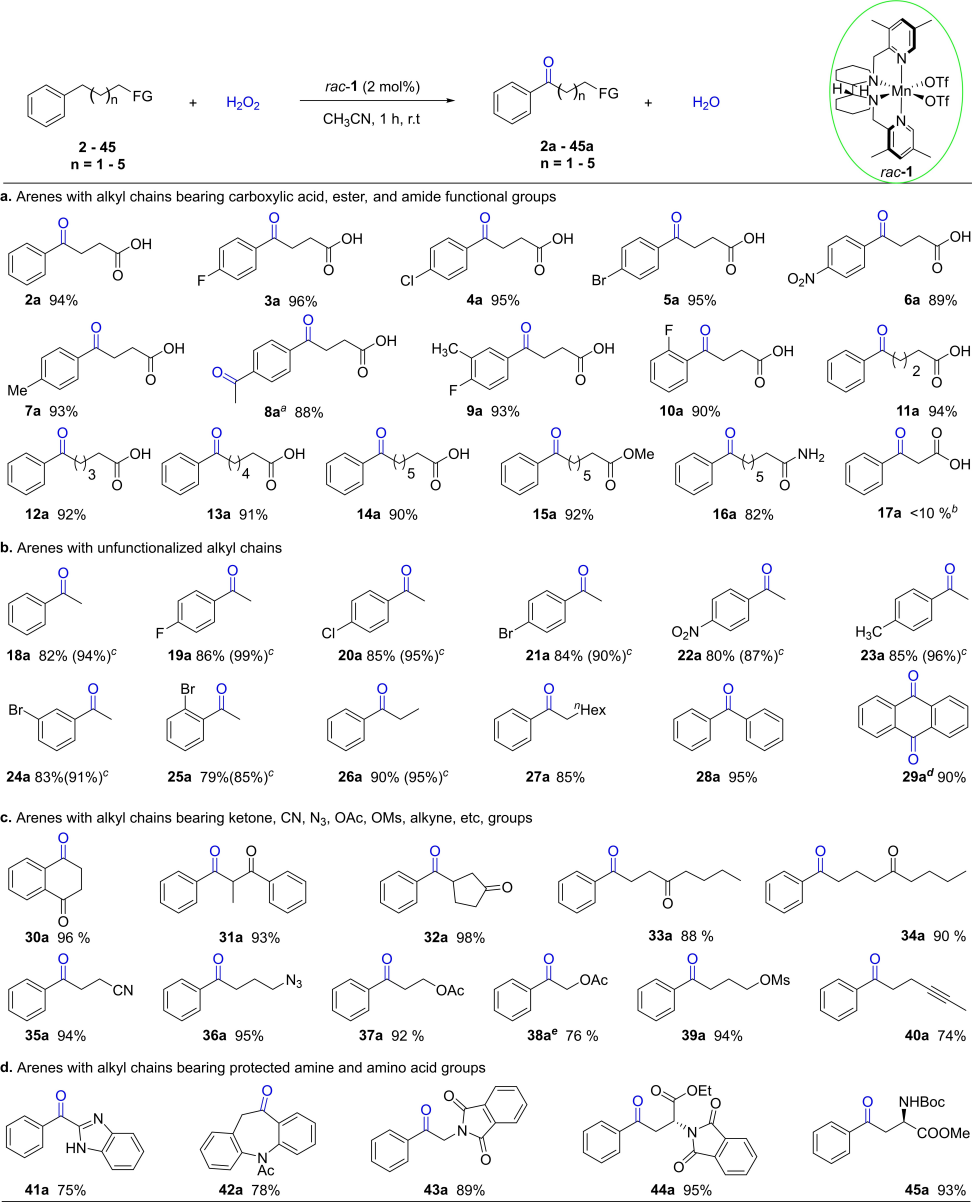
Substrate scope of the *rac*‐**1** catalyzed benzylic oxidation of alkylarenes. General reaction conditions: substrate (0.5 mmol), *rac*‐**1** (2 mol %), and AcOH (2.5 mmol) were dissolved in MeCN (1.5 mL), and then H_2_O_2_ (2.5 mmol) in 1 mL of MeCN was introduced with a syringe pump over 1 h under stirring at room temperature without nitrogen protection. Isolated yield reported; [a] H_2_O_2_ (4 mmol); [b] ^1^H NMR yield; [c] GC yield in parentheses; [d] 2 mol % of *rac*‐**1** at the beginning followed by another 2 mol % 20 minutes later; H_2_O_2_ (4 mmol) in 1 mL of MeCN delivered with a syringe pump over 1 h; [e] AcOH (7.5 mmol, 15 equiv).

Under the optimized reaction conditions, the catalytic system proved to be generally effective for the selective benzylic oxidation of various aryl alkanoic acids to aryl ketone acids (Figure 4a). Using 4‐phenylbutanoic acid as a reference, the effect of substituent group at the phenyl ring was examined. As is clear, the protocol tolerates both electron‐donating (*p*‐Me) and electron‐withdrawing (*p*‐F, *p*‐Cl, *p*‐Br, *p*‐NO_2_) substituents of high Hammett constant (σ_p_=0.78, NO_2_), affording the aryl ketone acids with excellent yields (**2 a**–**9 a**). Note that on replacing *p*‐Me with *p*‐Et on the phenyl ring, both benzylic sites of substrate **8** were oxidized to give the diketone **8 a** with 88 % yield when more H_2_O_2_ was used (8 equiv). *meta*‐Substitution has no notable effect on the product yield (**9 a**), and the same is true with *ortho*‐fluoro substitution (**10 a**).

Considering the electron‐withdrawing effect of the carboxylic acid unit and its potential coordination to the metal center, both of which could affect the benzylic oxidation, the impact of the alkyl chain length was examined. As demonstrated by the high yields of **11 a**–**14 a**, separating the phenyl ring from the carboxylic acid with longer alkyl chains had little effect on the efficacy of the oxidation. Furthermore, the aryl acid derivatives, an aryl ketone ester **15 a** and amide **16 a**, were also obtained in good yield. These observations appear to indicate that the acid functionality has no directing or promoting effect on the oxidation under the conditions employed, where excess AcOH was present.^[16]^ However, the shorter chain substrate 3‐phenylpropanoic acid **17** showed only a low activity (<10 % yield of **17 a**). This could result from a deactivating effect of the electron‐withdrawing carboxyl group^[2g]^ and/or product chelation to the metal center which inhibits the catalytic turnover. In support of the hypothesis on chelation, addition of 2‐oxo‐2‐phenylacetic acid to the model reaction reduced the yield of **2 a** to 50 % (cf. entry 24, Table S‐2).

As maybe expected, alkylbenzenes without the acid functionality are viable (Figure 4b). Thus, ethyl benzene and its derivatives containing both electron‐donating and withdrawing substituents were oxidized, leading to the aryl ketones with excellent yields (**18 a**–**25 a**). Note that the large *ortho*‐bromo moiety does not appear to hinder the oxidation of **25**. Subsequently, phenyl rings bearing longer alkyl chains (**26**, **27**) and a benzyl group (**28**) were oxidized to phenyl ketones (**26 a**–**28 a**) in high yields under the standard conditions. Anthraquinone (**29 a**), an important industrial organic chemical, was obtained in 90 % yield, albeit with a higher catalyst loading of 4 mol %.

To probe further the functional group tolerance of the protocol enabled by *rac*‐**1**, we examined alkylarenes containing a range of diverse functionalities in the alkyl chain (Figure 4c). As can be seen, substrates bearing ketone (**30**–**34**), nitrile (**35**), azide (**36**), OAc (**37**, **38**), OMs (**39**) and alkyne (**40**) units all underwent the benzylic oxidation, affording ketones in high yields in general. The lower yield of **38 a** is probably again due to a deactivating and/or coordinating effect of the acetate unit. In the case of **40 a**, the lower yield results partially from the oxidation of the alkyne moiety.

**Figure 5:**
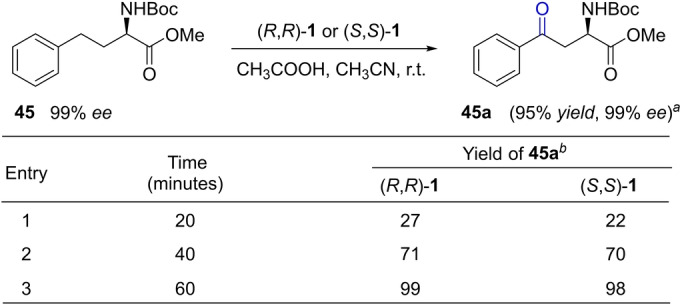
Kinetic follow of the oxidation of a chiral substrate with chiral catalysts. Reaction conditions were the same as the general conditions given in Figure 4; [a] (*R*,*R*)‐**1** as catalyst, 1 h, with *ee* measured by chiral HPLC and isolated yield given. [b] ^1^H NMR yield.

A still further demonstration of the applicability of the protocol is seen in the selective benzylic oxidation of compounds having protected amino groups (Figure 4d). Thus, under the optimized conditions, 2‐benzyl‐1*H*‐1,3‐benzodiazole (**41**) was converted to the corresponding ketone (**41 a**) in 75 % yield, and oxidation of **42** yielded an analogue of oxcarbazepine (**42 a**), a drug used to treat epilepsy and bipolar disorder, in 78 % yield. Notably, phthalimide **43** was oxidized to **43 a** in 89 % yield, in which the relatively bulky, potentially coordinating phthalimide neighbors the newly formed ketone. In addition, the protected amino acids **44** and **45** were both oxyfunctionalized with excellent yield via benzylic oxidation. Further examination of the oxidation of **45** shows the reaction to be highly site selective, with the α−C−H bond of the amino ester unit remaining intact, as revealed by the full retention of its stereochemistry (Figure 5). With other methods, such bonds could be more prone to react.^[17]^ Interestingly, the enantiomerically pure catalysts, (*R*,*R*)‐**1** and (*S*,*S*)‐**1** prepared from the enantiomerically pure **L^1^**, showed little kinetic differentiation in oxidizing **45**, as evidenced by the time‐yield data given in Figure 5.

The survival of the various functional groups in the oxidation above is remarkable, demonstrating the *rac*‐**1** enabled protocol to be highly tolerate of functionalities. It is noted that these functionalities are characterized by widely differing electronic and steric properties and sensitivity to redox reactions, acids and aqueous conditions, as highlighted by the highly electron‐withdrawing ketone and nitrile moieties and highly reactive azide and alkyne groups. The presence of a second functionality in the ketone products makes the ketones synthetically much more useful; a number of bioactive molecules can be derived from these products (see Supporting Information, Figure S‐2). As far as we are aware, there appear to be only a few reports on the oxidation of alkylarenes with the alkyl chain functionalized with a nitrile, an acetoxy, or a mesylate moiety,[^4d^, ^4f^, ^4h^] and no study is known of those bearing an azide or alkyne group.^[18]^

#### Benzylic Oxidation of Primary Amines to Access Cyclic Imines

**Figure 6:**
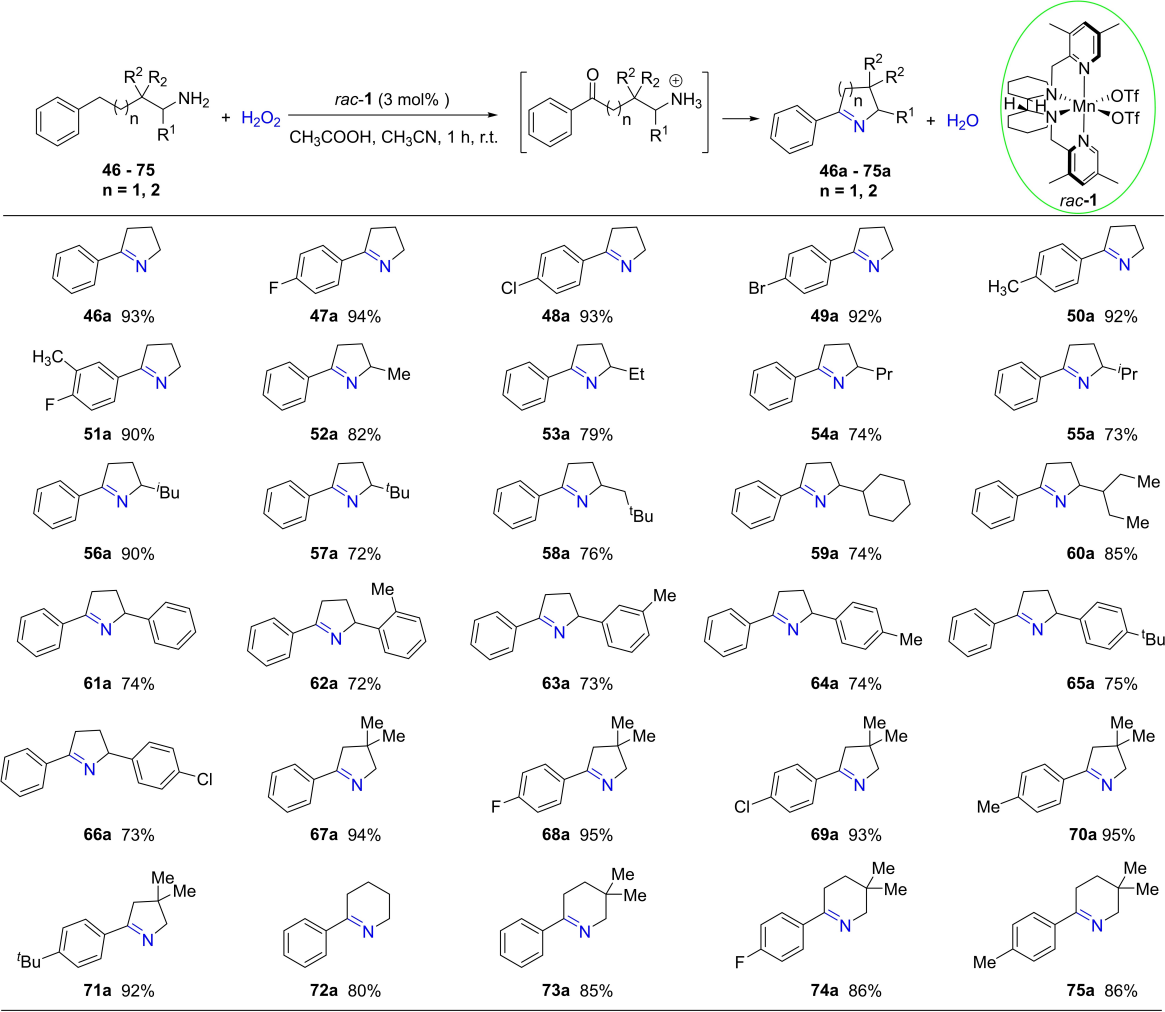
Substrate scope of *rac*‐**1** catalyzed benzylic oxidation of alkylarenes containing primary amines. General reaction conditions: substrate (0.5 mmol) and *rac*‐**1** (3 mol %) were dissolved in AcOH (1.5 mL), and then H_2_O_2_ (2.5 mmol) in 1.5 mL of MeCN was introduced with a syringe pump over 1 h under stirring at room temperature without nitrogen protection. Isolated yield reported.

Prompted by the importance of and challenges in amine oxidation, we went on to explore the benzylic oxidation of alkylarenes bearing an aliphatic amine side chain under the catalysis of *rac*‐**1** (Figure 6). Delightedly, with 4‐phenylbutan‐1‐amine (**46**) as a model substrate, the cyclic imine **46 a**, formed clearly via the ketone intermediate resulting from benzylic oxidation, was obtained in 23 % yield under the standard conditions. Increasing the amount of catalyst loading from 2 mol % to 3 mol %, the yield increased to 35 %. Bearing in mind the benefiting effect of acids demonstrated in previous studies of amine oxidation,[^4j^, ^4k^] we varied the amount of the acid. Remarkably, when the oxidation was conducted in a mixed solvent of AcOH and MeCN (1 : 1 volume), which did not decompose the catalyst, the starting material was completely transformed to the product **46 a** with an excellent isolated yield of 93 % (see Table S‐3 in the Supporting Information). Using an analogous Fe‐BPBP catalyst for a similar oxidation, Sanford and co‐workers noticed only substrate decomposition in the presence of CF_3_CO_2_H and no formation of the desired amino ketone product.^[4j]^

Under the newly optimized conditions, rac‐**1** was shown to be effective for the selective benzylic oxidation of a wide range of aliphatic amines, affording, in one step, synthetically highly valuable five and six‐membered cyclic imines (Figure 6). Thus, starting with 4‐arylbutan‐1‐amines, the 5‐memebred dihydropyrroles were obtained in over 90 % yields, regardless of the 4‐substituent on the phenyl ring being electron‐withdrawing (*p*‐F, *p*‐Cl, *p*‐Br) or electron‐donating (*p*‐Me) (**46 a**–**51 a**). The utility of the protocol was further explored by installing substituents α or β to the amino unit; this would lead to value‐added multisubstituted pyrrolidines. Pleasingly, the protocol tolerates a range of sterically and electronically varied alkyl and aryl substituents, as showcased by the imine products 5‐phenyl‐2‐alkyl (**52 a**–**60 a**) and 5‐phenyl‐2‐aryl (**61 a**–**66 a**) dihydropyrroles. Of particular note is that amines bearing the sterically demanding 2‐^*t*^Bu and 2‐(*o*‐tolyl) moieties are viable, although the product yields are lower. Equally significant is the synthesis of 2‐aryl‐4,4‐dimenthyl substituted five as well as six‐membered cyclic imines (**67 a**–**71 a**,**72 a**–**75 a**) in high yields. An easy one‐step reaction, i.e. hydrogenation, would convert these *N*‐heterocycles into highly sought‐after pyrrolidines and piperidines, the most important heterocycles seen in top‐selling drugs.^[19]^

#### Application in the Synthesis of Drug and Bioactive Molecules

**Figure 7:**
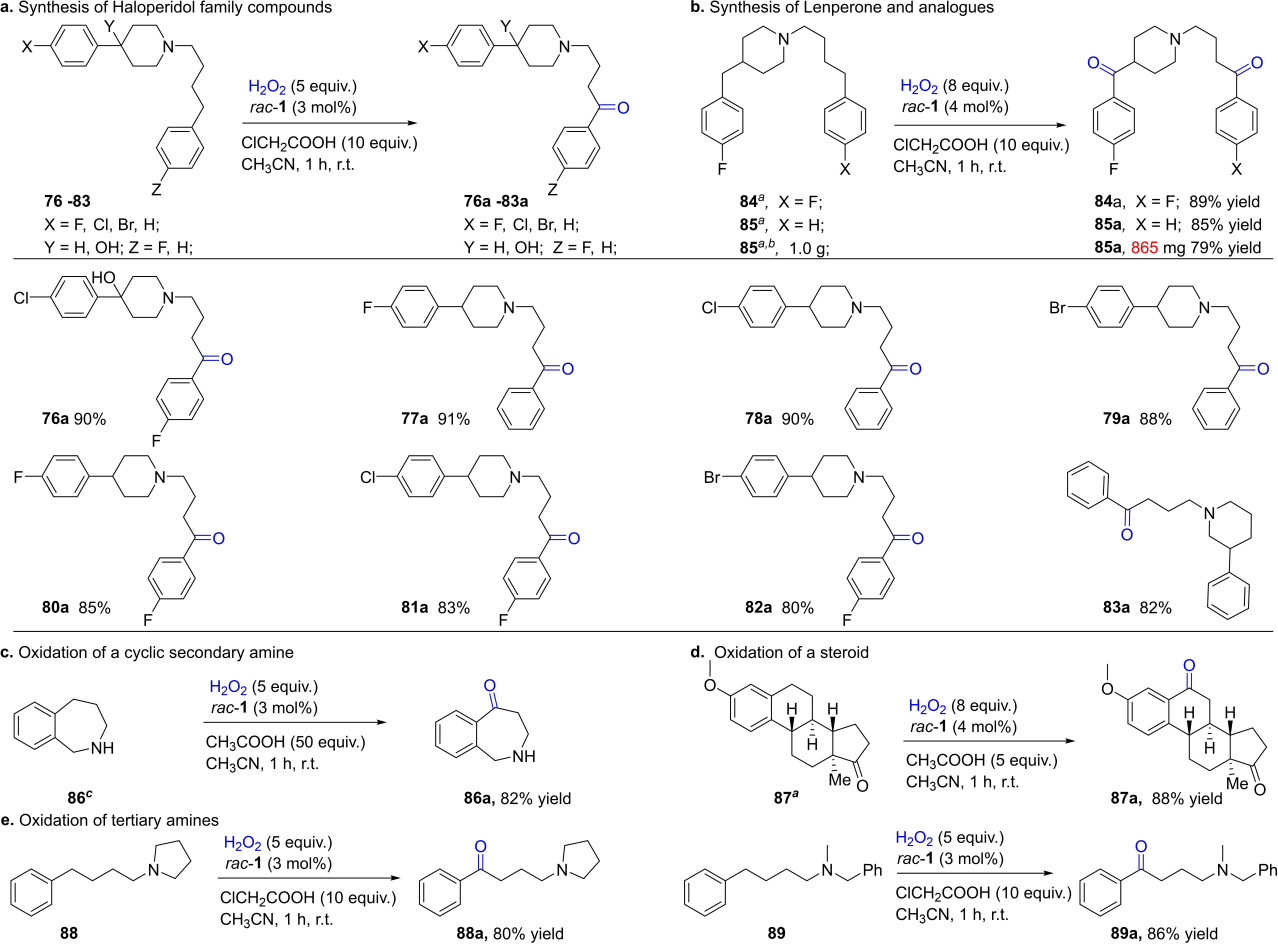
Oxyfunctionalization of bioactive and drug molecules via *rac*‐**1** catalyzed benzylic oxidation. General reaction conditions: substrate (0.25 mmol), *rac*‐**1** (3 mol %), and ClCH_2_COOH (2.5 mmol) were dissolved in MeCN (1.0 mL), and then H_2_O_2_ (1.25 mmol) in 1 mL of MeCN was introduced with a syringe pump over 1 h under stirring at room temperature without nitrogen protection. Isolated yield reported. [a] 2 mol % *rac*‐**1** at the beginning followed by another 2 mol % 20 minutes later; H_2_O_2_ (2 mmol) in 1 mL of MeCN delivered with a syringe pump over 1 h; [b] **85** (3.1 mmol, 1.0 g); [c] reaction conditions were the same as the general conditions given in Figure 6.

The high reactivity and chemoselectivity displayed by *rac*‐**1** for benzylic methylene oxidations in the presence of pharmaceutically relevant arenes and aminoalkanes provide an opportunity to effect late‐stage oxyfunctionalization of drug or other bioactive molecules. As an example, the drug molecule haloperidol (**76 a**), a nonselective D4 antagonist,^[20]^ was obtained in 90 % yield from the readily available precursor **76**, when HOAc was replaced with 10 equiv of a stronger acid, ClCH_2_CO_2_H (Figure 7). Under the standard conditions (see Figure 6), a much lower conversion of **76** (<10 %) was observed, however. The analogous dehydroxylated **77 a**–**83 a** were obtained in high yields similarly. Note that in these tertiary amino substrates, there are multiple sites that are susceptible to oxidation, i.e. the tertiary benzylic C−H bond and the secondary C−H bonds α to the nitrogen. The oxidation of **81** with ^*t*^BuOOH (18 equiv) catalyzed by FeCl_3_ has been reported by Sanford and co‐workers; but the yield was lower (41 %).^[4j]^ The isolation of **77 a**–**83 a** in high yields demonstrates the high chemoselectivity of *rac*‐**1**.

Where there are two benzylic sites, double oxidation takes place, as in the case of dihydroanthracene (**29**, Figure 4). This feature was taken advantage of in the synthesis of the drug lenperone (**84 a**), an antipsychotic, and an analogue **85 a**, with both compounds obtained in high yields. Although **84** could be oxidized to **84 a** with ^*t*^BuOOH under FeCl_3_ catalysis^[4j]^ or with free radical‐enabled molecular oxygen,^[4i]^ the reaction was less efficient and featured harsher conditions (54 equiv oxidant, 5 days, 26 % yield in the former case; 90 °C, 36 h, 62 % yield in the latter). Oxidation of a secondary amine, tetrahydrobenzazepine (**86**), was also possible, affording in good yield the tetrahydrobenzazepinone **86 a**, a building block for the synthesis of a respiratory syncytial virus fusion inhibitor (Figure 7c).^[21]^ Note that the oxidation took place remotely to the amino moiety; this may stem from an acid‐induced deactivation of the benzylic C−H bond α to nitrogen via protonation.^[6f]^ Furthermore, the protocol is shown to be highly efficient in the selective oxidation of a more complex steroid molecule, an estrone derivative **87**, converting it to a ketone product **87 a** with 88 % yield under the standard conditions (Figure 7d). In addition to the piperidine‐type tertiary amine substrates shown in Figure 7a, tertiary amines featuring pyrrolidine (**88**) and benzyl (**89**) units were also demonstrated to be viable. Oxidation of both substrates afforded the benzylic oxidation products in high yields (Figure 7e).

To further demonstrate the synthetic utility of *rac*‐**1**, the substrate **85** was subjected to a gram scale reaction. As shown in Figure 7b, the corresponding product **85 a** was obtained in 79 % isolated yield within a 1 h reaction. The somewhat lowered yield is due to the oxidation being incomplete in 1 h, with some **85** remaining in the reaction mixture.

## Conclusion

Green, selective oxidation of C−H bonds is one of the most significant challenges facing organic synthesis. The key to addressing the challenge lies in developing able catalysts. The *rac*‐**1** complex described in this paper contributes to widening the scope of benzylic oxidation with a benign oxidant, H_2_O_2_, showing an unprecedented level of functional group tolerance. As such, functionalized aryl ketones, cyclic imines, and modified bioactive molecules can be reached in a single step of oxidation, with no toxic waste generated. Carboxylic acid‐assisted mechanisms of benzylic oxidation with PYBP type ligands have been well studied by several groups.[^9a^, ^22^] A similar reaction pathway involving active Mn^V^=O species may well follow by the *rac*‐**1** catalyst.[^22c^, ^22d^, ^22e^, ^22f^, ^22g^, ^22h^] Although the full mechanistic details, including the cause of the remarkable difference between *rac*‐**1** and *meso*‐**1**, remain to be delineated, we anticipate that the *rac*‐**1**/H_2_O_2_ system will find applications in selective oxidation of C−H bonds and in the synthesis of complex organic chemicals.

## Conflict of interest

The authors declare no conflict of interest.

1

## Supporting information

As a service to our authors and readers, this journal provides supporting information supplied by the authors. Such materials are peer reviewed and may be re‐organized for online delivery, but are not copy‐edited or typeset. Technical support issues arising from supporting information (other than missing files) should be addressed to the authors.

Supporting Information

Supporting Information

Supporting Information

Supporting Information

## Data Availability

The data that support the findings of this study are available in the Supporting Information of this article.
