# Supplementary material for: Chemoselective Oxyfunctionalization of Functionalized Benzylic Compounds with a Manganese Catalyst
Source: Angew Chem Int Ed Engl. 2022 Jun 8;61(30):e202205983. doi: 10.1002/anie.202205983 (PMC9400980; doi:10.1002/anie.202205983)
Supplement: Supplementary file 2 — Supporting Information [file ANIE-61-0-s001.pdf]

Angewandte Chemie
Eine Zeitschrift der Gesellschaft Deutscher Chemiker

Supporting Information

Chemoselective Oxyfunctionalization of Functionalized Benzylic Compounds with a Manganese Catalyst

J. Zhou, M. Jia, M. Song, Z. Huang, A. Steiner, Q. An, J. Ma, Z. Guo, Q. Zhang, H. Sun, C. Robertson, J. Bacsa, J. Xiao, C. Li**

Table of Contents

1. The importantance of functionalized arene ketones, cyclic imines and amines ………...…………………...………...………………….…………...…...……….S3

2. General considerations.………..……..….………………….………………….……….S6

3. Procedures for preparation of ligands and manganese catalysts.……..…….……..S6

4. Identificantion of enabling catalyst for benzyli coxidation with H_2O_2..…….........S16

5. Comparison of the catalytic activities of *rac*-**1** and *meso*-**1**………....……..........S18

6. Comparison of the catalytic activities of manganese catalysts.………......….........S22

7. Preparation and analytic data of substrates.…………………………………........S23

8. General procedure for the catalytic benzylic oxidation with H_2O_2……………....S46

9. Oxidation of tetiary amine at gram-scale.........................……….……………...S48

10. Analytic data of products.………………………………………………..….......S48

11. X-ray structures of manganese catalysts.……...…………….……………..…...S81

12. References.…..……………………. ………….…………………….………....S110

13. ^{1}H and ^{13}C NMR spectra.………….................................……..………S115

14. HPLC Traces.………..…………..……….…..…S311

1. The importantance of functionalized arene ketones, cyclic imines and amines

Figure S-1. Selected examples of functionalized arene ketones seen in drug and bioactive molecules[1]

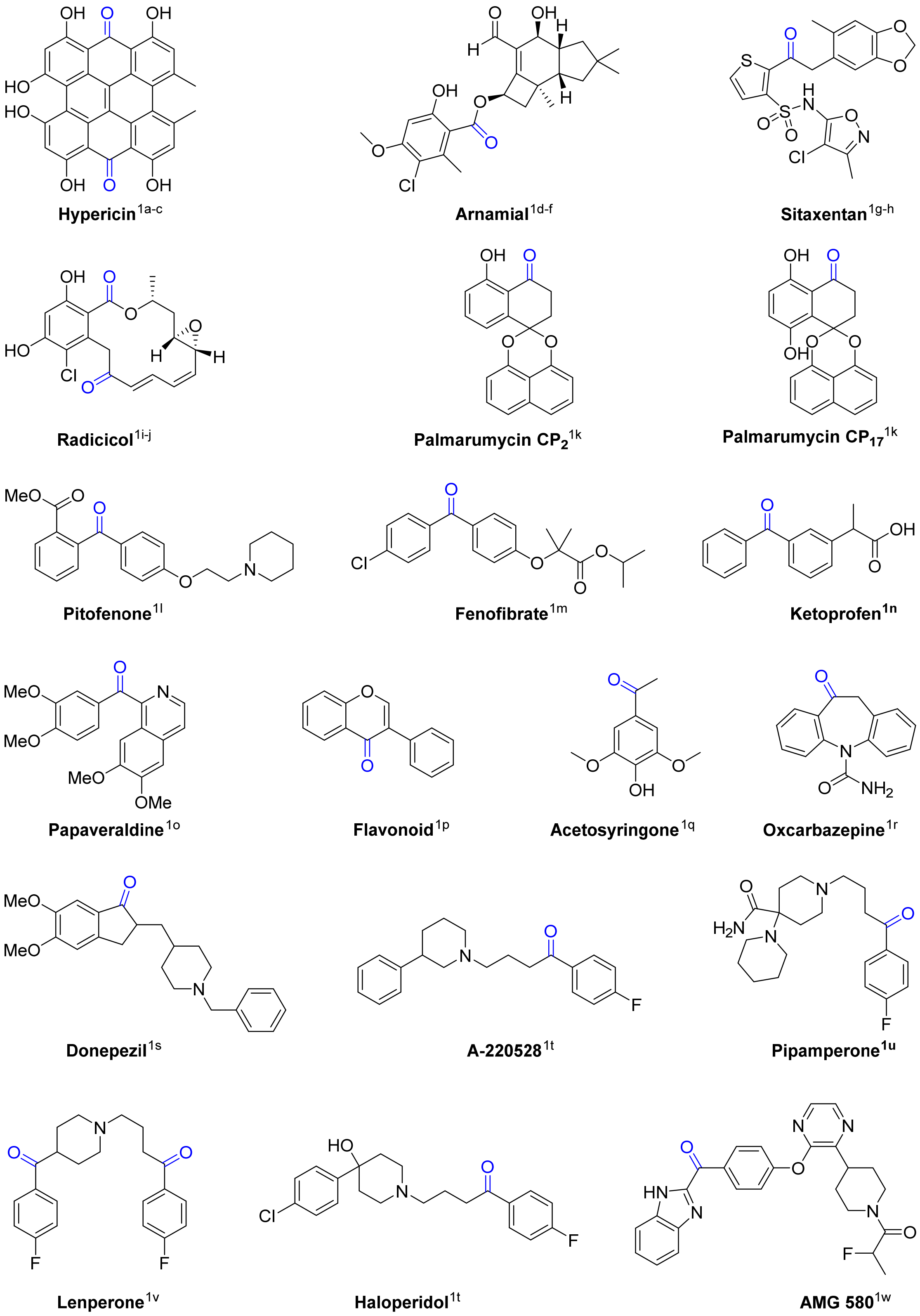

Figure S-2. Selected examples of cyclic imines and amines seen in drug and bioactive molecules[2]

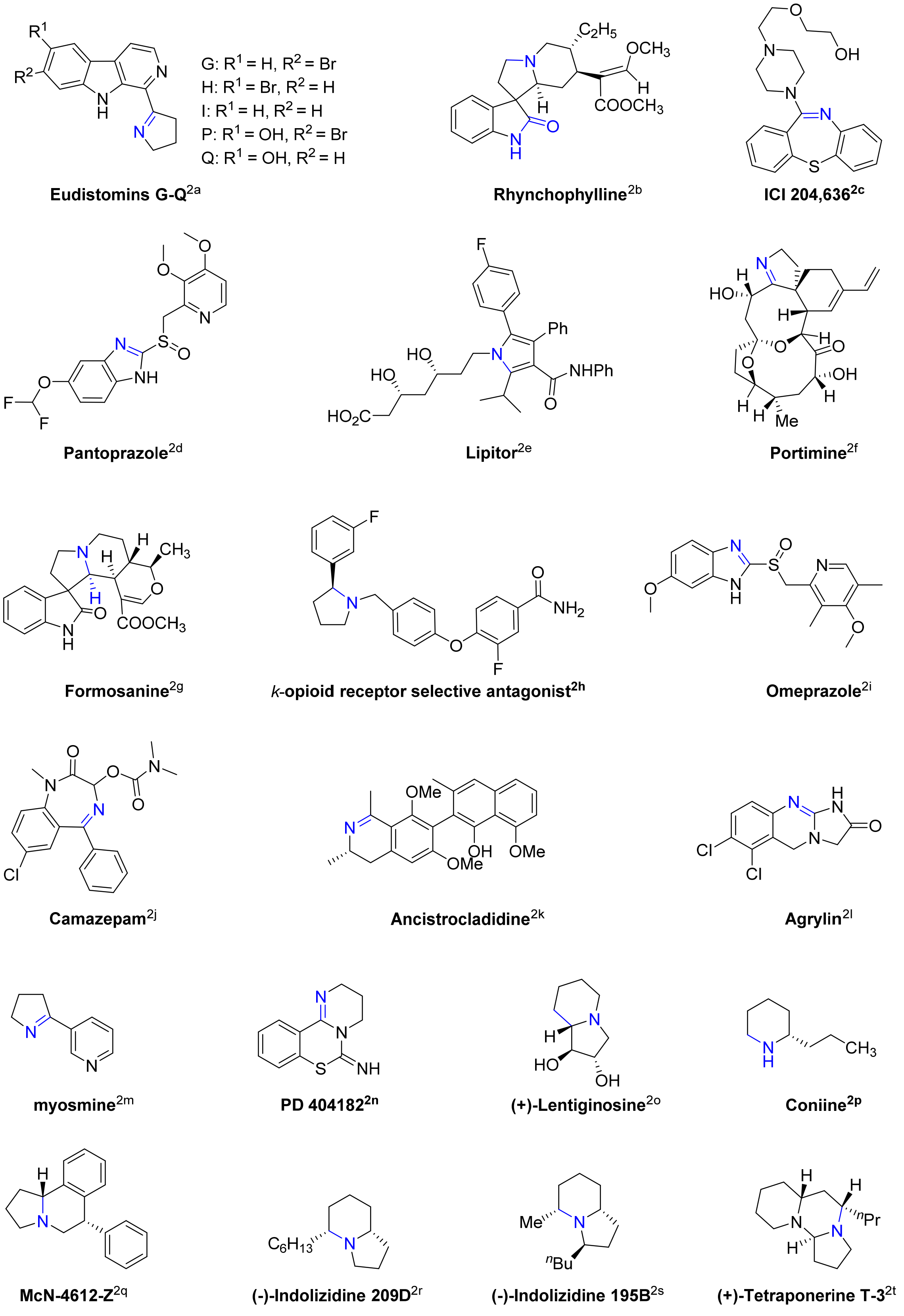

Figure S-3. Potential application of selected products from this and related study[3]

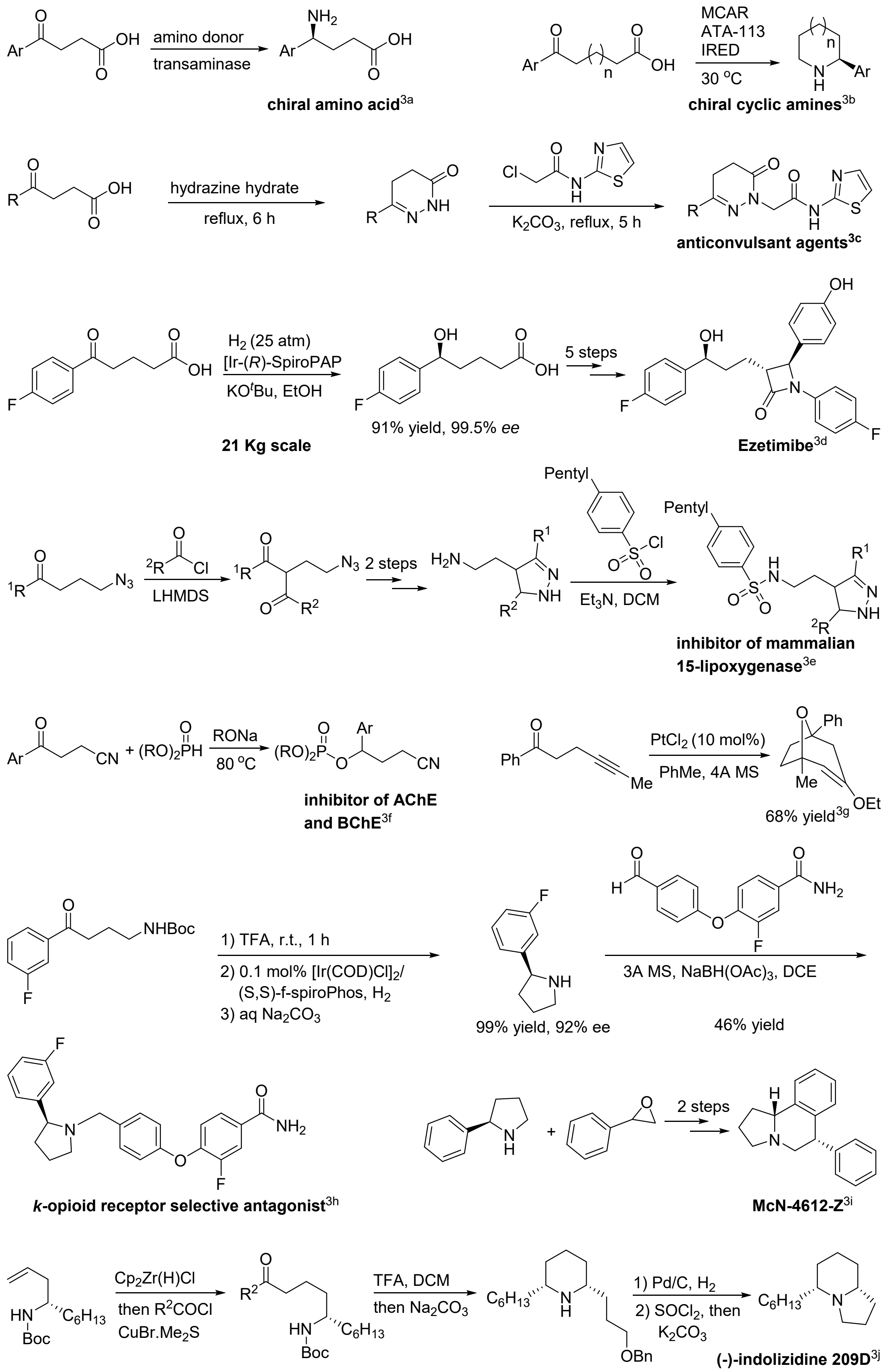

2. General considerations

The following commercially obtained reagents for the C-H oxidation reaction were used as received: H_2O_2 (30 wt% in H_2O, Sinopharm), $H_2{}^{18}O_2$ (3 wt% in $H_2{}^{18}O$, 90 atom% ^{18}O, Sigma-Aldrich), $H_2{}^{18}O$ (98 atom% ^{18}O, Energy Chemical), $^{18}O_2$ (97 atom% ^{18}O, Wuhan Newradar Special Gas Co. Ltd.), AcOH (Sinopharm), CH_3CN (Sinopharm), bipiperidine (Bide Pharmatech Ltd. China), (*R*,*R*)-bipiperidine and (*S*,*S*)-bipiperidine (Daicel Chiral Technologies Co. Ltd., China). All oxidation reactions were run under air with no precautions taken to exclude moisture. Commercial grade solvents used in the synthesis of components were used without further purification. Chemicals employed in the synthesis of ligands and substrates were purchased from commercial suppliers and used without further purification. ^1H NMR spectra were recorded on a Bruker Advance 400 (400 MHz) NMR spectrometer and reported in units of parts per million (ppm) relative to tetramethyl silane (δ 0 ppm) or $CDCl_3$ (δ 7.26 ppm) or DMSO-d_6 (δ 2.50 ppm). Multiplicities are given as: bs (broad singlet), s (singlet), d (doublet), t (triplet), q (quartet), dd (doublets of doublet), dt (doublets of triplet) or m (multiplet). ^{13}C NMR spectra were recorded on a Bruker Advance 400 (100 MHz) NMR spectrometer and reported in ppm relative to $CDCl_3$ (δ 77.0 ppm) or DMSO-d_6 (δ 40.0 ppm). Coupling constants were reported as a *J* value in Hz. Mass spectra were obtained by electrospray ionisation (ESI) at the Analytical Services of the Chemistry Department, Shannxi Normal University.

3. Procedures for preparation of ligands and manganese catalysts

3.1 Procedure for preparation of ligands

A. Synthesis of *rac*-L^1 and *meso*-L^1 [4a,4b]

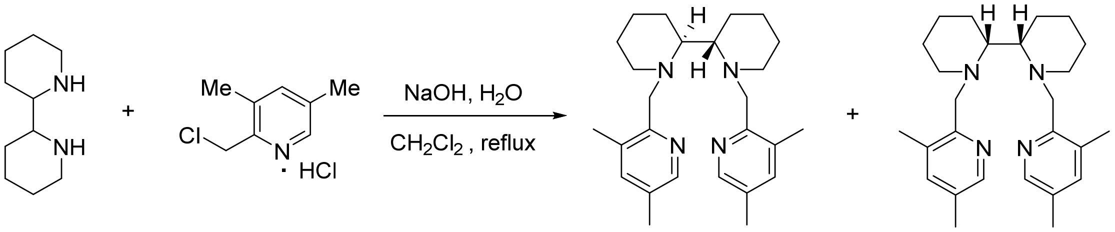

rac-**L^1**
38% yield

meso-**L1**
20% yield

***rac/meso*-1,1'-Bis((3,5-dimethylpyridin-2-yl)methyl)-2,2'-bipiperidine** was prepared according to the following procedure: To a solution of 2,2'-bipiperidine (335 mg, 2.0 mmol, 1 equiv.) and 2-(chloromethyl)-3,5-dimethylpyridine hydrochloride

(806 mg, 4.2 mmol, 2.1 equiv.) in DCM (20.0 mL), sodium hydroxide (400 mg, 10 mmol, 5 equiv.) in water (5.0 mL) was added dropwise for 5 minutes at 0 °C. Then, the resulting biphasic mixture was stirred for 5 days under reflux. When the starting material disappeared based on TLC analysis, the reaction mixture was diluted with DCM (20 mL) and transferred to a separatory funnel. Afterwards, 20.0 mL H_2O was added, and the mixture was extracted with DCM (3×20.0 mL). The combined organic layers were then dried over Na_2SO_4, and filtered. After concentration, the residue was purified by flash column chromatography on silica gel (gradient elution: petroleum ether/ethyl acetate/triethyl amine = 50:3:0.5-10:1:0.1) to give the racemic *rac*-**L**^{1} (310 mg, 38% yield) and the meso analogue *meso*-**L**^{1} (163 mg, 20% yield), both as a white solid.

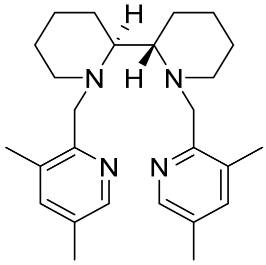

***rac*-(±)-1,1'-Bis((3,5-dimethylpyridin-2-yl)methyl)-2,2'-bipiperidine (*rac*-L^1)** ^{1}**H NMR** (400 MHz, $CDCl_3$) δ (ppm): 8.20 (s, 2H), 7.19 (s, 2H), 4.18 (d, *J* = 12.4 Hz, 2H), 3.25 (d, *J* = 12.4 Hz, 2H), 2.63 (t, *J* = 10.8 Hz, 4H), 2.36 (s, 6H), 2.24 (s, 6H), 2.02-1.94 (m, 4H), 1.67-1.64 (m, 2H), 1.47-1.35 (m, 6H), 1.16-1.10 (m, 2H); ^{13}**C NMR** (100 MHz, $CDCl_3$) δ (ppm): 154.4, 146.6, 138.5, 132.10, 131.06, 63.8, 59.1, 54.2, 25.7, 24.6, 24.3, 18.7, 17.8; **HRMS** (ESI) m/z calcd for $C_{26}H_{38}N_4Na$ $[M+Na]^+$: 429.2989; found: 429.2987.

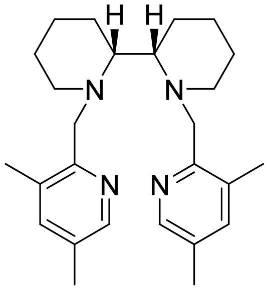

***meso*-(±)-1,1'-Bis((3,5-dimethylpyridin-2-yl)methyl)-2,2'-bipiperidine (*meso*-L^1)** ^{1}**H NMR** (400 MHz, $CDCl_3$) δ (ppm): 8.16 (s, 2H), 7.21 (s, 2H), 4.17 (d, *J* = 12.4 Hz, 2H), 3.67 (d, *J* = 12.4 Hz, 2H), 2.82 (s, 2H), 2.66-2.59 (m, 2H), 2.32 (s, 6H), 2.32-2.30

(m, 2H), 2.26 (s, 6H), 1.74-1.68 (m, 2H), 1.59-1.53 (m, 4H), 1.25-1.10 (m, 2H), 1.09-1.07 (m, 2H), 0.96-0.93 (m, 2H); **^{13}C NMR** (100 MHz, $CDCl_3$) δ (ppm): 155.4, 145.9, 138.4, 132.6, 131.0, 58.5, 55.7, 48.7 29.5, 21.2, 21.0, 18.1, 17.7; **HRMS** (ESI) *m/z* calcd for $C_{26}H_{38}N_4Na$ $[M+Na]^+$: 429.2989; found: 429.2988.

B. Synthesis of (*R*,*R*)-L$^{1\text{-}5}$ according to the following procedure[4]

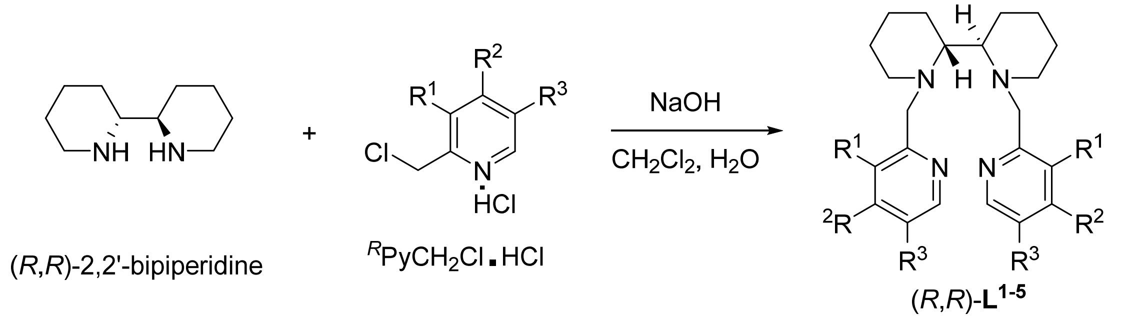

To a solution of (*R*,*R*)-2,2'-bipiperidine (134 mg, 0.8 mmol, 1 equiv.) and $^{R}PyCH_2Cl.HCl$ (1.7 mmol, 2.1 equiv.) in DCM (5.0 mL), sodium hydroxide (160 mg, 4 mmol, 5 equiv.) in water (2.0 mL) was added dropwise for 5 minutes at 0 °C. Then, the resulting biphasic mixture was stirred rapidly for 5 days in reflux when the starting material disappeared based on the TLC. Afterwards, DCM (10.0 mL) and H_2O (10.0 mL) were added, and the reaction mixture was extracted with DCM (3×10.0 mL). The combined organic layers were then dried over Na_2SO_4, and filtered. After concentration, the residue was purified by flash column chromatography on silica gel (gradient elution: petroleum ether/ethyl acetate/triethyl amine = 50:3:0.5-10:1:0.1) to give the desired ligand (*R*,*R*)-**L^1**, (*R*,*R*)-**L^2**, (*R*,*R*)-**L^3**, (*R*,*R*)-**L^4**, or (*R*,*R*)-**L^5**, abbreviated as (*R*,*R*)-**L^1**, **L^2**, **L^3**, **L^4**, and **L^5**, respectively.

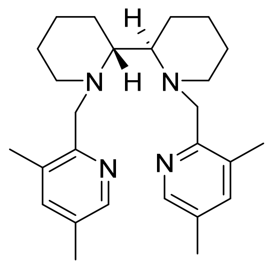

(*R*,*R*)-1,1'-Bis((3,5-dimethylpyridin-2-yl)methyl)-2,2'-bipiperidine ((*R*,*R*)-**L^1**) was synthesized according to the above procedure as a yellow solid (253 mg, 78 % yield); **^{1}H NMR** (400 MHz, $CDCl_3$) δ (ppm): 8.22 (s, 2H), 7.22 (s, 2H), 4.19 (d, *J* = 12.4 Hz, 2H), 3.28 (d, *J* = 12.4 Hz, 2H), 2.66 (t, *J* = 10.8 Hz, 4H), 2.38 (s, 6H), 2.26 (s, 6H), 2.05-1.96 (m, 4H), 1.69-1.66 (m, 2H), 1.49-1.34 (m, 6H), 1.18-1.12 (m, 2H); **^{13}C NMR**

(100 MHz, $CDCl_3$) δ (ppm): 154.4, 146.6, 138.5, 132.10, 131.06, 63.8, 59.1, 54.2, 25.7, 24.6, 24.3, 18.7, 17.8.

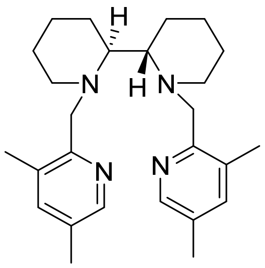

(*S*,*S*)-1,1'-Bis((3,5-dimethylpyridin-2-yl)methyl)-2,2'-bipiperidine ((*S*,*S*)-$\mathbf{L^1}$) was synthesized according to the above procedure, but the diamine was (*S*,*S*)-2,2'-bipiperidine (134 mg, 0.8 mmol). (*S*,*S*)-$\mathbf{L^1}$ obtained as a yellow solid (244 mg, 75% yield); ^{1}**H NMR** (400 MHz, $CDCl_3$) δ (ppm): 8.22 (s, 2H), 7.22 (s, 2H), 4.19 (d, *J* = 12.4 Hz, 2H), 3.27 (d, *J* = 12.4 Hz, 2H), 2.65 (t, *J* = 10.8 Hz, 4H), 2.38 (s, 6H), 2.26 (s, 6H), 2.05-1.96 (m, 4H), 1.69-1.66 (m, 2H), 1.49-1.34 (m, 6H), 1.18-1.12 (m, 2H); ^{13}**C NMR** (100 MHz, $CDCl_3$) δ (ppm): 154.4, 146.6, 138.5, 132.10, 131.06, 63.8, 59.1, 54.2, 25.7, 24.6, 24.3, 18.7, 17.8.

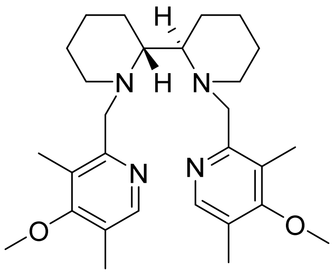

(*R*,*R*)-1,1'-Bis((4-methoxy-3,5-dimethylpyridin-2-yl)methyl)-2,2'bipiperidine ($\mathbf{L^2}$) was synthesized according to the above procedure as a yellow solid (291 mg, 78% yield); ^{1}**H NMR** (400 MHz, $CDCl_3$) δ (ppm): 8.17 (s, 2H), 4.16 (d, *J* = 12.4 Hz, 2H), 3.17 (s, 6H), 3.26 (d, *J* = 12.4 Hz, 2H), 2.64-2.60 (m, 4H), 2.31 (s, 6H), 2.20 (s, 6H), 2.03-1.90 (m, 4H), 1.66-1.63 (m, 2H), 1.46-1.30 (m, 6H), 1.14-1.02 (m, 2H); ^{13}**C NMR** (100 MHz, $CDCl_3$) δ (ppm): 163.6, 157.6, 148.5, 125.7, 124.3, 63.9, 59.8, 59.6, 54.2, 25.7, 24.6, 24.3, 13.0, 10.9; **HRMS** (ESI) m/z calc. for $C_{28}H_{42}N_4O_2Na$ $[M+Na]^+$: 489.3200; found: 489.3200.

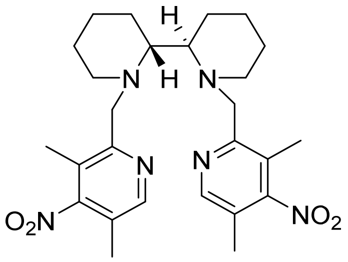

(*R*,*R*)-1,1'-Bis((3,5-dimethyl-4-nitropyridin-2-yl)methyl)-2,2'-bipiperidine (L^3) was synthesized according to the above procedure as a yellow solid (278 mg, 70 % yield); **^{1}H NMR** (400 MHz, $CDCl_3$) δ (ppm): 8.39 (s, 2H), 4.20 (d, *J* = 12.8 Hz, 2H), 3.44 (d, *J* = 12.8 Hz, 2H), 2.69-2.66 (m, 2H), 2.62-2.60 (m, 2H), 2.33 (s, 6H), 2.25 (s, 6H), 2.11-2.04 (m, 2H), 1.85-1.82 (m, 2H), 1.72-1.64 (m, 2H), 1.52-1.33 (m, 6H), 1.15-1.05 (m, 2H); **^{13}C NMR** (100 MHz, $CDCl_3$) δ (ppm): 158.4, 157.9, 148.7, 122.5, 121.4, 63.2, 59.7, 54.2, 25.5, 24.7, 24.1, 13.9, 12.8; **HRMS** (ESI) m/z calc. for $C_{26}H_{36}N_6O_4Na$ $[M+Na]^+$: 519.2690, found: 519.2695.

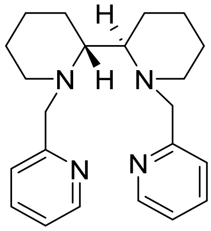

(*R*,*R*)-1,1'-Bis(pyridin-2-ylmethyl)-2,2'-bipiperidine (L^4)[4b] was synthesized according to the above procedure as a pale yellow solid (202 mg, 72% yield); **^{1}H NMR** (400 MHz, $CDCl_3$) δ (ppm): 8.53 (d, *J* = 4.4 Hz, 2H), 7.62 (td, *J* = 7.6, 1.8 Hz, 2H), 7.44 (d, *J* = 7.6 Hz, 2H), 7.13 (dd, *J* = 6.6, 4.8 Hz, 2H), 4.25 (d, *J* = 14.0 Hz, 2H), 3.21 (d, *J* = 14.4 Hz, 2H), 2.81 (d, *J* = 11.6 Hz, 2H), 2.71 (d, *J* = 10.4 Hz, 2H), 2.02-1.92 (m, 4H), 1.75-1.72 (m, 2H), 1.57-1.39 (m, 6H), 1.24-1.16 (m, 2H); **^{13}C NMR** (100 MHz, $CDCl_3$) δ (ppm): 160.2, 149.0 136.3, 122.6, 121.6, 62.2, 59.5, 54.5, 25.6, 24.8, 24.7. **HRMS** (ESI) m/z calcd for $C_{22}H_{30}N_4Na$ $[M+Na]^+$: 373.2363, found 373.2357.

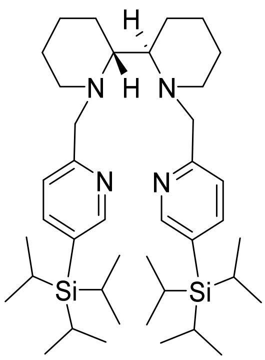

(*R*,*R*)-1,1'-Bis((5-triisopropylsilyl-2-yl)methyl)-2,2'-bipiperidine (L^5) was synthesized according to the above procedure as a white solid (381 mg, 72% yield); **^{1}H NMR** (400 MHz, $CDCl_3$) δ (ppm): 8.60 (s, 2H), 7.73 (dd, *J* = 7.6, 1.6 Hz, 2H), 7.43 (d, *J* = 7.6 Hz, 2H), 4.27 (d, *J* = 14.4 Hz, 2H), 3.20 (d, *J* = 14.4 Hz, 2H), 2.87 (d, *J* = 11.2 Hz, 2H), 2.71 (d, *J* = 11.2 Hz, 2H), 2.04-1.95 (m, 4H), 1.74 (d, *J* = 12.8 Hz, 2H), 1.55-1.48 (m, 5H), 1.44-1.37 (m, 7H), 1.08 (d, *J* = 7.5 Hz, 38H); **^{13}C NMR** (100 MHz, $CDCl_3$) δ (ppm): 160.2, 154.7, 143.1, 127.1, 122.0, 62.5, 59.8, 54.9, 25.5, 24.7 18.4, 10.6; **HRMS** (ESI) m/z calcd for $C_{40}H_{70}N_4Si_2Na$ $[M+H]^+$: 663.5212; found: 663.5214.

C. Synthesis of (*S*,*S*)-$L^{6,7}$ according to the following procedure[4a,4c,4d]

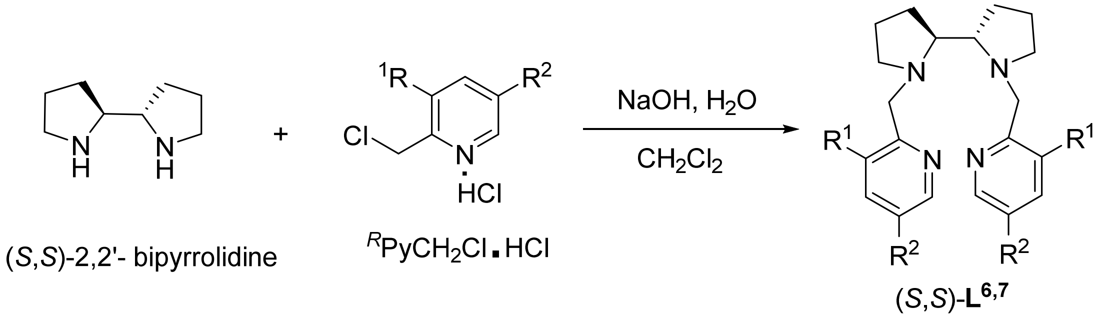

To a solution of (*S*,*S*)-2,2'-bipyrrolidine (112 mg, 0.8 mmol, 1 equiv.) and ^{R}PyCH$_2$Cl.HCl (1.7 mmol, 2.1 equiv.) in DCM (5.0 mL), sodium hydroxide (160 mg, 4 mmol, 5 equiv.) in water (2.0 mL) was added dropwise for 5 minutes at 0 °C. Then, the resulting biphasic mixture was stirred rapidly for 18 hours in reflux until the starting material disappeared based on the TLC. Afterwards, DCM (10.0 mL) and H_2O (10.0 mL) were added, and the reaction mixture was extracted with DCM (3×10.0 mL). The combined organic layers were then dried over Na_2SO_4, and filtered. After concentration, the residue was purified by flash column chromatography on silica gel (gradient elution: petroleum ether/ethyl acetate/triethyl amine = 20:1:0.5-10:1:0.1) to give the desired ligand of (*S*, *S*)-**L**^{6}, or (*S*, *S*)-**L**^{7}, abbreviated as **L**^{6} or **L**^{7}.

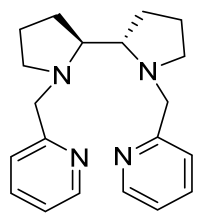

(*S*,*S*)-1,1'-Bis(pyridin-2-ylmethyl)-2,2'-bipyrrolidine (**L**[6])[4a] was synthesized according to the above procedure as a colorless liquid (183 mg, 71% yield); **^{1}H NMR** (400 MHz, $CDCl_3$) δ (ppm): 8.50 (d, *J* = 4.4 Hz, 2H), 7.60 (t, *J* = 7.6, 1.6 Hz, 2H), 7.39 (d, *J* = 7.6 Hz, 2H), 7.11 (dd, *J* = 6.8, 4.0 Hz, 2H), 4.19 (d, *J* = 14.0 Hz, 2H), 3.50 (d, *J* = 14.0 Hz, 2H), 3.02-2.98 (m, 2H), 2.80-2.78 (m, 2H), 2.24-2.20 (m, 2H), 1.83-1.68 (m, 8H); **^{13}C NMR** (100 MHz, $CDCl_3$) δ (ppm): 160.4, 148.8, 136.3, 122.7, 121.6, 65.4, 61.1, 55.3, 26.0, 23.6; HRMS (ESI) m/z calcd for $C_{20}H_{27}N_4$ [M+H]$^+$: 323.2230, found: 323.2236.

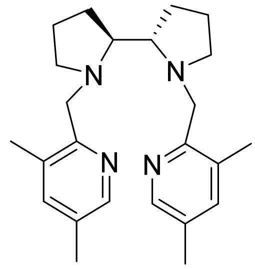

(*S*,*S*)-1,1'-Bis((3,5-dimethylpyridin-2-yl)methyl)-2,2'-bipyrrolidine (**L**[7])[4d] was synthesized according to the above procedure as a yellow liquid (230 mg, 76% yield); **^{1}H NMR** (400 MHz, $CDCl_3$) δ (ppm): 8.16 (s, 2H), 7.23 (s, 2H), 4.08 (d, *J* = 12.0 Hz, 2H), 3.38 (d, *J* = 12.0 Hz, 2H), 2.75-2.71 (m, 2H), 2.62-2.59 (m, 2H), 2.37 (s, 6H), 2.27 (s, 6H), 2.25-2.20 (m, 2H), 1.71-1.51 (m, 8H); **^{13}C NMR** (100 MHz, $CDCl_3$) δ (ppm): 154.8, 146.1, 138.5, 132.3, 131.4, 65.1, 60.3, 55.4, 25.9, 24.0, 18.2, 17.9; **HRMS** (ESI) m/z calcd for $C_{24}H_{35}N_4$ [M+H]$^+$: 379.2856, found: 379.2845.

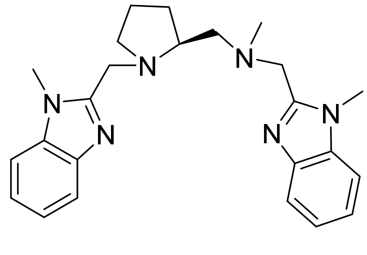

(*S*)-*N*-Methyl-1-(1-methyl-1*H*-benzo[*d*]imidazol-2-yl)-*N*-((1-((1-methyl-1*H* benzo[*d*]imidazol-2-yl)methyl)pyrrolidin-2-yl)methyl)methanamine (**L**[8])[4e] was synthesized according to the literature procedure as a yellow liquid; **^{1}H NMR** (400

MHz, $CDCl_3$) δ (ppm): 7.69-7.64 (m, 2H), 7.23-7.17 (m, 6H), 4.22 (d, *J* = 13.6 Hz, 1H), 3.74 (s, 3H), 3.72 (d, *J* = 6.4 Hz, 1H), 3.67 (d, *J* = 13.6 Hz, 1H), 3.63 (d, *J* = 6.4 Hz, 1H), 3.60 (s, 3H), 2.74-2.64 (m, 2H), 2.46 (dd, *J* = 12.4, 4.4 Hz, 1H), 2.34 (dd, *J* = 12.0, 7.6 Hz, 1H), 2.27 (dd, *J* = 16.8, 9.2 Hz, 1H), 2.21 (s, 3H), 1.62-1.51 (m, 2H), 1.49-1.42 (m, 1H); **^{13}C NMR** (100MHz, $CDCl_3$) δ 152.2, 151.6, 142.0, 136.1, 136.0, 122.4, 122.2, 121.8, 121.7, 119.4, 119.3, 109.0, 108.9, 62.5, 61.9, 55.9, 54.8, 52.2, 43.1, 30.3, 29.9, 29.8, 22.5.

3.2 Procedure for preparation of manganese catalysts[4]

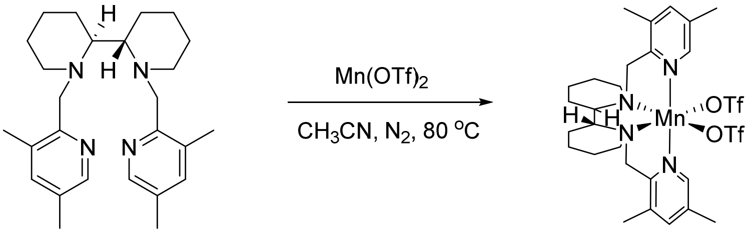

rac-**L1** *rac*-**1**

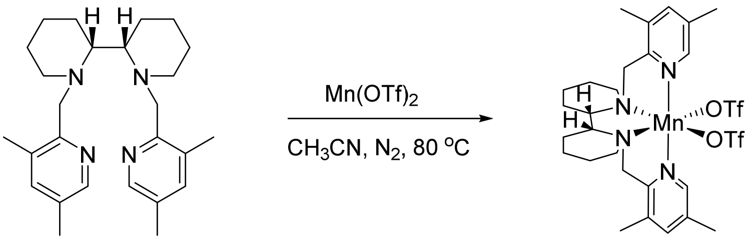

meso-**L1** *meso*-**1**

(*rac* or *meso*)-1,1'-Bis((3,5-dimethylpyridin-2-yl)methyl)-2,2'-bipiperidine (0.5 mmol, 203 mg) and $Mn(OTf)_2$ (0.5 mmol, 177 mg) were placed in an oven-dried, resealable Schlenk tube. The tube was capped with a Teflon screwcap, evacuated, and backfilled with nitrogen. The screw cap was replaced with a rubber septum, and dry CH_3CN (4.0 mL) was added via a syringe. The tube was purged with nitrogen for 1-2 minutes, and then the septum was replaced with the Teflon screw cap. The tube was sealed, and the reaction mixture was heated at 80 °C overnight. Then the resulting mixture was cooled to room temperature, and the solvent CH_3CN was removed under reduced pressure to afford the desired manganese catalyst.

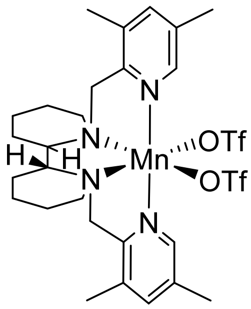

***rac*-1** was synthesized according to the above procedure as a pale yellow solid (368 mg, 97% yield); **HRMS** (ESI) m/z calcd for $C_{27}H_{38}F_{3}MnN_{4}O_{3}S$ [M-OTf]$^{+}$: 610.1997, found: 610.1993.

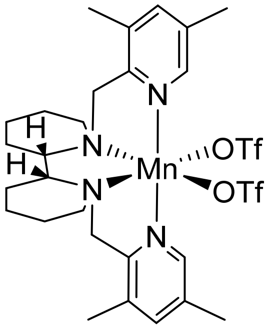

***meso*-1** was synthesized according to the above procedure as earthy yellow solid (365 mg, 96% yield); **HRMS** (ESI) m/z calcd for $C_{27}H_{38}F_{3}MnN_{4}O_{3}S$ [M-OTf]$^{+}$: 610.1997, found: 610.1988.

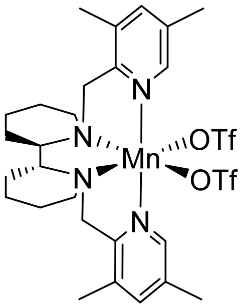

(*R*,*R*)-**1** was synthesized according to the above similar procedure as a pale yellow solid (364 mg, 96% yield); **HRMS** (ESI) m/z calcd for $C_{27}H_{38}F_{3}MnN_{4}O_{3}S$ [M-OTf]$^{+}$: 610.1997, found: 610.1973.

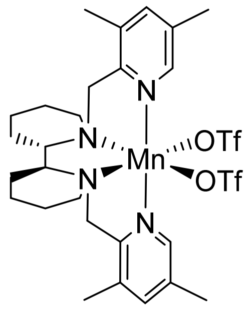

(*S*,*S*)-**1** was synthesized according to the above similar procedure as a pale yellow solid (368 mg, 97% yield); **HRMS** (ESI) m/z calcd for $C_{27}H_{38}F_3MnN_4O_3S$ [M-OTf]$^+$: 610.1997, found: 610.1972.

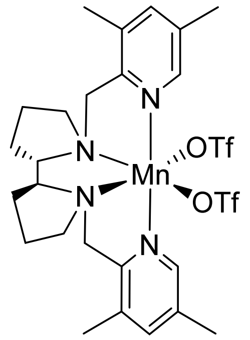

(*S*,*S*)-**(L[7])Mn(OTf)$_2$** was synthesized according to the above similar procedure as a pale yellow solid (351 mg, 96% yield); **HRMS** (ESI) m/z calcd for $C_{25}H_{34}F_3MnN_4O_3S$ [M-OTf]$^+$: 582.1684, found: 582.1683.

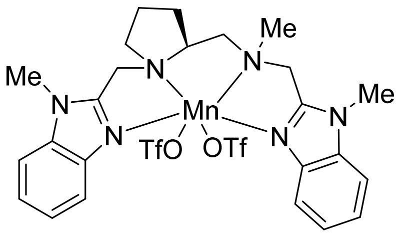

(*S*)-**(L[8])Mn(OTf)$_2$**[4e] was synthesized according to the above procedure as a pale yellow solid (370 mg, 98% yield); **HRMS** (ESI) m/z calcd for $C_{25}H_{30}F_3MnN_6O_3S$ [M-OTf]$^+$: 606.1433, found: 606.1404.

The X-ray structures of *rac*-**1** and the aqua complexes of *rac*-**1** and *meso*-**1** have been determined and are presented in Section 11. The aqua complexes were formed during crystallization of *rac*-**1** and *meso*-**1** due to water present in the solvent (see Section 11).

4. Identificantion of enabling catalyst for benzylic oxidation with H_2O_2

Table S-1. Benzylic oxidation with in-situ formed catalyst[a]

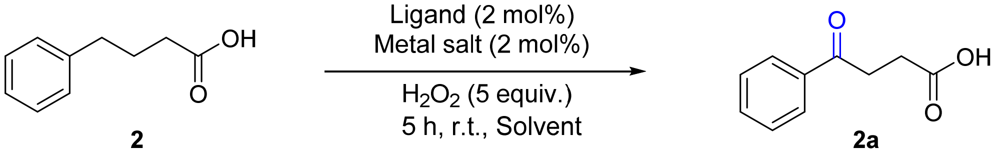

Entry	Ligand (mol %)	Metal salt or catalyst (mol %)	Solvent	Additive	Yield [b] (%)
1	-	$Mn(OTf)_2$	CH_3CN	-	0
2	-	**I**	CH_3CN	-	38
3	*rac*-**L1**	-	CH_3CN	-	0
4	*rac*-**L1**	$Mn(OTf)_2$	CH_3CN	-	90
5	*meso*-**L1**	$Mn(OTf)_2$	CH_3CN	-	9
6	**L2**	$Mn(OTf)_2$	CH_3CN	-	55
7	**L3**	$Mn(OTf)_2$	CH_3CN	-	7
8	**L4**	$Mn(OTf)_2$	CH_3CN	-	35
9	**L5**	$Mn(OTf)_2$	CH_3CN	-	28
10	**L6**	$Mn(OTf)_2$	CH_3CN	-	28
11	**L7**	$Mn(OTf)_2$	CH_3CN	-	80
12	**L1**	$MnCl_2$	CH_3CN	-	60
13	**L1**	$Mn(OAc)_2$	CH_3CN	-	75
14	**L1**	$Mn(OAc)_3.2H_2O$	CH_3CN	-	82
15	**L1**	$Fe(OTf)_2$	CH_3CN	-	45
16	**L1**	$Fe(OAc)_2$	CH_3CN	-	32
17	**L1**	$FeBr_2$	CH_3CN	-	36
18	**L1**	$Mn(OTf)_2$	CH_3CN	AcOH[c]	95
19	**L2**	$Mn(OTf)_2$	CH_3CN	AcOH[c]	93

[a] General conditions: **2** (0.5 mmol), ligand (2 mol%), metal salt (2 mol%), acetonitrile (3 mL), H_2O_2 (5 equiv., 2.5 mmol), room temperature in a reaction tube without nitrogen protection, 5 hours; [b] Yield determined by ^1H NMR of crude reaction mixture; [c] AcOH (5 equiv.), 1 hour.

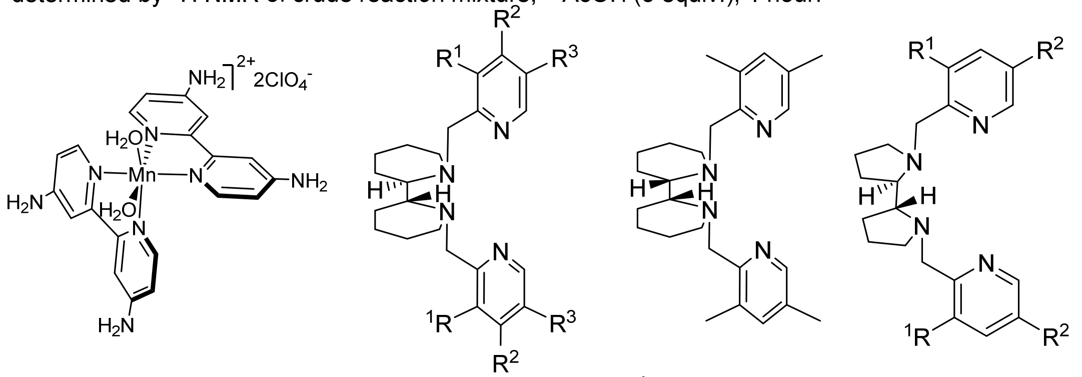

I

R^1,R^3 = Me, R^2 = H *rac*-**L1**
R^1,R^3 = Me, R^2 = OMe **L2**
R^1,R^3 = Me, R^2 = NO_2 **L3**
R^1,R^2,R^3 = H **L4**
R^1, R^2 = H, R^3 = $Si(^iPr)_3$ **L5**

meso-**L1**

R^1, R^2 = H, **L6**
R^1, R^2 = Me **L7**

Table S-2. Benzylic oxidation with pre-formed catalyst[a]

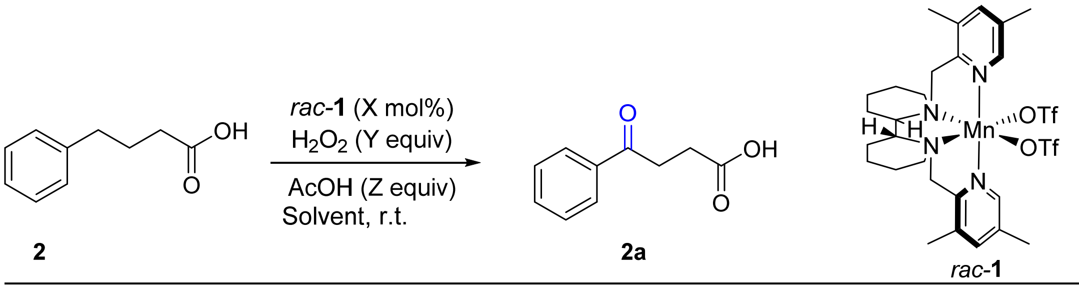

Entry	**1** (X mol %)	H_2O_2 (Y equiv.)	Solvent	AcOH (Z equiv.)	Yield of [b] **2a** (%)
1	-	5	CH_3CN	5	0
2[c]	2.0	5	CH_3CN	5	8
3	2.0	5	CH_3CN	5	96
4	1.5	5	CH_3CN	5	88
5	1.0	5	CH_3CN	5	75
6	0.5	5	CH_3CN	5	42
7	2.0	5	Benzene	5	31
8	2.0	5	Toluene	5	30
9	2.0	5	DCM	5	38
10	2.0	5	Acetone	5	83
11	2.0	5	EtOAc	5	35
12	2.0	5	CH_3OH	5	12
13	2.0	5	H_2O	5	32
14	2.0	4	CH_3CN	5	90
15	2.0	3	CH_3CN	5	77
16	2.0	2	CH_3CN	5	68
17	2.0	1	CH_3CN	5	39
18	2.0	0	CH_3CN	5	0
19	2.0	5	CH_3CN	10	96
20	2.0	5	CH_3CN	3	93
21	2.0	5	CH_3CN	1	91
22	2.0	5	CH_3CN	0	62
23	2.0	5	CH_3CN	0	91[d]
24	2.0	5	CH_3CN	5	50[e]

[a] General conditions:**2** (1 equiv., 0.5 mmol),*rac*-**1** (X mol%), H_2O_2 (Y equiv.), solvent (3 mL), room temperature in a reaction tube without nitrogen protection, 1 hour; [b] Yield determined by ^1H NMR of crude reaction mixture; [c] *meso*-**1** as catalyst [d] 5 hours; [e] 2-oxo-2-phenylacetic acid (10 mol%) added

Table S-3. Optimization of conditions for benzylic oxidation of primary amines

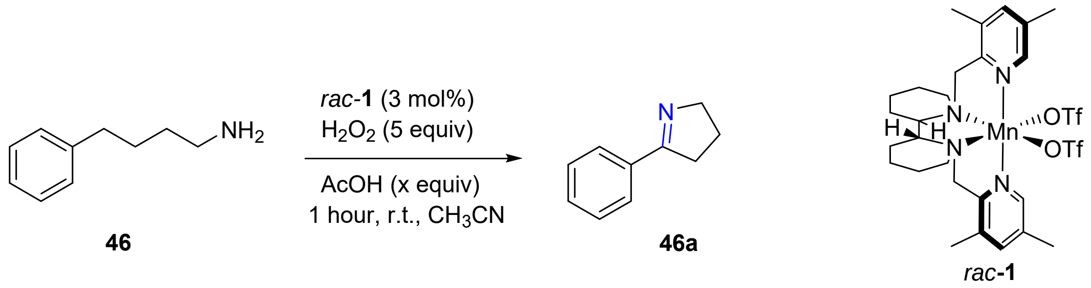

Entry	*rac*-**1** (mol %)	H_2O_2 (equiv.)	Solvent	AcOH (X equiv.)	Yield of **46a** (%)[b]
1	2	5	CH_3CN	5 equiv	23
2	3	5	CH_3CN	5 equiv	35
3	3	5	CH_3CN	10 equiv	58
4	3	5	CH_3CN	0.5 mL (35 equiv.)	76
5	3	5	CH_3CN	1.5 mL (105 equiv.)	99
6	3	5	CH_3CN	1.5 mL (105 equiv.)	93[c]

[a] General conditions: **46** (1 equiv., 0.5 mmol), *rac*-**1** (3 mol%), AcOH (X equiv.) were dissolved in MeCN (1.5 mL), and then H_2O_2 (5 equiv., 2.5 mmol) in 1.5 mL of MeCN was delivered by syringe pump over 1 hour stirring at room temperature without nitrogen protection. [b] Yield determined by ^1H NMR of crude reaction mixture; [c] Isolated yield.

5. Comparison of the catalytic activities of *rac*-1 and *meso*-1

To a solution of ***rac*-1** or ***meso*-1** (2 mol%), ethyl benzene (**18**) (0.5 mmol) and AcOH (2.5 mmol, 5 equiv.) in MeCN (1.5 mL), H_2O_2 (2.5 mmol, 5 equiv., 30 wt.% aqueous solution dissolved in 1.0 mL MeCN) was added via a syringe pump over 1 hour at room temperature. During this time, samples were taken every ten minutes and analyzed by GC to determine the conversion, using biphenyl as internal standard. The results are shown in **Table S-4** and plotted in **Figure S-4**. The color change observed in the two reactions is shown in **Figure S-5**.

Table S-4. Results obtained in the oxidation of ethyl benzene

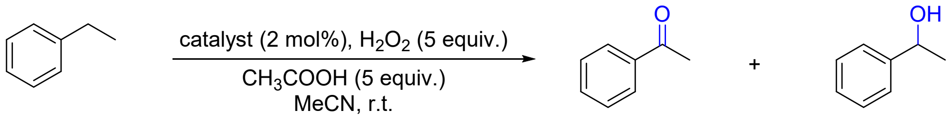

18   18a   18a'

Entry	catalyst		Time (minutes)	Yield of **18a** (%)	Yield of **18a'** (%)	Conversion of **18** (%)
1	*rac*-**1**	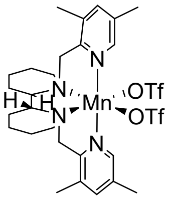	10	23.6	3.8	27.4
2	*rac*-**1**		20	58.6	4.2	62.8
3	*rac*-**1**		30	77.8	4.4	82.2
4	*rac*-**1**		40	88.8	3.8	92.6
5	*rac*-**1**		50	91.2	2.2	93.4
6	*rac*-**1**	*rac*-**1**	60	94.3	2.0	96.3
7	*meso*-**1**	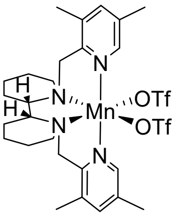	10	1.3	1.3	2.6
8	*meso*-**1**		20	1.8	1.5	3.3
9	*meso*-**1**		30	1.8	1.6	3.4
10	*meso*-**1**		40	1.8	1.7	3.5
11	*meso*-**1**		50	1.8	1.7	3.5
12	*meso*-**1**	*meso*-**1**	60	1.8	1.7	3.5

General reaction conditions: **18** (0.5 mmol), *rac*-**1** or *meso*-**1** (2 mol%), and AcOH (2.5 mmol) were dissolved in MeCN (1.5 mL), and then H_2O_2 (2.5 mmol) in MeCN (1.5 mL) was delivered by syringe pump over 1 h under stirring at room temperature without nitrogen protection; yield determined by GC of crude reaction mixture.

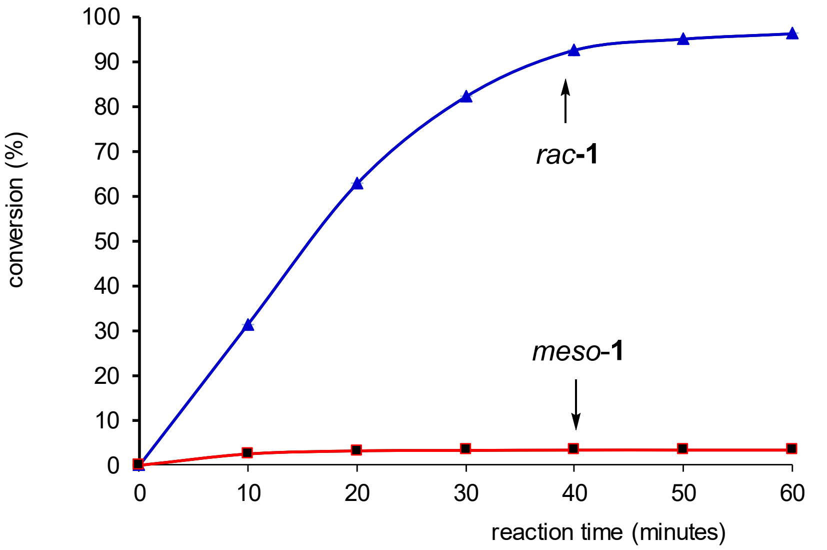

Figure S-4. The conversion-time course for the oxidation of **18** to **18a** (▲ *rac*-**1**, ■ *meso*-**1**) based on data in Table S-4. The alcohol **18a′** is oxidized to **18a**, as indicated by the data in Table S-5.

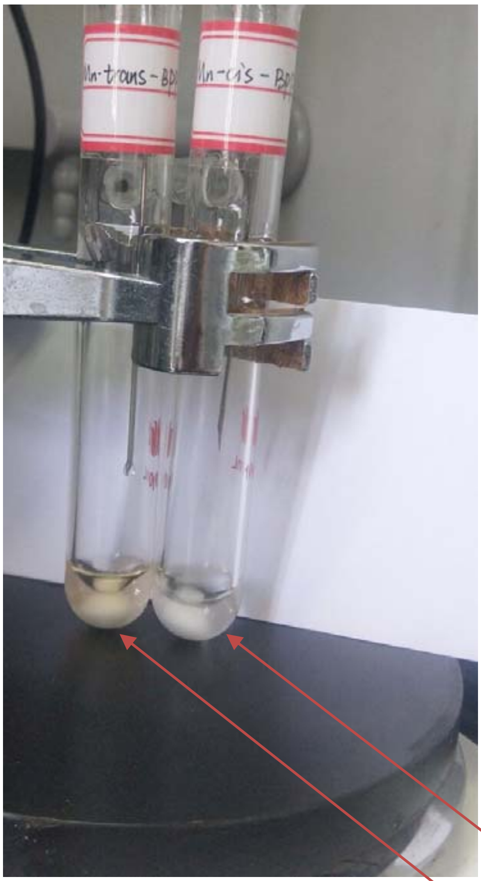

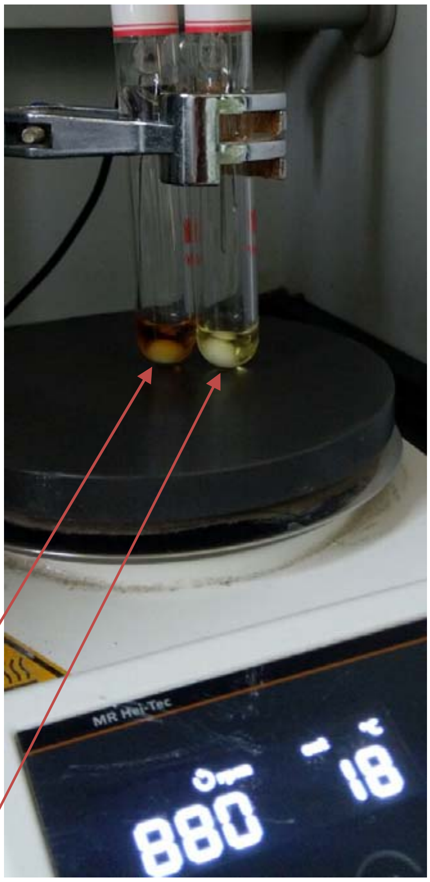

Figure S-5a. Before reaction (***rac*-1**, ***meso*-1**) **Figure S-5b**. 15 minutes later

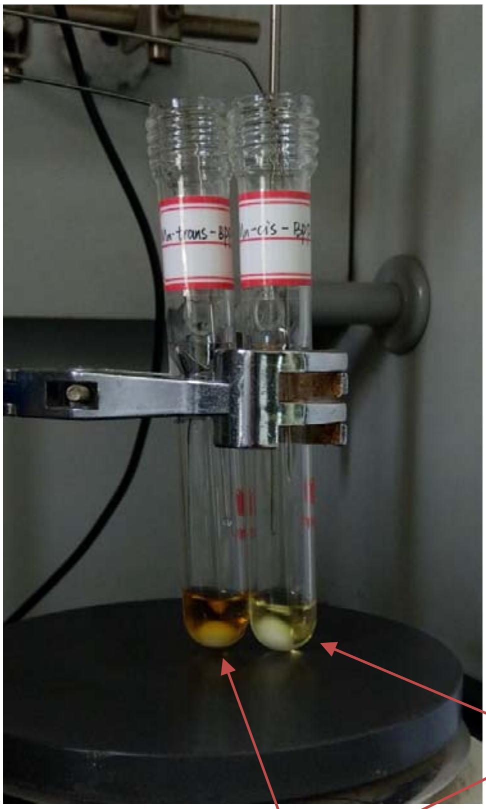

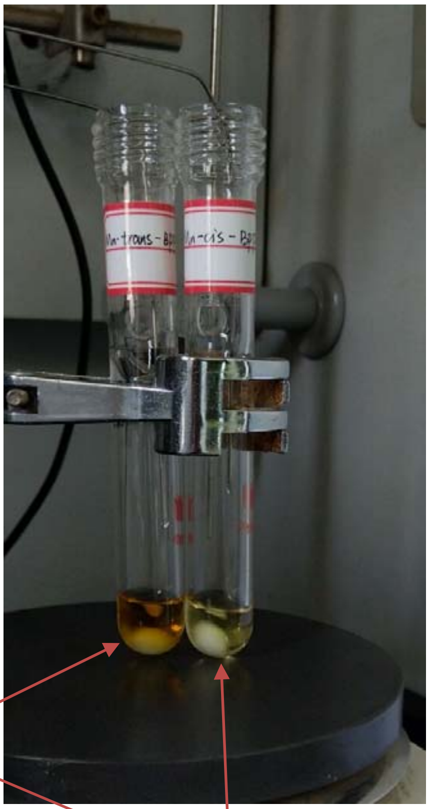

Figure S-5c. 30 minutes later **Figure S-5d**. 60 minutes later

(reaction on the left: *rac*-**1** as a catalyst; reaction on the right: *meso*-**1** as a catalyst)

Table S-5. Results obtained in the oxidation of alcohol **18a′**

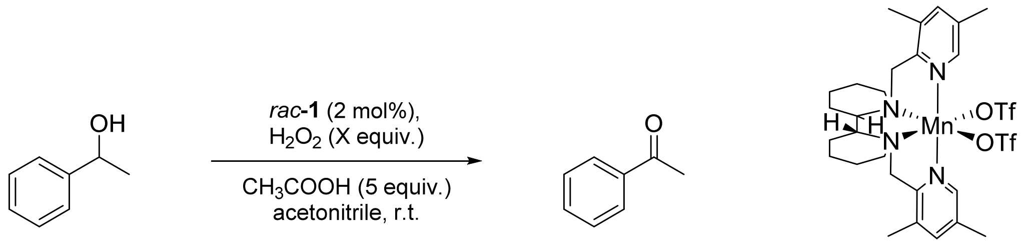

18a' **18a** *rac*-**1**

Entry	catalyst	Time (minutes)	H_2O_2 (X equiv)	Yield of **18a** (%)	remaining **18a'** (%)
1	*rac*-**1**	10	2.5	10	90
2	*rac*-**1**	30	2.5	72	28
3	*rac*-**1**	60	2.5	98	2
4	*rac*-**1**	10	5.0	72	28
5	*rac*-**1**	20	5.0	90	10
6	*rac*-**1**	30	5.0	98	2
7	*rac*-**1**	40	5.0	100	0

General reaction conditions: **18a'** (0.5 mmol, 1.0 equiv), *rac*-**1** (2 mol%), and AcOH (2.5 mmol, 5 equiv) were dissolved in MeCN (1.5 mL), and then H_2O_2 (X equiv) in MeCN (1.5 mL) was delivered by syringe pump over 1 h under stirring at room temperature without nitrogen protection; GC yield.

6. Comparison of the catalytic activities of manganese catalysts

Table S-6. Oxidation of benzylic compounds with *rac*-**1**, (*S*,*S*)-**(L⁷)Mn(OTf)₂** and (*S*)-**(L⁸)Mn(OTf)₂**

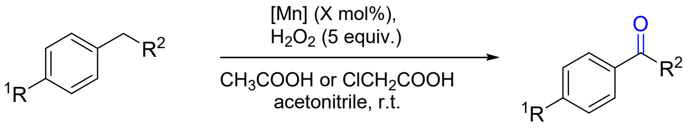

Entry	Substrate	Reaction conditions	Product	Yield of product (%) *rac*-**1**	(*S*,*S*)-**(L⁷)Mn(OTf)₂**	(*S*)-**(L⁸)Mn(OTf)₂**
1	***2***	**A**	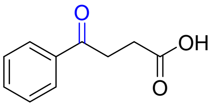	96[a]	80[a]	N/A
2	**15**	**A**	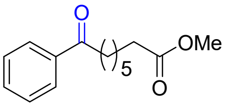	92	86	84
3	**18**	**A**	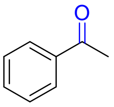	94[b]	N/A	68[c]
4	**35**	**A**	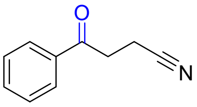	94	75	41
5	**38**	**A**	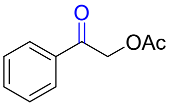	44 (76[d])	32	18
6	**45**	**A**	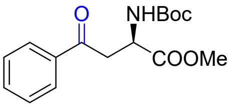	93	67	40
7	**46**	**B**	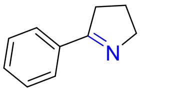	93	48	38
8	**88**	**C**	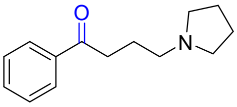	80	41	45
9	**89**	**C**	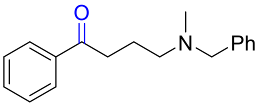	86	55	62

Reaction conditions: conditions **A:** same as in Figure 4 (article); conditions **B:** same as in Figure 6 (article); conditions **C:** same as in Figure 7 (article); isolated yield given; N/A: unavailable; [a] ^1H NMR yield; [b] GC yield; [c] data based on reference 4e, GC yield; [d] HOAc (15.0 equiv).

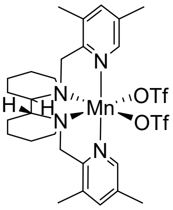

rac-**1**

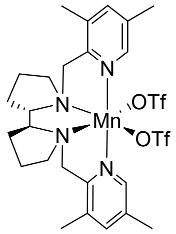

(*S*,*S*)-**(L⁷)Mn(OTf)₂**

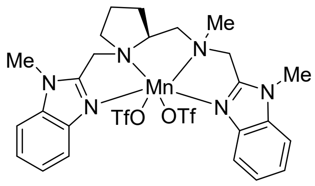

(*S*)-**(L⁸)Mn(OTf)₂**

7. Preparation and analytic data of substrates

7.1 Synthesis and characterization of methyl 8-phenyloctanoate (15) [5]

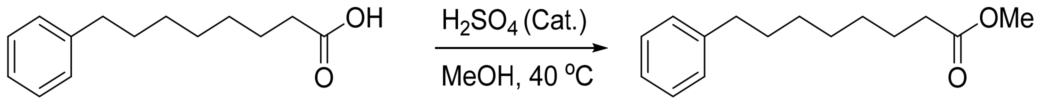

To a solution of 8-phenyloctanoic acid (1.1 g, 5 mmol) in a mixture of DCM (20.0 mL) and MeOH (3.0 mL) at 0 °C, H_2SO_4 (50 mg, 0.5 mmol, 98 wt%) was added dropwise to the stirring reaction over 60s. Then, the reaction mixture was stirred for 4 hours at 40 °C. After addition of DCM (20.0 mL) and H_2O (10.0 mL), the reaction mixture was extracted with DCM (3×20.0 mL). The combined organic layers were then dried over Na_2SO_4, and filtered. After concentration, the residue was purified by flash column chromatography on silica gel (gradient elution: petroleum ether/ethyl acetate = 200:1-100:1) to give **methyl 8-phenyloctanoate** (1.1 g, 95% yield). **^{1}H NMR** (400 MHz, $CDCl_3$) δ (ppm): 7.28-7.23 (m, 2H), 7.17-7.15 (m, 3H), 3.65 (s, 3H), 2.59 (t, J = 7.8 Hz, 2H), 2.29 (t, J = 7.6 Hz, 2H), 1.62-1.59 (m, 4H), 1.32-1.28 (m, 6H); **^{13}C NMR** (100 MHz, $CDCl_3$) δ (ppm): 174.3, 142.8, 128.4, 128.3, 125.6, 51.5, 36.0, 34.1, 31.5, 29.2, 29.1 (2C), 25.0.

7.2 Synthesis of 8-phenyloctanamide (16)[6]

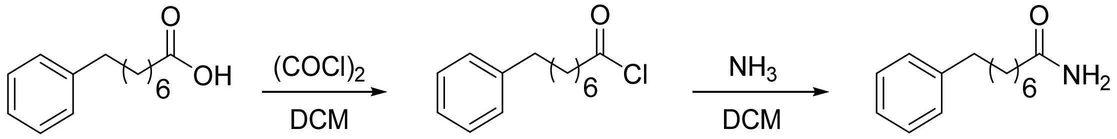

Synthesis of 8-phenyloctanoyl chloride

To a solution of 8-phenyloctanoic acid (1.1 g, 5 mmol) and DMF (10 µL) in dry dichloromethane (20 mL) at 0 °C, oxalyl chloride (0.85 mL, 10 mmol) was slowly added to this solution and the reaction mixture was warmed up to room temperature and stirred for 2 hours. After concentration, the generated acid chloride **8-phenyloctanoyl chloride** was directly used for next step without any further purification.

Synthesis of 8-phenyloctanamide

To a solution of the above synthesized 8-phenyloctanoyl chloride (5 mmol) in DCM (10.0 mL) at 0 °C, ammonia in methanol (2 mol/L, 5 mL) was added dropwise to the

stirring reaction over 5 minutes. Then the reaction mixture was allowed to warm to room temperature and stirred for 2 hours. After addition of 15.0 mL DCM and 15.0 mL H_2O, the reaction mixture was extracted with DCM (3x15 mL). The combined organic layers were then dried over Na_2SO_4, and filtered. After concentration, the residue was purified by flash column chromatography on silica gel (gradient elution: petroleum ether/ethyl acetate = 20:1-10:1) to give **8-Phenyloctanamide** (986 mg, 90% yield). **^{1}H NMR** (400 MHz, $CDCl_3$) δ (ppm): 7.29-7.25 (m, 2H), 7.18-7.16 (m, 3H), 5.98 (brs, 1H), 5.53 (brs, 1H), 2.59 (t, *J* = 7.6 Hz, 2H), 2.19 (t, *J* = 7.6 Hz, 2H), 1.62-1.60 (m, 4H), 1.33-1.26 (m, 6H); **^{13}C NMR** (100 MHz, $CDCl_3$) δ (ppm): 176.0, 142.8, 128.4, 128.3, 125.6, 36.0, 35.9, 31.5, 29.2 (2C), 29.1, 25.5.

7.3 Synthesis 1-phenyloctan-4-one (33) and 1-phenylnonan-5-one (34)[7a]

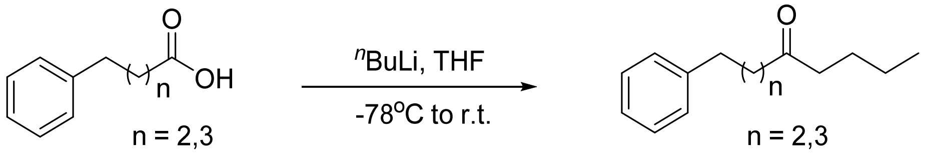

To a solution of 4-phenylbutanoic acid (820 mg, 5 mmol) or 5-phenylpentanoic acid (890 mg, 5 mmol) in dry anhydrous THF (20.0 mL) under nitrogen atmosphere at -78 °C , *n*-butyllithium (5 mL, 2.5 M in hexane, 12.5 mmol) was added via a syringe pump over 20 minutes. This solution was stirred at -78 °C for another 30 minutes. Then the reaction was allowed to warm to room temperature and stirred overnight. The reaction was quenched with saturated NH_4Cl aqueous solution. The aqueous layer was extracted with ethyl acetate. The combined organic layers were washed with brine, dried by $MgSO_4$, filtered, and concentrated. The crude product was purified by flash chromatography on silica gel (gradient elution: petroleum ether/ethyl acetate = 20:1-10:1) to give **33** and **34**

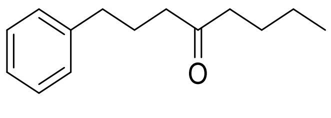

1-Phenyloctan-4-one (33) [7b] was synthesized according to the above procedure as colorless liquid (562 mg, 55% yield); **^{1}H NMR** (400 MHz, $CDCl_3$) δ (ppm): 7.30-7.25 (m, 2H), 7.20-7.16 (m, 3H), 2.61 (t, *J* = 7.6 Hz, 2H), 2.42-2.35 (m, 4H), 1.94-1.87 (m, 2H), 1.57-1.50 (m, 2H), 1.31-1.26 (m, 2H), 0.89 (t, *J* = 6.8 Hz, 3H); **^{13}C NMR** (100

MHz, $CDCl_3$) δ (ppm): 211.1, 141.7, 128.5, 128.4, 125.9, 42.6, 41.9, 35.1, 26.0, 25.3, 22.4, 13.9.

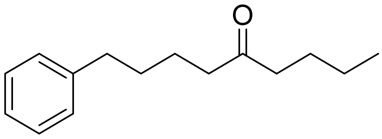

1-Phenylnonan-5-one (34) was synthesized according to the above procedure as colorless liquid (797 mg, 73% yield); **^{1}H NMR** (400 MHz, $CDCl_3$) δ (ppm): 7.33-7.29 (m, 2H), 7.21-7.19 (m, 3H), 2.64 (t, *J* = 7.6 Hz, 2H), 2.45-2.39 (m, 4H), 1.65-1.62 (m, 4H), 1.60-1.56 (m, 2H), 1.37-1.31 (m, 2H), 0.93 (t, *J* = 6.8 Hz, 3H); **^{13}C NMR** (100 MHz, $CDCl_3$) δ (ppm): 211.4, 142.3, 128.4, 128.3, 125.7, 42.6, 42.5, 35.8, 31.1, 26.0, 23.5, 22.4, 13.9.

7.4 Synthesis of (4-azidobutyl)benzene (36)[8]

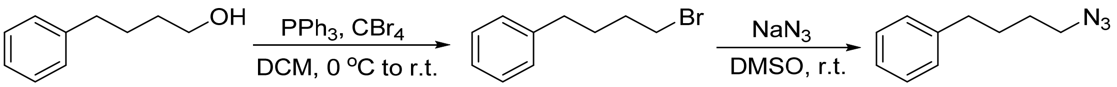

(4-Bromobutyl)benzene[8a] was prepared according to the following procedure: To a solution of 4-phenyl butanol (750 mg, 5 mmol) and CBr_4 (1.71 g, 5.2 mmol) in dry DCM (15.0 mL) at 0 °C, triphenylphosphine (1.38 g, 5.25 mmol) was slowly added to this solution and the reaction mixture was warmed up to room temperature and stirred for 3 hours. After addition of 15.0 mL DCM and 15.0 mL H_2O, the reaction mixture was extracted with DCM (3×15.0 mL). The combined organic layers were then dried over Na_2SO_4, and filtered. After concentration, the residue was purified by flash column chromatography on silica gel (gradient elution: petroleum ether/ethyl acetate = 20:1-10:1) to give (**4-bromobutyl)benzene**.

(4-Azidobutyl)benzene[8b] was prepared according to the following procedure: To a solution of the above synthesized (4-bromobutyl)benzene (848 mg, 4 mmol) in DMSO (25.0 mL), NaN_3 (260 mg, 4 mmol) was added to this solution and the reaction mixture was stirred for 18 hours at room temperature. After the reaction was complete, water (30.0 mL) and diethyl ether (30.0 mL) were added. The organic layer was separated, and the aqueous layer was extracted with diethyl ether (60.0 mL) three times. The combined organic layers were washed with water (120.0 mL) four times， and dried over Na_2SO_4. After concentration, the residue was purified by flash column chromatography on silica gel (gradient elution: petroleum ether/ethyl acetate = 20:1-

10:1) to give **(4-azidobutyl)benzene** (596 mg, 85% yield). **^{1}H NMR** (400 MHz, $CDCl_3$) δ (ppm): 7.39-7.35 (m, 2H), 7.29-7.25 (m, 3H), 3.34 (t, *J* = 6.8 Hz, 2H), 2.72 (d, *J* = 7.2 Hz, 2H), 1.83-1.67 (m, 4H); **^{13}C NMR** (100 MHz, $CDCl_3$) δ (ppm): 141.8, 128.4 (2C), 125.9, 51.3, 35.4, 28.5 (2C).

7.5 Synthesis of 3-phenylpropyl acetate (37)[9]

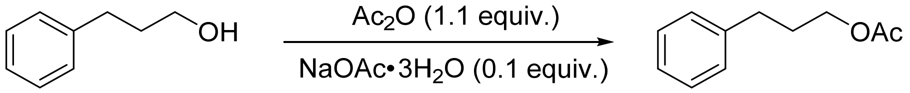

3-Phenylpropyl acetate was prepared according to the following procedure: A mixture of phenylpropanol (680 mg, 5 mmol), acetic anhydride (520 μL, 5.5 mmol), and $NaOAc{\cdot}3H_2O$ (68 mg, 0.5 mmol) was dissolved in DCM (15.0 mL) and the reaction mixture was stirred for 15 hours. After the reaction was complete, saturated solution of $NaHCO_3$ (10.0 mL) and diethyl ether (10.0 mL) were added to the reaction mixture. The organic layer was separated, and the aqueous layer was extracted with diethyl ether (30.0 mL) three times. The combined organic layers were washed with brine and dried over Na_2SO_4. After concentration, the residue was purified by flash column chromatography on silica gel (gradient elution: petroleum ether/ethyl acetate = 10:1-5:1) to give **3-phenylpropyl acetate** (788 mg, 89% yield).**^{1}H NMR** (400 MHz, $CDCl_3$) δ (ppm): 7.29-7.25 (m, 2H), 7.19-7.16 (m, 3H), 4.07 (t, *J* = 6.6 Hz, 2H), 2.67 (t, *J* = 7.6 Hz, 2H), 2.03 (s, 3H), 1.98-1.90 (m, 2H); **^{13}C NMR** (100 MHz, $CDCl_3$) δ (ppm): 171.1, 141.2, 128.4 (2C), 126.0, 63.8, 32.1, 30.2, 20.9.

7.6 Synthesis of 4-phenylbutyl methanesulfonate (39)[10]

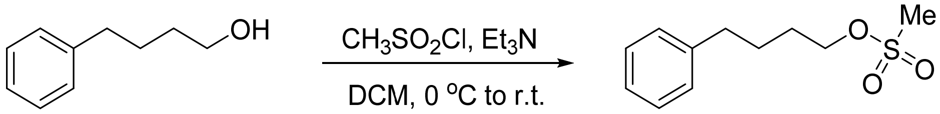

(4-(Methylsulfonyl)butyl)benzene was prepared according to the following procedure: To a solution of 4-phenylbutanol (750 mg, 5 mmol) and triethylamine (1.38 mL, 10 mmol) in dry dichloromethane (15.0 mL) at 0 °C, methanesulfonyl chloride (464 μL, 6 mmol) was slowly added to this solution and the reaction mixture was warmed up to room temperature and stirred for 2 hours. After the reaction was complete, water (15.0 mL) were added added to the reaction mixture. The organic layer was separated, and the aqueous layer was extracted with dichloromethane (3×15.0 mL) three times. The combined organic layers were washed with brine and dried over Na_2SO_4. After

concentration, the residue was purified by flash column chromatography on silica gel (gradient elution: petroleum ether/ethyl acetate = 10:1-5:1) to give **4-Phenylbutyl methanesulfonate** (1.05 g, 92% yield). **^{1}H NMR** (400 MHz, $CDCl_3$) δ (ppm): 7.34-7.31 (m, 2H), 7.25-7.21 (m, 3H), 4.26 (t, J = 6.0 Hz, 2H), 2.99 (s, 3H), 2.70 (t, J = 7.0 Hz, 2H), 1.83-1.77 (m, 4H); **^{13}C NMR** (100 MHz, $CDCl_3$) δ (ppm): 141.5, 128.4 (2C), 125.9, 69.9, 37.2, 35.1, 28.6, 27.1.

7.7 Synthesis of 1-(10,11-Dihydro-5*H*-dibenzo[*b,f*]azepin-5-yl)ethanone (42)[11]

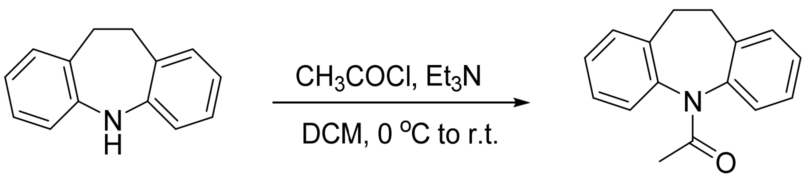

1-(10,11-Dihydro-5*H*-dibenzo[*b*, *f*]azepin-5-yl)ethan-1-one was prepared according to the following procedure: To a solution of 10,11-dihydro-5*H*-dibenzo[*b*, *f*]azepine (975 mg, 5 mmol) and triethylamine (1.38 mL, 10 mmol) in dry dichloromethane (20.0 mL) at 0 °C, acetyl chloride (424 µL, 6 mmol) was slowly added to this solution and the reaction mixture was warmed up to room temperature and stirred for 12 hours. After the reaction was complete, water (20.0 mL) were added to the reaction mixture. The organic layer was separated, and the aqueous layer was extracted with dichloromethane (60.0 mL) three times. The combined organic layers were washed with brine and dried over Na_2SO_4. After concentration, the residue was purified by flash column chromatography on silica gel (gradient elution: petroleum ether/ethyl acetate = 10:1-4:1) to give **1-(10,11-dihydro-5*H*-dibenzo[*b*, *f*]azepin-5-yl)ethan-1-one** (948 mg, 80% yield). **^{1}H NMR** (400 MHz, $CDCl_3$) δ (ppm): 7.36-7.14 (m, 8H), 3.44-3.28 (m, 2H), 2.86-2.78 (m, 2H), 2.01 (s, 3H); **^{13}C NMR** (100 MHz, $CDCl_3$) δ (ppm): 170.5, 142.6, 140.0, 137.4, 134.5, 130.6, 129.8, 128.6,128.5, 127.6, 127.4, 127.3, 126.5, 30.9, 30.2, 22.7.

7.8 Synthesis of 2-phenethylisoindoline-1,3-dione (43)[12]

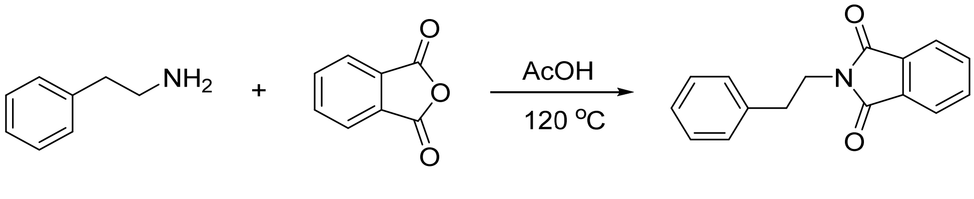

2-Phenethylisoindoline-1,3-dione was prepared according to the following procedure: A round flask was charged with phthalic anhydride (740 mg, 5 mmol), phenethylamine (605 mg, 5 mmol), and acetic acid (50.0 mL). The reaction mixture was heated to 120 °C and stirred for 24 hours. Then, most of acetic acid was removed, and dichloromethane (20.0 mL) and saturated solution of $NaHCO_3$ (20.0 mL) were added to the remained reaction mixture. The organic layer was separated, and the aqueous layer was extracted with dichloromethane (60.0 mL) three times. The combined organic layers were washed with brine and dried over Na_2SO_4. After concentration, the residue was purified by flash column chromatography on silica gel (gradient elution: petroleum ether/ethyl acetate = 10:1-4:1) to afford **2-phenethylisoindoline-1,3-dione** (1.13 g, 90% yield). **^{1}H NMR** (400 MHz, $CDCl_3$) δ (ppm): 7.83-7.81 (m, 2H), 7.71-7.68 (m, 2H), 7.30-7.21 (m, 5H), 3.92 (t, *J* = 7.8 Hz, 2H), 2.99 (t, *J* = 7.8 Hz, 2H); **^{13}C NMR** (100 MHz, $CDCl_3$) δ (ppm): 168.2, 138.1, 134.0, 132.1, 128.9, 128.6, 126.7, 123.3, 39.3, 34.7.

7.9 Synthesis of ethyl 2-(1,3-dioxoisoindolin-2-yl)-4-phenylbutanoate (44)[12,13]

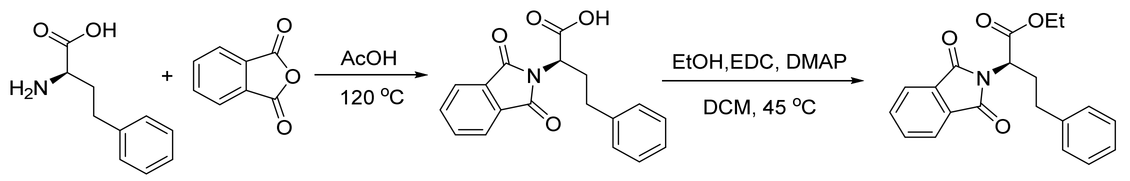

2-(1,3-Dioxoisoindolin-2-yl)-4-phenylbutanoic acid was prepared according to the following procedure: A round flask was charged with phthalic anhydride (740 mg, 5 mmol), (*R*)-2-amino-benzenebutanoic acid (895 mg, 5 mmol), and acetic acid (50.0 mL). The reaction mixture was heated to 120 °C and stirred for 24 hours. Then, most of acetic acid was removed, and dichloromethane (20.0 mL) and saturated solution of $NaHCO_3$ (20.0 mL) were added to the remained reaction mixture. The organic layer was separated, and the aqueous layer was extracted with dichloromethane (60.0 mL) three times. The combined organic layers were washed with brine and dried over Na_2SO_4. After concentration, the crude product **2-(1,3-dioxoisoindolin-2-yl)-4-phenylbutanoic acid** was obtained and used for the next step without further purification.

Ethyl 2-(1,3-dioxoisoindolin-2-yl)-4-phenylbutanoate (44) was prepared according to the following procedure: a round flask was charged with the above synthesized 2-

(1,3-dioxoisoindolin-2-yl)-4-phenylbutanoic acid (5 mmol, 1 equiv.), EDC [3-(ethyliminomethylideneamino)-*N*,*N*-dimethylpropan-1-amine, hydrochloride] (1.25 g, 6.5 mmol, 1.3 equiv.), DMAP (*N*,*N*-dimethylpyridin-4-amine) (153 mg, 1.25 mmol, 0.25 equiv.), and dichloromethane (20.0 mL). The reaction mixture was stirred rapidly and then EtOH (3.0 mL) was added. The reaction mixture was allowed to stir at room temperature overnight. After concentration, the residue was purified through flash column chromatography on silica gel (gradient elution: petroleum ether/ethyl acetate = 6:1-3:1) to give ethyl 2-(1,3-dioxoisoindolin-2-yl)-4-phenylbutanoate (1.26 g, 75% yield). **^{1}H NMR** (400 MHz, $CDCl_3$) δ (ppm): 7.85-7.83 (m, 2H), 7.735-7.71 (m, 2H), 7.22-7.06 (m, 5H), 4.87 (dd, *J* = 6.4, 4.0 Hz, 1H), 4.18 (q, *J* = 7.2 Hz, 2H), 2.69-2.57 (m, 4H), 1.21 (t, *J* = 7.2 Hz, 3H); **^{13}C NMR** (100 MHz, $CDCl_3$) δ (ppm): 169.2, 167.8, 140.4, 134.2, 131.9, 128.4, 126.1, 123.5, 61.9, 52.2, 32.8, 30.1, 14.1.

7.10 Synthesis of methyl 2-((*tert*-butoxycarbonyl)amino)-4-phenylbutanoate (45)[13]

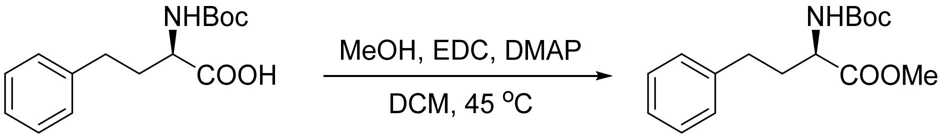

(2*R*)-2-((*tert*-Butoxycarbonyl)amino)-4-phenylbutyric acid methyl ester was prepared according to the following procedure: A round flask was charged with (*R*)-2-(*tert*-butoxycarbonylamino)-4-phenylbutanoic acid (837 mg, 3 mmol, 1 equiv.), EDC (3-(ethyliminomethylideneamino)-*N*,*N*-dimethylpropan-1-amine, hydrochloride) (748 mg, 3.9 mmol, 1.3 equiv.), DMAP (*N*,*N*-dimethylpyridin-4-amine) (92 mg, 0.75 mmol, 0.25 equiv.), and DCM (10.0 mL). The reaction mixture was stirred rapidly and then MeOH (2.0 mL) was added. The reaction mixture was allowed to stir at room temperature overnight. After concentration, the residue was purified through flash column chromatography on silica gel (gradient elution: petroleum ether/ethyl acetate = 8:1-4:1) to give methyl 2-(*tert*-butoxycarbonylamino)-4-phenylbutanoate (791 mg, 90% yield). **^{1}H NMR** (400 MHz, $CDCl_3$) δ (ppm): 7.20-7.17 (m, 2H), 7.11-7.07 (m, 3H), 5.11 (d, *J* = 6.4 Hz, 1H), 4.28-4.26 (m, 1H), 3.61 (s, 3H), 2.59 (t, *J* = 7.8 Hz, 2H), 2.08-2.03 (m, 1H), 1.90-1.83 (m, 1H), 1.36 (s, 9H); **^{13}C NMR** (100 MHz, $CDCl_3$) δ (ppm): 173.1, 155.4, 140.7, 128.4 (2C), 126.1, 79.8, 53.2, 52.2, 34.3, 31.6, 28.3.

7.11. Synthesis of 4-(4-fluorophenyl)butan-1-amine (47), 4-*p*-tolylbutan-1-amine (50), 4-(4-fluoro-3-methylphenyl)butan-1-amine (51), and 5-phenylpentan-1-amine (72)[6]

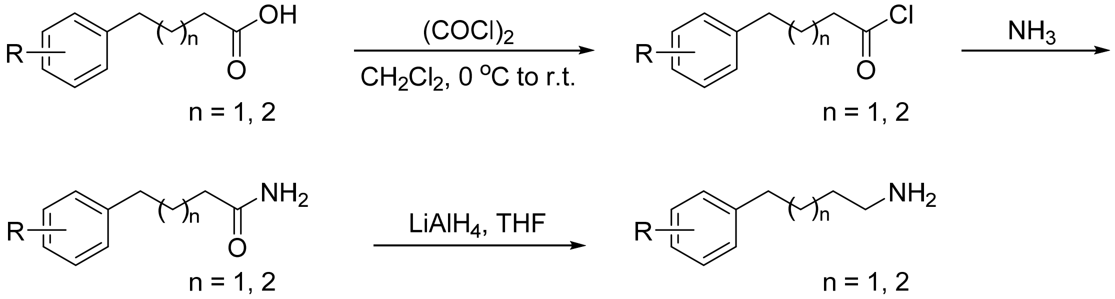

4-Arylbutanoyl chloride and **5-arylpentanoyl chloride** were prepared according to the following procedure: to a solution of 4-arylbutanoic acid (5 mmol) or 4-arylpentanoic acid (5 mmol) and DMF (10 μL) in dry dichloromethane (20 mL) at 0 °C, oxalyl chloride (0.85 mL, 10 mmol) was slowly added to this solution and the reaction mixture was warmed up to room temperature and stirred for 2 hours. After concentration, the generated acid chloride **4-arylbutanoyl chloride** or **5-arylpentanoyl chloride** was directly used for next step without any further purification.

4-Arylbutanamide and **5-arylpentanamide** were prepared according to the following procedure: to a solution of the above synthesized 4-arylbutanoyl chloride (5 mmol) or 5-arylpentanoyl chloride (5 mmol) in DCM (15 mL) at 0 °C, ammonia in MeOH (5 mL, 10 mmol, 2 mol/L) was slowly added to this solution and the reaction mixture was warmed up to room temperature and stirred for 2 hours. After the reaction was complete, water (20 mL) were added added to the reaction mixture. The organic layer was separated, and the aqueous layer was extracted with dichloromethane (60 mL) three times. The combined organic layers were washed with brine and dried over Na_2SO_4. After concentration, the crude product **4-arylbutanamide** or **5-arylpentanamide** was obtained and used for the next step without further purification.

4-Arylbutan-1-amine and **5-arylpentan-1-amine** was prepared according to the following procedure: The above synthesized 4-arylbutanamide or 5-arylpentanamide was dissolved in dry THF (15 mL) and placed in an ice bath under nitrogen atmosphere. $LiAlH_4$ (15 mmol, 570 mg) was added to this solution carefully. Then, the reaction mixture was warmed up to room temperature and heated to reflux for 4 hours. The reaction was quenched with water (1.0 mL), and the solution was stirred for an additional hour at room temperature. The mixture was filtered over $MgSO_4$, and the

solvent was removed to give the crude product, which was purified by flash column chromatography on silica gel (gradient elution: petroleum ether/ethyl acetate/triethyl amine = 10:1:1-3:1:0.3) to give **47, 50, 51, 72.**

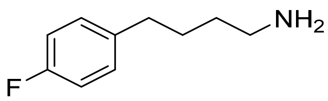

4-(4-Fluorophenyl)butan-1-amine (47) was synthesized according to the above procedure as colorless liquid (543 mg, 65% yield); **^{1}H NMR** (400 MHz, $CDCl_3$) δ (ppm): 7.13-7.10 (m, 2H), 6.97-6.93 (m, 2H), 2.70 (t, J = 7.0 Hz, 2H), 2.59 (t, J = 7.6 Hz, 2H), 1.66-1.58 (m, 2H), 1.50-1.42 (m, 2H); **^{13}C NMR** (100 MHz, $CDCl_3$) δ (ppm): 161.2 (d, J_{C-F} = 243.2 Hz), 138.1 (d, J_{C-F} = 3.3 Hz), 129.7 (d, J_{C-F} = 7.7 Hz), 115.0 (d, J_{C-F} = 21.1 Hz), 42.1, 35.0, 33.3, 28.9.

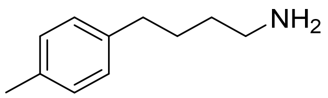

4-*p*-Tolylbutan-1-amine (50) was synthesized according to the above procedure as colorless liquid (546 mg, 67% yield); **^{1}H NMR** (400 MHz, $CDCl_3$) δ (ppm): 7.08 (s, 4H), 2.69 (t, J = 7.0 Hz, 2H), 2.59 (t, J = 7.2 Hz, 2H), 2.32 (s, 3H), 1.65-1.62 (m, 2H), 1.49-1.46 (m, 2H); **^{13}C NMR** (100 MHz, $CDCl_3$) δ (ppm): 139.0, 134.7, 128.7, 128.0, 41.6, 35.0, 32.8, 28.5, 20.7.

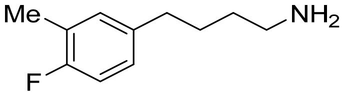

4-(4-Fluoro-3-methylphenyl)butan-1-amine (51) was synthesized according to the above procedure as colorless liquid (498 mg, 55% yield); **^{1}H NMR** (400 MHz, $CDCl_3$) δ (ppm): 7.00-6.86 (m, 3 H), 2.70 (t, J = 7.0 Hz, 2H), 2.55 (t, J = 7.6 Hz, 2H), 2.24 (s, 3H), 1.65-1.57 (m, 2H), 1.49-1.43 (m, 2H); **^{13}C NMR** (100 MHz, $CDCl_3$) δ (ppm): 159.8 (d, J_{C-F} = 240.3 Hz), 137.8 (d, J_{C-F} = 3.5 Hz), 131.3 (d, J_{C-F} = 4.9 Hz), 126.9 (d, J_{C-F} = 7.8 Hz), 124.3 (d, J_{C-F} = 17.1 Hz), 114.6 (d, J_{C-F} = 21.9 Hz), 42.1, 35.0, 33.4, 29.0, 14.6 (d, J = 3.5 Hz).

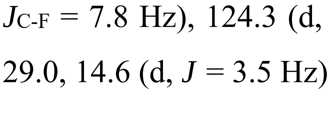

5-Phenylpentan-1-amine (72)[14] was synthesized according to the above procedure as colorless liquid (546 mg, 67% yield); **^{1}H NMR** (400 MHz, $CDCl_3$) δ (ppm): 7.29-

7.25 (m, 2H), 7.19-7.16 (m, 3H), 2.70 (t, J = 7.0 Hz, 2H), 2.62 (t, J = 7.6 Hz, 2H), 1.69-1.61 (m, 2H), 1.51-1.44 (m, 2H), 1.19-1.14 (m, 2H); **^{13}C NMR** (100 MHz, $CDCl_3$) δ (ppm): 142.7, 128.4, 128.2, 125.6, 42.1, 35.9, 33.6, 31.5, 26.6.

7.12 Synthesis of substrate 52-66[15]

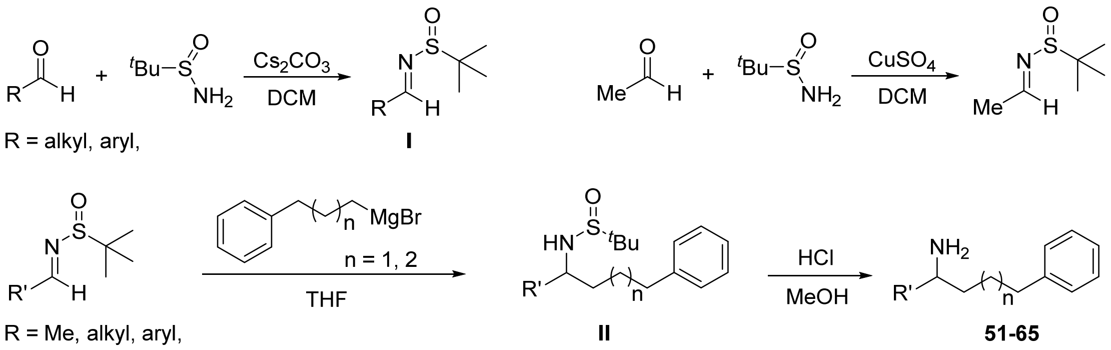

Synthesis of sulfinamide

a) General procedure for synthesis of imine (I)

To a solution of aldehyde (10 mmol, 1 equiv.) and cesium carbonate (6.52 g, 20 mmol, 2 equiv.) in dry dichloromethane (20 mL) under a nitrogen atmosphere, *tert*-butylsulfinamide (1.33 g, 11 mmol, 1.1 equiv.) was added to this solution and the reaction mixture was heated to 40 °C and stirred for 3 hours. Then, the mixture was filtered over $MgSO_4$, and the solvent was removed to give crude product of sulfinamide, which was used for next step without any further purification.

b) Synthesis of *N*-ethylidene-2-methylpropane-2-sulfinamide

To a solution of acetaldehyde (1.1 mL, 20 mmol, 1 equiv.) and anhydrous copper sulfate (6.4 g, 40 mmol, 2 equiv.) in dry dichloromethane (30 mL) under a nitrogen atmosphere, *tert*-butylsulfinamide (2.66 g, 22 mmol, 1.1 equiv.) was added to this solution and the reaction mixture was stirred at room temperature for 15 hours. Then, the mixture was filtered over $MgSO_4$, and the solvent was removed to give crude product of sulfinamide, which was used for next step without any further purification.

Preparation of Grignard reagents

To a flame-dried 50 mL three-necked round-bottom flask equipped with a dropping funnel and reflux condenser was charged activated Mg turning (18 mmol, 1.2 equiv.) and 5.0 mL anhydrous THF under a nitrogen atmosphere. The dropping funnel was charged with alkyl bromide (15 mmol, 1.0 equiv.) in 15.0 mL anhydrous THF. A small piece of I_2 crystal was added to the reaction flask to initiate the reaction, and the alkyl

bromide was added dropwise to maintain a steady reflux of solution. The addition was complete over 30 minutes. The solution was then cooled down and ready for use.

Synthesis of protected amine (II)

To a solution of the above synthesized imine **I** (10 mmol, 1 equiv.) or *N*-ethylidene-2-methylpropane-2-sulfinamide (10 mmol, 1 equiv.) in 15.0 mL anhydrous THF under a nitrogen atmosphere at -30 °C, the above synthesized Grignard reagent was added dropwise for 20 minutes. This solution was stirred at -30 °C for another 30 minutes, then the reaction was allowed to warm to room temperature and stirred overnight. The reaction was quenched with saturated NH_4Cl. The aqueous layer was extracted with ethyl acetate. The combined organic layers were washed with brine, dried over $MgSO_4$, filtered, and concentrated. The crude product **II** was obtained and used for next step without any further purification.

Synthesis of alkylarenes containing primary amines (52-66)

To a solution of the above synthesized amine **II** (10 mmol, 1 equiv.) in methanol (20 mL), hydrochloric acid (5 mL 6 M, 3 equiv.) added dropwise at room temperature overnight. After concentration, saturated solution of $NaHCO_3$ (20.0 mL) and diethyl ether (20.0 mL) were added to the reaction mixture. The organic layer was separated, and the aqueous layer was extracted with diethyl ether (60.0 mL) three times. The combined organic layers were washed with brine and dried over Na_2SO_4. After concentration, the residue was purified by flash column chromatography on silica gel (gradient elution: petroleum ether/ethyl acetate/triethyl amine = 10:1:1-4:1:0.4) to give the amine products **52-66**

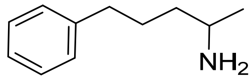

5-Phenylpentan-2-amine (52) [16]**:** was synthesized according to the general procedure (**7.12)** as yellow liquid (808 mg, 50% yield); ^{1}**H NMR** (400 MHz, $CDCl_3$) δ (ppm): 7.29-7.26 (m, 2H), 7.19-7.16 (m, 3H), 2.92-2.87 (m, 1H), 2.62 (t, *J* = 7.6 Hz, 2H), 1.71-1.58 (m, 2H), 1.41-1.32 (m, 2H), 1.05 (d, *J* = 6.4 Hz, 3H); ^{13}**C NMR** (100 MHz, $CDCl_3$) δ (ppm): 142.5, 128.4, 128.3, 125.7, 46.9, 39.9, 36.0, 28.4, 24.0.

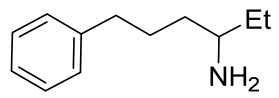

6-Phenylhexan-3-amine (53): was synthesized according to the general procedure (**7.12)** as colorless liquid (931 mg, 53% yield); **^{1}H NMR** (400 MHz, $CDCl_3$) δ (ppm): 7.29-7.26 (m, 2H), 7.19-7.16 (m, 3H), 2.67-2.60 (m, 3H), 1.79-1.66 (m, 1H), 1.65-1.57 (m, 1H), 1.51-1.40 (m, 2H), 1.35-1.23 (m, 2H), 0.90 (t, *J* = 7.2 Hz, 3H); **^{13}C NMR** (100 MHz, $CDCl_3$) δ (ppm): 142.6, 128.4, 128.3, 125.7, 52.6, 37.3, 36.1, 30.7, 28.1, 10.4.

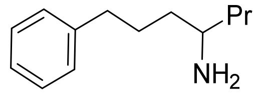

1-Phenylheptan-4-amine (54): was synthesized according to the general procedure (**7.12)** as colorless liquid (1.26 g, 66% yield); **^{1}H NMR** (400 MHz, $CDCl_3$) δ (ppm): 7.21-7.18 (m, 2H), 7.11-7.09 (m, 3H), 2.61-2.56 (m, 1H), 2.56-2.52 (m, 2H), 1.65-1.61 (m, 1H), 1.57-1.52 (m, 1H), 1.40-1.14 (m, 6H), 0.82 (t, *J* = 6.8 Hz, 3H); **^{13}C NMR** (100 MHz, $CDCl_3$) δ (ppm): 142.6, 128.5, 128.3, 125.7, 50.9, 40.4, 37.9, 36.2, 28.2, 19.3, 14.3.

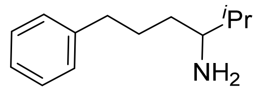

2-Methyl-6-phenylhexan-3-amine (55): was synthesized according to the general procedure (**7.12)** as colorless liquid (1.21 g, 63% yield); **^{1}H NMR** (400 MHz, $CDCl_3$) δ (ppm): 7.29-7.26 (m, 2H), 7.19-7.15 (m, 3H), 2.67-2.58 (m, 2H), 2.55-2.50 (m, 1H), 1.81-1.71 (m, 1H), 1.65-1.54 (m, 2H), 1.51-1.42 (m, 1H), 1.32-1.22 (m, 1H), 0.89 (d, *J* = 6.8 Hz, 3H), 0.85 (d, *J* = 6.8 Hz, 3H); **^{13}C NMR** (100 MHz, $CDCl_3$) δ (ppm): 142.6, 128.4, 128.3, 125.7, 56.4, 36.1, 34.6, 33.3, 28.6, 19.3, 17.0.

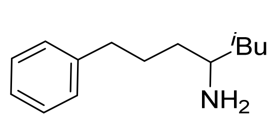

6-Methyl-1-phenylheptan-4-amine (56): was synthesized according to the general procedure (**7.12)** as colorless liquid (1.06 g, 52% yield); **^{1}H NMR** (400 MHz, $CDCl_3$) δ (ppm): 7.22-7.19 (m, 2H), 7.12-7.08 (m, 3H), 2.73-2.69 (m, 1H), 2.57-2.52 (m, 2H), 1.68-1.53 (m, 2H), 1.40-1.32 (m, 1H), 1.26-1.16 (m, 2H), 1.13-1.08 (m, 2H), 0.83 (d, *J* = 6.8 Hz, 3H), 0.80 (d, *J* = 6.8 Hz, 3H); **^{13}C NMR** (100 MHz, $CDCl_3$) δ (ppm): 142.6, 128.4, 128.3, 125.7, 48.8, 47.6, 38.3, 36.1, 28.1, 23.6, 22.0, 21.5.

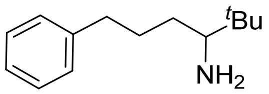

2,2-Dimethyl-6-phenylhexan-3-amine (57): was synthesized according to the general procedure (**7.12)** as colorless liquid (1.09 g, 53% yield); **^{1}H NMR** (400 MHz, $CDCl_3$) δ (ppm): 7.29-7.25 (m, 2H), 7.20-7.15 (m, 3H), 2.71-2.55 (m, 2H), 2.37-2.34 (m, 1H), 1.92-1.81 (m, 1H), 1.65-1.53 (m, 2H), 1.13-1.05 (m, 1H), 0.86 (s, 9H); **^{13}C NMR** (100 MHz, $CDCl_3$) δ (ppm): 142.7, 128.5, 128.3, 125.7, 60.6, 36.2, 34.5, 31.9, 29.8, 26.2.

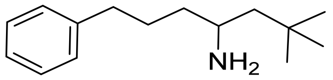

6,6-Dimethyl-1-phenylheptan-4-amine (58): was synthesized according to the general procedure (**7.12)** as colorless liquid (1.16 g, 53% yield); **^{1}H NMR** (400 MHz, $CDCl_3$) δ (ppm): 7.29-7.26 (m, 2H), 7.19-7.17 (m, 3H), 2.85-2.83 (m, 1H), 2.64-2.60 (m, 2H), 1.75-1.57 (m, 2H), 1.45-1.31 (m, 3H), 1.18-1.13 (m, 1H), 0.93 (s, 9H); **^{13}C NMR** (100 MHz, $CDCl_3$) δ (ppm): 142.6, 128.4, 128.3, 125.7, 52.3, 48.3, 40.1, 36.1, 30.2, 28.2, 21.5.

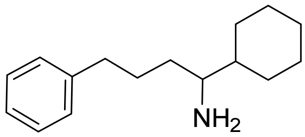

1-Cyclohexyl-4-phenylbutan-1-amine (59): was synthesized according to the general procedure (**7.12)** as colorless liquid (1.02 g, 44% yield); **^{1}H NMR** (400 MHz, $CDCl_3$) δ (ppm): 7.29-7.26 (m, 2H), 7.19-7.17 (m, 3H), 2.66-2.56 (m, 2H), 2.54-2.49 (m, 1H), 1.80-1.71 (m, 3H), 1.67-1.58 (m, 4H), 1.55-1.45 (m, 1H), 1.33-1.17 (m, 4H), 1.13-0.96 (m, 3H); **^{13}C NMR** (100 MHz, $CDCl_3$) δ (ppm): 142.6, 128.4, 128.3, 125.7, 56.0, 43.6, 36.1, 34.3, 29.8, 28.5, 27.8, 26.7, 26.6.

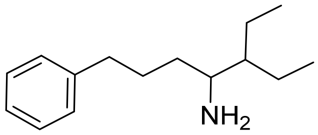

5-ethyl-1-phenylheptan-4-amine (60): was synthesized according to the general procedure (**7.12)** as colorless liquid (987 mg, 45% yield); **^{1}H NMR** (400 MHz, $CDCl_3$) δ (ppm): 7.22-7.18 (m, 2H), 7.12-7.08 (m, 3H), 2.72-2.68 (m, 1H), 2.61-2.49 (m, 2H), 1.75-1.63 (m, 1H) 1.59-1.48 (m, 1H), 1.42-1.32 (m, 1H), 1.29-1.19 (m, 4H), 1.17-1.09

(m, 1H), 1.02-0.99 (m, 1H), 0.81 (t, *J* = 7.4 Hz, 6H); **^{13}C NMR** (100 MHz, $CDCl_3$) δ (ppm): 142.6, 128.4, 128.3, 125.7, 52.2, 47.1, 36.2, 34.6, 28.9, 22.5, 21.3, 12.3, 12.2.

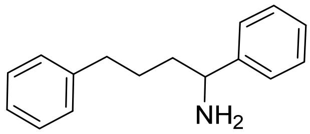

1,4-Diphenylbutan-1-amine (61)[17]**:** was synthesized according to the general procedure (**7.12)** as pale yellow liquid (1.18 g, 52% yield); **^{1}H NMR** (400 MHz, $CDCl_3$) δ (ppm): 7.33-7.21 (m, 7H), 7.17-7.12 (m, 3H), 3.88 (t, *J* = 6.6 Hz, 1H), 2.61-2.58 (m, 2H), 1.74-1.66 (m, 2H), 1.55-1.51 (m, 2H); **^{13}C NMR** (100 MHz, $CDCl_3$) δ (ppm): 146.5, 142.3, 128.5, 128.4, 128.3, 127.0, 126.3, 125.7, 56.2, 39.2, 35.8, 28.4.

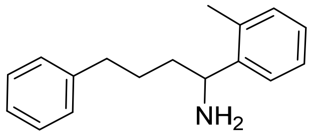

4-Phenyl-1-(*o*-tolyl)butan-1-amine (62): was synthesized according to the general procedure (**7.12)** as pale yellow liquid (1.12 g, 47% yield); **^{1}H NMR** (400 MHz, $CDCl_3$) δ (ppm): 7.37 (d, *J* = 7.2 Hz, 1H) 7.27-7.22 (m, 2H), 7.21-7.18 (m, 1H), 7.16-7.11 (m, 5H), 4.16 (t, *J* = 6.4 Hz, 1H), 2.61 (t, *J* = 7.2 Hz, 2H), 2.32 (s, 3H), 1.75-1.56 (m, 4H); **^{13}C NMR** (100 MHz, $CDCl_3$) δ (ppm): 144.6, 142.3, 134.8, 130.4, 128.4, 128.3, 126.5, 126.4, 125.8, 125.0, 51.2, 38.5, 35.9, 28.5, 19.3.

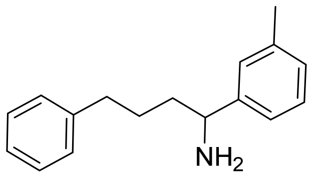

4-Phenyl-1-(*p*-tolyl)butan-1-amine (63): was synthesized according to the general procedure (**7.12)** as pale yellow liquid (1.15 g, 48% yield); **^{1}H NMR** (400 MHz, $CDCl_3$) δ (ppm): 7.27-7.16 (m, 4H), 7.15-7.04 (m, 5H), 3.85 (t, *J* = 6.4 Hz, 1H), 2.62-2.58 (m, 2H), 2.34 (s, 3H), 1.73-1.62 (m, 2H), 1.57-1.50 (m, 2H); **^{13}C NMR** (100 MHz, $CDCl_3$) δ (ppm): 146.6, 142.4, 138.1, 128.5, 128.4, 128.3, 127.7, 127.1, 125.7, 123.4, 56.2, 39.2, 35.9, 28.5, 21.5.

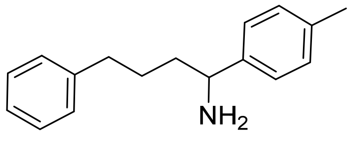

4-Phenyl-1-(*p*-tolyl)butan-1-amine (64): was synthesized according to the general procedure (**7.12**) as pale yellow liquid (1.17 g, 49% yield); **^{1}H NMR** (400 MHz, $CDCl_3$) δ (ppm): 7.26-7.22 (m, 2H), 7.18-7.15 (m, 3H), 7.13-7.11 (m, 4H), 3.84 (t, *J* = 6.4 Hz, 1H), 2.61-2.57 (m, 2H), 2.32 (s, 3H), 1.71-1.64 (m, 2H), 1.55-1.52 (m, 2H); **^{13}C NMR** (100 MHz, $CDCl_3$) δ (ppm): 143.6, 142.4, 136.5, 129.2, 128.4, 128.3, 126.3, 125.7, 56.0, 39.2, 35.9, 28.5, 21.1.

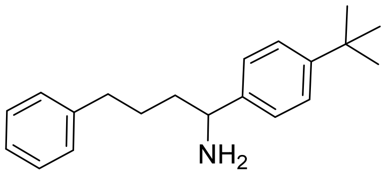

1-(4-(*tert*-Butyl)phenyl)-4-phenylbutan-1-amine (65): was synthesized according to the general procedure (**7.12**) as pale yellow liquid (1.41 g, 50% yield); **^{1}H NMR** (400 MHz, $CDCl_3$) δ (ppm): 7.34-7.32 (m, 2H), 7.27-7.22 (m, 4H), 7.17-7.13 (m, 3H), 3.86 (t, *J* = 6.4 Hz, 1H), 2.60 (t, *J* = 7.0 Hz, 2H), 1.71-1.66 (m, 2H), 1.57-1.52 (m, 2H), 1.31 (s, 9H); **^{13}C NMR** (100 MHz, $CDCl_3$) δ (ppm): 149.8, 143.6, 142.4, 128.5, 128.3, 126.0, 125.7, 125.4, 55.9, 39.2, 35.9, 34.5, 31.5, 28.5.

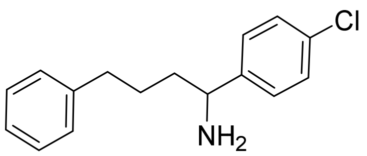

1-(4-Chlorophenyl)-4-phenylbutan-1-amine (66): was synthesized according to the general procedure (**7.12**) as pale yellow liquid (1.36 g, 53% yield); **^{1}H NMR** (400 MHz, $CDCl_3$) δ (ppm): 7.28-7.20 (m, 7H), 7.18-7.111 (m, 2H), 3.87 (t, *J* = 6.4 Hz, 1H), 2.61-2.57 (m, 2H), 1.71-1.61 (m, 2H), 1.52-1.48 (m, 2H); **^{13}C NMR** (100 MHz, $CDCl_3$) δ (ppm): 145.0, 142.2, 132.5, 128.6, 128.4 (2C), 127.8, 125.8, 55.7, 39.2, 35.8, 28.3.

7.13 Synthesis of substrate 67-71 and 73-75

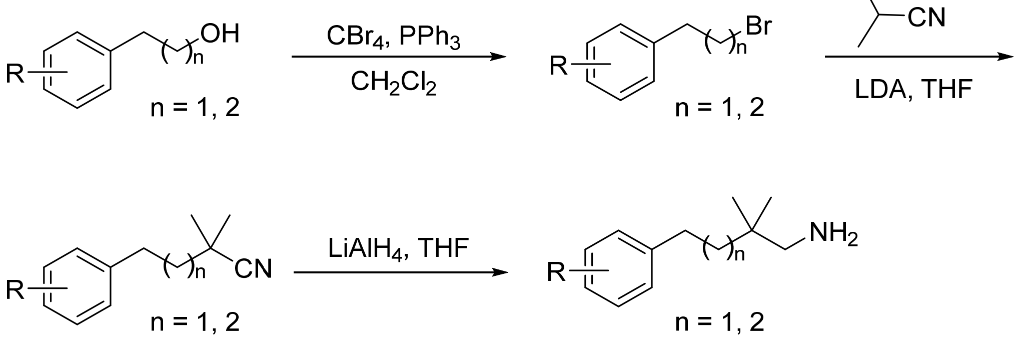

a) Synthesis of 1-aryl -2-bromoethane and 1-aryl -3-bromopropane[8a]

1-Aryl-2-bromoethane and **1-aryl -3-bromopropane** was prepared according to the following procedure: to a solution of 2-arylethan-1-ol (20 mmol) or 3-arylpropan-1-ol (20 mmol) and CBr_4 (7.20 g, 22 mmol) in DCM (30.0 mL) at 0 °C, triphenylphosphine (5.76 g, 22 mmol) was slowly added to this solution and the reaction mixture was warmed up to room temperature and stirred for 2 hours. After the reaction was complete, water (30 mL) was added to the reaction mixture. The organic layer was separated, and the aqueous layer was extracted with dichloromethane (90 mL) three times. The combined organic layers were washed with brine and dried over Na_2SO_4. After concentration, the residue was purified by flash column chromatography on silica gel (gradient elution: petroleum ether/ethyl acetate = 50:1:1-20:1) to give the product of **1-aryl -2-bromoethane** or **1-aryl -3-bromopropane.**

b) Synthesis of 2,2-dimethyl-4-arylbutanenitrile and 2,2-dimethyl-5-arylpentanenitrile[18]

2,2-Dimethyl-4-arylbutanenitrile and **2,2-dimethyl-5-arylpentanenitrile** were prepared according to the following procedure: to a solution of isobutyronitrile (690 mg, 10 mmol) in dry THF (20.0 mL) was added LDA (5.5 mL, 2 M, 11 mmol) via a syringe pump over 30 minutes at -78 °C under nitrogen atmosphere. This solution was stirred at -78 °C for another 30 minutes, and then was warmed up to room temperature gradually over 30 minutes, and then cooled to -78 ºC again. 1-Aryl -2-bromoethane (10 mmol) or 1-aryl -3-bromopropane (10 mmol) was then added and the reaction was allowed to warm to room temperature and stirred overnight. The reaction was quenched with saturated NH_4Cl. The aqueous layer was extracted with ethyl acetate. The combined organic layers were washed with brine, dried over $MgSO_4$, filtered, and concentrated. The crude product was purified by flash column chromatography on silica gel (gradient elution: petroleum ether/ethyl acetate = 20:1-10:1) to give **2,2-dimethyl-4-arylbutanenitrile** or **2,2-dimethyl-5-arylpentanenitrile.**

c) Synthesis of 2,2-dimethyl-4-arylbutan-1-amine and 2,2-dimethyl-5-arylpentan-1-amine[18]

2,2-Dimethyl-4-arylbutan-1-amine and 2,2-dimethyl-5-arylpentan-1-amine were prepared according to the following procedure: the above synthesized 2,2-dimethyl-4-arylbutanenitrile or 2,2-dimethyl-5-arylpentanenitrile was dissolved in dry THF (15 ml) and placed in an ice bath under nitrogen atmosphere. $LiAlH_4$ (15 mmol, 570 mg) was

added to this solution carefully. Then, the reaction mixture was warmed up to room temperature and heated to reflux for 4 hours. The reaction was quenched with water (1.0 mL), and the solution was stirred for an additional hour at room temperature. The mixture was filtered over $MgSO_4$, and the solvent was removed to give the crude product, which was purified by flash column chromatography on silica gel (gradient elution: petroleum ether/ethyl acetate/triethyl amine = 10:1:1-4:1:0.4) to give **66-70 and 72-74.**

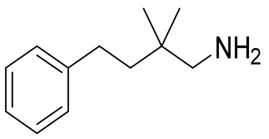

2,2-Dimethyl-5-phenylpentan-1-amine (67) was synthesized according to the above procedure as colorless liquid (816 mg, 46% yield); **^{1}H NMR** (400 MHz, $CDCl_3$) δ (ppm): 7.26-7.23 (m, 2 H), 7.17-7.12 (m, 3H), 2.55-2.53 (m, 2H), 2.50 (s, 2H), 1.53-1.47 (m, 2H), 0.90 (s, 6H); **^{13}C NMR** (100 MHz, $CDCl_3$) δ (ppm): 143.1, 128.2, 128.1, 125.4, 52.7, 41.6, 34.5, 30.4, 24.4.

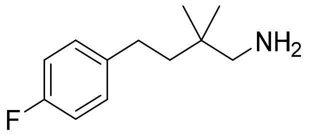

4-(4-Fluorophenyl)-2,2-dimethylbutan-1-amine (68) was synthesized according to the above procedure as colorless liquid (918 mg, 47% yield); **^{1}H NMR** (400 MHz, $CDCl_3$) δ (ppm): 7.15-7.12 (m, 2H), 6.96-6.92 (m, 2H), 2.56 (s, 2H), 2.54-2.50 (m, 2H), 1.53-1.49 (m, 2H), 0.96 (s, 6H); **^{13}C NMR** (100 MHz, $CDCl_3$) δ (ppm): 161.2 (d, J_{C-F} = 241.6 Hz), 138.5 (d, J_{C-F} = 3.2 Hz), 129.7 (d, J_{C-F} = 7.8 Hz), 115.3 (d, J_{C-F} = 20.9 Hz), 52.0, 41.8, 34.3, 29.7, 24.6.

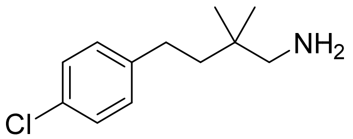

4-(4-Chlorophenyl)-2,2-dimethylbutan-1-amine (69) was synthesized according to the above procedure as light yellow oil (1.10 g, 52% yield); **^{1}H NMR** (400 MHz, $CDCl_3$) δ (ppm): 7.25-7.20 (m, 2H), 7.19-7.08 (m, 2H), 2.62-2.49 (m, 2H), 2.48 (s, 2H), 1.48-1.44 (m, 2H), 0.90 (s, 6H); **^{13}C NMR** (100 MHz, $CDCl_3$) δ (ppm): 141.5, 131.2, 129.6, 128.3, 52.5, 41.6, 34.8, 29.9, 24.6.

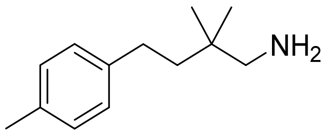

2,2-Dimethyl-4-*p*-tolylbutan-1-amine (70) was synthesized according to the above procedure as colorless liquid (1.11 g, 58% yield); **^{1}H NMR** (400 MHz, $CDCl_3$) δ (ppm): 7.12 (s, 4H), 2.53-2.50 (m, 2H), 2.49 (s, 2H), 2.31 (s, 3H), 1.50-1.46 (m, 2H), 0.91 (s, 6H); **^{13}C NMR** (100 MHz, $CDCl_3$) δ (ppm): 140.2, 135.1, 129.1, 128.2, 52.9, 42.0, 34.8, 30.1, 24.6, 21.0.

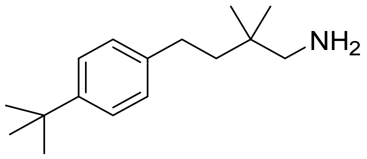

4-(4-*tert*-Butylphenyl)-2,2-dimethylbutan-1-amine (71) was synthesized according to the above procedure as colorless liquid (1.07 g, 46% yield); **^{1}H NMR** (400 MHz, $CDCl_3$) δ (ppm): 7.32 (d, *J* = 8.2 Hz, 2H), 7.14 (d, *J* = 8.2 Hz, 2H), 2.57-2.54 (m, 2H), 2.53 (s, 2H), 1.56-1.51 (m, 2H), 1.33 (s, 9H), 0.92 (s, 6H); **^{13}C NMR** (100 MHz, $CDCl_3$) δ (ppm): 148.5, 140.1, 127.9, 125.6, 52.8, 41.7, 34.7, 34.4, 31.5, 29.9, 24.6.

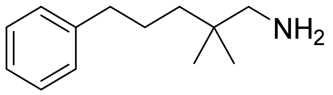

2,2-Dimethyl-5-phenylpentan-1-amine (73) was synthesized according to the above procedure as colorless liquid (1.01 g, 53% yield); **^{1}H NMR** (400 MHz, $CDCl_3$) δ (ppm): 7.29-7.24 (m, 2 H), 7.24-7.22 (m, 3 H), 2.58 (t, *J* = 7.6 Hz, 2H), 2.4 (s, 2H), 1.60-1.52 (m, 2 H), 1.26-1.22 (m, 2 H), 0.82 (s, 6H); **^{13}C NMR** (100 MHz, $CDCl_3$) δ (ppm): 142.7, 128.4, 128.3, 125.7, 52.5, 39.1, 36.8, 34.1, 26.0, 24.7.

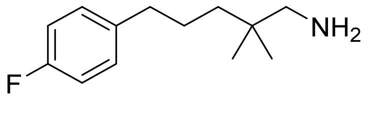

5-(4-Fluorophenyl)-2,2-dimethylpentan-1-amine (74) was synthesized according to the above procedure as colorless liquid (1.17 g, 56% yield); **^{1}H NMR** (400 MHz, $CDCl_3$) δ (ppm): 7.27-7.18 (m, 2H), 7.05-7.01 (m, 2H), 2.63 (t, *J* = 7.6 Hz, 2H), 2.50 (s, 2H), 1.60-1.59 (m, 2H), 1.34-1.28 (s, 2H), 0.90 (s, 6H); **^{13}C NMR** (100 MHz, $CDCl_3$) δ (ppm): 161.2 (d, J_{C-F} = 241.8 Hz), 138.2 (d, J_{C-F} = 3.0 Hz), 129.6 (d, J_{C-F} = 7.7 Hz), 115.1 (d, J_{C-F} = 21.0 Hz), 52.7, 39.0, 35.9, 34.4, 26.0, 24.5.

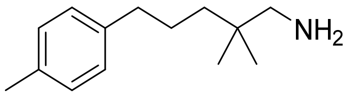

2,2-Dimethyl-5-p-tolylpentan-1-amine (75) was synthesized according to the above procedure as colorless liquid (985 mg, 48% yield); ^{1}**H NMR** (400 MHz, $CDCl_3$) δ (ppm): 7.19 (s, 4H), 2.63 (t, *J* = 7.6 Hz, 2H), 2.51 (s, 2H), 2.40 (s, 3H), 1.68-1.60 (m, 2H), 1.37-1.30 (m, 2H), 0.91 (s, 6H); ^{13}**C NMR** (100 MHz, $CDCl_3$) δ (ppm): 139.5, 135.0, 128.9, 128.2, 52.7, 39.0, 36.3, 34.3, 26.1, 24.6, 20.9.

7.14 Synthesis of substrate 76-8^{3}[19]

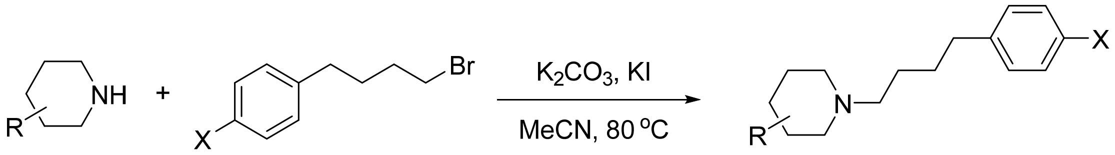

A 50 mL round bottom flask was charged with a stir bar, substituted piperidine (5 mmol), K_2CO_3 (897 mg, 6.5 mmol), KI (83 mg, 0.5 mmol), 1-aryl -4-bromobutane (5 mmol), and MeCN (20.0 mL). The reaction mixture was stirred rapidly and heated at 80 °C overnight. The reaction mixture was allowed to cool to room temperature, Then, the reaction mixture was filtered through Celite to remove K_2CO_3 and washed with ethyl acetate. The solvent was removed to give the crude product, which was purified by flash column chromatography on silica gel (gradient elution: petroleum ether/ethyl acetate = 10:1-4:1) to give the compound **76-8**3.

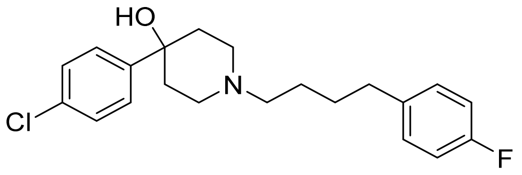

4-(4-Chlorophenyl)-1-(4-(4-fluorophenyl)butyl)piperidin-4-ol (76) was synthesized according to the above procedure as white solid (995 mg, 55% yield); ^{1}**H NMR** (400 MHz, $CDCl_3$) δ (ppm): 7.43 (d, *J* = 8.4 Hz, 2H), 7.30 (d, *J* = 8.4 Hz, 2H), 7.13 (dd, *J* = 8.4, 6.0 Hz, 2H), 6.97 (t, *J* = 8.4 Hz, 2H), 2.78 (dd, *J* = 9.2, 5.6 Hz, 2H), 2.60 (t, *J* = 6.8 Hz, 2H), 2.41-2.35 (m, 4H), 2.12 (td, *J* = 12.8, 4.0 Hz, 2H), 1.71-1.54 (m, 6H); ^{13}**C NMR** (100 MHz, $CDCl_3$) δ (ppm): 161.2 (d, J_{C-F} = 241.6 Hz), 147.1, 138.1 (d, J_{C-F} = 3.3 Hz), 132.8, 129.7 (d, J_{C-F} = 7.8 Hz), 128.5, 126.2, 115.1 (d, J_{C-F} = 20.8 Hz), 71.2, 58.7, 50.0, 38.6, 35.1, 29.7, 26.7.

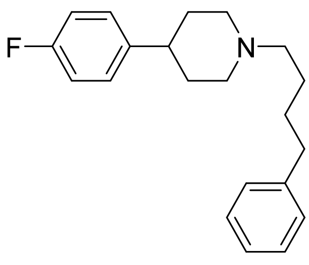

4-(4-Fluorophenyl)-1-(4-phenylbutyl)piperidine (77) was synthesized according to the above procedure as white solid (825 mg, 53% yield); **^{1}H NMR** (400 MHz, $CDCl_3$) δ (ppm): 7.29-7.26 (m, 2H), 7.20-7.15 (m, 5H), 6.99-6.94 (m, 2H), 3.03 (d, *J* = 11.6 Hz, 2H), 2.67-2.63 (m, 2H), 2.50-2.41 (m, 1H), 2.39-2.34 (m, 2H), 2.00 (td, *J* = 11.2, 3.2 Hz, 2H), 1.81-1.54 (m, 8H); **^{13}C NMR** (100 MHz, $CDCl_3$) δ (ppm): 161.1 (d, $J_{C\text{-}F}$ = 242.2 Hz), 142.3, 141.9 (d, $J_{C\text{-}F}$ = 3.1 Hz), 128.2, 128.1, 128.0 (d, $J_{C\text{-}F}$ = 7.6 Hz), 125.6, 115.1 (d, $J_{C\text{-}F}$ = 21.0 Hz), 58.8, 54.2, 42.0, 35.7, 33.6, 29.4, 26.6.

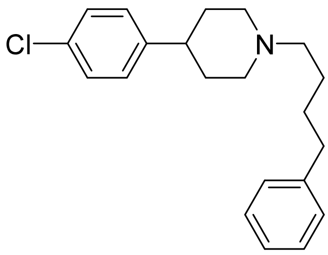

4-(4-Chlorophenyl)-1-(4-phenylbutyl)piperidine (78) was synthesized according to the above procedure as white solid (852 mg, 52% yield); **^{1}H NMR** (400 MHz, $CDCl_3$) δ (ppm): 7.30-7.24(m, 4H), 7.19-7.13(m, 5H), 3.04-3.01 (m, 2H), 2.66-2.62 (m, 2H), 2.49-2.40 (m, 1H), 2.39-2.35 (m, 2H), 2.03-1.97 (m, 2H), 1.81-1.53 (m, 8H); **^{13}C NMR** (100 MHz, $CDCl_3$) δ (ppm): 144.9, 142.5, 131.6, 128.4, 128.3, 128.2, 128.1 125.6, 59.0, 54.3, 42.3, 35.9, 33.5, 29.5, 26.8.

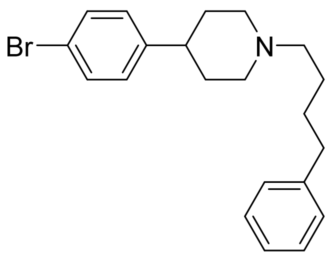

4-(4-Bromophenyl)-1-(4-phenylbutyl)piperidine (79) was synthesized according to the above procedure as white solid (931 mg, 50% yield); **^{1}H NMR** (400 MHz, $CDCl_3$) δ (ppm): 7.40-7.37 (m, 2H),7.28-7.24 (m, 2H), 7.18-7.17 (m, 3H), 7.09-7.06 (m, 2H), 3.03-3.00 (m, 2H), 2.65-2.61 (m, 2H), 2.46-2.41 (m, 1H), 2.39-2.35 (m, 2H), 2.02-1.96

(m, 2H), 1.77-1.56 (m, 8H); **^{13}C NMR** (100 MHz, $CDCl_3$) δ (ppm): 145.4, 142.4, 131.4, 128.6, 128.3, 128.2, 125.6, 119.7, 58.9, 54.3, 42.2, 35.8, 33.4, 29.5, 26.7.

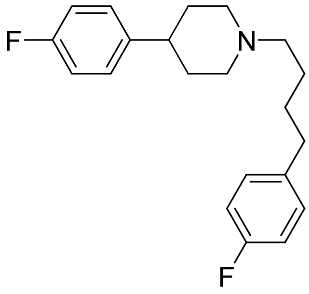

4-(4-Fluorophenyl)-1-(4-(4-fluorophenyl)butyl)piperidine (80) was synthesized according to the above procedure as white solid (675 mg, 41% yield); **^{1}H NMR** (400 MHz, $CDCl_3$) δ (ppm): 7.40-7.37 (m, 2H), 7.29-7.24 (m, 2H), 7.18-7.16 (m, 2H), 7.09-7.06 (m, 2H), 3.03-3.00 (m, 2H), 2.65-2.61 (m, 2H), 2.49-2.44 (m, 1H), 2.42-2.35 (m, 2H), 2.02 (td, J = 12.0, 4.0 Hz, 2H), 1.77-1.56 (m, 8H); **^{13}C NMR** (100 MHz, $CDCl_3$) δ (ppm): 161.4 (d, J_{C-F} = 242.1 Hz), 161.3 (d, J_{C-F} = 241.8 Hz), 142.2 (d, J_{C-F} = 3.0 Hz), 138.1 (d, J_{C-F} = 3.0 Hz), 129.7 (d, J_{C-F} = 7.6 Hz), 128.2 (d, J_{C-F} = 7.5 Hz), 115.1 (d, J_{C-F} = 20.7 Hz), 115.0 (d, J_{C-F} = 20.9 Hz), 59.0, 54.5, 42.1, 35.1, 33.8, 29.7, 26.7.

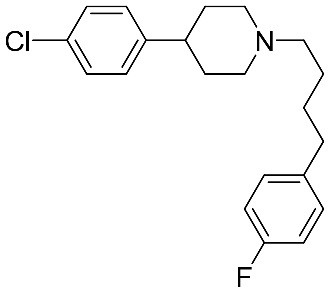

4-(4-Chlorophenyl)-1-(4-(4-fluorophenyl)butyl)piperidine (81) was synthesized according to the above procedure as white solid (951 mg, 55% yield); **^{1}H NMR** (400 MHz, $CDCl_3$) δ (ppm): 7.27-7.24 (m, 2H), 7.17-7.11 (m, 4H), 6.99-6.92 (m, 2H), 3.02 (d, J = 9.6 Hz, 2H), 2.61 (t, J = 7.0 Hz, 2H), 2.53-2.42 (m, 1H), 2.41-2.34 (m, 2H), 2.00 (td, J = 11.6, 3.2 Hz, 2H), 1.85-1.50 (m, 8H); **^{13}C NMR** (100 MHz, $CDCl_3$) δ (ppm): 161.4 (d, J_{C-F} = 242.9 Hz), 145.1, 138.3 (d, J_{C-F} = 3.2 Hz), 131.8, 129.8 (d, J_{C-F} = 7.6 Hz), 128.7, 128.3, 115.1 (d, J_{C-F} = 21.1 Hz), 59.1, 54.4, 42.3, 35.3, 33.6, 29.9, 26.8.

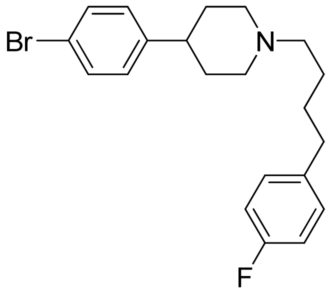

4-(4-Bromophenyl)-1-(4-(4-fluorophenyl)butyl)piperidine (82) was synthesized according to the above procedure as white solid (742 mg, 38% yield); **^{1}H NMR** (400 MHz, $CDCl_3$) δ (ppm): 7.40-7.38 (m, 2H), 7.13-7.07 (m, 4H), 6.97-6.92 (m, 2H), 3.01 (d, J = 11.6 Hz, 2H), 2.60 (t, J = 7.6 Hz, 2H), 2.48-2.40 (m, 1H), 2.36 (t, J = 7.2 Hz, 2H), 1.99 (td, J = 11.4, 2.8 Hz, 2H), 1.80-1.71 (m, 4H), 1.65-1.52 (m, 4H); **^{13}C NMR** (100 MHz, $CDCl_3$) δ (ppm): 161.4 (d, $J_{C\text{-}F}$ = 241.5 Hz), 145.5, 138.1 (d, $J_{C\text{-}F}$ = 3.2 Hz), 131.5, 129.7 (d, $J_{C\text{-}F}$ = 7.7 Hz), 128.7, 119.7, 115.1 (d, $J_{C\text{-}F}$ = 21.0 Hz), 59.0, 54.4, 42.3, 35.1, 34.5, 29.7, 26.7.

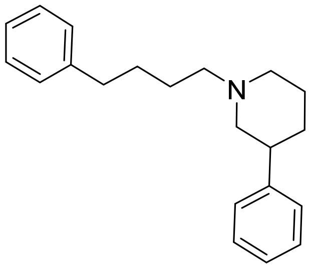

3-Phenyl-1-(4-phenylbutyl)piperidine (83) was synthesized according to the above procedure as white solid (734 mg, 50% yield); **^{1}H NMR** (400 MHz, $CDCl_3$) δ (ppm): 7.28-7.12 (m, 10H), 2.99-2.91 (m, 2H), 2.83-2.76 (m, 1H), 2.61-2.57 (m, 2H), 2.36-2.34 (m, 2H), 1.96-1.86 (m, 3H), 1.76-1.67 (m, 2H), 1.63-1.51 (m, 4H), 1.47-1.36 (m, 1H); **^{13}C NMR** (100 MHz, $CDCl_3$) δ (ppm): 144.8, 142.4, 128.3, 128.3, 128.2, 127.2, 126.2, 125.6, 61.4, 59.0, 54.0, 43.0, 35.8, 31.7, 29.5, 26.6, 25.8.

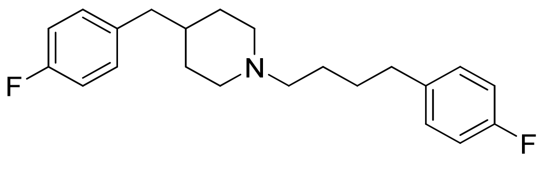

4-(4-Fluorobenzyl)-1-(4-(4-fluorophenyl)butyl)piperidine (84) [19] was synthesized according to the above procedure as white solid (859 mg, 50% yield); **^{1}H NMR** (400 MHz, $CDCl_3$) δ (ppm): 7.17-7.06 (m, 4H), 7.02-6.91 (m, 4H), 2.88 (d J = 11.6 Hz, 2H), 2.65-2.55 (m, 2H), 2.51 (d, J = 7.2 Hz, 2H), 2.33-2.28 (m, 2H), 1.89-1.78 (m, 2H),

1.65-1.56 (m, 4H), 1.56-1.42 (m, 3H), 1.36-1.23 (m, 2H); **^{13}C NMR** (100 MHz, $CDCl_3$) δ (ppm): 161.4 (d, J_{C-F} = 243.2 Hz), 161.2 (d, J_{C-F} = 243.0 Hz), 138.2 (d, J_{C-F} = 3.1 Hz), 136.5 (d, J_{C-F} = 3.2 Hz), 130.6 (d, J_{C-F} = 7.7 Hz), 129.8 (d, J_{C-F} = 7.7 Hz), 115.1 (d, J_{C-F} = 21.1 Hz), 115.0 (d, J_{C-F} = 21.0 Hz), 59.2, 54.2, 42.5, 38.2, 35.3, 32.3, 29.8, 26.8.

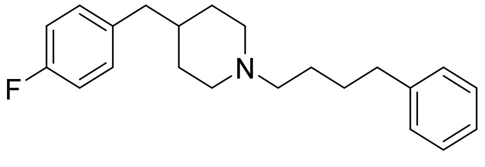

4-(4-Fluorobenzyl)-1-(4-phenylbutyl)piperidine (85) was synthesized according to the above procedure as white solid (684 mg, 42% yield); **^{1}H NMR** (400 MHz, $CDCl_3$) δ (ppm): 7.20-7.17 (m, 3H), 7.10-7.08 (m, 2H), 7.02-6.98 (m, 2H), 6.89-6.85 (m, 2H), 2.80 (d, J = 11.6 Hz, 2H), 2.54 (d, J = 7.2 Hz, 2H), 2.41 (d, J = 7.0 Hz, 2H), 2.24-2.20 (m, 2H), 1.75 (td, J = 11.8, 2.2 Hz, 2H), 1.57-1.36 (m, 7H), 1.25-1.15 (m, 2H); **^{13}C NMR** (100 MHz, $CDCl_3$) δ (ppm): 161.4 (d, J_{C-F} = 241.9 Hz), 142.6, 136.4 (d, J_{C-F} = 3.3 Hz), 130.5 (d, J_{C-F} = 7.8 Hz), 128.5, 128.3, 125.7, 114.9 (d, J_{C-F} = 20.8 Hz), 59.1, 54.1, 42.5, 38.2, 36.0, 32.2, 29.6, 26.9.

7.15 Synthesis of substrate 88, 89[19]

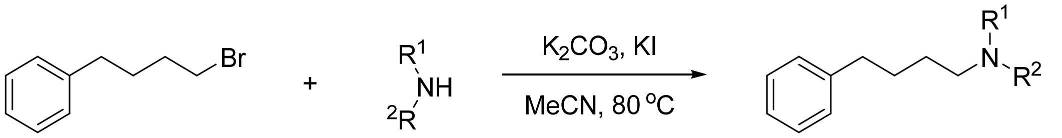

A 50 mL round bottom flask was charged with a stir bar, secondary amine (5 mmol), K_2CO_3 (897 mg, 6.5 mmol), KI (83 mg, 0.5 mmol), 1-aryl -4-bromobutane (5 mmol), and MeCN (20.0 mL). The reaction mixture was stirred rapidly and heated at 80 °C overnight. The reaction mixture was allowed to cool to room temperature, Then, the reaction mixture was filtered through Celite to remove K_2CO_3 and washed with ethyl acetate. The solvent was removed to give the crude product, which was purified by flash column chromatography on silica gel (gradient elution: petroleum ether/ethyl acetate = 10:1-4:1) to give the compound **88, 89**.

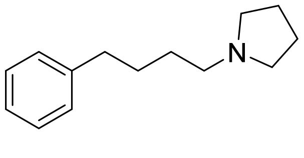

1-(4-Phenylbutyl)pyrrolidine (88) was synthesized according to the above procedure as white solid (629 mg, 62% yield); **^{1}H NMR** (400 MHz, $CDCl_3$) δ (ppm): 7.28-7.24

(m, 2H), 7.18-7.14 (m, 3H), 2.63 (t, *J* = 7.2 Hz, 2H), 2.49-2.41 (m, 6H), 1.78-1.74 (m, 4H), 1.68-1.61 (m, 2H), 1.59-1.54 (m, 2H); **^{13}C NMR** (100 MHz, $CDCl_3$) δ (ppm): 142.5, 128.4, 128.2, 125.6, 56.5, 54.2, 35.8, 29.5, 28.8, 23.4.

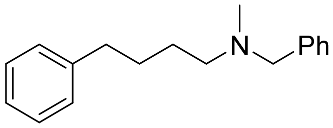

***N*-Benzyl-*N*-methyl-4-phenylbutan-1-amine (89)** was synthesized according to the above procedure as white solid (645 mg, 51% yield); **^{1}H NMR** (400 MHz, $CDCl_3$) δ (ppm): 7.30-7.27 (m, 4H), 7.26-7.20 (m, 3H), 7.17-7.14 (m, 3H), 3.45 (s, 2H), 2.60 (t, *J* = 7.2 Hz, 2H), 2.37 (t, *J* = 7.2 Hz, 2H), 3.16 (s, 3H), 1.68-1.60 (m, 2H), 1.58-1.51 (m, 2H); **^{13}C NMR** (100 MHz, $CDCl_3$) δ (ppm): 142.6, 139.3, 129.0, 128.4, 128.2, 128.1, 126.8, 125.6, 62.4, 57.2, 42.2, 35.8, 29.2, 27.0.

8. General procedure for the catalytic benzylic oxidation with H_2O_2

Procedure A: oxidation of molecules with functional groups other than amines

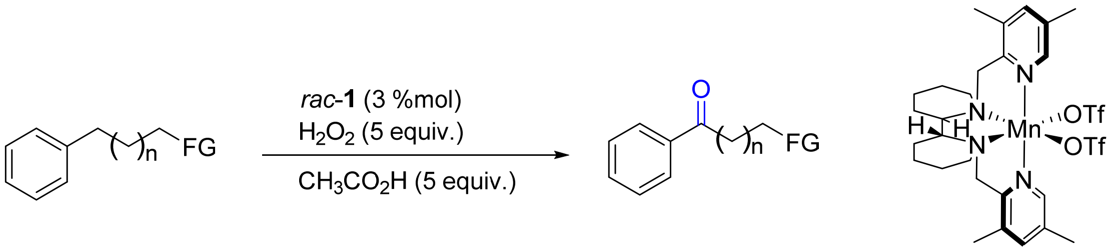

rac-**1**

To a solution of substrate (0.5 mmol, 1 equiv.), *rac*-**1** (3 mol%) and AcOH (2.5 mmol, 5 equiv.) in MeCN (1.5 mL), H_2O_2 (2.5 mmol, 284 µL, 30 wt.% aqueous solution) in 1.5 mL MeCN was delivered by a syringe pump over 1 hour at room temperature without nitrogen protection. The resulting mixture was quenched with saturated Na_2SO_3 aqueous solution and extracted with DCM (3×15.0 mL). The organic layer was dried over Na_2SO_4. The crude product was purified by silica gel column chromatography to afford the product. When the FG is a carboxylic acid, following quenching with soild Na_2SO_3 (252 mg, 2 mmol), the mixture was filtered and the solvent evaporated in vacuo. The crude product was purified by silica column chromatography.

Procedure B: oxidation of molecules with primary amino functional groups

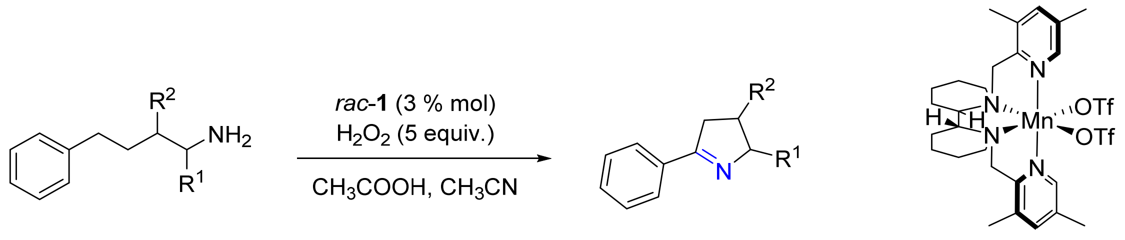

Primary amine (0.5 mmol) was dissoloved in AcOH (1.5 mL) and stirred for 10 minutes at room temperature, followed by addition of *rac*-**1** (3 mol%) to the mixture. H_2O_2 (2.5 mmol, 284 µL, 30 wt.% aqueous solution dissolved in 1.5 mL of MeCN) was then delivered by a syringe pump over 1 hour at room temperature without nitrogen protection. The reaction mixture was quenched with solid Na_2SO_3 (252 mg, 2 mmol), filtered and following addition of MeCN (20 mL), most of the acetic acid was removed in vacuo. The resulting mixture was adjusted to pH = 7 with NaOH (10 wt.%) and extracted with DCM (3×15 mL). The organic layer was dried over Na_2SO_4, filtered and evaporated in vacuo. The residue was purified by silica gel column chromatography to afford the product.

Procedure C: oxidation of molecules with tertiary amino functional groups

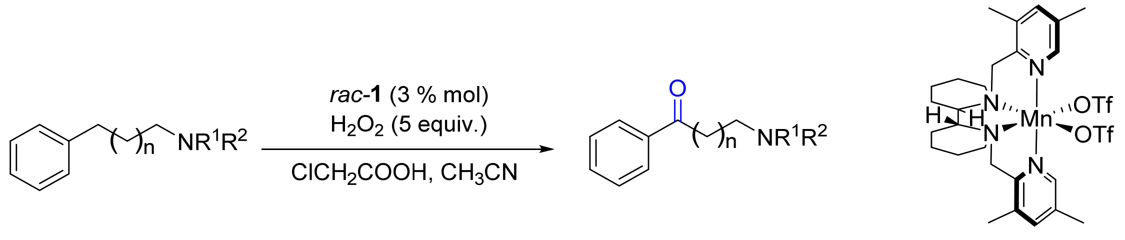

Tertiary amine (0.25 mmol) and $ClCH_2COOH$ (239 mg, 2.5 mmol, 10 equiv.) were dissoloved in MeCN (1.0 mL) and stirred for 10 min at room temperature, followed by addition of *rac*-**1** (2 mol%). H_2O_2 (1.25 mmol, 142 µL, 30 wt% in H_2O dissolved in 1.0 mL of MeCN) was then added by syringe pump over 1 hour at room temperature. After completion of the oxidation, the resulting mixture was adjusted to pH = 7 by NaOH (10 wt%) and quenched with saturated Na_2SO_3 aqueous solution, and the mixture was then extracted with DCM (3×15 mL). The organic layer was dried over Na_2SO_4, filtered and evaporated in vacuo. The residue was purified by silica gel column chromatography to afford the product.

9. Oxidation of amine 85 at gram-scale

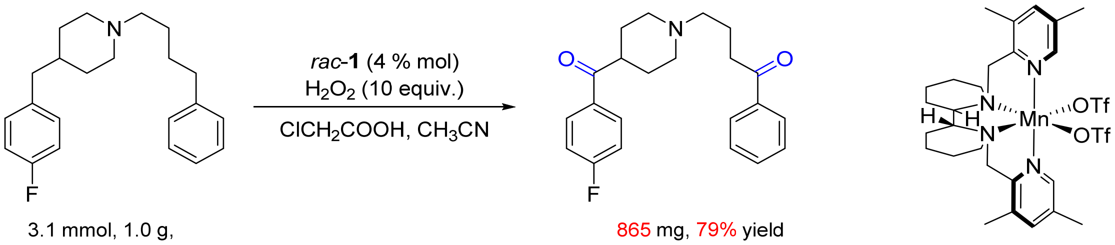

To a solution of 4-(4-fluorobenzyl)-1-(4-phenylbutyl)piperidine (**85,** 1.0 g, 3.1 mmol) and $ClCH_2COOH$ (2.91 g, 31 mmol, 10 equiv.) in MeCN (25.0 mL) stirred for 10 minutes at room temperature, the catalyst *rac*-**1** (47 mg, 2 mol%) was added. H_2O_2 (31 mmol, 3.51 mL, 30 wt% in H_2O dissolved in 5 mL of MeCN) was then introduced by a syringe pump over 1 hour at room temperature. Halfway in the addition of H_2O_2, another portion of the catalyst *rac*-**1** (47 mg, 2 mol%) was added to the stirred reaction. After completion of the oxidation, the resulting mixture was quenched with saturated Na_2SO_3 aqueous solution, and basified to pH = 8 with a NaOH (20 wt%) aqueous solution, and the resulting mixture was extracted with DCM (3×50.0 mL). The combined organic layers were then dried over Na_2SO_4, and filtered. After concentration, the residue was purified by flash column chromatography on silica gel (gradient elution: petroleum ether/ethyl acetate = 10:1-4:1) to give the desired product **85a** (865 mg, 79% yield).

10. Analytic data of products

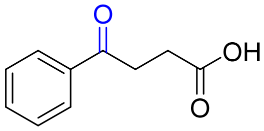

4-Oxo-4-phenylbutanoic acid (2a)[20] was synthesized by the general Procedure **A** and purified by flash column chromatography on silica gel (gradient elution: Hexanes/EtOAc = 10:1-4:1) to give the desired product as white solid (84 mg, 94% yield); **^{1}H NMR** (400 MHz, $CDCl_3$) δ (ppm): 7.99 (d, *J* = 7.2 Hz, 2H, ArH), 7.60-7.56 (m, 1H, ArH), 7.49-7.45 (m, 2H, ArH), 3.32 (t, *J* = 6.6 Hz, 2H), 2.83 (t, *J* = 6.4 Hz, 2H); **^{13}C NMR** (100 MHz, $CDCl_3$) δ (ppm): 197.8 (ArC=O), 179.0, 136.3, 133.3, 128.6, 128.0, 33.1, 28.0; **HRMS** (ESI) m/z calcd for $C_{10}H_{10}O_3Na$ $[M+Na]^+$: 201.0522, found: 201.0521; **IR** (KBr, plate), ν (cm^{-1}): 1738, 1688 (C=O).

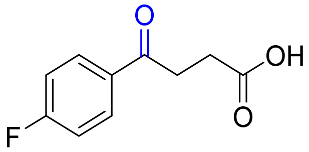

4-(4-Fluorophenyl)-4-oxobutanoic acid (3a)[21] was synthesized by the general Procedure **A** and purified by flash column chromatography on silica gel (gradient elution: Hexanes/EtOAc = 10:1-4:1) to give the desired product as white solid (94 mg, 96% yield); **^{1}H NMR** (400 MHz, $CDCl_3$) δ (ppm): 8.03-8.00 (m, 2H, ArH), 7.14 (t, *J* = 8.6 Hz, 2H, ArH), 3.29 (t, *J* = 6.4 Hz, 2H), 2.82 (t, *J* = 6.4 Hz, 2H); **^{13}C NMR** (100 MHz, $CDCl_3$) δ (ppm): 196.2 (ArC=O), 178.0, 166.0 (d, $J_{C\text{-}F}$ = 254.0 Hz), 132.8, 130.7 (d, $J_{C\text{-}F}$ = 9.0 Hz), 115.7 (d, $J_{C\text{-}F}$ = 21.0Hz), 33.2, 28.0; **HRMS** (ESI) m/z calcd for $C_{10}H_9FO_3Na$ $[M+Na]^+$: 219.0428, found: 219.0430; **IR** (KBr, plate), ν (cm^{-1}): 1695 (C=O).

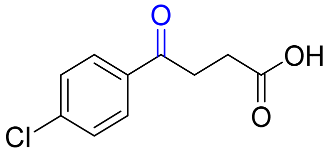

4-(4-Chlorophenyl)-4-oxobutanoic acid (4a)[22] was synthesized by the general Procedure **A** and purified by flash column chromatography on silica gel (gradient elution: Hexanes/EtOAc = 10:1-4:1) to give the desired product as white solid (101 mg, 95% yield); **^{1}H NMR** (400 MHz, $CDCl_3$) δ (ppm): 7.92 (d, *J* = 8.4 Hz, 2H, ArH), 7.45 (d, *J* = 8.4 Hz, 2H, ArH), 3.28 (t, *J* = 6.6 Hz, 2H), 2.82 (t, *J* = 6.4 Hz, 2H); **^{13}C NMR** (100 MHz, $CDCl_3$) δ (ppm): 196.6 (ArC=O), 178.2, 139.8, 134.7, 129.5, 129.0, 33.1, 27.9; **HRMS** (ESI) m/z calcd for $C_{10}H_9ClO_3Na$ $[M+Na]^+$: 235.0132, found: 235.0133; **IR** (KBr, plate), ν (cm^{-1}): 1686 (C=O).

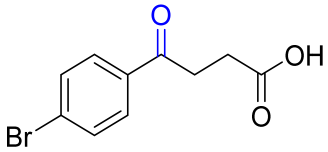

4-(4-Bromophenyl)-4-oxobutanoic acid (5a)[23] was synthesized by the general Procedure **A** and purified by flash column chromatography on silica gel (gradient elution: Hexanes/EtOAc = 10:1-4:1) to give the desired product as white solid (122 mg, 95% yield); **^{1}H NMR** (400 MHz, $CDCl_3$) δ (ppm): 7.84 (d, *J* = 8.6 Hz, 2H, ArH), 7.61 (d, *J* = 8.4 Hz, 2H, ArH), 3.27 (t, *J* = 6.4 Hz, 2H), 2.81 (t, *J* = 6.4 Hz, 2H); **^{13}C NMR** (100 MHz, $CDCl_3$) δ (ppm): 196.8 (ArC=O), 178.6, 135.1, 132.0, 129.5, 128.5,

33.1, 27.9; **HRMS** (ESI) m/z calcd for $C_{10}H_9BrO_3Na$ $[M+Na]^+$: 278.9627, found: 278.9626; **IR** (KBr, plate), ν (cm^{-1}): 1746, 1674 (C=O).

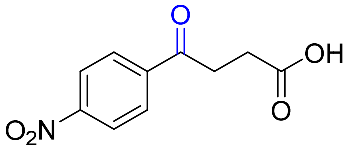

4-(4-Nitrophenyl)-4-oxobutanoic acid (6a) was synthesized by the general Procedure **A** and purified by flash column chromatography on silica gel (gradient elution: Hexanes/EtOAc = 10:1-4:1) to give the desired product as yellow solid (99 mg, 89% yield); **^{1}H NMR** (400 MHz, DMSO-d6) δ (ppm): 12.22 (brs, 1H, COOH), 8.34 (d, *J* = 8.8 Hz, 2H, ArH), 8.20 (d, *J* = 8.8 Hz, 2H, ArH), 3.33 (t, *J* = 6.2 Hz), 2.62 (t, *J* = 6.2 Hz, 2H); **^{13}C NMR** (100 MHz, DMSO-d6) δ (ppm): 198.4 (ArC=O), 174.2, 150.5, 141.5, 129.8, 124.4, 34.3, 28.3; **HRMS** (ESI) m/z calcd for $C_{10}H_9NO_5Na$ $[M+Na]^+$: 246.0373, found: 246.0346; **IR** (KBr, plate), ν (cm^{-1}): 1744, 1678 (C=O).

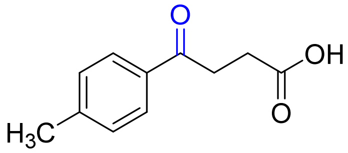

4-Oxo-4-(*p*-tolyl)butanoic acid (7a)[24] was synthesized by the general Procedure **A** and purified by flash column chromatography on silica gel (gradient elution: Hexanes/EtOAc = 10:1-4:1) to give the desired product as white solid (89 mg, 93% yield); **^{1}H NMR** (400 MHz, $CDCl_3$) δ (ppm): 7.88 (d, *J* = 8.0 Hz, 2H, ArH), 7.26 (d, *J* = 7.6 Hz, 2H, ArH), 3.30 (t, *J* = 6.6 Hz, 2H), 2.81 (t, *J* = 6.4 Hz, 2H), 2.42 (s, 3H); **^{13}C NMR** (100 MHz, $CDCl_3$) δ (ppm): 197.5(ArC=O), 180.7, 144.2, 133.9, 129.3, 128.2, 33.1, 28.0, 21.7; **HRMS** (ESI) m/z calcd for $C_{11}H_{12}O_3Na$ $[M+Na]^+$: 215.0679, found: 215.0680; **IR** (KBr, plate), ν (cm^{-1}): 1674 (C=O).

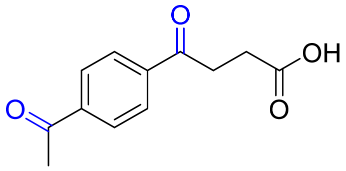

4-(4-Acetylphenyl)-4-oxobutanoic acid (8a) was synthesized by the general Procedure **A** and purified by flash column chromatography on silica gel (gradient elution: Hexanes/EtOAc = 10:1-4:1) to give the desired product as white solid (97 mg, 88% yield); **^{1}H NMR** (400 MHz, $CDCl_3$) δ (ppm): 8.04 (s, 4H, ArH), 3.34 (t, *J* = 6.4

Hz, 2H), 2.85 (t, J = 6.4 Hz, 2H), 2.65 (s, 3H, CH_3); **^{13}C NMR** (100 MHz, DMSO-d6) δ (ppm): 198.9 (ArC=O), 198.2 (ArC=O), 174.2, 140.4, 140.0, 128.9, 128.6, 34.0, 28.3, 27.5; **HRMS** (ESI) m/z calcd for $C_{12}H_{12}O_4Na$ $[M+Na]^+$: 243.0628, found: 243.0623; **IR** (KBr, plate), ν (cm^{-1}): 1727, 1686 (C=O).

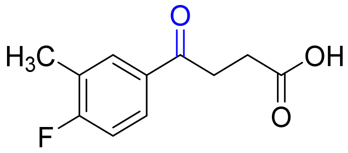

4-(4-Fluoro-3-methylphenyl)-4-oxobutanoic acid (9a) was synthesized by the general Procedure **A** and purified by flash column chromatography on silica gel (gradient elution: Hexanes/EtOAc = 10:1-4:1) to give the desired product as white solid (98 mg, 93% yield); **^{1}H NMR** (400 MHz, $CDCl_3$) δ (ppm): 7.86-7.79 (m, 2H, ArH), 7.07 (t, J = 9.0 Hz, 1H, ArH), 3.27 (t, J = 6.4 Hz, 2H), 2.81 (t, J = 6.4 Hz, 2H), 2.32 (s, 3H, $ArCH_3$); **^{13}C NMR** (100 MHz, $CDCl_3$) δ (ppm): 196.5 (ArC=O), 178.3, 164.6 (d, $J_{C\text{-}F}$ = 252.0 Hz), 132.6 (d, $J_{C\text{-}F}$ = 3.0 Hz), 131.9 (d, $J_{C\text{-}F}$ = 7.0 Hz), 128.0 (d, $J_{C\text{-}F}$ = 10.0 Hz), 125.4(d, $J_{C\text{-}F}$ = 18.0 Hz), 115.3(d, $J_{C\text{-}F}$ = 23.0 Hz), 33.0, 28.0, 14.0 (d, $J_{C\text{-}F}$ = 3.0 Hz); **HRMS** (ESI) m/z calcd for $C_{11}H_{11}FO_3Na$ $[M+Na]^+$: 233.0584, found: 233.0582; **IR** (KBr, plate), ν (cm^{-1}): 1678 (C=O).

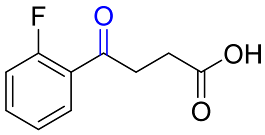

4-(2-Fluorophenyl)-4-oxobutanoic acid (10a) was synthesized by the general Procedure **A** and purified by flash column chromatography on silica gel (gradient elution: Hexanes/EtOAc = 10:1-4:1) to give the desired product as white solid (94 mg, 96% yield); **^{1}H NMR** (400 MHz, $CDCl_3$) δ (ppm): 7.93-7.89 (m, 1H, ArH), 7.56-7.51 (m, 1H, ArH), 7.26-7.22 (m, 1H, ArH), 7.17-7.12 (m, 1H, ArH), 3.32 (t, J = 6.4 Hz, 2H), 2.80 (t, J = 6.4 Hz, 2H); **^{13}C NMR** (100 MHz, $CDCl_3$) δ (ppm): 196.0 (d, $J_{C\text{-}F}$ = 4.0 Hz) (ArC=O), 178.3, 162.2 (d, $J_{C\text{-}F}$ = 253.0 Hz), 134.8 (d, $J_{C\text{-}F}$ = 9.0 Hz), 130.7 (d, $J_{C\text{-}F}$ = 3.0 Hz), 124.6 (d, $J_{C\text{-}F}$ = 3.2 Hz), 116.7 (d, $J_{C\text{-}F}$ = 23.0 Hz), 38.1 (d, $J_{C\text{-}F}$ = 9.0 Hz), 28.2; **HRMS** (ESI) m/z calcd for $C_{10}H_9FO_3Na$ $[M+Na]^+$: 219.0428, found: 219.0430; **IR** (KBr, plate), ν (cm^{-1}): 1690 (C=O).

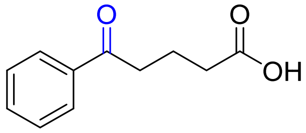

5-Oxo-5-phenylpentanoic acid (11a)[25] was synthesized by the general Procedure **A** and purified by flash column chromatography on silica gel (gradient elution: Hexanes/EtOAc = 10:1-4:1) to give the desired product as white solid (90 mg, 94% yield); **^{1}H NMR** (400 MHz, $CDCl_3$) δ (ppm): 7.96 (d, *J* = 8.0 Hz, 2H, ArH), 7.57 (t, *J* = 7.6 Hz, 1H, ArH), 7.48-7.44 (m, 2H, ArH), 3.09 (t, *J* = 7.2 Hz, 2H), 2.51 (t, *J* = 7.2 Hz, 2H), 2.13-2.06 (m, 2H); **^{13}C NMR** (100 MHz, $CDCl_3$) δ (ppm): 199.3 (ArC=O), 179.4, 136.7, 133.1, 128.6, 128.0, 37.3, 33.0, 18.9; **HRMS** (ESI) m/z calcd for $C_{11}H_{12}O_3Na$ $[M+Na]^+$: 215.0679, found: 215.0677; **IR** (KBr, plate), ν (cm^{-1}): 1726, 1685 (C=O).

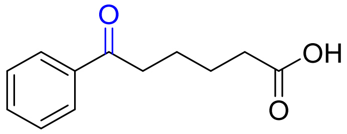

6-Oxo-6-phenylhexanoic acid (12a) [26] was synthesized by the general Procedure **A** and purified by flash column chromatography on silica gel (gradient elution: Hexanes/EtOAc = 10:1-4:1) to give the desired product as white solid (95 mg, 92% yield); **^{1}H NMR** (400 MHz, $CDCl_3$) δ (ppm): 7.95 (d, *J* = 8.0. Hz, 2H, ArH), 7.56 (t, *J* = 7.6 Hz, 1H, ArH), 7.48-7.44 (m, 2H, ArH), 3.01 (t, *J* = 7.2 Hz, 2H), 2.42 (t, *J* = 7.2 Hz, 2H), 1.83-1.72 (m, 4H); **^{13}C NMR** (100 MHz, $CDCl_3$) δ (ppm): 199.8 (ArC=O), 179.2, 136.9, 133.0, 128.6, 128.0, 38.1, 33.8, 24.3, 23.5; **HRMS** (ESI) m/z calcd for $C_{12}H_{14}O_3Na$ $[M+Na]^+$: 229.0835; found: 229.0825; **IR** (KBr, plate), ν (cm^{-1}): 1747, 1683 (C=O).

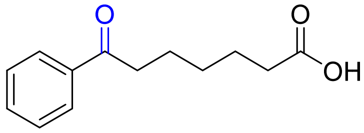

7-Oxo-7-phenylheptanoic acid (13a)[26]**:** was synthesized by the general Procedure **A** and purified by flash column chromatography on silica gel (gradient elution: Hexanes/EtOAc = 10:1-4:1) to give the desired product as white solid (99 mg, 90% yield); **^{1}H NMR** (400 MHz, $CDCl_3$) δ (ppm): 7.96-7.94 (m, 2H, ArH), 7.56 (t, *J* = 7.2 Hz, 1H, ArH), 7.48-7.44 (m, 2H, ArH), 2.98 (t, *J* = 7.6 Hz, 2H), 2.38 (t, *J* = 7.6 Hz, 2H), 1.81-1.66 (m, 4H), 1.49-1.41(m, 2H); **^{13}C NMR** (100 MHz, $CDCl_3$) δ (ppm):

200.2 (ArC=O), 179.6, 137.0, 132.9, 128.6, 128.0, 38.2, 33.8, 28.7, 24.5, 23.8; **HRMS** (ESI) m/z calcd for $C_{13}H_{16}O_3Na$ $[M+Na]^+$: 243.0992; found: 243.0990; **IR** (KBr, plate), ν (cm^{-1}): 1712 (C=O).

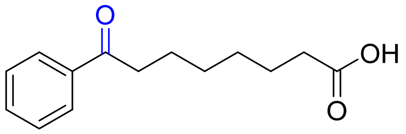

8-Oxo-8-phenyloctanoic acid (14a)[26] was synthesized by the general Procedure **A** and purified by flash column chromatography on silica gel (gradient elution: Hexanes/EtOAc = 10:1-4:1) to give the desired product as white solid (105 mg, 90 % yield); **^{1}H NMR** (400 MHz, $CDCl_3$) δ (ppm): 7.96-7.94 (m, 2H, ArH), 7.55 (t, *J* = 7.2 Hz, 1H, ArH), 7.48-7.44 (m, 2H, ArH), 2.96 (t, *J* = 7.2 Hz, 2H), 2.36 (t, *J* = 7.2 Hz, 2H), 1.77-1.64 (m, 4H), 1.43-1.39 (m, 4H); **^{13}C NMR** (100 MHz, $CDCl_3$) δ (ppm): 200.4 (ArC=O), 174.2, 137.1, 132.9, 128.5, 128.0, 51.4, 38.4, 34.0, 28.9, 24.8, 24.1; **HRMS** (ESI) m/z calcd for $C_{14}H_{18}O_3Na$ $[M+Na]^+$: 257.1148; found: 257.1144; **IR** (KBr, plate), ν (cm^{-1}): 1747, 1683 (C=O).

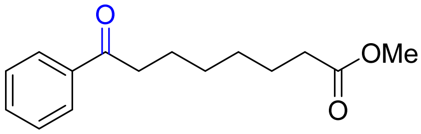

Methyl 8-oxo-8-phenyloctanoate (15a)[27] was synthesized by the general Procedure **A** and purified by flash column chromatography on silica gel (gradient elution: Hexanes/EtOAc = 30:1-5:1) to give the desired product as colorless liquid (112 mg, 90% yield); **^{1}H NMR** (400 MHz, $CDCl_3$) δ (ppm): 7.96-7.94 (m, 2H, ArH), 7.55 (t, *J* = 7.2 Hz, 1H, ArH), 7.48-7.44 (m, 2H, ArH), 3.66 (s, 3H, OCH_3), 2.96 (t, *J* = 7.4 Hz, 2H), 2.31 (t, *J* = 7.4 Hz, 2H), 1.76-1.71 (m, 2H), 1.68-1.61 (m, 2H), 1.40-1.37 (m, 4H); **^{13}C NMR** (100 MHz, $CDCl_3$) δ (ppm): 200.4 (ArC=O), 178.7, 137.1, 132.9, 128.4, 128.2, 38.4, 33.8, 29.7, 28.93, 28.86, 24.5, 24.1; **HRMS** (ESI) m/z calcd for $C_{15}H_{20}O_3Na$ $[M+Na]^+$: 271.1305; found: 271.1304; **IR** (KBr, plate), ν (cm^{-1}): 1774, 1724 (C=O).

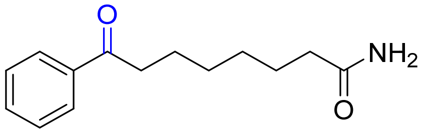

8-Oxo-8-phenyloctanamide (16a): was synthesized by the general Procedure **A** and purified by flash column chromatography on silica gel (gradient elution: Hexanes/EtOAc = 10:1-4:1) to give the desired product white solid (96 mg, 82% yield); **^{1}H NMR** (400 MHz, $CDCl_3$) δ (ppm): 7.96-7.94 (m, 2H, ArH), 7.57-7.53 (m, 1H, ArH), 7.48-7.44 (m, 2H, ArH), 2.97 (t, J = 7.4 Hz, 2H), 2.36 (t, J = 7.2 Hz, 2H), 1.77-1.64 (m, 4H), 1.42-1.39 (m, 4H); **^{13}C NMR** (100 MHz, $CDCl_3$) δ (ppm): 200.4 (ArC=O), 179.7 ($CONH_2$), 137.0, 132.9, 128.6, 128.0, 38.4, 33.9, 28.91, 28.85, 24.5, 24.1; **HRMS** (ESI) m/z calcd for $C_{14}H_{19}NONa$ $[M+Na]^+$: 256.1308; found: 256.1306; **IR** (KBr, plate), ν (cm^{-1}): 1745 (C=O).

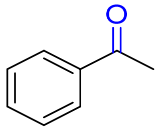

Acetophenone(18a)[28] was synthesized by the general Procedure **A** and purified by flash column chromatography on silica gel (gradient elution: Hexanes/EtOAc = 50:1-20:1) to give the desired product as colorless liquid (49 mg, 82% isolated yield, 94% GC yield); **^{1}H NMR** (400 MHz, $CDCl_3$) δ (ppm): 7.96 (d, J = 7.2 Hz, 2H, ArH), 7.58 (t, J = 8.0 Hz, 1H, ArH), 7.49-7.45 (m, 2H, ArH), 2.60 (s, 3H, CH_3); **^{13}C NMR** (100 MHz, $CDCl_3$) δ (ppm): 198.1 (ArC=O), 137.0, 133.0, 128.5, 128.2, 26.5.

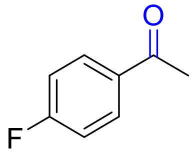

1-(4-Fluorophenyl)ethan-1-one (19a)[28] was synthesized by the general Procedure **A** and purified by flash column chromatography on silica gel (gradient elution: Hexanes/EtOAc = 50:1-20:1) to give the desired product as colorless liquid (59 mg, 86% isolated yield, 99% GC yield); **^{1}H NMR** (400 MHz, $CDCl_3$) δ (ppm): 8.00-7.96 (m, 2H, ArH), 7.15-7.10 (m, 2H, ArH), 2.58 (s, 3H, CH_3); **^{13}C NMR** (100 MHz, $CDCl_3$) δ (ppm): 196.5 (ArC=O), 165.8 (d, J_{C-F} = 253.0 Hz), 133.6 (d, J_{C-F} = 3.0 Hz), 130.9 (d, J_{C-F} = 10.0 Hz), 115.6 (d, J_{C-F} = 22.0 Hz), 26.5.

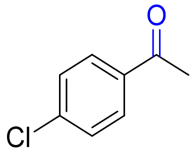

1-(4-Chlorophenyl)ethan-1-one (20a)[28] was synthesized by the general Procedure **A** and purified by flash column chromatography on silica gel (gradient elution: Hexanes/EtOAc = 50:1-20:1) to give the desired product as colorless liquid (66 mg, 86% isolated yield, 95% GC yield); **^{1}H NMR** (400 MHz, $CDCl_3$) δ (ppm): 7.89 (d, *J* = 8.8 Hz, 2H, ArH), 7.43 (d, *J* = 8.8 Hz, 2H, ArH), 2.59 (s, 3H, CH_3); **^{13}C NMR** (100 MHz, $CDCl_3$) δ (ppm): 196.7 (ArC=O), 139.5, 135.4, 129.6, 128.8, 26.5.

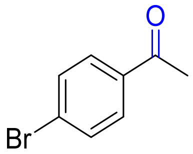

1-(4-Bromophenyl)ethan-1-one (21a)[29] was synthesized by the general Procedure **A** and purified by flash column chromatography on silica gel (gradient elution: Hexanes/EtOAc = 50:1-20:1) to give the desired product as white solid (83 mg, 84% isolated yield, 90% GC yield); **^{1}H NMR** (400 MHz, $CDCl_3$) δ (ppm): 7.82 (d, *J* = 8.4 Hz, 2H, ArH), 7.61 (d, *J* = 8.4 Hz, 2H, ArH), 2.59 (s, 3H, CH_3); **^{13}C NMR** (100 MHz, $CDCl_3$) δ (ppm): 196.9 (ArC=O), 135.7, 131.8, 129.7, 128.2, 26.4.

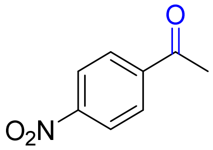

1-(4-Nitrophenyl)ethan-1-one (22a)[29] was synthesized by the general Procedure **A** and purified by flash column chromatography on silica gel (gradient elution: Hexanes/EtOAc = 30:1-10:1) to give the desired product as pale yellow crystal (66 mg, 80% isolated yield, 87% GC yield); **^{1}H NMR** (400 MHz, $CDCl_3$) δ (ppm): 8.30 (d, *J* = 8.8 Hz, 2H, ArH), 8.10 (d, *J* = 8.8 Hz, 2H, ArH), 2.67 (s, 3H, CH_3); **^{13}C NMR** (100 MHz, $CDCl_3$) δ (ppm): 196.3 (ArC=O), 150.3, 141.4, 129.3, 123.8, 26.9.

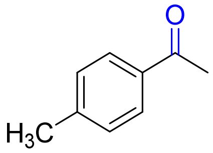

1-(*p*-Tolyl)ethan-1-one (23a)[29] was synthesized by the general Procedure **A** and purified by flash column chromatography on silica gel (gradient elution: Hexanes/EtOAc = 50:1-20:1) to give the desired product as colorless liquid (57 mg, 85% isolated yield, 96% GC yield) ; **^{1}H NMR** (400 MHz, $CDCl_3$) δ (ppm): 7.80 (d, *J*

= 8.0 Hz, 2H, ArH), 7.20 (d, J = 8.0 Hz, 2H, ArH), 2.51 (s, 3H, CH_3), 2.35 (s, 3H, $ArCH_3$); **^{13}C NMR** (100 MHz, $CDCl_3$) δ (ppm): 197.5 (ArC=O), 143.6, 134.5, 129.0, 128.2, 26.3, 21.4.

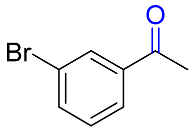

1-(3-Bromophenyl)ethan-1-one (24a)[29] was synthesized by the general Procedure **A** and purified by flash column chromatography on silica gel (gradient elution: Hexanes/EtOAc = 50:1-20:1) to give the desired product as colorless liquid (82 mg, 83% isolated yield, 91% GC yield); **^{1}H NMR** (400 MHz, $CDCl_3$) δ (ppm): 8.06 (s, 1H, ArH), 7.85 (d, J = 7.6 Hz, 1H, ArH), 7.66 (d, J = 8.0 Hz, 1H, ArH), 7.32 (t, J = 8.0 Hz, 1H, ArH), 2.57 (s, 3H, CH_3); **^{13}C NMR** (100 MHz, $CDCl_3$) δ (ppm): 196.5 (ArC=O), 138.7, 135.9, 131.3, 130.1, 126.8, 122.8, 26.5.

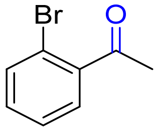

1-(2-Bromophenyl)ethan-1-one (25a)[29] was synthesized by the general Procedure **A** and purified by flash column chromatography on silica gel (gradient elution: Hexanes/EtOAc = 50:1-20:1) to give the desired product as colorless liquid (78 mg, 79% isolated yield, 85% GC yield); **^{1}H NMR** (400 MHz, $CDCl_3$) δ (ppm): 7.61 (d, J = 8.0 Hz, 1H, ArH), 7.46 (d, J = 7.6 Hz, 1H, ArH), 7.37 (t, J = 7.6 Hz, 1H, ArH), 7.29 (td, J = 7.6, 1.6 Hz, 1H, ArH), 2.63 (s, 3H); **^{13}C NMR** (100 MHz, $CDCl_3$) δ (ppm): 201.3 (ArC=O), 141.4, 133.8, 131.7, 128.9, 127.4, 118.9, 30.3.

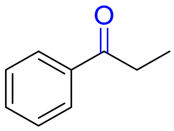

Propiophenone (26a)[29] was synthesized by the general Procedure **A** and purified by flash column chromatography on silica gel (gradient elution: Hexanes/EtOAc = 50:1-20:1) to give the desired product as colorless liquid (60 mg, 90% isolated yield, 95% GC yield); **^{1}H NMR** (400 MHz, $CDCl_3$) δ (ppm): 7.97 (d, J = 7.6 Hz, 2H, ArH), 7.56 (t, J = 7.6 Hz, 1H, ArH), 7.46 (t, J = 7.6 Hz, 2H, ArH), 3.01 (q, J = 7.2 Hz, 2H), 1.23

(t, J = 7.2 Hz, 3H); **^{13}C NMR** (100 MHz, $CDCl_3$) δ (ppm): 200.8 (ArC=O), 136.9, 132.9, 128.5, 128.0, 31.8, 8.2.

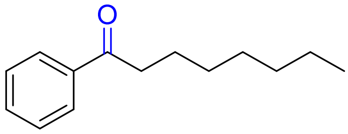

1-Phenyloctan-1-one (27a)[30] was synthesized by the general Procedure **A** and purified by flash column chromatography on silica gel (gradient elution: Hexanes/EtOAc = 20:1-5:1) to give the desired product colorless liquid (87 mg, 85 % yield); **^{1}H NMR** (400 MHz, $CDCl_3$) δ (ppm): 7.96 (d, J = 8.4 Hz, 2H, ArH), 7.55 (t, J = 7.6 Hz, 1H, ArH), 7.48-7.44 (m, 2H, ArH), 2.96 (t, J = 7.4 Hz, 2H), 1.77-1.70 (m, 2H), 1.38-1.26 (m, 8H), 0.88 (t, J = 6.4 Hz, 3H); **^{13}C NMR** (100 MHz, $CDCl_3$) δ (ppm): 200.6 (ArC=O), 137.1, 132.8, 128.5, 128.0, 38.6, 31.7, 29.3, 29.1, 24.4, 22.6, 14.1; **HRMS** (ESI) m/z calcd for $C_{14}H_{20}ONa$ $[M+Na]^+$: 227.1406; found: 227.1405; **IR** (KBr, plate), ν (cm^{-1}): 1745 (C=O).

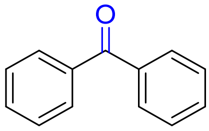

Benzophenone (28a) [29] **:** was synthesized by the general Procedure **A** and purified by flash column chromatography on silica gel (gradient elution: Hexanes/EtOAc = 10:1-4:1) to give the desired product white solid (86 mg, 95% yield); **^{1}H NMR** (400 MHz, $CDCl_3$) δ (ppm): 7.81 (d, J = 7.2 Hz, 4H, ArH), 7.59 (t, J = 7.2 Hz, 2H, ArH), 7.49 (t, J = 7.6 Hz, 4H, ArH); **^{13}C NMR** (100 MHz, $CDCl_3$) δ (ppm): 196.7 (ArC=O), 137.6, 132.3, 130.0, 128.2; **HRMS** (ESI) m/z calcd for $C_{13}H_{10}ONa$ $[M+Na]^+$: 205.0624; found: 205.0628; **IR** (KBr, plate), ν (cm^{-1}): 1652 (C=O).

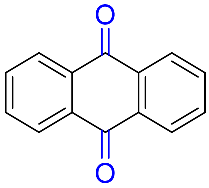

Anthracene-9,10-dione (29a)[31]**:** was synthesized by the general Procedure **A (*rac*-1 (4 mol%))** and purified by flash column chromatography on silica gel (gradient elution: Hexanes/EtOAc = 20:1-4:1) to give the desired product as white solid (97 mg, 93% yield); **^{1}H NMR** (400 MHz, $CDCl_3$) δ (ppm): 8.35-8.31 (m, 4H, ArH), 7.83-7.79 (m,

4H, ArH); **^{13}C NMR** (100 MHz, $CDCl_3$) δ (ppm): 183.2 (ArC=O), 134.1, 133.6, 127.3; **HRMS** (ESI) m/z calcd for $C_{14}H_8O_2Na$ $[M+H]^+$: 209.0597; found: 209.0602; **IR** (KBr, plate), ν (cm^{-1}): 1689 (C=O).

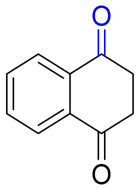

2,3-Dihydronaphthalene-1,4-dione (30a)[32] was synthesized by the general Procedure **A** and purified by flash column chromatography on silica gel (gradient elution: Hexanes/EtOAc = 10:1-4:1) to give the desired product as colorless liquid (77 mg, 96% yield); **^{1}H NMR** (400 MHz, $CDCl_3$) δ (ppm): 8.08-8.04 (m, 2H, ArH), 7.78-7.74 (m, 2H, ArH), 3.10 (s, 4H); **^{13}C NMR** (100 MHz, $CDCl_3$) δ (ppm): 195.8(ArC=O), 135.1, 134.1, 126.5, 37.4; **HRMS** (ESI) m/z calcd for $C_{10}H_8O_2Na$ $[M+Na]^+$: 183.0417, found: 183.0417; **IR** (KBr, plate), ν (cm^{-1}): 1659 (C=O).

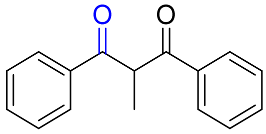

2-Methyl-1,3-diphenylpropan-1-one (31a)[33] was synthesized by the general Procedure **A** and purified by flash column chromatography on silica gel (gradient elution: Hexanes/EtOAc = 10:1-4:1) to give the desired product as white solid (111 mg, 93% yield); **^{1}H NMR** (400 MHz, $CDCl_3$) δ (ppm): 7.96 (d, J = 7.6 Hz, 4H, ArH), 7.56 (t, J = 7.6 Hz, 2H, ArH), 7.45 (t, J = 8.0 Hz, 4H, ArH), 5.28 (q, J = 7.2 Hz, 1H), 1.60 (d, J = 7.2 Hz, 3H, CH_3); **^{13}C NMR** (100 MHz, $CDCl_3$) δ (ppm): 197.2 (ArC=O), 135.6, 133.5, 128.9, 128.5, 51.0, 14.3; **HRMS** (ESI) m/z calcd for $C_{16}H_{14}O_2Na$ $[M+Na]^+$: 261.0886, found: 261.0890; **IR** (KBr, plate), ν (cm^{-1}): 1686 (C=O).

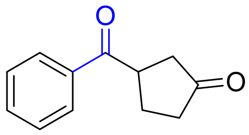

3-Benzoylcyclopentan-1-one (32a)[34] was synthesized by the general Procedure **A** and purified by flash column chromatography on silica gel (gradient elution: Hexanes/EtOAc = 10:1-4:1) to give the desired product as white solid (92 mg, 98 % yield); **^{1}H NMR** (400 MHz, $CDCl_3$) δ (ppm): 8.00 (d, J = 7.2 Hz, 2H, ArH), 7.61 (t, J = 7.4 Hz, 1H, ArH), 7.51 (t, J = 7.6 Hz, 2H, ArH), 4.17-4.09 (m, 1H), 2.75-2.68 (m,

1H), 2.50-2.28 (m, 4H), 2.23-2.15 (m, 1H); **^{13}C NMR** (100 MHz, $CDCl_3$) δ (ppm): 216.8 (C=O), 200.2 (ArC=O), 135.6, 133.5, 128.8, 128.4, 43.0, 41.0, 37.3, 26.9; **HRMS** (ESI) m/z calcd for $C_{12}H_{12}O_2Na$ $[M+Na]^+$: 211.0730, found: 211.0735; **IR** (KBr, plate), ν (cm^{-1}): 1727, 1684 (C=O) .

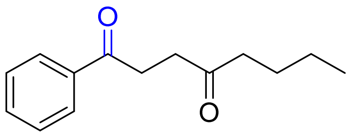

1-Phenyloctane-1,4-dione (33a)[35] was synthesized by the general Procedure **A** and purified by flash column chromatography on silica gel (gradient elution: Hexanes/EtOAc = 20:1-4:1) to give the desired product as colorless liquid (96 mg, 88% yield); **^{1}H NMR** (400 MHz, $CDCl_3$) δ (ppm): 7.98 (d, *J* = 7.6 Hz, 2H, ArH), 7.57 (t, *J* = 7.6 Hz, 1H, ArH), 7.46 (t, *J* = 7.2 Hz, 2H, ArH), 3.28 (t, *J* = 6.4 Hz, 2H), 2.86 (t, *J* = 6.4 Hz, 2H), 2.53 (t, *J* = 7.2 Hz, 2H), 1.65-1.57 (m, 2H), 1.39-1.30 (m, 2H), 0.89 (t, *J* = 7.2 Hz, 3H); **^{13}C NMR** (100 MHz, $CDCl_3$) δ (ppm): 209.8 (C=O), 198.7 (ArC=O), 136.7, 133.1, 128.5, 128.0, 42.7, 36.2, 32.4, 26.0, 22.3, 13.8; **HRMS** (ESI) m/z calcd for $C_{14}H_{18}O_2Na$ $[M+Na]^+$: 241.1199; found: 241.1198; **IR** (KBr, plate), ν (cm^{-1}): 1747, 1697 (C=O).

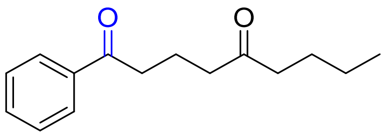

1-Phenylnonane-1,5-dione (34a)[36] was synthesized by the general Procedure **A** and purified by flash column chromatography on silica gel (gradient elution: Hexanes/EtOAc = 20:1-4:1) to give the desired product as colorless liquid (104 mg, 90% yield); **^{1}H NMR** (400 MHz, $CDCl_3$) δ (ppm): 7.98 (d, *J* = 7.6 Hz, 2H, ArH), 7.58 (t, *J* = 7.4 Hz, 1H, ArH), 7.45 (t, *J* = 7.4 Hz, 2H, ArH), 3.01 (t, *J* = 7.0 Hz, 2H), 2.54 (t, *J* = 7.0 Hz, 2H), 2.40 (t, *J* = 7.6 Hz, 2H), 2.05-1.98 (m, 2H), 1.65-1.51 (m, 2H), 1.35-1.25 (m, 2H), 0.90 (t, *J* = 7.2 Hz, 3H); **^{13}C NMR** (100 MHz, $CDCl_3$) δ (ppm): 210.9 (C=O), 199.8 (ArC=O), 136.8, 133.0, 128.6, 128.0, 42.6, 41.6, 37.5, 25.9, 22.3, 18.3, 13.8; **HRMS** (ESI) m/z calcd for $C_{15}H_{20}O_2Na$ $[M+Na]^+$: 255.1356; found: 255.1355; **IR** (KBr, plate), ν (cm^{-1}): 1707, 1677 (C=O).

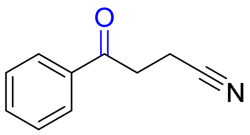

4-Oxo-4-phenylbutanenitrile (35a)[34] was synthesized by the general Procedure **A** and purified by flash column chromatography on silica gel (gradient elution: Hexanes/EtOAc = 10:1-4:1) to give the desired product as colorless liquid (75 mg, 94% yield); **^{1}H NMR** (400 MHz, $CDCl_3$) δ (ppm): 7.94 (d, *J* = 7.6 Hz, 2H, ArH), 7.61 (t, *J* = 7.4 Hz, 1H, ArH), 7.49 (t, *J* = 7.8 Hz, 2H, ArH), 3.37 (t, *J* = 7.2 Hz, 2H), 2.77 (t, *J* = 7.2 Hz, 2H); **^{13}C NMR** (100 MHz, $CDCl_3$) δ (ppm): 195.3 (ArC=O), 135.6, 133.8, 128.8, 128.0, 119.2, 34.2, 11.7; **HRMS** (ESI) m/z calcd for $C_{10}H_9NONa$ $[M+Na]^+$: 182.0576, found: 182.0576; **IR** (KBr, plate), ν (cm^{-1}): 2171 (C≡N), 1668 (C=O).

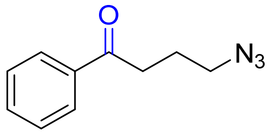

4-Azido-1-phenylbutan-1-one (36a)[37] was synthesized by the general Procedure **A** and purified by flash column chromatography on silica gel (gradient elution: Hexanes/EtOAc = 10:1-4:1) to give the desired product as colorless liquid (90 mg, 95% yield); **^{1}H NMR** (400 MHz, $CDCl_3$) δ (ppm): 7.96 (d, *J* = 7.6 Hz, 2H, ArH), 7.57 (t, *J* = 7.4 Hz, 1H, ArH), 7.47 (t, *J* = 7.6 Hz, 2H, ArH), 3.43 (t, *J* = 6.6 Hz, 2H), 3.09 (t, *J* = 7.0 Hz, 2H), 2.08-2.01 (m, 2H); **^{13}C NMR** (100 MHz, $CDCl_3$) δ (ppm): 198.3 (ArC=O), 136.7, 133.2, 128.6, 128.0, 38.1, 13.6, 3.5 **HRMS** (ESI) m/z calcd for $C_{10}H_{11}N_3ONa$ $[M+Na]^+$: 212.0794, found: 212.0794; **IR** (KBr, plate), ν (cm^{-1}): 2102 (N_3), 1686 (C=O).

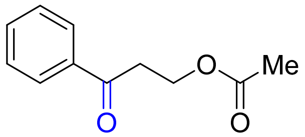

3-Oxo-3-phenylpropyl acetate (37a)[38] was synthesized by the general Procedure **A** and purified by flash column chromatography on silica gel (gradient elution: Hexanes/EtOAc = 10:1-4:1) to give the desired product as wite solid (88 mg, 92% yield); **^{1}H NMR** (400 MHz, $CDCl_3$) δ (ppm): 7.96 (d, *J* = 7.0 Hz, 2H, ArH), 7.60 (t, *J* = 7.2 Hz, 1H, ArH), 7.48 (t, *J* = 7.2 Hz, 2H, ArH), 4.52 (t, *J* = 6.4 Hz, 2H), 3.32 (t, *J* = 6.4 Hz, 2H), 2.03 (s, 3H); **^{13}C NMR** (100 MHz, $CDCl_3$) δ (ppm): 197.0 (ArC=O), 171.0 (OC=O), 136.6, 133.4, 128.7, 128.0, 59.6, 37.3, 20.9; **HRMS** (ESI) m/z calcd for $C_{11}H_{12}O_3Na$ $[M+Na]^+$: 215.0679, found: 215.0676; **IR** (KBr, plate), ν (cm^{-1}): 1738，1656 (C=O).

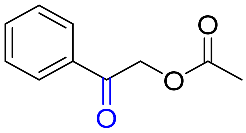

2-Oxo-2-phenylethyl acetate (38a)[38] was synthesized by the general Procedure **A** and purified by flash column chromatography on silica gel (gradient elution: Hexanes/EtOAc = 10:1-4:1) to give the desired product as wite solid (68 mg, 76% yield); **^{1}H NMR** (400 MHz, $CDCl_3$) δ (ppm): 7.91 (d, J = 7.2 Hz, 2H, ArH), 7.60 (t, J = 7.2 Hz, 1H, ArH), 7.48 (t, J = 7.2 Hz, 2H, ArH), 5.34 (s, 2H), 2.22 (s, 3H); **^{13}C NMR** (100 MHz, $CDCl_3$) δ (ppm): 192.1 (ArC=O), 171.4 (OC=O), 134.2, 133.9, 128.8, 127.7, 66.0, 20.5; **HRMS** (ESI) m/z calcd for $C_{10}H_{10}O_3Na$ $[M+Na]^+$: 201.0522, found: 215.0525; **IR** (KBr, plate), ν (cm^{-1}): 1740，1703 (C=O).

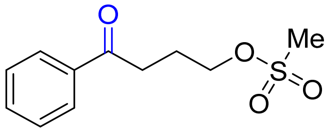

4-Oxo-4-phenylbutyl methanesulfonate (39a) was synthesized by the general Procedure **A** and purified by flash column chromatography on silica gel (gradient elution: Hexanes/EtOAc = 10:1-4:1) to give the desired product as colorless liquid (114 mg, 94% yield); **^{1}H NMR** (400 MHz, $CDCl_3$) δ (ppm): 7.96 (d, J = 8.0 Hz, 2H, ArH), 7.58 (t, J = 7.4 Hz, 1H, ArH), 7.49-7.45 (m, 2H, ArH), 4.36 (t, J = 6.2 Hz, 2H), 3.15 (t, J = 6.8 Hz, 2H), 3.00 (s, 3H, SO_2CH_3), 2.25-2.18 (m, 2H); **^{13}C NMR** (100 MHz, $CDCl_3$) δ (ppm): 198.6 (ArC=O), 136.6, 133.3, 128.7, 128.0, 69.4, 37.3, 34.0, 23.6; **HRMS** (ESI) m/z calcd for $C_{11}H_{14}O_4SNa$ $[M+Na]^+$: 265.0505, found: 265.0500; **IR** (KBr, plate), ν (cm^{-1}): 1687 (C=O).

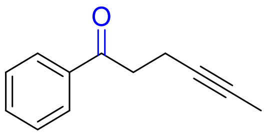

1-Phenylhex-4-yn-1-one (40a)[39] was synthesized by the general Procedure **A** and purified by flash column chromatography on silica gel (gradient elution: Hexanes/EtOAc = 100:1-5:1) to give the desired product as colorless liquid (64 mg, 74% yield); **^{1}H NMR** (400 MHz, $CDCl_3$) δ (ppm): 7.98-7.96 (d, J = 7.6 Hz, 2H, ArH), 7.56 (t, J = 7.4 Hz, 1H, ArH), 7.46 (t, J = 7.4 Hz, 2H, ArH), 3.19 (t, J = 7.4 Hz, 2H), 2.59-2.54 (m, 2H), 1.76 (t, J = 2.5 Hz, 3H); **^{13}C NMR** (100 MHz, $CDCl_3$) δ (ppm): 198.3 (ArC=O), 136.7, 133.2, 128.6, 128.0, 77.9, 76.1, 38.1, 13.6, 3.5. **HRMS** (ESI)

m/z calcd for $C_{12}H_{12}ONa$ [M+Na]$^+$: 195.0780, found: 195.0777. **IR** (KBr, plate), ν (cm^{-1}): 1680 (C=O).

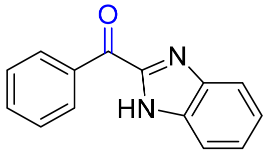

(1H-Benzo[*d*]imidazol-2-yl)(phenyl)methanone (41a)[40] was synthesized by the general Procedure **A** and purified by flash column chromatography on silica gel (gradient elution: Hexanes/EtOAc = 10:1-4:1) to give the desired product as white solid (83 mg, 75% yield); **^{1}H NMR** (400 MHz, $CDCl_3$) δ (ppm): 10.51 (s, 1H, NH), 8.71 (d, *J* = 7.6 Hz, 2H, ArH), 7.98 (d, *J* = 8.1 Hz, 1H, ArH), 7.67 (t, *J* = 7.4 Hz, 1H, ArH), 7.61-7.56 (m, 3H, ArH), 7.46 (t, *J* = 7.6 Hz, 1H, ArH), 7.39 (t, *J* = 8.0 Hz, 1H, ArH); **^{13}C NMR** (100 MHz, $CDCl_3$) δ (ppm): 183.9 (ArC=O), 147.8, 144.0, 135.4, 133.9, 133.1, 131.3, 128.5, 126.5, 123.8, 122.3, 111.9; **HRMS** (ESI) m/z calcd for $C_{14}H_{10}N_2ONa$ [M+Na]$^+$: 245.0685, found: 245.0688; **IR** (KBr, plate), ν (cm^{-1}): 1672 (ArC=O).

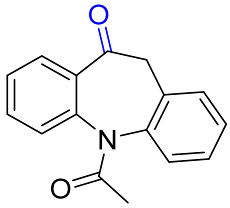

5-Acetyl-5,11-dihydro-10H-dibenzo[*b*, *f*]azepin-10-one (42a)[41] was synthesized by the general Procedure **A** and purified by flash column chromatography on silica gel (gradient elution: Hexanes/EtOAc = 10:1-4:1) to give the desired product as white solid (117 mg, 93% yield); **^{1}H NMR** (400 MHz, $CDCl_3$) δ (ppm): 8.11 (d, *J* = 7.9 Hz, 1H, ArH), 7.61-7.57 (m, 2H, ArH), 7.42-7.31 (m, 5H, ArH), 4.35 (d, *J* = 14.6 Hz, 1H), 3.86 (d, *J* = 14.6 Hz, 1H), 2.12 (s, 3H); **^{13}C NMR** (100 MHz, $CDCl_3$) δ (ppm): 191.6 (ArC=O), 169.6, 142.3, 133.8, 130.6, 129.9, 129.0, 128.7, 128.4, 127.6, 48.9, 23.0. **HRMS** (ESI) m/z calcd for $C_{16}H_{13}NO_2Na$ [M+Na]$^+$: 274.0838; found: 274.0846; **IR** (KBr, plate), ν (cm^{-1}): 1672 (C=O).

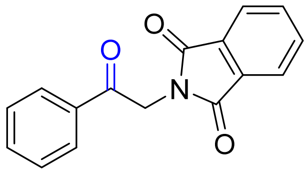

2-(2-Oxo-2-phenylethyl)isoindoline-1,3-dione (43a)[42] was synthesized by the general Procedure **A** and purified by flash column chromatography on silica gel (gradient elution: Hexanes/EtOAc = 10:1-4:1) to give the desired product as white solid (118 mg, 89% yield). **^{1}H NMR** (400 MHz, $CDCl_3$) δ (ppm): 8.01 (d, *J* = 7.2 Hz, 2H, ArH), 7.91-7.89 (m, 2H, ArH), 7.76-7.74 (m, 2H, ArH), 7.63 (t, *J* = 7.6 Hz, 1H, ArH), 7.51 (t, *J* = 7.6 Hz, 2H, ArH), 5.14 (s, 2H); **^{13}C NMR** (100 MHz, $CDCl_3$) δ (ppm): 190.9 (ArC=O), 167.9 (C=O), 134.4, 134.2, 134.1, 134.0, 132.2, 128.8, 128.1, 123.5, 44.2; **HRMS** (ESI) m/z calcd for $C_{16}H_{11}NO_3Na$ $[M+Na]^+$: 288.0631; found: 288.0640; **IR** (KBr, plate), ν (cm^{-1}): 1721, 1689 (C=O).

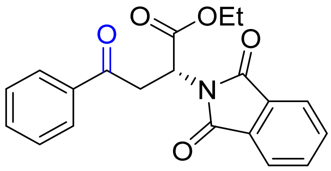

Ethyl 2-(1,3-dioxoisoindolin-2-yl)-4-oxo-4-phenylbutanoate (44a)[43] was synthesized by the general Procedure **A** and purified by flash column chromatography on silica gel (gradient elution: Hexanes/EtOAc = 20:1-4:1) to give the desired product as white solid (167 mg, 95% yield). **^{1}H NMR** (400 MHz, $CDCl_3$) δ (ppm): 7.95 (d, *J* = 7.6 Hz, 2H, ArH), 7.85-7.82 (m, 2H, ArH), 7.72-7.68 (m, 2H, ArH), 7.54 (t, *J* = 7.2 Hz, 1H, ArH), 7.45-7.41 (m, 2H, ArH), 5.69 (dd, *J* = 8.0, 3.2 Hz, 1H, CH), 4.26-4.14 (m, 2H), 4.06 (dd, *J* = 18.0, 5.6 Hz, 1H), 3.83 (dd, *J* = 18.0, 7.2 Hz, 1H), 1.18 (t, *J* = 7.2 Hz, 3H); **^{13}C NMR** (100 MHz, $CDCl_3$) δ (ppm): 195.7 (ArC=O), 168.8 (C=O), 167.3 (C=O), 136.1, 134.1, 133.4, 131.7, 128.6, 128.1, 123.5, 62.2, 47.8, 37.8, 13.9; **HRMS** (ESI) m/z calcd for $C_{20}H_{17}NO_5Na$ $[M+Na]^+$: 374.0999; found: 374.0999; **IR** (KBr, plate), ν (cm^{-1}): 1743, 1721, 1688 (C=O).

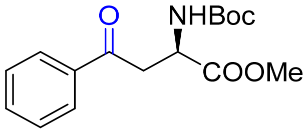

(2*R*)-2-*tert*-Butoxycarbonylamino)-4-oxo-4-phenylbutyric acid methyl ester (45a)[44] was synthesized by the general Procedure **A** and purified by flash column chromatography on silica gel (gradient elution: Hexanes/EtOAc = 20:1-4:1) to give the desired product as white solid (143 mg, 93% yield); **^{1}H NMR** (400 MHz, $CDCl_3$) δ (ppm): 7.95-7.93 (m, 2H, ArH), 7.61-7.57 (m, 1H, ArH), 7.49-7.46 (m, 2H, ArH), 5.63 (d, *J* = 8.0 Hz, 1H, NH), 4.72-4.68 (m, 1H), 3.76-3.71 (m, 1H), 3.74 (s, 3H), 3.56-3.51

(m, 1H), 1.44 (s, 9H); **^{13}C NMR** (100 MHz, $CDCl_3$) δ (ppm): 197.8 (ArC=O), 171.9, 155.6, 136.1, 133.7, 128.7, 128.1, 80.0, 52.6, 49.6, 40.9, 28.3; **HRMS** (ESI) m/z calcd for $C_{16}H_{21}NO_5Na$ $[M+Na]^+$: 330.1312; found: 330.1318; **IR** (KBr, plate), ν (cm^{-1}): 1710, 1685 (C=O). Chiral HPLC (Daicel Chiralcel OD-H, 2.5% isopropanol-hexanes rate 1.0 mL/min): t_{major} = 19.26 min., t_{minor} = 27.74 min.; 99% *ee*.

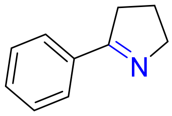

5-Phenyl-3,4-dihydro-*2H*-pyrrole (46a)[45] was synthesized by the general Procedure **B** and purified by flash column chromatography on silica gel (gradient elution: Hexanes/EtOAc = 10:1-4:1) to give the desired product as colorless liquid (67 mg, 93% yield); **^{1}H NMR** (400 MHz, $CDCl_3$) δ (ppm): 7.85-7.82 (m, 2H, ArH), 7.41-7.36 (m, 3H, ArH), 4.09-4.06 (m, 2H), 2.97-2.93 (m, 2H), 2.08-2.02 (m, 2H);**^{13}C NMR** (100 MHz, $CDCl_3$) δ (ppm): 173.3 (C=N), 134.6, 130.3, 128.4, 127.6, 61.5, 34.9, 22.7; **HRMS** (ESI) m/z calcd for $C_{10}H_{12}N$ $[M+H]^+$: 146.0964; found: 146.0965; **IR** (KBr, plate), ν (cm^{-1}): 1615 (C=N).

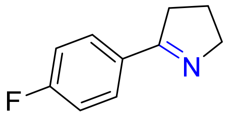

5-(4-Fluorophenyl)-3,4-dihydro-*2H*-pyrrole (47a)[45] was synthesized by the general Procedure **B** and purified by flash column chromatography on silica gel (gradient elution: Hexanes/EtOAc = 10:1-4:1) to give the desired product as colorless liquid (77 mg, 94% yield); **^{1}H NMR** (400 MHz, $CDCl_3$) δ (ppm): 7.85-7.80 (m, 2H, ArH), 7.08 (t, *J* = 8.7 Hz, 2H, ArH), 4.05 (t, *J* = 7.2 Hz, 2H, $C=NCH_2$), 2.95-2.88 (m, 2H), 2.09-1.98 (m, 2H); **^{13}C NMR** (100 MHz, $CDCl_3$) δ (ppm): 172.2 (C=N), 164.2 (d, $J_{C\text{-}F}$ = 249.0 Hz), 130.7 (d, $J_{C\text{-}F}$ = 3.0 Hz), 129.7(d, $J_{C\text{-}F}$ = 9.0 Hz) 115.4 (d, $J_{C\text{-}F}$ = 22.0 Hz), 61.4, 35.0, 22.6; **HRMS** (ESI) m/z calcd for $C_{10}H_{11}FN$ $[M+H]^+$: 164.0870; found: 164.0868; **IR** (KBr, plate), ν (cm^{-1}): 1610 (C=N).

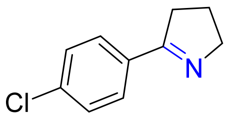

5-(4-Chlorophenyl)-3,4-dihydro-*2H*-pyrrole (48a)[45] was synthesized by the general Procedure **B** and purified by flash column chromatography on silica gel (gradient

elution: Hexanes/EtOAc = 10:1-4:1) to give the desired product as colorless liquid (83 mg, 93% yield); ^{1}**H NMR** (400 MHz, $CDCl_3$) δ (ppm): 7.77 (d, *J* = 8.4 Hz, 2H, ArH), 7.38 (d, *J* = 8.4 Hz, 2H, ArH), 4.09-4.04 (m, 2H), 2.95-2.88 (m, 2H), 2.10-1.99 (m, 2H); ^{13}**C NMR** (100 MHz, $CDCl_3$) δ (ppm): 172.1 (C=N), 136.3, 133.0, 128.8, 128.6, 61.5, 34.8, 22.7; **HRMS** (ESI) m/z calcd for $C_{10}H_{11}ClN$ $[M+H]^+$: 180.0575, found: 180.0573; **IR** (KBr, plate), ν (cm^{-1}): 1611 (C=N).

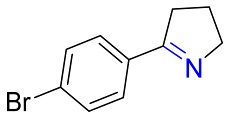

5-(4-Bromophenyl)-3,4-dihydro-*2H*-pyrrole (49a)[45] was synthesized by the general Procedure **B** and purified by flash column chromatography on silica gel (gradient elution: Hexanes/EtOAc = 10:1-4:1) to give the desired product as colorless liquid (103 mg, 92% yield); ^{1}**H NMR** (400 MHz, $CDCl_3$) δ (ppm): 7.70 (d, *J* = 8.4 Hz, 2H, ArH), 7.53 (d, *J* = 8.4 Hz, 2H, ArH), 4.07-4.03 (m, 2H), 2.93-2.89 (m, 2H), 2.08-2.00 (m, 2H); ^{13}**C NMR** (100 MHz, $CDCl_3$): δ (ppm) 172.3, 133.5, 131.6, 129.1, 124.8, 61.7, 34.9, 22.7; **HRMS** (ESI) m/z calcd for $C_{10}H_{11}BrN$ $[M+H]^+$: 224.0069, found: 224.0068; **IR** (KBr, plate), ν (cm^{-1}): 1611 (C=N).

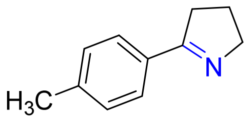

5-(*p*-Tolyl)-3,4-dihydro-*2H*-pyrrole (50a)[45] was synthesized by the general Procedure **B** and purified by flash column chromatography on silica gel (gradient elution: Hexanes/EtOAc = 10:1-4:1) to give the desired product as colorless liquid (73 mg, 92% yield); ^{1}**H NMR** (400 MHz, $CDCl_3$) δ (ppm): 7.73 (d, *J* = 8.0 Hz, 2H, ArH), 7.21 (d, *J* = 8.0 Hz, 2H, ArH), 4.06-4.03 (m, 2H), 2.95-2.91(m, 2H), 2.38 (s, 3H), 2.04-2.00 (m, 2H); ^{13}**C NMR** (100 MHz, $CDCl_3$) δ (ppm): 173.2 (C=N), 140.5, 131.8, 129.1, 127.5, 61.3, 34.9, 22.6, 21.4; HRMS (ESI) m/z calcd for $C_{11}H_{14}N$ $[M+H]^+$: 160.1120; found: 160.1121; **IR** (KBr, plate), ν (cm^{-1}):1609 (C=N).

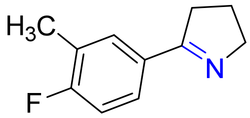

5-(4-Fluoro-3-methylphenyl)-3,4-dihydro-*2H*-pyrrole (51a) was synthesized by the general Procedure **B** and purified by flash column chromatography on silica gel

(gradient elution: Hexanes/EtOAc = 10:1-4:1) to give the desired product as colorless liquid (80 mg, 90% yield); **^{1}H NMR** (400 MHz, $CDCl_3$) δ (ppm): 7.74 (d, *J* = 7.6 Hz, 1H, ArH), 7.62-7.58 (m, 1H, ArH), 7.02 (t, *J* = 8.8 Hz, 1H, ArH), 4.06-4.03 (m, 2H), 2.94-2.89 (m, 2H), 2.30 (s, 3H), 2.07-2.00 (m, 2H); **^{13}C NMR** (100 MHz, $CDCl_3$) δ (ppm): 172.4 (C=N), 162.7 (d, *J* = 247.0 Hz), 130.9 (d, *J* = 5.7 Hz), 130.5 (d, *J* = 3.5 Hz), 127.0 (d, *J* = 8.5 Hz), 125.0 (d, *J* = 17.5 Hz), 115.0 (d, *J* = 22.6 Hz), 61.4, 34.9, 22.8, 14.5 (d, *J* = 3.5 Hz); **HRMS** (ESI) m/z calcd for $C_{11}H_{13}FN$ $[M+H]^+$: 178.1027, found: 178.1027; **IR** (KBr, plate), ν (cm^{-1}): 1618 (C=N).

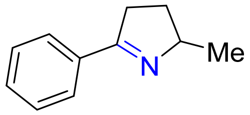

2-Methyl-5-phenyl-3,4-dihydro-*2H*-pyrrole (52a)[46] was synthesized by the general Procedure **B** and purified by flash column chromatography on silica gel (gradient elution: Hexanes/EtOAc = 10:1-4:1) to give the desired product as colorless liquid (65 mg, 82% yield); **^{1}H NMR** (400 MHz, $CDCl_3$) δ (ppm): 7.85-7.82 (m, 2H, ArH), 7.42-7.39 (m, 3H, ArH), 4.33-4.25 (m, 1H), 3.10-3.02 (m, 1H), 2.93-2.83 (m, 1H), 2.27-2.22 (m, 1H), 1.60-1.51 (m, 1H), 1.36 (d, *J* = 6.8 Hz, 3H); **^{13}C NMR** (100 MHz, $CDCl_3$) δ (ppm): 171.8 (C=N), 134.6, 130.2, 128.3, 127.6, 68.3, 35.1, 30.6, 22.0; **HRMS** (ESI) m/z calcd for $C_{11}H_{14}N$ $[M+H]^+$: 160.1121, found: 160.1119; **IR** (KBr, plate), ν (cm^{-1}): 1615 (C=N).

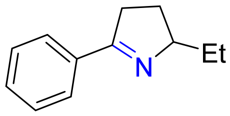

2-Ethyl-5-phenyl-3,4-dihydro-*2H*-pyrrole (53a)[46] was synthesized by the general Procedure **B** and purified by flash column chromatography on silica gel (gradient elution: Hexanes/EtOAc = 10:1-4:1) to give the desired product as colorless liquid (68 mg, 79% yield); **^{1}H NMR** (400 MHz, $CDCl_3$) δ (ppm): 7.78-7.76 (m, 2H, ArH), 7.34-7.32 (m, 3H, ArH), 4.10-4.03 (m, 1H), 2.98-2.90 (m, 1H), 2.86-2.77 (m, 1H), 2.16-2.07 (m, 1H), 1.86-1.75 (m, 1H), 1.59-1.44 (m, 2H), 0.95 (t, *J* = 7.2 Hz, 3H); **^{13}C NMR** (100 MHz, $CDCl_3$) δ (ppm): 171.9 (C=N), 134.8, 130.2, 128.4, 127.6, 74.3, 35.0, 29.4, 28.0, 10.9; **HRMS** (ESI) m/z calcd for $C_{12}H_{16}N$ $[M+H]^+$: 174.1277, found: 174.1280; **IR** (KBr, plate), ν (cm^{-1}): 1623 (C=N).

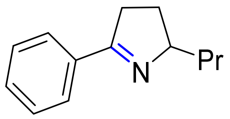

5-Phenyl-2-propyl-3,4-dihydro-*2H*-pyrrole (54a)[46] was synthesized by the general Procedure **B** and purified by flash column chromatography on silica gel (gradient elution: Hexanes/EtOAc = 10:1-4:1) to give the desired product as colorless liquid (69 mg, 74% yield); **^{1}H NMR** (400 MHz, $CDCl_3$) δ (ppm): 7.86-7.83 (m, 2H, ArH), 7.41-7.39 (m, 3H, ArH), 4.21-4.18 (m, 1H), 3.05-2.97 (m, 1H), 2.91-2.82 (m, 1H), 2.23-2.15 (m, 1H), 1.85-1.80 (m, 1H), 1.65-1.57 (m, 1H), 1.52-1.45 (m, 3H), 0.98 (t, *J* = 7.2 Hz, 3H); **^{13}C NMR** (100 MHz, $CDCl_3$) δ (ppm): 171.7 (C=N), 134.7, 130.2, 128.3, 127.6, 73.0, 39.9, 34.9, 28.5, 19.9, 14.2; **HRMS** (ESI) m/z calcd for $C_{13}H_{18}N$ $[M+H]^+$: 188.1434, found: 188.1438; **IR** (KBr, plate), ν (cm^{-1}): 1615 (C=N).

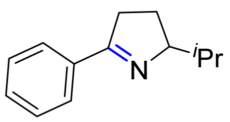

2-(*iso*-Propyl)-5-phenyl-3,4-dihydro-*2H*-pyrrole (55a)[47] was synthesized by the general Procedure **B** and purified by flash column chromatography on silica gel (gradient elution: Hexanes/EtOAc = 10:1-4:1) to give the desired product as colorless liquid (68 mg, 73% yield); **^{1}H NMR** (400 MHz, $CDCl_3$) δ (ppm): 7.85-7.83 (m, 2H, ArH), 7.41-7.38 (m, 3H, ArH), 4.13-4.06 (m, 1H), 2.96-2.89 (m, 2H), 2.10-1.96 (m, 2H), 1.72-1.66 (m, 1H), 1.07 (d, *J* = 6.8 Hz, 3H), 0.90 (d, *J* = 6.8 Hz, 3H); **^{13}C NMR** (100 MHz, $CDCl_3$) δ (ppm): 172.1 (C=N), 134.6, 130.2, 128.3, 127.6, 78.8, 35.3, 33.3, 24.8, 19.9, 18.2; **HRMS** (ESI) m/z calcd for $C_{13}H_{18}N$ $[M+H]^+$: 188.1434, found: 188.1439; **IR** (KBr, plate), ν (cm^{-1}): 1616 (C=N).

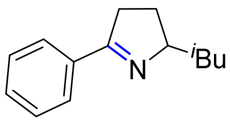

2-(*iso*-Butyl)-5-phenyl-3,4-dihydro-*2H*-pyrrole (56a)[46] was synthesized by the general Procedure **B** and purified by flash column chromatography on silica gel (gradient elution: Hexanes/EtOAc = 10:1-4:1) to give the desired product as colorless liquid (91 mg, 90% yield); **^{1}H NMR** (400 MHz, $CDCl_3$) δ (ppm): 7.85-7.83 (m, 2H, ArH), 7.40-7.38 (m, 3H, ArH), 4.27-4.20 (m, 1H), 3.06-2.98 (m, 1H), 2.92-2.83 (m, 1H), 2.25-2.17 (m, 1H), 1.91-1.81 (m, 1H), 1.78-1.72 (m, 1H), 1.62-1.52 (m, 1H), 1.35-1.28 (m, 1H), 1.01 (d, *J* = 6.4 Hz, 3H), 0.98 (d, *J* = 6.4 Hz, 3H); **^{13}C NMR** (100 MHz,

$CDCl_3$) δ (ppm): 171.6 (C=N), 134.8, 130.2, 128.3, 127.6, 71.5, 46.2, 34.8, 29.0, 25.9, 23.4, 22.5; **HRMS** (ESI) m/z calcd for $C_{14}H_{20}N$ $[M+H]^+$: 202.1590, found : 202.1591; **IR** (KBr, plate), ν (cm^{-1}): 1615 (C=N).

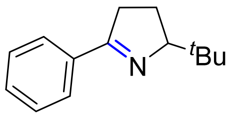

2-(*tert*-Butyl)-5-phenyl-3,4-dihydro-*2H*-pyrrole (57a) was synthesized by the general Procedure **B** and purified by flash column chromatography on silica gel (gradient elution: Hexanes/EtOAc = 10:1-4:1) to give the desired product as colorless liquid (72 mg, 72% yield); **^{1}H NMR** (400 MHz, $CDCl_3$) δ (ppm): 7.87-7.84 (m, 2H, ArH), 7.43-7.37 (m, 3H, ArH), 4.00-3.96 (m, 1H), 2.94-2.89 (m, 2H), 2.06-1.97 (m, 1H), 1.82-1.72 (m, 1H), 1.00 (s, 9H); **^{13}C NMR** (100 MHz, $CDCl_3$) δ (ppm): 171.9 (C=N), 134.9, 130.1, 128.3, 127.6, 82.5, 35.4, 34.8, 26.6, 23.8; **HRMS** (ESI) m/z calcd for $C_{14}H_{20}N$ $[M+H]^+$: 202.1590, found: 202.1586; **IR** (KBr, plate), ν (cm^{-1}): 1617 (C=N).

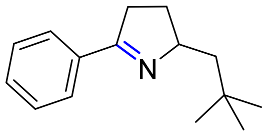

2-Neopentyl-5-phenyl-3,4-dihydro-*2H*-pyrrole (58a) was synthesized by the general Procedure **B** and purified by flash column chromatography on silica gel (gradient elution: Hexanes/EtOAc = 10:1-4:1) to give the desired product as colorless liquid (82 mg, 76% yield); **^{1}H NMR** (400 MHz, $CDCl_3$) δ (ppm): 7.85-7.82 (m, 2H, ArH), 7.40-7.39 (m, 3H, ArH), 4.24-4.21 (m, 1H), 3.06-2.98 (m, 1H), 2.91-2.82 (m, 1H), 2.33-2.24 (m, 1H), 1.93 (dd, J = 13.8, 4.4 Hz, 1H), 1.65-1.58 (m, 1H), 1.35 (dd, J = 13.8, 8.0 Hz, 1H), 1.04 (s, 9H); **^{13}C NMR** (100 MHz, $CDCl_3$) δ (ppm): 171.1 (C=N), 134.8, 130.1, 128.3, 127.6, 70.5, 50.9, 35.2, 31.4, 30.5, 30.2; **HRMS** (ESI) m/z calcd for $C_{15}H_{22}N$ $[M+H]^+$: 216.1747, found: 216.1741; **IR** (KBr, plate), ν (cm^{-1}): 1615 (C=N).

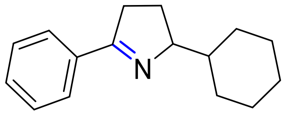

2-Cyclohexyl-5-phenyl-3,4-dihydro-*2H*-pyrrole (59a) was synthesized by the general Procedure **B** and purified by flash column chromatography on silica gel (gradient elution: Hexanes/EtOAc = 10:1-4:1) to give the desired product as colorless

liquid (84 mg, 74% yield); **^{1}H NMR** (400 MHz, $CDCl_3$) δ (ppm): 7.85-7.83 (m, 2H, ArH), 7.41-7.39 (m, 3H, ArH), 4.02-3.97 (m, 1H), 2.99-2.81 (m, 2H), 2.11-2.04 (m, 2H), 1.80-1.55 (m, 6H), 1.33-1.02 (m, 5H); **^{13}C NMR** (100 MHz, $CDCl_3$) δ (ppm): 171.7 (C=N), 134.9, 130.1, 128.3, 127.6, 78.3, 43.7, 35.1, 30.6, 29.0, 26.7, 26.4, 26.3, 25.6; **HRMS** (ESI) m/z calcd for $C_{16}H_{22}N$ $[M+H]^+$: 228.1747, found: 228.1742; **IR** (KBr, plate), ν (cm^{-1}): 1617 (C=N).

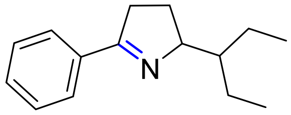

2-(Pentan-3-yl)-5-phenyl-3,4-dihydro-*2H*-pyrrole (60a) was synthesized by the general Procedure **B** and purified by flash column chromatography on silica gel (gradient elution: Hexanes/EtOAc = 10:1-4:1) to give the desired product as colorless liquid (91 mg, 85% yield); **^{1}H NMR** (400 MHz, $CDCl_3$) δ (ppm): 7.85-7.83 (m, 2H, ArH), 7.41-7.38 (m, 3H, ArH), 4.32-4.27 (m, 1H), 2.99-2.83 (m, 2H), 2.11-2.03 (m, 1H), 1.72-1.52 (m, 3H), 1.48-1.37 (m, 2H), 1.26-1.16 (m, 1H), 0.99 (t, *J* = 7.2 Hz, 3H), 0.93 (t, *J* = 7.2 Hz, 3H); **^{13}C NMR** (100 MHz, $CDCl_3$) δ (ppm): 171.5 (C=N), 134.9, 130.0, 128.3, 127.5, 75.4, 46.2, 35.2, 24.9, 23.2, 21.8, 11.8, 11.7; **HRMS** (ESI) m/z calcd for $C_{15}H_{22}N$ $[M+H]^+$: 216.1747, found: 216.1741; **IR** (KBr, plate), ν (cm-1): 1616 (C=N).

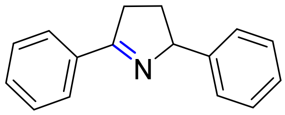

2,5-Diphenyl-3,4-dihydro-*2H*-pyrrole (61a)[48] was synthesized by the general Procedure **B** and purified by flash column chromatography on silica gel (gradient elution: Hexanes/EtOAc = 10:1-4:1) to give the desired product as pale yellow liquid (82 mg, 74% yield); **^{1}H NMR** (400 MHz, $CDCl_3$) δ (ppm): 7.96-7.93 (m, 2H, ArH), 7.45-7.41 (m, 3H, ArH), 7.34-7.29 (m, 4H, ArH), 7.26-7.23 (m, 1H, ArH), 5.33-5.29 (m, 1H), 3.20-3.12 (m, 1H), 3.04-2.95 (m, 1H), 2.63-2.54 (m, 1H), 1.94-1.85 (m, 1H); **^{13}C NMR** (100 MHz, $CDCl_3$) δ (ppm): 173.6 (C=N), 146.6, 134.4, 130.5, 128.4, 127.8, 126.8, 126.5, 76.0, 35.5, 32.4. **HRMS** (ESI) m/z calcd for $C_{16}H_{16}N$ $[M+H]^+$: 222.1277, found: 222.1270; **IR** (KBr, plate), ν (cm^{-1}): 1611 (C=N).

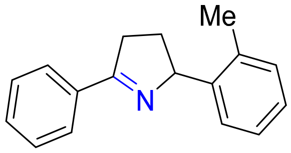

5-Phenyl-2-(*o*-tolyl)-3,4-dihydro-*2H*-pyrrole (62a) was synthesized by the general Procedure **B** and purified by flash column chromatography on silica gel (gradient elution: Hexanes/EtOAc = 10:1-4:1) to give the desired product as pale yellow liquid (85 mg, 72% yield); **^{1}H NMR** (400 MHz, $CDCl_3$) δ (ppm): 7.99-7.97 (m, 2H, ArH), 7.48-7.45 (m, 3H, ArH), 7.19-7.14 (m, 4H, ArH), 5.54-5.51 (m, 1H), 3.20-3.11 (m, 1H), 3.08-2.99 (m, 1H), 2.68-2.59 (m, 1H), 2.43 (s, 3H), 1.84-1.74 (m, 1H); **^{13}C NMR** (100 MHz, $CDCl_3$) δ (ppm): 173.6 (C=N), 142.9, 134.54, 134.45, 130.5, 130.1, 128.4, 127.8, 126.6, 126.1, 125.5, 72.9, 35.3, 31.2, 19.5; **HRMS** (ESI) m/z calcd for $C_{17}H_{18}N$ $[M+H]^+$: 236.1434, found: 236.1432; **IR** (KBr, plate), ν (cm^{-1}): 1615 (C=N).

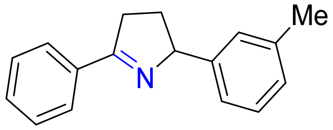

5-Phenyl-2-(*m*-tolyl)-3,4-dihydro-*2H*-pyrrole (63a) was synthesized by the general Procedure **B** and purified by flash column chromatography on silica gel (gradient elution: Hexanes/EtOAc = 10:1-4:1) to give the desired product as pale yellow liquid (86 mg, 73% yield); **^{1}H NMR** (400 MHz, $CDCl_3$) δ (ppm): 7.98-7.95 (m, 2H, ArH), 7.47-7.44 (m, 3H, ArH), 7.24-7.22 (m, 1H, ArH), 7.13-7.06 (m, 3H, ArH), 5.31-5.27 (m, 1H), 3.23-3.15 (m, 1H), 3.06-2.96 (m, 1H), 2.64-2.55 (m, 1H), 2.36 (s, 3H), 1.96-1.87 (m, 1H); **^{13}C NMR** (100 MHz, $CDCl_3$) δ (ppm): 173.5 (C=N), 144.6, 138.0, 134.5, 130.5, 128.4, 128.3, 127.9, 127.5, 127.2, 123.6, 76.1, 35.6, 32.4, 21.5; **HRMS** (ESI) m/z calcd for $C_{17}H_{18}N$ $[M+H]^+$: 236.1434, found : 236.1429; **IR** (KBr, plate), ν (cm^{-1}): 1615 (C=N).

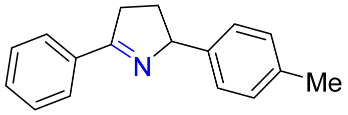

5-Phenyl-2-(*p*-tolyl)-3,4-dihydro-*2H*-pyrrole (64a): was synthesized by the general Procedure **B** and purified by flash column chromatography on silica gel (gradient elution: Hexanes/EtOAc = 10:1-4:1) to give the desired product as pale yellow liquid (87 mg, 74% yield); **^{1}H NMR** (400 MHz, $CDCl_3$) δ (ppm): 7.96-7.93 (m, 2H, ArH), 7.46-7.41 (m, 3H, ArH), 7.20 (d, *J* = 7.4 Hz, 2H, ArH), 7.14 (d, *J* = 7.4 Hz, 2H, ArH), 5.31-5.27 (m, 1H), 3.21-3.13 (m, 1H), 3.05-2.96 (m, 1H), 2.62-2.54 (m, 1H), 2.34 (s, 3H), 1.94-1.85 (m, 1H); **^{13}C NMR** (100 MHz, $CDCl_3$) δ (ppm): 173.4 (C=N), 141.6, 136.3, 134.5, 130.5, 129.1, 128.4, 127.9, 126.4, 75.8, 35.5, 32.5, 21.1; **HRMS** (ESI)

m/z calcd for $C_{17}H_{18}N$ $[M+H]^+$: 236.1434, found: 236.1425; **IR** (KBr, plate), ν (cm^{-1}): 1612 (C=N).

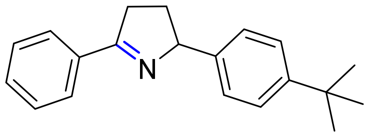

2-(4-(*tert*-Butyl)phenyl)-5-phenyl-3,4-dihydro-*2H*-pyrrole (65a): was synthesized by the general Procedure **B** and purified by flash column chromatography on silica gel (gradient elution: Hexanes/EtOAc = 10:1-4:1) to give the desired product as pale yellow liquid (104 mg, 75% yield); **^{1}H NMR** (400 MHz, $CDCl_3$) δ (ppm): 7.95-7.93 (m, 2H, ArH), 7.45-7.42 (m, 3H, ArH), 7.36 (d, *J* = 7.6 Hz, 2H, ArH), 7.24 (d, *J* = 7.6 Hz, 2H, ArH), 5.30-5.27 (m, 1H), 3.22-3.14 (m, 1H), 3.05-2.96 (m, 1H), 2.62-2.53 (m, 1H), 1.99-1.89 (m, 1H), 1.31 (s, 9H); **^{13}C NMR** (100 MHz, $CDCl_3$) δ (ppm): 173.3 (C=N), 149.6, 141.5, 134.5, 130.5, 128.4, 127.9, 126.3, 125.4, 75.8, 35.6, 34.4, 32.2, 31.4; **HRMS** (ESI) m/z calcd for $C_{20}H_{24}N$ $[M+H]^+$: 278.1903, found: 278.1907; **IR** (KBr, plate), ν (cm^{-1}): 1609 (C=N).

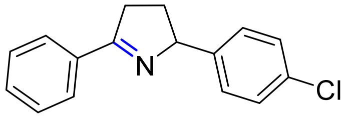

2-(4-Chlorophenyl)-5-phenyl-3,4-dihydro-*2H*-pyrrole (66a): was synthesized by the general Procedure **B** and purified by flash column chromatography on silica gel (gradient elution: Hexanes/EtOAc = 10:1-4:1) to give the desired product as pale yellow liquid (93 mg, 73% yield); **^{1}H NMR** (400 MHz, $CDCl_3$) δ (ppm): 7.86-7.84 (m, 2H, ArH), 7.37-7.35 (m, 3H, ArH), 7.22 (d, *J* = 7.6 Hz, 2H, ArH), 7.16 (d, *J* = 7.6 Hz, 2H, ArH), 5.21-5.18 (m, 1H), 3.12-3.05 (m, 1H), 2.97-2.88 (m, 1H), 2.56-2.47 (m, 1H), 1.81-1.71 (m, 1H); **^{13}C NMR** (100 MHz, $CDCl_3$) δ (ppm): 174.0 (C=N), 143.2, 134.3, 132.5, 130.7, 128.54, 128.48, 127.91, 127.87, 75.3, 35.6, 32.4; **HRMS** (ESI) m/z calcd for $C_{16}H_{15}ClN$ $[M+H]^+$: 256.0888, found: 256.0890; **IR** (KBr, plate), ν (cm^{-1}): 1612 (C=N).

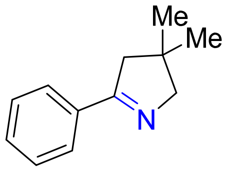

3,3-Dimethyl-5-phenyl-3,4-dihydro-*2H*-pyrrole (67a)[49] was synthesized by the general Procedure **B** and purified by flash column chromatography on silica gel (gradient elution: Hexanes/EtOAc = 10:1-4:1) to give the desired product as colorless liquid (81 mg, 94% yield); ^{1}**H NMR** (400 MHz, $CDCl_3$) δ (ppm): 7.82-7.79 (m, 2H, ArH), 7.41-7.40 (m, 3H, ArH), 3.79 (s, 2H), 2.79 (s, 2H), 1.18 (s, 6H); ^{13}**C NMR** (100 MHz, $CDCl_3$) δ (ppm): 172.9 (C=N), 134.9, 130.3, 128.4, 127.4, 74.8, 49.9, 38.5, 28.1; **HRMS** (ESI) m/z calcd for $C_{12}H_{16}N$ $[M+H]^+$: 174.1277, found: 174.1278; **IR** (KBr, plate), ν (cm^{-1}): 1615(C=N).

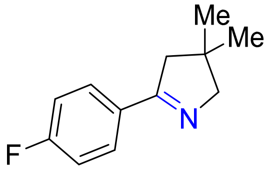

5-(4-Fluorophenyl)-3,3-dimethyl-3,4-dihydro-*2H*-pyrrole (68a) was synthesized by the general Procedure **B** and purified by flash column chromatography on silica gel (gradient elution: Hexanes/EtOAc = 10:1-4:1) to give the desired product as colorless liquid (91 mg, 95% yield); ^{1}**H NMR** (400 MHz, $CDCl_3$) δ (ppm): 7.81-7.78 (m, 2H, ArH), 7.10-7.06 (m, 2H, ArH), 3.78 (s, 2H), 2.76 (s, 2H), 1.18 (s, 6H); ^{13}**C NMR** (100 MHz, $CDCl_3$) δ (ppm): 171.7 (C=N), 164.1 (d, J_{C-F} = 250.0 Hz), 131.1 (d, J_{C-F} = 3.0 Hz), 129.5 (d, J = 9.0 Hz), 115.3 (d, J = 22.0 Hz), 74.6, 49.9, 38.6, 28.0; **HRMS** (ESI) m/z calcd for $C_{12}H_{15}FN$ $[M+H]^+$: 192.1183, found: 192.1193; **IR** (KBr, plate), ν (cm^{-1}): 1610 (C=N).

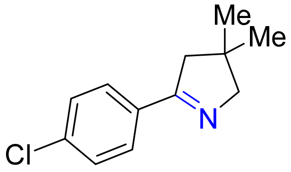

5-(4-Chlorophenyl)-3,3-dimethyl-3,4-dihydro-*2H*-pyrrole (69a) was synthesized by the general Procedure **B** and purified by flash column chromatography on silica gel (gradient elution: Hexanes/EtOAc = 10:1-4:1) to give the desired product as colorless liquid (96 mg, 93% yield); ^{1}**H NMR** (400 MHz, $CDCl_3$) δ (ppm): 7.73 (d, J = 8.4 Hz, 2H, ArH), 7.37 (t, J = 8.4 Hz, 2H, ArH), 3.79 (s, 2H), 2.75 (s, 2H), 1.17 (s, 6H); ^{13}**C NMR** (100 MHz, $CDCl_3$) δ (ppm): 171.8(C=N), 136.4, 133.3, 128.7, 128.6, 74.8, 49.9, 38.6, 28.1; **HRMS** (ESI) m/z calcd for $C_{12}H_{15}ClN$ $[M+H]^+$: 208.0888, found: 208.0893; **IR** (KBr, plate), ν (cm^{-1}): 1611 (C=N).

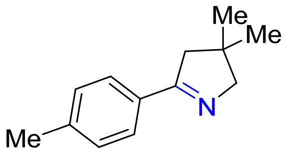

3,3-Dimethyl-6-(*p*-tolyl)-2,3,4,5-tetrahydropyridine (70a) was synthesized by the general Procedure **B** and purified by flash column chromatography on silica gel (gradient elution: Hexanes/EtOAc = 10:1-4:1) to give the desired product as colorless liquid (89 mg, 95% yield); **^{1}H NMR** (400 MHz, $CDCl_3$) δ (ppm): 7.69 (d, *J* = 8.0 Hz, 2H, ArH), 7.20 (d, *J* = 8.0 Hz, 2H, ArH), 3.78 (s, 2H), 2.77 (s, 2H), 2.38 (s, 3H), 1.17 (s, 6H); **^{13}C NMR** (100 MHz, $CDCl_3$) δ (ppm): 173.0 (C=N), 140.6, 132.0, 129.1, 127.4, 74.4, 49.9, 38.4, 28.1, 21.4; **HRMS** (ESI) m/z calcd for $C_{13}H_{18}N$ $[M+H]^+$: 188.1434, found: 188.1444; **IR** (KBr, plate), ν (cm^{-1}): 1610 (C=N).

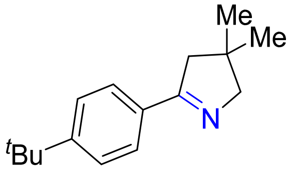

5-(4-*tert*-Butylphenyl)-3,3-dimethyl-3,4-dihydro-*2H*-pyrrole (71a) was synthesized by the general Procedure **B** and purified by flash column chromatography on silica gel (gradient elution: Hexanes/EtOAc = 10:1-4:1) to give the desired product as colorless liquid (105 mg, 92% yield); **^{1}H NMR** (400 MHz, $CDCl_3$) δ (ppm): 7.74 (d, *J* = 8.4 Hz, 2H, ArH), 7.42 (d, *J* = 8.4 Hz, 2H, ArH), 3.78 (s, 2H), 2.77 (s, 2H), 1.33 (s, 9H), 1.16 (s, 6H); **^{13}C NMR** (100 MHz, $CDCl_3$) δ (ppm): 172.7 (C=N), 153.7, 132.0, 127.2, 125.3, 74.5, 49.9, 38.4, 34.8, 31.2, 28.0; **HRMS** (ESI) m/z calcd for $C_{16}H_{24}N$ $[M+H]^+$: 230.1903, found: 230.1913; **IR** (KBr, plate), ν (cm^{-1}): 1615 (C=N).

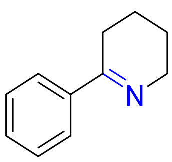

6-Phenyl-2,3,4,5-tetrahydropyridine (72a)[45] was synthesized by the general Procedure **B** and purified by flash column chromatography on silica gel (gradient elution: Hexanes/EtOAc = 10:1-4:1) to give the desired product as colorless liquid (64 mg, 80% yield); **^{1}H NMR** (400 MHz, $CDCl_3$) δ (ppm): 7.76-7.42 (m, 2H, ArH), 7.37-7.36 (m, 3H, ArH), 3.83-3.80 (m, 2H), 2.64-2.61 (m, 2H), 1.87-1.80 (m, 2H), 1.70-1.64 (m, 2H); **^{13}C NMR** (100 MHz, $CDCl_3$) δ (ppm): 165.6 (C=N), 140.2, 129.5, 128.2,

125.9, 49.8, 27.0, 21.8, 19.7; **HRMS** (ESI) m/z calcd for $C_{11}H_{14}N$ [M+H]$^+$: 160.1121, found: 160.1119; **IR** (KBr, plate), ν (cm^{-1}): 1615 (C=N).

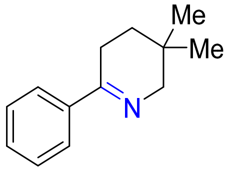

6-Phenyl-2,3,4,5-tetrahydropyridine (73a) was synthesized by the general Procedure **B** and purified by flash column chromatography on silica gel (gradient elution: Hexanes/EtOAc = 10:1-4:1) to give the desired product colorless liquid (80 mg, 85% yield); **^{1}H NMR** (400 MHz, $CDCl_3$) δ (ppm): 7.80-7.77 (m, 2H, ArH), 7.38-7.36 (m, 3H, ArH), 3.55 (s, 2H), 2.68-2.63 (m, 2H), 1.58 (t, *J* = 6.8 Hz, 2H), 0.95 (s, 6H); **^{13}C NMR** (100 MHz, $CDCl_3$) δ (ppm): 164.7 (C=N), 139.7, 129.5, 128.2, 125.9, 62.2, 32.6, 27.5, 26.3, 25.2; **HRMS** (ESI) m/z calcd for $C_{13}H_{18}N$ [M+H]$^+$: 188.1434, found: 188.1444; **IR** (KBr, plate), ν (cm^{-1}): 1616 (C=N).

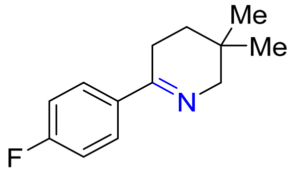

6-(4-Fluorophenyl)-3,3-dimethyl-2,3,4,5-tetrahydropyridine (74a) was synthesized by the general Procedure **B** and purified by flash column chromatography on silica gel (gradient elution: Hexanes/EtOAc = 10:1-4:1) to give the desired product as colorless liquid (88 mg, 86% yield); **^{1}H NMR** (400 MHz, $CDCl_3$) δ (ppm): 7.80-7.77 (m, 2H, ArH), 7.07-7.03 (m, 2H, ArH), 3.54 (s, 2H), 2.65-2.61 (m, 2H), 1.59 (t, *J* = 6.8 Hz, 2H), 0.95 (s, 6H); **^{13}C NMR** (100 MHz, $CDCl_3$) δ (ppm): 163.8 (d, *J* = 247.0 Hz), 163.5 (C=N), 135,9 (d, *J* = 3.0 Hz), 127.9 (d, *J* = 8.0 Hz), 115.0 (d, *J* = 22.0 Hz), 62.1, 32.6, 27.5, 26.3, 25.2; **HRMS** (ESI) m/z calcd for $C_{13}H_{17}FN$ [M+H]$^+$: 206.1340, found: 206.1349; **IR** (KBr, plate), ν (cm^{-1}): 1615 (C=N).

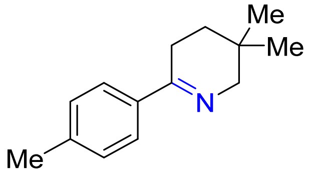

3,3-Dimethyl-6-(*p*-tolyl)-2,3,4,5-tetrahydropyridine (75a) was synthesized by the general Procedure **B** and purified by flash column chromatography on silica gel

(gradient elution: Hexanes/EtOAc = 10:1-4:1) to give the desired product as colorless liquid (87 mg, 86% yield); **^{1}H NMR** (400 MHz, $CDCl_3$) δ (ppm): 7.68 (d, J = 8.0 Hz, 2H, ArH), 7.18 (d, J = 8.0 Hz, 2H, ArH), 3.53 (s, 2H), 2.66-2.62 (m, 2H), 2.36 (s, 3H), 1.58 (t, J = 6.8 Hz, 2H), 0.95 (s, 6H); **^{13}C NMR** (100 MHz, $CDCl_3$) δ (ppm): 164.4 (C=N), 139.5, 137.0, 128.9, 125.9, 62.2, 32.7, 27.5, 26.3, 25.1, 21.2; **HRMS** (ESI) m/z calcd for $C_{14}H_{20}N$ $[M+H]^+$: 202.1590, found: 202.1595; **IR** (KBr, plate), ν (cm^{-1}): 1609 (C=N).

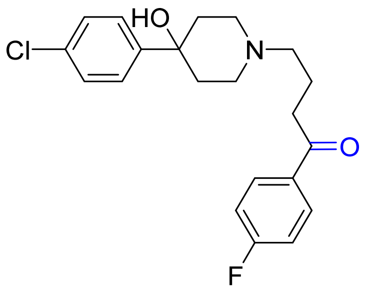

4-(4-(4-Chlorophenyl)-4-hydroxypiperidin-1-yl)-1-(4-fluorophenyl)butan-1-one (76a) [50] was synthesized by the general Procedure **C** and purified by flash column chromatography on silica gel (gradient elution: Hexanes/EtOAc = 10:1-4:1) to give the desired product as white solid (84 mg, 90% yield); **^{1}H NMR** (400 MHz, $CDCl_3$) δ (ppm): 8.03-8.00 (m, 2H, ArH), 7.38 (d, J = 8.4 Hz, 2H, ArH), 7.29 (t, J = 8.4 Hz, 2H, ArH), 7.13 (t, J = 8.4 Hz, 2H, ArH), 2.99 (t, J = 6.8 Hz, 2H), 2.80 (d, J = 10.8 Hz, 2H), 2.52-2.43 (m, 4H), 2.05-1.97 (m, 4H), 1.70-1.64 (m, 3H); **^{13}C NMR** (100 MHz, $CDCl_3$) δ (ppm): 198.3 (C=O), 165.6 (d, J_{C-F} = 253.0 Hz), 146.9, 133.6 (d, J_{C-F} = 3.0 Hz), 132.7, 130.6 (d, J_{C-F} = 9.0 Hz), 128.3, 126.1, 115.5 (d, J_{C-F} = 22.0 Hz), 71.0, 57.8, 49.3, 38.3, 36.2, 21.8; **HRMS** (ESI) m/z calcd for $C_{21}H_{24}ClFNO_2$ $[M+H]^+$: 376.1474, found: 376.1475; **IR** (KBr, plate), ν (cm^{-1}): 1683 (C=O).

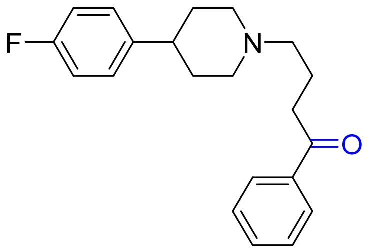

4-(4-(4-Fluorophenyl)piperidin-1-yl)-1-phenylbutan-1-one (77a) was synthesized by the general Procedure **C** and purified by flash column chromatography on silica gel (gradient elution: Hexanes/EtOAc = 10:1-4:1) to give the desired product as white solid (74 mg, 91% yield); **^{1}H NMR** (400 MHz, $CDCl_3$) δ (ppm): 8.00-7.98 (m, 2H, ArH),

7.58-7.52 (m, 1H, ArH), 7.49-7.45 (m, 2H, ArH), 7.16-7.12 (m, 2H, ArH), 6.99-6.95 (m, 2H, ArH), 3.10-3.01 (m, 4H), 2.53-2.45 (m, 3H), 2.14-2.08 (m, 2H), 2.05-1.98 (m, 3H) , 1.80-1.69 (m, 3H); ^{13}**C NMR** (100 MHz, $CDCl_3$) δ (ppm): 199.9 (C=O), 161.3 (d, J_{C-F} = 242.0 Hz), 141.8, 137.1, 132.9, 128.5, 128.1 (d, J_{C-F} = 7.0 Hz), 126.6, 115.1 (d, J_{C-F} = 21.0 Hz), 57.9, 54.0, 41.8, 36.3, 33.2, 21.5; **HRMS** (ESI) m/z calcd for $C_{21}H_{25}FNO$ $[M+H]^+$: 326.1915, found: 326.1912; **IR** (KBr, plate), ν (cm^{-1}): 1684 (C=O).

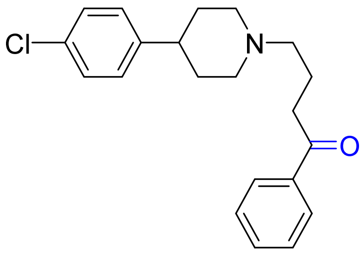

4-(4-(4-Chlorophenyl)piperidin-1-yl)-1-phenylbutan-1-one (78a) was synthesized by the general Procedure **C** and purified by flash column chromatography on silica gel (gradient elution: Hexanes/EtOAc = 20:1-4:1) to give the desired product as white solid (77 mg, 90% yield); ^{1}**H NMR** (400 MHz, $CDCl_3$) δ (ppm): 7.99 (d, J = 7.6 Hz, 2H, ArH), 7.56 (t, J = 7.6 Hz, 1H, ArH), 7.47 (t, J = 7.6 Hz, 2H, ArH), 7.24 (d, J = 8.4 Hz, 2H, ArH), 7.11 (d, J = 8.4 Hz, 2H, ArH), 3.02 (t, J = 7.2 Hz, 4H), 2.47-2.43 (m, 3H), 2.08-1.95 (m, 4H), 1.78-1.62 (m, 4H); ^{13}**C NMR** (100 MHz, $CDCl_3$) δ (ppm): 200.0 (C=O), 144.9, 137.2, 132.9, 131.7, 128.5, 128.4, 128.2, 128.1, 58.1, 54.1, 42.1, 36.4, 33.3, 21.9; **HRMS** (ESI) m/z calcd for $C_{21}H_{25}ClNO$ $[M+H]^+$: 342.1619, found: 342.1623; **IR** (KBr, plate), ν (cm^{-1}): 1684 (C=O).

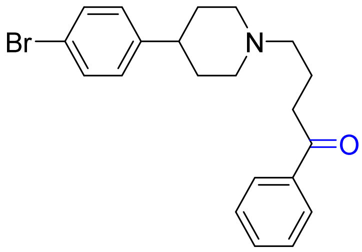

4-(4-(4-Bromophenyl)piperidin-1-yl)-1-phenylbutan-1-one (79a): was synthesized by the general Procedure **C** and purified by flash column chromatography on silica gel (gradient elution: Hexanes/EtOAc = 20:1-4:1) to give the desired product as white solid (85 mg, 88% yield); ^{1}**H NMR** (400 MHz, $CDCl_3$) δ (ppm): 7.99 (d, J = 7.6 Hz, 2H, ArH), 7.56 (t, J = 7.6 Hz, 1H, ArH), 7.46 (t, J = 7.6 Hz, 2H, ArH), 7.39 (d, J = 8.4 Hz,

2H, ArH), 7.05 (d, J = 8.4 Hz, 2H, ArH), 3.01 (t, J = 7.2 Hz, 4H), 2.46-2.41 (m, 3H), 2.07-1.95 (m, 4H), 1.78-1.72 (m, 2H), 1.65 (dt, J = 12.4, 3.6 Hz, 2H); **^{13}C NMR** (100 MHz, $CDCl_3$) δ (ppm): 200.0 (C=O), 145.4, 137.3, 132.8, 131.4, 128.6, 128.5, 128.1, 119.7, 58.1, 54.1, 42.2, 36.4, 33.3, 21.9; **HRMS** (ESI) m/z calcd for $C_{21}H_{25}BrNO$ $[M+H]^+$: 386.1114, found: 386.1109; **IR** (KBr, plate), ν (cm^{-1}): 1684 (C=O).

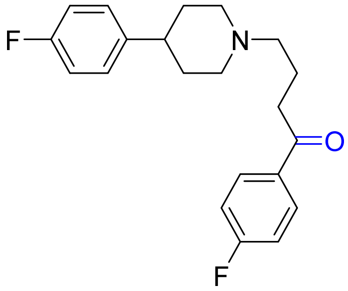

1-(4-Fluorophenyl)-4-(4-(4-fluorophenyl)piperidin-1-yl)butan-1-one (80a) was synthesized by the general Procedure **C** and purified by flash column chromatography on silica gel (gradient elution: Hexanes/EtOAc = 20:1-4:1) to give the desired product as white solid (73 mg, 85% yield); **^{1}H NMR** (400 MHz, $CDCl_3$) δ (ppm): 8.04-8.00 (m, 2H, ArH), 7.16-7.11 (m, 4H, ArH), 6.99-6.94 (m, 2H, ArH), 3.02-2.97 (m, 4H), 2.45-2.41 (m, 3H), 2.07-1.94 (m, 4H), 1.79-1.75 (m, Hz, 2H), 1.69-1.62 (m, 2H); **^{13}C NMR** (100 MHz, $CDCl_3$) δ (ppm): 198.4 (C=O), 165.6 (d, J_{C-F} = 252.0 Hz), 161.2 (d, J_{C-F} = 242.0 Hz), 142.0, 133.6, 130.6 (d, J_{C-F} = 9.0 Hz), 128.1 (d, J_{C-F} = 8.0 Hz), 115.5 (d, J_{C-F} = 22.0 Hz), 115.0 (d, J_{C-F} = 21.0 Hz), 58.0, 54.1, 41.9, 36.2, 33.5, 21.8; **HRMS** (ESI) m/z calcd for $C_{21}H_{24}F_2NO$ $[M+H]^+$: 344.1820, found: 344.1819; **IR** (KBr, plate), ν (cm^{-1}): 1684 (C=O).

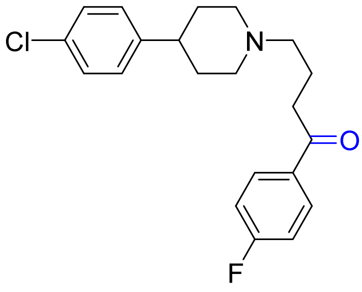

4-(4-(4-Chlorophenyl)piperidin-1-yl)-1-(4-fluorophenyl)butan-1-one (81a)[19] was synthesized by the general Procedure **C** and purified by flash column chromatography on silica gel (gradient elution: Hexanes/EtOAc = 20:1-4:1) to give the desired product as white solid (75 mg, 83% yield); **^{1}H NMR** (400 MHz, $CDCl_3$) δ (ppm): 8.06-8.02 (m, 2H, ArH), 7.28-7.26 (m, 2H, ArH), 7.18-7.13 (m, 4H, ArH), 3.05-2.99 (m, 4H), 2.50-

2.44 (m, 3H), 2.09-1.98 (m, 4H), 1.80-1.65 (m, 4H); **^{13}C NMR** (100 MHz, $CDCl_3$) δ (ppm): 198.4 (C=O), 165.6 (d, $J_{C\text{-}F}$ = 253.0 Hz), 144.9, 133.7, 131.7, 130.6 (d, $J_{C\text{-}F}$ = 9.0 Hz), 128.5, 128.2, 115.6 (d, $J_{C\text{-}F}$ = 21.0 Hz), 58.0, 54.1, 42.1, 36.3, 33.3, 21.9; **HRMS** (ESI) m/z calcd for $C_{21}H_{24}ClFNO$ $[M+H]^+$: 360.1525, found: 360.1524; **IR** (KBr, plate), ν (cm^{-1}): 1684 (C=O).

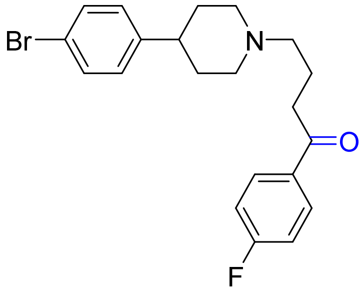

4-(4-(4-Bromophenyl)piperidin-1-yl)-1-(4-fluorophenyl)butan-1-one (82a) was synthesized by the general Procedure **C** and purified by flash column chromatography on silica gel (gradient elution: Hexanes/EtOAc = 20:1-4:1) to give the desired product as white solid (81 mg, 80% yield); **^{1}H NMR** (400 MHz, $CDCl_3$) δ (ppm): 8.03-8.00 (m, 2H, ArH), 7.40 (d, J = 8.4 Hz, 2H, ArH), 7.12 (t, J = 8.4 Hz, 2H, ArH), 7.05 (d, J = 8.4 Hz, 2H, ArH), 3.03-2.97 (m, 4H), 2.46-2.42 (m, 3H), 2.07-1.95 (m, 4H), 1.78-1.76 (m, 2H), 1.69-1.62 (m, 2H); **^{13}C NMR** (100 MHz, $CDCl_3$) δ (ppm): 198.3 (C=O), 165.6 (d, $J_{C\text{-}F}$ = 253.0 Hz), 145.2, 133.6 (d, $J_{C\text{-}F}$ = 3.0 Hz), 131.3, 130.6 (d, $J_{C\text{-}F}$ = 9.0 Hz), 128.5, 119.6, 115.6 (d, $J_{C\text{-}F}$ = 21.0 Hz), 57.9, 54.0, 42.0, 36.2, 33.1, 21.7; **HRMS** (ESI) m/z calcd for $C_{21}H_{24}BrFNO$ $[M+H]^+$: 404.1020, found: 404.1015; **IR** (KBr, plate), ν (cm^{-1}): 1684 (C=O).

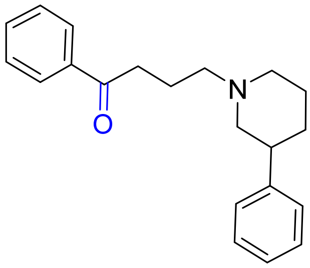

1-Phenyl-4-(3-phenylpiperidin-1-yl)butan-1-one (83a): was synthesized by the general Procedure **C** and purified by flash column chromatography on silica gel (gradient elution: Hexanes/EtOAc = 20:1-4:1) to give the desired product as white solid (63 mg, 82% yield); **^{1}H NMR** (400 MHz, $CDCl_3$) δ (ppm): 7.99-7.97 (m, 2H, ArH), 7.58-7.53 (m, 1H, ArH), 7.48-7.44 (m, 2H, ArH), 7.33-7.27 (m, 2H, ArH), 7.22-7.18

(m, 3H, ArH), 3.02-2.96 (m, 4H), 2.76-2.70 (m, 1H), 2.46-2.42 (m, 2H), 2.03-1.84 (m, 5H), 1.79-1.73 (m, 1H), 1.70-1.59 (m, 1H), 1.49-1.39 (m, 1H); **^{13}C NMR** (100 MHz, $CDCl_3$) δ (ppm): 200.1 (C=O), 144.8, 137.2, 132.8, 128.5, 128.3, 128.1, 127.2, 126.3, 61.2, 58.2, 53.7, 42.8, 36.4, 31.5, 25.7, 21.8; **HRMS** (ESI) m/z calcd for $C_{21}H_{26}NO$ $[M+H]^+$: 308.2009, found: 308.2011; **IR** (KBr, plate), ν (cm^{-1}): 1685 (C=O).

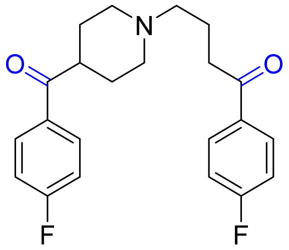

4-(4-(4-Fluorobenzoyl)piperidin-1-yl)-1-(4-fluorophenyl)butan-1-one (84a) was synthesized by the general Procedure **C** (***rac*-1** (4 mol%)) and purified by flash column chromatography on silica gel (gradient elution: Hexanes/EtOAc = 20:1-4:1) to give the desired product as white solid (83 mg, 89% yield); **^{1}H NMR** (400 MHz, $CDCl_3$) δ (ppm): 8.02-7.93 (m, 4H, ArH), 7.14-7.10 (m, 4H, ArH), 3.20-3.13 (m, 1H), 3.00-2.94 (m, 4H), 2.42 (t, J = 7.2 Hz, 2H), 2.09 (td, J =11.2, 3.2 Hz, 2H), 1.98-1.91 (m, 2H), 1.83-1.71 (m, 4H); **^{13}C NMR** (100 MHz, $CDCl_3$) δ (ppm): 201.1 (C=O), 198.6 (C=O), 165.7 (d, $J_{C\text{-}F}$ = 252.0 Hz), 165.6 (d, $J_{C\text{-}F}$ = 252.0 Hz), 133.7 (d, $J_{C\text{-}F}$ = 3.0 Hz), 132.5 (d, $J_{C\text{-}F}$ = 3.0 Hz), 130.8 (d, $J_{C\text{-}F}$ = 9.0 Hz), 130.7 (d, $J_{C\text{-}F}$ = 9.0 Hz), 115.8 (d, $J_{C\text{-}F}$ = 22.0 Hz), 115.7 (d, $J_{C\text{-}F}$ = 22.0 Hz), 57.7, 53.1, 43.8, 36.1, 28.7, 21.7; **HRMS** (ESI) m/z calcd for $C_{22}H_{24}F_2NO_2$ $[M+H]^+$: 372.1770, found: 372.1768; **IR** (KBr, plate), ν (cm^{-1}): 1682 (C=O).

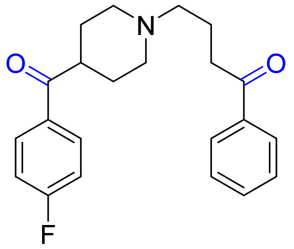

4-(4-(4-Fluorobenzoyl)piperidin-1-yl)-1-phenylbutan-1-one (85a)[19] was synthesized by the general Procedure **C** (***rac*-1** (4 mol%)) and purified by flash column chromatography on silica gel (gradient elution: Hexanes/EtOAc = 20:1-4:1) to give the desired product as white solid (75 mg, 85% yield); **^{1}H NMR** (400 MHz, $CDCl_3$) δ (ppm): 7.99-7.94 (m, 4H, ArH), 7.57-7.52 (m, 1H, ArH), 7.48-7.44 (m, 2H, ArH), 7.15-

7.11 (m, 2H, ArH), 3.21-3.14 (m, 1H), 3.05-2.96 (m, 4H), 2.45 (t, *J* = 7.2 Hz, 2H), 2.15-2.07 (m, 2H), 2.00-1.93 (m, 2H), 1.84-1.76 (m, 4H); **^{13}C NMR** (100 MHz, $CDCl_3$) δ (ppm): 201.0 (C=O), 200.1 (C=O), 165.6 (d, J_{C-F} = 253.0 Hz), 137.1, 132.8, 132.4 (d, J_{C-F} = 3.0 Hz), 130.8 (d, J_{C-F} = 9.0 Hz), 128.5, 128.0, 115.7 (d, J_{C-F} = 22.0 Hz), 57.8, 53.1, 43.7, 36.2, 28.6, 21.6; **HRMS** (ESI) m/z calcd for $C_{22}H_{25}FNO_2$ [M+H]$^+$: 354.1864, found: 354.1871; **IR** (KBr, plate), ν (cm^{-1}): 1683 (C=O).

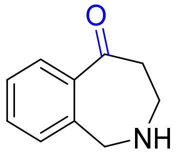

1,2,3,4-Tetrahydro-5*H*-benzo[*c*]azepin-5-one (86a) was synthesized by the general Procedure **B** (***rac*-1** (3 mol%)) and purified by flash column chromatography on silica gel (gradient elution: Hexanes/EtOAc = 20:1-4:1) to give the desired product as white solid (66 mg, 82% yield); **^{1}H NMR** (400 MHz, $CDCl_3$) δ (ppm): 7.80 (d, *J* = 7.6 Hz, 1H, ArH), 7.44 (t, *J* = 7.6 Hz, 1H, ArH), 7.35 (t, *J* =7.6 Hz, 1H, ArH), 7.24 (d, *J* = 7.6 Hz, 1H, ArH), 4.15 (s, 2H), 3.23 (t, *J* = 6.4 Hz, 2H), 2.95 (t, *J* = 6.4 Hz, 2H); **^{13}C NMR** (100 MHz, $CDCl_3$) δ (ppm): 203.5 (C=O), 142.8, 138.7, 132.0, 128.7, 128.5, 127.3, 51.3, 43.4, 43.3; **HRMS** (ESI) m/z calcd for $C_{10}H_{12}NO$ [M+H]$^+$: 162.0913, found: 162.0915; **IR** (KBr, plate), ν (cm^{-1}): 1663 (C=O).

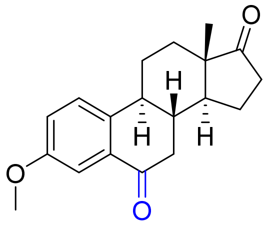

(*8R*,*9S*,*13S*,*14S*)-3-Methoxy-13-methyl-8,9,11,12,13,14,15,16-octahydro-6H-cyclopenta[a]phenanthrene-6,17(7H)-dione (87a) [51] was synthesized by the general Procedure **A** (***rac*-1** (4 mol%)) and purified by flash column chromatography on silica gel (gradient elution: Hexanes/EtOAc = 10:1-4:1) to give the desired product as white solid (131 mg, 88% yield); **^{1}H NMR** (400 MHz, $CDCl_3$) δ (ppm): 7.58 (d, *J* = 3.2 Hz, 1H, ArH), 7.36 (d, *J* = 8.8 Hz, 1H, ArH), 7.12 (dd, *J* = 8.8, 3.2 Hz, 1H, ArH), 3.85 (s, 3H), 2.88 (dd, *J* = 16.8, 3.2 Hz, 1H), 2.57-2.47 (m, 3H), 2.32 (dd, *J* = 16.8, 13.2 Hz, 1H), 2.22-2.02 (m, 4H), 1.69-1.56 (m, 4H), 0.93 (s, 3H); **^{13}C NMR** (100 MHz, $CDCl_3$) δ (ppm): 219.6, 197.3, 158.4, 138.9, 133.3, 126.6, 121.7, 109.8, 55.5, 50.3, 47.7, 43.3,

43.0, 39.6, 35.7, 31.2, 25.2, 21.4, 13.7; **HRMS** (ESI) m/z calcd for $C_{19}H_{22}O_3Na$ $[M+Na]^+$: 321.1461, found: 321.1468; **IR** (KBr, plate), ν (cm^{-1}): 1738, 1632 (C=O).

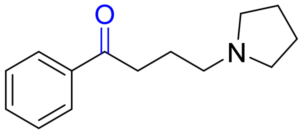

1-Phenyl-4-(pyrrolidin-1-yl)butan-1-one (88a) was synthesized according to the above procedure as white solid (87 mg, 80% yield); **^{1}H NMR** (400 MHz, $CDCl_3$) δ (ppm): 7.97 (d, *J* = 7.6 Hz, 2H, ArH), 7.53 (t, *J* = 7.2 Hz, 1H, ArH), 7.43 (t, *J* = 7.2 Hz, 2H, ArH), 3.02 (t, *J* = 7.2 Hz, 2H), 2.53-2.49 (m, 6H), 1.97-1.93 (m, 2H), 1.76-1.72 (m, 4H); **^{13}C NMR** (100 MHz, $CDCl_3$) δ (ppm): 200.0, 137.0, 132.8, 128.5, 128.0, 55.7, 54.0, 36.4, 23.5, 23.4; **HRMS** (ESI) m/z calcd for $C_{14}H_{20}NO$ $[M+H]^+$: 218.1539, found: 218.1541; **IR** (KBr, plate), ν (cm^{-1}): 1682 (C=O).

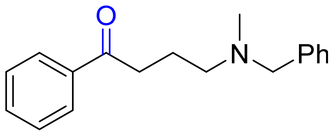

4-(Benzyl(methyl)amino)-1-phenylbutan-1-one (89a) was synthesized according to the above procedure as white solid (115 mg, 86% yield); **^{1}H NMR** (400 MHz, $CDCl_3$) δ (ppm): 7.97 (d, *J* = 7.6 Hz, 2H, ArH), 7.56 (t, *J* = 7.2 Hz, 1H, ArH), 7.46 (t, *J* = 7.2 Hz, 2H, ArH), 7.28-7.22 (m, 5H), 3.50 (s, 2H), 3.03 (t, *J* = 6.8 Hz, 2H), 2.47 (t, *J* = 6.8 Hz, 2H), 2.21 (s, 3H), 2.00-1.92 (m, 2H); **^{13}C NMR** (100 MHz, $CDCl_3$) δ (ppm): 200.2, 139.1, 137.1, 132.8, 128.9, 128.5, 128.1, 128.0, 126.9, 62.3, 56.5, 42.0, 36.2, 21.8; **HRMS** (ESI) m/z calcd for $C_{18}H_{22}NO$ $[M+H]^+$: 268.1696, found: 268.1702; **IR** (KBr, plate), ν (cm^{-1}): 1682 (C=O).

11. X-ray structures of manganese catalysts

CCDC number: **CCDC2046042**

In order to confirm the configuration of manganese catalyst, X-ray diffraction analysis of the manganese catalyst ***rac*-1** was carried out. The crystals were obtained from dried hexane/MeCN under N_2. The full crystallographic data for ***rac*-1** (CCDC 2046042) can be obtained free for charge from the Cambridge Crystallographic Data Center via https://www.ccdc.cam.ac.uk/structures/

Crystal Data for $C_{28}H_{38}F_6MnN_4O_6S_2$ (*M* =759.68 g/mol): orthorhombic, space group $P2_12_12_1$ (no. 19), *a* = 12.8207(3) Å, *b* = 14.4524(3) Å, *c* = 17.9758(4) Å, *V* = 3330.73(13) Å^3, *Z* = 4, *T* = 199.95 K, μ(CuKα) = 5.114 mm^{-1}, *Dcalc* = 1.515 g/cm^3, 54931 reflections measured (7.848° ≤ 2Θ ≤ 136.494°), 5853 unique (R_{int} = 0.0409, R_{sigma} = 0.0298) which were used in all calculations. The final R_1 was 0.0272 (I > 2σ(I)) and wR_2 was 0.0656 (all data).

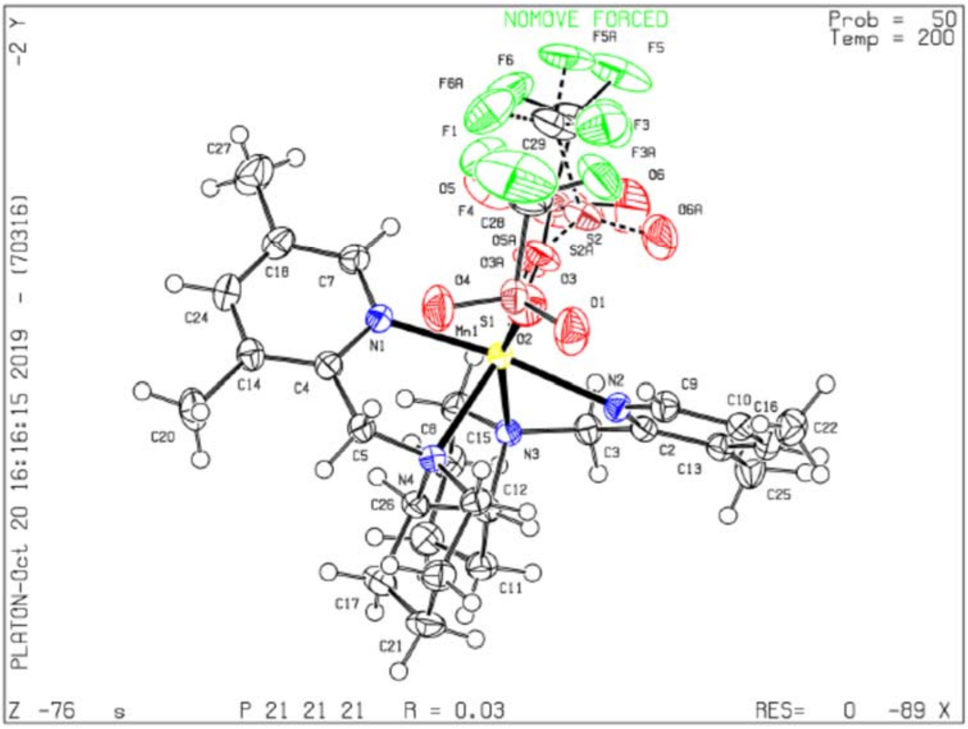

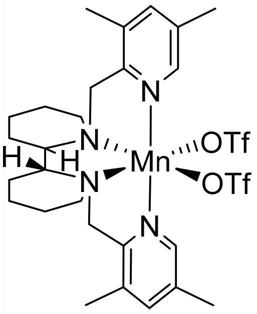

Table S-6 Crystal data and structure refinement for *rac*-1.	
Identification code	***rac*-1**
Empirical formula	$C_{28}H_{38}F_6MnN_4O_6S_2$
Formula weight	759.68
Temperature/K	199.95
Crystal system	orthorhombic
Space group	$P2_12_12_1$
a/Å	12.8207(3)
b/Å	14.4524(3)
c/Å	17.9758(4)
α/°	90
β/°	90
γ/°	90
Volume/Å^3	3330.73(13)

Z	4
ρ_{calc}g/cm^3	1.515
μ/mm^{-1}	5.114
F(000)	1572.0
Crystal size/mm^3	0.5 × 0.4 × 0.3
Radiation	CuKα (λ = 1.54178)
2Θ range for data collection/°	7.848 to 136.494
Index ranges	$-15 \le h \le 15$, $-17 \le k \le 17$, $-21 \le l \le 21$
Reflections collected	54931
Independent reflections	5853 [R_{int} = 0.0409, R_{sigma} = 0.0298]
Data/restraints/parameters	5853/201/501
Goodness-of-fit on F^2	1.090
Final R indexes [I>=2σ (I)]	R_1 = 0.0272, wR_2 = 0.0644
Final R indexes [all data]	R_1 = 0.0286, wR_2 = 0.0656
Largest diff. peak/hole / e Å^{-3}	0.19/-0.27
Flack parameter	0.045(2)

Table S-7 Fractional Atomic Coordinates (×10^4) and Equivalent Isotropic Displacement Parameters (Å^2×10^3) for s. U_{eq} is defined as 1/3 of of the trace of the orthogonalised U_{IJ} tensor.

Atom	*x*	*y*	*z*	U(eq)
Mn1	5243.8(3)	5197.9(3)	3877.1(2)	22.90(10)
S1	6010.6(5)	7505.1(5)	3902.6(4)	33.73(15)
S2	7526.8(17)	3799(2)	3975(2)	37.0(6)
O1	5725(2)	8023.1(19)	4544.3(13)	59.3(7)
N1	5334.5(16)	5152.5(15)	2634.7(11)	27.6(4)
O2	5963(2)	6514.4(15)	4021.2(13)	49.9(6)
F1	7800(2)	7160(2)	3268.5(14)	80.9(8)
N2	4953.0(16)	5185.5(15)	5116.8(11)	25.5(4)
O3	6745(9)	4537(9)	4016(10)	42(3)
N3	4291.4(15)	3884.0(14)	4116.7(11)	22.9(4)
F2	7909.6(19)	7460(2)	4427.8(14)	79.1(7)
O4	5588(2)	7799.1(18)	3211.7(13)	55.8(6)
F3	8688(12)	5320(9)	4285(8)	62(3)
N4	3631.6(16)	5661.2(15)	3485.8(11)	23.6(4)
C2	4781(2)	4343.1(17)	5406.5(13)	23.9(5)
C3	4609(2)	3558.2(17)	4863.5(14)	27.0(5)
C4	4524.5(19)	5531.5(17)	2270.0(14)	24.9(5)
C5	3783(2)	6088.9(18)	2743.7(14)	27.1(5)
C6	2976.0(19)	4803.3(18)	3425.3(13)	25.5(5)
C7	6066(2)	4701.1(19)	2244.5(14)	32.0(6)
C8	4483(2)	3153.0(17)	3544.6(14)	27.2(5)
C9	5099(2)	5905.9(18)	5581.2(14)	28.6(5)
C10	5091(2)	5825.0(19)	6346.4(14)	28.6(6)

C11	2423(2)	3361.0(18)	4178.0(15)	30.3(5)
C12	3165.3(19)	4187.4(17)	4114.4(14)	24.3(5)
C13	4797(2)	4184.9(17)	6175.5(14)	27.6(5)
C14	4408(2)	5442.2(17)	1501.8(14)	29.1(5)
C15	3744(2)	2324.2(19)	3603.5(16)	33.7(6)
C16	4959(2)	4940.4(19)	6631.9(14)	30.2(6)
C17	1828(2)	5040(2)	3271.6(16)	34.7(6)
C18	6033(2)	4599.9(19)	1481.6(15)	33.9(6)
C19	3174(2)	6346.9(18)	4013.2(15)	28.6(6)
C20	3480(3)	5838(2)	1092.8(18)	47.6(8)
F4	7602(2)	8551.8(19)	3646(3)	126.3(16)
C21	1378(2)	5731(2)	3826.9(19)	40.0(6)
C22	5223(3)	6654(2)	6843.4(16)	39.5(6)
C23	2048(2)	6598(2)	3844.9(18)	35.5(6)
C24	5182(2)	4968.7(18)	1118.1(15)	34.7(6)
C25	4650(3)	3229(2)	6497.2(16)	41.6(7)
C26	2616(2)	2636.9(19)	3575.9(16)	35.3(6)
F5	9478(4)	4004(4)	4307(6)	89(2)
C27	6884(3)	4089(3)	1072(2)	54.7(9)
C28	7404(3)	7678(3)	3811(2)	57.0(9)
C29	8715(7)	4498(8)	3998(7)	49(3)
F6	8818(6)	4543(7)	3298(4)	99(3)
O5	7527(4)	3219(3)	3342(4)	58.7(17)
O6	7745(5)	3371(5)	4674(4)	86(3)
S2A	7450(2)	3907(2)	4300(3)	35.0(7)
C29A	8556(10)	4550(10)	3819(8)	51(4)
F3A	8738(16)	5174(12)	4362(10)	68(4)
F6A	8498(7)	5027(5)	3182(4)	70(2)
F5A	9507(4)	4095(5)	3773(5)	70(2)
O5A	7524(5)	3003(4)	4011(7)	81(3)
O6A	7414(5)	3965(6)	5089(3)	63(2)
O3A	6622(11)	4382(11)	3916(11)	36(3)

Table S-8 Anisotropic Displacement Parameters (Å^2×10^3) for s. The Anisotropic displacement factor exponent takes the form: $-2\pi^2[h^2a^{*2}U_{11}+2hka^*b^*U_{12}+\ldots]$.

Atom	U_{11}	U_{22}	U_{33}	U_{23}	U_{13}	U_{12}
Mn1	20.82(18)	27.67(18)	20.23(17)	2.35(16)	- 1.01(15)	0.43(15)
S1	39.8(3)	35.1(3)	26.2(3)	2.9(3)	-4.4(3)	-4.5(3)
S2	23.3(7)	37.4(11)	50.3(16)	17.5(11)	-2.3(10)	3.7(6)
O1	73.2(17)	65.3(16)	39.4(12)	-12.5(11)	-6.8(12)	21.6(14)
N1	25.9(10)	34.3(11)	22.6(10)	2.0(8)	1.4(8)	2.9(10)
O2	57.3(13)	38.3(11)	54.0(14)	7.6(10)	-1.4(11)	-18.8(10)
F1	61.0(15)	120(2)	62.0(14)	8.3(15)	20.1(12)	7.9(14)
N2	27.9(11)	26.7(10)	21.8(9)	1.5(8)	0.7(8)	-2.4(8)

O3	23(3)	50(5)	52(5)	-5(4)	-11(3)	7(3)
N3	22.5(10)	24.3(10)	21.8(10)	-1.2(8)	-1.5(8)	1.5(8)
F2	55.7(13)	107(2)	74.6(15)	-11.7(15)	-28.0(12)	-0.2(14)
O4	64.9(16)	68.4(16)	33.9(11)	10.1(11)	-10.6(11)	7.7(13)
F3	60(5)	45(4)	81(6)	-5(3)	11(4)	-16(3)
N4	21.9(10)	26.4(10)	22.5(10)	-0.2(8)	0.4(8)	0.6(8)
C2	20.3(11)	27.4(11)	24.0(11)	1.6(9)	-1.7(10)	-1.4(10)
C3	30.1(13)	25.2(12)	25.7(12)	2.7(9)	-2.7(10)	0.7(10)
C4	25.5(13)	24.0(11)	25.4(12)	3.6(9)	-0.7(9)	-2.0(9)
C5	26.6(12)	29.7(12)	24.8(12)	5.5(10)	-0.8(10)	4.9(10)
C6	23.0(12)	27.9(11)	25.5(12)	-2.0(11)	-1.6(9)	-1.5(10)
C7	29.0(13)	37.6(14)	29.5(13)	1.0(12)	2.3(10)	6.7(12)
C8	29.2(14)	26.5(12)	25.8(12)	-5.5(10)	3.4(10)	2.8(10)
C9	31.3(14)	27.9(12)	26.6(12)	0.6(10)	-0.3(10)	-3.3(10)
C10	24.8(13)	35.2(13)	25.8(12)	-2.8(10)	-0.6(9)	-4.2(10)
C11	25.9(13)	32.5(13)	32.3(13)	1.5(11)	2.5(11)	-2.7(11)
C12	20.7(12)	28.4(12)	23.7(12)	-2.9(10)	1.2(9)	1.4(10)
C13	23.8(11)	32.8(13)	26.4(12)	4.5(10)	-1.8(11)	-4.0(10)
C14	33.8(14)	27.3(12)	26.3(12)	3.2(10)	-3.8(10)	-0.9(10)
C15	41.7(16)	27.8(13)	31.6(13)	-6.4(11)	2.6(11)	-1.9(12)
C16	29.6(14)	40.2(15)	20.8(11)	0.7(10)	0.0(9)	-6.6(10)
C17	24.8(13)	38.5(16)	40.8(15)	3.4(12)	-6.3(11)	-1.9(11)
C18	38.7(15)	32.6(14)	30.6(13)	-0.6(11)	7.3(12)	4.1(12)
C19	28.3(13)	28.8(12)	28.8(14)	-3.2(10)	2.8(10)	4.2(10)
C20	53.9(19)	59.1(19)	29.8(14)	-1.1(15)	-12.1(15)	15.5(15)
F4	84(2)	68.0(16)	227(5)	47(2)	0(2)	-42.0(16)
C21	21.6(12)	46.8(16)	51.5(17)	5.8(15)	2.8(12)	6.0(11)
C22	46.0(16)	40.7(15)	31.8(14)	-8.0(12)	1.1(14)	-10.1(14)
C23	32.2(14)	37.5(14)	36.9(14)	-2.6(13)	3.7(12)	9.8(11)
C24	45.9(15)	35.7(13)	22.5(11)	-1.1(11)	0.1(12)	0.7(11)
C25	55.8(19)	38.1(15)	30.9(14)	10.0(12)	-5.8(14)	-12.8(14)
C26	37.1(15)	31.5(13)	37.4(14)	-3.9(12)	-0.5(12)	-6.7(12)
F5	29(2)	85(3)	154(7)	-15(4)	-21(3)	9(2)
C27	59(2)	63(2)	42.2(17)	-2.2(17)	11.7(16)	24.2(17)
C28	46.7(19)	53.8(19)	70(2)	10.2(19)	-2.3(18)	-15.9(16)
C29	21(4)	53(4)	74(5)	-18(4)	-7(4)	-4(3)
F6	72(4)	158(7)	68(4)	-41(5)	37(4)	-27(5)
O5	45(3)	36(2)	95(4)	-14(3)	-17(3)	5.4(19)
O6	91(4)	82(5)	85(5)	60(5)	-10(4)	11(4)
S2A	24.4(9)	30.9(10)	49.8(19)	2.7(13)	-6.1(12)	1.6(7)
C29A	23(5)	56(6)	74(7)	-22(5)	-9(5)	2(4)
F3A	65(6)	69(8)	69(6)	-36(7)	-17(5)	-3(5)
F6A	82(5)	61(4)	67(4)	13(3)	30(3)	-12(3)
F5A	19(2)	85(4)	107(6)	-20(4)	6(3)	3(2)
O5A	57(4)	33(3)	151(10)	-13(4)	5(5)	1(3)

O6A	62(4)	82(5)	46(3)	30(3)	-13(3)	-8(4)
O3A	20(4)	47(5)	40(5)	-6(4)	-12(3)	7(4)

Table S-9 Bond Lengths for *rac*-1.

Atom	Atom	Length/Å	Atom	Atom	Length/Å
Mn1	N1	2.237(2)	C4	C14	1.395(4)
Mn1	O2	2.130(2)	C6	C12	1.544(3)
Mn1	N2	2.260(2)	C6	C17	1.537(3)
Mn1	O3	2.164(9)	C7	C18	1.380(4)
Mn1	N3	2.298(2)	C8	C15	1.530(4)
Mn1	N4	2.284(2)	C9	C10	1.381(4)
Mn1	O3A	2.126(10)	C10	C16	1.388(4)
S1	O1	1.423(2)	C10	C22	1.504(4)
S1	O2	1.449(2)	C11	C12	1.532(4)
S1	O4	1.420(2)	C11	C26	1.526(4)
S1	C28	1.811(4)	C13	C16	1.381(4)
S2	O3	1.465(9)	C13	C25	1.509(4)
S2	C29	1.829(9)	C14	C20	1.511(4)
S2	O5	1.412(6)	C14	C24	1.389(4)
S2	O6	1.429(6)	C15	C26	1.516(4)
N1	C4	1.345(3)	C17	C21	1.525(4)
N1	C7	1.340(3)	C18	C24	1.379(4)
F1	C28	1.330(5)	C18	C27	1.509(4)
N2	C2	1.342(3)	C19	C23	1.519(4)
N2	C9	1.348(3)	F4	C28	1.321(4)
N3	C3	1.480(3)	C21	C23	1.519(4)
N3	C8	1.495(3)	F5	C29	1.332(11)
N3	C12	1.509(3)	C29	F6	1.267(12)
F2	C28	1.323(5)	S2A	C29A	1.903(12)
F3	C29	1.296(13)	S2A	O5A	1.409(7)
N4	C5	1.483(3)	S2A	O6A	1.422(7)
N4	C6	1.502(3)	S2A	O3A	1.440(11)
N4	C19	1.491(3)	C29A	F3A	1.349(14)
C2	C3	1.513(3)	C29A	F6A	1.339(14)
C2	C13	1.401(3)	C29A	F5A	1.388(14)
C4	C5	1.509(3)			

Table S-10 Bond Angles for *rac*-1.

Atom	Atom	Atom	Angle/°	Atom	Atom	Atom	Angle/°
N1	Mn1	N2	173.14(8)	C14	C4	C5	122.7(2)
N1	Mn1	N3	100.98(8)	N4	C5	C4	111.6(2)
N1	Mn1	N4	75.42(8)	N4	C6	C12	109.25(19)
O2	Mn1	N1	97.18(9)	N4	C6	C17	111.4(2)
O2	Mn1	N2	87.63(9)	C17	C6	C12	115.0(2)
O2	Mn1	O3	89.7(4)	N1	C7	C18	123.4(2)
O2	Mn1	N3	160.94(8)	N3	C8	C15	113.8(2)
O2	Mn1	N4	99.65(9)	N2	C9	C10	123.4(2)
N2	Mn1	N3	73.79(7)	C9	C10	C16	116.6(2)
N2	Mn1	N4	99.02(7)	C9	C10	C22	121.6(2)
O3	Mn1	N1	93.2(5)	C16	C10	C22	121.9(2)
O3	Mn1	N2	91.7(5)	C26	C11	C12	112.4(2)

O3	Mn1	N3	94.9(4)	N3	C12	C6	108.67(19)
O3	Mn1	N4	166.0(4)	N3	C12	C11	111.6(2)
N4	Mn1	N3	79.58(7)	C11	C12	C6	114.3(2)
O3A	Mn1	N1	88.5(6)	C2	C13	C25	121.7(2)
O3A	Mn1	O2	97.6(5)	C16	C13	C2	117.3(2)
O3A	Mn1	N2	95.7(6)	C16	C13	C25	121.0(2)
O3A	Mn1	N3	88.7(5)	C4	C14	C20	122.1(3)
O3A	Mn1	N4	157.7(5)	C24	C14	C4	117.4(2)
O1	S1	O2	112.95(16)	C24	C14	C20	120.5(2)
O1	S1	C28	104.76(19)	C26	C15	C8	110.8(2)
O2	S1	C28	101.01(16)	C13	C16	C10	121.8(2)
O4	S1	O1	116.95(15)	C21	C17	C6	113.0(2)
O4	S1	O2	114.11(15)	C7	C18	C27	121.0(3)
O4	S1	C28	104.81(18)	C24	C18	C7	117.0(3)
O3	S2	C29	99.6(7)	C24	C18	C27	122.0(3)
O5	S2	O3	118.2(7)	N4	C19	C23	113.9(2)
O5	S2	C29	110.2(5)	C23	C21	C17	109.9(2)
O5	S2	O6	116.8(5)	C19	C23	C21	110.2(2)
O6	S2	O3	113.9(8)	C18	C24	C14	121.4(3)
O6	S2	C29	93.2(5)	C15	C26	C11	109.6(2)
C4	N1	Mn1	115.76(16)	F1	C28	S1	111.5(3)
C7	N1	Mn1	124.96(17)	F2	C28	S1	112.0(3)
C7	N1	C4	118.9(2)	F2	C28	F1	107.0(3)
S1	O2	Mn1	151.98(17)	F4	C28	S1	110.0(3)
C2	N2	Mn1	114.64(15)	F4	C28	F1	107.5(4)
C2	N2	C9	118.9(2)	F4	C28	F2	108.7(4)
C9	N2	Mn1	125.58(17)	F3	C29	S2	119.5(10)
S2	O3	Mn1	157.6(9)	F3	C29	F5	110.2(11)
C3	N3	Mn1	106.65(14)	F5	C29	S2	109.0(7)
C3	N3	C8	110.74(19)	F6	C29	S2	95.3(7)
C3	N3	C12	111.01(19)	F6	C29	F3	110.6(12)
C8	N3	Mn1	111.59(15)	F6	C29	F5	111.4(9)
C8	N3	C12	111.13(19)	O5A	S2A	C29A	103.6(6)
C12	N3	Mn1	105.53(14)	O5A	S2A	O6A	115.2(6)
C5	N4	Mn1	106.30(15)	O5A	S2A	O3A	108.4(8)
C5	N4	C6	110.63(19)	O6A	S2A	C29A	116.7(5)
C5	N4	C19	110.3(2)	O6A	S2A	O3A	115.2(9)
C6	N4	Mn1	106.66(14)	O3A	S2A	C29A	95.7(9)
C19	N4	Mn1	110.78(15)	F3A	C29A	S2A	97.3(11)
C19	N4	C6	112.00(19)	F3A	C29A	F5A	102.0(14)
N2	C2	C3	117.0(2)	F6A	C29A	S2A	126.8(9)
N2	C2	C13	121.9(2)	F6A	C29A	F3A	106.5(13)
C13	C2	C3	121.1(2)	F6A	C29A	F5A	104.0(10)
N3	C3	C2	112.8(2)	F5A	C29A	S2A	116.8(9)
N1	C4	C5	115.4(2)	S2A	O3A	Mn1	153.3(14)
N1	C4	C14	121.9(2)				

Table S-11 Hydrogen Atom Coordinates (Å×104) and Isotropic Displacement Parameters (Å2×103) for *rac*-1.

Atom	x	y	z	U(eq)

H3A	5262	3197	4818	32
H3B	4063	3141	5062	32
H5A	4062	6723	2806	32
H5B	3101	6136	2488	32
H6	3236	4451	2984	31
H7	6636	4437	2507	38
H8A	5210	2931	3594	33
H8B	4409	3432	3044	33
H9	5214	6500	5371	34
H11A	2511	3070	4673	36
H11B	1695	3584	4140	36
H12	3056	4582	4564	29
H15A	3881	1890	3189	40
H15B	3875	1993	4076	40
H16	4980	4852	7155	36
H17A	1767	5300	2764	42
H17B	1410	4464	3289	42
H19A	3601	6918	4001	34
H19B	3214	6092	4524	34
H20A	2839	5548	1277	71
H20B	3553	5717	559	71
H20C	3446	6508	1177	71
H21A	657	5895	3682	48
H21B	1356	5449	4328	48
H22A	5918	6641	7070	59
H22B	4691	6640	7235	59
H22C	5144	7220	6549	59
H23A	2009	6917	3358	43
H23B	1783	7026	4231	43
H24	5124	4898	594	42
H25A	3937	3017	6399	62
H25B	4770	3248	7035	62
H25C	5148	2802	6266	62
H26A	2150	2100	3655	42
H26B	2460	2904	3081	42
H27A	7362	4536	845	82
H27B	6573	3701	684	82
H27C	7269	3698	1423	82

Table S-12 Atomic Occupancy for *rac*-1.

Atom	Occupancy	Atom	Occupancy	Atom	Occupancy
S2	0.553(8)	O3	0.553(8)	F3	0.553(8)
F5	0.553(8)	C29	0.553(8)	F6	0.553(8)
O5	0.553(8)	O6	0.553(8)	S2A	0.447(8)
C29A	0.447(8)	F3A	0.447(8)	F6A	0.447(8)
F5A	0.447(8)	O5A	0.447(8)	O6A	0.447(8)
O3A	0.447(8)				

CCDC number: **CCDC2046041**

In order to confirm the configuration of manganese catalyst, X-ray diffraction analysis of the water-substituted ***rac*-1** was carried out. The crystals of **[*rac*-L^{1}Mn(H_2O)$_2$][OTf]$_2$** were obtained by dissolving ***rac*-1** in undried DCM layered with undried hexane with the solution open to air. The full crystallographic data for **[*rac*-L^{1}Mn(H_2O)$_2$][OTf]$_2$** (CCDC 2046041) can be obtained free for charge from the Cambridge Crystallographic Data Center via https://www.ccdc.cam.ac.uk/structures/

Crystal Data for $C_{28}H_{44}F_6MnN_4O_9S_2$ (M =813.73 g/mol): monoclinic, space group P2$_1$/c (no. 14), a = 14.919(4) Å, b = 13.061(3) Å, c = 18.685(5) Å, β = 98.016(11) °, V = 3605.4(17) Å^3, Z = 4, T = 153.0 K, μ(MoKα) = 0.567 mm^{-1}, $Dcalc$ = 1.499 g/cm^3, 60387 reflections measured (4.526° ≤ 2Θ ≤ 53.296°), 7440 unique (R_{int} = 0.0430, R_{sigma} = 0.0230) which were used in all calculations. The final R_1 was 0.0491 (I > 2σ(I)) and wR_2 was 0.1371 (all data).

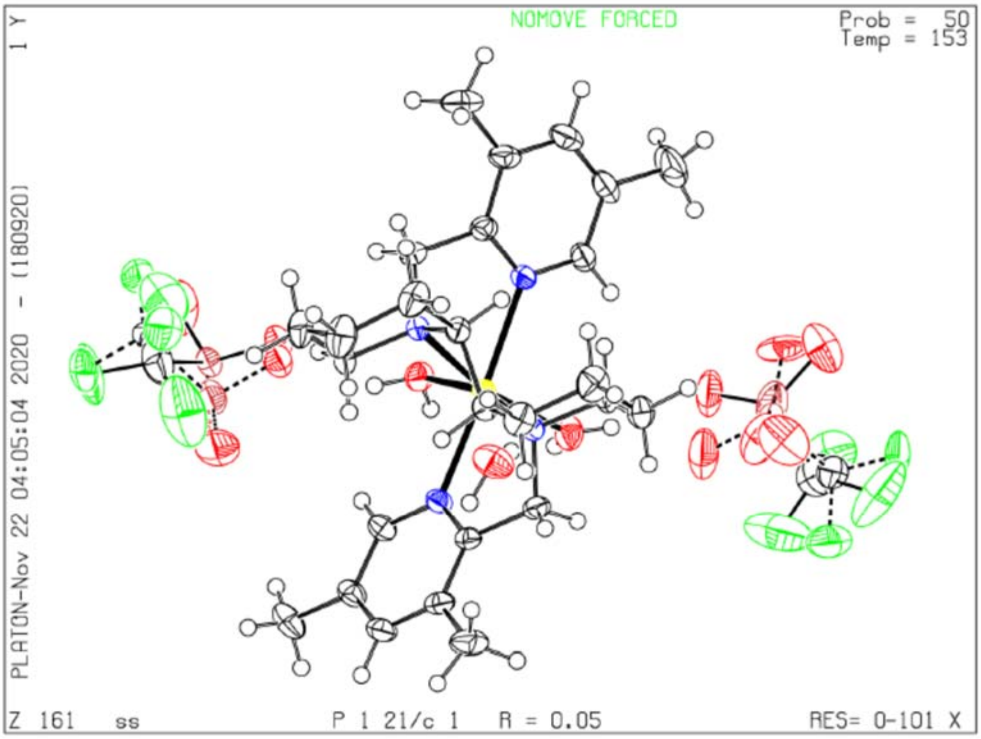

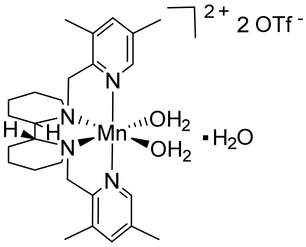

**Table S-13 Crystal data and structure refinement for [*rac*-L^{1}Mn(H_2O)$_2$][OTf]$_2$.**	
Identification code	**[*rac*-L^{1}Mn(H_2O)$_2$]$^{2+}$**
Empirical formula	[$MnC_{26}H_{42}N_4O_2$][OTf]$_2$.H_2O
Formula weight	813.73

Temperature/K	153.0
Crystal system	monoclinic
Space group	$P2_1/c$
a/Å	14.919(4)
b/Å	13.061(3)
c/Å	18.685(5)
α/°	90
β/°	98.016(11)
γ/°	90
Volume/Å^3	3605.4(17)
Z	4
ρ_{calc}g/cm^3	1.499
μ/mm^{-1}	0.567
F(000)	1692.0
Crystal size/mm^3	0.5 × 0.4 × 0.3
Radiation	MoKα (λ = 0.71073)
2Θ range for data collection/°	4.526 to 53.296
Index ranges	-18 ≤ h ≤ 18, -16 ≤ k ≤ 16, -23 ≤ l ≤ 23
Reflections collected	60387
Independent reflections	7440 [R_{int} = 0.0430, R_{sigma} = 0.0230]
Data/restraints/parameters	7440/750/609
Goodness-of-fit on F^2	1.037
Final R indexes [I>=2σ (I)]	R_1 = 0.0491, wR_2 = 0.1307
Final R indexes [all data]	R_1 = 0.0557, wR_2 = 0.1371
Largest diff. peak/hole / e Å^{-3}	1.70/-0.77

Table S-14 Fractional Atomic Coordinates (×104) and Equivalent Isotropic Displacement Parameters (Å2×103) for ss. Ueq is defined as 1/3 of of the trace of the orthogonalised UIJ tensor.

Atom	x	y	z	U(eq)
Mn01	7435.3(2)	2469.8(2)	5168.0(2)	19.50(11)
S002	10788.4(5)	1162.0(6)	6551.1(5)	26.5(3)
S003	3905(2)	1826(3)	4148.1(18)	59.1(9)
O004	8216.8(11)	1215.7(13)	5693.3(9)	29.7(4)
O005	6496.3(14)	1369.4(15)	4617.0(11)	35.9(4)
O006	7522.9(16)	-357.8(17)	4832.7(12)	45.8(5)
N007	6908.1(12)	3818.9(14)	4438.9(9)	20.9(4)
O008	9875(3)	1513(7)	6464(4)	35.7(11)
N009	8088.1(11)	3849.8(14)	5804.4(9)	20.8(4)
N00A	8266.9(13)	2413.7(14)	4250.3(10)	24.3(4)
N00B	6666.0(13)	2634.9(15)	6110.1(10)	24.2(4)
F00C	3322.2(18)	81.3(16)	3700.5(13)	78.2(7)
F00D	12298(4)	2145(6)	6703(5)	84(2)
C00E	7030.6(15)	3278.8(18)	6636.3(11)	25.1(4)
C00F	7989.0(15)	3051.3(17)	3697.8(11)	23.5(4)
C00G	7584.8(14)	4797.1(16)	5517.4(11)	23.2(4)

C00H	5921.0(14)	3959.1(18)	4467.1(12)	26.2(5)
C00I	9577.9(15)	1967.2(17)	3705.3(13)	27.9(5)
C00J	7062.1(16)	3520.2(19)	3701.8(11)	27.9(5)
F00K	11174(3)	3008(4)	7060(3)	102.0(17)
C00L	7445.4(14)	4756.5(17)	4684.3(12)	23.4(4)
C00M	9073.5(14)	3909.9(18)	5738.3(13)	26.8(5)
C00N	7963.7(15)	3674.6(19)	6568.8(11)	27.6(5)
C00O	9041.5(15)	1891.2(17)	4253.1(12)	26.1(5)
C00P	5424.2(16)	2295(2)	6764.4(14)	35.9(6)
C00Q	8503.2(16)	3222.3(18)	3141.2(12)	27.3(5)
O00R	11059(4)	642(5)	5943(3)	80(2)
C00S	9293.8(17)	2662.3(19)	3152.4(13)	29.6(5)
C00T	6594.3(17)	3509(2)	7231.3(12)	31.3(5)
C00U	5517.7(16)	4919(2)	4087.3(14)	34.4(5)
C00V	5787.4(17)	2998(2)	7282.2(13)	36.5(6)
C00W	7030.6(17)	5734.7(19)	4317.1(14)	33.7(5)
C00X	8047.5(18)	5776.7(19)	5833.8(15)	36.7(6)
C00Y	8228(2)	3993(2)	2552.4(13)	40.7(6)
C00Z	5885.1(15)	2161(2)	6172.8(13)	29.7(5)
C010	6980(2)	4276(2)	7800.3(14)	46.9(7)
C011	10435.0(18)	1353(2)	3732.9(17)	41.0(6)
C012	9536.5(17)	4886(2)	6049.3(16)	38.1(6)
C013	6035.0(18)	5857(2)	4395.2(15)	38.4(6)
O014	4119(5)	2720(6)	3726(4)	70(2)
O015	3138(3)	1971(3)	4504(3)	58.7(14)
C016	9036.9(19)	5823(2)	5711.1(18)	44.3(7)
C017	4572(2)	1704(3)	6824(2)	57.3(9)
F018	2808(6)	1265(5)	2990(4)	118(3)
O019	4658(2)	1377(4)	4528(2)	59.7(14)
O01A	11052(2)	730(4)	7240(2)	91.6(14)
F01B	11214(2)	2830(3)	5933(3)	108.5(15)
C01D	3522(7)	948(7)	3425(5)	65(2)
C01E	11413(3)	2349(4)	6566(3)	54.4(12)
F3	4148(6)	703(5)	3034(4)	118(2)
O0	3630(7)	1853(6)	4648(3)	64(2)
S0	3956(2)	1965(2)	3992.6(19)	27.0(7)
O1	3835(8)	2942(7)	3603(7)	70(3)
O2	4878(4)	1582(6)	3932(4)	78(2)
C1	3330(7)	1038(7)	3391(5)	44(2)
F0	2479(4)	1296(8)	3301(5)	85(2)
F3A	3542(5)	1116(6)	2716(3)	75.6(19)
O01B	9929(11)	1490(30)	6291(13)	38(4)
S1	10833.7(19)	1554(3)	6144(2)	39.2(11)
O3	10883(8)	2208(9)	5547(5)	59(3)
O00S	11267(12)	569(9)	6149(9)	43(3)
C01F	11509(7)	2224(8)	6884(5)	24(2)
F00E	11458(5)	1943(9)	7529(4)	71(3)
F00L	11255(9)	3158(9)	6757(6)	56(3)
F01C	12354(10)	2351(16)	6733(10)	42(3)

Table S-15 Anisotropic Displacement Parameters (Å2×103) for ss. The Anisotropic displacement factor exponent takes the form: -2π2[h2a*2U11+2hka*b*U12+…].						
**Atom**	**U11**	**U22**	**U33**	**U23**	**U13**	**U12**
Mn01	19.34(18)	20.61(19)	18.96(18)	-0.45(11)	4.11(12)	1.26(11)
S002	21.5(4)	26.8(4)	31.7(5)	5.3(3)	6.0(3)	1.9(3)
S003	42.3(10)	72.4(16)	58.6(16)	20.1(10)	-7.6(9)	-22.2(9)
O004	30.0(8)	29.2(8)	29.2(8)	2.2(7)	1.2(6)	6.5(7)
O005	29.0(10)	31.6(10)	46.1(11)	-11.7(8)	1.4(8)	-2.6(8)
O006	55.6(13)	32.3(11)	53.6(13)	-8.3(9)	21.8(11)	2.0(9)
N007	18.6(8)	23.6(9)	20.2(8)	-2.5(7)	2.3(6)	2.6(7)
O008	23.9(15)	40.4(19)	44(3)	2(2)	8.6(13)	0.1(13)
N009	18.0(8)	23.9(9)	20.4(8)	-0.3(7)	2.5(6)	1.1(7)
N00A	23.7(9)	26.4(9)	23.9(9)	-1.3(7)	7.4(7)	3.2(7)
N00B	21.5(9)	30.3(10)	21.6(9)	1.8(7)	5.7(7)	1.4(7)
F00C	104.3(17)	46.1(11)	83.4(15)	-7.6(10)	10.5(13)	-16.5(11)
F00D	25.8(17)	75(4)	150(4)	-11(3)	13(2)	-16.7(18)
C00E	27.0(11)	29.0(11)	19.3(10)	3.4(8)	3.1(8)	5.7(9)
C00F	26.3(11)	25.6(11)	18.8(9)	-4.6(8)	3.8(8)	1.1(8)
C00G	22.3(10)	20.9(10)	25.9(10)	-3.4(8)	1.7(8)	2.8(8)
C00H	17.5(10)	30.2(11)	30.5(11)	-2.2(9)	2.0(8)	3.6(8)
C00I	26.7(11)	24.9(11)	34.3(12)	-7.0(9)	11.4(9)	-1.3(9)
C00J	29.0(11)	36.0(12)	18.3(10)	-1.1(9)	1.5(8)	7.7(10)
F00K	77(2)	88(3)	147(4)	-76(3)	38(3)	-30(2)
C00L	23.1(10)	21.7(10)	25.4(10)	1.7(8)	3.7(8)	1.2(8)
C00M	17.2(10)	29.2(11)	33.7(12)	-0.3(9)	2.2(8)	0.5(8)
C00N	27.9(11)	36.2(12)	18.1(10)	-2.1(9)	1.5(8)	-0.7(9)
C00O	26.3(11)	23.6(11)	29.7(11)	-0.3(9)	8.4(9)	3.5(8)
C00P	22.5(11)	49.4(15)	37.8(13)	18.8(12)	11.0(10)	8.5(10)
C00Q	34.6(12)	29.0(11)	19.0(10)	-4.2(9)	5.7(9)	-3.2(9)
O00R	65(3)	93(4)	90(4)	-54(3)	38(3)	-13(2)
C00S	32.1(12)	32.4(12)	26.9(11)	-7.6(9)	13.0(9)	-5.4(10)
C00T	37.1(13)	37.6(13)	20.3(10)	5.1(9)	8.0(9)	13.6(10)
C00U	26.6(12)	39.0(13)	36.4(13)	1.9(11)	0.2(10)	10.7(10)
C00V	35.7(13)	48.7(15)	28.2(12)	11.9(11)	15.0(10)	16.5(11)
C00W	38.3(13)	24.5(11)	37.0(13)	6.7(10)	1.0(10)	1.4(10)
C00X	39.2(14)	23.0(11)	44.3(14)	-8.7(10)	-6.4(11)	3.0(10)
C00Y	54.3(16)	45.6(15)	24.0(11)	7.0(11)	12.0(11)	6.3(13)
C00Z	22.0(11)	37.2(13)	30.3(11)	6.7(10)	5.5(9)	1.0(9)
C010	65.0(19)	52.7(17)	25.5(12)	-6.6(12)	14.8(12)	8.3(15)
C011	31.5(13)	36.0(14)	59.2(17)	-3.3(12)	19.9(12)	5.0(11)
C012	24.9(12)	37.2(14)	50.0(15)	-3.2(12)	-2.2(10)	-6.6(10)
C013	39.5(14)	29.3(12)	44.7(14)	4.9(11)	-0.6(11)	13.0(11)
O014	71(4)	60(4)	71(3)	14(3)	-14(3)	-28(3)
O015	47(2)	27.2(18)	106(4)	-4(2)	27(2)	2.9(19)
C016	39.1(14)	28.2(13)	61.8(18)	-2.7(12)	-5.8(13)	-10.4(11)
C017	32.6(15)	80(2)	63(2)	21.8(18)	19.6(14)	-5.2(15)
F018	142(6)	99(4)	91(4)	23(3)	-66(4)	-39(4)
O019	27.1(18)	80(3)	67(3)	10(2)	-10.6(17)	-1.5(17)
O01A	63(2)	125(3)	83(2)	73(2)	-3.4(18)	6(2)

F01B	70(2)	89(2)	169(4)	78(3)	25(2)	-11.3(18)
C01D	67(4)	69(4)	60(4)	2(3)	10(3)	-23(3)
C01E	34(2)	49(2)	80(3)	-7(2)	11(2)	-4.8(17)
F3	160(5)	112(4)	100(4)	-42(3)	85(4)	-56(4)
O0	103(6)	68(4)	27(3)	-5(2)	28(3)	-41(4)
S0	26.8(9)	17.4(8)	34.8(10)	-3.1(7)	-2.5(6)	-1.7(6)
O1	85(6)	34(3)	97(6)	21(3)	33(5)	-7(3)
O2	34(3)	105(5)	88(5)	-10(4)	-11(3)	4(3)
C1	37(4)	55(4)	40(4)	-9(3)	5(3)	-12(3)
F0	26(2)	138(5)	86(5)	-27(4)	-11(2)	-6(3)
F3A	76(4)	107(5)	47(3)	-24(3)	18(3)	-20(4)
O01B	29(5)	44(6)	43(9)	4(6)	8(4)	-1(4)
S1	27.7(14)	41.9(18)	48(2)	-6.9(16)	6.6(12)	0.9(11)
O3	69(6)	70(6)	41(4)	22(4)	15(4)	-1(5)
O00S	54(7)	20(4)	56(6)	4(4)	9(5)	9(4)
C01F	22(4)	29(4)	22(3)	3(3)	5(3)	-1(3)
F00E	39(4)	147(7)	26(3)	18(4)	2(3)	-5(4)
F00L	54(3)	49(3)	65(3)	1.8(19)	2.6(19)	3.8(19)
F01C	26(5)	39(5)	62(6)	14(4)	6(4)	-13(3)

Table S-16 Bond Lengths for [*rac*-L¹Mn(H_2O)$_2$]$^{2+}$.

Atom	Atom	Length/Å	Atom	Atom	Length/Å
Mn01	O004	2.1650(16)	C00I	C00O	1.388(3)
Mn01	O005	2.1647(19)	C00I	C00S	1.396(4)
Mn01	N007	2.2979(18)	C00I	C011	1.504(3)
Mn01	N009	2.2996(18)	F00K	C01E	1.347(6)
Mn01	N00A	2.2536(19)	C00L	C00W	1.539(3)
Mn01	N00B	2.2418(19)	C00M	C012	1.526(3)
S002	O008	1.425(6)	C00P	C00V	1.388(4)
S002	O00R	1.429(5)	C00P	C00Z	1.392(3)
S002	O01A	1.410(3)	C00P	C017	1.505(4)
S002	C01E	1.807(5)	C00Q	C00S	1.386(3)
S003	O014	1.469(7)	C00Q	C00Y	1.506(3)
S003	O015	1.414(5)	C00T	C00V	1.392(4)
S003	O019	1.373(5)	C00T	C010	1.515(4)
S003	C01D	1.803(8)	C00U	C013	1.518(4)
N007	C00H	1.492(3)	C00W	C013	1.521(4)
N007	C00J	1.480(3)	C00X	C016	1.526(4)
N007	C00L	1.500(3)	C012	C016	1.524(4)
N009	C00G	1.507(3)	F018	C01D	1.315(10)
N009	C00M	1.495(3)	F01B	C01E	1.335(7)
N009	C00N	1.483(3)	C01D	F3	1.304(9)
N00A	C00F	1.346(3)	O0	S0	1.388(6)
N00A	C00O	1.341(3)	S0	O1	1.468(8)
N00B	C00E	1.349(3)	S0	O2	1.483(6)
N00B	C00Z	1.339(3)	S0	C1	1.819(8)
F00C	C01D	1.295(9)	C1	F0	1.302(11)
F00C	C1	1.378(10)	C1	F3A	1.345(10)

F00D	C01E	1.335(7)	O01B	S1	1.417(15)
C00E	C00N	1.507(3)	S1	O3	1.416(9)
C00E	C00T	1.397(3)	S1	O00S	1.439(11)
C00F	C00J	1.513(3)	S1	C01F	1.817(10)
C00F	C00Q	1.394(3)	C01F	F00E	1.272(12)
C00G	C00L	1.542(3)	C01F	F00L	1.289(13)
C00G	C00X	1.533(3)	C01F	F01C	1.340(16)
C00H	C00U	1.523(3)			

Table S-17 Bond Angles for [*rac*-L^{1}Mn(H_2O)$_2$]$^{2+}$.

Atom	Atom	Atom	Angle/°	Atom	Atom	Atom	Angle/°
O004	Mn01	N007	165.27(6)	N007	C00L	C00W	112.15(18)
O004	Mn01	N009	101.31(7)	C00W	C00L	C00G	114.25(18)
O004	Mn01	N00A	90.09(7)	N009	C00M	C012	114.25(19)
O004	Mn01	N00B	91.10(7)	N009	C00N	C00E	112.00(17)
O005	Mn01	O004	89.15(8)	N00A	C00O	C00I	123.1(2)
O005	Mn01	N007	94.70(8)	C00V	C00P	C00Z	116.7(2)
O005	Mn01	N009	164.94(7)	C00V	C00P	C017	122.7(3)
O005	Mn01	N00A	90.28(7)	C00Z	C00P	C017	120.7(3)
O005	Mn01	N00B	93.96(8)	C00F	C00Q	C00Y	122.0(2)
N007	Mn01	N009	77.92(7)	C00S	C00Q	C00F	117.4(2)
N00A	Mn01	N007	75.70(7)	C00S	C00Q	C00Y	120.6(2)
N00A	Mn01	N009	100.40(7)	C00Q	C00S	C00I	121.6(2)
N00B	Mn01	N007	102.79(7)	C00E	C00T	C010	121.8(2)
N00B	Mn01	N009	75.22(7)	C00V	C00T	C00E	117.0(2)
N00B	Mn01	N00A	175.61(7)	C00V	C00T	C010	121.2(2)
O008	S002	O00R	115.7(4)	C013	C00U	C00H	109.93(19)
O008	S002	C01E	102.1(4)	C00P	C00V	C00T	121.8(2)
O00R	S002	C01E	102.5(3)	C013	C00W	C00L	112.4(2)
O01A	S002	O008	111.9(3)	C016	C00X	C00G	111.6(2)
O01A	S002	O00R	117.5(3)	N00B	C00Z	C00P	123.0(2)
O01A	S002	C01E	104.6(3)	C016	C012	C00M	110.1(2)
O014	S003	C01D	100.0(5)	C00U	C013	C00W	109.3(2)
O015	S003	O014	113.4(4)	C012	C016	C00X	109.4(2)
O015	S003	C01D	104.2(4)	F00C	C01D	S003	109.0(6)
O019	S003	O014	113.1(4)	F00C	C01D	F018	108.0(7)
O019	S003	O015	118.2(4)	F00C	C01D	F3	103.4(7)
O019	S003	C01D	105.4(4)	F018	C01D	S003	114.1(7)
C00H	N007	Mn01	109.33(13)	F3	C01D	S003	113.5(6)
C00H	N007	C00L	112.14(16)	F3	C01D	F018	108.2(8)
C00J	N007	Mn01	105.36(13)	F00D	C01E	S002	109.0(4)
C00J	N007	C00H	110.32(16)	F00D	C01E	F00K	110.2(6)
C00J	N007	C00L	110.74(17)	F00K	C01E	S002	111.9(4)
C00L	N007	Mn01	108.70(12)	F01B	C01E	S002	109.8(4)
C00G	N009	Mn01	107.79(12)	F01B	C01E	F00D	110.9(5)
C00M	N009	Mn01	110.43(13)	F01B	C01E	F00K	105.0(5)
C00M	N009	C00G	112.00(17)	O0	S0	O1	119.7(6)
C00N	N009	Mn01	106.03(13)	O0	S0	O2	118.4(6)
C00N	N009	C00G	110.26(16)	O0	S0	C1	105.2(4)

C00N	N009	C00M	110.13(16)	O1	S0	O2	107.8(6)
C00F	N00A	Mn01	114.83(14)	O1	S0	C1	104.8(6)
C00O	N00A	Mn01	125.38(15)	O2	S0	C1	97.5(4)
C00O	N00A	C00F	119.40(19)	F00C	C1	S0	112.2(6)
C00E	N00B	Mn01	115.69(15)	F0	C1	F00C	102.8(8)
C00Z	N00B	Mn01	124.85(16)	F0	C1	S0	108.4(7)
C00Z	N00B	C00E	119.4(2)	F0	C1	F3A	102.3(8)
N00B	C00E	C00N	115.86(19)	F3A	C1	F00C	118.6(8)
N00B	C00E	C00T	121.9(2)	F3A	C1	S0	111.2(6)
C00T	C00E	C00N	122.1(2)	O01B	S1	O00S	112.4(15)
N00A	C00F	C00J	115.51(19)	O01B	S1	C01F	109.0(11)
N00A	C00F	C00Q	121.9(2)	O3	S1	O01B	110.2(12)
C00Q	C00F	C00J	122.5(2)	O3	S1	O00S	118.1(9)
N009	C00G	C00L	108.74(16)	O3	S1	C01F	103.0(7)
N009	C00G	C00X	111.90(17)	O00S	S1	C01F	103.0(7)
C00X	C00G	C00L	114.08(19)	F00E	C01F	S1	118.8(8)
N007	C00H	C00U	114.35(19)	F00E	C01F	F00L	113.1(11)
C00O	C00I	C00S	116.4(2)	F00E	C01F	F01C	114.7(11)
C00O	C00I	C011	120.8(2)	F00L	C01F	S1	101.4(8)
C00S	C00I	C011	122.8(2)	F00L	C01F	F01C	96.0(12)
N007	C00J	C00F	111.83(17)	F01C	C01F	S1	109.7(11)
N007	C00L	C00G	109.13(17)				

Table S-18 Torsion Angles for [*rac*-L¹Mn(H_2O)$_2$]$^{2+}$.

A	B	C	D	Angle/°	A	B	C	D	Angle/°
Mn01	N007	C00H	C00U	172.11(15)	C00N	N009	C00G	C00X	72.5(2)
Mn01	N007	C00J	C00F	-44.1(2)	C00N	N009	C00M	C012	-71.7(2)
Mn01	N007	C00L	C00G	-42.11(18)	C00N	C00E	C00T	C00V	-171.9(2)
Mn01	N007	C00L	C00W	-169.74(15)	C00N	C00E	C00T	C010	7.7(4)
Mn01	N009	C00G	C00L	-45.25(18)	C00O	N00A	C00F	C00J	173.8(2)
Mn01	N009	C00G	C00X	-172.18(16)	C00O	N00A	C00F	C00Q	-2.9(3)
Mn01	N009	C00M	C012	171.50(16)	C00O	C00I	C00S	C00Q	-1.9(3)
Mn01	N009	C00N	C00E	-42.3(2)	C00Q	C00F	C00J	N007	-143.0(2)
Mn01	N00A	C00F	C00J	-13.0(2)	O00R	S002	C01E	F00D	-64.7(6)
Mn01	N00A	C00F	C00Q	170.28(17)	O00R	S002	C01E	F00K	173.1(5)
Mn01	N00A	C00O	C00I	-172.97(17)	O00R	S002	C01E	F01B	57.0(4)
Mn01	N00B	C00E	C00N	-8.5(2)	C00S	C00I	C00O	N00A	2.9(3)
Mn01	N00B	C00E	C00T	175.61(17)	C00T	C00E	C00N	N009	-148.3(2)
Mn01	N00B	C00Z	C00P	-179.24(18)	C00V	C00P	C00Z	N00B	3.5(4)
N007	C00H	C00U	C013	-55.9(3)	C00X	C00G	C00L	N007	-174.42(17)
N007	C00L	C00W	C013	52.9(3)	C00X	C00G	C00L	C00W	-48.0(3)
O008	S002	C01E	F00D	175.1(6)	C00Y	C00Q	C00S	C00I	177.9(2)
O008	S002	C01E	F00K	53.0(6)	C00Z	N00B	C00E	C00N	172.6(2)
O008	S002	C01E	F01B	-63.2(4)	C00Z	N00B	C00E	C00T	-3.3(3)
N009	C00G	C00L	N007	59.9(2)	C00Z	C00P	C00V	C00T	-2.9(4)
N009	C00G	C00L	C00W	-173.65(18)	C010	C00T	C00V	C00P	179.8(2)
N009	C00G	C00X	C016	54.9(3)	C011	C00I	C00O	N00A	-179.3(2)
N009	C00M	C012	C016	-54.7(3)	C011	C00I	C00S	C00Q	-179.7(2)

N00A	C00F	C00J	N007	40.2(3)	O014	S003	C01D	F00C	178.2(6)
N00A	C00F	C00Q	C00S	3.8(3)	O014	S003	C01D	F018	-61.1(8)
N00A	C00F	C00Q	C00Y	-175.4(2)	O014	S003	C01D	F3	63.5(8)
N00B	C00E	C00N	N009	35.8(3)	O015	S003	C01D	F00C	-64.4(7)
N00B	C00E	C00T	C00V	3.7(3)	O015	S003	C01D	F018	56.3(8)
N00B	C00E	C00T	C010	-176.6(2)	O015	S003	C01D	F3	-179.1(7)
C00E	N00B	C00Z	C00P	-0.4(3)	C017	C00P	C00V	C00T	177.6(3)
C00E	C00T	C00V	C00P	-0.5(4)	C017	C00P	C00Z	N00B	-177.0(2)
C00F	N00A	C00O	C00I	-0.5(3)	O019	S003	C01D	F00C	60.6(7)
C00F	C00Q	C00S	C00I	-1.2(3)	O019	S003	C01D	F018	-178.6(7)
C00G	N009	C00M	C012	51.4(2)	O019	S003	C01D	F3	-54.0(8)
C00G	N009	C00N	C00E	74.1(2)	O01A	S002	C01E	F00D	58.4(6)
C00G	C00L	C00W	C013	-71.9(3)	O01A	S002	C01E	F00K	-63.8(5)
C00G	C00X	C016	C012	-57.9(3)	O01A	S002	C01E	F01B	-179.9(4)
C00H	N007	C00J	C00F	-161.95(19)	O0	S0	C1	F00C	-48.6(8)
C00H	N007	C00L	C00G	78.9(2)	O0	S0	C1	F0	64.2(9)
C00H	N007	C00L	C00W	-48.7(2)	O0	S0	C1	F3A	175.9(8)
C00H	C00U	C013	C00W	57.1(3)	O1	S0	C1	F00C	-175.7(7)
C00J	N007	C00H	C00U	-72.5(2)	O1	S0	C1	F0	-62.9(9)
C00J	N007	C00L	C00G	-157.40(17)	O1	S0	C1	F3A	48.8(9)
C00J	N007	C00L	C00W	75.0(2)	O2	S0	C1	F00C	73.5(7)
C00J	C00F	C00Q	C00S	-172.8(2)	O2	S0	C1	F0	-173.6(8)
C00J	C00F	C00Q	C00Y	8.1(3)	O2	S0	C1	F3A	-61.9(8)
C00L	N007	C00H	C00U	51.5(2)	O01B	S1	C01F	F00E	49.2(17)
C00L	N007	C00J	C00F	73.3(2)	O01B	S1	C01F	F00L	-75.5(16)
C00L	C00G	C00X	C016	-69.1(3)	O01B	S1	C01F	F01C	-176.2(16)
C00L	C00W	C013	C00U	-56.9(3)	O3	S1	C01F	F00E	166.2(10)
C00M	N009	C00G	C00L	76.4(2)	O3	S1	C01F	F00L	41.6(10)
C00M	N009	C00G	C00X	-50.5(2)	O3	S1	C01F	F01C	-59.2(11)
C00M	N009	C00N	C00E	-161.82(19)	O00S	S1	C01F	F00E	-70.4(12)
C00M	C012	C016	C00X	56.7(3)	O00S	S1	C01F	F00L	164.9(11)
C00N	N009	C00G	C00L	-160.57(17)	O00S	S1	C01F	F01C	64.2(13)

Table S-19 Hydrogen Atom Coordinates (Å×104) and Isotropic Displacement Parameters (Å2×103) for [*rac*-L¹Mn(H_2O)₂]²⁺.

Atom	x	y	z	U(eq)
H00A	8793.63	1362.39	5737.41	45
H00B	8158.93	677.09	5413.01	45
H00G	6971.87	4770.08	5675.53	28
H00E	5821.55	3984.87	4979.7	31
H00F	5592.58	3354.68	4243.97	31
H00H	7002.47	4131.8	3385.81	34
H00I	6593.27	3020.82	3503.03	34
H00L	8057.33	4671.11	4531.61	28
H00J	9146.34	3860.82	5220.78	32
H00K	9384.04	3313.34	5988.61	32
H00M	8065.28	4325.3	6839.66	33
H00N	8420.01	3174.52	6788.01	33
H00O	9231.73	1448.41	4648.56	31

H00S	9651.44	2753.63	2774.55	36
H00P	5549.34	4868.94	3562.69	41
H00Q	4873.33	4981.75	4154.28	41
H00V	5476.52	3133.4	7682.42	44
H00R	7369.28	6336.97	4533.05	40
H00T	7095.55	5714.75	3797.21	40
H00U	7721.84	6379.66	5605.76	44
H00W	8014.33	5804.24	6358.94	44
H00X	7677.04	3756.92	2249.36	61
H00Y	8715.23	4069.44	2255.22	61
H	8110.77	4654.33	2769.3 61	
H00Z	5634.21	1713.75	5796.66	36
H01A	7585.83	4055.76	8016.65	70
H01B	6585.14	4319.67	8176.4	70
H01C	7020.13	4950.11	7575.94	70
H01D	10513.02	927.09	4168.83	61
H01E	10952.11	1818.49	3743.63	61
H01F	10398.3	914.52	3304.43	61
H01G	9541.82	4899.57	6579.64	46
H01H	10171.45	4897.75	5950.8	46
H01I	5966.85	5936.87	4911.7	46
H01J	5788.51	6476.94	4133.59	46
H01K	9074.01	5841.81	5186.39	53
H01L	9321.75	6453.8	5932.17	53
H01M	4680.98	1226	7230.9	86
H01N	4391.57	1320.61	6375.97	86
H01O	4087.9	2180.46	6903.09	86
H00C	6710(30)	800(30)	4618(19)	55(10)
H00D	5960(30)	1360(30)	4533(19)	54(11)
H00	7280(30)	-850(40)	4940(20)	76(14)
HA	7920(30)	-540(30)	4540(20)	72(12)

Table S-20 Atomic Occupancy for [*rac*-$L^1Mn(H_2O)_2]^{2+}$.

Atom	Occupancy	Atom	Occupancy	Atom	Occupancy
S002	0.771(3)	S003	0.583(6)	O008	0.771(3)
F00D	0.771(3)	F00K	0.771(3)	O00R	0.771(3)
O014	0.583(6)	O015	0.583(6)	F018	0.583(6)
O019	0.583(6)	O01A	0.771(3)	F01B	0.771(3)
C01D	0.583(6)	C01E	0.771(3)	F3	0.583(6)
O0	0.417(6)	S0	0.417(6)	O1	0.417(6)
O2	0.417(6)	C1	0.417(6)	F0	0.417(6)
F3A	0.417(6)	O01B	0.229(3)	S1	0.229(3)
O3	0.229(3)	O00S	0.229(3)	C01F	0.229(3)
F00E	0.229(3)	F00L	0.229(3)	F01C	0.229(3)

CCDC number: **CCDC2046040**

In order to confirm the configuration of manganese catalyst, X-ray diffraction analysis of the water-substituted ***meso*-1** was carried out. The crystals of **[*meso*-$L^1Mn(H_2O)_2$][OTf]$_2$** were obtained by dissolving ***meso*-1** in undried DCM layered with undried hexane with the solution open to air. The full crystallographic data for **[*meso*-$L^1Mn(H_2O)_2$][OTf]$_2$** (CCDC 2046040) can be obtained free for charge from the Cambridge Crystallographic Data Center via https://www.ccdc.cam.ac.uk/structures/

Crystal Data for $C_{28}H_{44}F_6MnN_4O_9S_2$ (M =813.73 g/mol): monoclinic, space group Cc (no. 9), a = 22.773(4) Å, b = 12.573(3) Å, c = 15.215(6) Å, β = 124.309(5) °, V = 3598.4(17) Å^3, Z = 4, T = 153.0 K, μ(MoKα) = 0.568 mm^{-1}, $Dcalc$ = 1.502 g/cm^3, 19673 reflections measured (6.284° ≤ 2Θ ≤ 52.98°), 6695 unique (R_{int} = 0.0547, R_{sigma} = 0.0620) which were used in all calculations. The final R_1 was 0.0860 (I > 2σ(I)) and wR_2 was 0.2478 (all data).

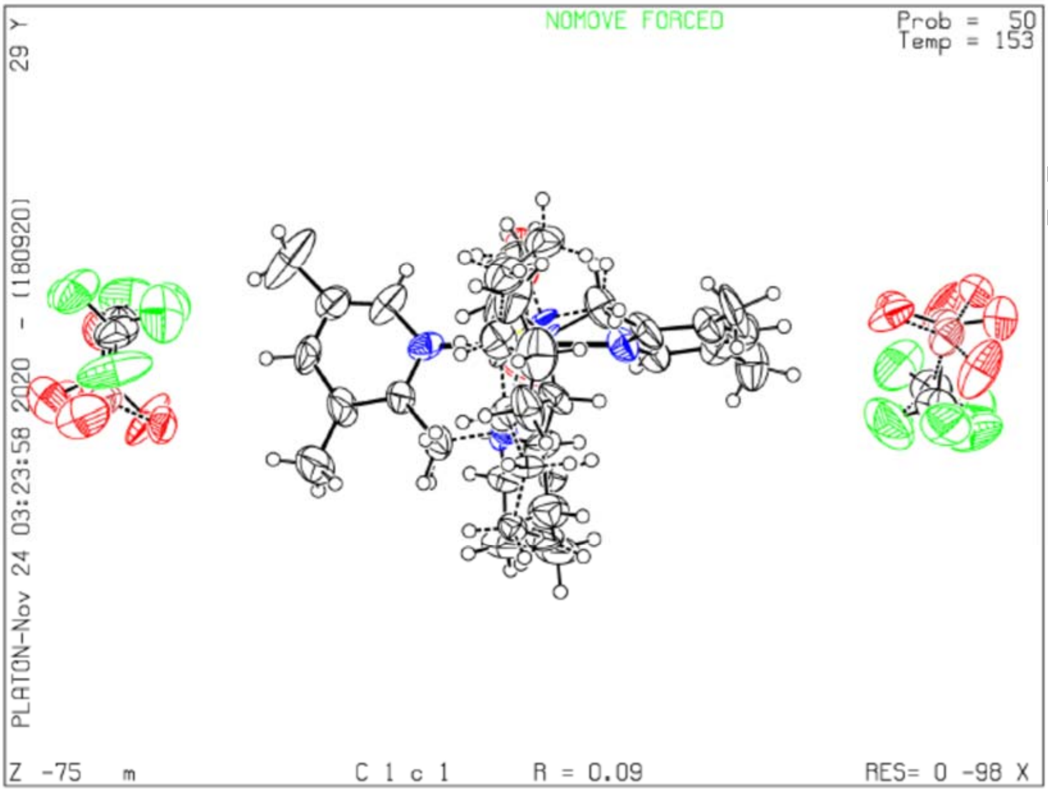

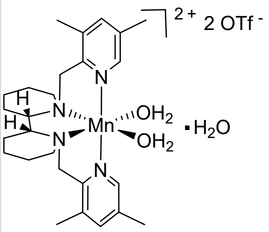

**Table S-21 Crystal data and structure refinement for [*meso*-$L^1Mn(H_2O)_2]^{2+}$.**	
Identification code	**[*meso*-$L^1Mn(H_2O)_2]^{2+}$**
Empirical formula	$[MnC_{26}H_{42}N_4O_2][OTf]_2.H_2O$
Formula weight	813.73
Temperature/K	153.0
Crystal system	monoclinic
Space group	Cc
a/Å	22.773(4)
b/Å	12.573(3)
c/Å	15.215(6)
α/°	90
β/°	124.309(5)
γ/°	90
Volume/Å^3	3598.4(17)
Z	4
ρ_{calc}g/cm^3	1.502
μ/mm^{-1}	0.568
F(000)	1692.0
Crystal size/mm^3	0.5 × 0.4 × 0.3
Radiation	MoKα (λ = 0.71073)
2Θ range for data collection/°	6.284 to 52.98
Index ranges	-28 ≤ h ≤ 28, -15 ≤ k ≤ 15, -18 ≤ l ≤ 19
Reflections collected	19673
Independent reflections	6695 [R_{int} = 0.0547, R_{sigma} = 0.0620]
Data/restraints/parameters	6695/1301/719
Goodness-of-fit on F^2	1.059
Final R indexes [I>=2σ (I)]	R_1 = 0.0860, wR_2 = 0.2292
Final R indexes [all data]	R_1 = 0.0988, wR_2 = 0.2478
Largest diff. peak/hole / e Å^{-3}	0.73/-0.73
Flack parameter	0.516(13)

Table S-22 Fractional Atomic Coordinates (×104) and Equivalent Isotropic Displacement Parameters (Å2×103) for m. Ueq is defined as 1/3 of of the trace of the orthogonalised U_{IJ} tensor.

**Atom**	**x**	**y**	**z**	**U(eq)**
Mn1	4989.3(11)	7622.5(7)	5011.7(17)	47.9(4)
S1	998(4)	8498(8)	-2253(6)	62(2)
S2	8925(3)	7840(7)	12328(5)	64(2)
N1	5644(6)	9087(9)	5185(9)	29(3)
N2	5865(5)	7677(8)	6744(9)	57(3)
O1	5563(5)	6392(8)	4802(9)	76(3)
N3	4259(6)	8963(10)	4826(9)	31(3)
F1	1566(19)	8000(40)	-3280(20)	135(11)

O2	1282(12)	8550(30)	-1130(15)	75(8)
O3	4446(5)	6375(8)	5283(10)	82(3)
C1	2992(7)	7648(11)	1241(10)	64(3)
O4	8717(11)	8160(20)	11279(14)	87(6)
C2	5870(6)	7008(10)	7434(10)	56(2)
C3	6508(8)	7018(10)	8517(11)	70(3)
O5	4989(10)	4582(6)	4978(12)	119(4)
N4	4120(5)	7686(7)	3274(8)	52(2)
C4	3596(5)	8373(11)	2973(10)	65(3)
C5	2391(8)	9097(16)	1677(15)	107(6)
F2	2341(13)	8600(30)	-1650(30)	119(10)
C6	2996(6)	8381(11)	1994(11)	73(4)
C7	6382(6)	8369(12)	7000(11)	71(4)
C8	4015(8)	7020(9)	2507(10)	68(3)
C9	3379(17)	6226(14)	673(16)	145(11)
C10	3538(8)	6992(11)	1534(11)	66(3)
O6	9561(9)	8187(14)	13210(13)	73(5)
C11	7009(7)	8450(12)	8071(11)	71(3)
O7	8792(15)	6746(13)	12337(15)	106(8)
C12	7054(10)	7733(11)	8792(12)	80(4)
F3	7642(10)	8290(20)	11680(20)	109(7)
O8	920(19)	9521(18)	-2690(20)	126(10)
F4	1766(17)	6942(14)	-2060(30)	132(8)
C13	7580(7)	9289(17)	8338(14)	117(7)
C14	4762(8)	9814(14)	5495(16)	76(5)
C15	4412(12)	10671(16)	5800(20)	80(5)
C16	6338(5)	8838(9)	6051(8)	70(3)
C17	3983(11)	10168(18)	6190(20)	87(5)
C18	5912(14)	11115(18)	4820(20)	103(6)
C19	3774(7)	8543(11)	5152(11)	51(3)
C20	6029(16)	10232(17)	4250(19)	93(6)
C21	6440(8)	6286(13)	9301(11)	85(4)
C22	3755(5)	9353(7)	3706(7)	57(2)
C23	5302(10)	11026(13)	4915(17)	81(5)
C24	5637(14)	9233(18)	4209(14)	90(6)
C25	1721(14)	7970(20)	-2280(20)	69(5)
C26	5380(6)	10017(10)	5497(12)	53(3)
F5	8454(12)	9637(14)	12543(18)	120(6)
C27	8304(13)	8688(19)	12420(20)	80(5)
O9	436(13)	7820(30)	-2910(30)	115(10)
C28	3409(11)	9446(16)	5350(20)	89(5)
F6	8307(19)	8360(30)	13260(20)	118(9)
S1A	1063(3)	7821(8)	-2307(4)	59(2)
O2A	1136(16)	8270(30)	-1370(18)	115(10)
O8A	447(12)	8280(20)	-3217(17)	101(8)
C25A	1747(13)	8500(30)	-2350(20)	83(5)
O9A	1211(12)	6758(14)	-2366(18)	94(6)
F2A	1665(14)	9616(14)	-2350(20)	145(8)
F1A	1731(15)	8310(20)	-3222(19)	103(7)
F4A	2367(13)	8140(30)	-1520(30)	154(11)

S2A	9003(4)	8524(8)	12304(6)	65(2)
O4A	9228(19)	9445(17)	12920(19)	115(9)
O6A	9469(14)	7640(20)	12730(20)	100(8)
O7A	8754(16)	8660(13)	11216(18)	73(6)
C27A	8260(14)	8090(30)	12330(20)	84(6)
F5A	8390(20)	7970(30)	13220(20)	106(10)
F6A	7977(15)	7230(30)	11760(20)	134(9)
F3A	7746(17)	8790(40)	11650(30)	169(13)
C24A	6013(16)	8480(30)	4430(20)	86(6)
C20A	6579(15)	9300(20)	4680(30)	71(6)
C18A	6150(20)	10230(30)	4010(30)	79(7)
C23A	5581(16)	10610(20)	4140(30)	69(6)
C26A	5234(9)	9716(13)	4354(14)	32(3)
C14A	4819(9)	10124(14)	4670(15)	58(5)
C15A	4762(13)	11144(16)	5140(20)	49(5)
C17A	4102(10)	11287(16)	5153(19)	42(4)
C28A	4069(19)	10460(20)	5880(30)	66(6)
C19A	4458(11)	9432(15)	5887(15)	42(4)
N1A	5714(9)	8868(15)	5054(15)	40(5)
N3A	4403(10)	9202(14)	4841(14)	36(5)

Table S-23 Anisotropic Displacement Parameters (Å2×103) for m. The Anisotropic displacement factor exponent takes the form: -2π2[h2a*2U11+2hka*b*U12+…].

Atom	U11	U22	U33	U23	U13	U12
Mn1	46.6(5)	43.6(5)	62.8(7)	1.2(9)	36.5(5)	0.6(8)
S1	71(4)	61(5)	62(4)	-3(3)	43(3)	9(3)
S2	67(4)	79(5)	56(3)	21(3)	41(3)	18(3)
N1	51(6)	26(5)	23(5)	-14(4)	29(4)	-13(4)
N2	47(5)	65(6)	55(6)	16(5)	25(5)	7(4)
O1	58(5)	71(6)	113(8)	-9(5)	58(5)	17(4)
N3	26(5)	44(6)	25(5)	4(4)	15(4)	-10(4)
F1	101(17)	250(40)	91(13)	-3(16)	78(13)	20(20)
O2	45(8)	140(20)	43(8)	-5(10)	25(7)	-9(11)
O3	66(5)	62(5)	131(9)	41(6)	63(6)	21(4)
C1	51(5)	90(8)	38(6)	-25(5)	17(5)	-28(5)
O4	68(9)	149(19)	56(8)	26(11)	43(7)	34(12)
C2	50(4)	58(5)	57(6)	14(4)	29(4)	0(4)
C3	103(8)	45(5)	60(7)	7(5)	45(6)	20(6)
O5	140(7)	72(4)	81(6)	28(7)	24(5)	-12(9)
N4	59(6)	41(4)	66(6)	-26(4)	41(5)	-15(4)
C4	39(5)	77(7)	54(6)	-33(6)	11(4)	-5(5)
C5	60(7)	106(10)	103(12)	-54(9)	15(7)	7(7)
F2	73(11)	170(20)	109(15)	10(15)	49(12)	-63(14)
C6	43(5)	71(7)	64(8)	-30(6)	5(5)	-9(5)
C7	41(5)	85(8)	71(7)	35(6)	22(5)	25(5)
C8	122(10)	44(5)	56(7)	-8(5)	60(7)	-14(6)
C9	300(30)	83(10)	86(12)	-22(9)	132(18)	-34(14)

C10	83(8)	63(7)	58(7)	1(5)	44(6)	7(6)
O6	80(9)	68(9)	61(9)	6(7)	34(7)	-17(7)
C11	62(6)	73(7)	58(7)	9(5)	23(5)	20(5)
O7	190(20)	60(8)	64(11)	-31(7)	68(13)	-10(10)
C12	99(9)	59(6)	53(7)	4(5)	26(6)	31(6)
F3	90(10)	129(16)	137(14)	38(12)	82(10)	20(9)
O8	210(30)	89(13)	123(19)	52(12)	120(20)	63(13)
F4	170(20)	53(8)	200(20)	-2(10)	118(19)	17(10)
C13	43(6)	129(13)	81(10)	2(9)	-24(6)	12(7)
C14	57(7)	84(9)	80(10)	-62(8)	35(7)	-20(7)
C15	74(10)	76(11)	91(12)	-17(9)	48(9)	-3(8)
C16	46(4)	71(6)	73(5)	21(5)	22(4)	-7(4)
C17	84(11)	100(13)	106(15)	12(10)	71(10)	29(9)
C18	121(15)	85(11)	86(14)	25(10)	47(12)	-7(12)
C19	59(7)	64(7)	54(7)	37(5)	46(6)	28(5)
C20	120(15)	101(12)	63(11)	-1(9)	54(10)	-61(11)
C21	81(7)	94(9)	59(7)	12(6)	26(6)	-24(6)
C22	63(5)	52(4)	43(4)	12(3)	21(4)	12(4)
C23	69(9)	46(7)	74(10)	4(7)	7(7)	6(7)
C24	130(13)	105(11)	52(7)	-24(8)	62(8)	-68(10)
C25	65(10)	76(12)	75(12)	-4(12)	45(10)	0(9)
C26	25(5)	41(6)	74(9)	-3(5)	16(5)	-2(4)
F5	155(15)	79(8)	130(15)	30(8)	83(12)	60(8)
C27	93(10)	86(11)	88(13)	42(10)	67(10)	34(9)
O9	62(12)	180(20)	108(19)	-88(18)	52(13)	-62(16)
C28	95(11)	90(12)	122(15)	17(10)	86(11)	24(8)
F6	162(17)	150(20)	131(12)	7(15)	135(13)	2(17)
S1A	66(4)	77(5)	44(3)	-1(3)	36(3)	-12(3)
O2A	150(20)	170(20)	75(12)	-62(14)	91(14)	-82(17)
O8A	92(11)	136(17)	87(11)	66(12)	59(9)	28(11)
C25A	83(10)	100(12)	99(13)	-22(13)	71(10)	-16(11)
O9A	92(12)	74(9)	94(14)	-21(9)	40(11)	-5(8)
F2A	240(20)	86(9)	270(20)	-26(11)	240(20)	-36(11)
F1A	116(16)	132(14)	102(11)	-33(10)	87(11)	-48(12)
F4A	76(10)	270(30)	117(13)	-20(20)	53(11)	10(15)
S2A	85(5)	67(5)	55(4)	9(3)	46(4)	-3(4)
O4A	200(20)	83(11)	78(12)	-8(9)	87(16)	-50(13)
O6A	79(13)	132(16)	65(12)	-3(11)	27(11)	52(12)
O7A	140(18)	39(7)	86(10)	9(7)	92(12)	16(9)
C27A	88(12)	99(16)	86(13)	32(12)	62(12)	14(10)
F5A	120(20)	130(20)	79(11)	11(11)	65(13)	-26(17)
F6A	121(17)	180(20)	123(18)	-55(15)	84(15)	-64(15)
F3A	127(17)	240(30)	130(20)	100(20)	68(19)	100(20)
C24A	88(11)	100(12)	108(11)	-5(10)	79(10)	-1(8)
C20A	76(10)	86(12)	75(12)	-4(9)	56(9)	-1(8)
C18A	86(11)	92(12)	81(13)	-2(9)	61(9)	-13(9)
C23A	69(10)	56(10)	75(12)	-7(8)	36(8)	-10(8)
C26A	41(7)	35(7)	21(7)	-5(5)	18(5)	-8(5)
C14A	88(9)	46(8)	45(9)	15(6)	40(7)	16(7)
C15A	52(8)	47(8)	67(10)	-5(7)	45(8)	5(7)

C17A	36(8)	37(8)	48(10)	-2(7)	21(7)	-1(6)
C28A	78(14)	91(16)	65(14)	29(11)	62(12)	32(12)
C19A	44(8)	44(8)	38(7)	-1(6)	23(6)	-7(6)
N1A	52(9)	46(10)	37(9)	-16(7)	34(7)	3(7)
N3A	26(8)	41(9)	42(9)	6(8)	19(7)	-2(7)

Table S-24 Bond Lengths for [*meso*-$L^1Mn(H_2O)_2]^{2+}$.

Atom	Atom	Length/Å	Atom	Atom	Length/Å
Mn1	N1	2.289(10)	F3	C27	1.37(3)
Mn1	N2	2.237(11)	F4	C25	1.33(3)
Mn1	O1	2.161(8)	C14	C15	1.555(17)
Mn1	N3	2.271(12)	C14	C26	1.428(17)
Mn1	O3	2.176(9)	C15	C17	1.542(19)
Mn1	N4	2.240(11)	C16	N1A	1.375(19)
Mn1	N1A	2.250(19)	C17	C28	1.513(19)
Mn1	N3A	2.325(17)	C18	C20	1.52(2)
S1	O2	1.447(18)	C18	C23	1.48(2)
S1	O8	1.41(2)	C19	C28	1.535(16)
S1	C25	1.80(2)	C20	C24	1.522(17)
S1	O9	1.38(2)	C22	N3A	1.52(2)
S2	O4	1.443(17)	C23	C26	1.498(17)
S2	O6	1.377(16)	F5	C27	1.23(3)
S2	O7	1.410(18)	C27	F6	1.34(3)
S2	C27	1.84(2)	S1A	O2A	1.455(17)
N1	C16	1.408(14)	S1A	O8A	1.42(2)
N1	C24	1.488(15)	S1A	C25A	1.81(2)
N1	C26	1.509(15)	S1A	O9A	1.393(19)
N2	C2	1.340(16)	C25A	F2A	1.42(3)
N2	C7	1.335(18)	C25A	F1A	1.33(2)
N3	C14	1.476(14)	C25A	F4A	1.33(4)
N3	C19	1.537(13)	S2A	O4A	1.39(2)
N3	C22	1.502(14)	S2A	O6A	1.418(18)
F1	C25	1.35(3)	S2A	O7A	1.426(19)
C1	C6	1.466(19)	S2A	C27A	1.80(2)
C1	C10	1.34(2)	C27A	F5A	1.22(3)
C2	C3	1.458(18)	C27A	F6A	1.31(4)
C3	C12	1.39(3)	C27A	F3A	1.35(3)
C3	C21	1.582(19)	C24A	C20A	1.52(2)
N4	C4	1.328(16)	C24A	N1A	1.53(2)
N4	C8	1.344(15)	C20A	C18A	1.50(2)
C4	C6	1.338(16)	C18A	C23A	1.50(2)
C4	C22	1.561(14)	C23A	C26A	1.51(2)
C5	C6	1.48(2)	C26A	C14A	1.38(2)
F2	C25	1.42(3)	C26A	N1A	1.468(19)
C7	C11	1.442(18)	C14A	C15A	1.508(18)
C7	C16	1.510(14)	C14A	N3A	1.610(18)
C8	C10	1.249(19)	C15A	C17A	1.527(17)
C9	C10	1.50(2)	C17A	C28A	1.553(19)

C11	C12	1.38(2)	C28A	C19A	1.559(19)
C11	C13	1.54(3)	C19A	N3A	1.552(18)

Table S-25 Bond Angles for [*meso*-$L^1Mn(H_2O)_2]^{2+}$.

Atom	Atom	Atom	Angle/°	Atom	Atom	Atom	Angle/°
N2	Mn1	N1	75.5(4)	C26	C14	C15	122.9(13)
N2	Mn1	N3	103.2(4)	C17	C15	C14	112.0(16)
N2	Mn1	N4	176.2(2)	N1	C16	C7	114.6(9)
N2	Mn1	N1A	78.0(6)	N1A	C16	C7	122.4(11)
N2	Mn1	N3A	100.4(5)	C28	C17	C15	110.6(18)
O1	Mn1	N1	100.6(4)	C23	C18	C20	118.4(19)
O1	Mn1	N2	89.1(4)	C28	C19	N3	112.2(12)
O1	Mn1	N3	166.9(4)	C18	C20	C24	109.9(17)
O1	Mn1	O3	88.1(2)	N3	C22	C4	105.9(9)
O1	Mn1	N4	93.3(4)	N3A	C22	C4	113.5(10)
O1	Mn1	N1A	90.4(6)	C18	C23	C26	108.6(15)
O1	Mn1	N3A	162.2(5)	N1	C24	C20	112.6(13)
N3	Mn1	N1	78.5(4)	F1	C25	S1	111(2)
O3	Mn1	N1	163.9(5)	F1	C25	F2	106(3)
O3	Mn1	N2	91.3(4)	F2	C25	S1	111(2)
O3	Mn1	N3	96.0(4)	F4	C25	S1	107(2)
O3	Mn1	N4	91.8(4)	F4	C25	F1	104(3)
O3	Mn1	N1A	169.3(6)	F4	C25	F2	118(2)
O3	Mn1	N3A	106.5(5)	C14	C26	N1	115.3(11)
N4	Mn1	N1	101.1(4)	C14	C26	C23	111.2(14)
N4	Mn1	N3	74.2(4)	C23	C26	N1	113.9(13)
N4	Mn1	N1A	98.9(5)	F3	C27	S2	105.3(17)
N4	Mn1	N3A	76.5(5)	F5	C27	S2	114.6(19)
N1A	Mn1	N3A	77.0(6)	F5	C27	F3	124(2)
O2	S1	C25	103.8(13)	F5	C27	F6	108(3)
O8	S1	O2	111.7(18)	F6	C27	S2	107.8(19)
O8	S1	C25	101.2(16)	F6	C27	F3	95(3)
O9	S1	O2	120.1(19)	C17	C28	C19	107.7(15)
O9	S1	O8	113(2)	O2A	S1A	C25A	103.0(14)
O9	S1	C25	104.0(17)	O8A	S1A	O2A	107.5(19)
O4	S2	C27	97.8(13)	O8A	S1A	C25A	99.9(14)
O6	S2	O4	119.9(13)	O9A	S1A	O2A	122.7(18)
O6	S2	O7	114.4(13)	O9A	S1A	O8A	117.8(15)
O6	S2	C27	100.0(13)	O9A	S1A	C25A	101.8(16)
O7	S2	O4	110.1(14)	F2A	C25A	S1A	110.6(17)
O7	S2	C27	112.7(14)	F1A	C25A	S1A	114.4(18)
C16	N1	Mn1	104.1(7)	F1A	C25A	F2A	104(3)
C16	N1	C24	109.6(14)	F4A	C25A	S1A	107(2)
C16	N1	C26	108.3(10)	F4A	C25A	F2A	114(3)
C24	N1	Mn1	110.1(9)	F4A	C25A	F1A	108(3)
C24	N1	C26	115.9(14)	O4A	S2A	O6A	116.7(18)
C26	N1	Mn1	108.1(7)	O4A	S2A	O7A	115.8(13)
C2	N2	Mn1	120.8(8)	O4A	S2A	C27A	103.3(18)

C7	N2	Mn1	114.7(8)	O6A	S2A	O7A	109.3(14)
C7	N2	C2	124.4(11)	O6A	S2A	C27A	102.9(17)
C14	N3	Mn1	102.8(9)	O7A	S2A	C27A	107.3(16)
C14	N3	C19	116.6(12)	F5A	C27A	S2A	114(2)
C14	N3	C22	110.2(12)	F5A	C27A	F6A	111(3)
C19	N3	Mn1	108.3(8)	F5A	C27A	F3A	120(3)
C22	N3	Mn1	114.9(7)	F6A	C27A	S2A	110(2)
C22	N3	C19	104.5(9)	F6A	C27A	F3A	98(3)
C10	C1	C6	122.0(12)	F3A	C27A	S2A	102(2)
N2	C2	C3	116.3(11)	C20A	C24A	N1A	103.6(19)
C2	C3	C21	112.8(13)	C18A	C20A	C24A	103(2)
C12	C3	C2	119.8(12)	C20A	C18A	C23A	116(2)
C12	C3	C21	126.7(13)	C18A	C23A	C26A	113(2)
C4	N4	Mn1	117.0(7)	C14A	C26A	C23A	110.0(17)
C4	N4	C8	114.9(11)	C14A	C26A	N1A	113.3(16)
C8	N4	Mn1	127.5(9)	N1A	C26A	C23A	115.4(17)
N4	C4	C6	122.9(11)	C26A	C14A	C15A	137.8(15)
N4	C4	C22	117.7(9)	C26A	C14A	N3A	111.7(14)
C6	C4	C22	118.1(11)	C15A	C14A	N3A	108.7(14)
C1	C6	C5	122.2(12)	C14A	C15A	C17A	117.1(17)
C4	C6	C1	114.7(12)	C15A	C17A	C28A	112.0(18)
C4	C6	C5	123.0(13)	C17A	C28A	C19A	107.8(15)
N2	C7	C11	121.6(12)	N3A	C19A	C28A	115.4(16)
N2	C7	C16	113.8(11)	C16	N1A	Mn1	107.3(11)
C11	C7	C16	122.9(12)	C16	N1A	C24A	97.7(15)
C10	C8	N4	131.0(14)	C16	N1A	C26A	134.8(17)
C1	C10	C9	112.5(16)	C24A	N1A	Mn1	109.7(14)
C8	C10	C1	113.6(13)	C26A	N1A	Mn1	104.5(11)
C8	C10	C9	132.5(16)	C26A	N1A	C24A	101.0(18)
C7	C11	C13	119.8(13)	C22	N3A	Mn1	111.2(10)
C12	C11	C7	115.9(14)	C22	N3A	C14A	90.5(11)
C12	C11	C13	124.4(14)	C22	N3A	C19A	127.4(15)
C11	C12	C3	121.9(14)	C14A	N3A	Mn1	106.2(11)
N3	C14	C15	112.1(13)	C19A	N3A	Mn1	109.6(11)
C26	C14	N3	120.9(12)	C19A	N3A	C14A	108.5(14)

Table S-26 Torsion Angles for [*meso*-$L^1Mn(H_2O)_2]^{2+}$.

A	B	C	D	Angle/°	A	B	C	D	Angle/°
Mn1	N1	C16	C7	-45.6(12)	C16	N1	C26	C23	110.9(12)
Mn1	N1	C24	C20	176.7(18)	C16	C7	C11	C12	-161.1(13)
Mn1	N1	C26	C14	-6.5(16)	C16	C7	C11	C13	18(2)
Mn1	N1	C26	C23	-136.9(10)	C18	C20	C24	N1	-36(3)
Mn1	N2	C2	C3	172.5(8)	C18	C23	C26	N1	-40(2)
Mn1	N2	C7	C11	-175.7(10)	C18	C23	C26	C14	-172.3(17)
Mn1	N2	C7	C16	-10.1(14)	C19	N3	C14	C15	-43(2)
Mn1	N3	C14	C15	-161.2(15)	C19	N3	C14	C26	159.2(15)
Mn1	N3	C14	C26	41(2)	C19	N3	C22	C4	-88.2(11)
Mn1	N3	C19	C28	164.2(12)	C20	C18	C23	C26	59(3)

Mn1	N3	C22	C4	30.3(10)	C21	C3	C12	C11	-169.8(14)
Mn1	N4	C4	C6	-169.7(12)	C22	N3	C14	C15	75.8(19)
Mn1	N4	C4	C22	23.8(16)	C22	N3	C14	C26	-82(2)
Mn1	N4	C8	C10	178.3(12)	C22	N3	C19	C28	-72.9(16)
N2	C2	C3	C12	2.9(19)	C22	C4	C6	C1	160.2(12)
N2	C2	C3	C21	174.7(12)	C22	C4	C6	C5	-18(2)
N2	C7	C11	C12	3(2)	C23	C18	C20	C24	-19(3)
N2	C7	C11	C13	-177.9(15)	C24	N1	C16	C7	-163.4(13)
N2	C7	C16	N1	40.1(16)	C24	N1	C26	C14	117.6(17)
N2	C7	C16	N1A	22.5(19)	C24	N1	C26	C23	-12.7(17)
N3	C14	C15	C17	47(3)	C26	N1	C16	C7	69.3(13)
N3	C14	C26	N1	-25(2)	C26	N1	C24	C20	54(3)
N3	C14	C26	C23	107(2)	C26	C14	C15	C17	-156(2)
N3	C19	C28	C17	-57(2)	O9	S1	C25	F1	61(3)
O2	S1	C25	F1	-172(3)	O9	S1	C25	F2	179(2)
O2	S1	C25	F2	-55(2)	O9	S1	C25	F4	-52(3)
O2	S1	C25	F4	75(3)	O2A	S1A	C25A	F2A	-55(3)
O4	S2	C27	F3	67(2)	O2A	S1A	C25A	F1A	-172(3)
O4	S2	C27	F5	-73(2)	O2A	S1A	C25A	F4A	69(3)
O4	S2	C27	F6	168(2)	O8A	S1A	C25A	F2A	56(2)
C2	N2	C7	C11	0(2)	O8A	S1A	C25A	F1A	-61(3)
C2	N2	C7	C16	166.1(12)	O8A	S1A	C25A	F4A	180(2)
C2	C3	C12	C11	1(2)	O9A	S1A	C25A	F2A	177(2)
N4	C4	C6	C1	-6(2)	O9A	S1A	C25A	F1A	60(3)
N4	C4	C6	C5	175.8(17)	O9A	S1A	C25A	F4A	-59(2)
N4	C4	C22	N3	-35.4(16)	O4A	S2A	C27A	F5A	-56(3)
N4	C4	C22	N3A	-19.8(18)	O4A	S2A	C27A	F6A	179(2)
N4	C8	C10	C1	-10(2)	O4A	S2A	C27A	F3A	75(3)
N4	C8	C10	C9	-175.4(17)	O6A	S2A	C27A	F5A	66(3)
C4	N4	C8	C10	7(2)	O6A	S2A	C27A	F6A	-59(3)
C4	C22	N3A	Mn1	6.1(14)	O6A	S2A	C27A	F3A	-163(3)
C4	C22	N3A	C14A	113.8(12)	O7A	S2A	C27A	F5A	-178(3)
C4	C22	N3A	C19A	-132.3(17)	O7A	S2A	C27A	F6A	56(3)
C6	C1	C10	C8	5(2)	O7A	S2A	C27A	F3A	-48(3)
C6	C1	C10	C9	172.8(14)	C24A	C20A	C18A	C23A	54(4)
C6	C4	C22	N3	157.5(13)	C20A	C24A	N1A	Mn1	-170.8(18)
C6	C4	C22	N3A	173.1(14)	C20A	C24A	N1A	C16	-59(2)
C7	N2	C2	C3	-3(2)	C20A	C24A	N1A	C26A	79(2)
C7	C11	C12	C3	-4(2)	C20A	C18A	C23A	C26A	-35(5)
C7	C16	N1A	Mn1	-21.1(17)	C18A	C23A	C26A	C14A	169(3)
C7	C16	N1A	C24A	-134.5(17)	C18A	C23A	C26A	N1A	39(4)
C7	C16	N1A	C26A	112(2)	C23A	C26A	C14A	C15A	-21(4)
C8	N4	C4	C6	2(2)	C23A	C26A	C14A	N3A	176.8(19)
C8	N4	C4	C22	-164.1(11)	C23A	C26A	N1A	Mn1	-173.6(17)
C10	C1	C6	C4	3(2)	C23A	C26A	N1A	C16	53(3)
C10	C1	C6	C5	-179.3(17)	C23A	C26A	N1A	C24A	-60(2)
O6	S2	C27	F3	-170.2(18)	C26A	C14A	C15A	C17A	168(2)
O6	S2	C27	F5	50(2)	C26A	C14A	N3A	Mn1	18.3(17)
O6	S2	C27	F6	-70(2)	C26A	C14A	N3A	C22	-94.0(15)
C11	C7	C16	N1	-154.5(12)	C26A	C14A	N3A	C19A	136.0(16)

C11	C7	C16	N1A	-172.1(15)	C14A	C26A	N1A	Mn1	58.5(16)
O7	S2	C27	F3	-48(2)	C14A	C26A	N1A	C16	-75(2)
O7	S2	C27	F5	172(2)	C14A	C26A	N1A	C24A	172.3(16)
O7	S2	C27	F6	52(3)	C14A	C15A	C17A	C28A	65(3)
O8	S1	C25	F1	-56(3)	C15A	C14A	N3A	Mn1	-149.2(15)
O8	S1	C25	F2	61(2)	C15A	C14A	N3A	C22	98.5(17)
O8	S1	C25	F4	-169(2)	C15A	C14A	N3A	C19A	-31(2)
C13	C11	C12	C3	177.5(15)	C15A	C17A	C28A	C19A	-29(3)
C14	N3	C19	C28	49.0(19)	C17A	C28A	C19A	N3A	-32(3)
C14	N3	C22	C4	145.9(10)	C28A	C19A	N3A	Mn1	-178.4(18)
C14	C15	C17	C28	-59(3)	C28A	C19A	N3A	C22	-39(3)
C15	C14	C26	N1	179.8(18)	C28A	C19A	N3A	C14A	66(2)
C15	C14	C26	C23	-49(3)	N1A	C24A	C20A	C18A	-76(3)
C15	C17	C28	C19	62(2)	N1A	C26A	C14A	C15A	110(3)
C16	N1	C24	C20	-69(2)	N1A	C26A	C14A	N3A	-52.4(19)
C16	N1	C26	C14	-118.7(14)	N3A	C14A	C15A	C17A	-29(3)

Table S-27 Hydrogen Atom Coordinates (Å×104) and Isotropic Displacement Parameters (Å2×103) for [*meso*-$L^1Mn(H_2O)_2]^{2+}$.

Atom	x	y	z	U(eq)
H1A	5659.54	6377.79	4324.82	114
H1B	5622.53	5739.2	5026.26	114
H3A	4262.13	6464.72	5646.8	123
H3B	4580	5712.86	5388.84	123
H1	2589.95	7636.41	524.21	77
H2	5479.51	6552.91	7229.63	67
H5D	4934.74	3961.31	4688.78	179
H5E	4780.46	5022.12	4444.71	179
H5A	2104.32	9192.64	903.34	160
H5B	2571.42	9788.09	2025.9	160
H5C	2096.2	8783.13	1892.32	160
H8	4363.02	6476.2	2746.46	82
H9A	3607.07	6469.84	322.66	218
H9B	2863.91	6184.54	150.24	218
H9C	3562.46	5520.76	983.72	218
H12	7469.63	7726	9497.33	96
H13A	7629.42	9352.76	7740.32	175
H13B	8035.51	9069.65	8977.07	175
H13C	7438.78	9976.84	8465.39	175
H14	5008.44	9415.16	6182.49	91
H15A	4786.11	11135.91	6358.46	95
H15B	4091.33	11118.61	5164.79	95
H16A	6629.16	9494.04	6279.19	83
H16B	6548.89	8327.24	5807.81	83
H16C	6509.83	9580.66	6240.06	83
H16D	6687.94	8450.41	5980.41	83
H17A	4306.87	9754.71	6847.78	104

H17B	3764.39	10737.33	6366.45	104
H18A	6350.1	11178.56	5542.06	124
H18B	5858.23	11789.14	4440.61	124
H19A	3406.77	8074.24	4582.59	61
H19B	4060.07	8111.81	5807.15	61
H20A	6543.85	10079.79	4632.15	112
H20B	5852.97	10460.4	3517.91	112
H21A	5954.72	6328.2	9122.16	128
H21B	6776.09	6527.31	10034.38	128
H21C	6548.07	5548.11	9232.02	128
H22A	3975.13	9932.56	3545.59	69
H22B	3311.72	9622.92	3602.45	69
H22C	3831.84	9984.6	3394.35	69
H22D	3334.83	9494.63	3725.36	69
H23A	4850.43	11015.32	4199.8	98
H23B	5294.19	11644.12	5310.65	98
H24A	5138.64	9271.65	3583.26	108
H24B	5860.99	8605.88	4116.82	108
H26	5766.58	10165.21	6256.92	64
H28A	3088.83	9848.3	4685.78	107
H28B	3122.16	9151.11	5597.84	107
H24C	6224.05	7761.96	4663.99	103
H24D	5637.61	8459.27	3654.5	103
H20C	6864.5	9491.14	5444.77	86
H20D	6900.11	9040.1	4483.26	86
H18C	5923.65	10035.61	3250.43	95
H18D	6477.39	10829.81	4166.12	95
H23C	5212.62	10990.32	3493.42	83
H23D	5791.68	11115.8	4744.74	83
H26A	4896.52	9371.55	3648.81	39
H14A	4409.62	10294.8	3929.67	69
H15C	4782.86	11744.75	4735.34	59
H15D	5185.71	11198.87	5879.24	59
H17C	3672.41	11217.79	4419.52	50
H17D	4104.15	12011.59	5409.81	50
H28C	3568.47	10291.65	5603.22	80
H28D	4304.49	10740.18	6610.74	80
H19C	4967.65	9491.35	6473.89	50
H19D	4262.04	8815.13	6045.2	50

Table S-28 Atomic Occupancy for [*meso*-$L^1Mn(H_2O)_2]^{2+}$.

Atom	Occupancy	Atom	Occupancy	Atom	Occupancy
S1	0.459(14)	S2	0.543(13)	N1	0.629(13)
N3	0.629(13)	F1	0.459(14)	O2	0.459(14)
O4	0.543(13)	F2	0.459(14)	O6	0.543(13)
O7	0.543(13)	F3	0.543(13)	O8	0.459(14)
F4	0.459(14)	C14	0.629(13)	H14	0.629(13)
C15	0.629(13)	H15A	0.629(13)	H15B	0.629(13)

H16A	0.629(13)	H16B	0.629(13)	H16C	0.371(13)
H16D	0.371(13)	C17	0.629(13)	H17A	0.629(13)
H17B	0.629(13)	C18	0.629(13)	H18A	0.629(13)
H18B	0.629(13)	C19	0.629(13)	H19A	0.629(13)
H19B	0.629(13)	C20	0.629(13)	H20A	0.629(13)
H20B	0.629(13)	H22A	0.629(13)	H22B	0.629(13)
H22C	0.371(13)	H22D	0.371(13)	C23	0.629(13)
H23A	0.629(13)	H23B	0.629(13)	C24	0.629(13)
H24A	0.629(13)	H24B	0.629(13)	C25	0.459(14)
C26	0.629(13)	H26	0.629(13)	F5	0.543(13)
C27	0.543(13)	O9	0.459(14)	C28	0.629(13)
H28A	0.629(13)	H28B	0.629(13)	F6	0.543(13)
S1A	0.541(14)	O2A	0.541(14)	O8A	0.541(14)
C25A	0.541(14)	O9A	0.541(14)	F2A	0.541(14)
F1A	0.541(14)	F4A	0.541(14)	S2A	0.457(13)
O4A	0.457(13)	O6A	0.457(13)	O7A	0.457(13)
C27A	0.457(13)	F5A	0.457(13)	F6A	0.457(13)
F3A	0.457(13)	C24A	0.371(13)	H24C	0.371(13)
H24D	0.371(13)	C20A	0.371(13)	H20C	0.371(13)
H20D	0.371(13)	C18A	0.371(13)	H18C	0.371(13)
H18D	0.371(13)	C23A	0.371(13)	H23C	0.371(13)
H23D	0.371(13)	C26A	0.371(13)	H26A	0.371(13)
C14A	0.371(13)	H14A	0.371(13)	C15A	0.371(13)
H15C	0.371(13)	H15D	0.371(13)	C17A	0.371(13)
H17C	0.371(13)	H17D	0.371(13)	C28A	0.371(13)
H28C	0.371(13)	H28D	0.371(13)	C19A	0.371(13)
H19C	0.371(13)	H19D	0.371(13)	N1A	0.371(13)
N3A	0.371(13)				

12. References

[1] (a) A. M. Prince, D. Pascual, D. Meruelo, L. Liebes, Y. Mazur, E. Dubovi, M. Mandel, G. Lavie, *Photochem. Photobiol.* **2000**, *71*, 188–195. (b) S. Rahimipour, C. Palivan, F. Barbosa, I. Bilkis, Y. Koch, L. Weiner, M. Fridkin, Y. Mazur, G. Gescheidt, *J. Am. Chem. Soc.* **2003**, *125*, 1376–1384. (c) M. van de Putte, T. Roskams, J. R. Vandenheede, P. Agostinis, P. A. M. de Witte, *Br. J. Cancer.* **2005**, *92*, 1406–1413. (d) M. Misiek, J. Williams, K. Schmich, W. Hüttel, I. Merfort, C. E. Salomon, C. C. Aldrich, D. Hoffmeister, *J. Nat. Prod.* **2009**, *72*, 1888–1891. (e) M. Misiek, D. Hoffmeister, *Mycol. Prog.* **2012**, *11*, 7–15. (f) H. Hajime Kobori, A. Sekiya, T. Suzuki, J. H. Choi, H. Hirai, H. Kawagishi, *J. Nat. Prod.* **2015**, *78*, 163–167. (g) L. J. Scott, *Drugs* **2007**, *67*, 761–770. (h) J. C. L. Erve, S. Gauby, J. W. Maynard, Jr. M. A. Svensson, G. Tonn, K. P. Quinn, *Chem. Res. Toxicol.* **2013**, *26*, 926–936. (i) D. T. Wicklow, B. K. Joshi, W. R. Gamble, J. B. Gloer, P. F. Dowd, *Appl. Environ. Microbiol.* **1998**, *64*, 4482–4484. (j) E. Moulin, V. Zoete, S. Barluenga, M. Karplus, N. Winssinger, *J. Am. Chem. Soc.* **2005**, *127*, 6999–7004. (k) S. B. Singh, D. L. Zink, J. M. Liesch, R. G. Ball, M. A. Goetz, E. A. Bolessa, R. A. Giacobbe, K. C. Silverman, G. F. Bills, F. Pelaez, C. Cascales, J. B. Gibbs, R. B. Lingham, *J. Org. Chem.* **1994**, *59*, 6296. (l) T. Ukita, Y. Nakamura, A. Kubo, Y. Yamamoto, Y. Moritani, K. Saruta, T. Higashijima, J. Kotera, M. Takagi, K. Kikkawa, K. Omori, *J. Med. Chem.* **2001**, *44*, 2204–2218. (m) T. Y. Wong, R. Simó, P. Mitchell, *Am. J. Ophthalmol.* **2012**, *154*, 6−12. (n) C. D. Borsarelli, S. E. Braslavsky, S. Sortino, G. Marconi, S. Monti, *Photochem. Photobiol.* **2000**, 72, 163-171. (o) L. Wu, H. Ling, L. Li, L. Jiang, M. He, *J. Pharm. Pharmacol.* **2007**, 59, 695− 701. (p) N. Masuoka, T. Isobe, I. Kubo, *Phytother. Res.* **2006**, *20*, 206. (q) K. D. Hargrave, F. K. Hess, J. T. Oliver, *J. Med. Chem.* **1983**, *26*, 1158–1163. (r) L. M. Bang, K. L. Goa, *CNS Drugs* **2004**, *18*, 57–61. (s) K. P. Bogeso, A. V. Christensen, J. Hyttel， T. Liljefors, *J. Med. Chem.* **1985**, *28*, 1817–1828. (t) X. Wang, P. A. Bhatia, J. F. Daanen, S. P. Latsaw, J. Rohde, T. Kolasa, A. A. Hakeem, M. A. Matulenko, M. Nakane, M. E. Uchic, L. N. Miller, R. Chang, R. B. Moreland, J. D. Brioni, A. O. Stewart, *Bioorg. Med. Chem.* **2005**, *13*, 4667−4678. (u) D. de Chaffoy De Courcelles, J. E. Leysen, P. Roevens, H. Van Belle, *Drug Dev Res* **1986**; 8, 173–178 (v) C. T. Mbofana, E. Chong, J. Lawniczak, M. S. Sanford, *Org. Lett.* **2016**, *18*, 4258-4261. (w) E. Hu, N. Chen, R. K. Kunz, D. R. Hwang, K. Michelsen, C. Davis,

J. Ma, J. Shi, D. Lester-Zeiner, R. Hungate, J. Treanor, H. Chen, J. R. Allen, *ACS Med. Chem. Lett.* **2016**, *7*, 719−723.

[2] (a) K. L. Rinehart, Jr. J. Kobayashi, G. C. Harbour, J. Gilmore, M. Mascal, T. G. Holt, L. S. Shield, F. Lafargue, *J. Am. Chem. Soc.* **1987**, *109*, 3378-3387. (b) J. S. Shi, J. X. Yu, X. P. Chen, R. X. Xu, *Acta Pharmacol. Sin.* **2003**, *24*, 97−101. (c) J. M. Goldstein, L. A. Arvanitis, *CNS Drug Rev.* **1995**, *1*, 50-73. (d) A. Fitton, L. Wiseman, *Drugs* **1996**, *51*, 460−482. (e) G. DeSantis, Z. Zhu, W. A. Greenberg, K. Wong, J. Chaplin, S. R. Hanson, B. Farwell, L. W. Nicholson, C. L. Rand, D. P. Weiner, D. E. Robertson, M. J. Burk, *J. Am. Chem. Soc.* **2002**, *124*, 9024-9025. (f) S. L. Cuddihy, S. Drake, D. T. Harwood, A. I. Selwood, P. S. McNabb, M. B. Hampton, *Apoptosis* **2016**, *21*, 1447−1452. (g) M. Shamma, R. J. Shine, I. Kompis, T. Sticzay, F. Morsingh, J. Poisson, J. L. Pousset, *J. Am. Chem. Soc.* **1967**, *89*, 1739−1740. (h) C. H. Mitch, S. J. Quimby, N. Diaz, C. Pedregal, M. G. de la Torre, A. Jimenez, Q. Shi, E. J. Canada, S. D. Kahl, M. A. Statnick, D. L. McKinzie, D. R. Benesh, K. S. Rash, V. N. Barth, *J. Med. Chem.* **2011**, *54*, 8000-8012. (i) J. C. Lauffenburger, C. L. Mayer, R. L. Hawke, K. L. Brouwer, M. W. Fried, J. F. Farley, *Eur. J. Gastroenterol Hepatol.* **2014**; *26*, 1073-1082. (j) A. W. Sobanska, G. Zydek, P. Wlodno, E. Brzezinska, *Eur. J. Med. Chem.* **2015**; *89*, 147-155. (k) G. Bringmann, M. Dreyer, J. H. Faber, P. W. Dalsgaard, J. W. Jaroszewski, H. Ndangalasi, F. Mbago, P. Brun, S. B. Christensen, *J. Nat. Prod.* **2004**, *67*, 743−748. (l) A. J. Wagstaff, G. M. Keating, *Drugs* **2006**, 66, 111-131. (m) R. L. Stedman, *Chem. Rev.* **1968**, *68*, 153-207. (n) M. R. Birck, T. P. Holler, R. W. Woodard, *J. Am. Chem. Soc.* **2000**, *122*, 9334−9335. (o) F. M. Cordero, D. Giomi, A. Brandi, *Curr. Top. Med Chem.* **2014**, *14*, 1294–1307. (p) T. Reynolds, *Phytochemistry* **2005**, *66*, 1399-1406. (q) B. E. Maryanoff, D. F. McComsey, M. J. Costanzo, P. E. Setler, J. F. Gardocki, R. P. Shank, C. R. Schneider, *J. Med. Chem.* **1984**, *27*, 943−946. (r) G. Kim, S. D. Jung, W. J. Kim, *Org. Lett.* **2001**, 3, 2985-2898. (s) N. Yamazaki, C. Kibayashi, *J. Am. Chem. Soc.* **1989**, *111*, 1396-1408. (t) W. R. Kem, K. Wildeboer, S. LeFrancois, M. Raja, W. Marszalec, J. C. Braekman, *Cell. Mol. Neurobiol.* **2004**, *24*, 535-551.

[3] (a) M. Shon, R. Shanmugavel, G. Shin, S. Mathew, S. H. Lee, H. Yun, *Chem. Commun.* **2014**, *50*, 12680−12683. (b) S. P. France, S. Hussain, A. M. Hill, L. J. Hepworth, R. M. Howard, K. R. Mulholland, S. L. Flitsch, N. J. Turner, *ACS Catal.* **2016**, *6*, 3753−3759. (c) A. A. Siddiqui, S, Partapa, S. Khisala, M. S. Yara, R.

Mishra, *Bioorg. Chem.* **2020**, *99*, 103584. (d) Y. Y. Hua, H. Y. Bin, T. Wei, H. A. Cheng, Z. P. Lin, X. F. Fu, Y. Q. Li, J. H. Xie, P. C. Yan, Q. L. Zhou, *Org. Lett.* **2020**, *22*, 818-822. (e) D. S. Weinstein, W. Liu, C. Langevine, D. W. Combs, S. Zhuang, X. Chen, C. S. Madsen, T. W. Harper, S. Ahmad, J. A. Robl, K. Ngu, *Bioorg. Med. Chem. Lett.* **2011**, *21*, 4141-4145. (f) I. Aouani, B. Sellami, K. Lahbib, J. F. Cavalier, S. Touil, *Bioorg Chem* **2017**, *72*, 301–307. (g) H. Kusama, K. Ishida, H. Funami, N. Iwasawa, *Angew. Chem., Int. Ed.* **2008**, *47*, 4903−4905; *Angew. Chem.* **2008**, *120*, 4981−4983. (h) Y. Zhang, Q. Yan, G. Zi, G. Hou, *Org. Lett.* **2017**, *19*, 4215−4218. (i) Y. Zhang, D. Kong, R. Wang, G. Hou, *Org. Biomol. Chem.* **2017**, 15, 3006-3012. (j) N. Coia, N. Mokhtari, J. L. Vasse, J. Szymoniak, *Org. Lett.* 2011, *13*, 6292-6295.

[4] (a) M. S. Chen, M. C. White, *Science* **2007**, *318*, 783−787. (b) E. A. Mikhalyova, O. V. Makhlynets, T. D. Palluccio, A. S. Filatov, E. V. Rybak-Akimova, *Chem. Commun.* **2012**, *48*, 687− 689. (c) O. Cussó, I. Garcia-Bosch, X. Ribas, J. Lloret-Fillol, M. Costas, *J. Am. Chem. Soc.* **2013**, *135*, 14871−14878. (d) R. V. Ottenbacher, D. G. Samsonenko, E. P. Talsi, K. P. Bryliakov, *ACS Catal.* **2014**, *4*, 1599−1606. (e) D. Shen, C. Miao, S. Wang, C. Xia, W. Sun, *Org. Lett.* **2014**, *16*, 1108−1111.

[5] J. Li, S. Qu, W. Zhao, *Angew. Chem., Int. Ed.* **2020**, *59*, 2360−2364; *Angew. Chem.* **2020**, *132*, 2380−2384.

[6] H. Chen, W. Jin, S. Yu, *Org. Lett.* **2020**, *22*, 5910−5914.

[7] (a) D. T. Genna, G. H. Posner, *Org. Lett.* **2011**, *13*, 5358-5361. (b) J. C. Gilbert, D. H. Giamalva, U. Weerasooriya, *J. Org. Chem.* **1983**, *48*, 5251-5256.

[8] (a) L. A. Clarke, A. Ring, A. Ford, A. S. Sinha, S. E. Lawrence, A. R. Maguire, *Org. Biomol. Chem.* **2014**, *12*, 7612−7628. (b) G. Colombano, C. Travelli, U. Galli, A. Caldarelli, M. G. Chini, P. L. Canonico, G. Sorba, G. Bifulco, G. C. Tron, A. A. Genazzani, *J. Med. Chem.* **2010,** 53, 616-623.

[9] M. M. Mojtahedi, S. Samadian, *J. Chem.* **2013**, Article ID 642479.

[10] R. Kundu, Z. T. Ball, *Org. Lett.* **2010**, *12*, 2460−2463.

[11] M. Parravicini, L. Vaghi, G. Cravotto, N. Masciocchi, A. Maspero, G. Palmisano, A. Penoni, *Arkivoc*. **2014**, *6*, 72-85.

[12] A. K. Maity, S. Roy, *Adv. Synth. Catal.* **2014**, *356*, 2627-2642.

[13] (a) C. Q. Li, K. Lang, H. J. Lu, Y. Hu, X. Cui, L. Wojtas, X. P. Zhang, *Angew. Chem., Int. Ed.* **2018**, *57*, 16837−16841; *Angew. Chem.***2018**, *130*, 17079−17083. (b) S. Chandrasekhar, A. Raza, M. Takhi, *Tetrahedron: Asymmetry* **2002**, *13*, 423–428.

[14] S. Guo, J. C. Yang, S. L. Buchwald, *J. Am. Chem. Soc.* **2018**, *140*, 15976−15984.

[15] D. A. Petrone, H. Yoon, H. Weinstabl, M. Lautens, *Angew. Chem., Int. Ed.* **2014**, *53*, 7908−7912; *Angew. Chem.***2014**, *126*, 8042−8046.

[16] F. Poulhès, R. Sylvain, P. Perfetti, M. P. Bertrand, G. Gil, S. Gastaldi, *Synthesis* **2010**, 1334-1338.

[17] A. Koperniku, P. J. Foth, G. M. Sammis, L. L. Schafer, *J. Am. Chem. Soc.* **2019**, **141**, 18944−18948.

[18] H. Zhang, P. Tian, L. Ma, Y. Zhou, C. Jiang, X. Lin, X. Xiao, *Org. Lett.* **2020**, *22*, 997−1002.

[19] C. T. Mbofana, E. Chong, J. Lawniczak, M. S. Sanford, *Org. Lett.* **2016**, *18*, 4258−4261.

[21] S. K. Santra, A. M. Szpilman, *J. Org. Chem.* **2021**, *86*, 1164-1171.

[21] A. Jõgi, A. Paju, T. Kailus, A. M. Müürisepp, T. Kanger, M. Lopp, *Synthesis*, **2006**, 3031-3036.

[22] A. Mori, Y. Miyakawa, E. Ohashi, T. Haga, T. Maegawa, H. Sajiki, *Org. Lett.* **2006**, *8*, 3279−3281.

[23] A. A. Kiryanov, P. Sampson, A. J. Seed, *J. Mater. Chem.* **2001**, *11*, 3068–3077.

[24] T. Ikawa, H. Sajiki, K. Hirota, *Tetrahedron* 2005, 61, 2217−2231.

[25] K. Saigo, T. Yamashita, A. Hongu, M. Hasegawa, *Synth. Commun.* **1985**, *15*, 715-721.

[26] P. P. Thottumkara, T. K. Vinod, *Org. Lett.* **2010**, *12*, 5640−5643.

[27] G. Zhao, Y. He, Z. Xu, J. Hou, M. Zhang, J. Min, H. Y. Chen, M. Ye, Z. Hong, Y. Yang, Y. Li, *Adv. Funct. Mater.* **2010**, *20*, 1480−1487.

[28] J. A. Murphy, A. G. J. Commeureuc, T. N. Snaddon, T. M. McGuire, T. A. Khan, K. Hisler, M. L. Dewis, R. Carling, *Org. Lett.* **2005**, *7*, 1427−1429.

[29] P. H. Li, Y. Y. Wang, X. Wang, Y. Wang, Y. Liu, K. K. Huang, J. Hu, L. M. Duan, C. W. Hu, J. H. Liu, *J. Org. Chem.* **2020**, *85*, 3101−3109.

[30] B. Pandey, S. Xu, K. Ding, *Org. Lett.* 2019, 21, 7420−7423.

[31] G. Urgoitia, A. Maiztegi, R. SanMartin, M. T. Herrero, E. Domínguez, *RSC Adv.* **2015**, *5*, 103210−103217.

[32] K. P. Shing, B. Cao, Y. Liu, H. K. Lee, M. D. Li, D. L. Phillips, X. Y. Chang, C. M. Che, *J. Am. Chem. Soc.* **2018**, *140*, 7032−7042.

[33] S. L. Bartlett, C. M. Beaudry, *J. Org. Chem.* **2011**, *76*, 9852−9855.

[34] G. Z. Wang, R. Shang, W. M. Cheng, Y. Fu, *Org. Lett.* **2015**, *17*, 4830-4833.

[35] A. M. Echavarren, M. Pérez, A. M. Castano, J. M. Cuerva, *J. Org. Chem.* **1994**, *59*, 4179−4185.

[36] H. Ahlbrecht, M. Dietz, L. Weber, *Synthesis* 1987, 251-254.

[37] J. D. Grayson, B. M. Partridge, *ACS Catal.* **2019**, *9*, 4296− 4301.

[38] K. Moriyama, M. Takemura, H. Togo, *Org. Lett.* **2012**, *14*, 2414-2417.

[39] H. Kusama, K. Ishida, H. Funami, N. Iwasawa, *Angew. Chem., Int. Ed.* **2008**, *47*, 4903−4905; *Angew. Chem., Int. Ed.* **2008**, *120*, 4981−4983.

[40] Z. Zhan, H. Ma, X. Cui, P. Jiang, J. Pu, Y. Zhang, G. Huang, *Org. Biomol. Chem.* **2019**, *17*, 5148–5152.

[41] F. Haász, V. Galamb, *Synth. Commun.* **1994**, *24*, 683-687.

[42] T. Sueda, A. Kawada, Y. Urashi, N. Teno, *Org. Lett.* **2013**, *15*, 1560−1563.

[43] X. Han, *Tetrahedron Lett.* **2007**, *48*, 2845–2849.

[44] K. Merkens, F. J. Aguilar Troyano, K. Anwar, A. Gomez-Suarez, *J. Org. Chem.* **2021**, *86*, 8448−8456.

[45] F. Chen, Z. Ding, J. Qin, T. Wang, Y. He, Q. H. Fan, *Org. Lett.* **2011**, *13*, 4348−4351.

[46] D. S. Wang, Z. S. Ye, Q. A. Chen, Y. G. Zhou, C. B. Yu, H. J. Fan, Y. Duan, *J. Am. Chem. Soc.* **2011**, *133*, 8866−8869.

[47] F. Huang, S. Zhang, *Org. Lett.* **2019**, *21*, 7430-7434.

[48] C. G. Savarin, C. Grise, J. A. Murry, R. A. Reamer, D. L. Hughes, *Org. Lett.* **2007**, *9*, 981−983.

[49] W. L. Lei, K. W. Feng, T. Wang, L. Z. Wu, Q. Liu, *Org. Lett.* **2018**, *20*, 7220−7224.

[50] J. W. Hulshof, P. Casarosa, W. M. Menge, L. M. Kuusisto, H. van der Goot, M. J. Smit, I. J. de Esch, R. Leurs, *J. Med. Chem.* **2005**, *48*, 6461−6471.

[51] A. J. Pearson, G. R. Han, *J. Org. Chem.* **1985**, *50*, 2791– 2792.

13. ^{1}H and ^{13}C NMR spectra

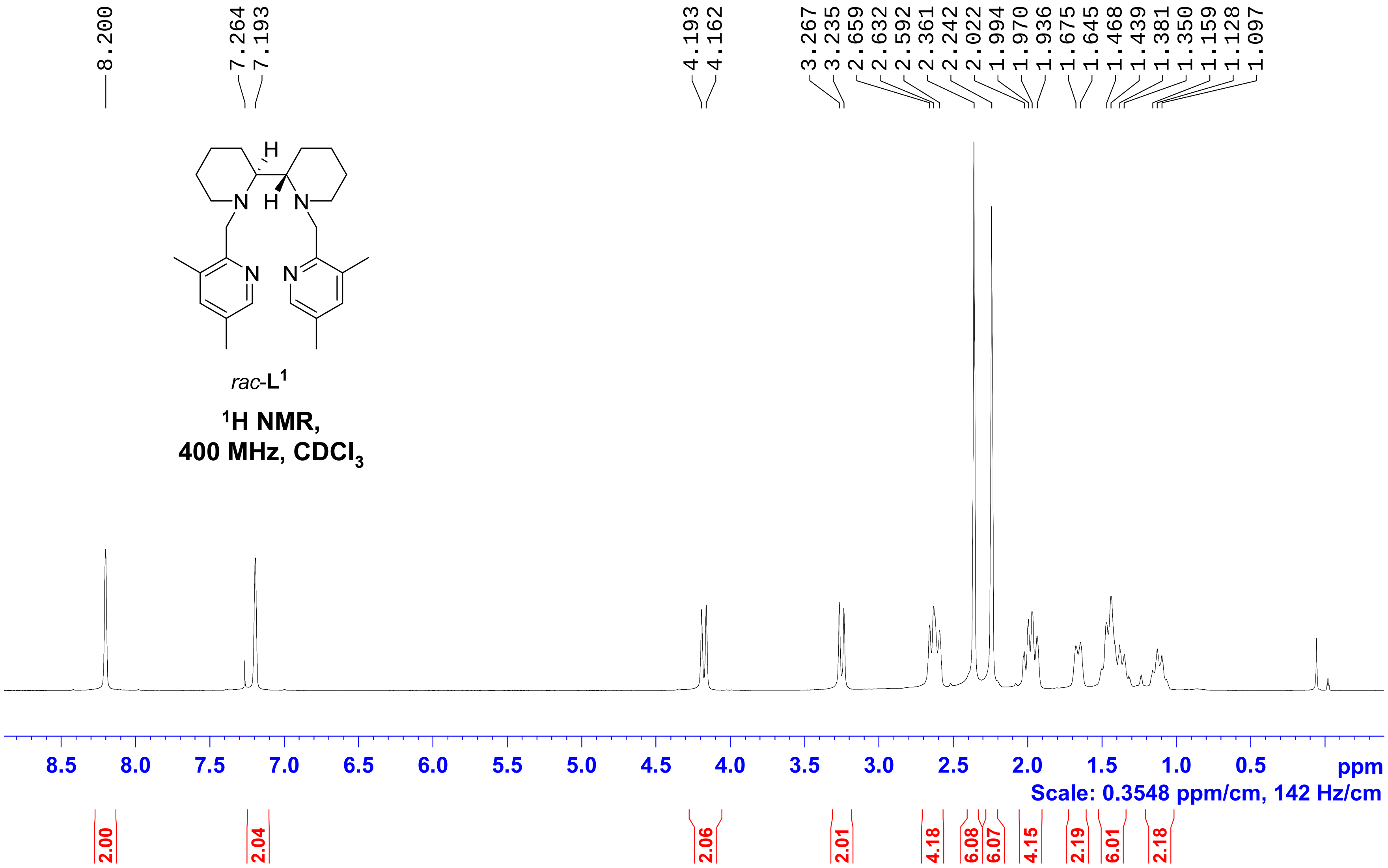

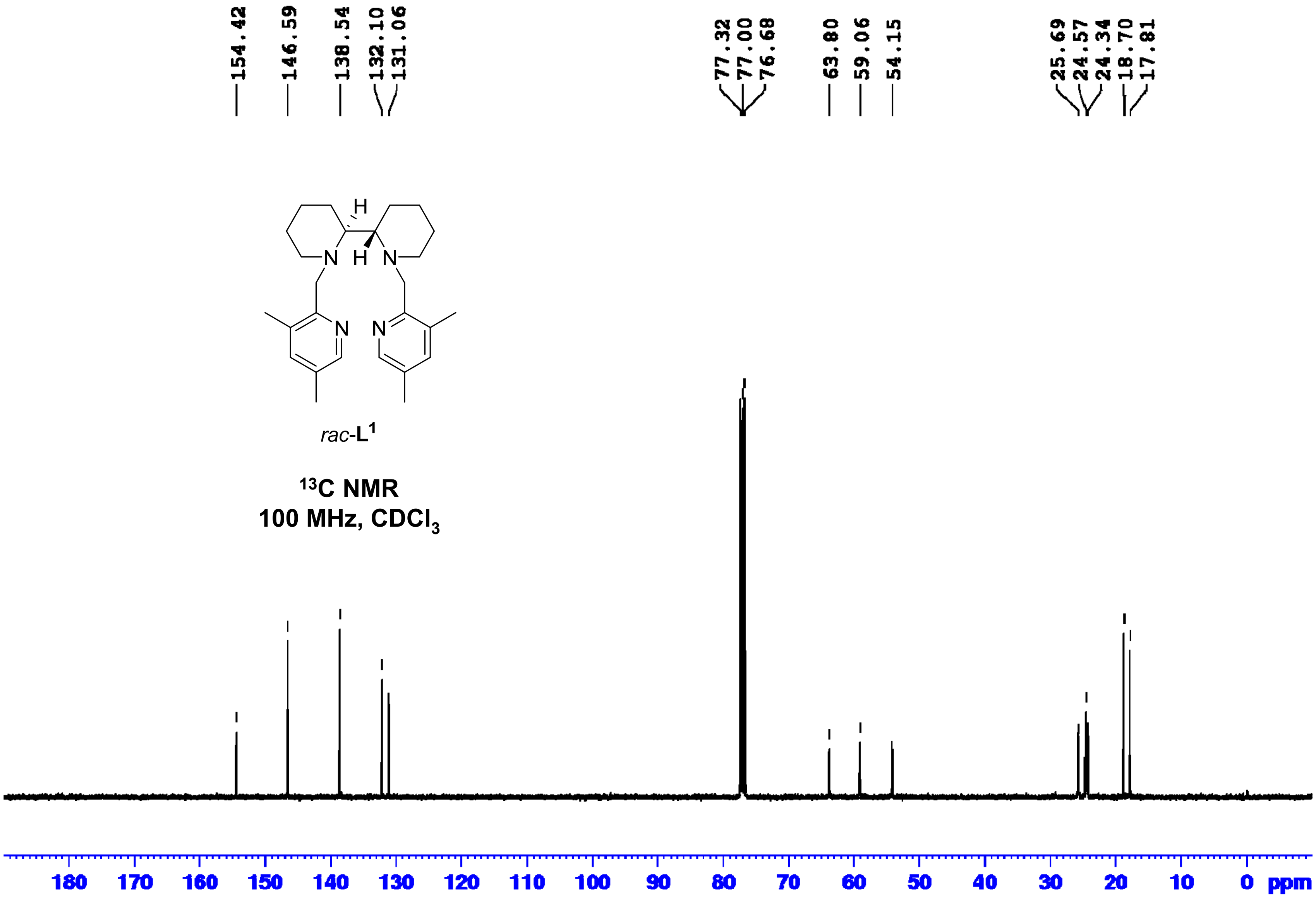
154.42
146.59
138.54
132.10
131.06
77.32
77.00
76.68
63.80
59.06
54.15
25.69
24.57
24.34
18.70
17.81
H
N
H
N
N
N
rac-L1
13C NMR
100 MHz, CDCl3
180 170 160 150 140 130 120 110 100 90 80 70 60 50 40 30 20 10 0 ppm

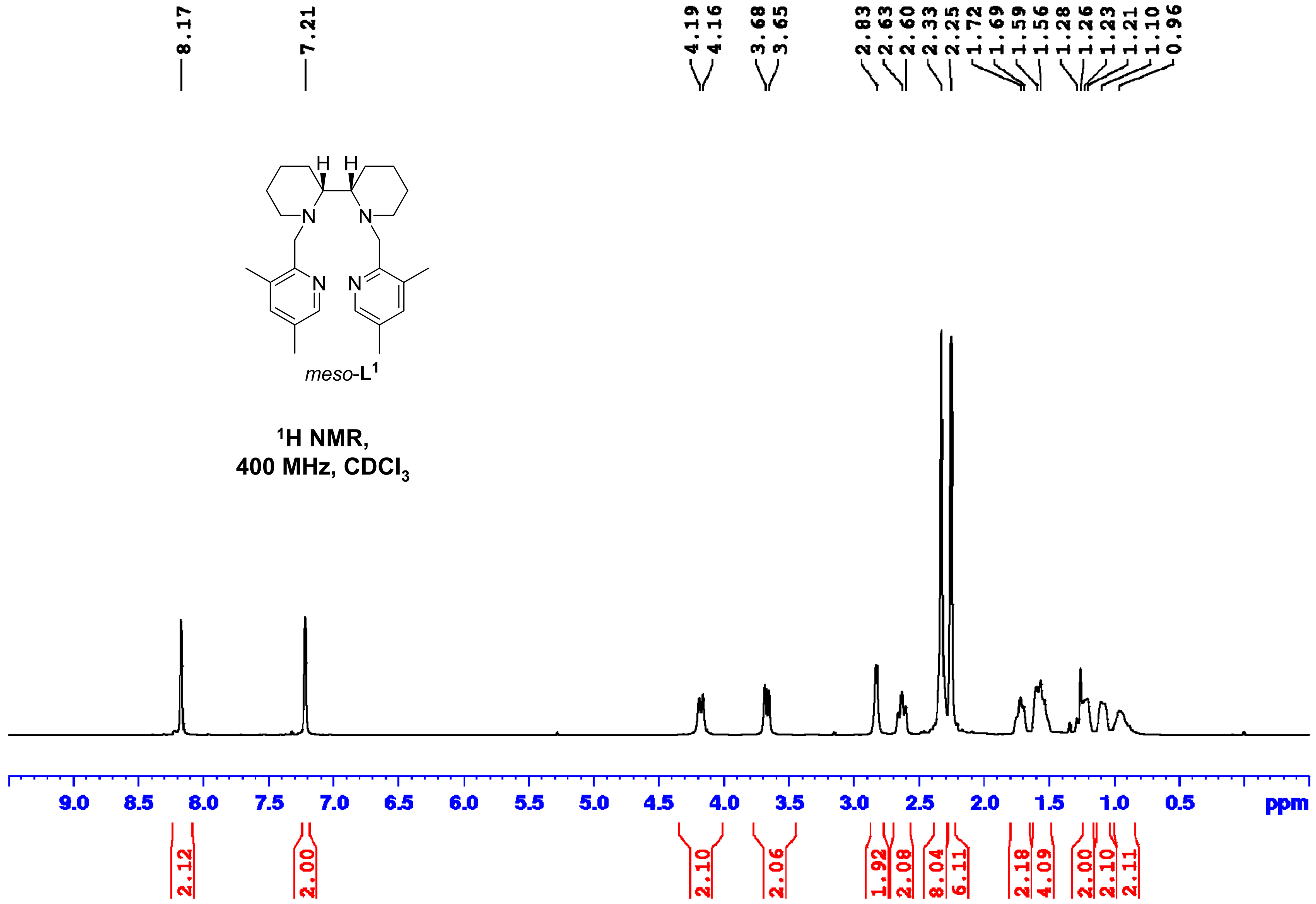
8.17
7.21
4.19
4.16
3.68
3.65
2.83
2.63
2.60
2.33
2.25
1.72
1.69
1.59
1.56
1.28
1.26
1.23
1.21
1.10
0.96
H H
N N
N N
meso-L1
1H NMR,
400 MHz, CDCl3
9.0 8.5 8.0 7.5 7.0 6.5 6.0 5.5 5.0 4.5 4.0 3.5 3.0 2.5 2.0 1.5 1.0 0.5 ppm
2.12
2.00
2.10
2.06
1.92
2.08
8.04
6.11
2.18
4.09
2.00
2.10
2.11

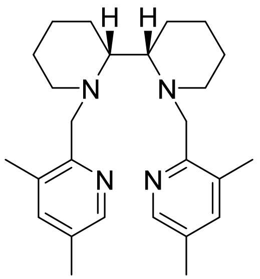

meso-**L¹**

^{13}C NMR
100 MHz, $CDCl_3$

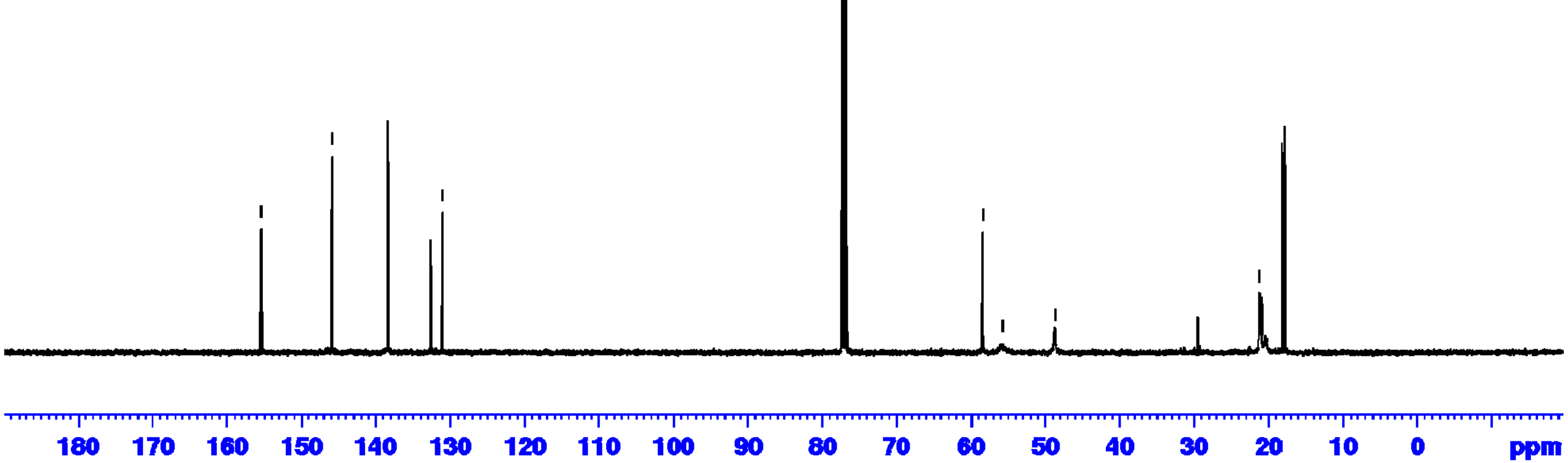

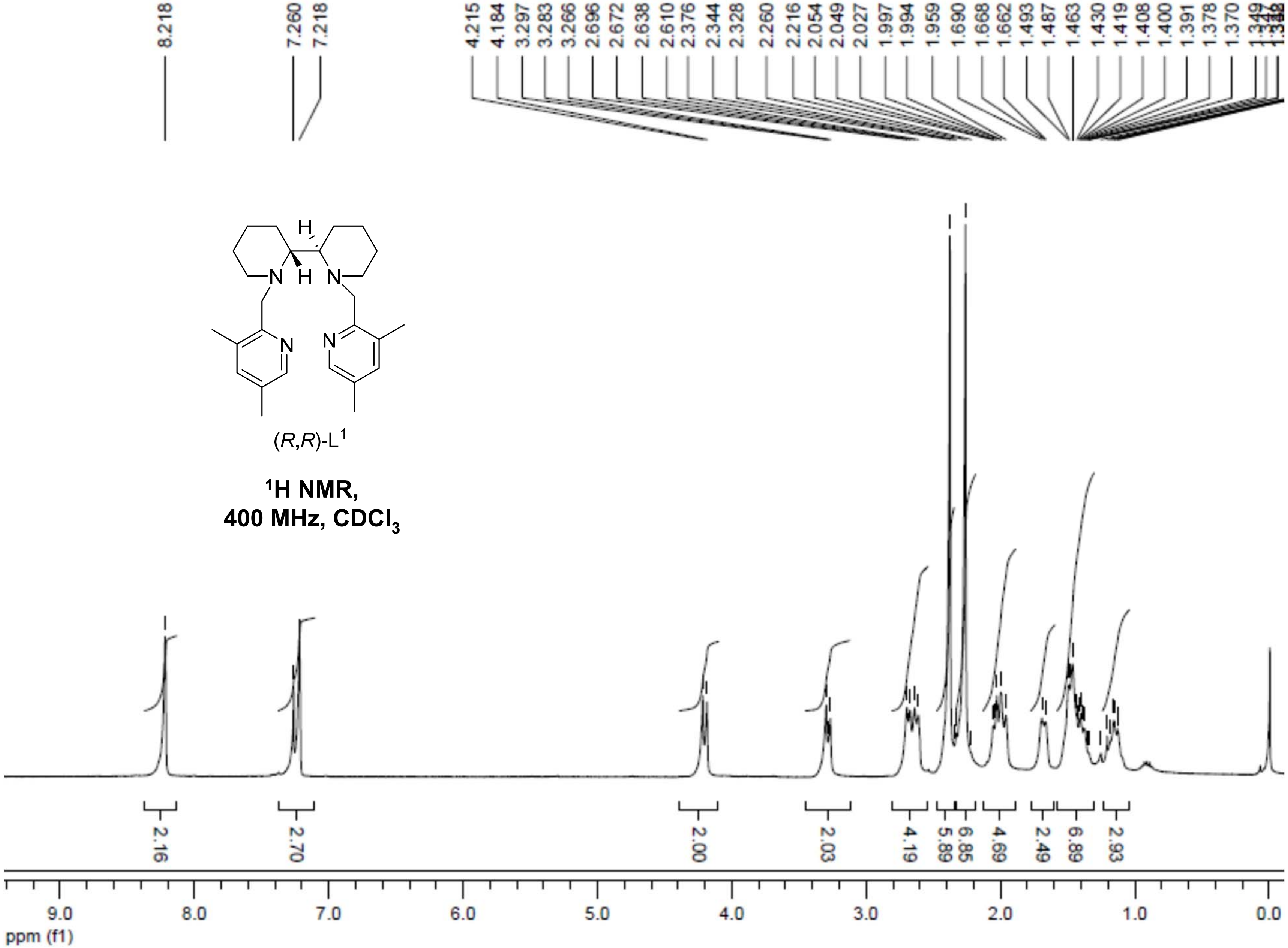

(R,R)-L[1]

^{1}H NMR,
400 MHz, $CDCl_3$

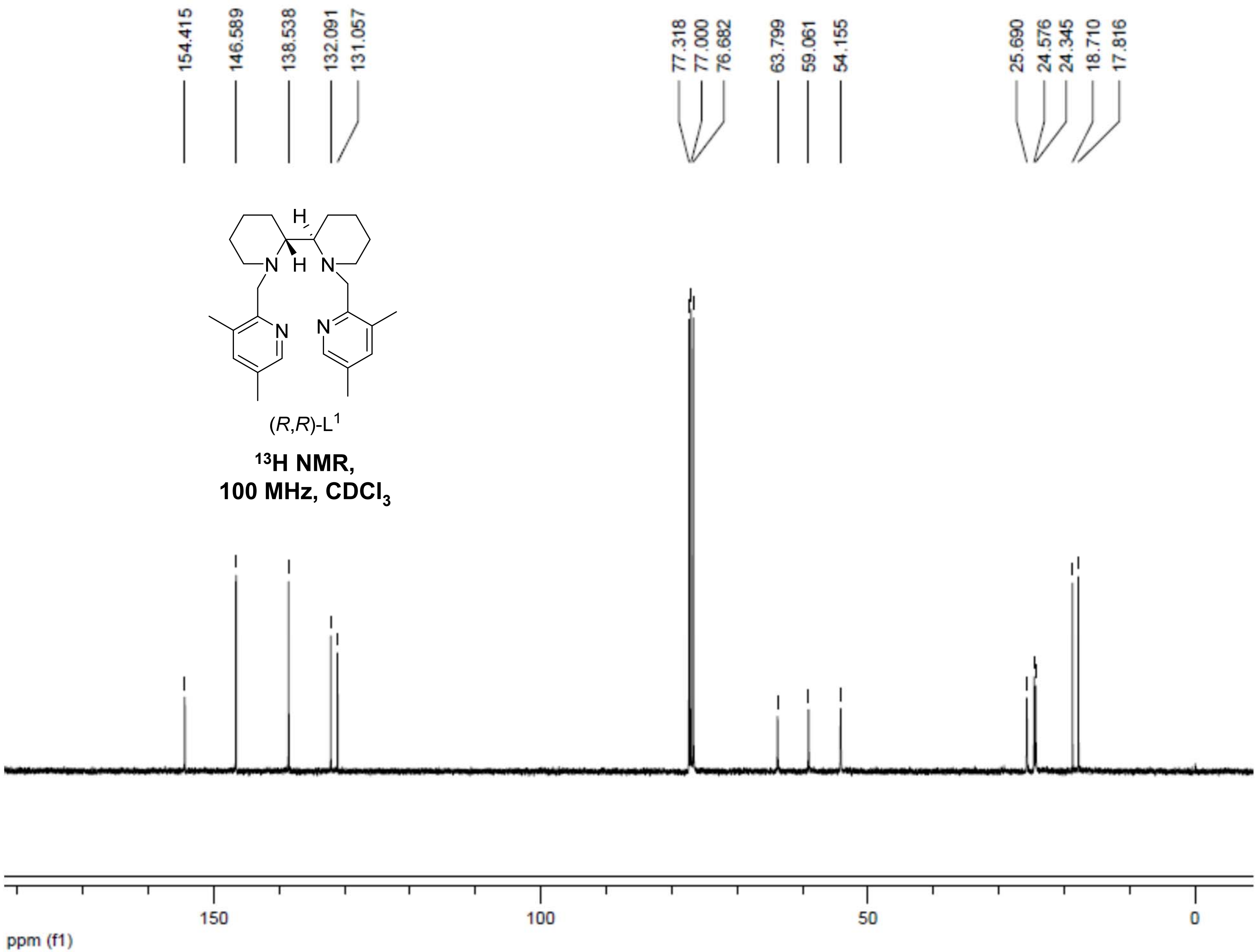
154.415
146.589
138.538
132.091
131.057
77.318
77.000
76.682
63.799
59.061
54.155
25.690
24.576
24.345
18.710
17.816
(R,R)-L1
13H NMR,
100 MHz, CDCl3
150
100
50
0
ppm (f1)

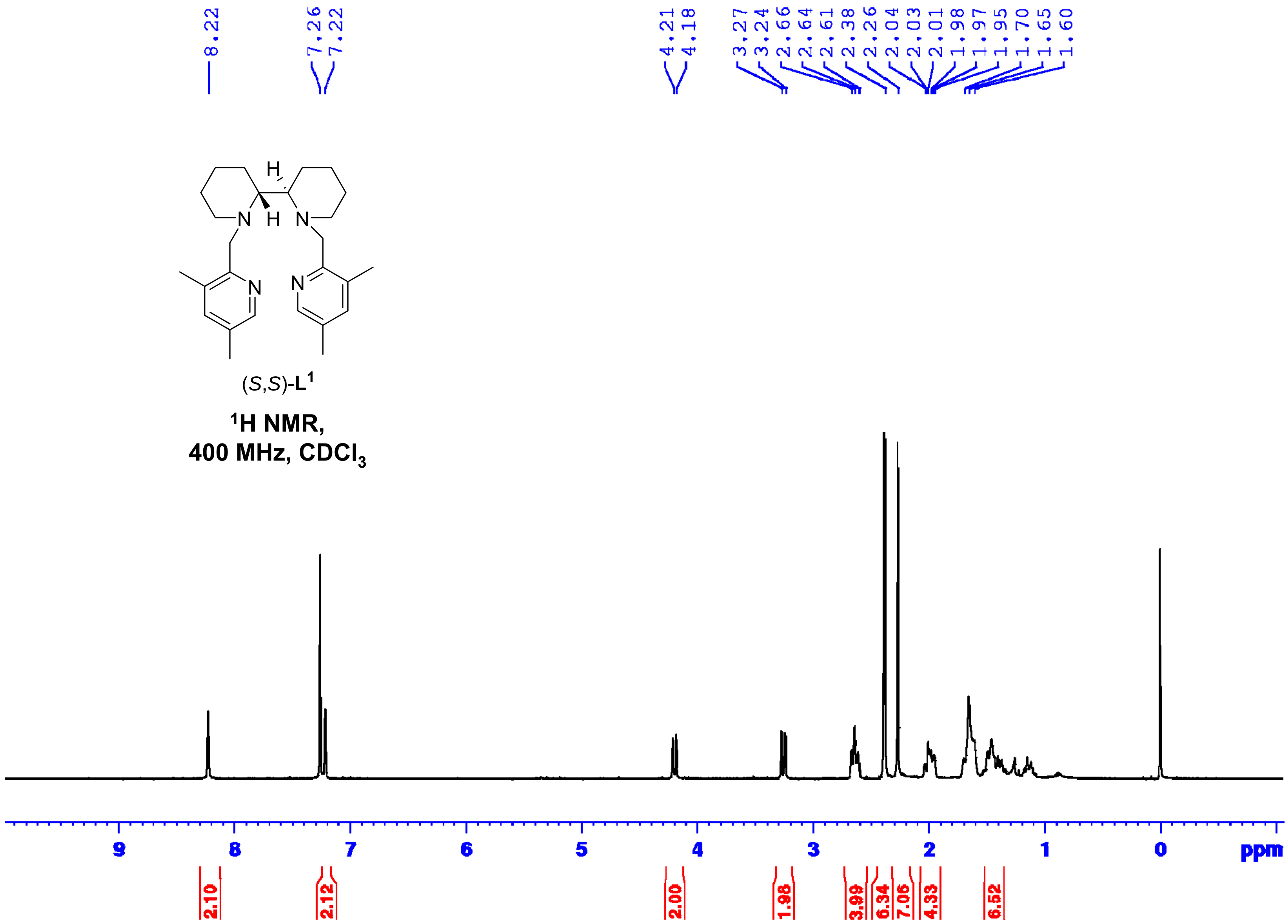
8.22
7.26
7.22
4.21
4.18
3.27
3.24
2.66
2.64
2.61
2.38
2.26
2.04
2.03
2.01
1.98
1.97
1.95
1.70
1.65
1.60
H
N
H
N
N
N
(S,S)-L1
1H NMR,
400 MHz, CDCl3
9
8
7
6
5
4
3
2
1
0
ppm
2.10
2.12
2.00
1.98
3.99
6.34
7.06
4.33
6.52

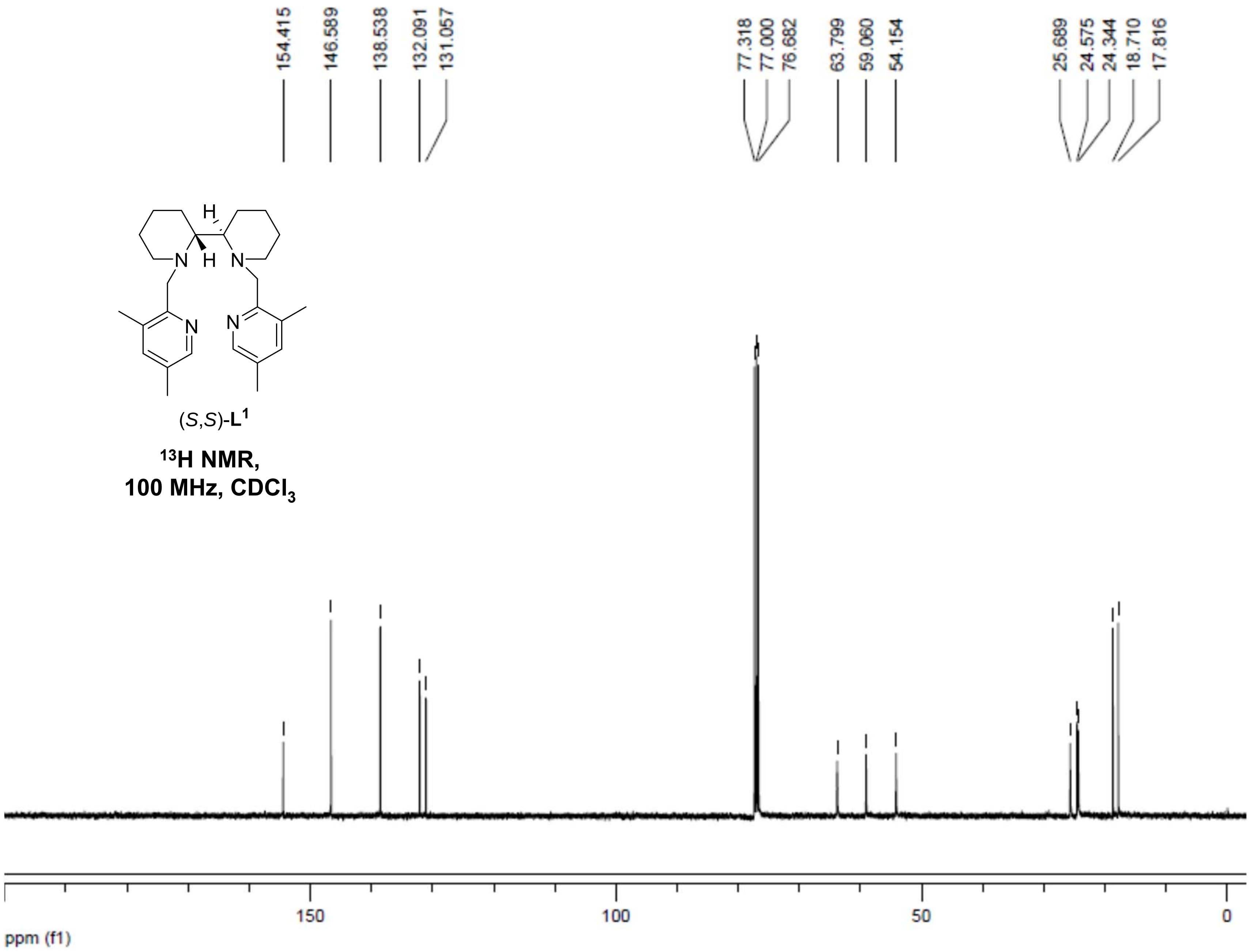
154.415
146.589
138.538
132.091
131.057
77.318
77.000
76.682
63.799
59.060
54.154
25.689
24.575
24.344
18.710
17.816
H
N
H
N
N
N
(S,S)-L1
13H NMR,
100 MHz, CDCl3
150
100
50
0
ppm (f1)

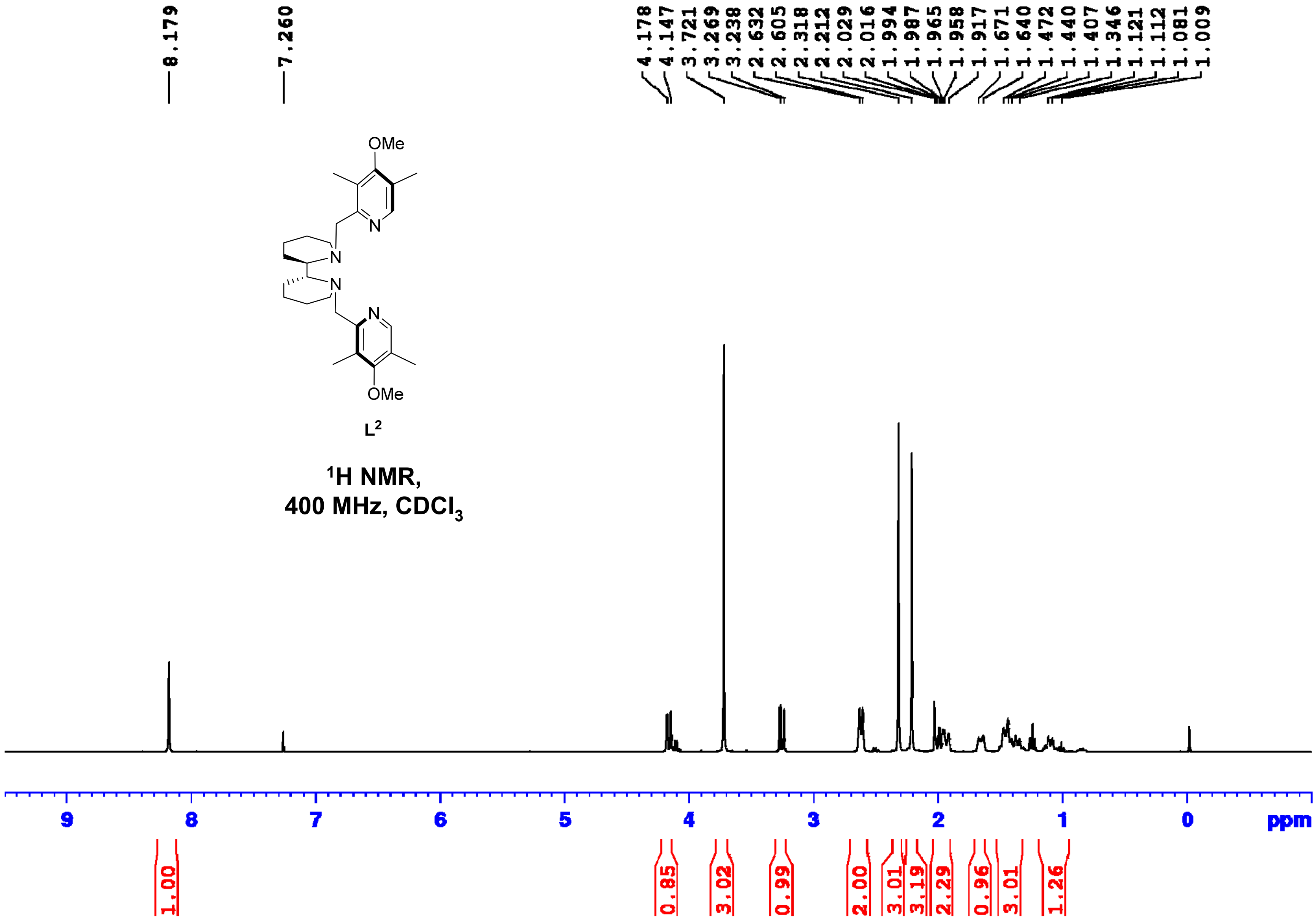
8.179
7.260
4.178
4.147
3.721
3.269
3.238
2.632
2.605
2.318
2.212
2.029
2.016
1.994
1.987
1.965
1.958
1.917
1.671
1.640
1.472
1.440
1.407
1.346
1.121
1.112
1.081
1.009
OMe
N
N
N
N
OMe
L2
1H NMR,
400 MHz, CDCl3
9
8
7
6
5
4
3
2
1
0
ppm
1.00
0.85
3.02
0.99
2.00
3.01
3.19
2.29
0.96
3.01
1.26

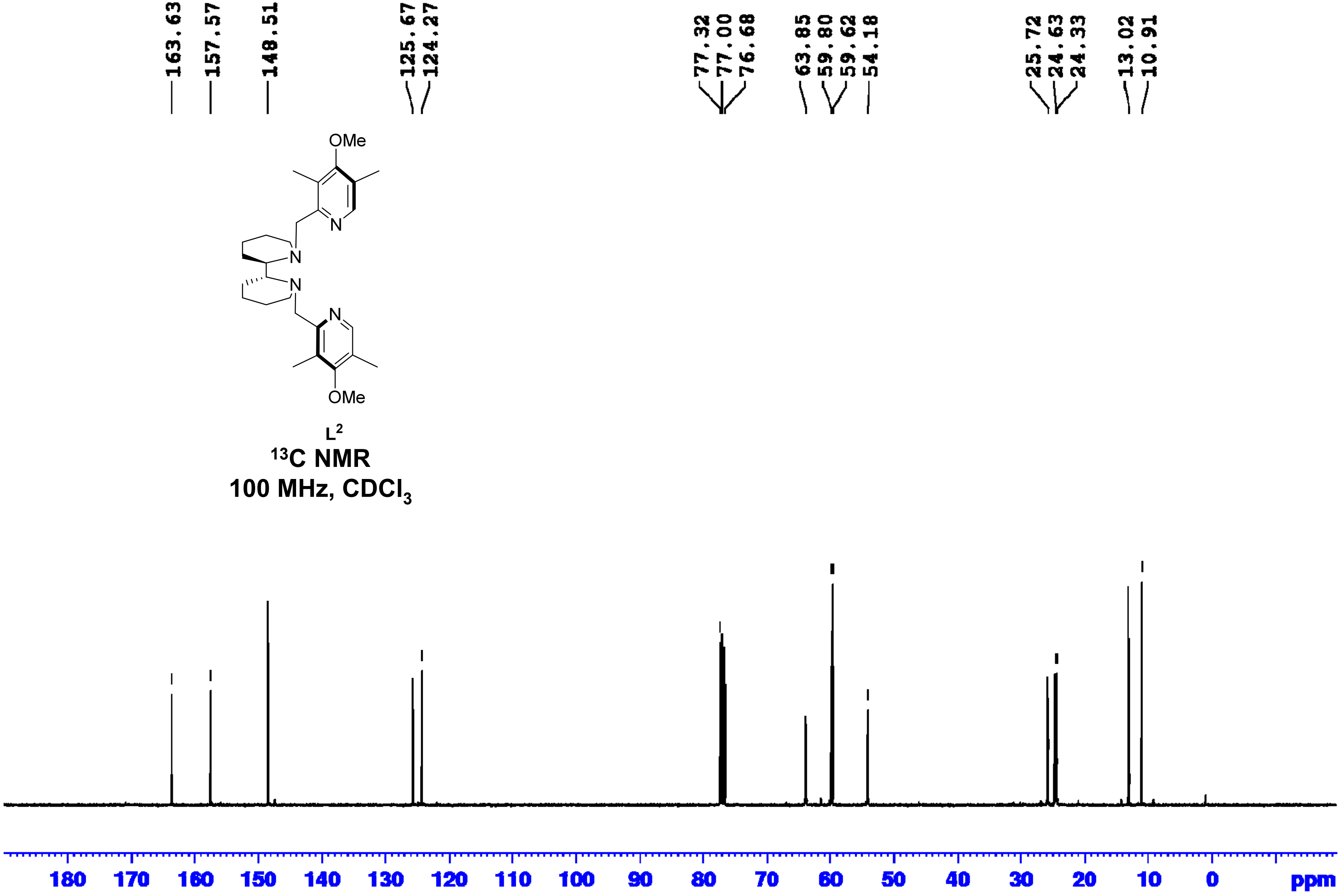
163.63
157.57
148.51
125.67
124.27
77.32
77.00
76.68
63.85
59.80
59.62
54.18
25.72
24.63
24.33
13.02
10.91
OMe
N
N
N
N
OMe
L2
13C NMR
100 MHz, CDCl3
180
170
160
150
140
130
120
110
100
90
80
70
60
50
40
30
20
10
0
ppm

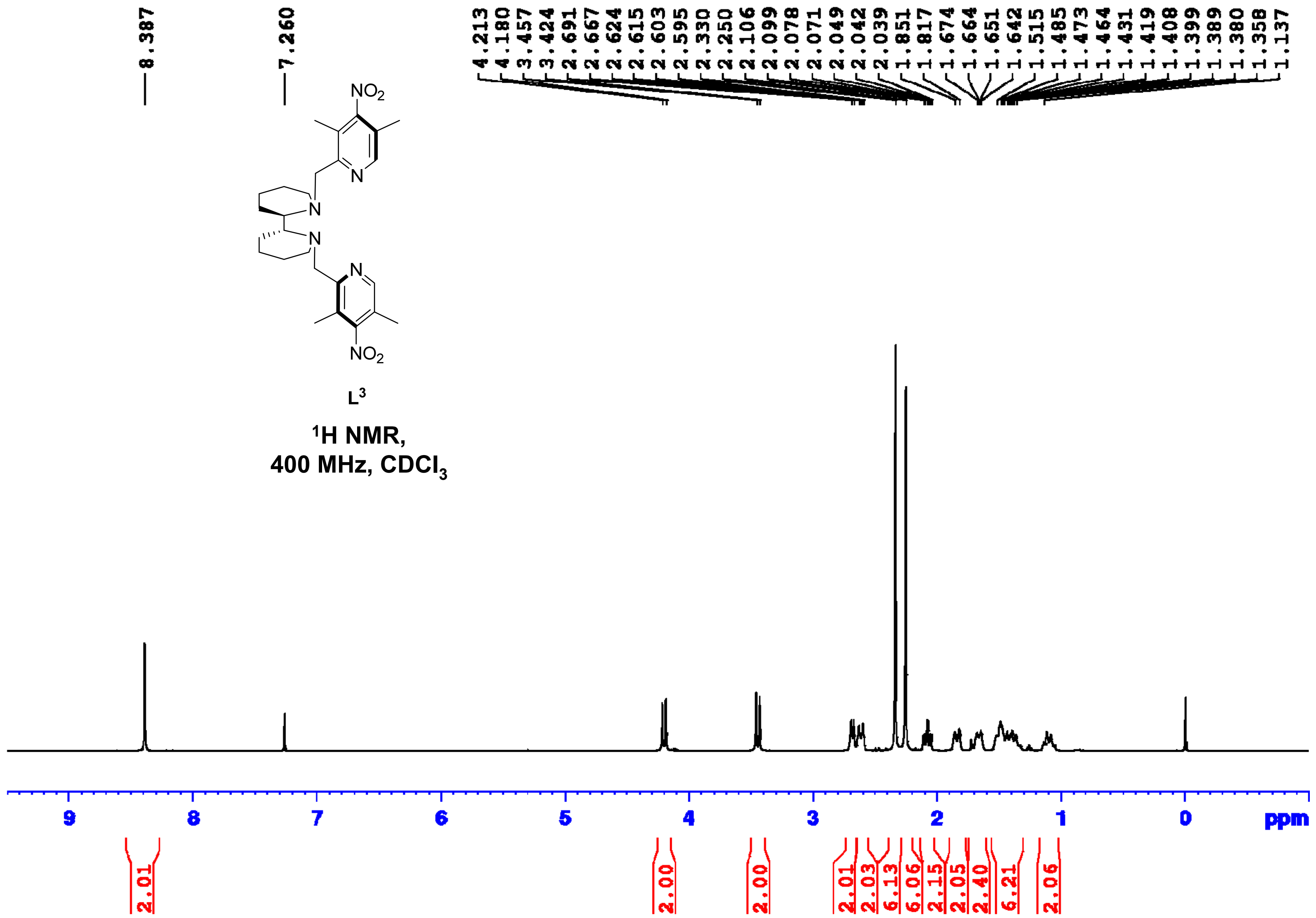
8.387
7.260
4.213
4.180
3.457
3.424
2.691
2.667
2.624
2.615
2.603
2.595
2.330
2.250
2.106
2.099
2.078
2.071
2.049
2.042
2.039
1.851
1.817
1.674
1.664
1.651
1.642
1.515
1.485
1.473
1.464
1.431
1.419
1.408
1.399
1.389
1.380
1.358
1.137
NO2
N
N
N
N
NO2
L3
1H NMR,
400 MHz, CDCl3
9
8
7
6
5
4
3
2
1
0
ppm
2.01
2.00
2.00
2.01
2.03
6.13
6.06
2.15
2.05
2.40
6.21
2.06

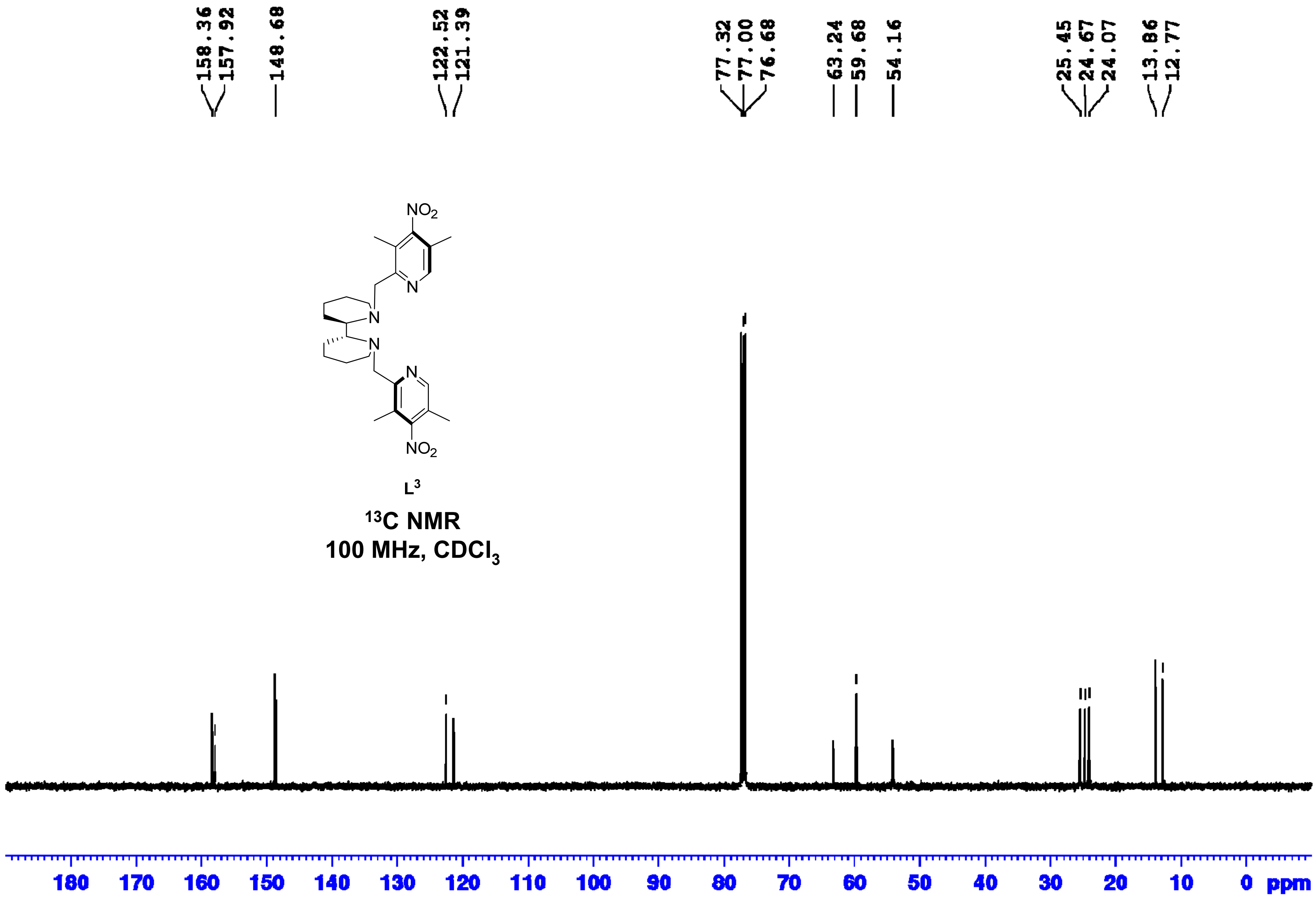
158.36
157.92
148.68
122.52
121.39
77.32
77.00
76.68
63.24
59.68
54.16
25.45
24.67
24.07
13.86
12.77
NO2
N
N
N
N
NO2
L3
13C NMR
100 MHz, CDCl3
180
170
160
150
140
130
120
110
100
90
80
70
60
50
40
30
20
10
0
ppm

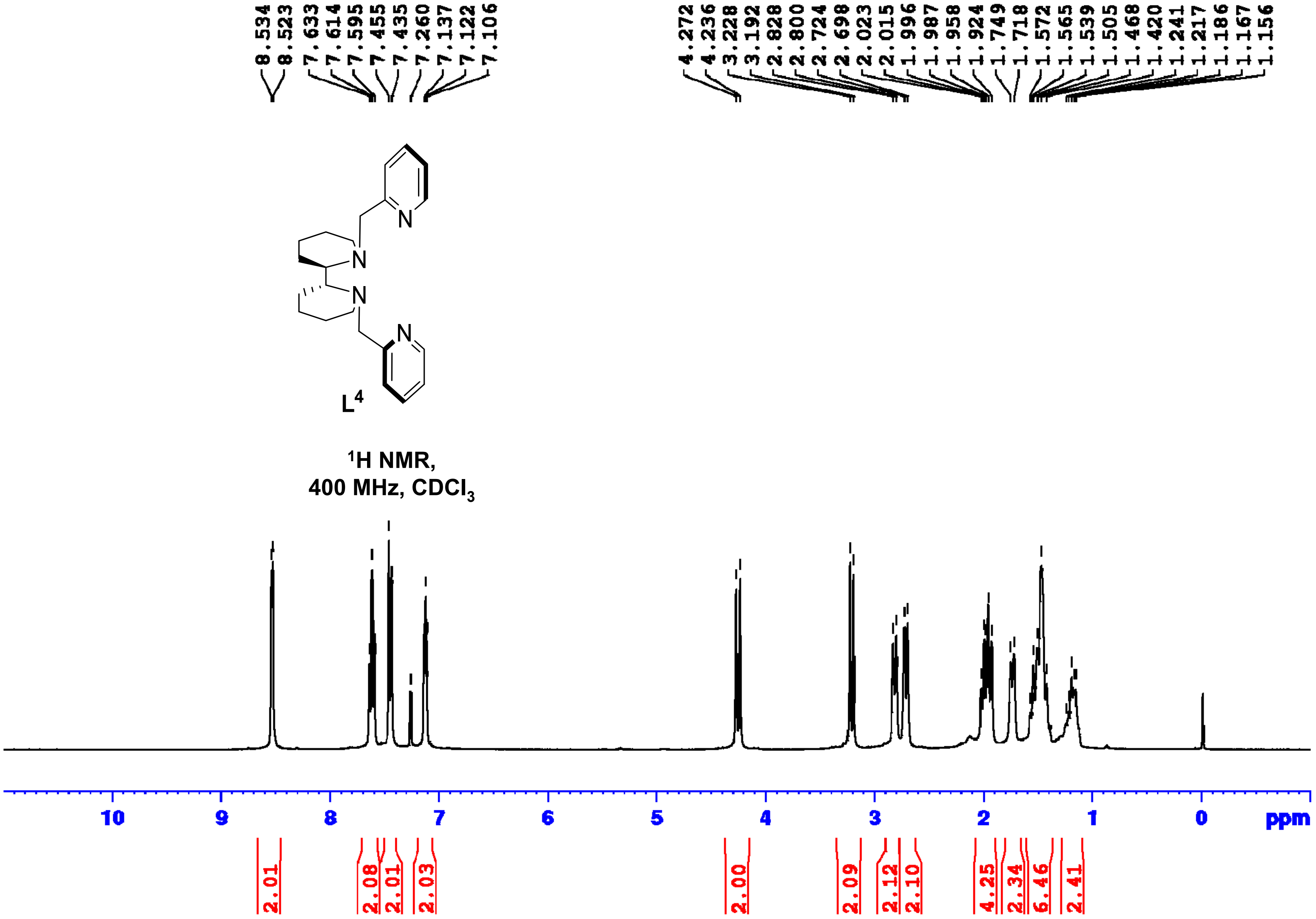
8.534 8.523 7.633 7.614 7.595 7.455 7.435 7.260 7.137 7.122 7.106
4.272 4.236 3.228 3.192 2.828 2.800 2.724 2.698 2.023 2.015 1.996 1.987 1.958 1.924 1.749 1.718 1.572 1.565 1.539 1.505 1.468 1.420 1.241 1.217 1.186 1.167 1.156
N
N
N
N
L4
1H NMR,
400 MHz, CDCl3
10 9 8 7 6 5 4 3 2 1 0 ppm
2.01 2.08 2.01 2.03 2.00 2.09 2.12 2.10 4.25 2.34 6.46 2.41

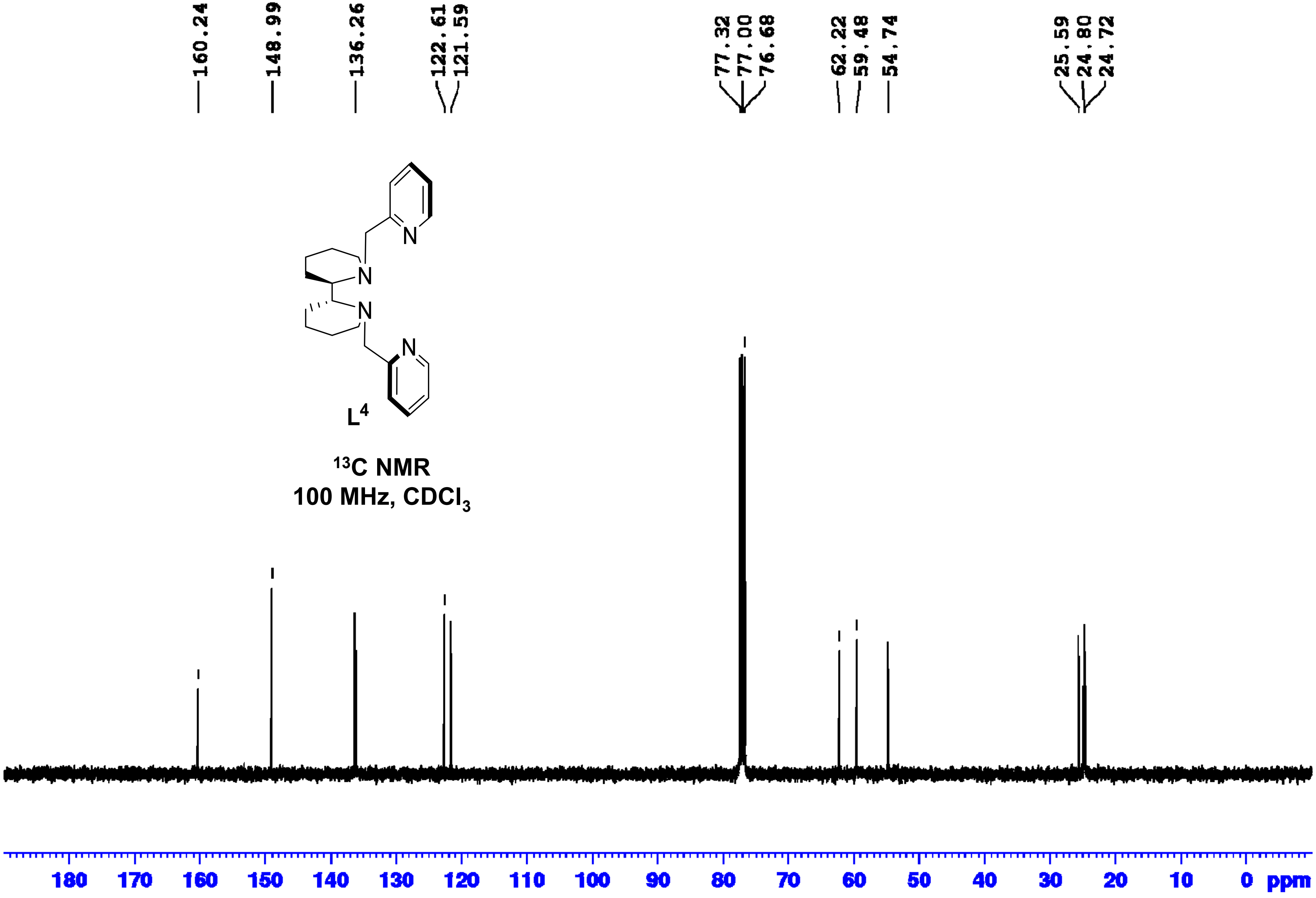
160.24
148.99
136.26
122.61
121.59
77.32
77.00
76.68
62.22
59.48
54.74
25.59
24.80
24.72
N
N
N
N
L4
13C NMR
100 MHz, CDCl3
180 170 160 150 140 130 120 110 100 90 80 70 60 50 40 30 20 10 0 ppm

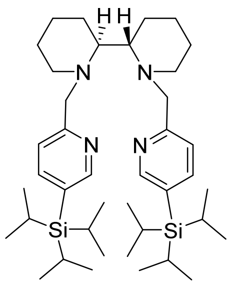

L[5]

^{1}H NMR, 400 MHz, $CDCl_3$

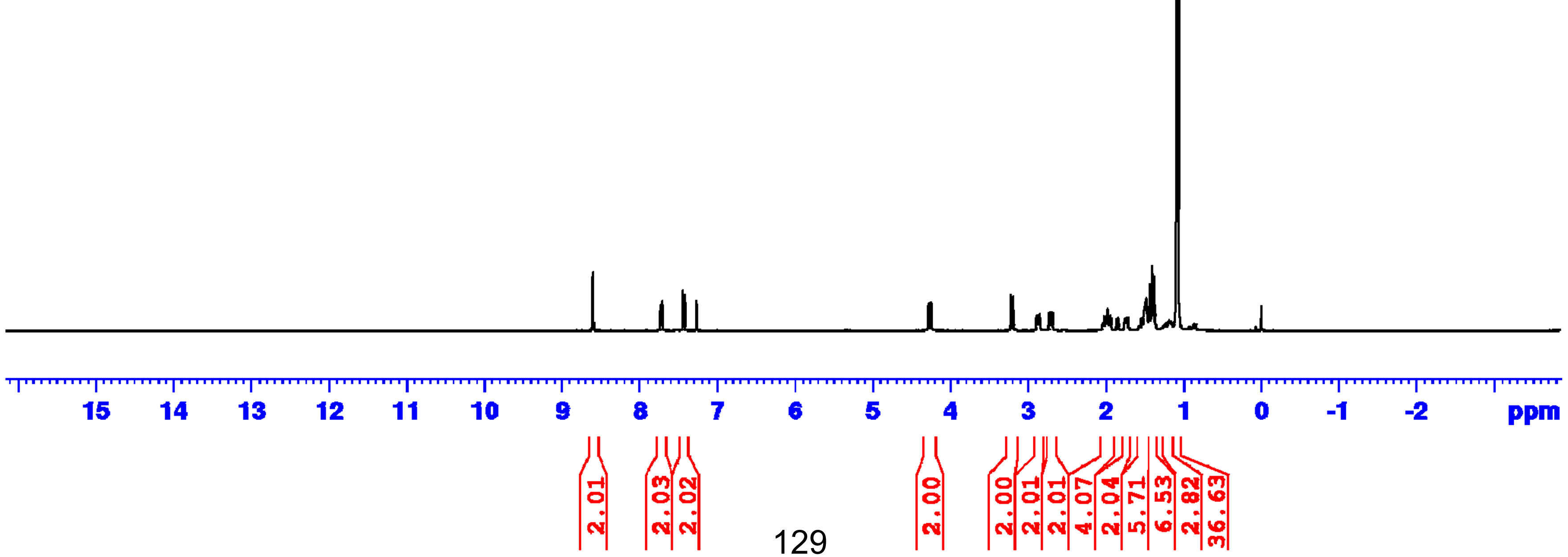

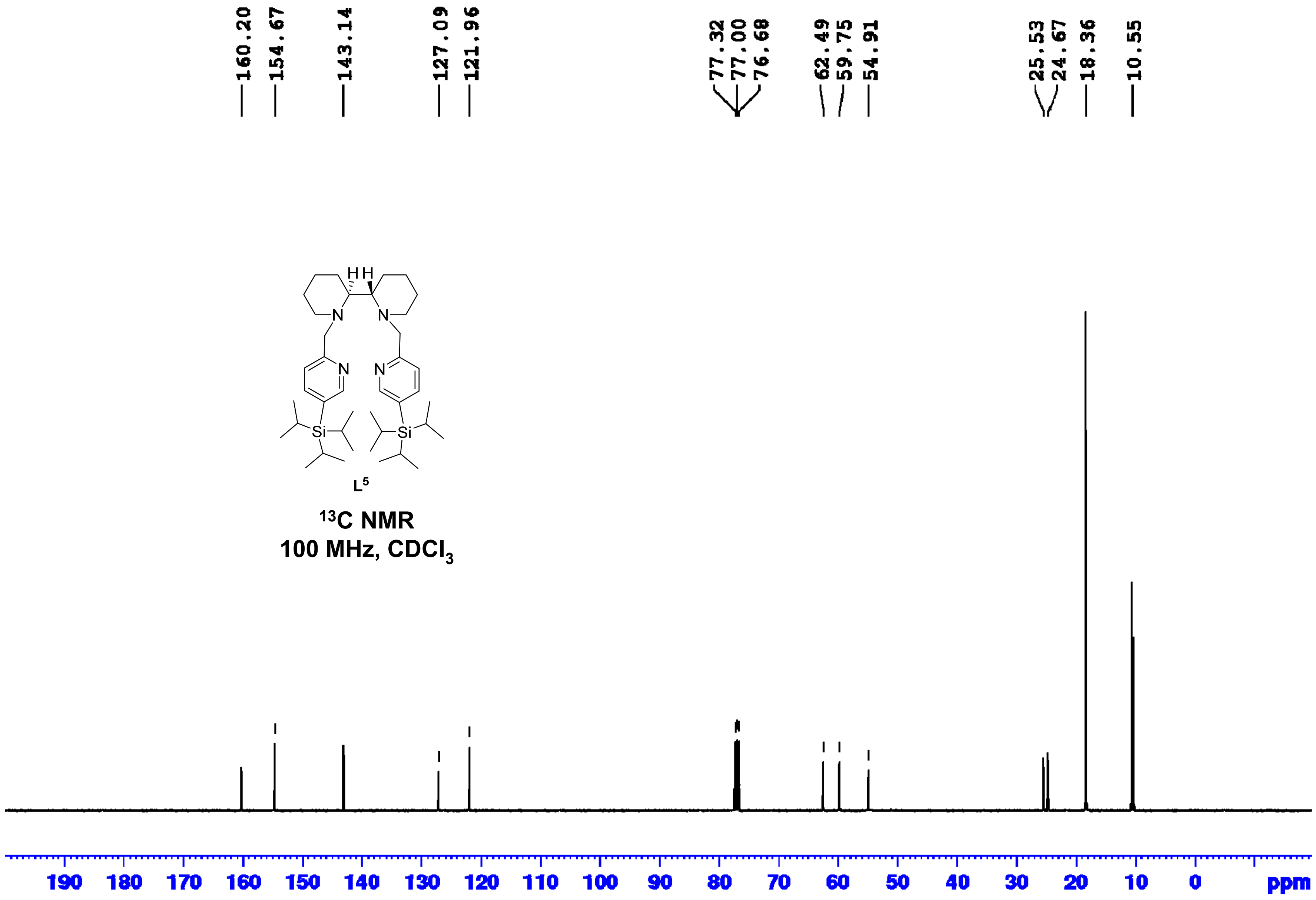
160.20
154.67
143.14
127.09
121.96
77.32
77.00
76.68
62.49
59.75
54.91
25.53
24.67
18.36
10.55
H H
N
N
N
N
Si
Si
L5
13C NMR
100 MHz, CDCl3
190 180 170 160 150 140 130 120 110 100 90 80 70 60 50 40 30 20 10 0 ppm

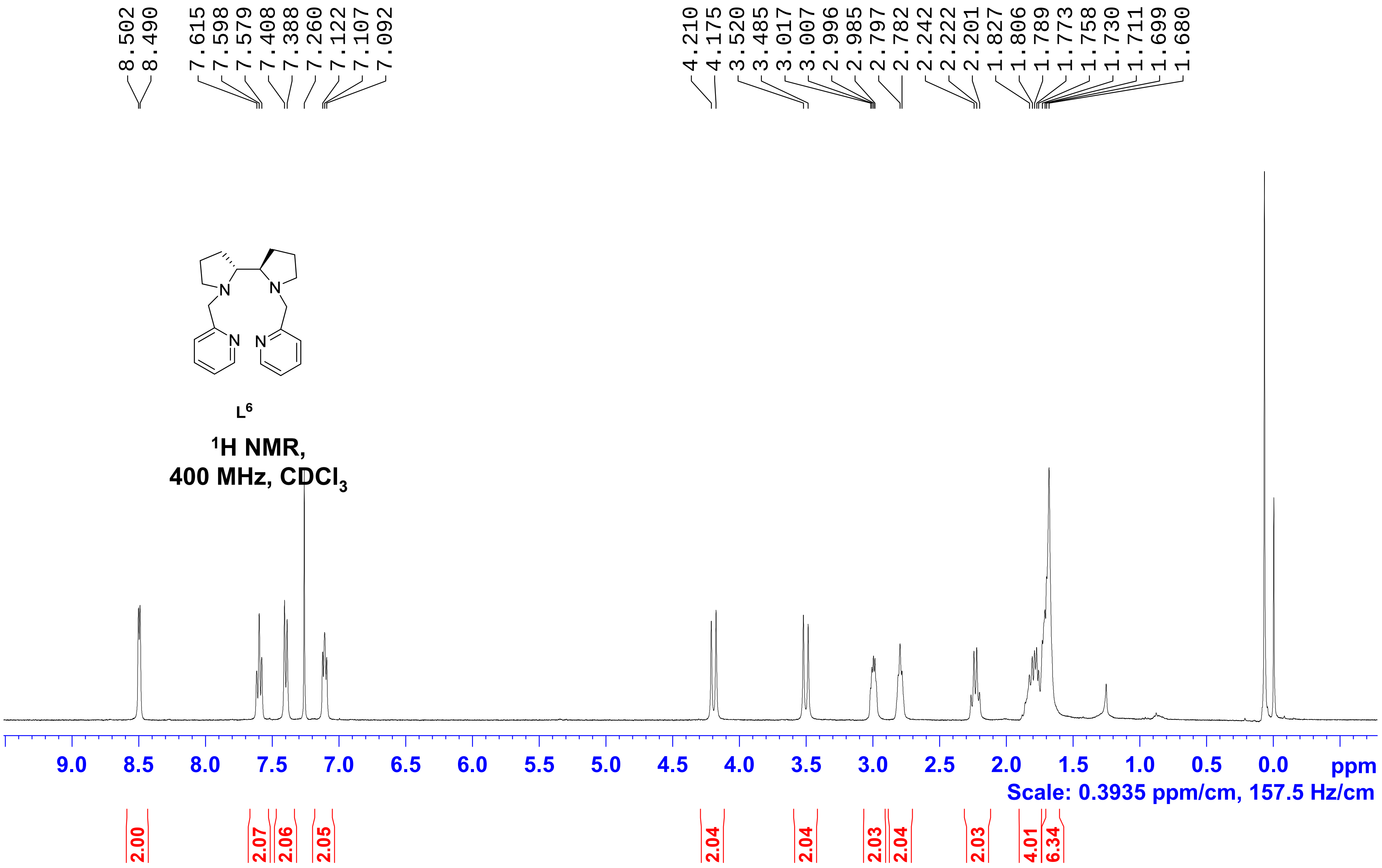
8.502
8.490
7.615
7.598
7.579
7.408
7.388
7.260
7.122
7.107
7.092
4.210
4.175
3.520
3.485
3.017
3.007
2.996
2.985
2.797
2.782
2.242
2.222
2.201
1.827
1.806
1.789
1.773
1.758
1.730
1.711
1.699
1.680
N
N
N
N
L6
1H NMR,
400 MHz, CDCl3
9.0 8.5 8.0 7.5 7.0 6.5 6.0 5.5 5.0 4.5 4.0 3.5 3.0 2.5 2.0 1.5 1.0 0.5 0.0 ppm
Scale: 0.3935 ppm/cm, 157.5 Hz/cm
2.00
2.07
2.06
2.05
2.04
2.04
2.03
2.04
2.03
4.01
6.34

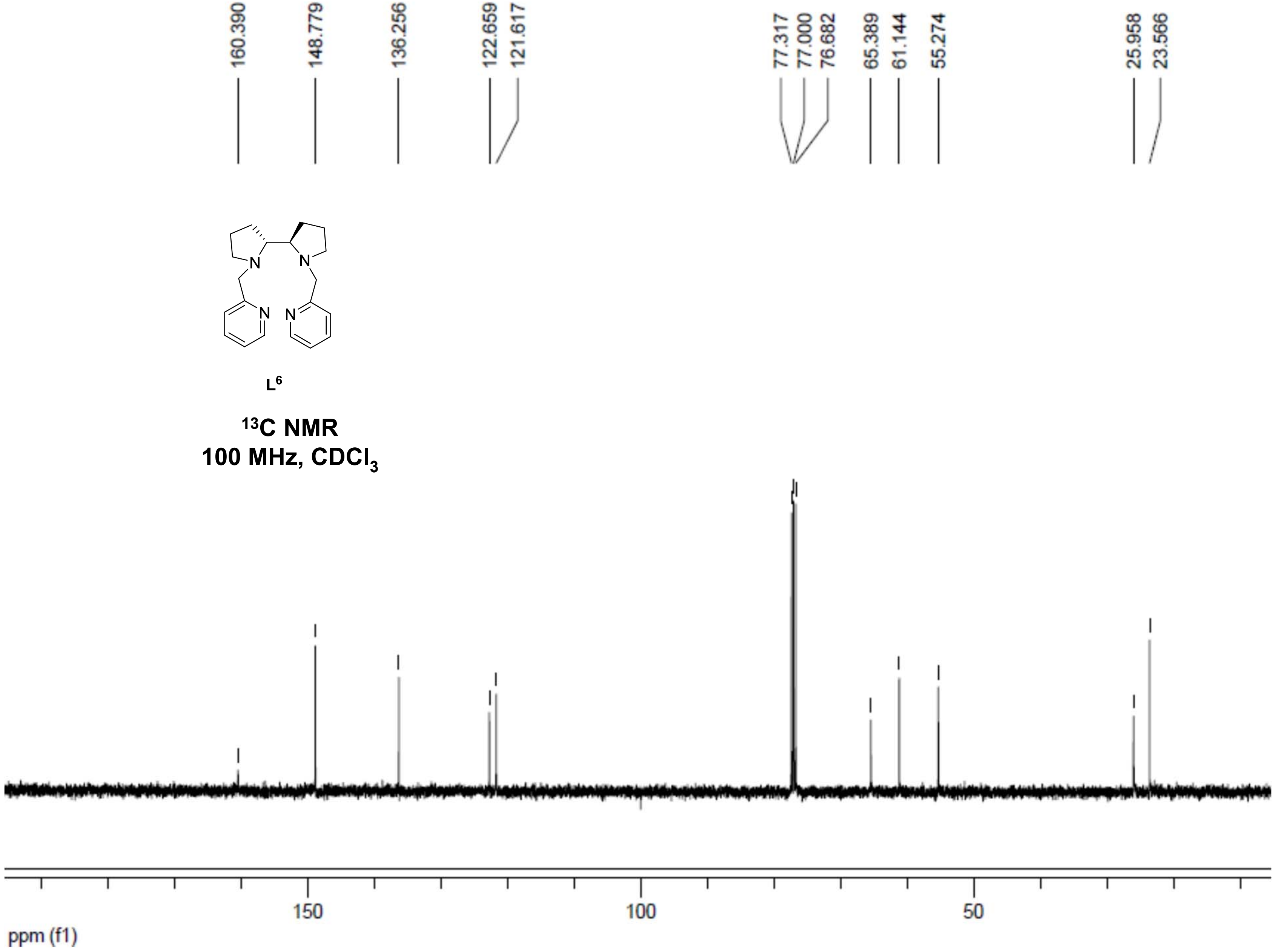
160.390
148.779
136.256
122.659
121.617
77.317
77.000
76.682
65.389
61.144
55.274
25.958
23.566
L6
13C NMR
100 MHz, CDCl3
150
100
50
ppm (f1)

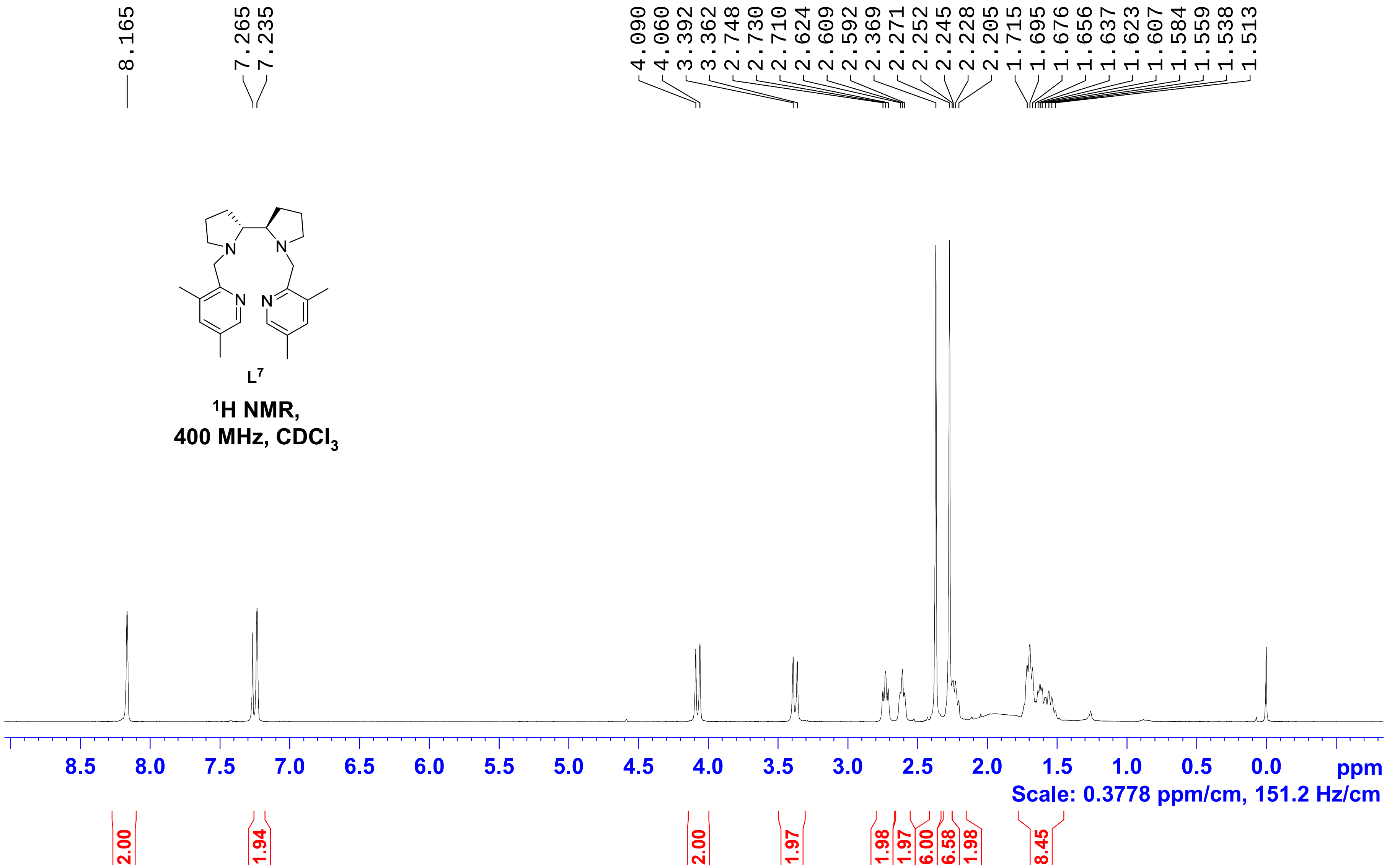
8.165
7.265
7.235
4.090
4.060
3.392
3.362
2.748
2.730
2.710
2.624
2.609
2.592
2.369
2.271
2.252
2.245
2.228
2.205
1.715
1.695
1.676
1.656
1.637
1.623
1.607
1.584
1.559
1.538
1.513
L7
1H NMR,
400 MHz, CDCl3
8.5 8.0 7.5 7.0 6.5 6.0 5.5 5.0 4.5 4.0 3.5 3.0 2.5 2.0 1.5 1.0 0.5 0.0 ppm
Scale: 0.3778 ppm/cm, 151.2 Hz/cm
2.00
1.94
2.00
1.97
1.98
1.97
6.00
6.58
1.98
8.45

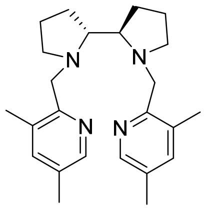

L[7]

^{13}C NMR
100 MHz, $CDCl_3$

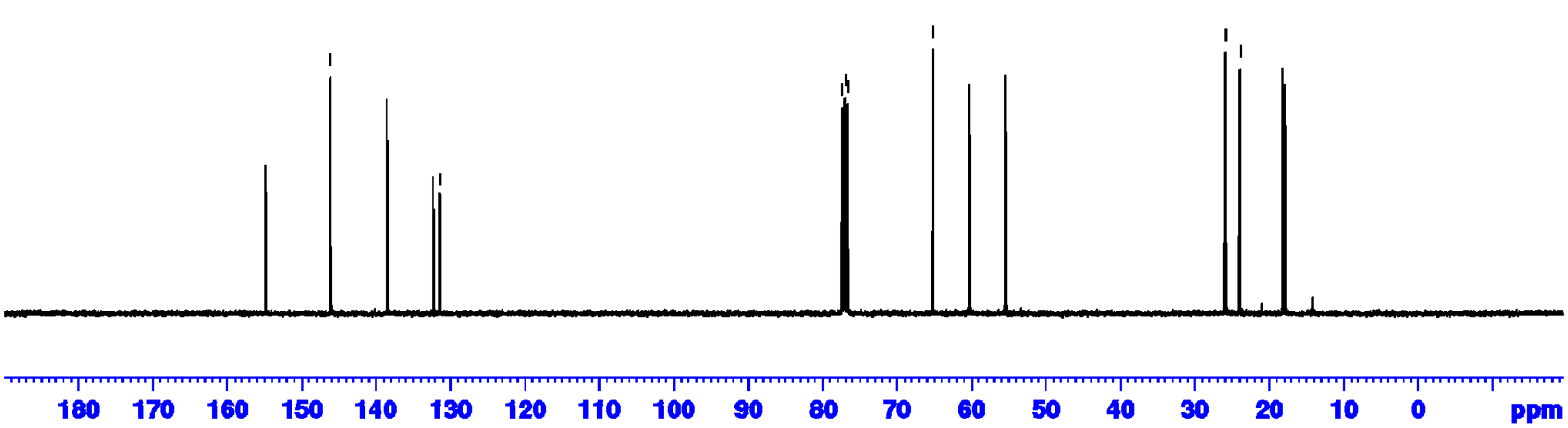

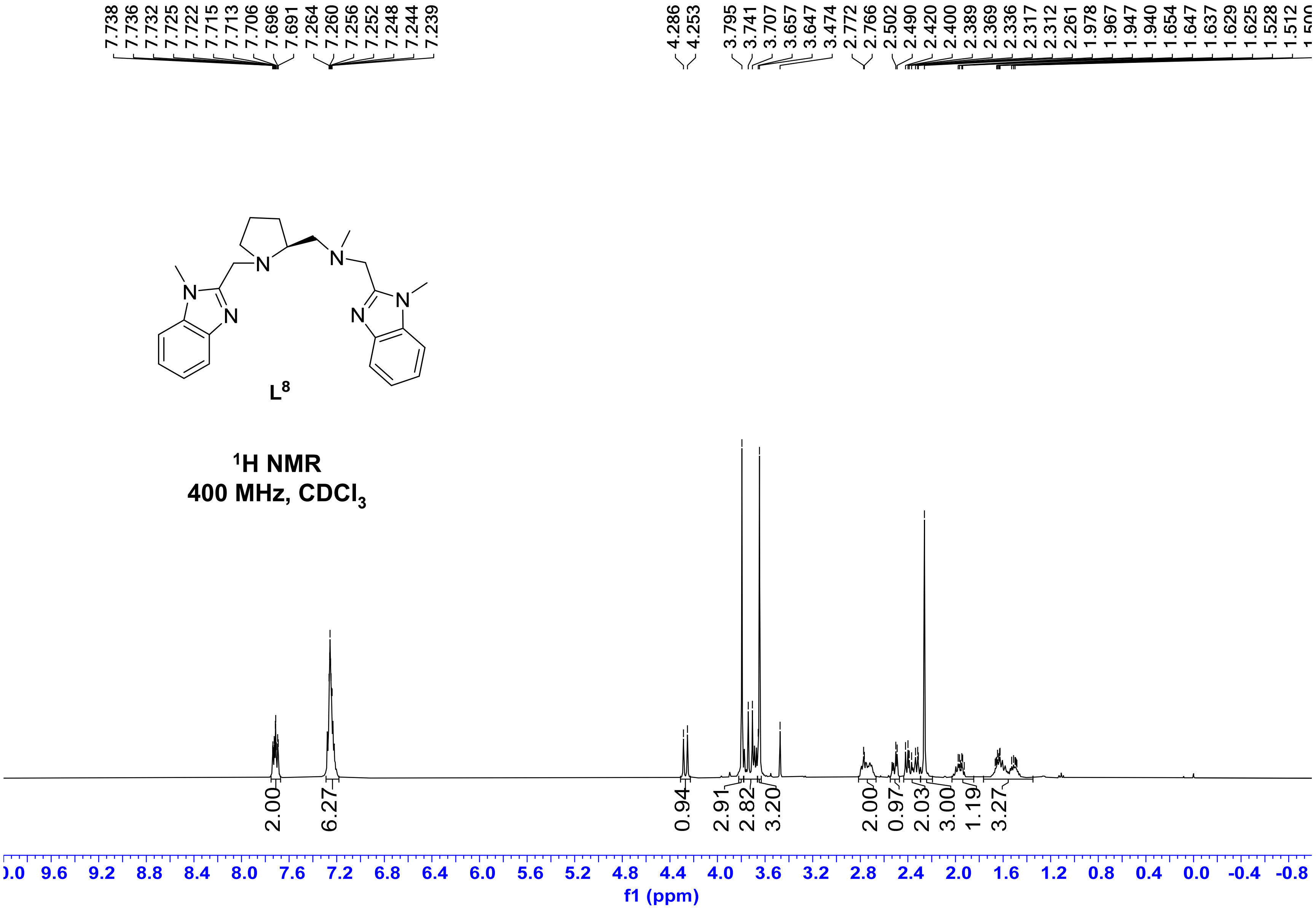
7.738
7.736
7.732
7.725
7.722
7.715
7.713
7.706
7.696
7.691
7.264
7.260
7.256
7.252
7.248
7.244
7.239
4.286
4.253
3.795
3.741
3.707
3.657
3.647
3.474
2.772
2.766
2.502
2.490
2.420
2.400
2.389
2.369
2.336
2.317
2.312
2.261
1.978
1.967
1.947
1.940
1.654
1.647
1.637
1.629
1.625
1.528
1.512
N
N
N
N
N
N
L8
1H NMR
400 MHz, CDCl3
2.00
6.27
0.94
2.91
2.82
3.20
2.00
0.97
2.03
3.00
1.19
3.27
9.6 9.2 8.8 8.4 8.0 7.6 7.2 6.8 6.4 6.0 5.6 5.2 4.8 4.4 4.0 3.6 3.2 2.8 2.4 2.0 1.6 1.2 0.8 0.4 0.0 -0.4 -0.8
f1 (ppm)

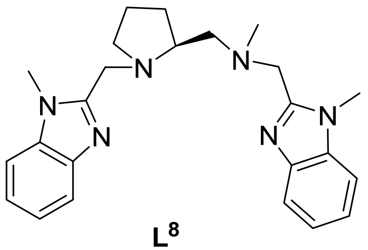

L[8]

^{13}C NMR
100 MHz, $CDCl_3$

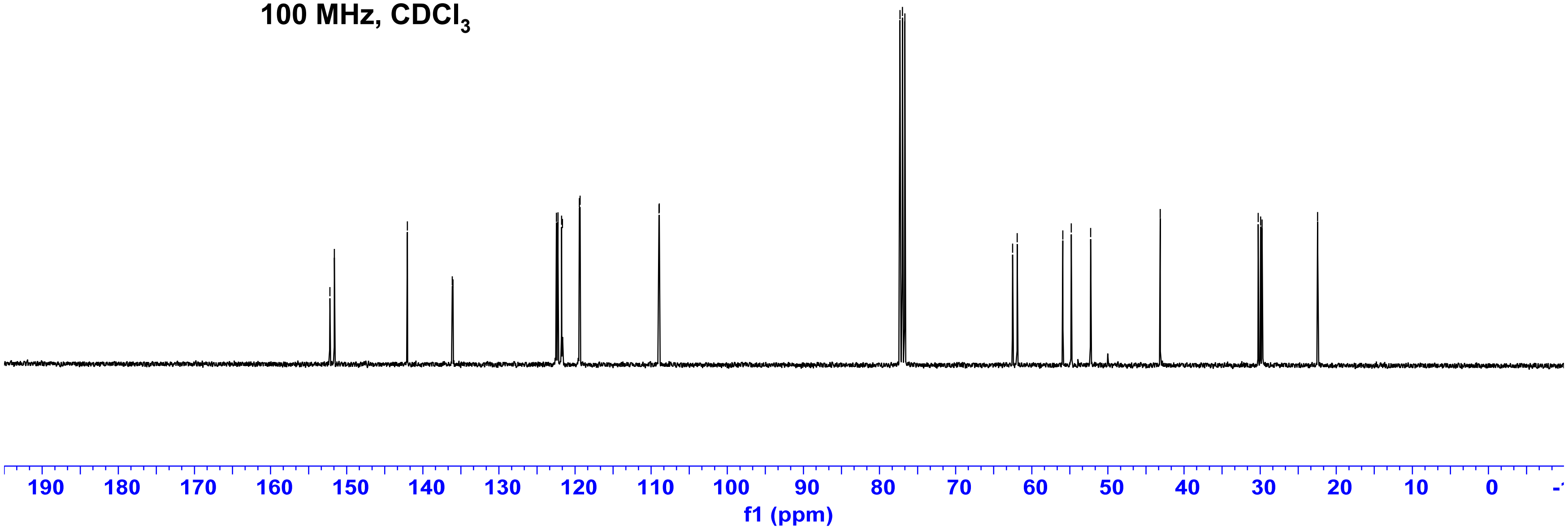

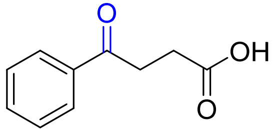

2a

^{1}H NMR,
400 MHz, $CDCl_3$

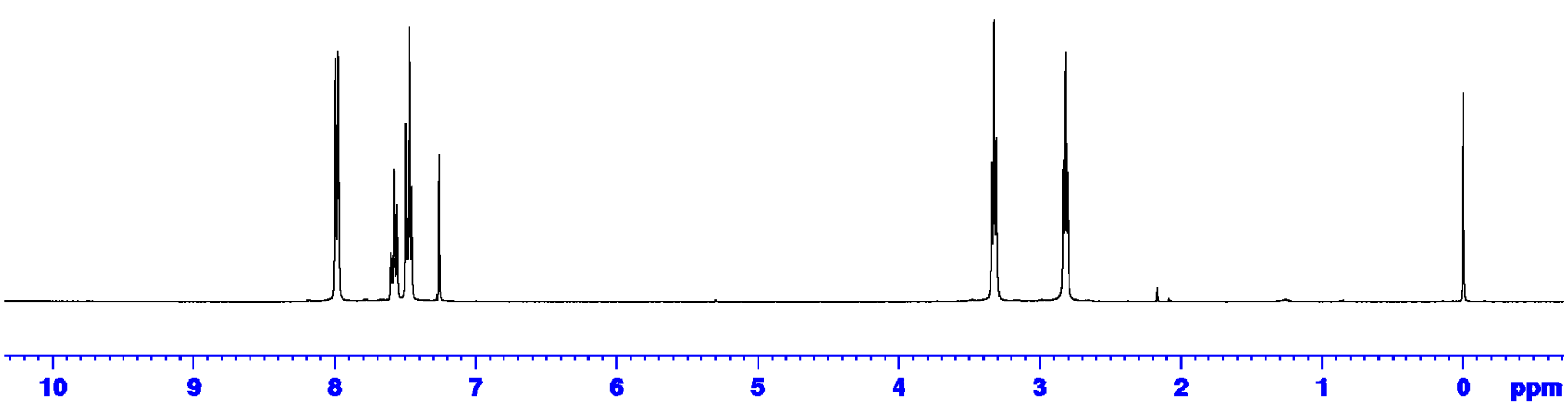

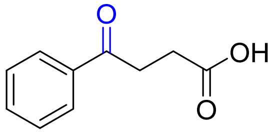

2a

^{13}C NMR
100 MHz, $CDCl_3$

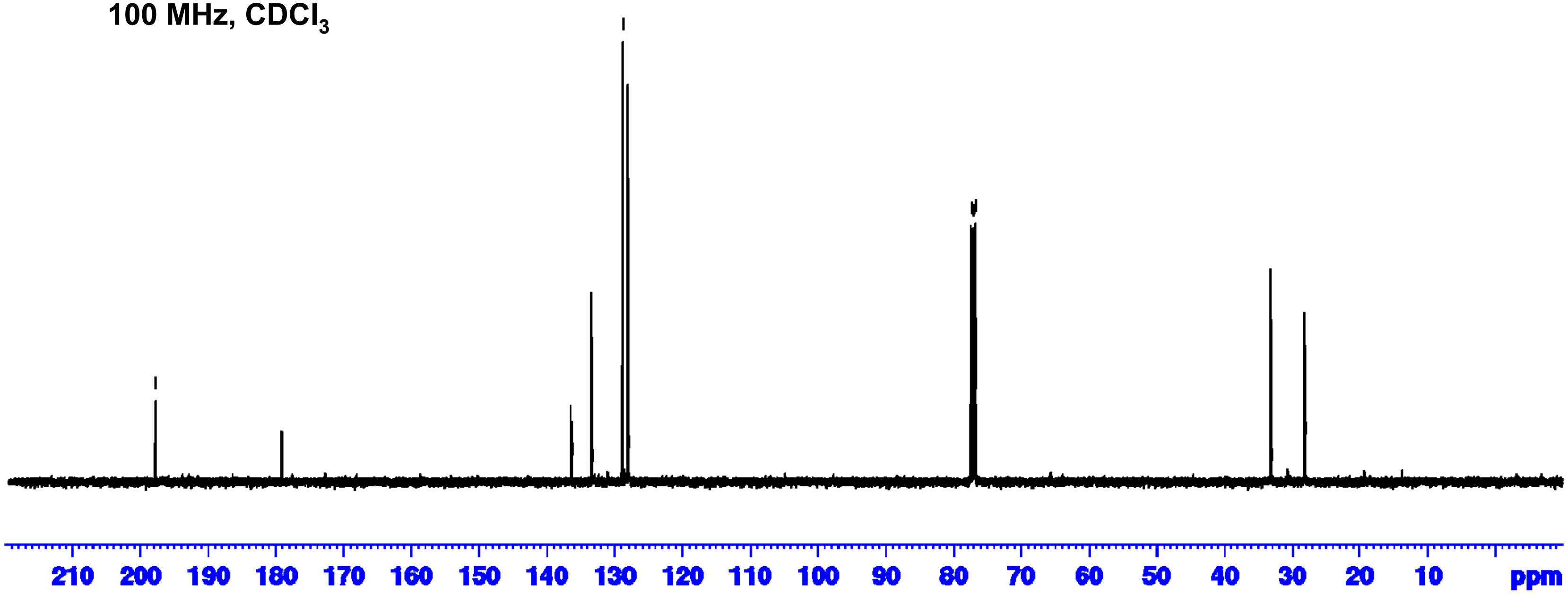

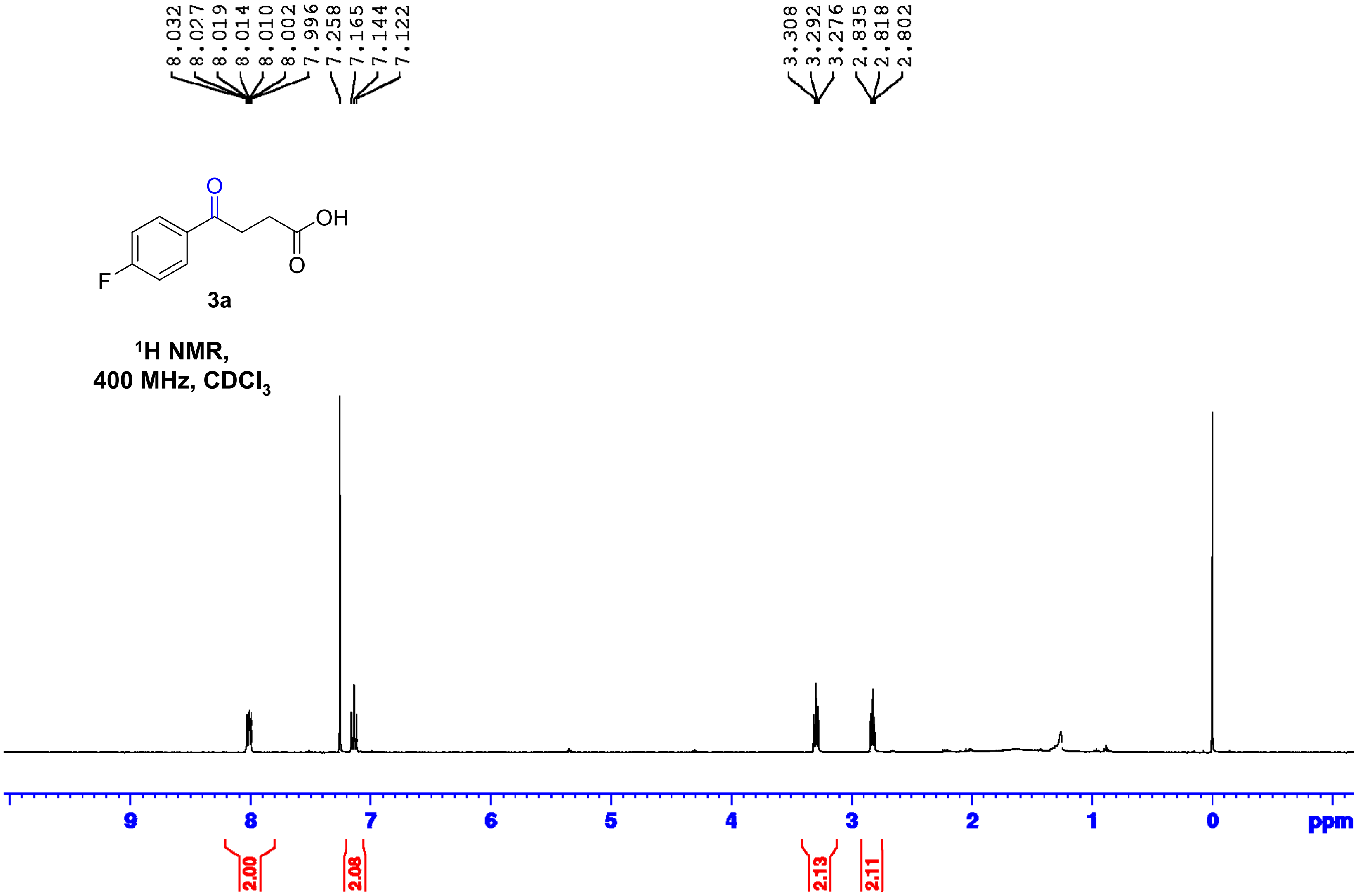
8.032
8.027
8.019
8.014
8.010
8.002
7.996
7.258
7.165
7.144
7.122
3.308
3.292
3.276
2.835
2.818
2.802
O
OH
F
O
3a
1H NMR,
400 MHz, CDCl3
9
8
7
6
5
4
3
2
1
0
ppm
2.00
2.08
2.13
2.11

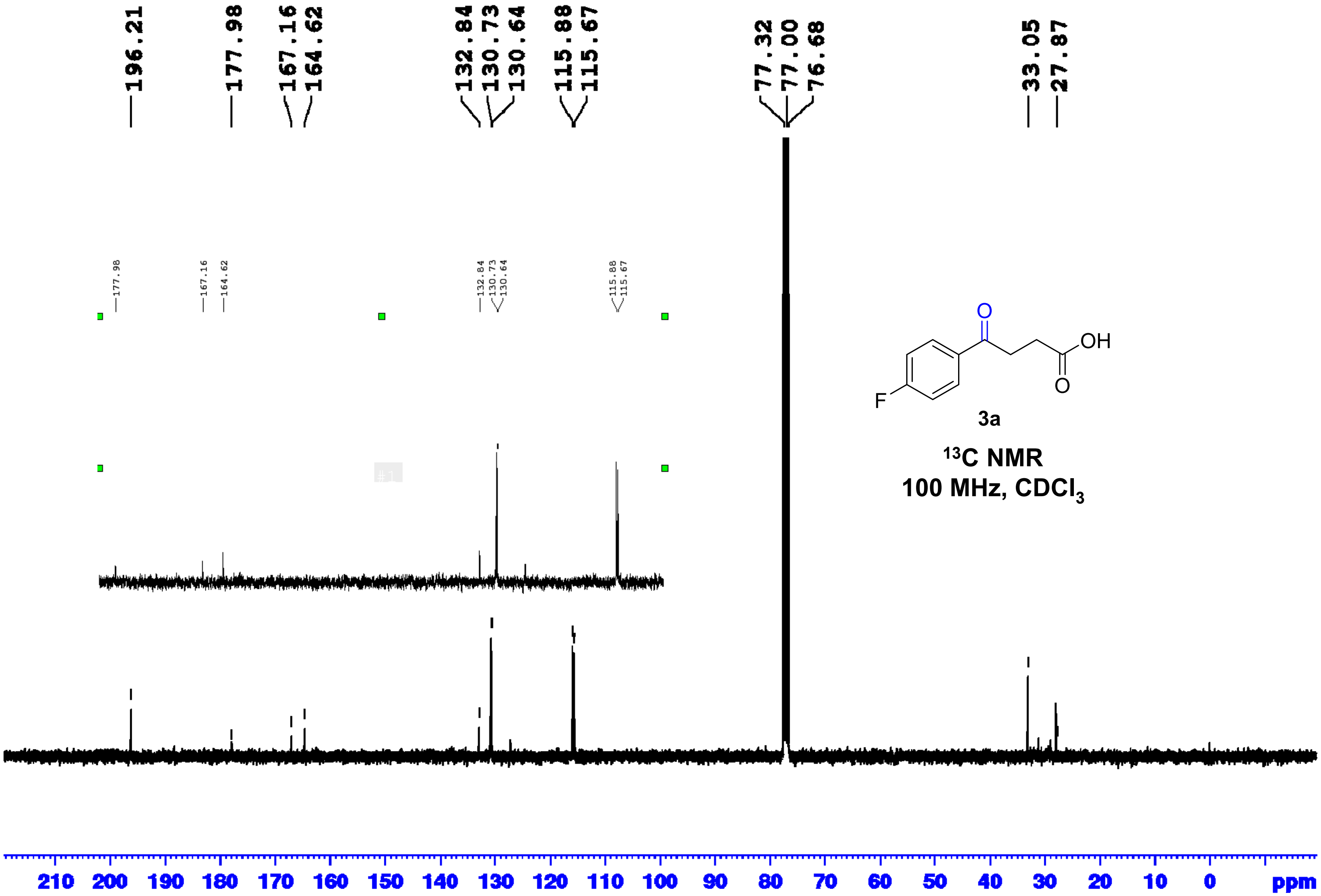

3a

^{13}C NMR
100 MHz, $CDCl_3$

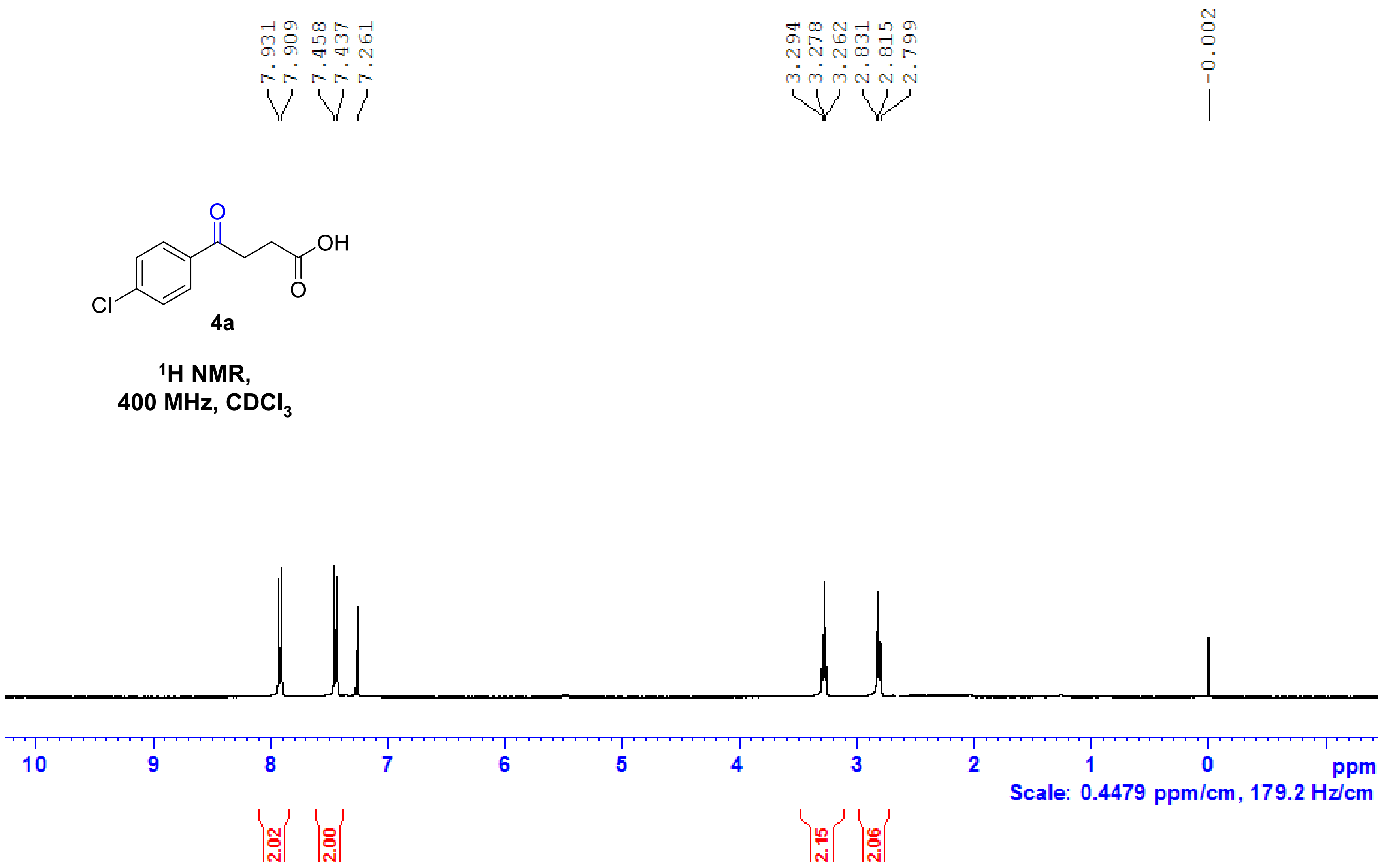
7.931
7.909
7.458
7.437
7.261
3.294
3.278
3.262
2.831
2.815
2.799
-0.002
O
OH
O
Cl
4a
1H NMR,
400 MHz, CDCl3
10
9
8
7
6
5
4
3
2
1
0
ppm
Scale: 0.4479 ppm/cm, 179.2 Hz/cm
2.02
2.00
2.15
2.06

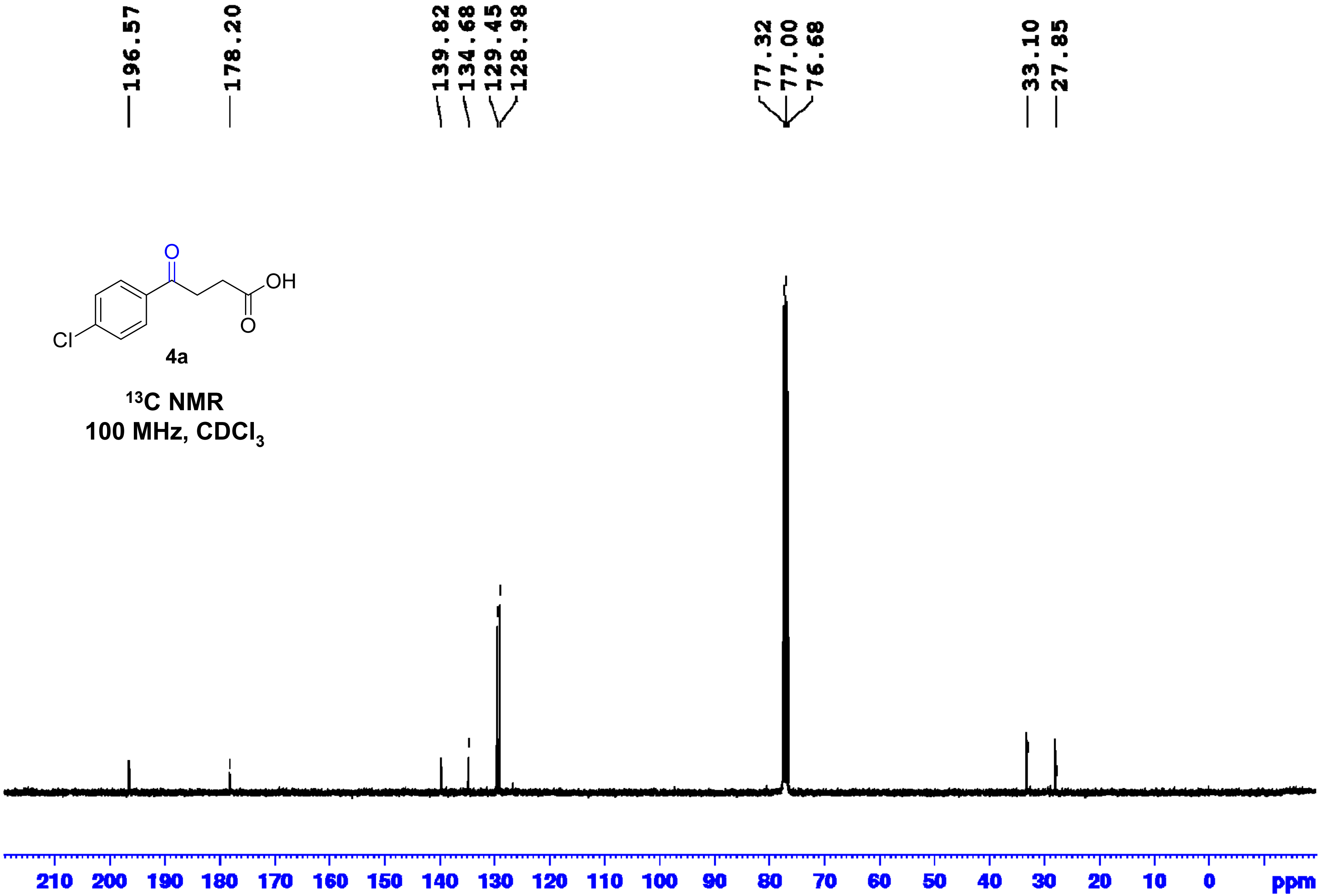
196.57
178.20
139.82
134.68
129.45
128.98
77.32
77.00
76.68
33.10
27.85
O
OH
O
Cl
4a
^{13}C NMR
100 MHz, $CDCl_3$
210 200 190 180 170 160 150 140 130 120 110 100 90 80 70 60 50 40 30 20 10 0 ppm

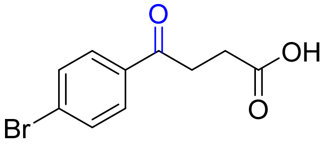

5a

^{1}H NMR,
400 MHz, $CDCl_3$

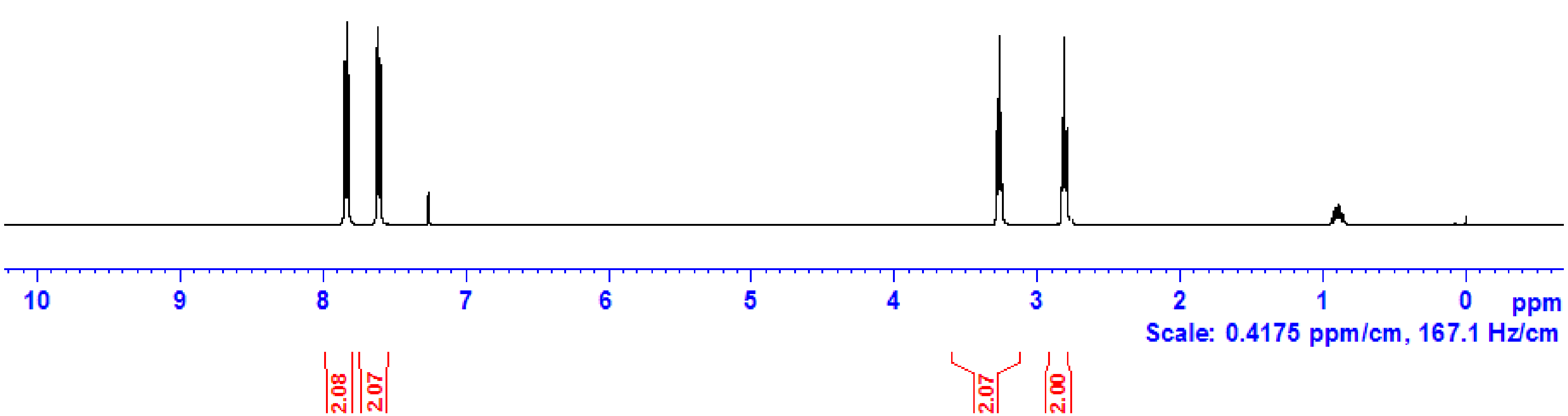

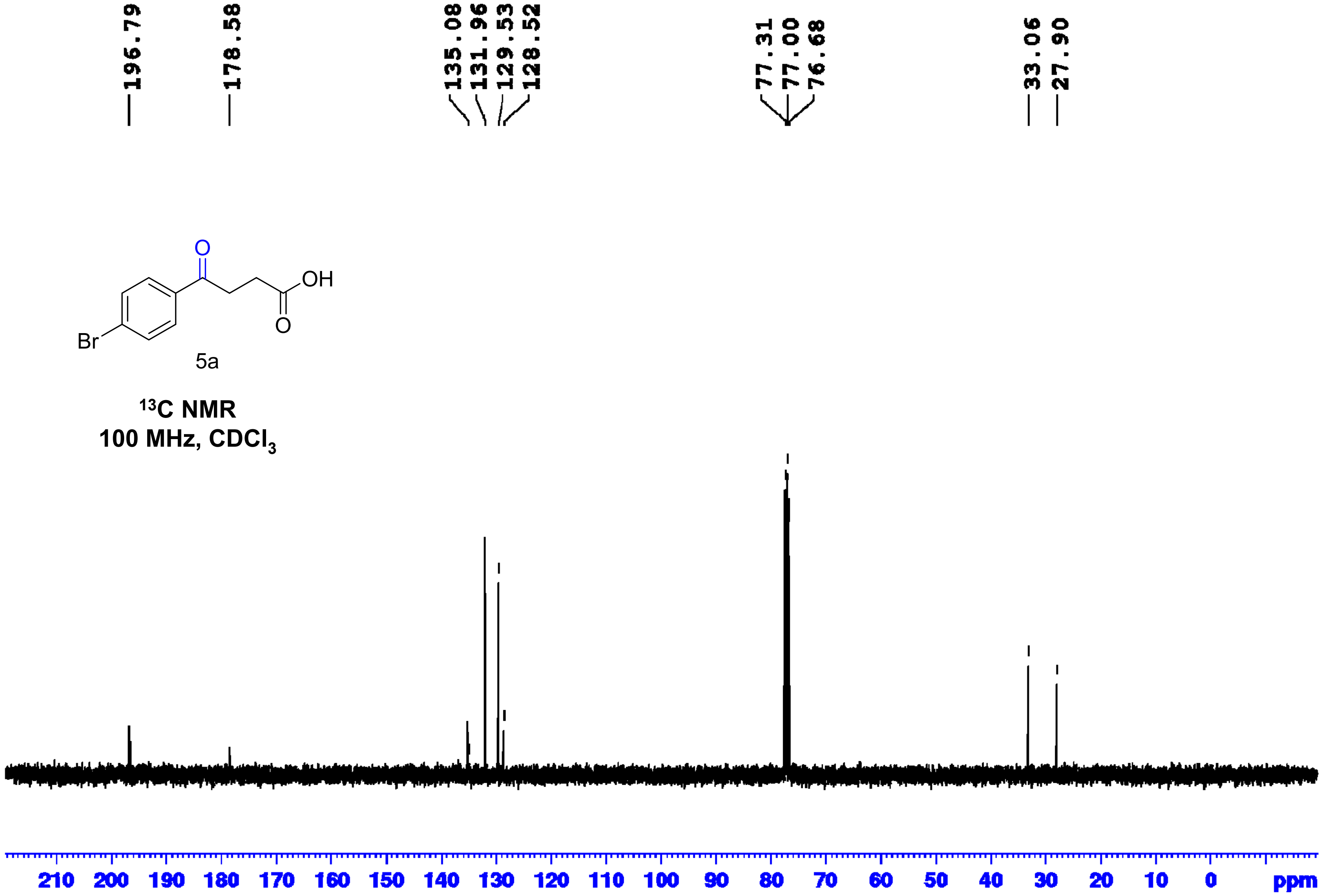

5a

^{13}C NMR
100 MHz, $CDCl_3$

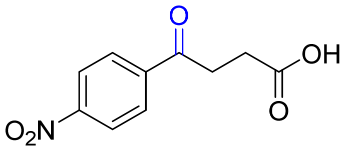

6a

^{1}H NMR,
400 MHz, DMSO-d6

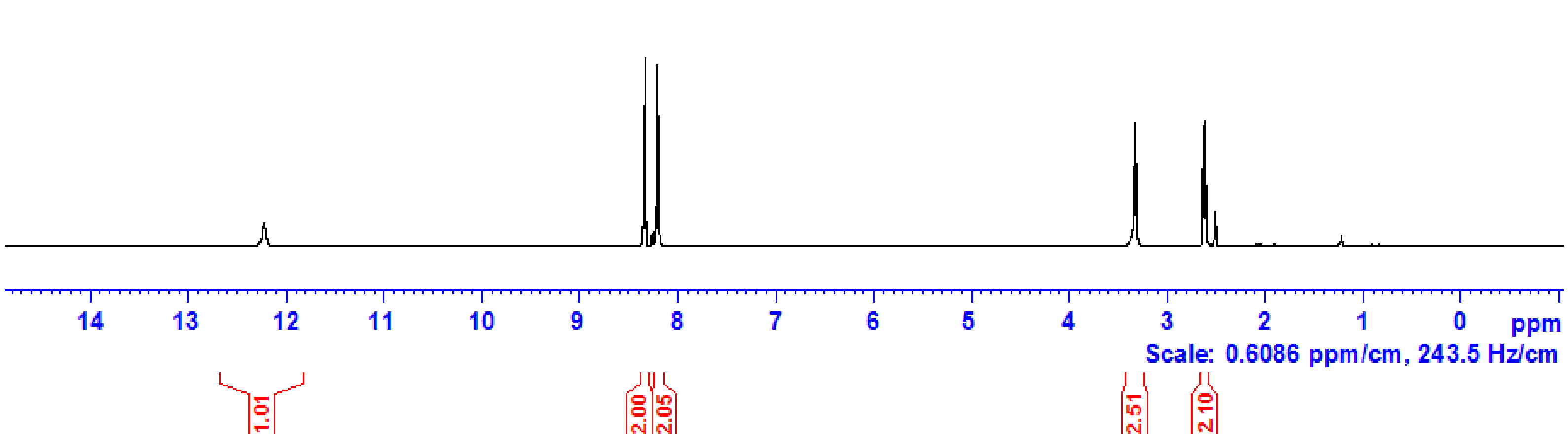

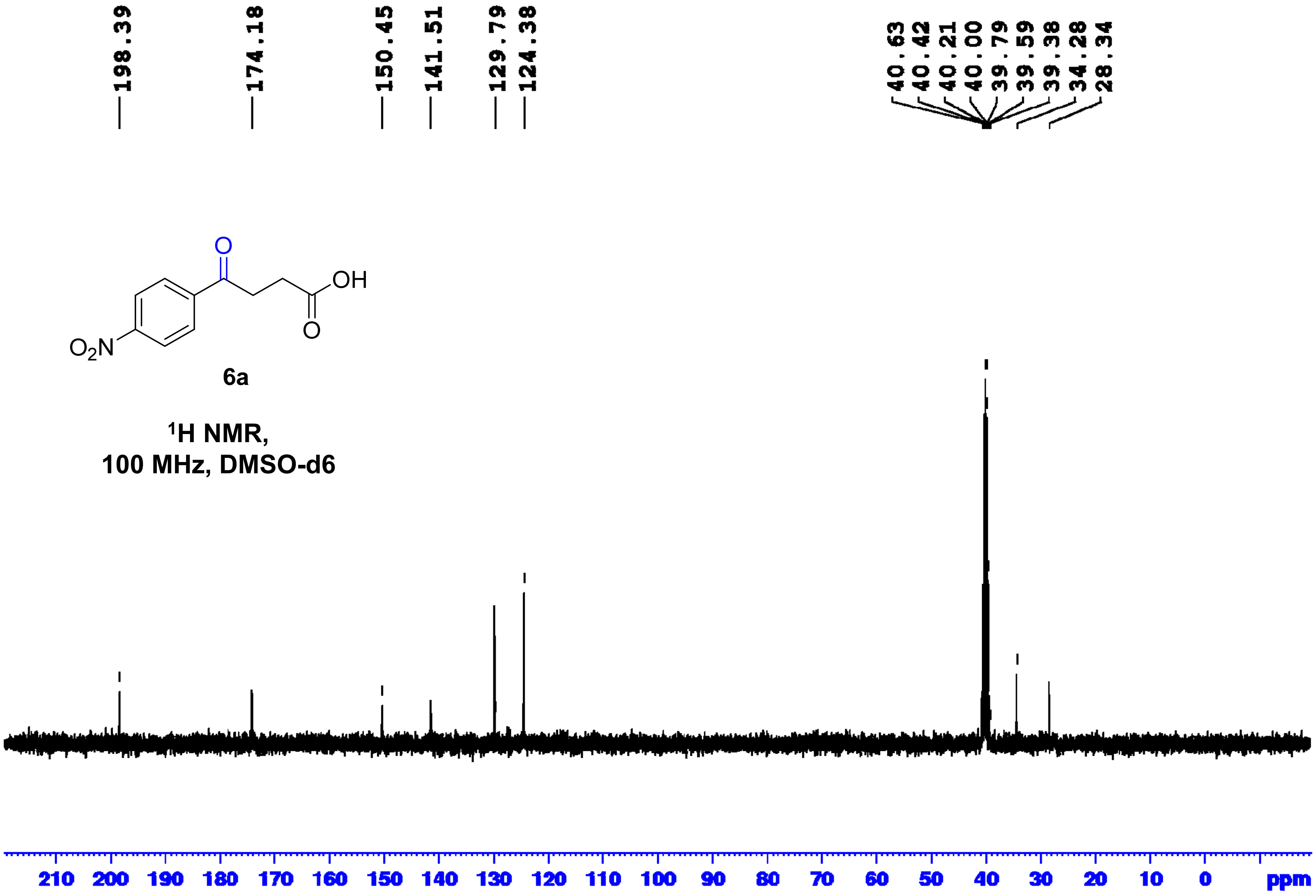
198.39
174.18
150.45
141.51
129.79
124.38
40.63
40.42
40.21
40.00
39.79
39.59
39.38
34.28
28.34
O
OH
O
O_2N
6a
^{1}H NMR,
100 MHz, DMSO-d6
210 200 190 180 170 160 150 140 130 120 110 100 90 80 70 60 50 40 30 20 10 0 ppm

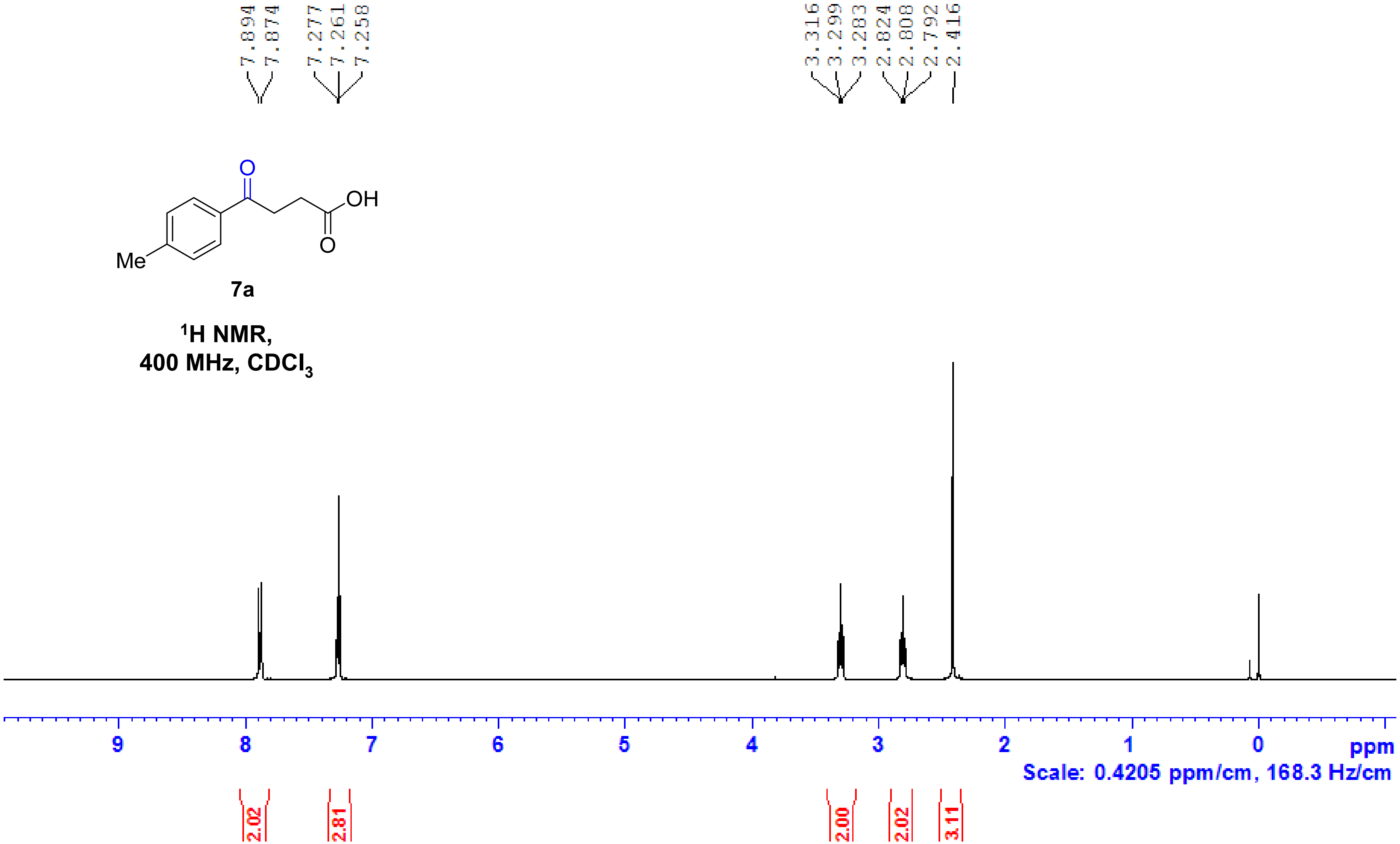
7.894
7.874
7.277
7.261
7.258
3.316
3.299
3.283
2.824
2.808
2.792
2.416
O
OH
O
Me
7a
^{1}H NMR,
400 MHz, $CDCl_3$
9 8 7 6 5 4 3 2 1 0 ppm
Scale: 0.4205 ppm/cm, 168.3 Hz/cm
2.02
2.81
2.00
2.02
3.11

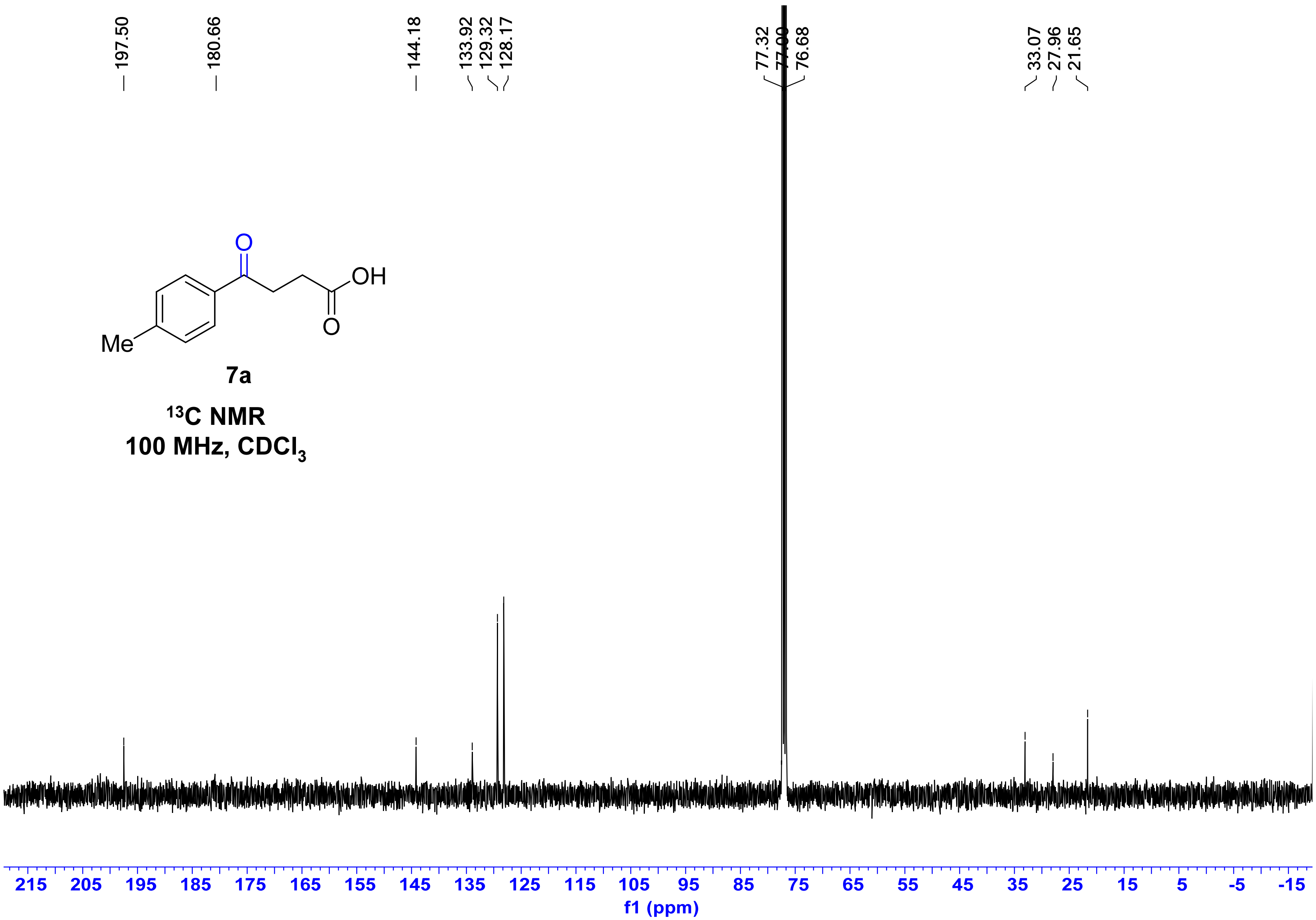
197.50
180.66
144.18
133.92
129.32
128.17
77.32
77.00
76.68
33.07
27.96
21.65
O
OH
O
Me
7a
13C NMR
100 MHz, CDCl3
215
205
195
185
175
165
155
145
135
125
115
105
95
85
75
65
55
45
35
25
15
5
-5
-15
f1 (ppm)

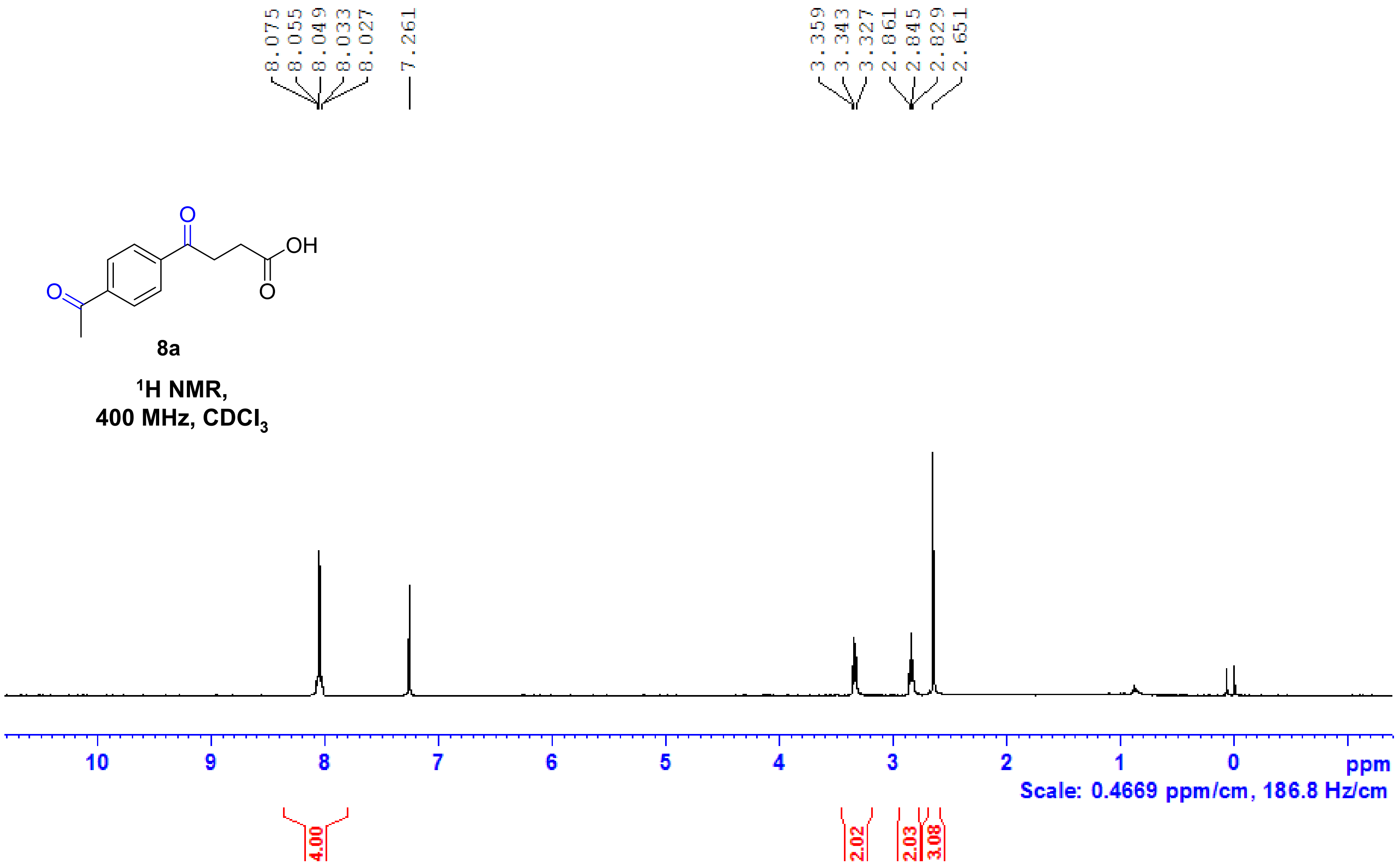
8.075
8.055
8.049
8.033
8.027
7.261
3.359
3.343
3.327
2.861
2.845
2.829
2.651
O
OH
O
O
8a
1H NMR,
400 MHz, CDCl3
10
9
8
7
6
5
4
3
2
1
0
ppm
Scale: 0.4669 ppm/cm, 186.8 Hz/cm
4.00
2.02
2.03
3.08

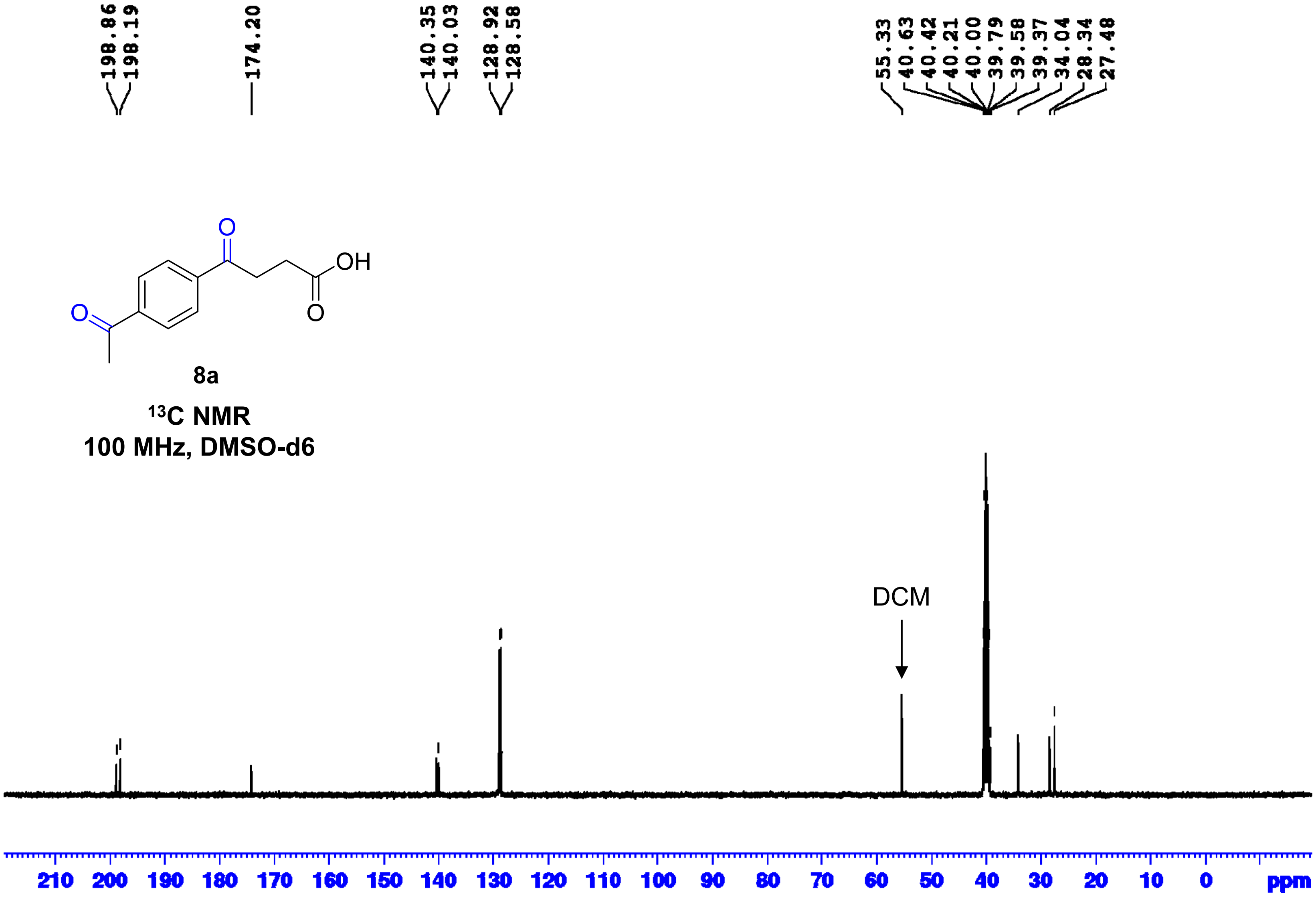
198.86
198.19
174.20
140.35
140.03
128.92
128.58
55.33
40.63
40.42
40.21
40.00
39.79
39.58
39.37
34.04
28.34
27.48
O
OH
O
O
8a
^{13}C NMR
100 MHz, DMSO-d6
DCM
210 200 190 180 170 160 150 140 130 120 110 100 90 80 70 60 50 40 30 20 10 0 ppm

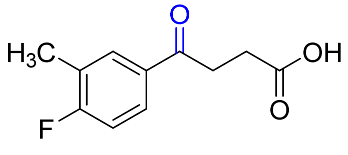

9a

^{1}H NMR,
400 MHz, $CDCl_3$

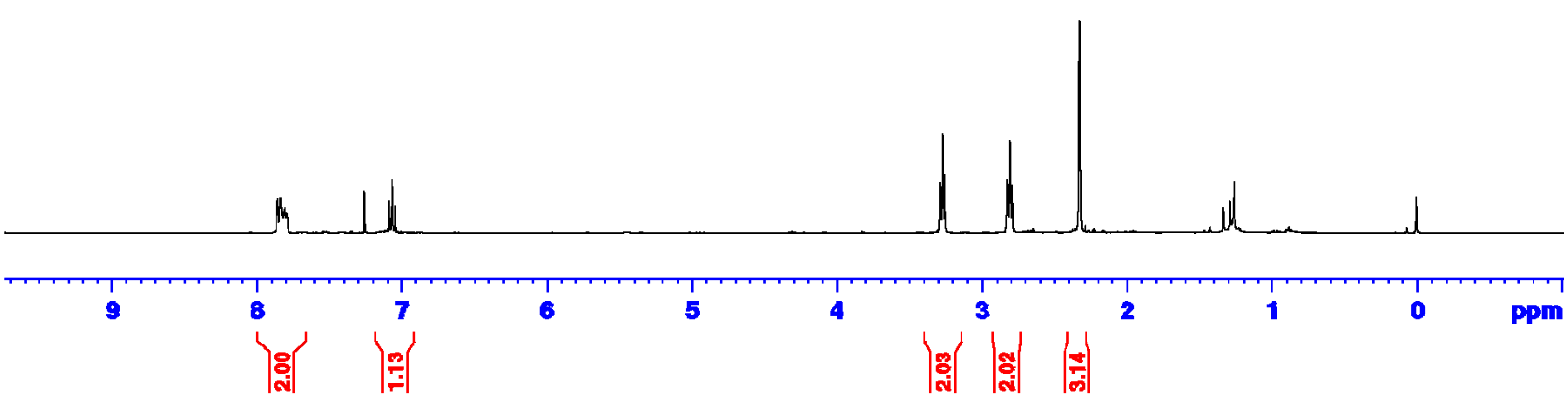

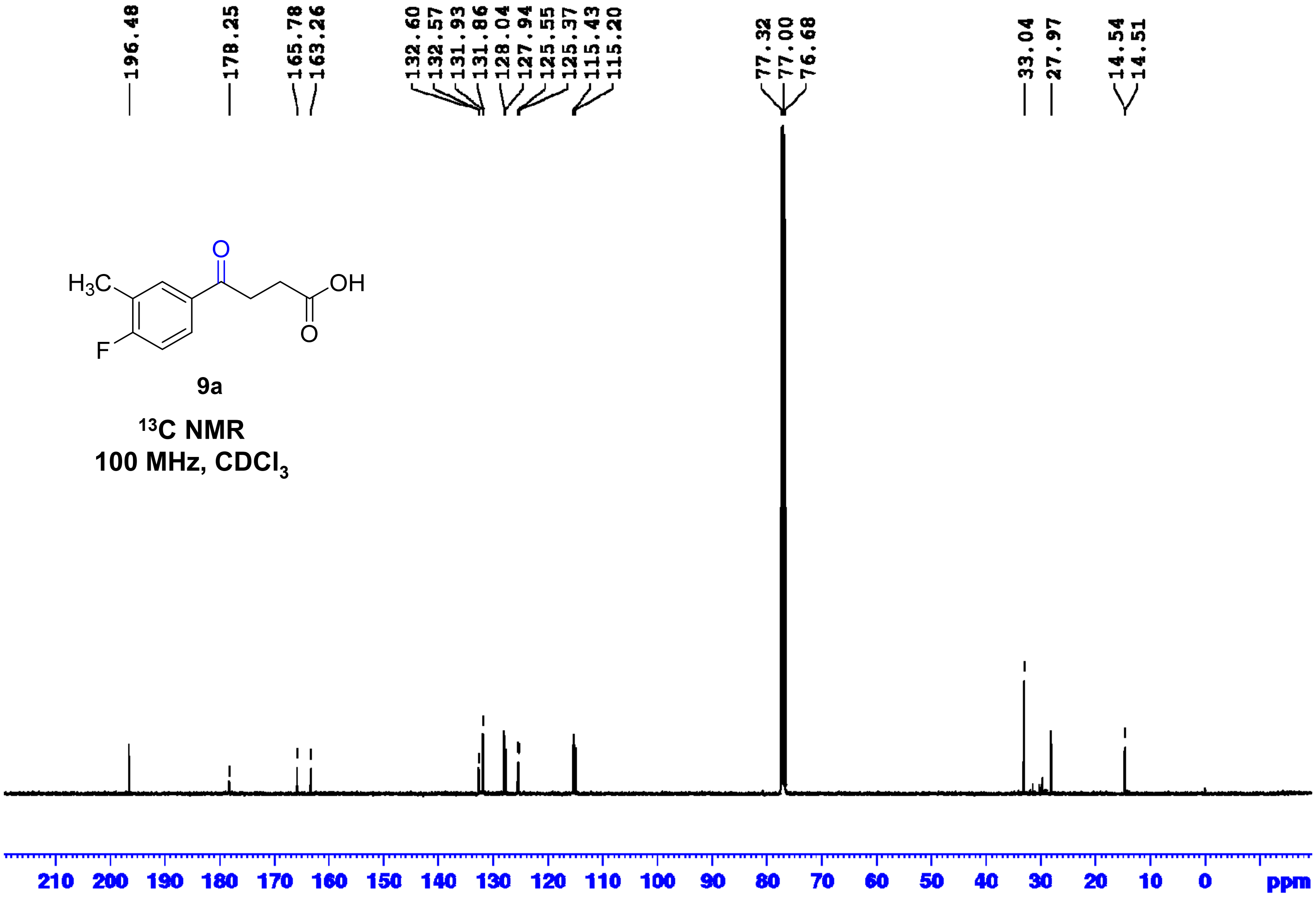

9a

^{13}C NMR
100 MHz, $CDCl_3$

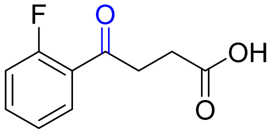

10a

^{1}H NMR,
400 MHz, $CDCl_3$

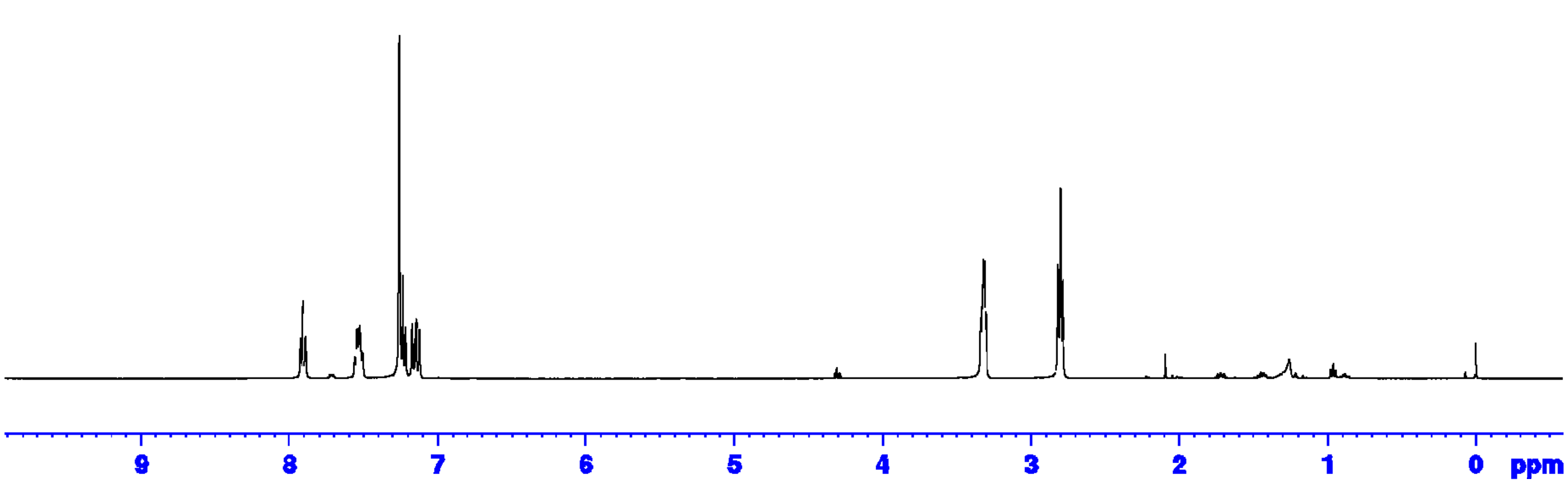

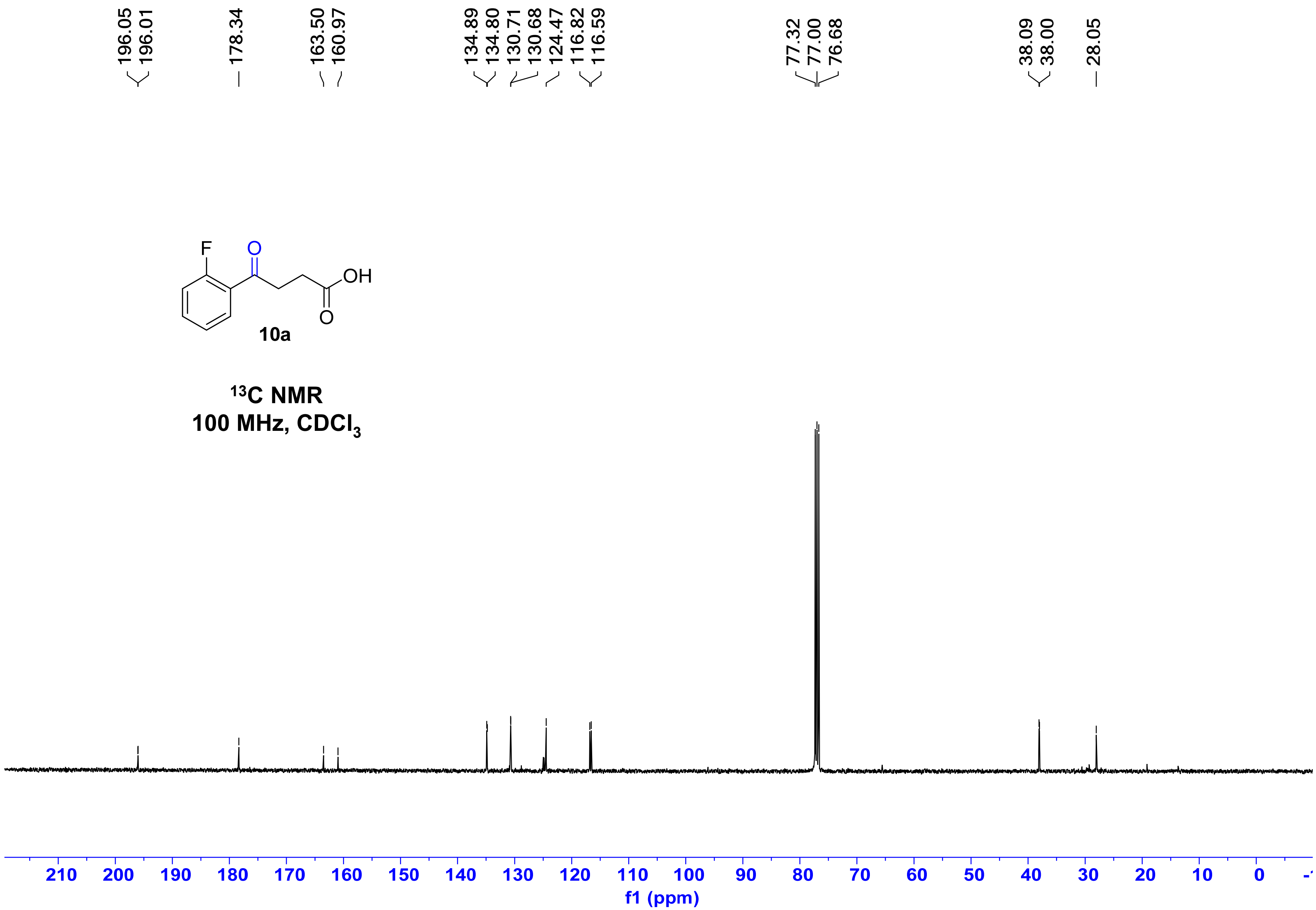
196.05
196.01
178.34
163.50
160.97
134.89
134.80
130.71
130.68
124.47
116.82
116.59
77.32
77.00
76.68
38.09
38.00
28.05
F
O
OH
O
10a
^{13}C NMR
100 MHz, $CDCl_3$
210
200
190
180
170
160
150
140
130
120
110
100
90
80
70
60
50
40
30
20
10
0
f1 (ppm)

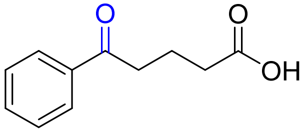

11a

^{1}H NMR,
400 MHz, $CDCl_3$

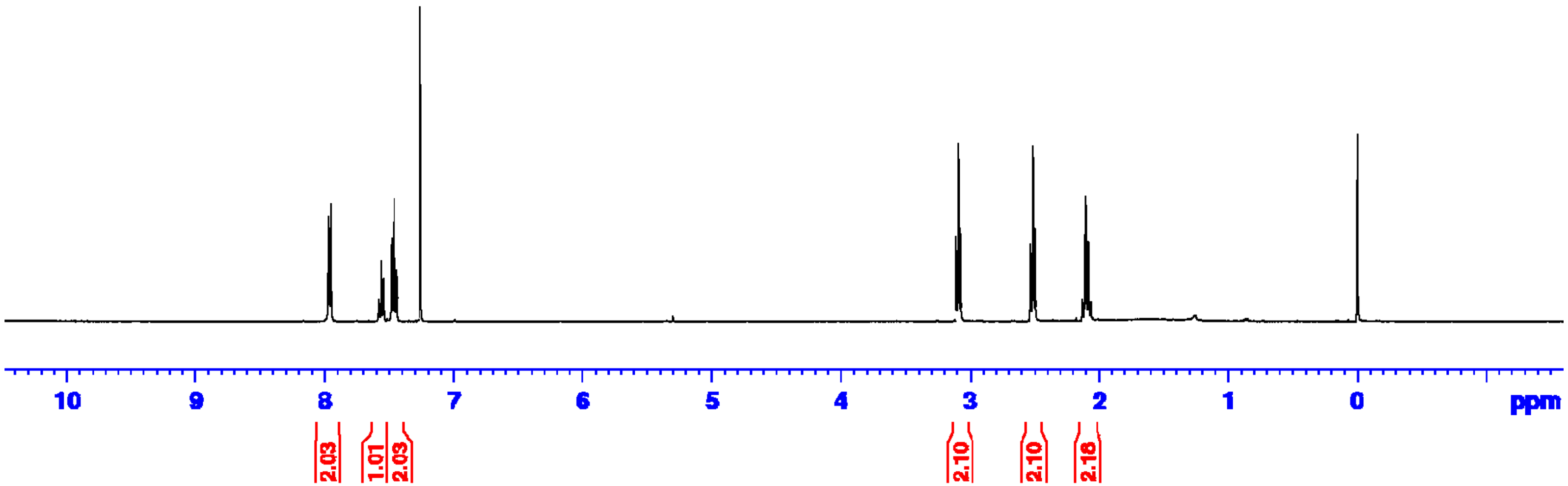

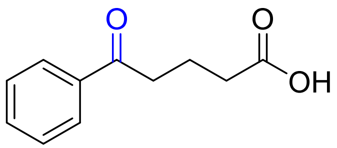

11a

^{13}C NMR
100 MHz, $CDCl_3$

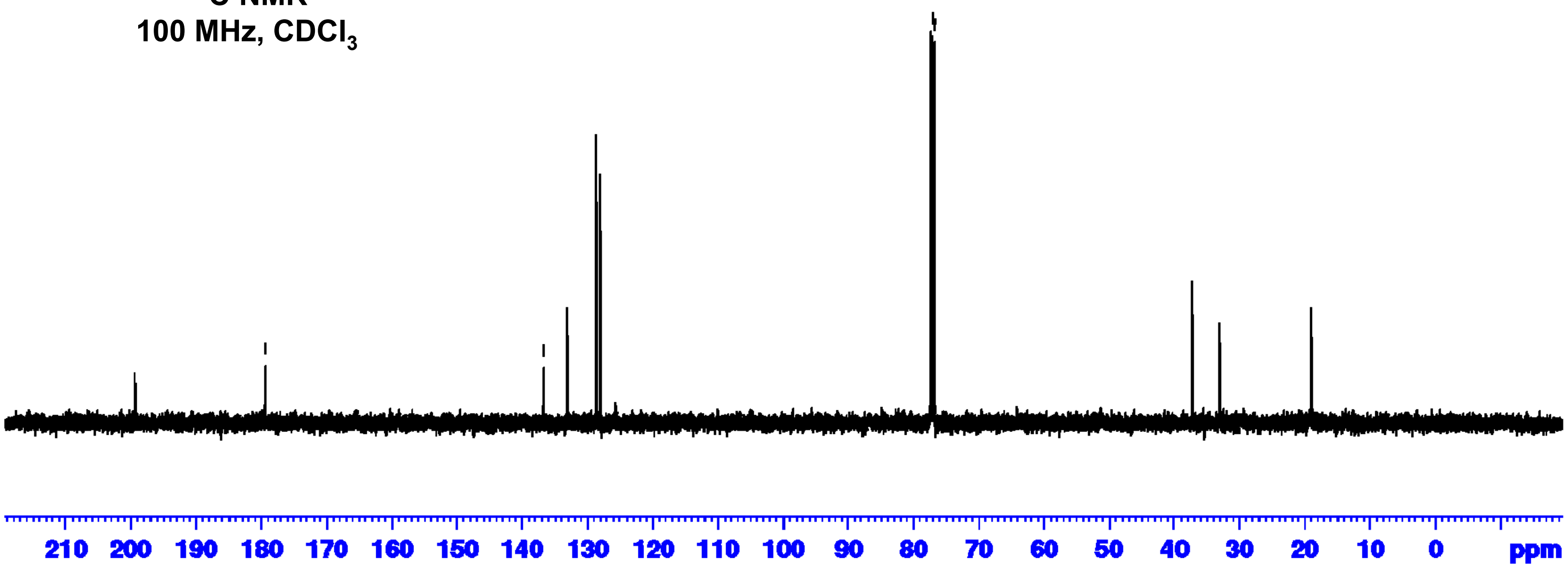

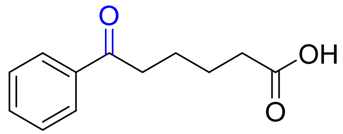

12a

^{1}H NMR,
400 MHz, $CDCl_3$

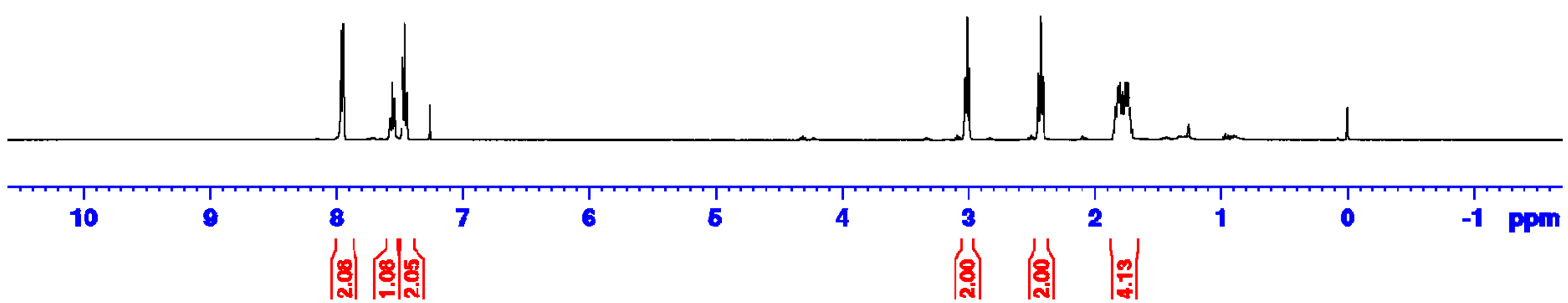

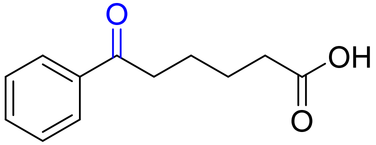

12a

^{13}C NMR
100 MHz, $CDCl_3$

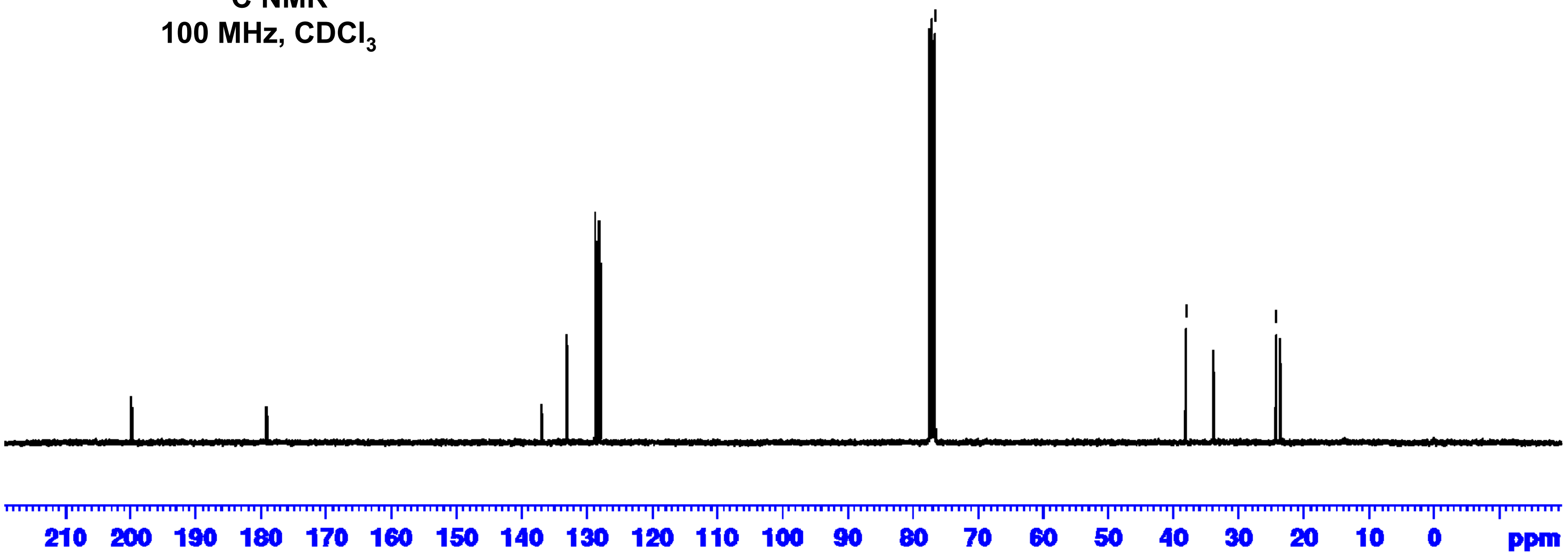

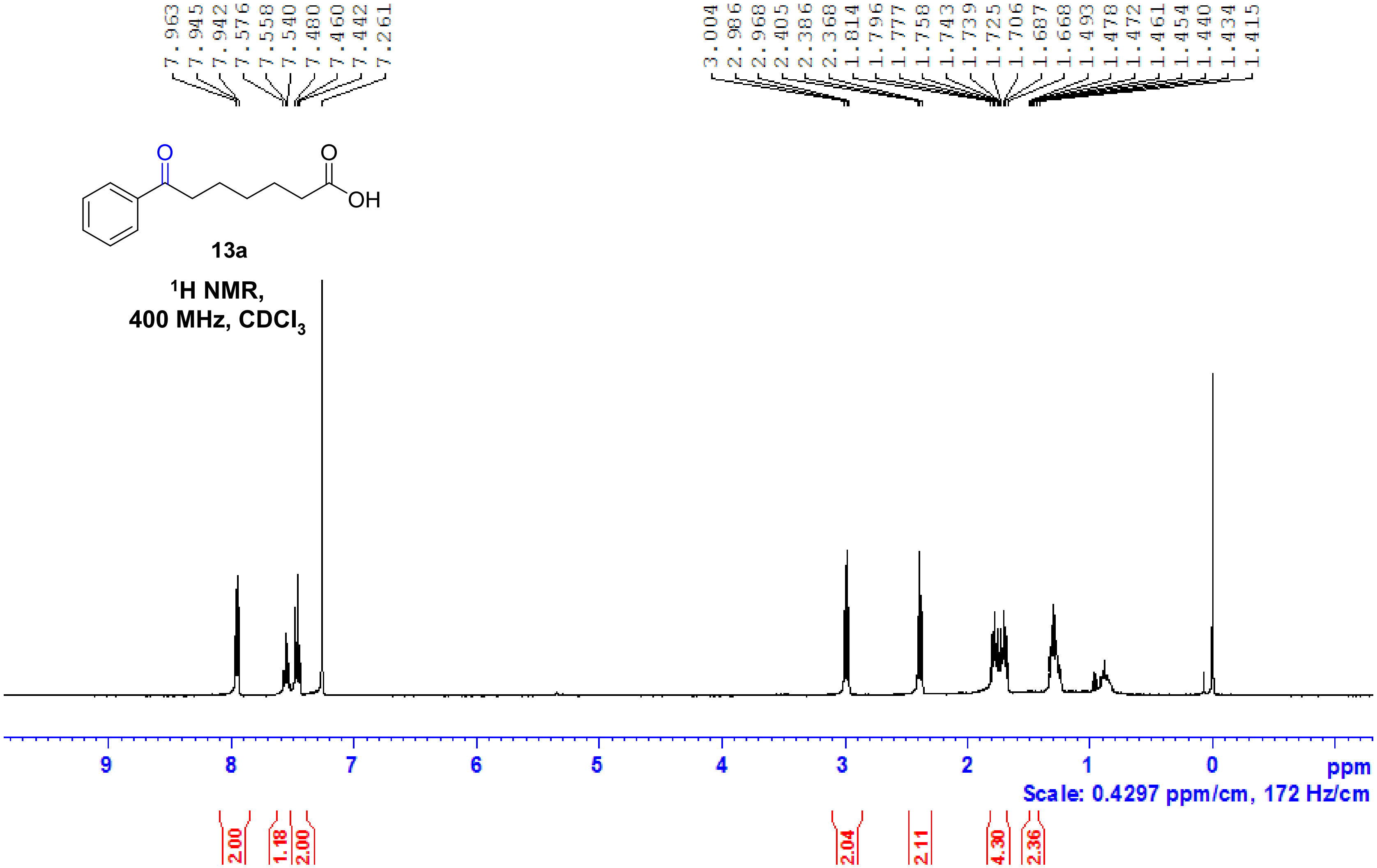

7.963
7.945
7.942
7.576
7.558
7.540
7.480
7.460
7.442
7.261
3.004
2.986
2.968
2.405
2.386
2.368
1.814
1.796
1.777
1.758
1.743
1.739
1.725
1.706
1.687
1.668
1.493
1.478
1.472
1.461
1.454
1.440
1.434
1.415
O
O
OH
13a
1H NMR,
400 MHz, CDCl3
9
8
7
6
5
4
3
2
1
0
ppm
Scale: 0.4297 ppm/cm, 172 Hz/cm
2.00
1.18
2.00
2.04
2.11
4.30
2.36

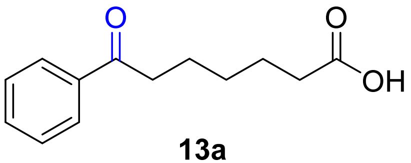

13a

^{13}C NMR
100 MHz, $CDCl_3$

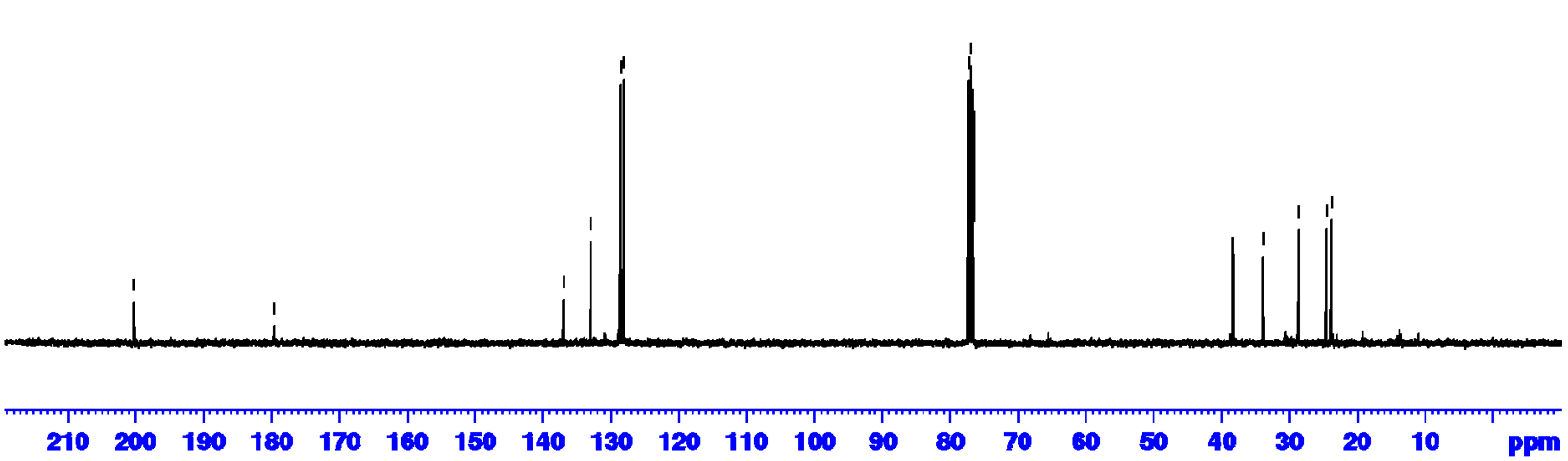

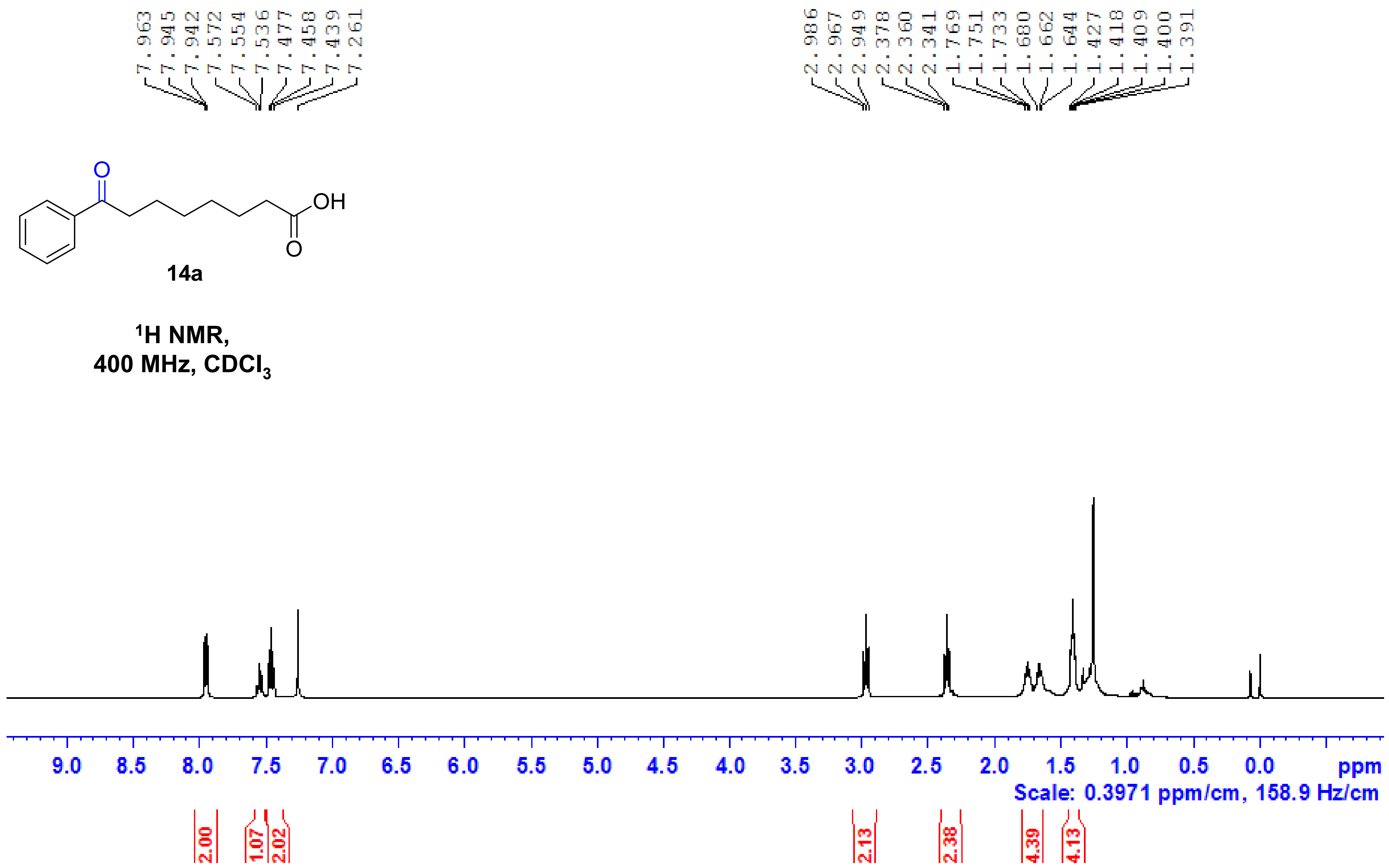
7.963
7.945
7.942
7.572
7.554
7.536
7.477
7.458
7.439
7.261
2.986
2.967
2.949
2.378
2.360
2.341
1.769
1.751
1.733
1.680
1.662
1.644
1.427
1.418
1.409
1.400
1.391
OH
O
O
14a
^{1}H NMR,
400 MHz, $CDCl_3$
9.0
8.5
8.0
7.5
7.0
6.5
6.0
5.5
5.0
4.5
4.0
3.5
3.0
2.5
2.0
1.5
1.0
0.5
0.0
ppm
Scale: 0.3971 ppm/cm, 158.9 Hz/cm
2.00
1.07
2.02
2.13
2.38
4.39
4.13

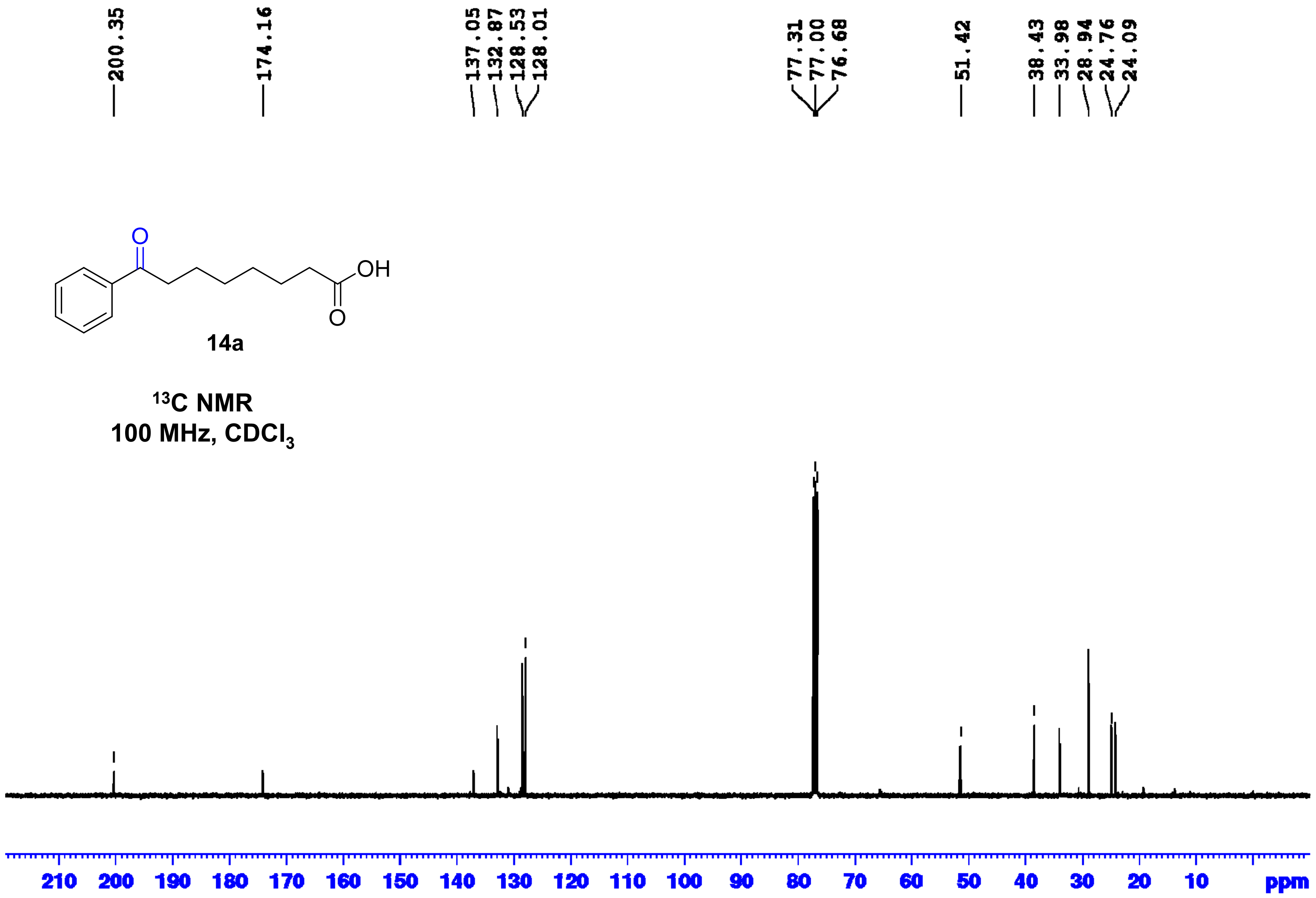
200.35
174.16
137.05
132.87
128.53
128.01
77.31
77.00
76.68
51.42
38.43
33.98
28.94
24.76
24.09
OH
O
O
14a
^{13}C NMR
100 MHz, $CDCl_3$
210
200
190
180
170
160
150
140
130
120
110
100
90
80
70
60
50
40
30
20
10
ppm

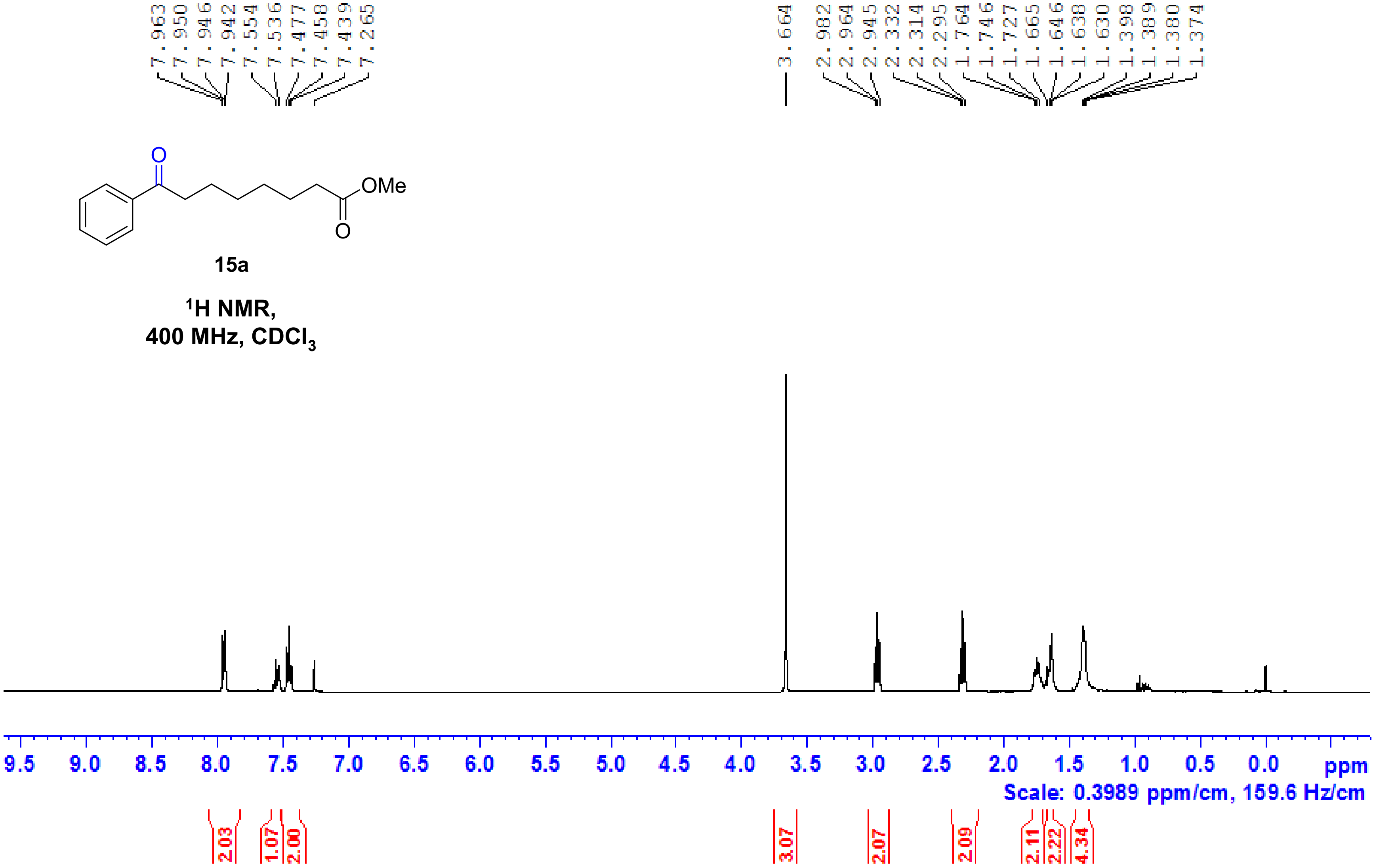

7.963
7.950
7.946
7.942
7.554
7.536
7.477
7.458
7.439
7.265
3.664
2.982
2.964
2.945
2.332
2.314
2.295
1.764
1.746
1.727
1.665
1.646
1.638
1.630
1.398
1.389
1.380
1.374
O
OMe
O
15a
1H NMR,
400 MHz, CDCl3
9.5
9.0
8.5
8.0
7.5
7.0
6.5
6.0
5.5
5.0
4.5
4.0
3.5
3.0
2.5
2.0
1.5
1.0
0.5
0.0
ppm
Scale: 0.3989 ppm/cm, 159.6 Hz/cm
2.03
1.07
2.00
3.07
2.07
2.09
2.11
2.22
4.34

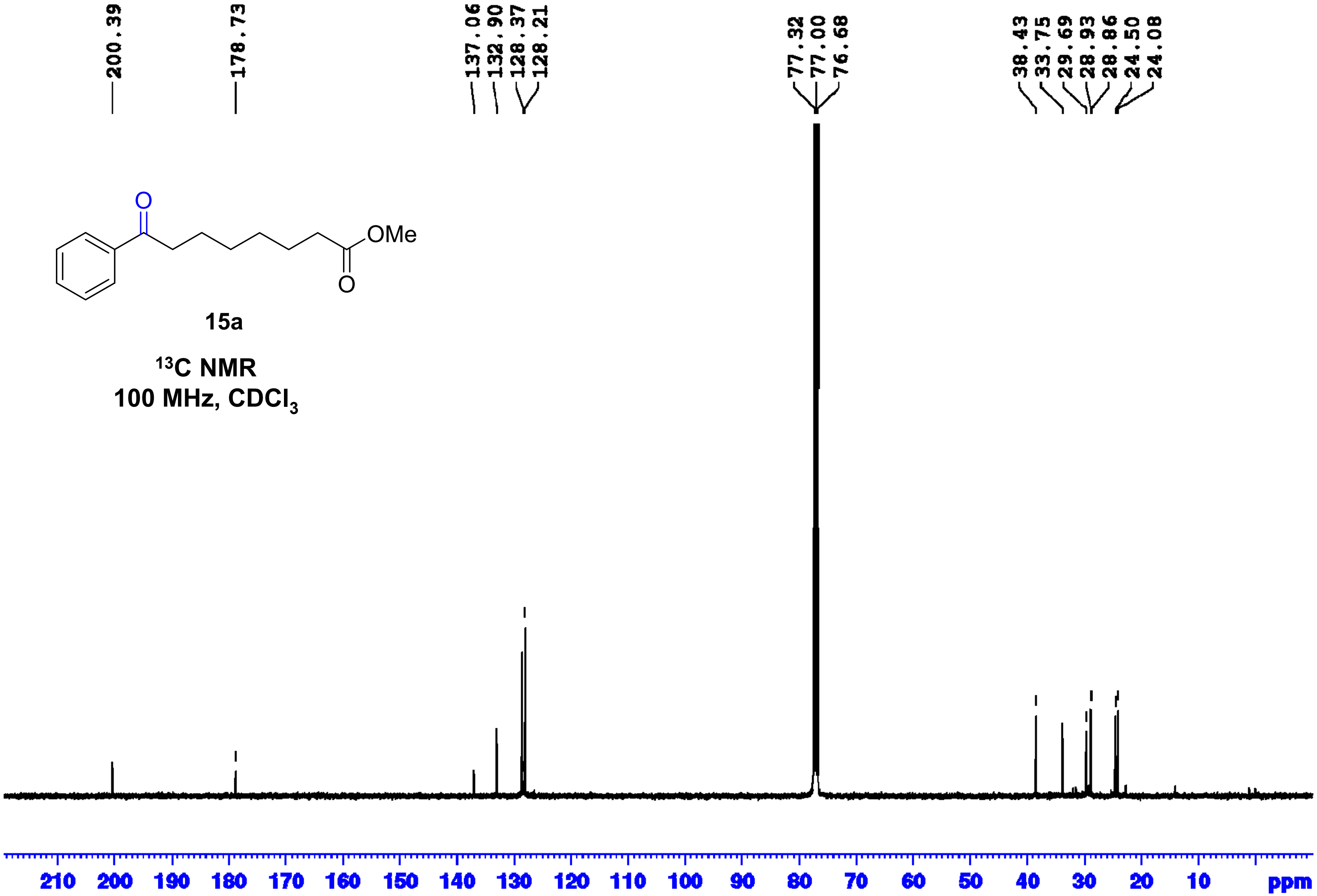

15a

^{13}C NMR
100 MHz, $CDCl_3$

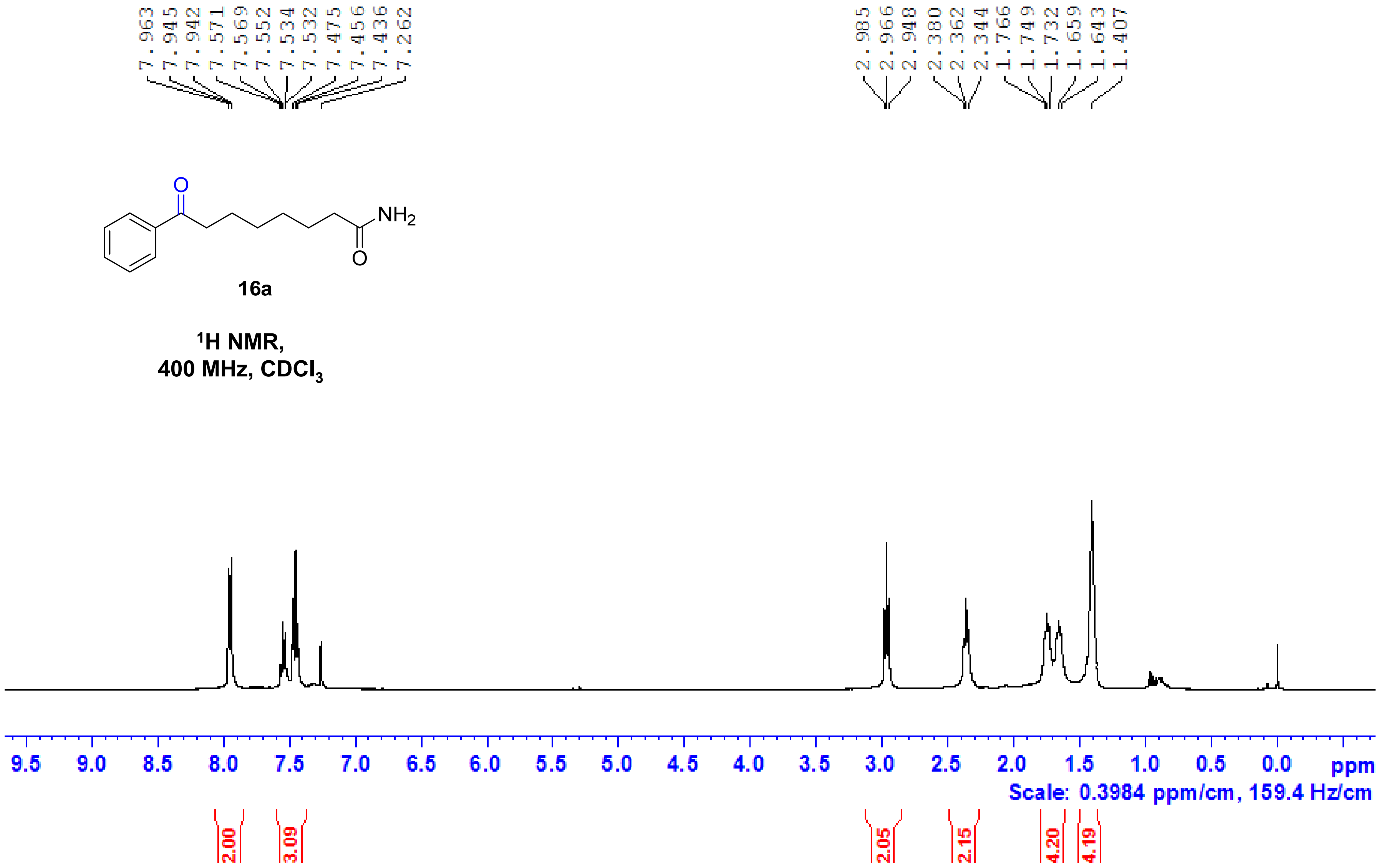
7.963
7.945
7.942
7.571
7.569
7.552
7.534
7.532
7.475
7.456
7.436
7.262
2.985
2.966
2.948
2.380
2.362
2.344
1.766
1.749
1.732
1.659
1.643
1.407
O
NH2
O
16a
1H NMR,
400 MHz, CDCl3
9.5 9.0 8.5 8.0 7.5 7.0 6.5 6.0 5.5 5.0 4.5 4.0 3.5 3.0 2.5 2.0 1.5 1.0 0.5 0.0 ppm
Scale: 0.3984 ppm/cm, 159.4 Hz/cm
2.00
3.09
2.05
2.15
4.20
4.19

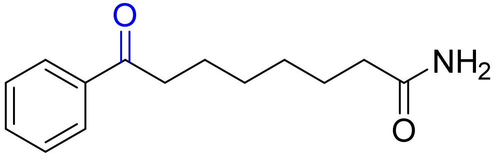

16a

^{13}C NMR
100 MHz, $CDCl_3$

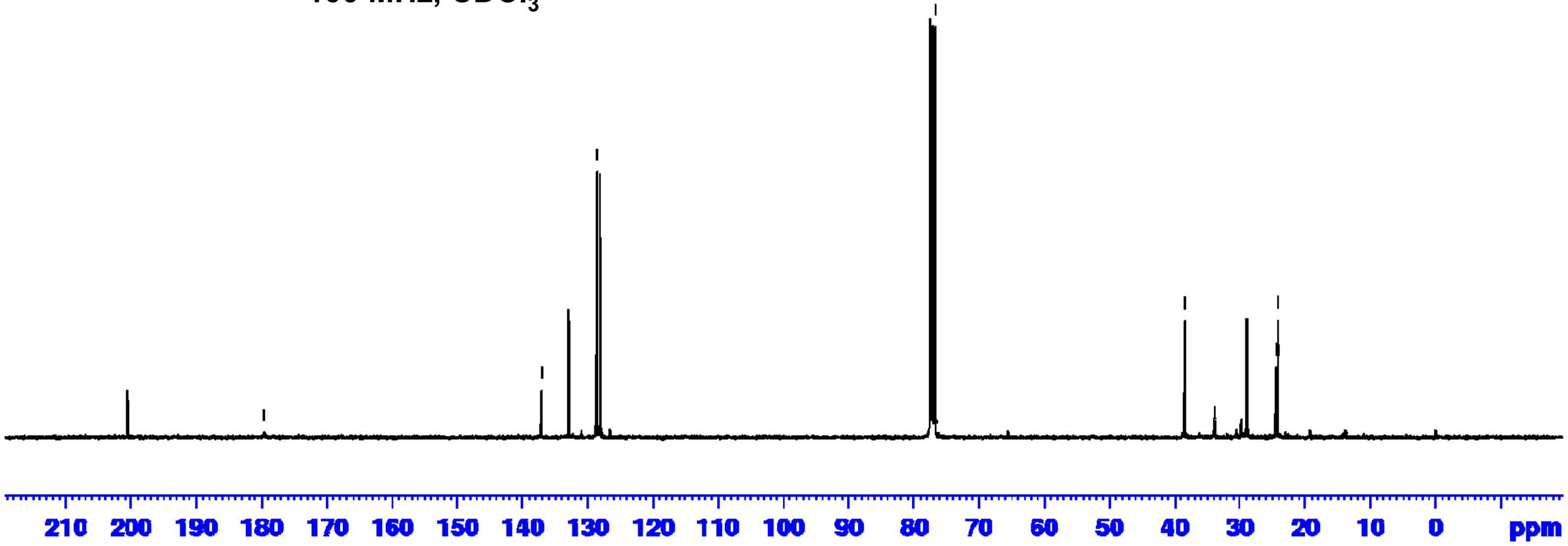

18a

^{1}H NMR,
400 MHz, $CDCl_3$

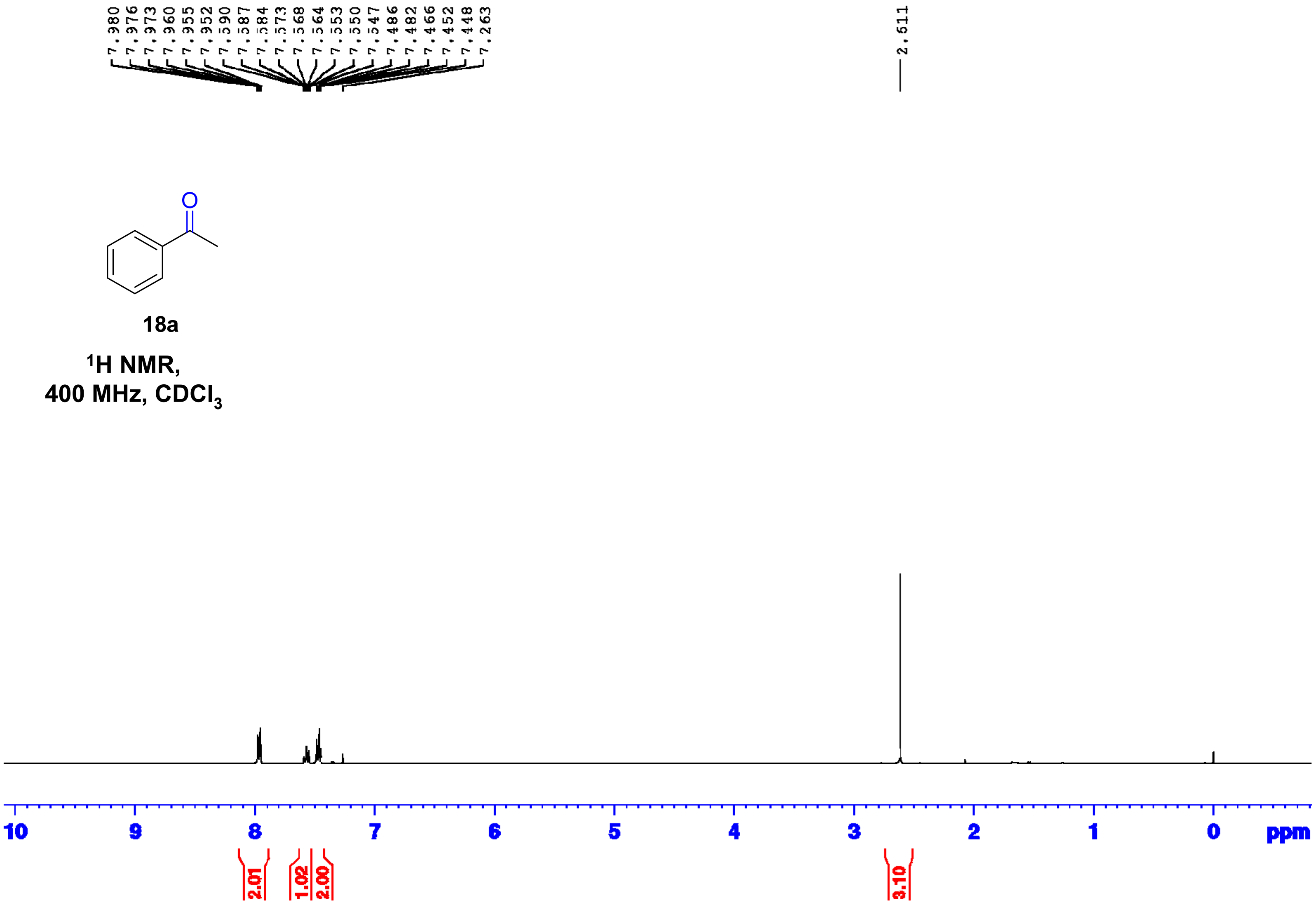

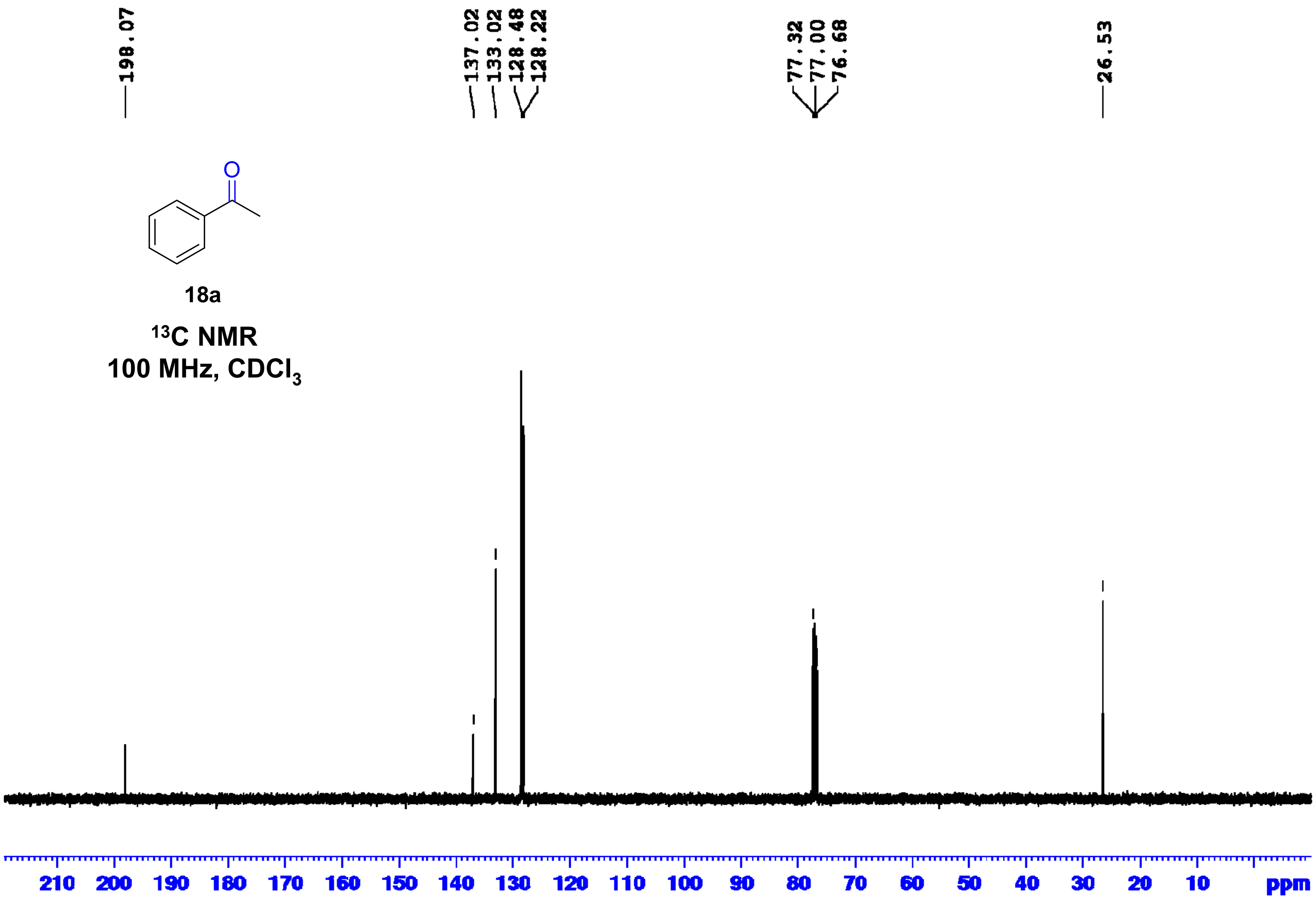
198.07
137.02
133.02
128.48
128.22
77.32
77.00
76.68
26.53
O
18a
^{13}C NMR
100 MHz, $CDCl_3$
210 200 190 180 170 160 150 140 130 120 110 100 90 80 70 60 50 40 30 20 10 ppm

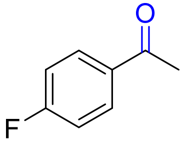

19a

^{1}H NMR,
400 MHz, $CDCl_3$

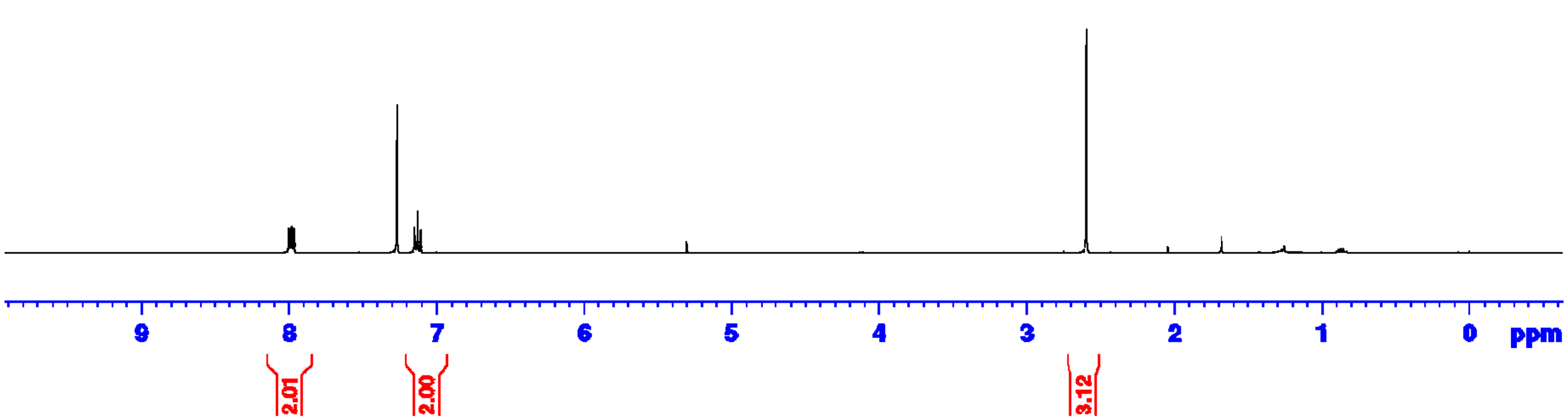

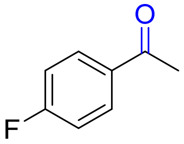

19a

^{13}C NMR
100 MHz, $CDCl_3$

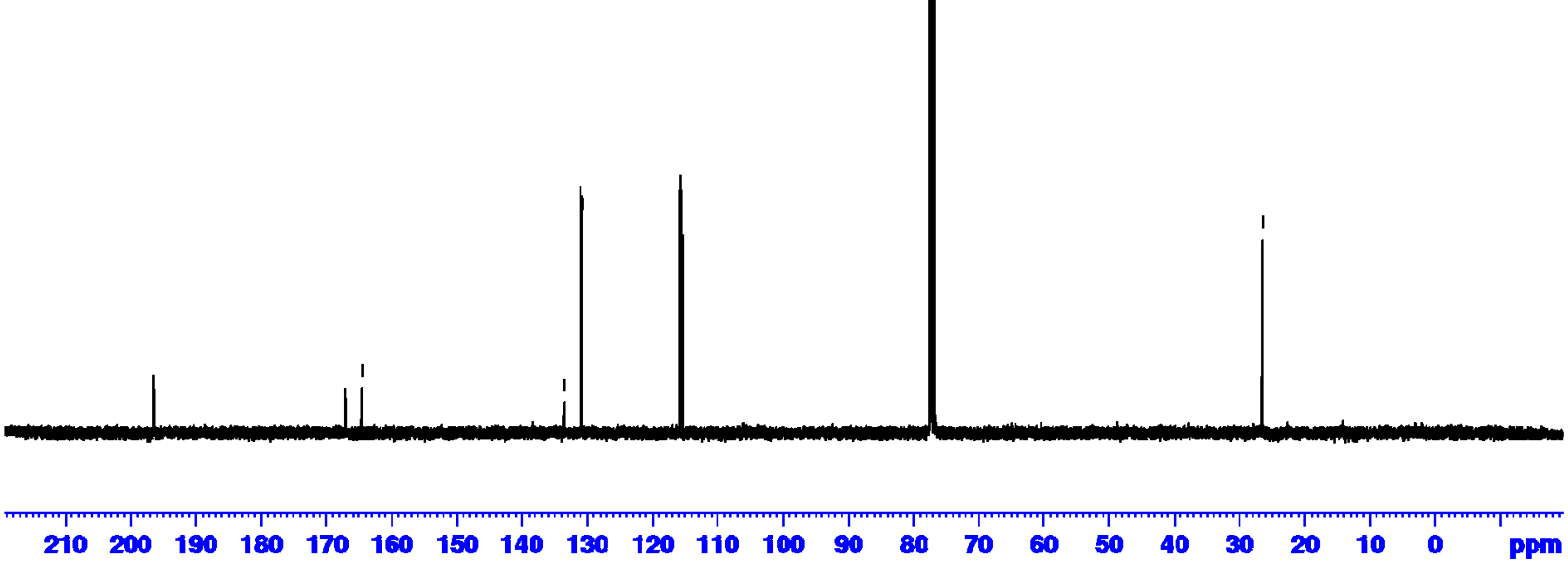

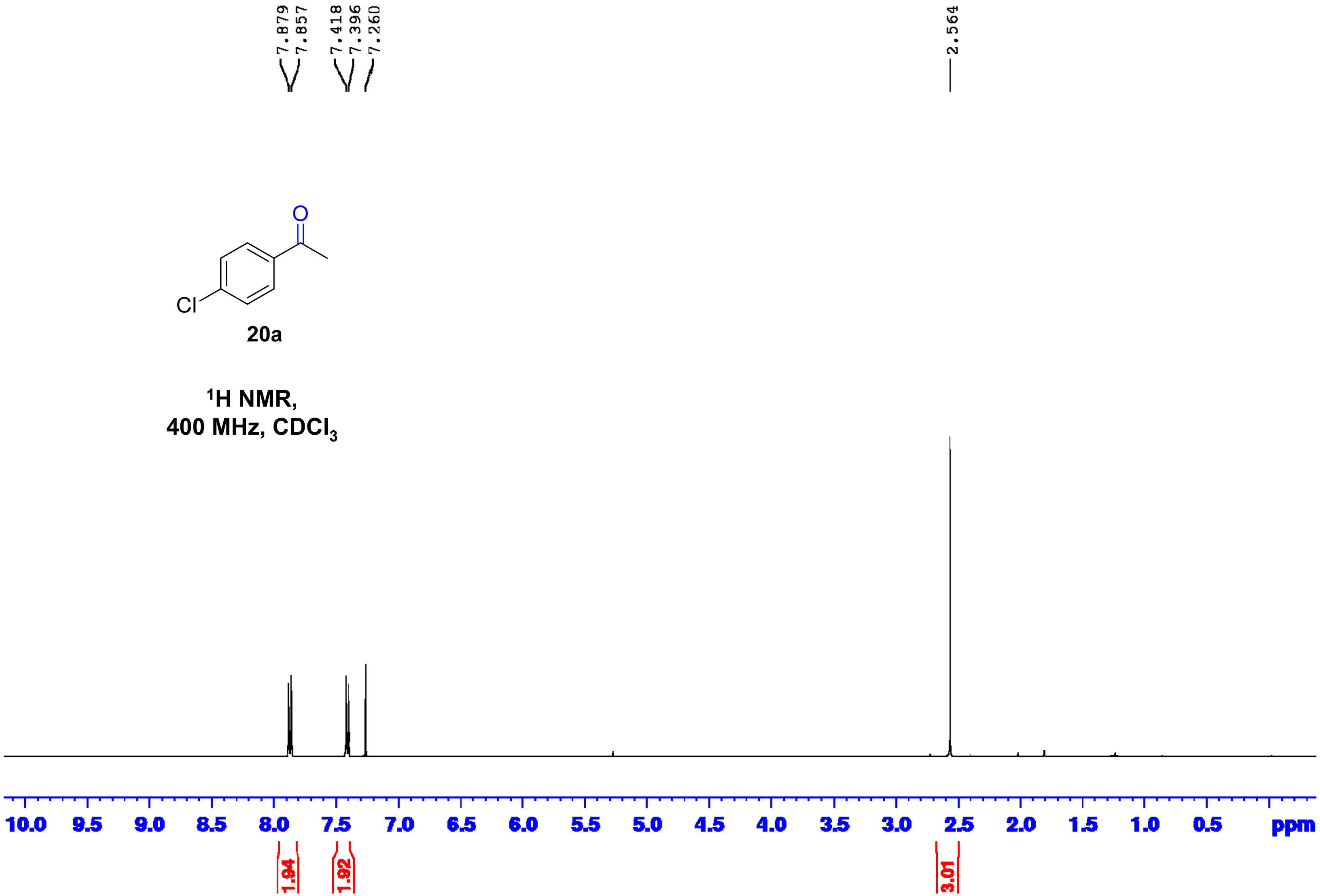
7.879
7.857
7.418
7.396
7.260
2.564
O
Cl
20a
1H NMR,
400 MHz, CDCl3
10.0 9.5 9.0 8.5 8.0 7.5 7.0 6.5 6.0 5.5 5.0 4.5 4.0 3.5 3.0 2.5 2.0 1.5 1.0 0.5 ppm
1.94
1.92
3.01

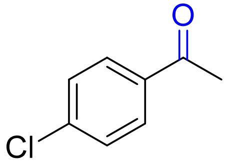

20a

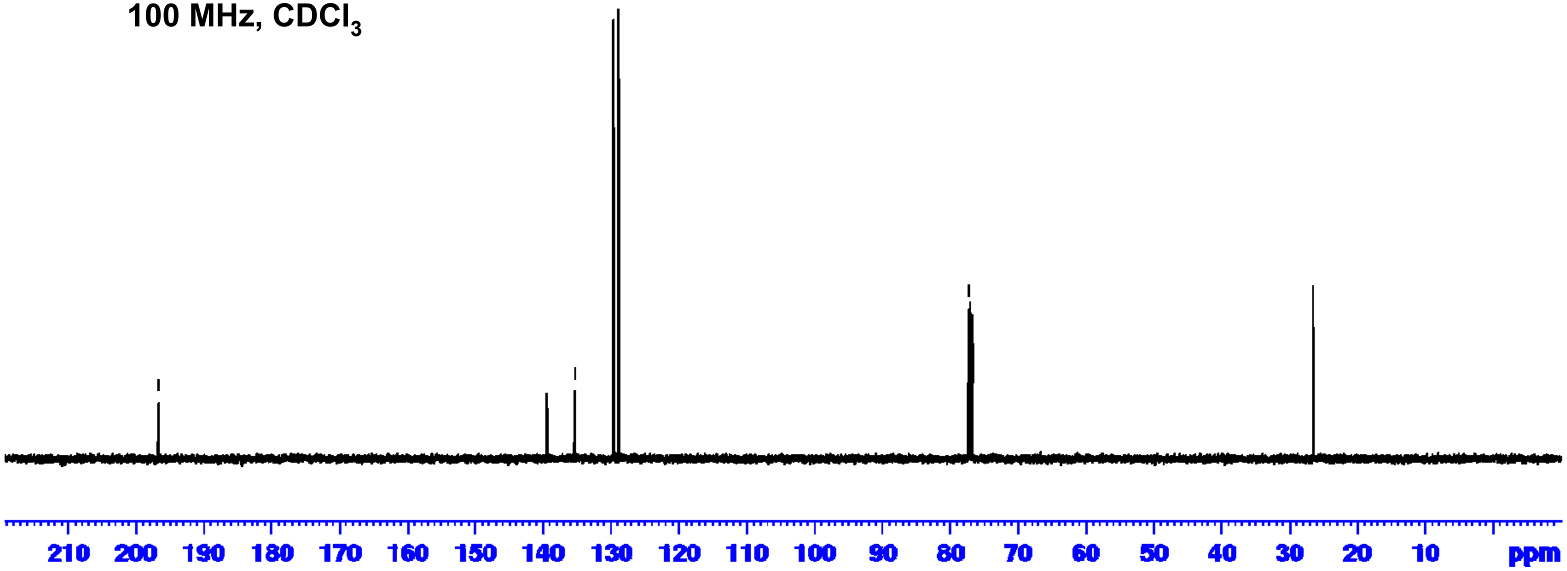

^{13}C NMR
100 MHz, $CDCl_3$

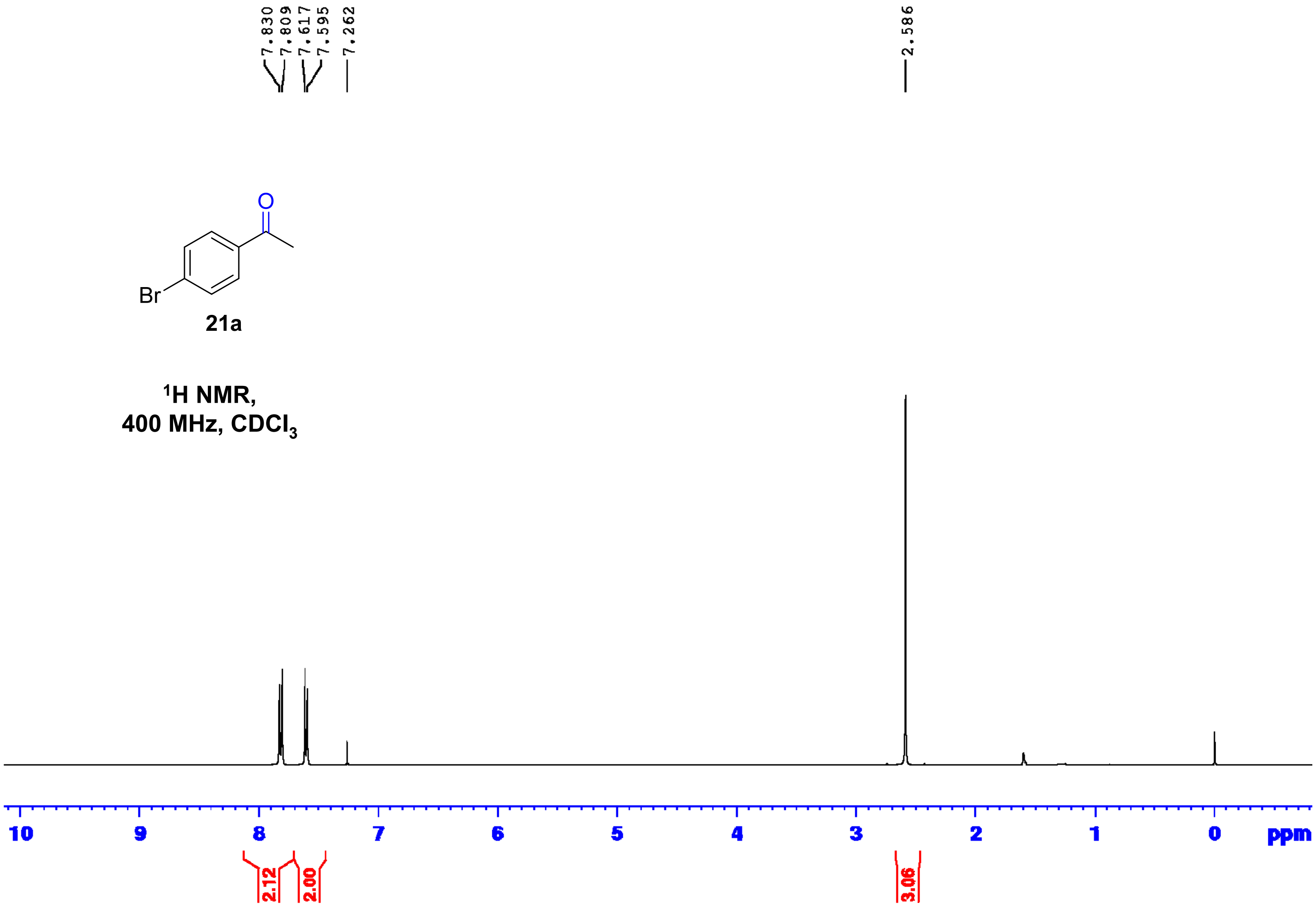
7.830
7.809
7.617
7.595
7.262
2.586
O
Br
21a
1H NMR,
400 MHz, CDCl3
10
9
8
7
6
5
4
3
2
1
0
ppm
2.12
2.00
3.06

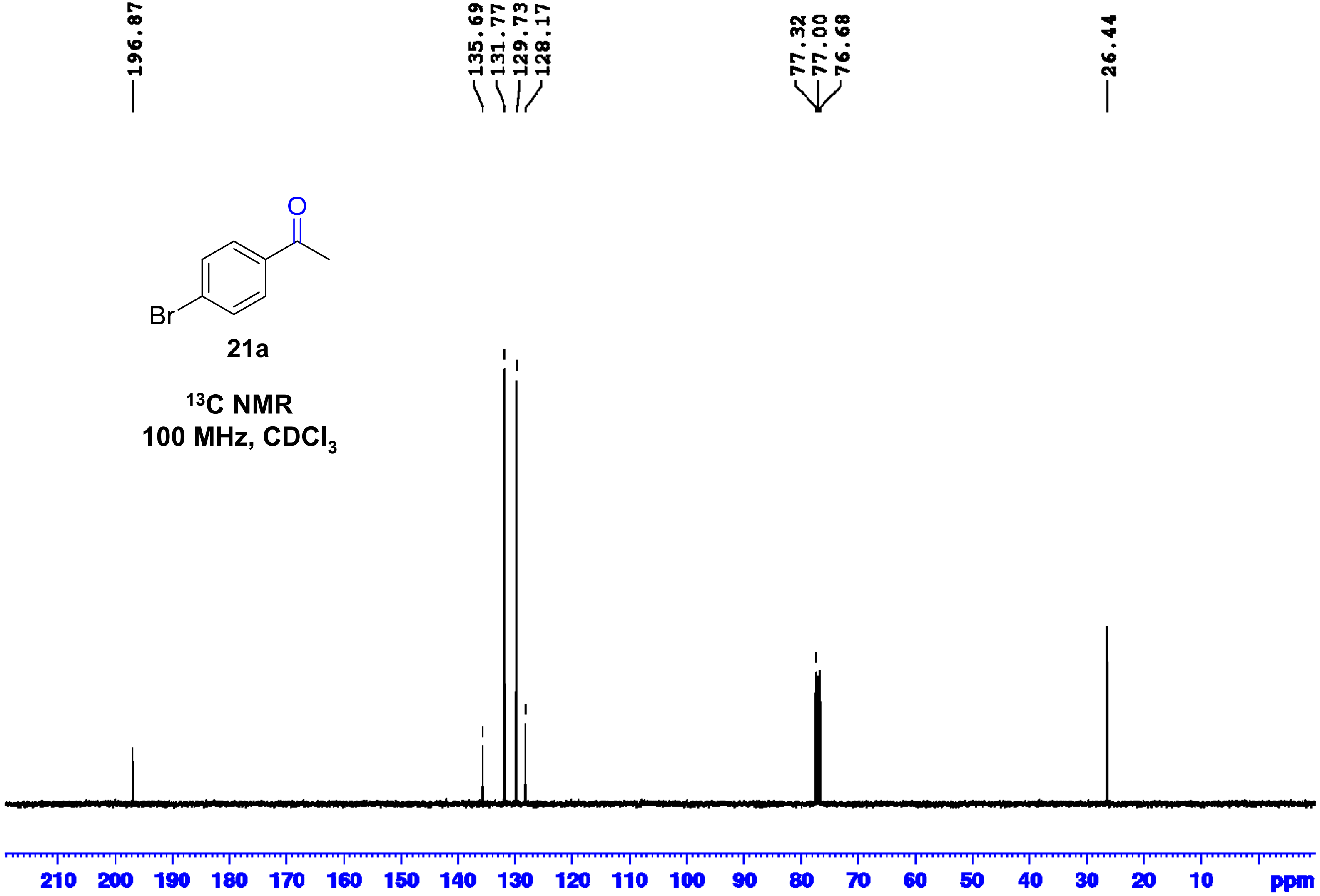
196.87
135.69
131.77
129.73
128.17
77.32
77.00
76.68
26.44
Br
O
21a
13C NMR
100 MHz, CDCl3
210 200 190 180 170 160 150 140 130 120 110 100 90 80 70 60 50 40 30 20 10 ppm

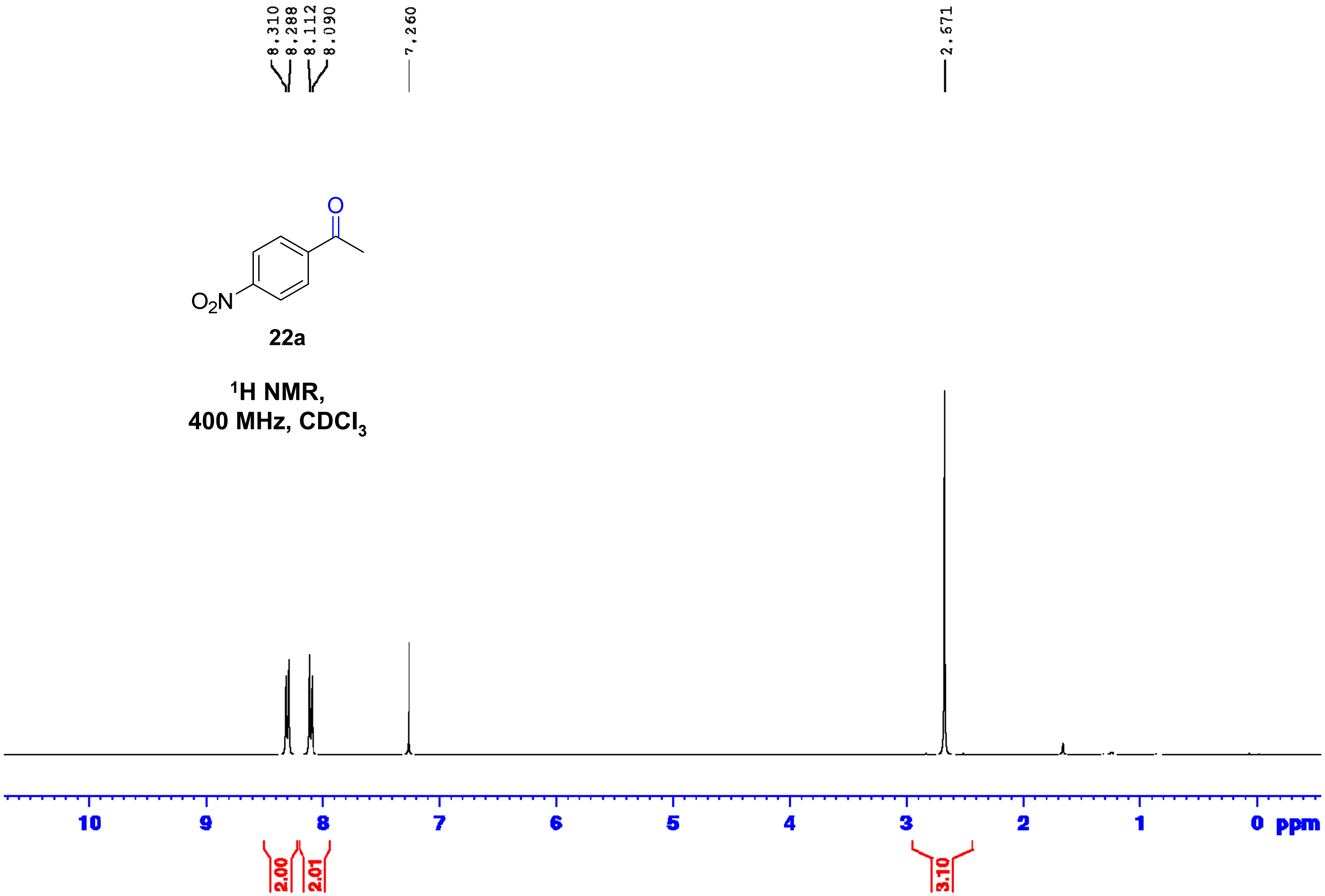
8.310
8.288
8.112
8.090
7.260
2.571
O
O2N
22a
1H NMR,
400 MHz, CDCl3
10
9
8
7
6
5
4
3
2
1
0
ppm
2.00
2.01
3.10

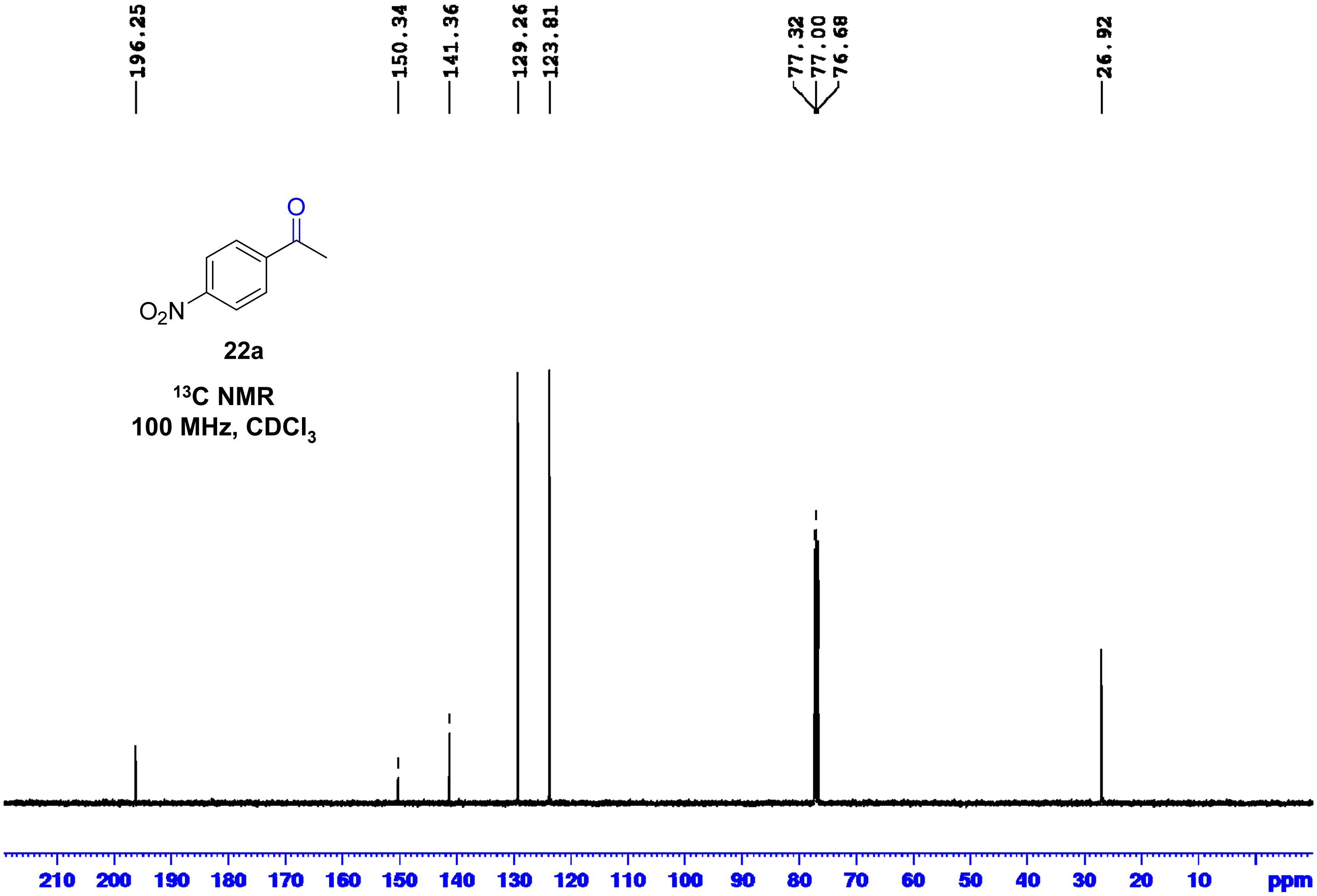
196.25
150.34
141.36
129.26
123.81
77.32
77.00
76.68
26.92
O
O2N
22a
13C NMR
100 MHz, CDCl3
210 200 190 180 170 160 150 140 130 120 110 100 90 80 70 60 50 40 30 20 10 ppm

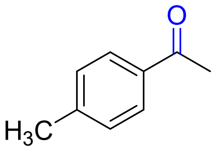

23a

^{1}H NMR,
400 MHz, $CDCl_3$

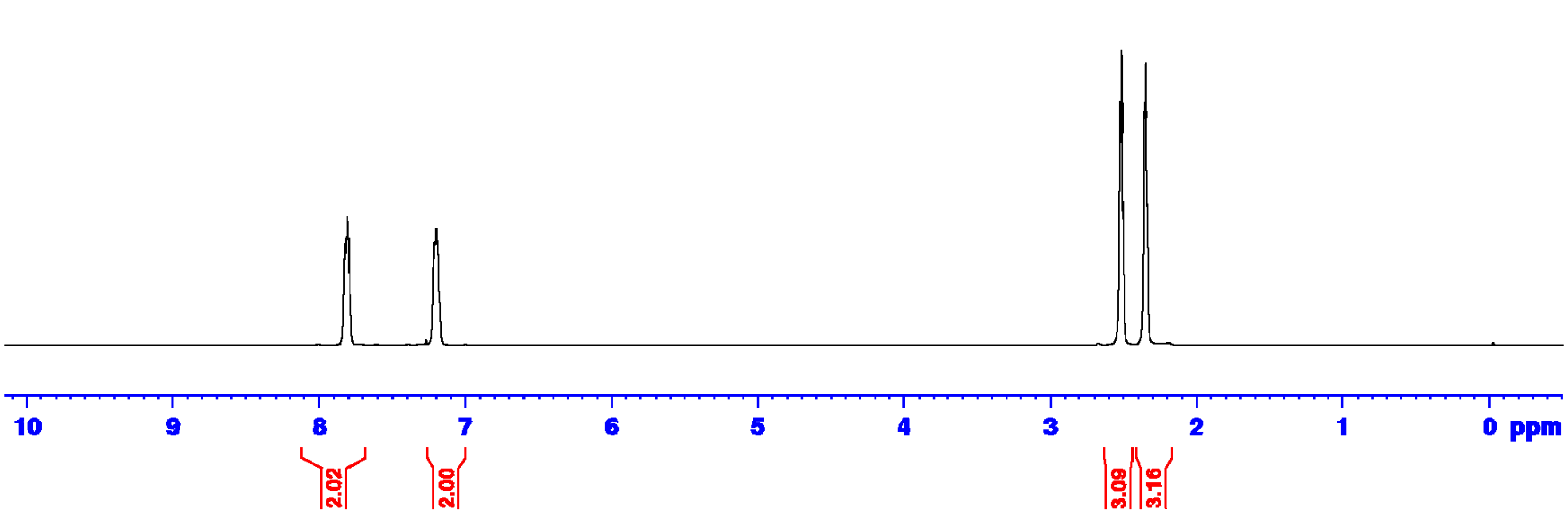

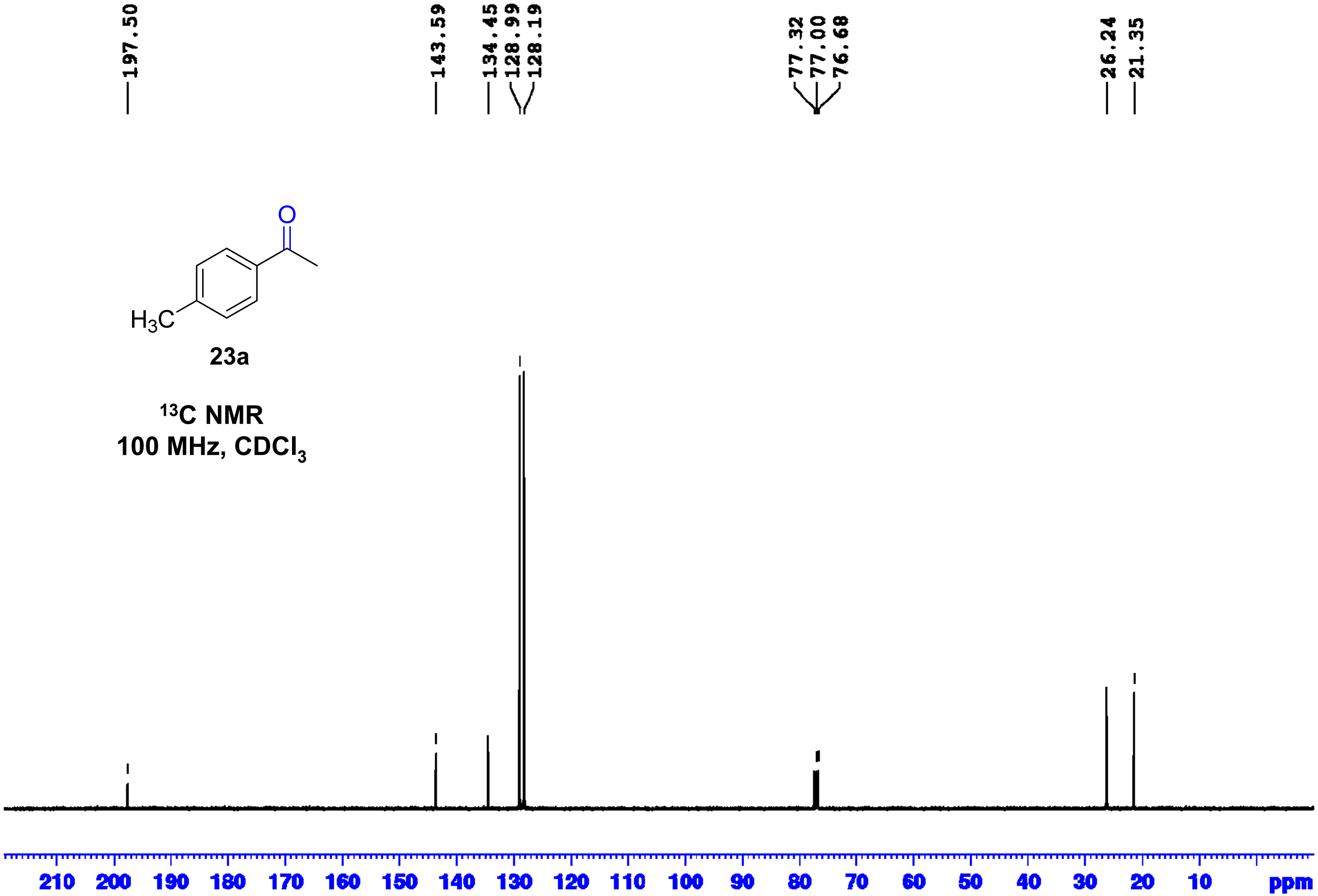
197.50
143.59
134.45
128.99
128.19
77.32
77.00
76.68
26.24
21.35
O
H3C
23a
13C NMR
100 MHz, CDCl3
210 200 190 180 170 160 150 140 130 120 110 100 90 80 70 60 50 40 30 20 10 ppm

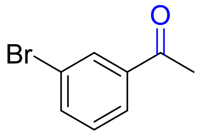

24a

**^{1}H NMR,
400 MHz, $CDCl_3$**

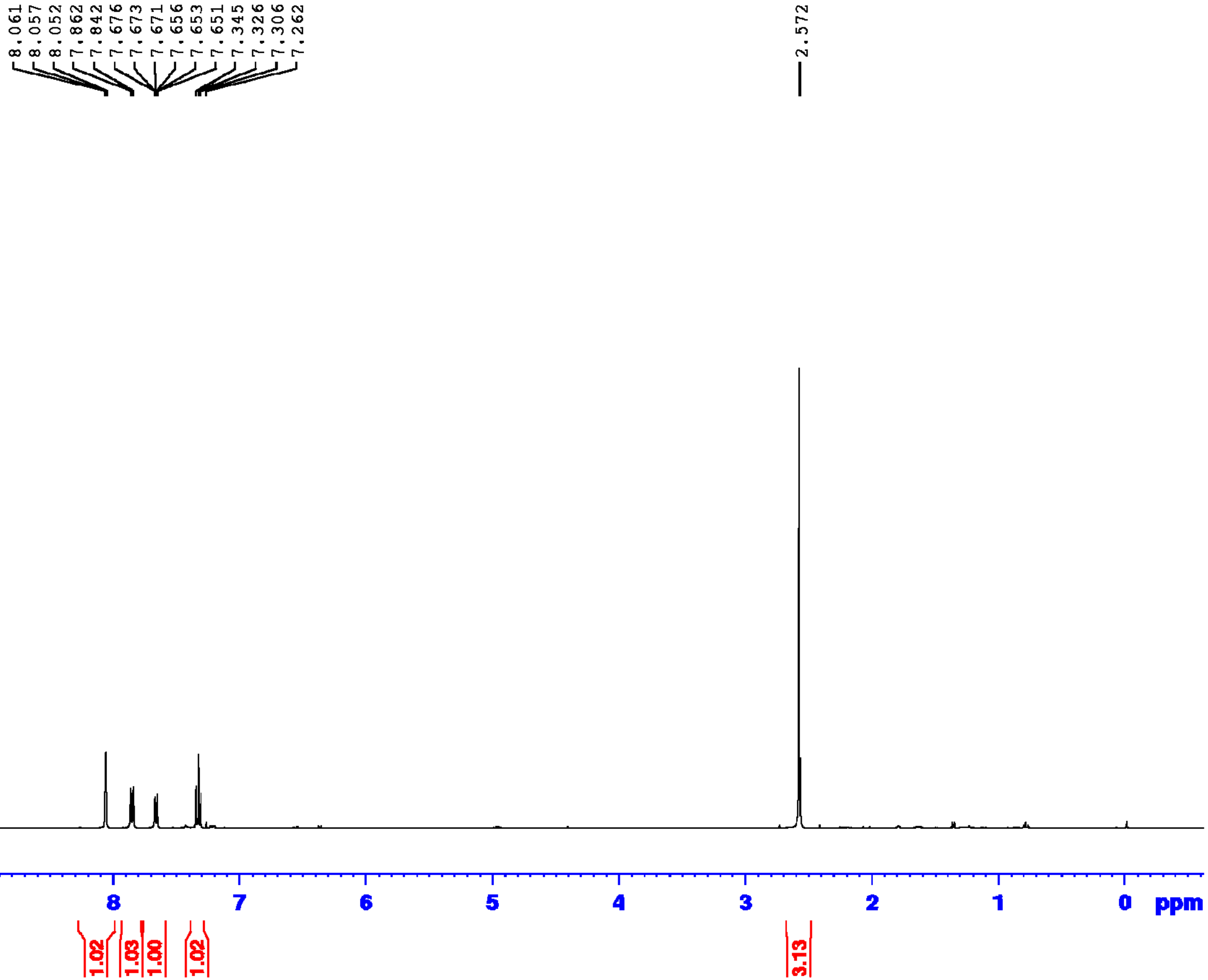

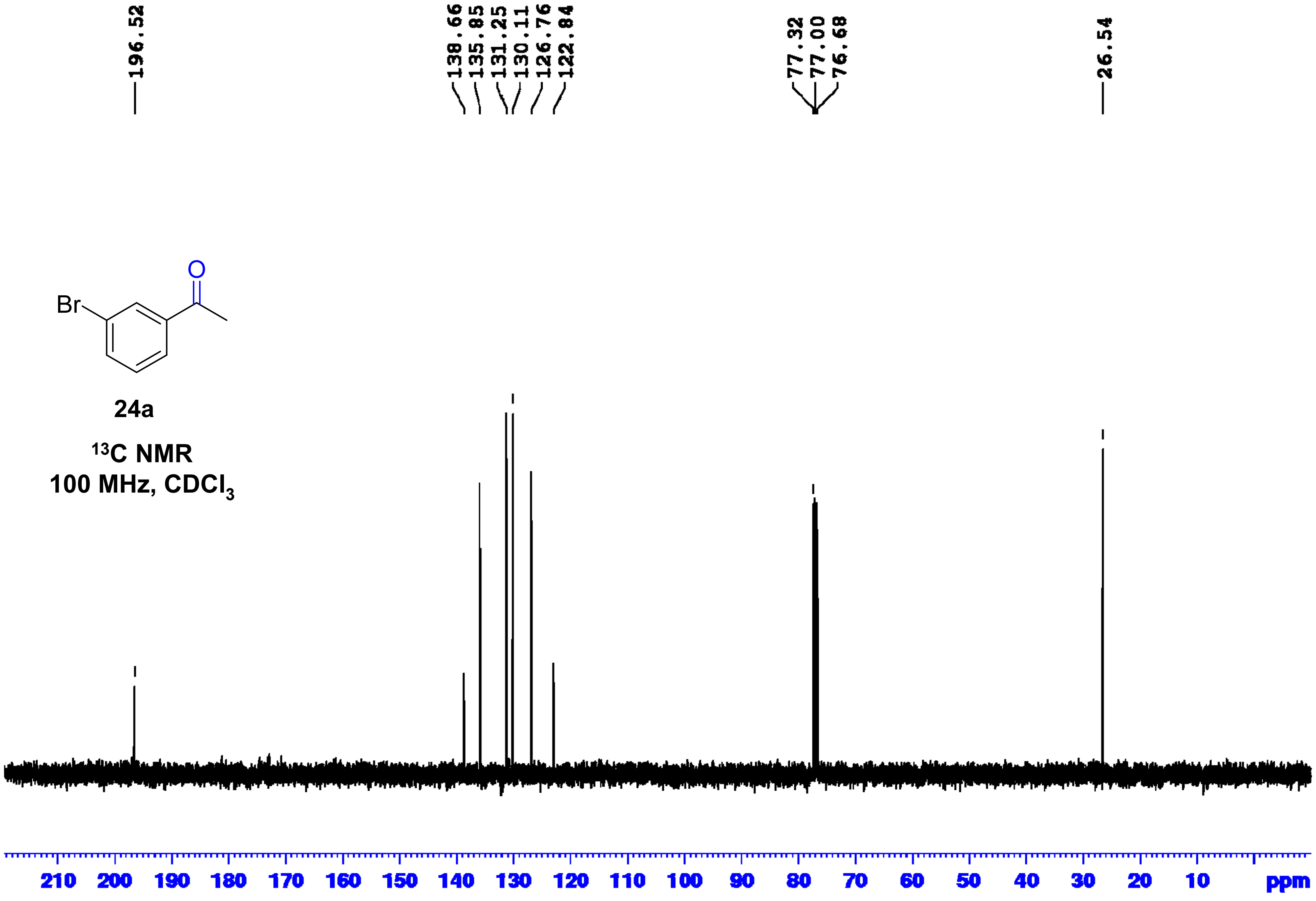
196.52
138.66
135.85
131.25
130.11
126.76
122.84
77.32
77.00
76.68
26.54
Br
O
24a
^{13}C NMR
100 MHz, $CDCl_3$
210 200 190 180 170 160 150 140 130 120 110 100 90 80 70 60 50 40 30 20 10 ppm

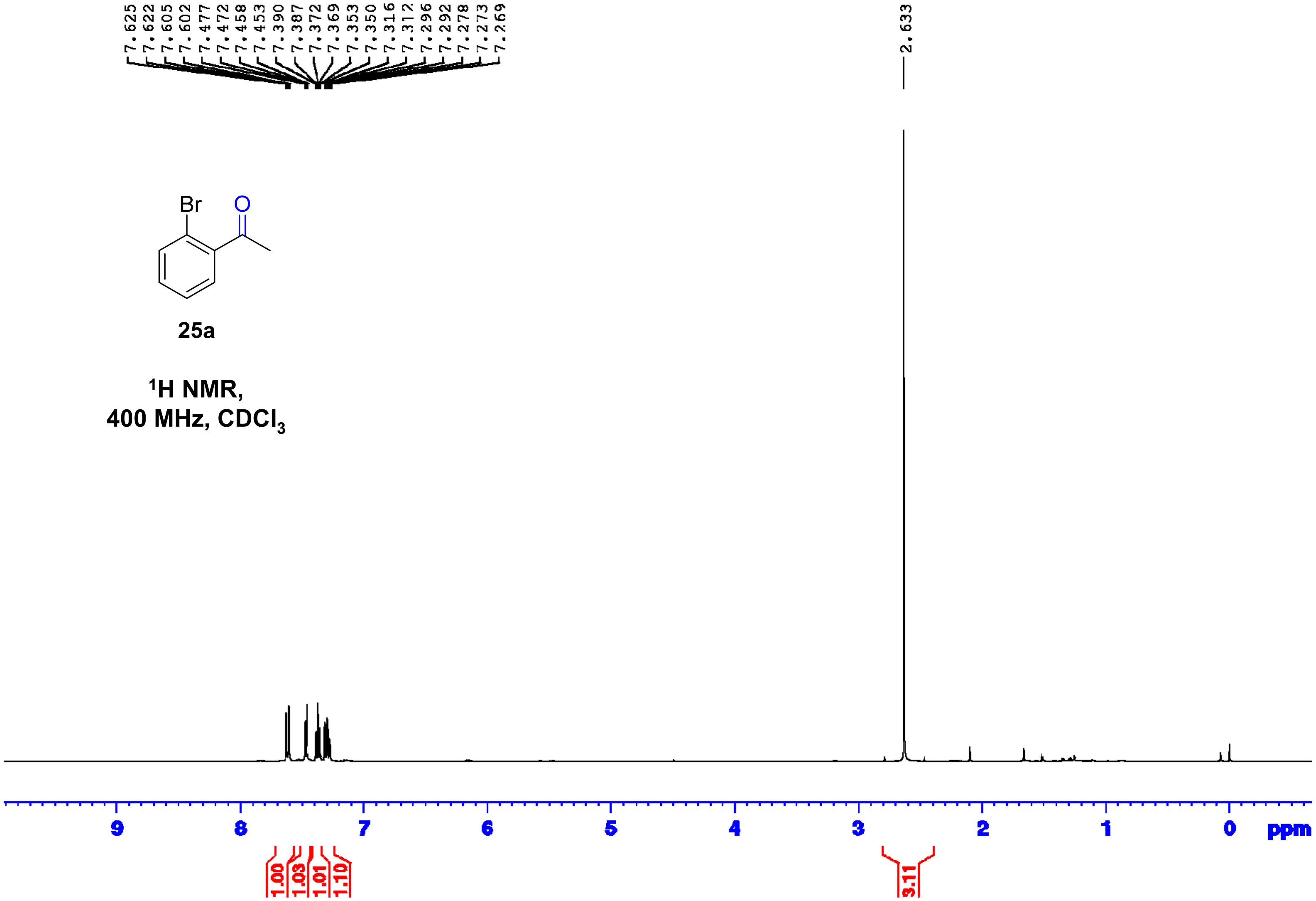

25a

^{1}H NMR,
400 MHz, $CDCl_3$

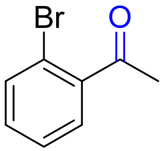

25a

^{13}C NMR
100 MHz, $CDCl_3$

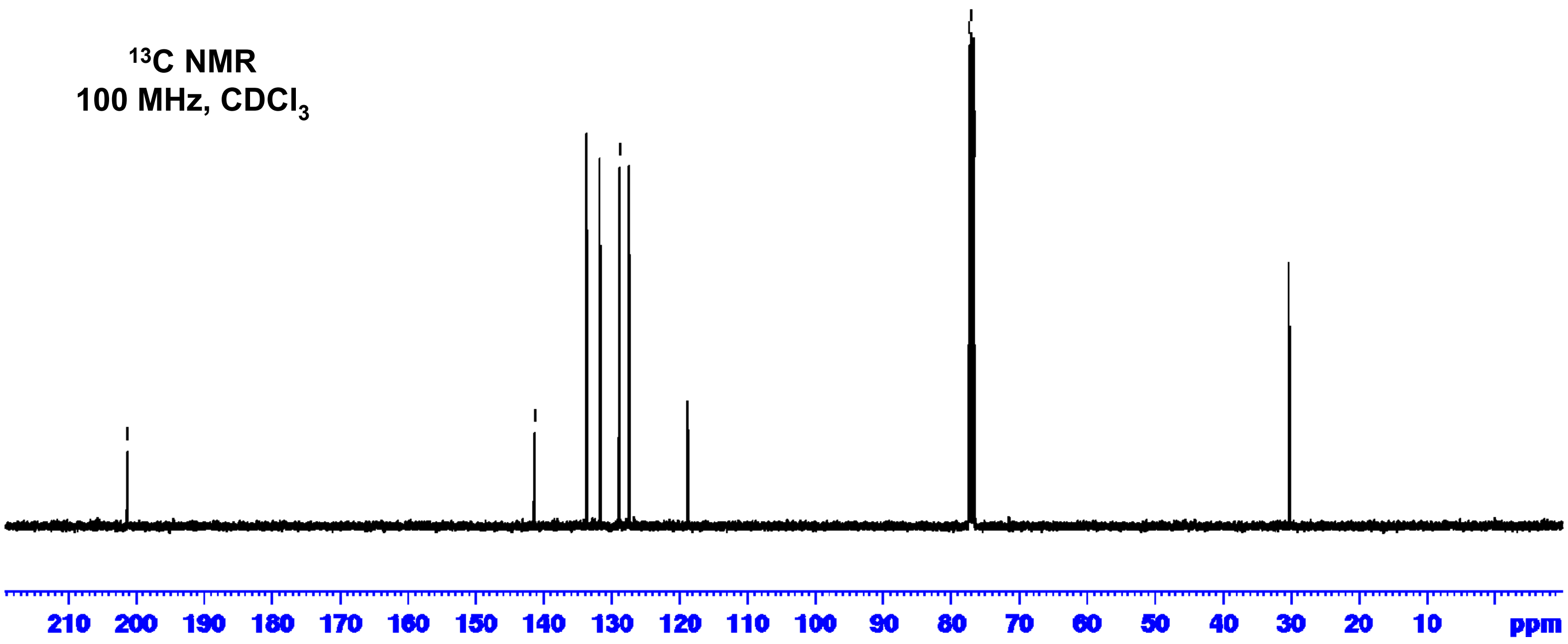

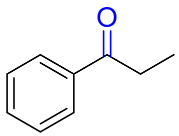

26a

[1]**H NMR,**
400 MHz, $CDCl_3$

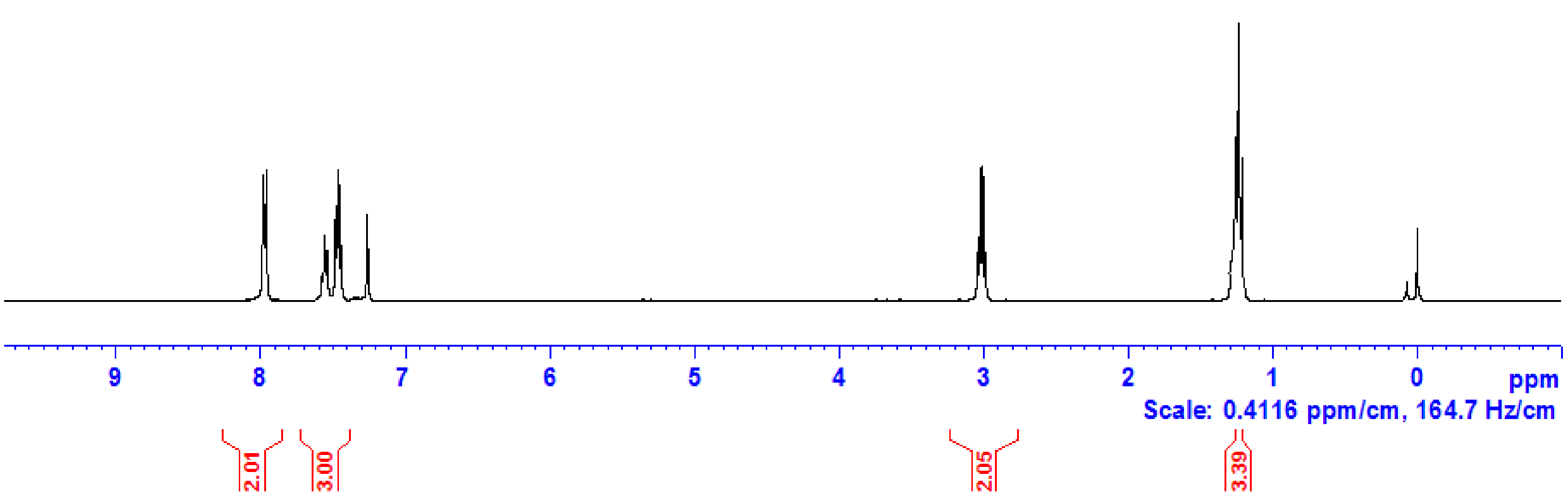

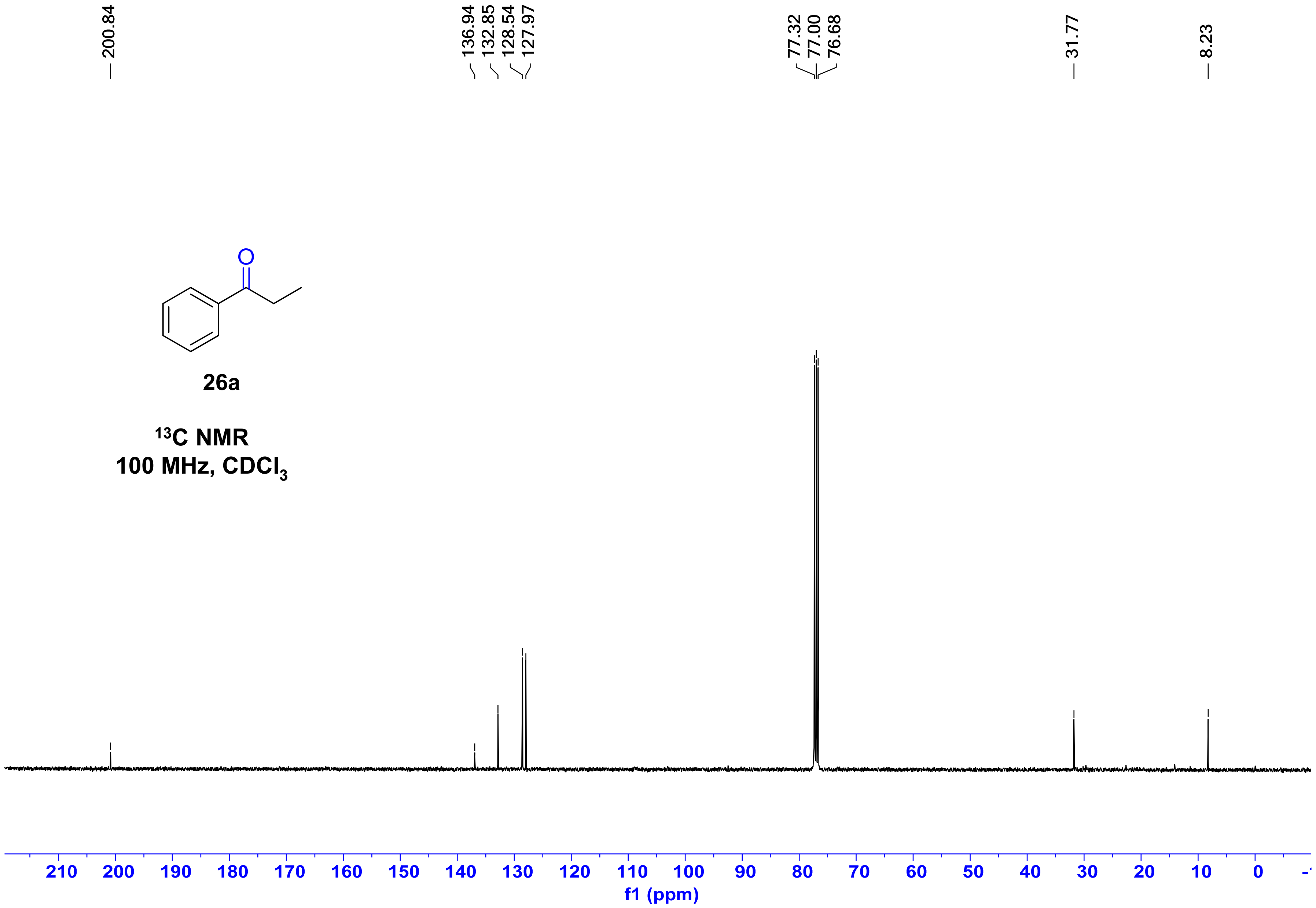

26a

^{13}C NMR
100 MHz, $CDCl_3$

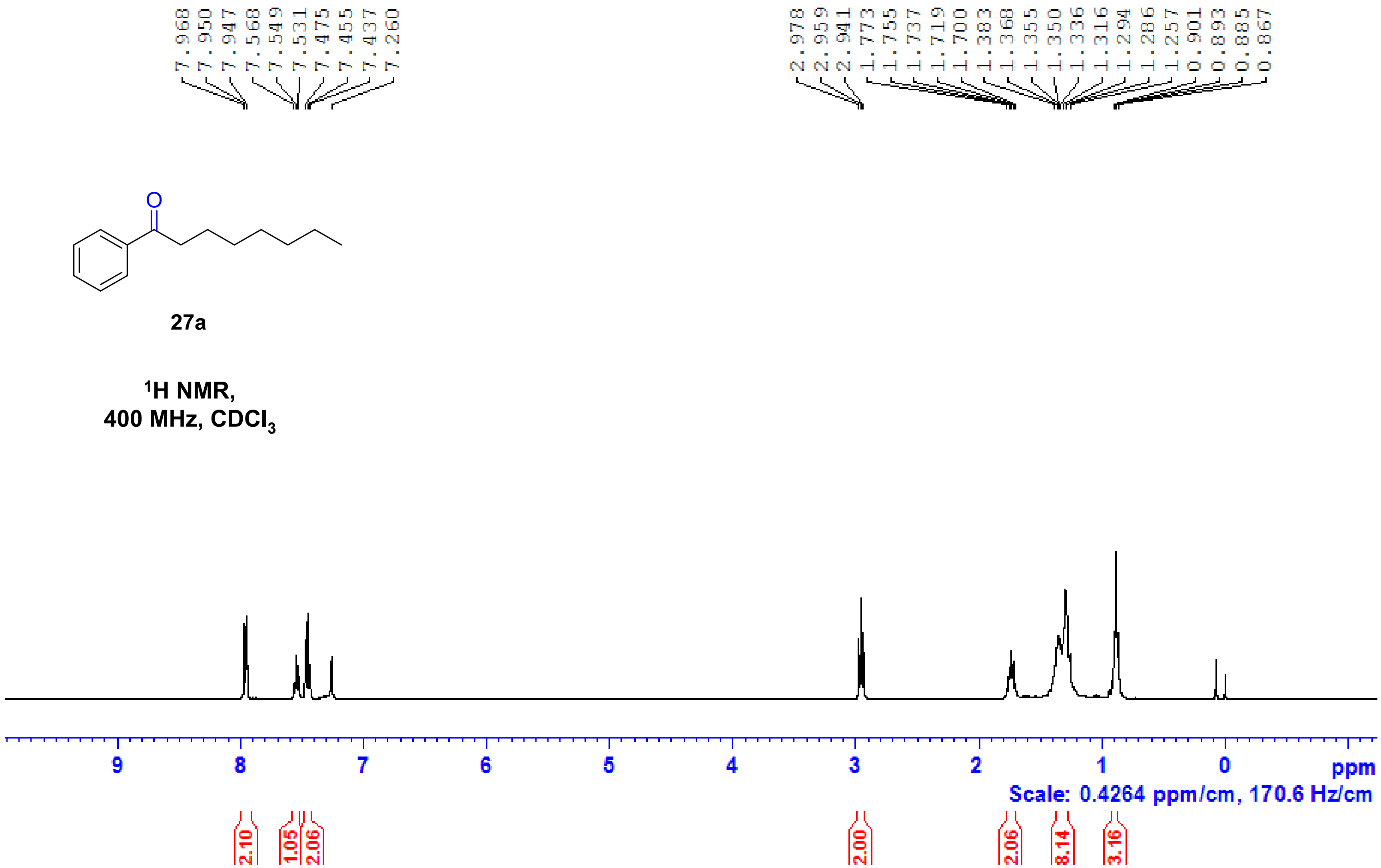
7.968
7.950
7.947
7.568
7.549
7.531
7.475
7.455
7.437
7.260
2.978
2.959
2.941
1.773
1.755
1.737
1.719
1.700
1.383
1.368
1.355
1.350
1.336
1.316
1.294
1.286
1.257
0.901
0.893
0.885
0.867
O
27a
1H NMR,
400 MHz, CDCl3
9
8
7
6
5
4
3
2
1
0
ppm
Scale: 0.4264 ppm/cm, 170.6 Hz/cm
2.10
1.05
2.06
2.00
2.06
8.14
3.16

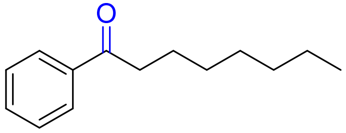

27a

^{13}C NMR
100 MHz, $CDCl_3$

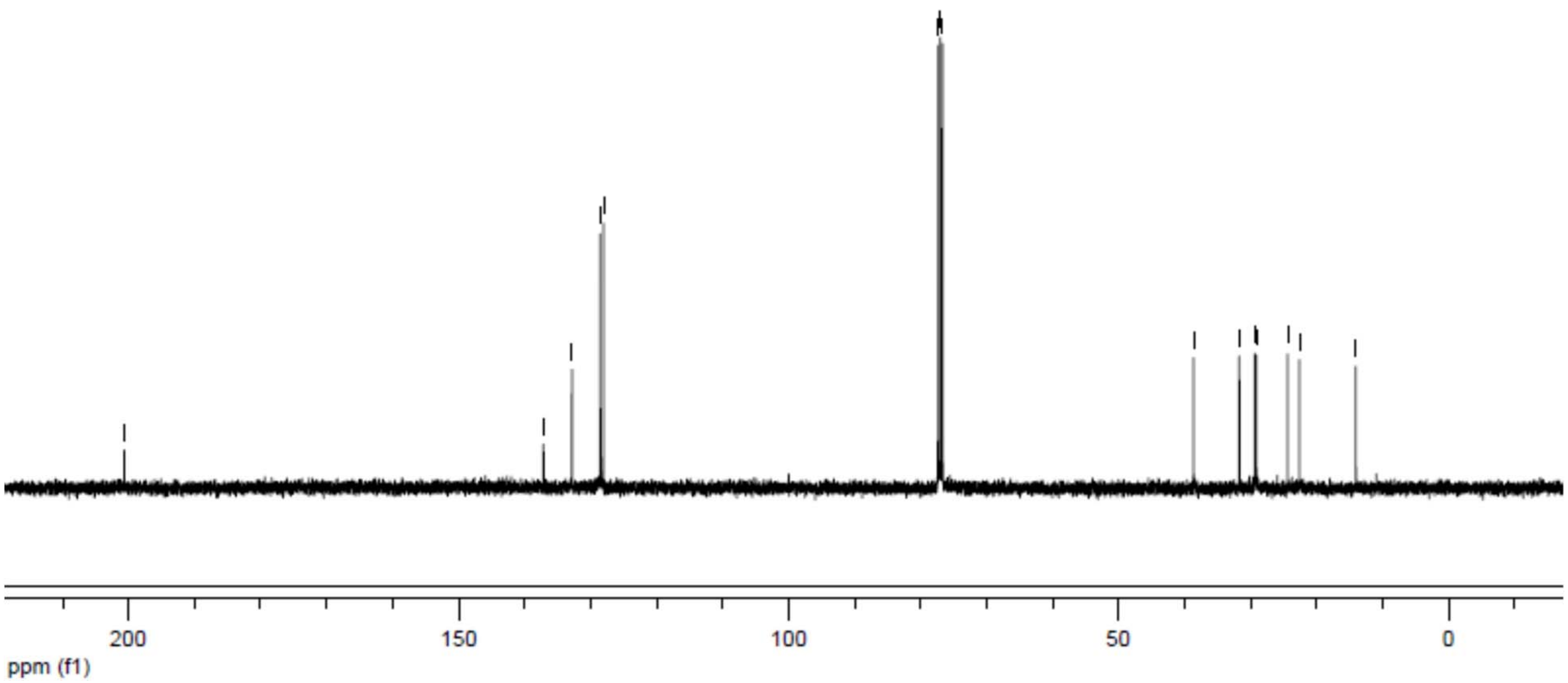

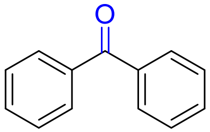

28a

^{1}H NMR,
400 MHz, $CDCl_3$

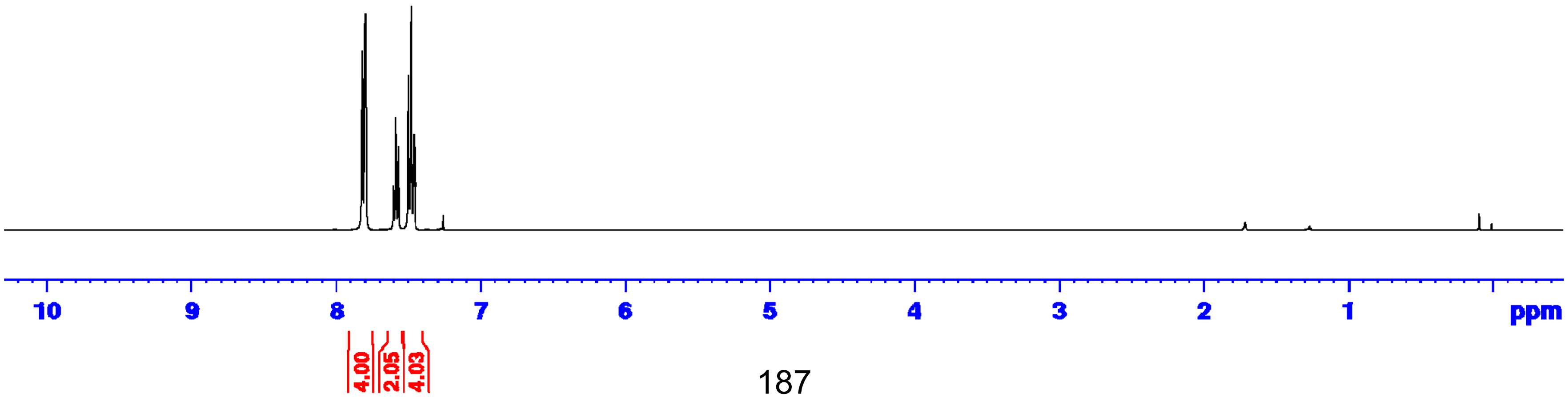

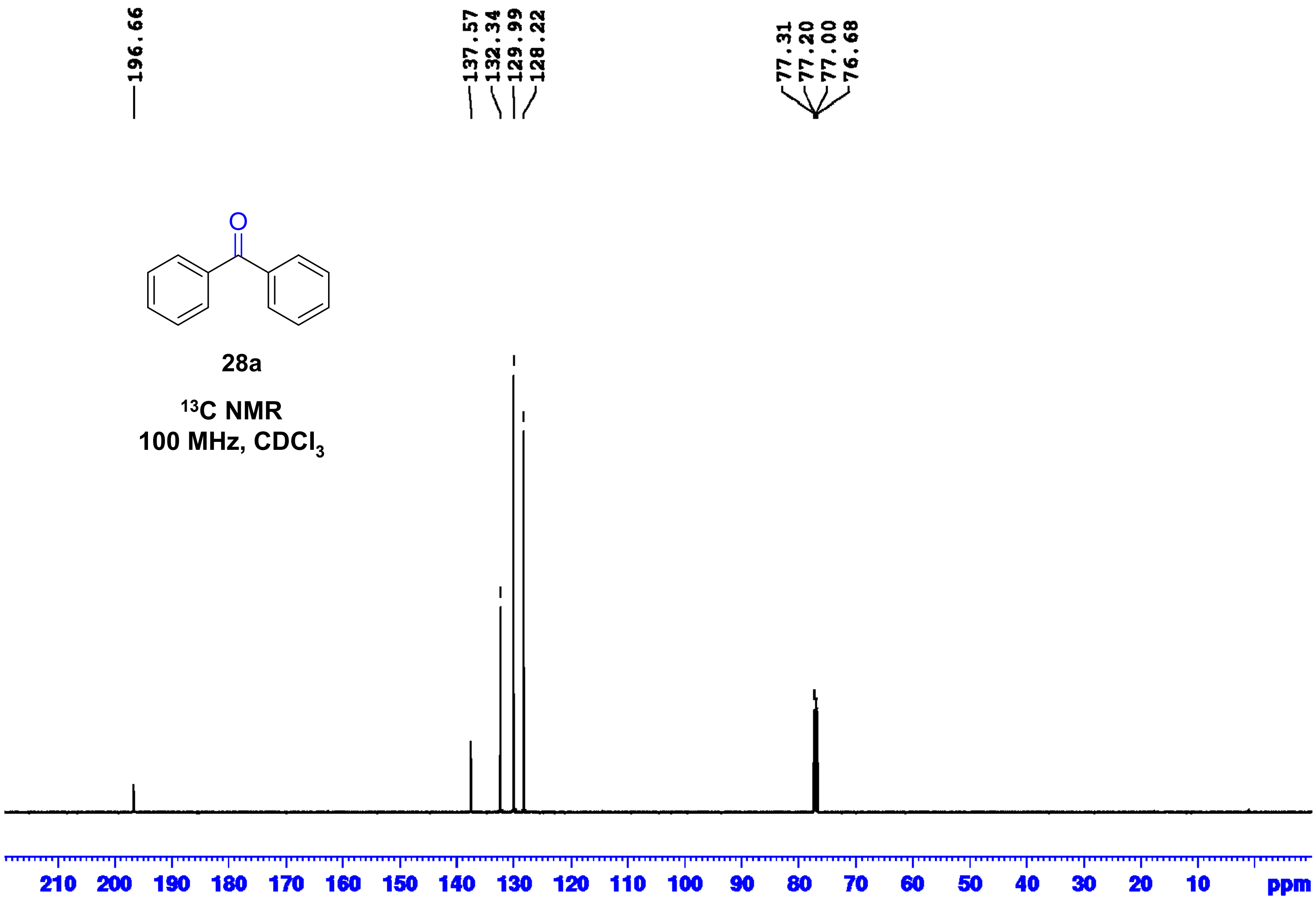
196.66
137.57
132.34
129.99
128.22
77.31
77.20
77.00
76.68
O
28a
13C NMR
100 MHz, CDCl3
210 200 190 180 170 160 150 140 130 120 110 100 90 80 70 60 50 40 30 20 10 ppm

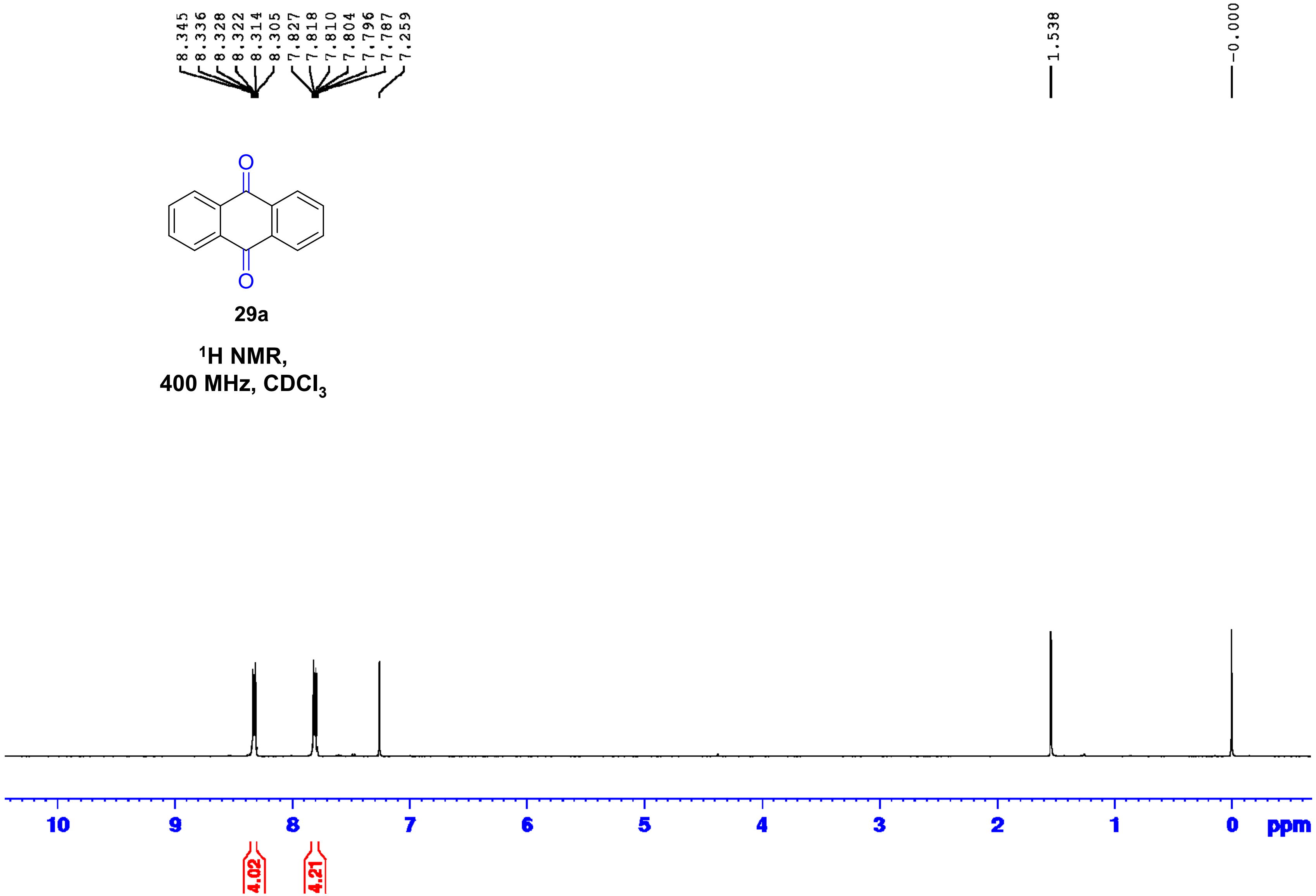
8.345
8.336
8.328
8.322
8.314
8.305
7.827
7.818
7.810
7.804
7.796
7.787
7.259
1.538
-0.000
29a
1H NMR,
400 MHz, CDCl3
10
9
8
7
6
5
4
3
2
1
0
ppm
4.02
4.21

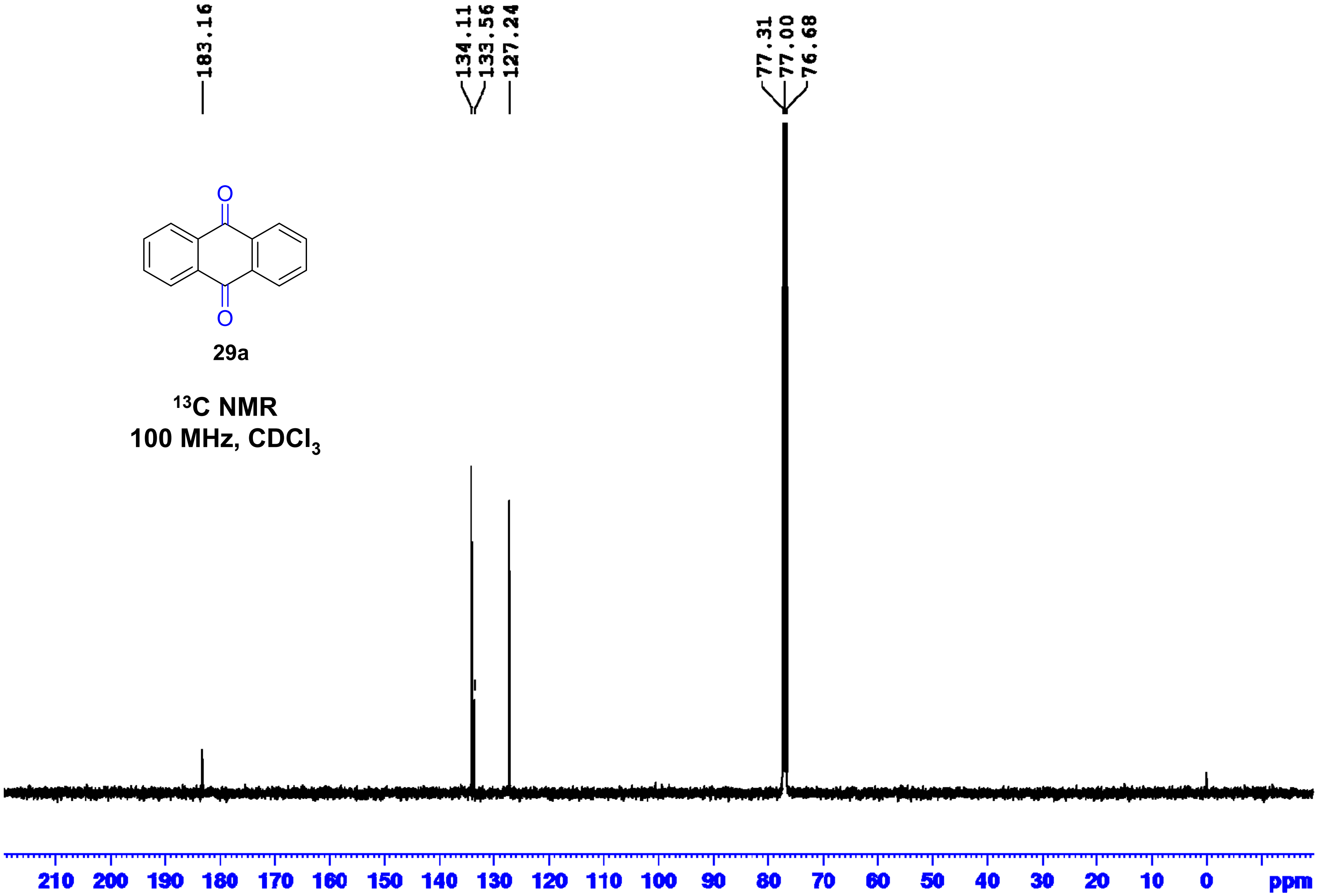
183.16
134.11
133.56
127.24
77.31
77.00
76.68
O
O
29a
^{13}C NMR
100 MHz, $CDCl_3$
210 200 190 180 170 160 150 140 130 120 110 100 90 80 70 60 50 40 30 20 10 0 ppm

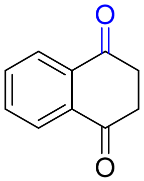

30a

^{1}H NMR,
400 MHz, $CDCl_3$

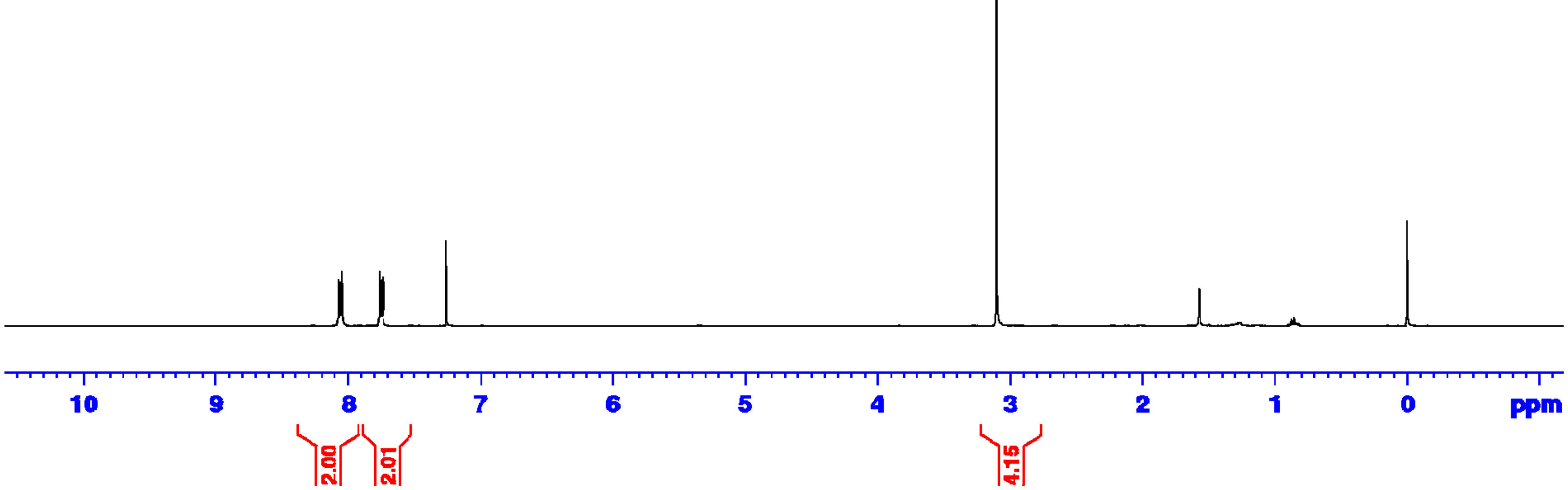

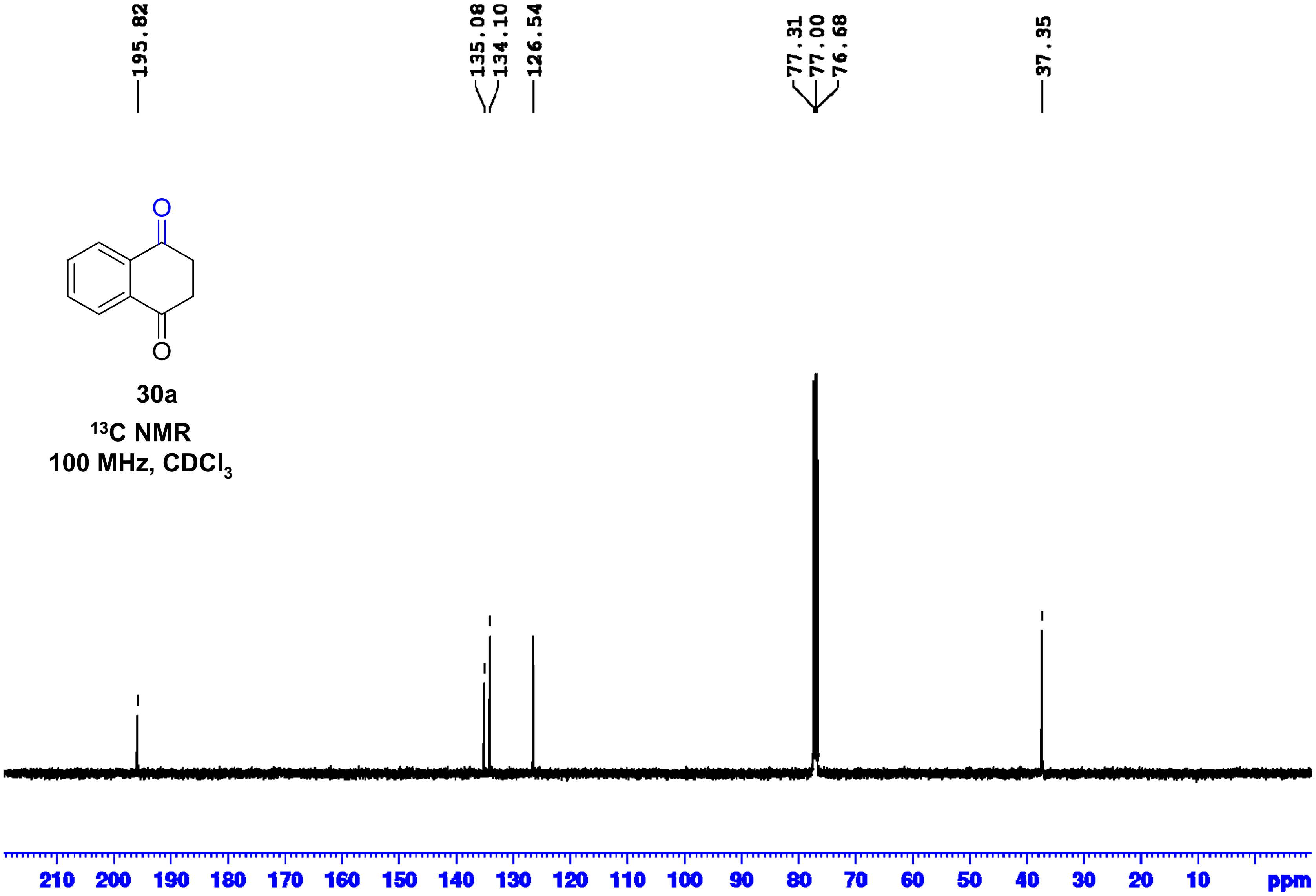
195.82
135.08
134.10
126.54
77.31
77.00
76.68
37.35
O
O
30a
13C NMR
100 MHz, CDCl3
210 200 190 180 170 160 150 140 130 120 110 100 90 80 70 60 50 40 30 20 10 ppm

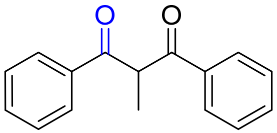

31a

^{1}H NMR,
400 MHz, $CDCl_3$

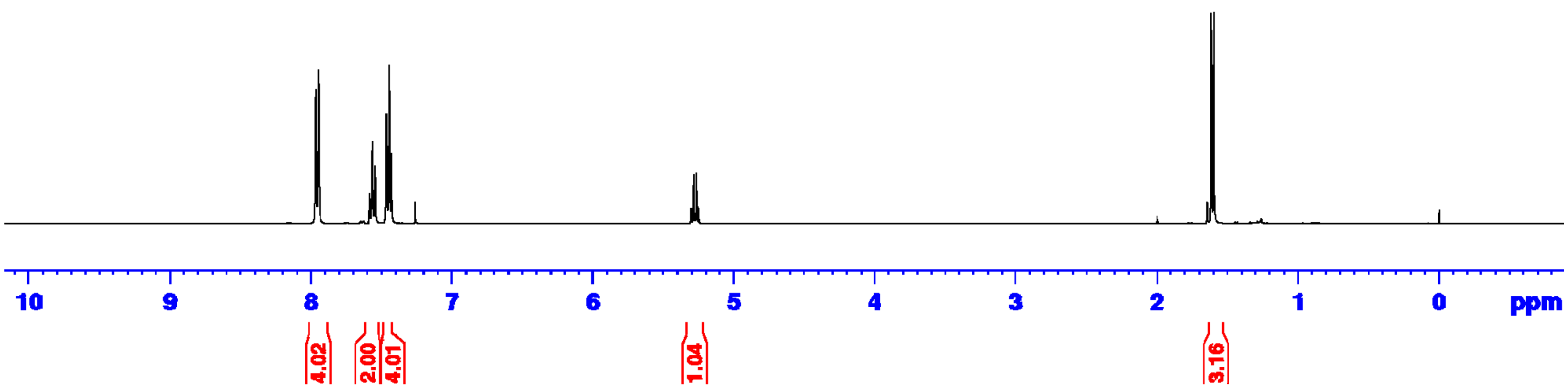

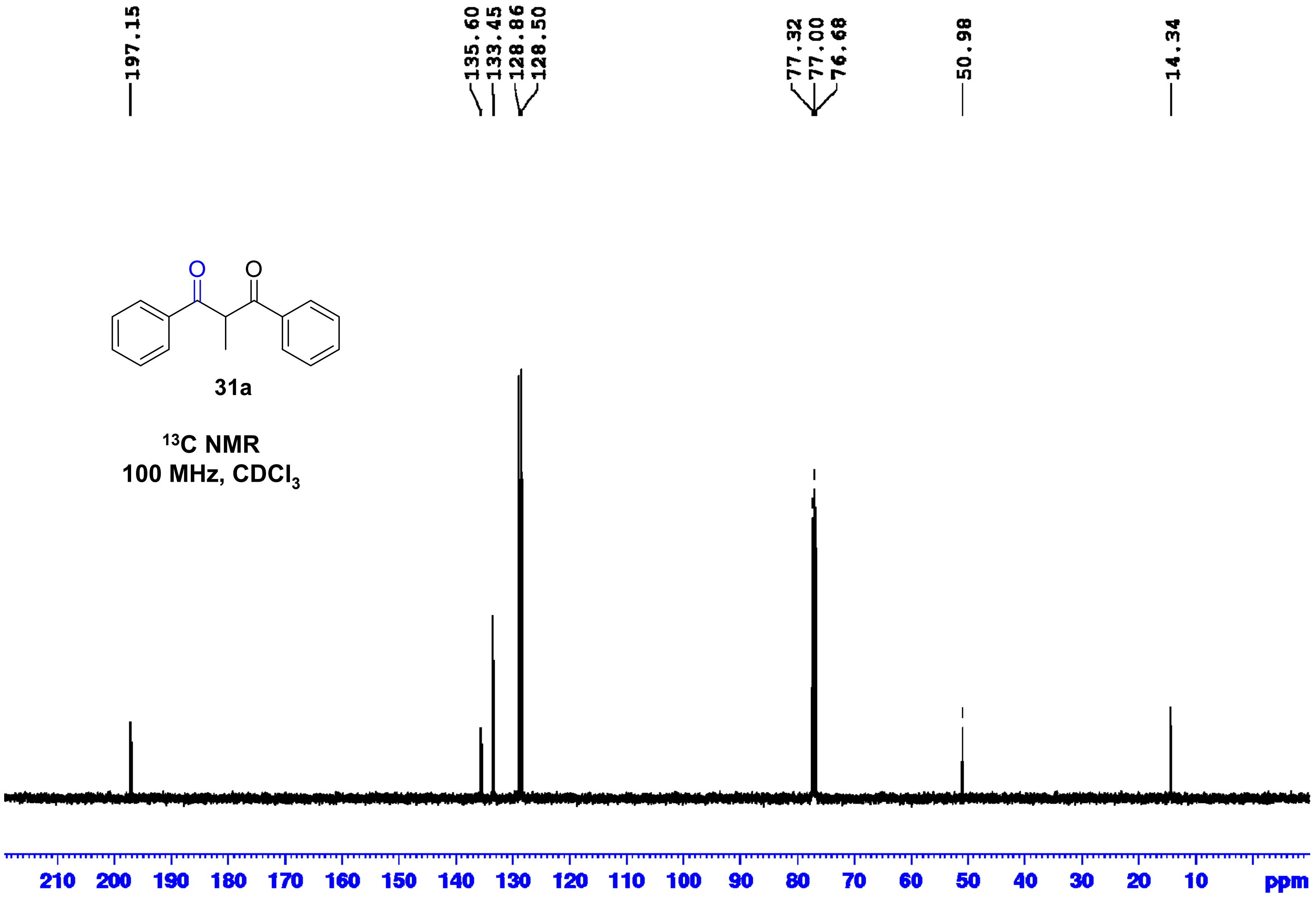
197.15
135.60
133.45
128.86
128.50
77.32
77.00
76.68
50.98
14.34
O
O
31a
^{13}C NMR
100 MHz, $CDCl_3$
210 200 190 180 170 160 150 140 130 120 110 100 90 80 70 60 50 40 30 20 10 ppm

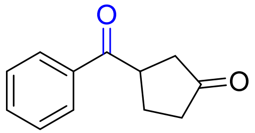

32a

^{1}H NMR,
400 MHz, $CDCl_3$

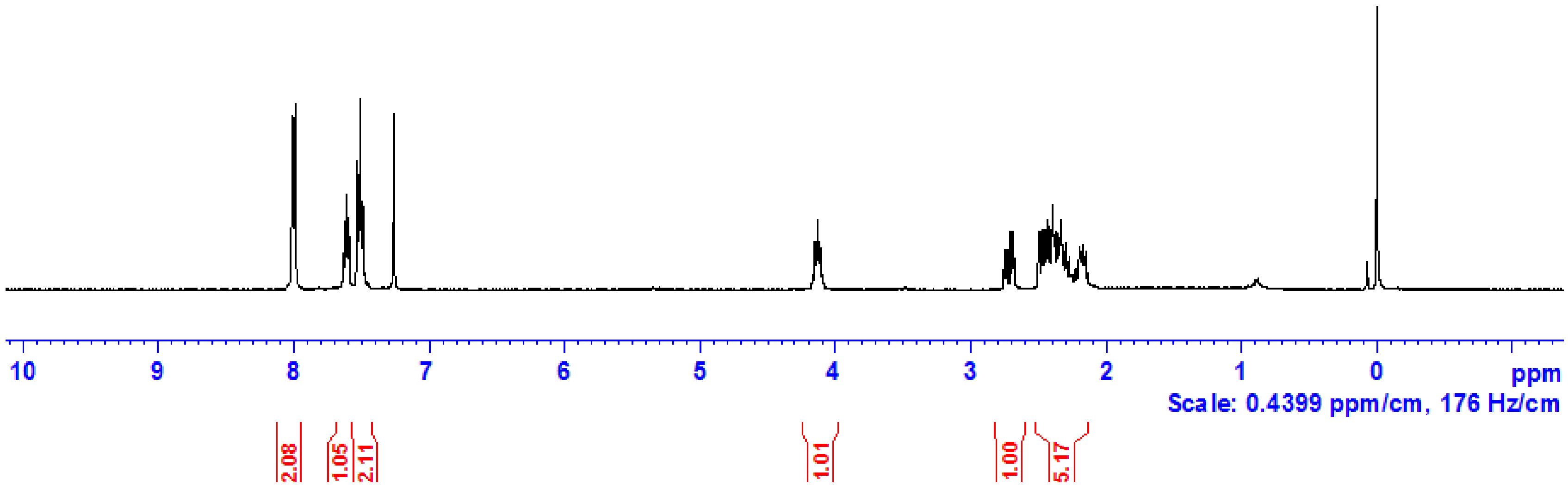

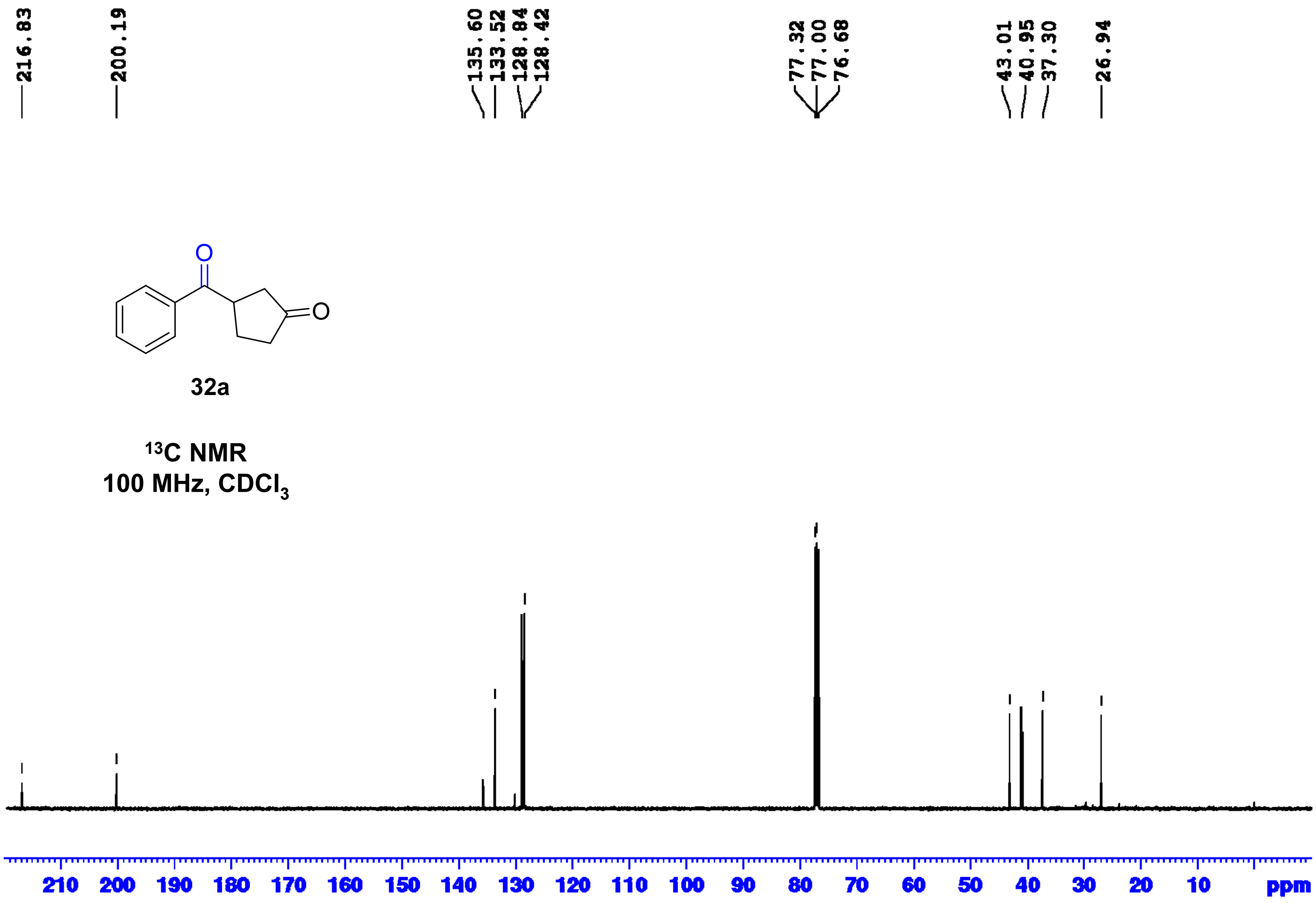
216.83
200.19
135.60
133.52
128.84
128.42
77.32
77.00
76.68
43.01
40.95
37.30
26.94
O
O
32a
13C NMR
100 MHz, CDCl3
210 200 190 180 170 160 150 140 130 120 110 100 90 80 70 60 50 40 30 20 10 ppm

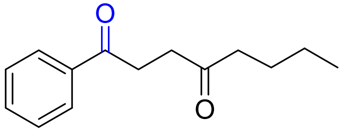

33a

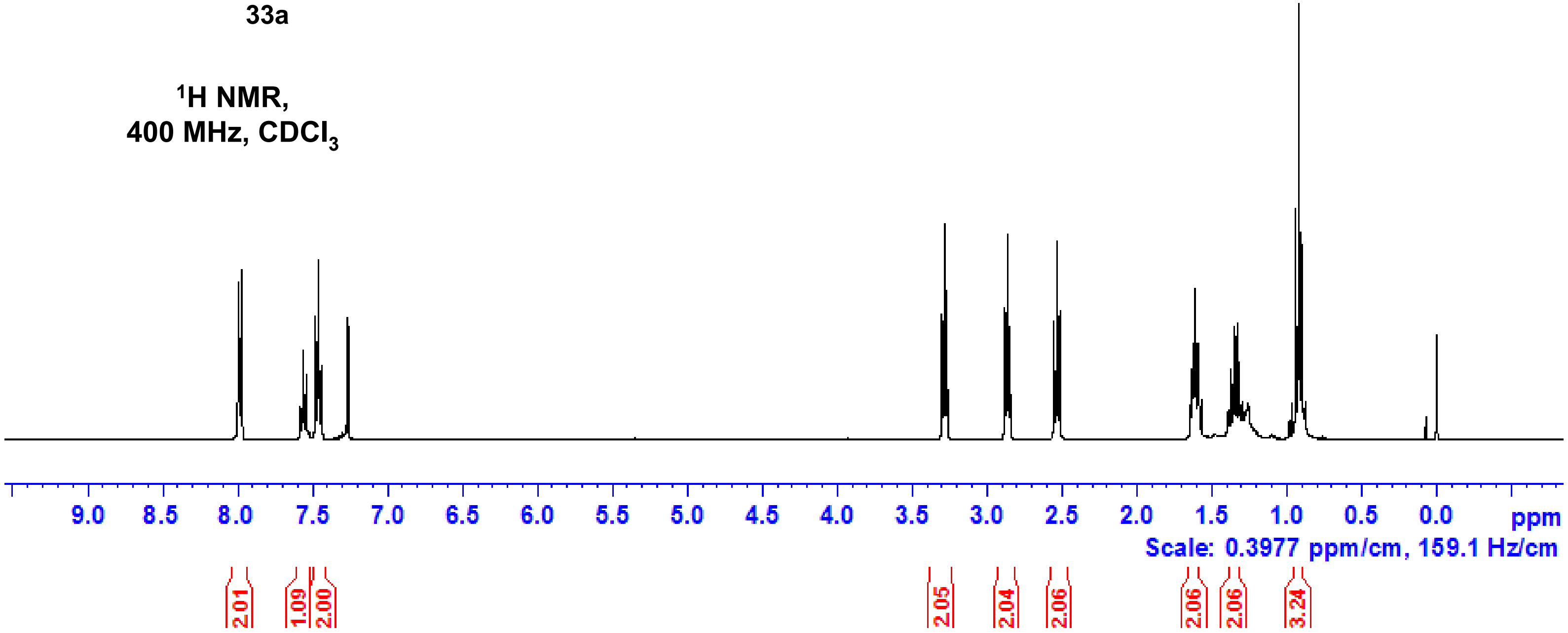

7.994
7.976
7.973
7.581
7.562
7.544
7.479
7.460
7.442
7.263
3.299
3.284
3.268
2.878
2.862
2.847
2.552
2.534
2.515
1.646
1.627
1.622
1.608
1.589
1.570
1.389
1.371
1.352
1.333
1.315
1.297
0.980
0.961
0.939
0.920
0.902
0.896
0.887
0.878
^{1}H NMR,
400 MHz, $CDCl_3$
9.0 8.5 8.0 7.5 7.0 6.5 6.0 5.5 5.0 4.5 4.0 3.5 3.0 2.5 2.0 1.5 1.0 0.5 0.0 ppm
Scale: 0.3977 ppm/cm, 159.1 Hz/cm
2.01
1.09
2.00
2.05
2.04
2.06
2.06
2.06
3.24

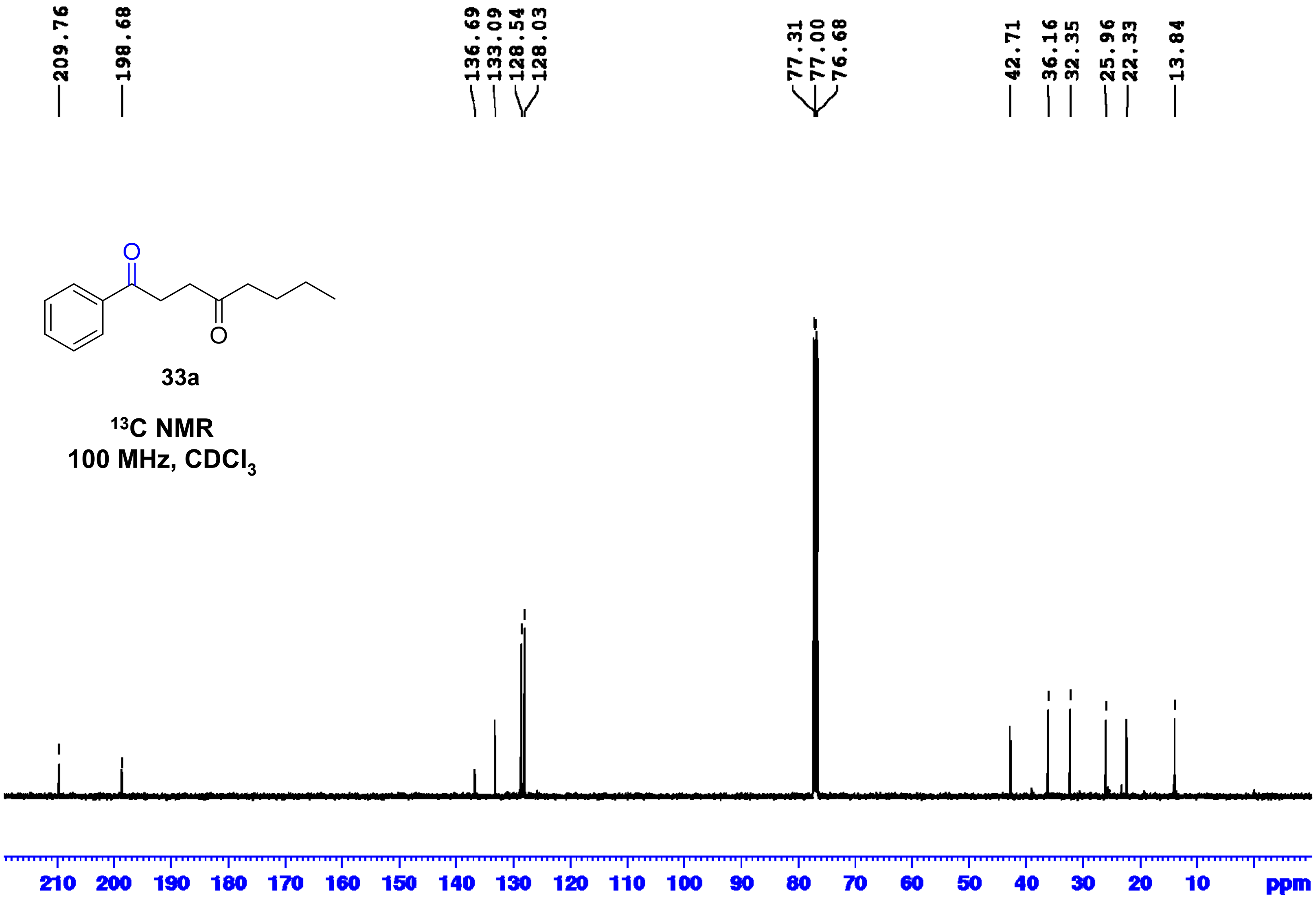

33a

^{13}C NMR
100 MHz, $CDCl_3$

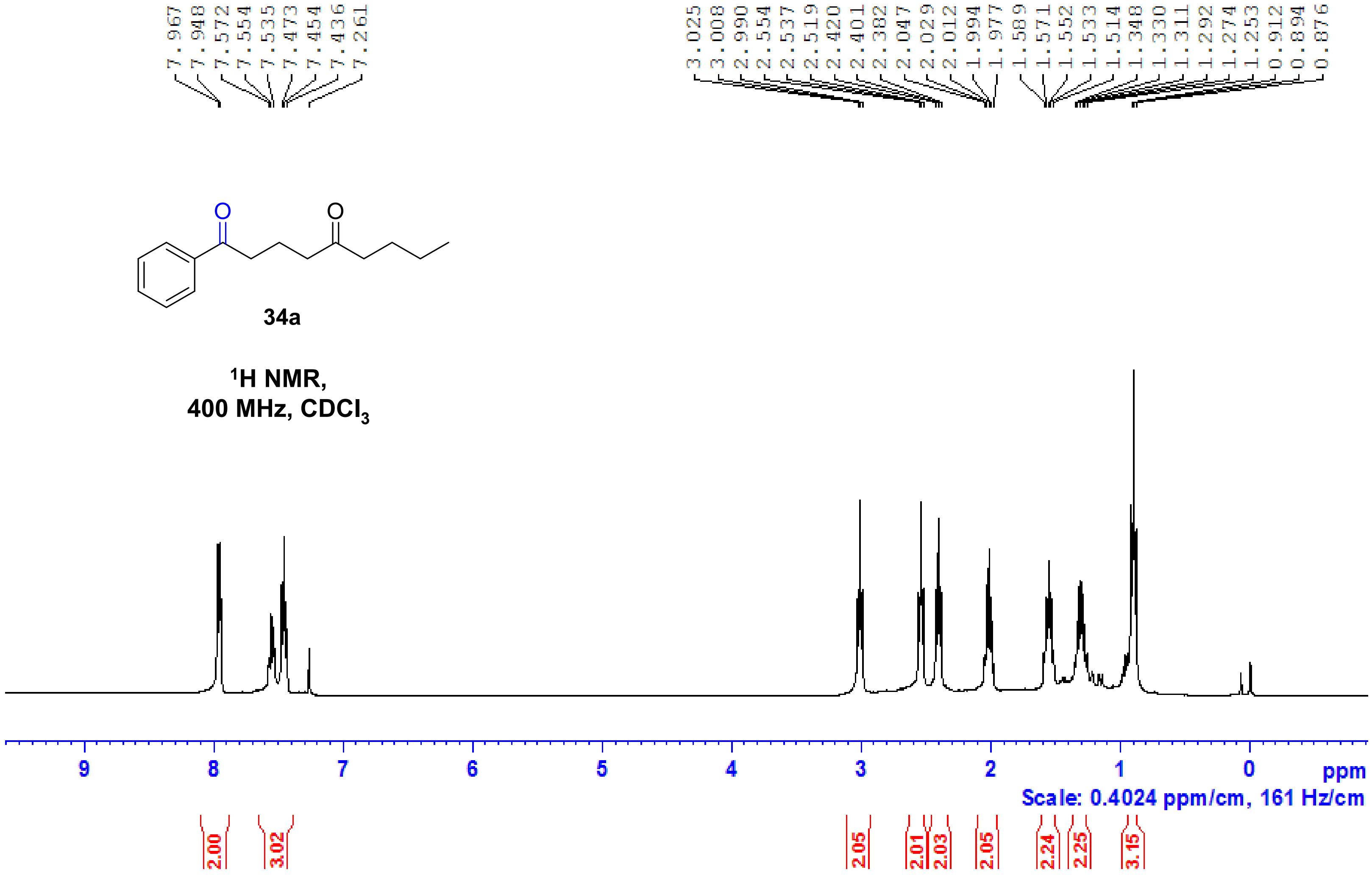

7.967
7.948
7.572
7.554
7.535
7.473
7.454
7.436
7.261
3.025
3.008
2.990
2.554
2.537
2.519
2.420
2.401
2.382
2.047
2.029
2.012
1.994
1.977
1.589
1.571
1.552
1.533
1.514
1.348
1.330
1.311
1.292
1.274
1.253
0.912
0.894
0.876
O
O
34a
^{1}H NMR,
400 MHz, $CDCl_3$
9
8
7
6
5
4
3
2
1
0
ppm
Scale: 0.4024 ppm/cm, 161 Hz/cm
2.00
3.02
2.05
2.01
2.03
2.05
2.24
2.25
3.15

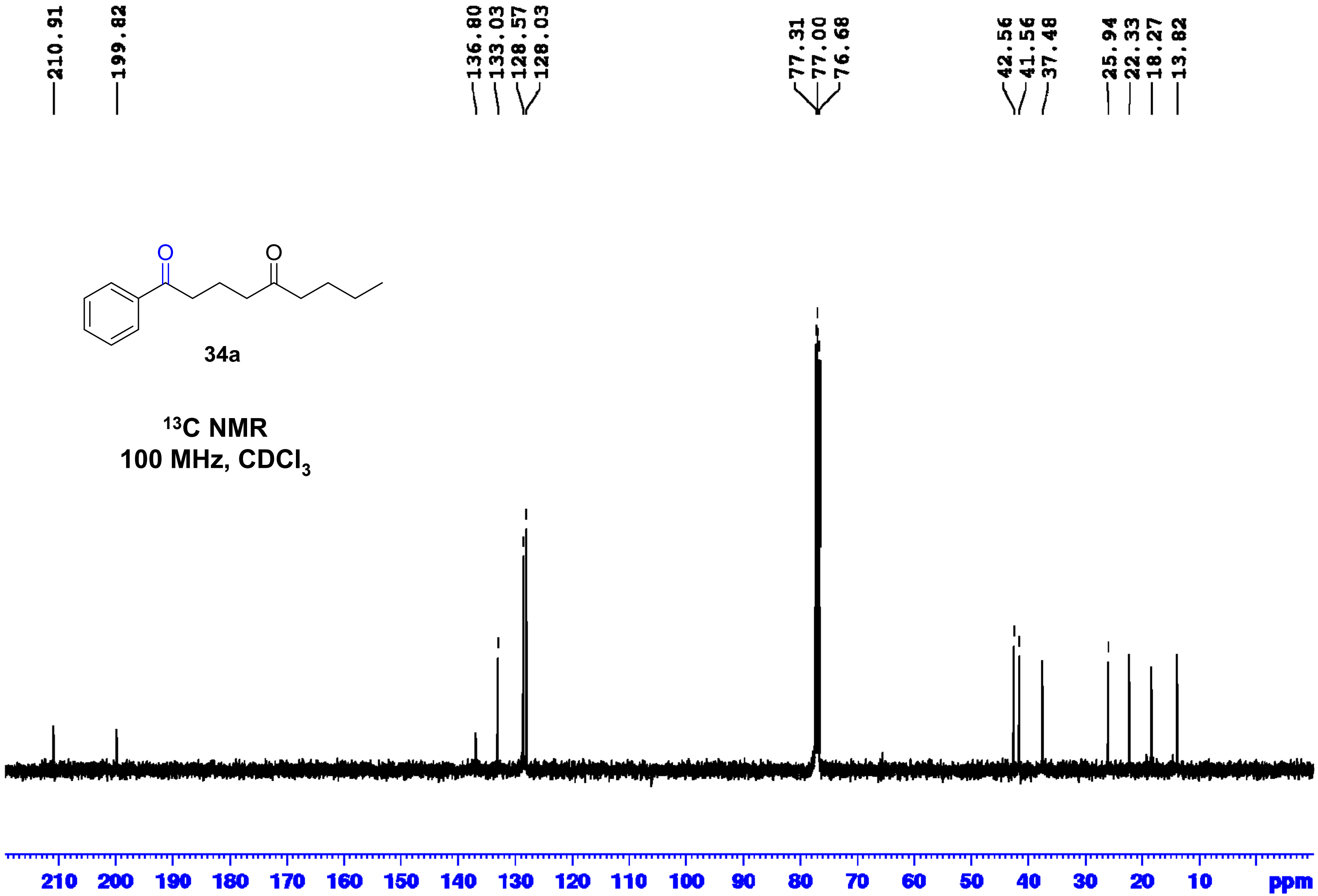

34a

^{13}C NMR
100 MHz, $CDCl_3$

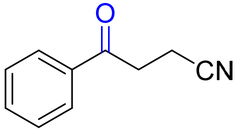

35a

^{1}H NMR,
400 MHz, $CDCl_3$

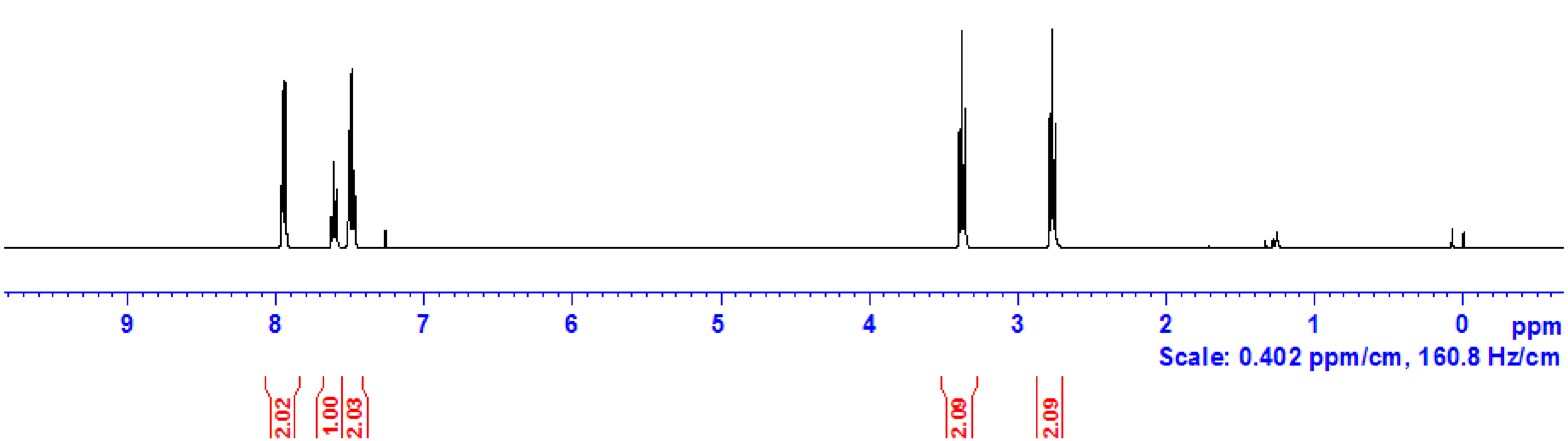

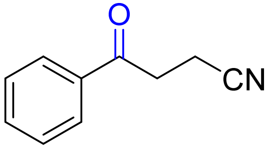

35a

^{13}C NMR
100 MHz, $CDCl_3$

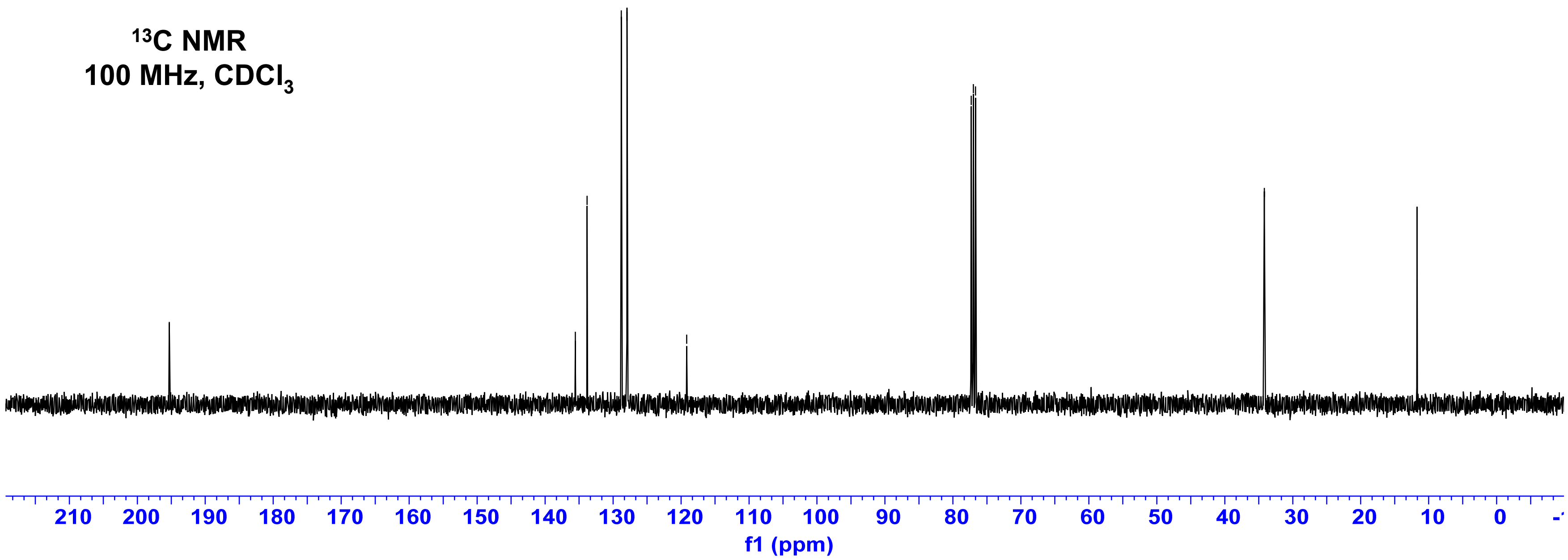

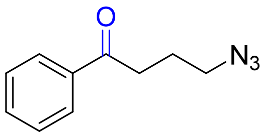

36a

^{1}H NMR,
400 MHz, $CDCl_3$

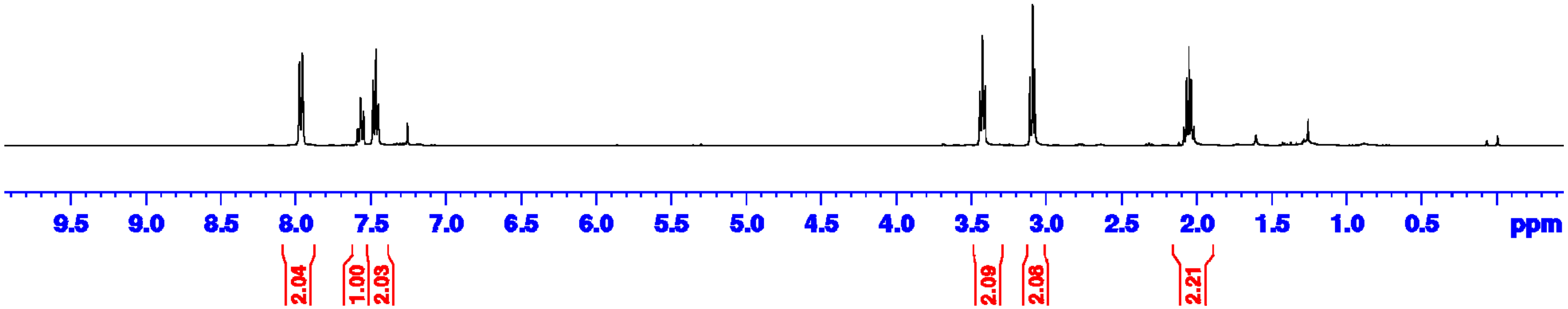

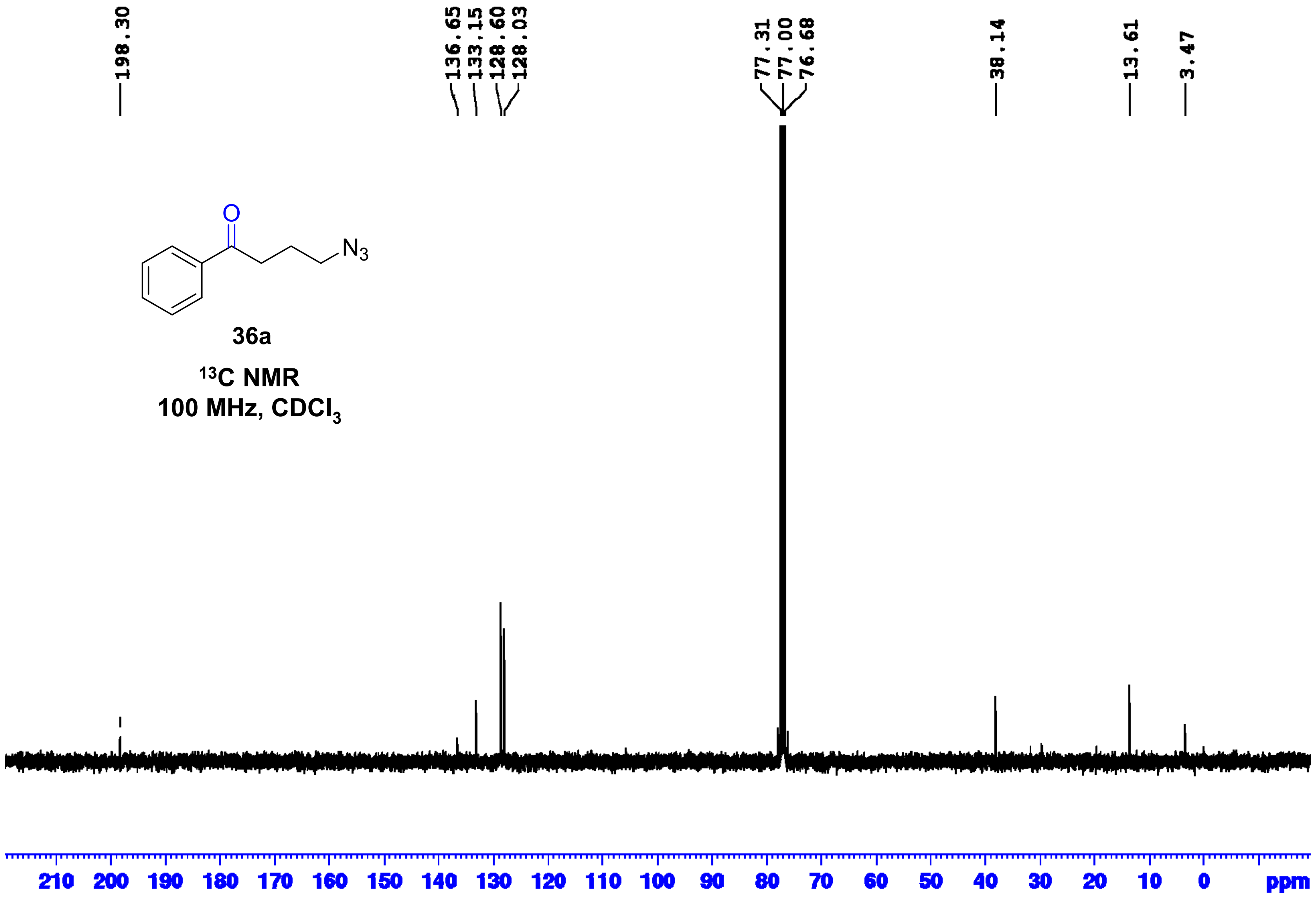
198.30
136.65
133.15
128.60
128.03
77.31
77.00
76.68
38.14
13.61
3.47
O
N_3
36a
^{13}C NMR
100 MHz, $CDCl_3$
210 200 190 180 170 160 150 140 130 120 110 100 90 80 70 60 50 40 30 20 10 0 ppm

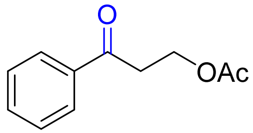

37a

^{1}H NMR, 400 MHz, $CDCl_3$

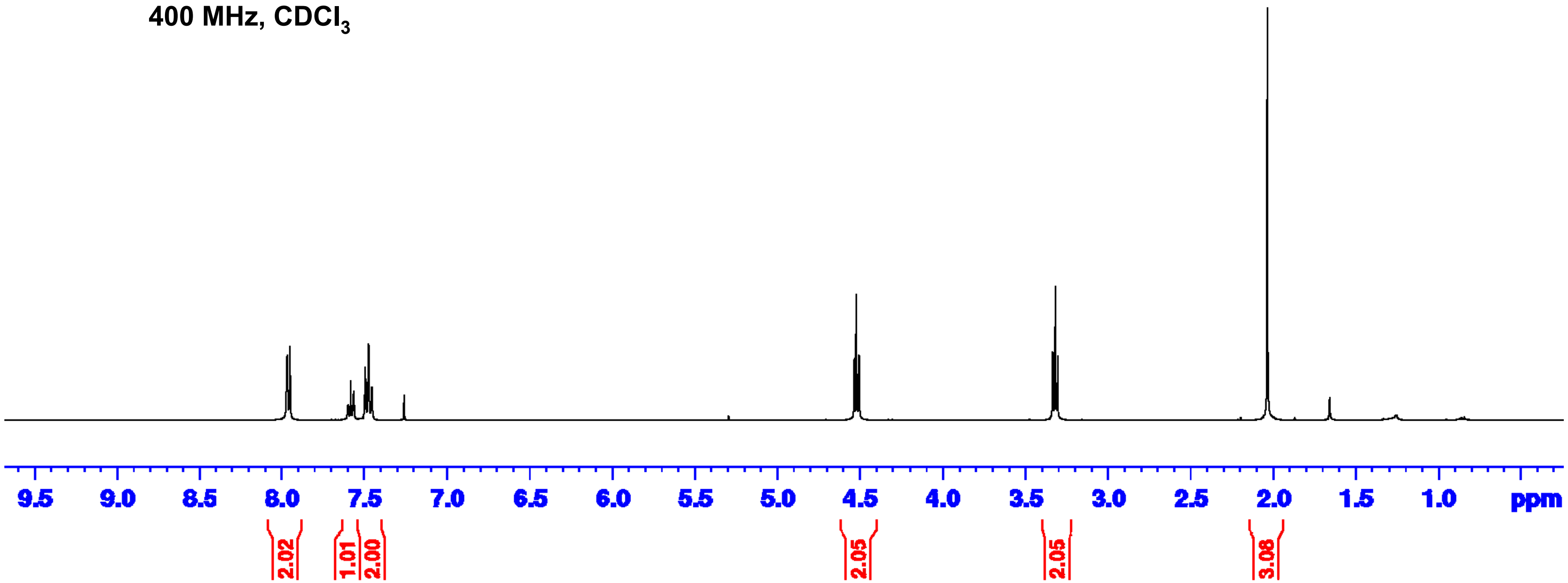

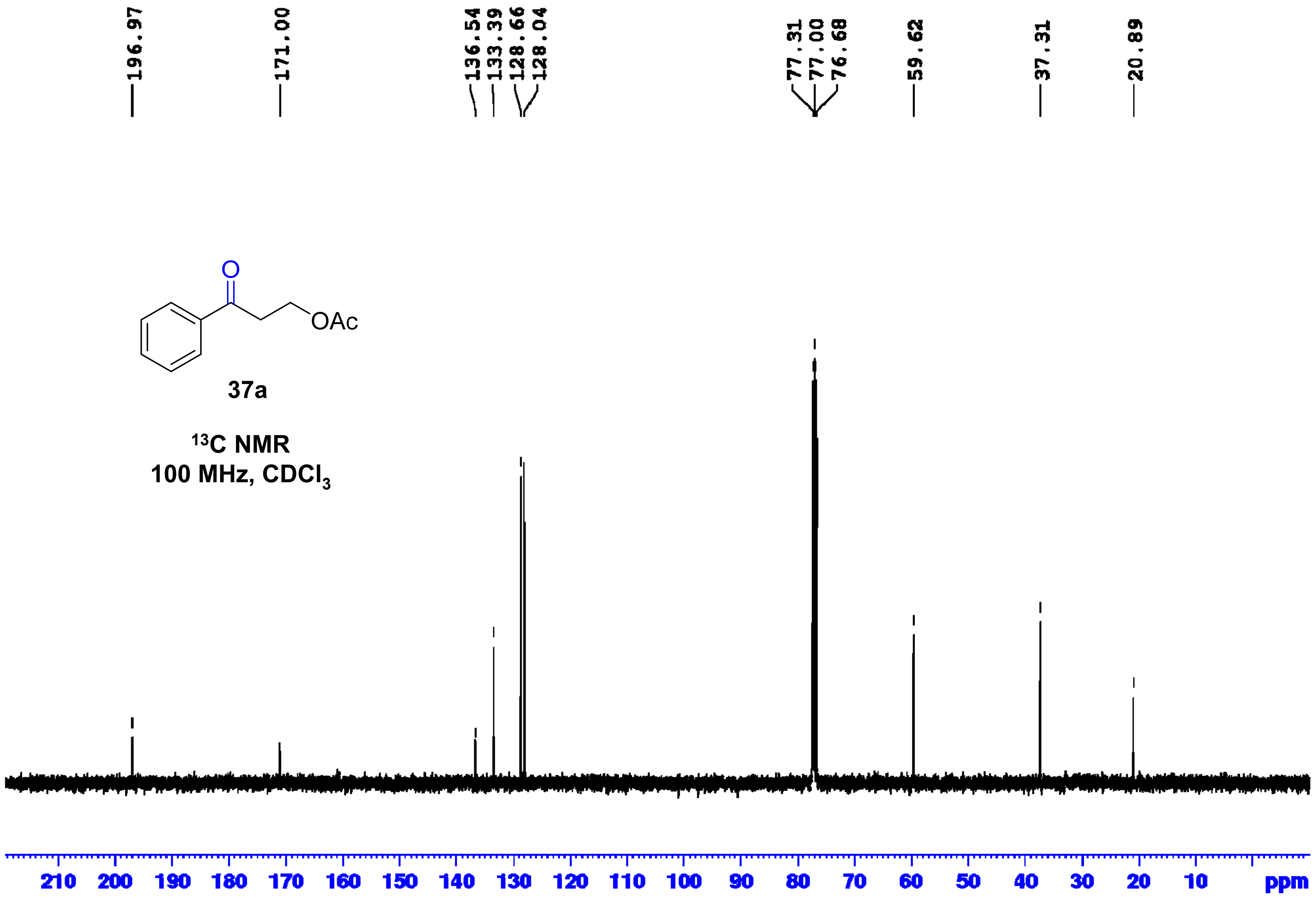
196.97
171.00
136.54
133.39
128.66
128.04
77.31
77.00
76.68
59.62
37.31
20.89
O
OAc
37a
13C NMR
100 MHz, CDCl3
210 200 190 180 170 160 150 140 130 120 110 100 90 80 70 60 50 40 30 20 10 ppm

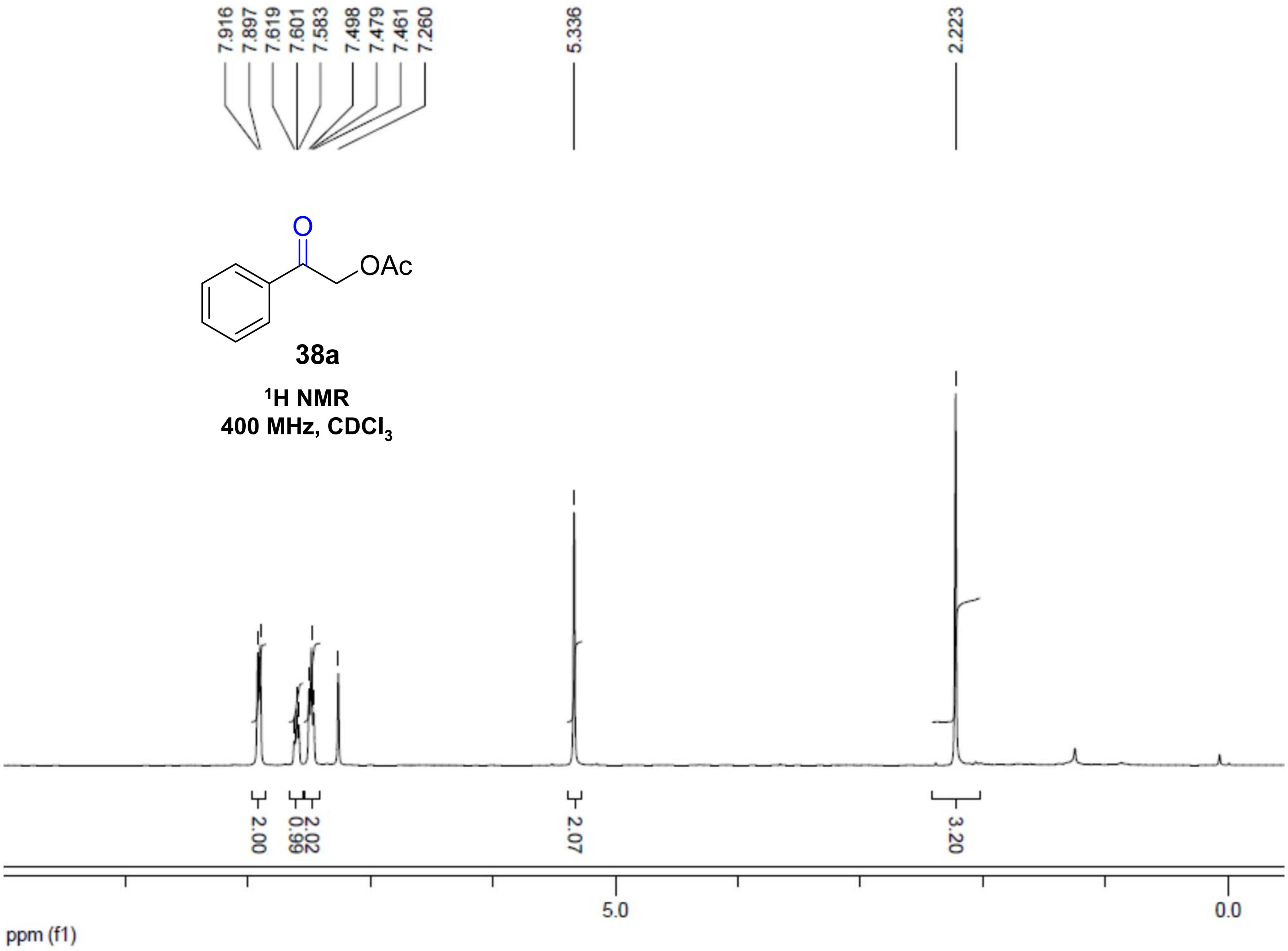

7.916
7.897
7.619
7.601
7.583
7.498
7.479
7.461
7.260
5.336
2.223
O
OAc
38a
1H NMR
400 MHz, CDCl3
2.00
0.99
2.02
2.07
3.20
5.0
0.0
ppm (f1)

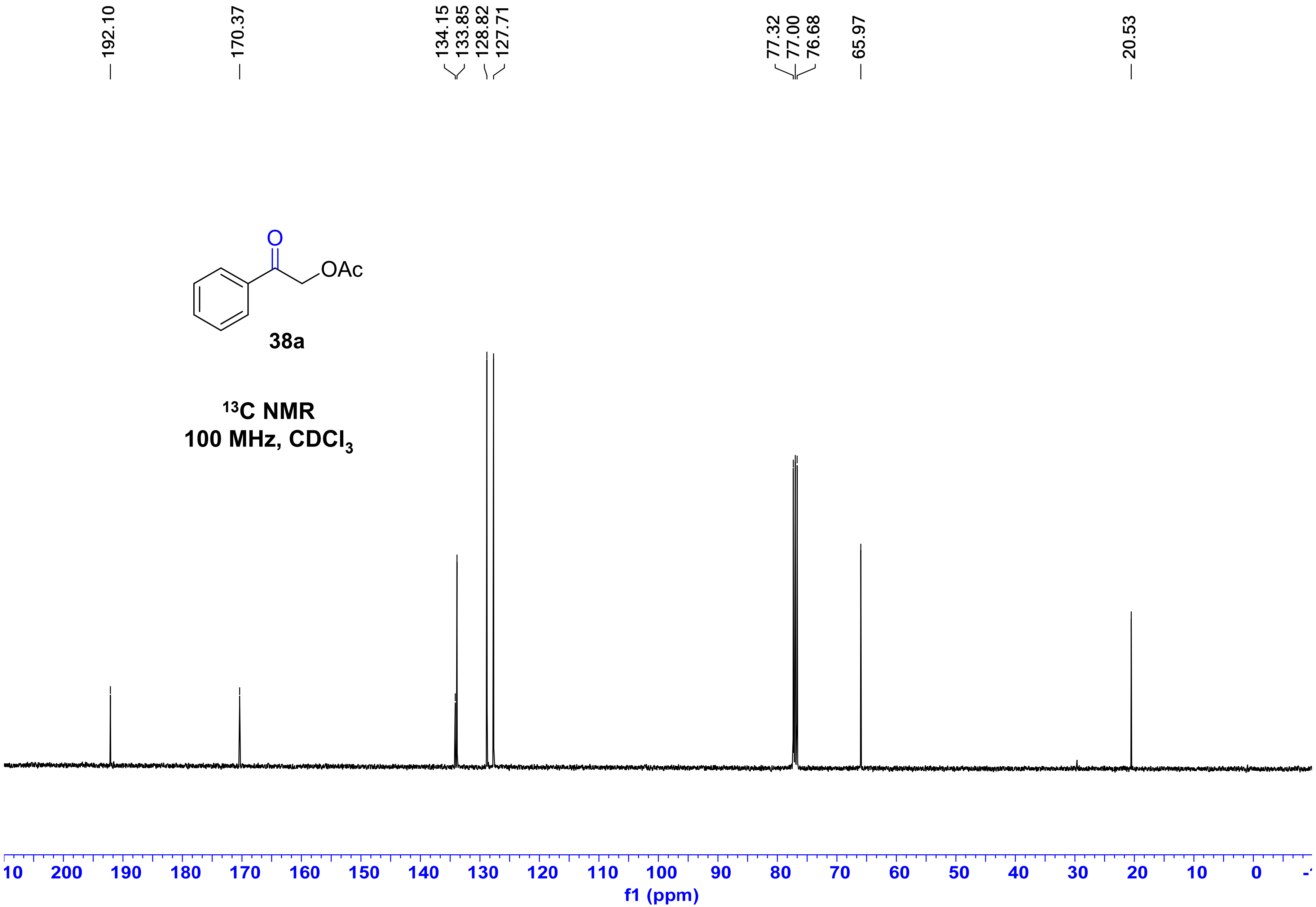

192.10
170.37
134.15
133.85
128.82
127.71
77.32
77.00
76.68
65.97
20.53
O
OAc
38a
^{13}C NMR
100 MHz, $CDCl_3$
f1 (ppm)

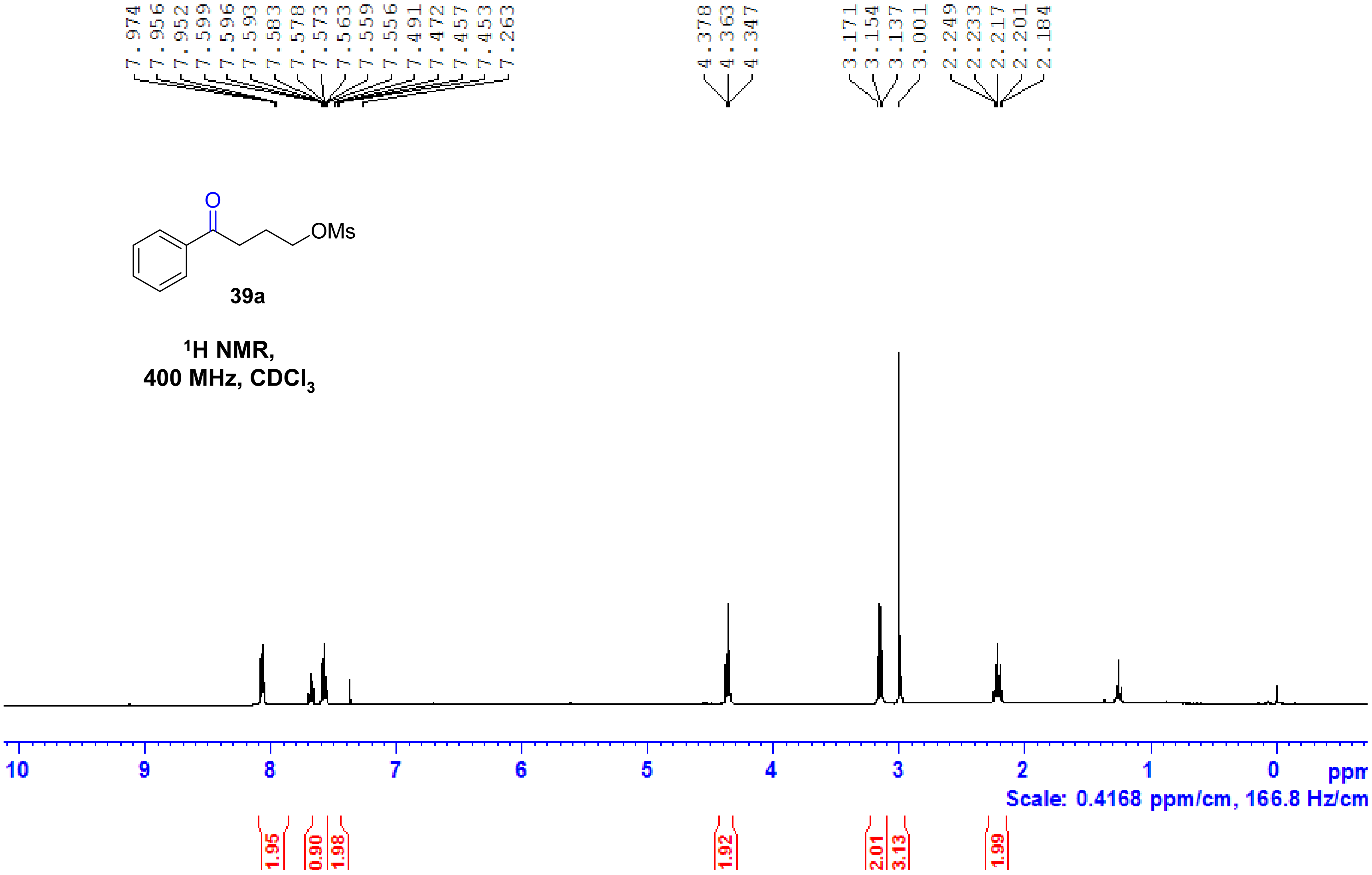
7.974
7.956
7.952
7.599
7.596
7.593
7.583
7.578
7.573
7.563
7.559
7.556
7.491
7.472
7.457
7.453
7.263
4.378
4.363
4.347
3.171
3.154
3.137
3.001
2.249
2.233
2.217
2.201
2.184
O
OMs
39a
^{1}H NMR,
400 MHz, $CDCl_3$
10
9
8
7
6
5
4
3
2
1
0
ppm
Scale: 0.4168 ppm/cm, 166.8 Hz/cm
1.95
0.90
1.98
1.92
2.01
3.13
1.99

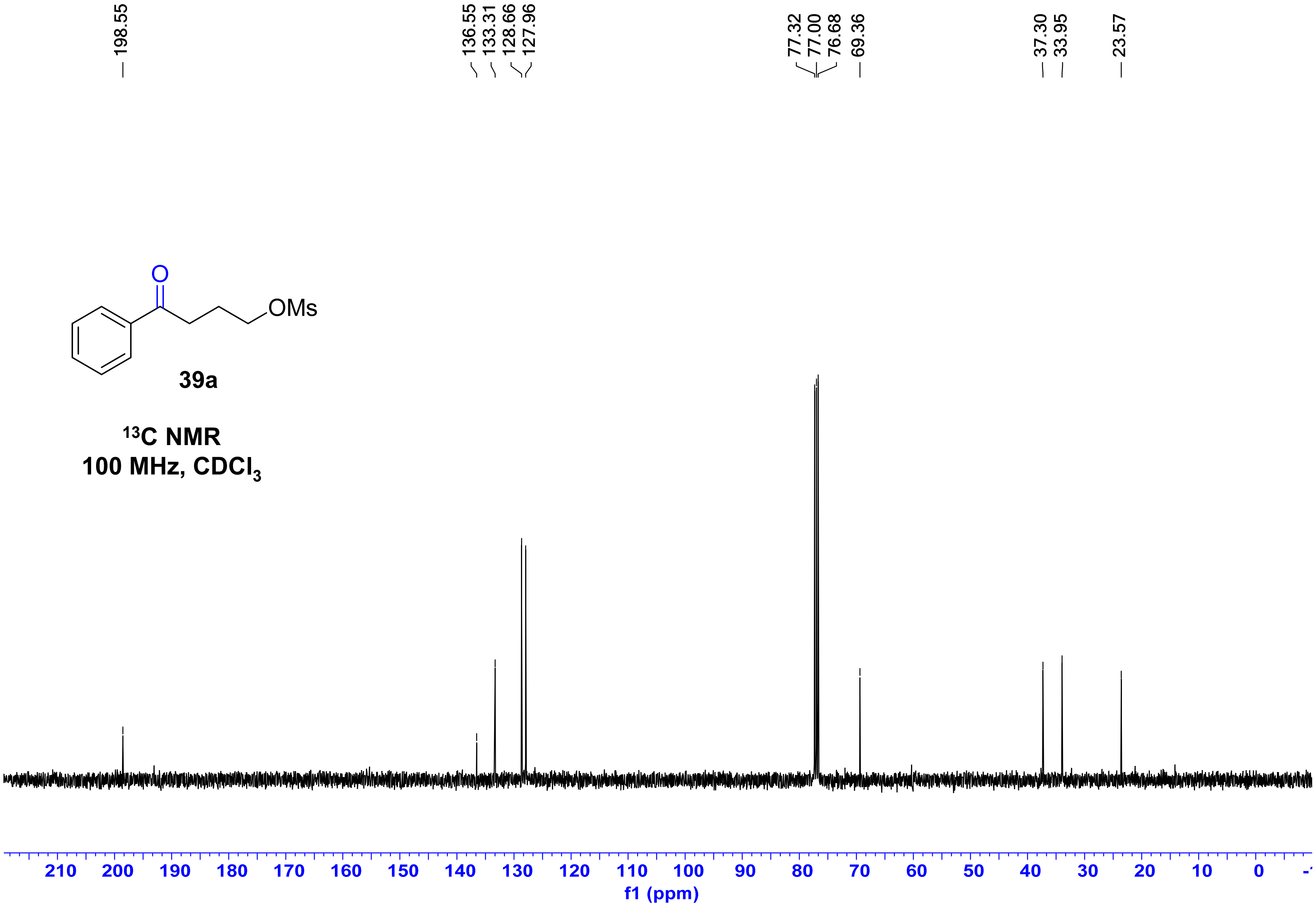
198.55
136.55
133.31
128.66
127.96
77.32
77.00
76.68
69.36
37.30
33.95
23.57
O
OMs
39a
13C NMR
100 MHz, CDCl3
210
200
190
180
170
160
150
140
130
120
110
100
90
80
70
60
50
40
30
20
10
0
f1 (ppm)

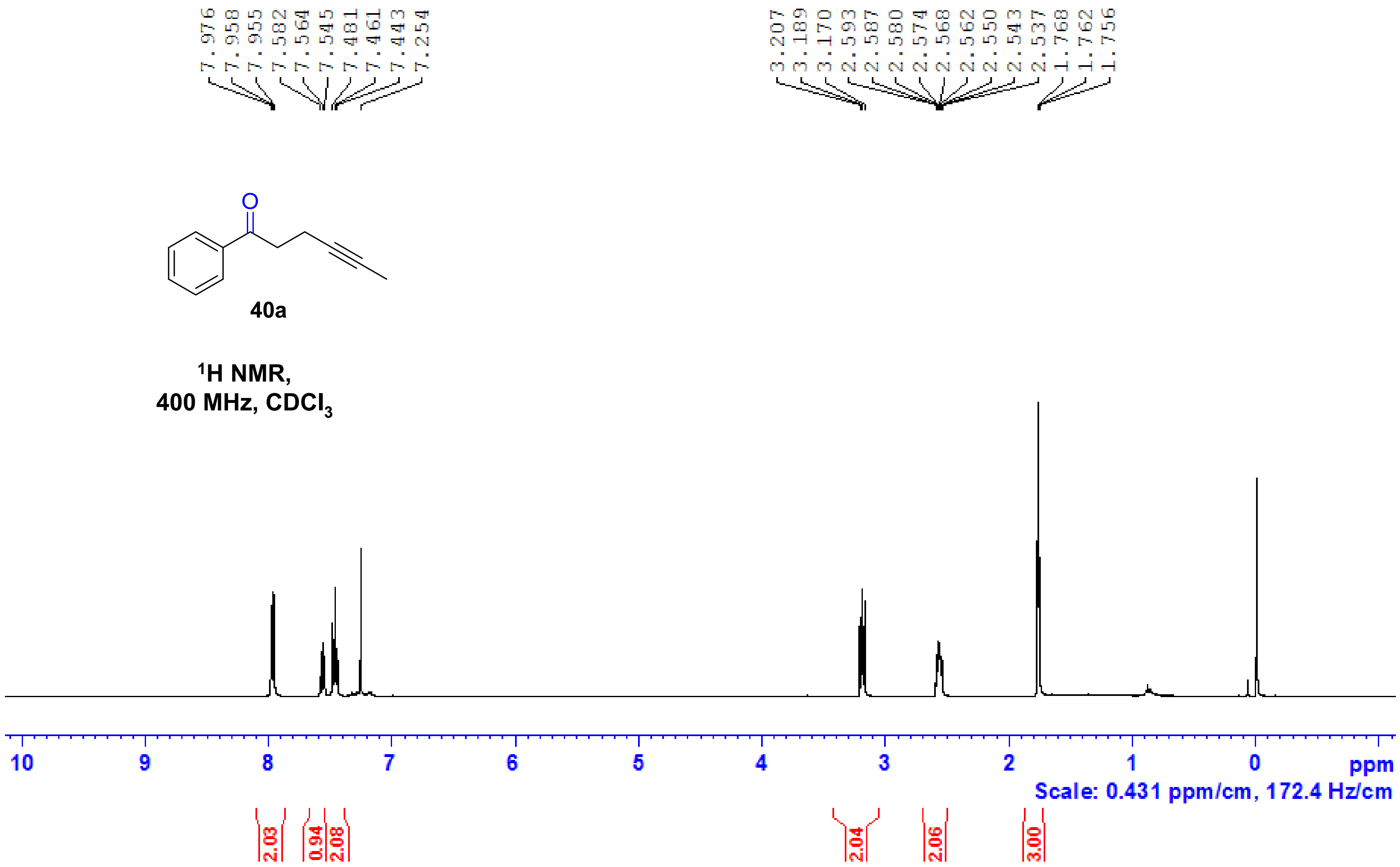
7.976
7.958
7.955
7.582
7.564
7.545
7.481
7.461
7.443
7.254
3.207
3.189
3.170
2.593
2.587
2.580
2.574
2.568
2.562
2.550
2.543
2.537
1.768
1.762
1.756
O
40a
^{1}H NMR,
400 MHz, $CDCl_3$
10
9
8
7
6
5
4
3
2
1
0
ppm
Scale: 0.431 ppm/cm, 172.4 Hz/cm
2.03
0.94
2.08
2.04
2.06
3.00

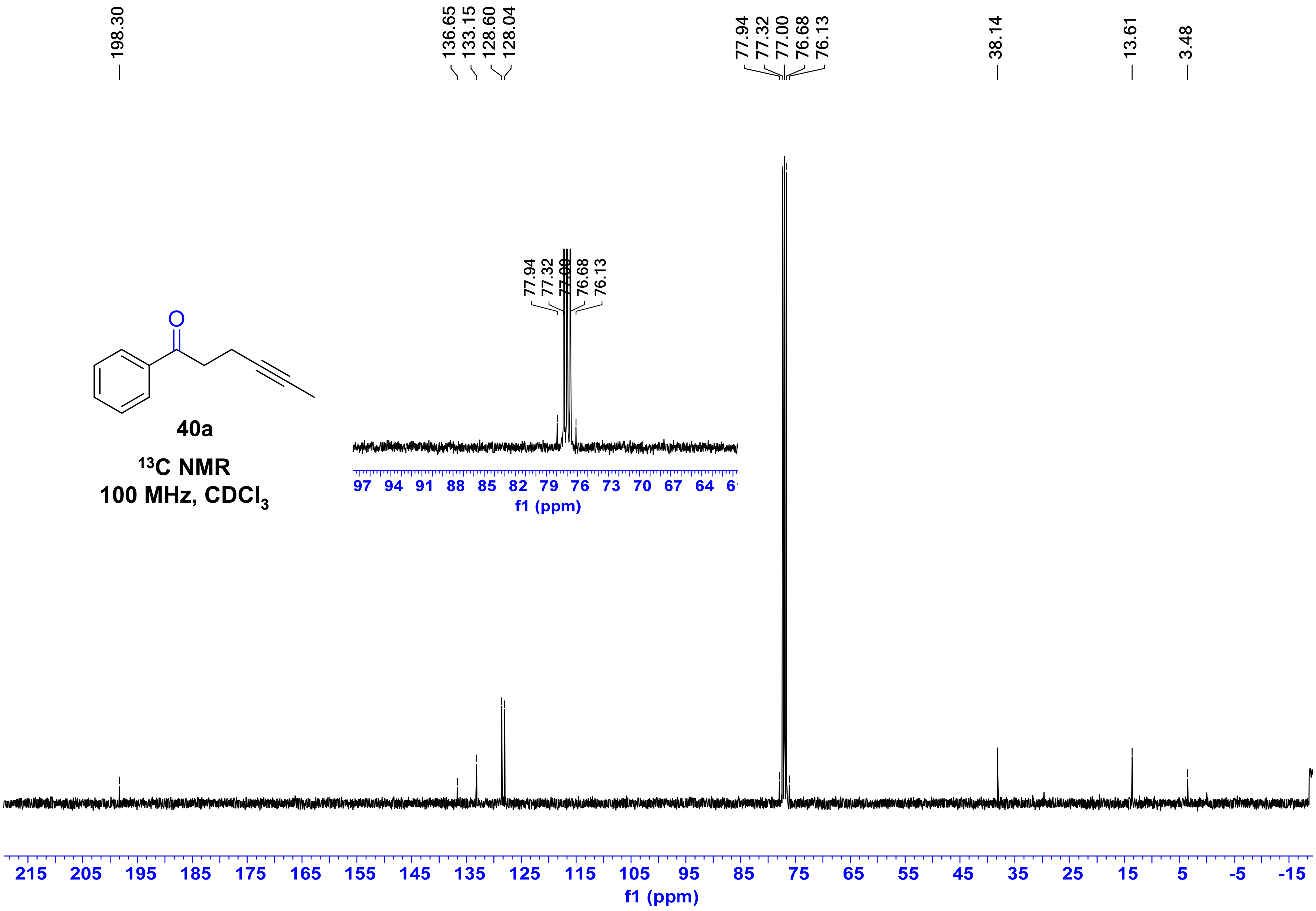

O
40a
^{13}C NMR
100 MHz, $CDCl_3$
198.30
136.65
133.15
128.60
128.04
77.94
77.32
77.00
76.68
76.13
38.14
13.61
3.48
97 94 91 88 85 82 79 76 73 70 67 64
f1 (ppm)
215 205 195 185 175 165 155 145 135 125 115 105 95 85 75 65 55 45 35 25 15 5 -5 -15
f1 (ppm)

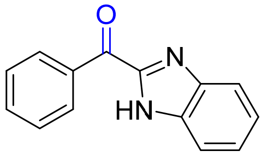

41a

^{1}H NMR,
400 MHz, $CDCl_3$

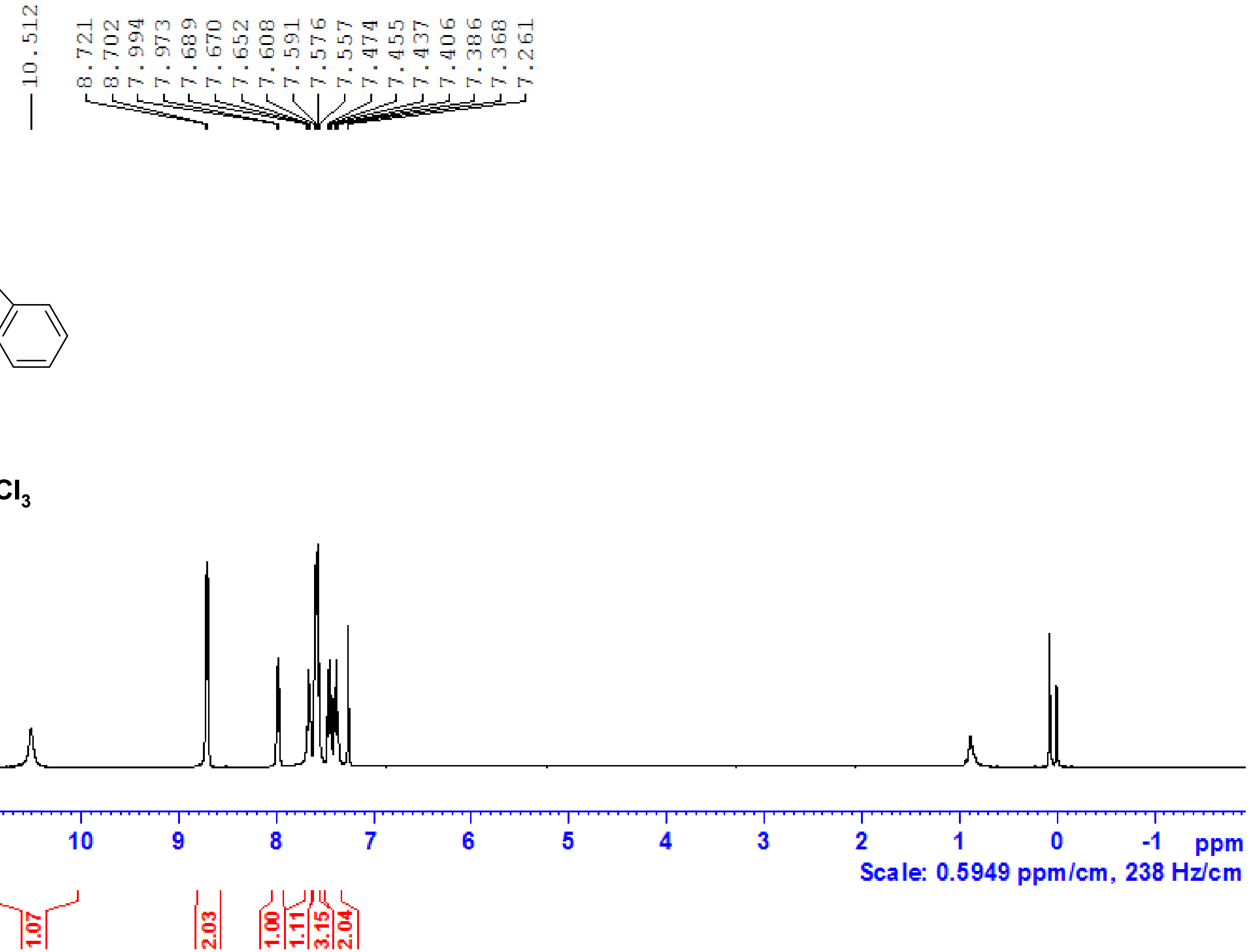

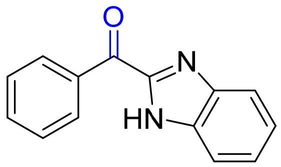

41a

^{13}C NMR
100 MHz, $CDCl_3$

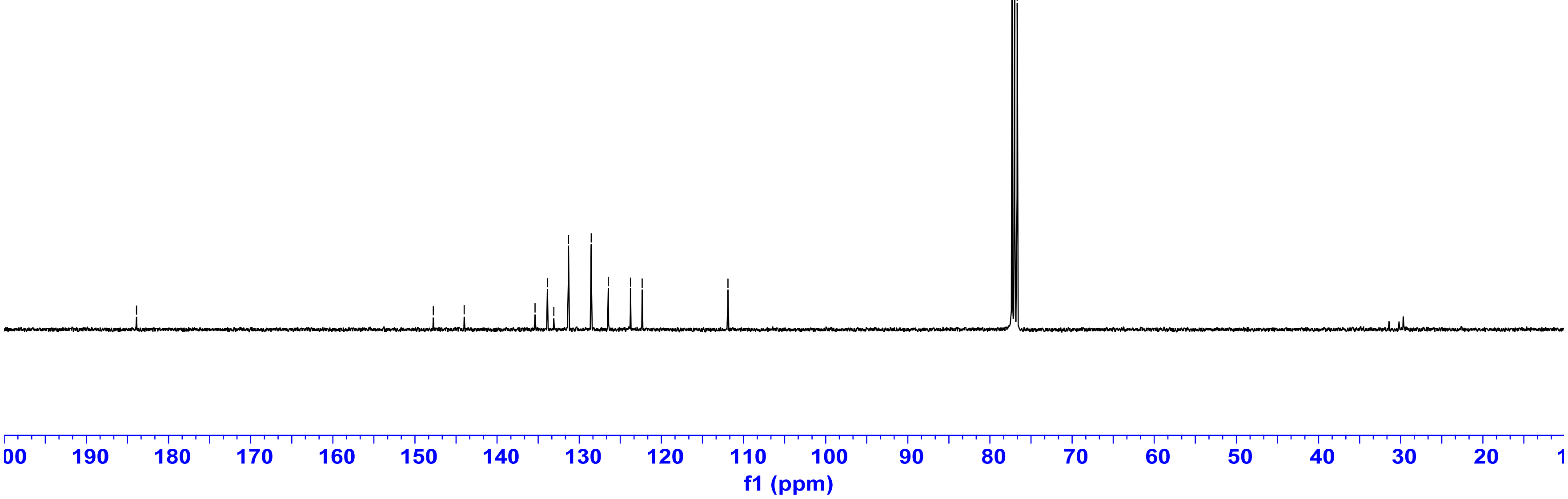

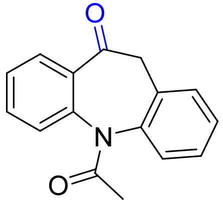

42a

^{1}H NMR,
400 MHz, $CDCl_3$

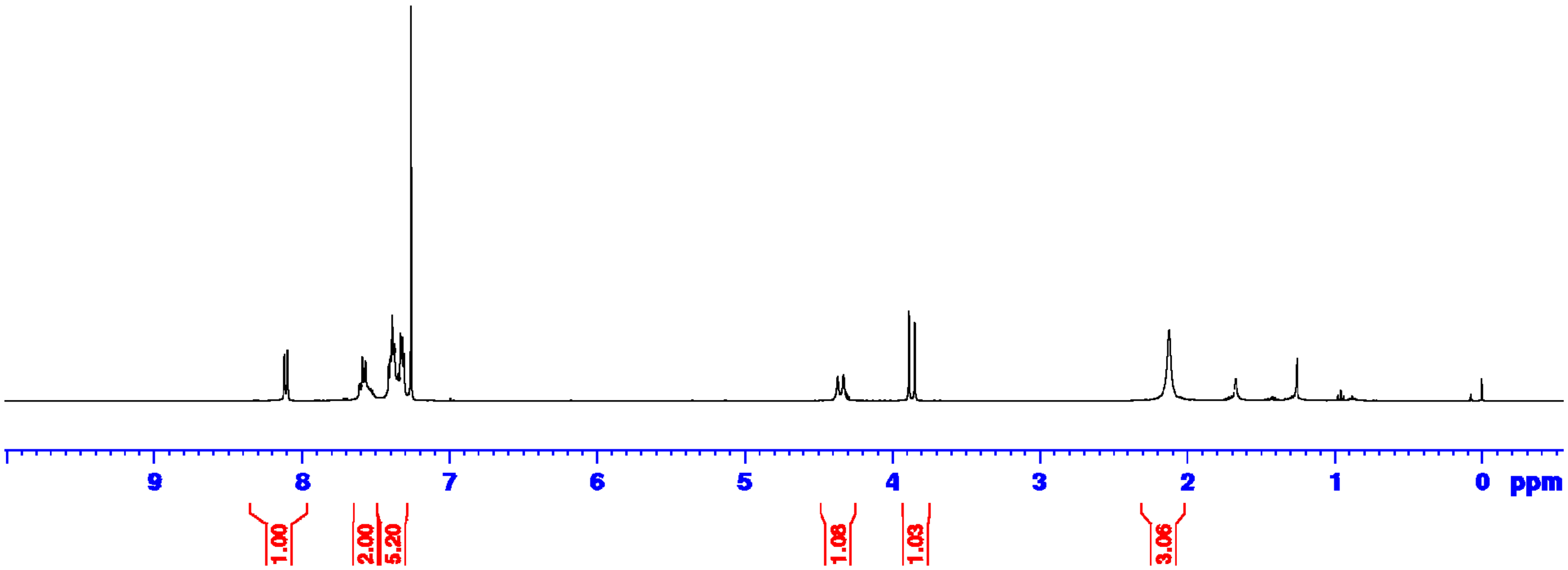

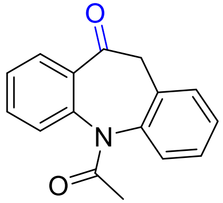

42a

^{13}C NMR
100 MHz, $CDCl_3$

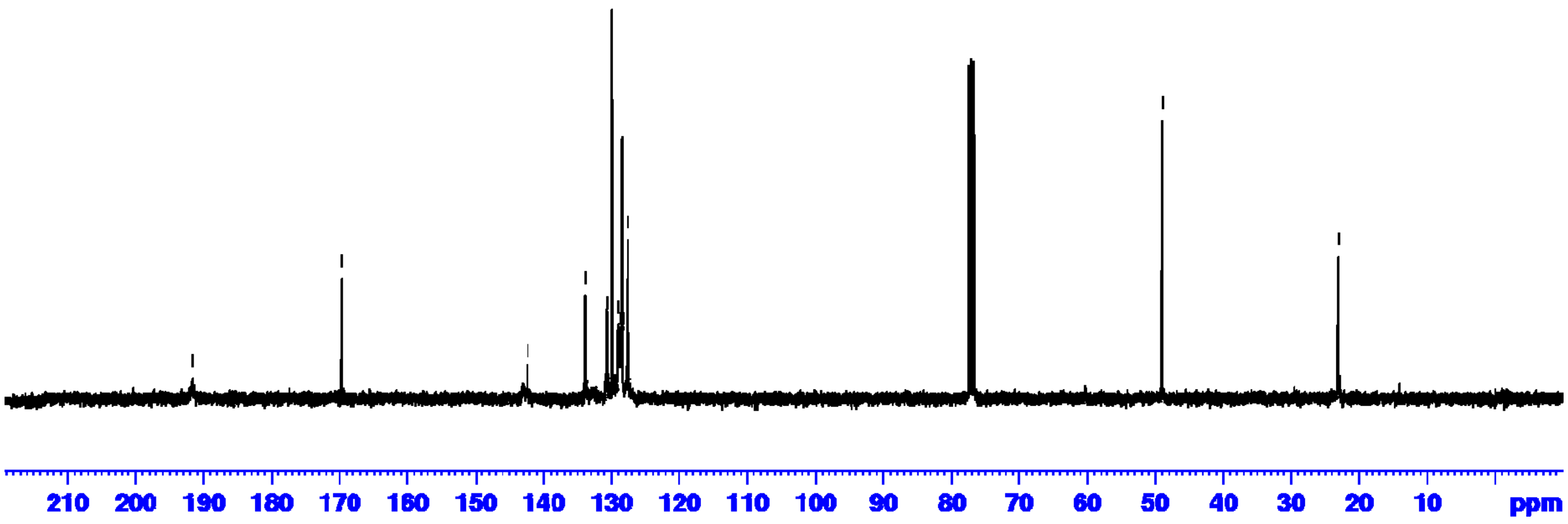

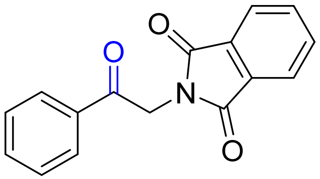

43a

^{1}H NMR, 400 MHz, $CDCl_3$

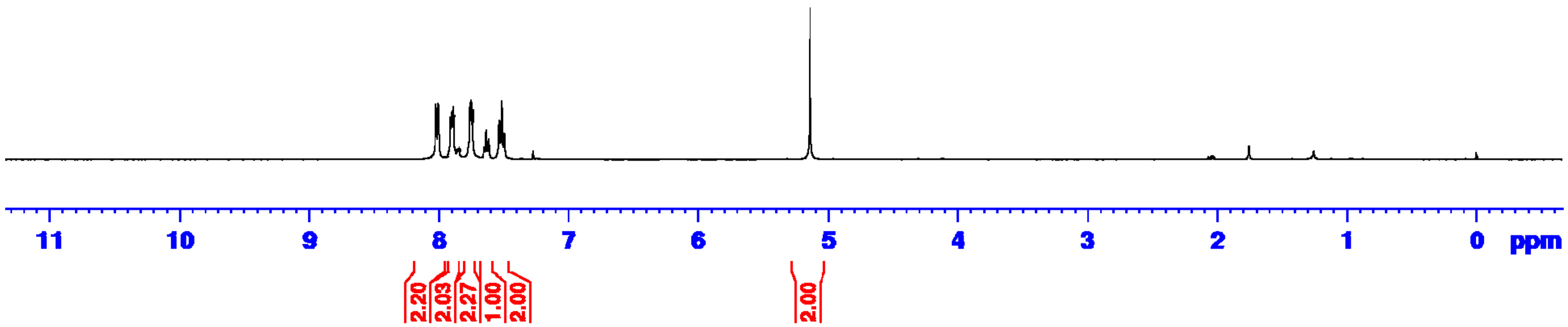

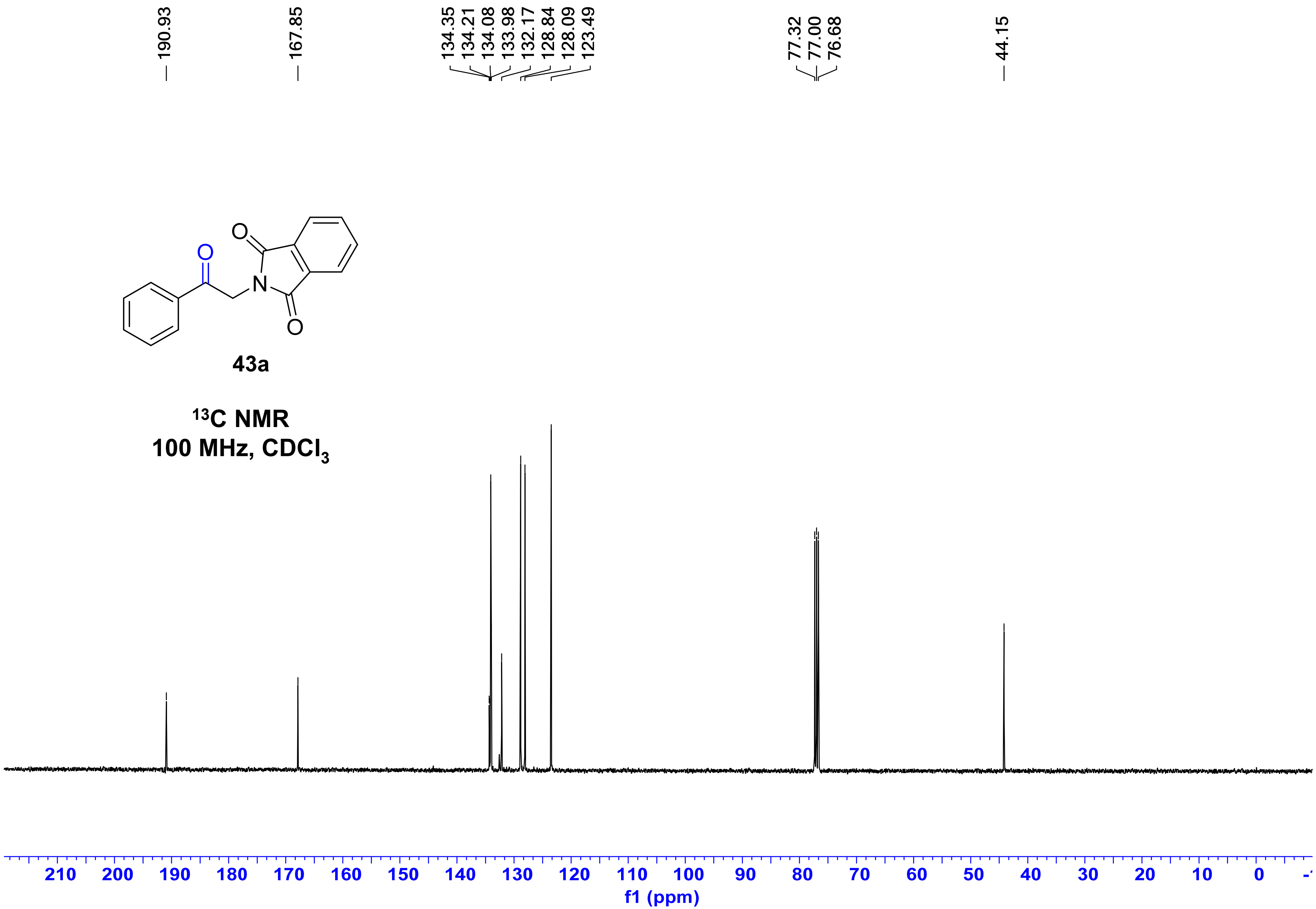
190.93
167.85
134.35
134.21
134.08
133.98
132.17
128.84
128.09
123.49
77.32
77.00
76.68
44.15
O
O
N
O
43a
13C NMR
100 MHz, CDCl3
210
200
190
180
170
160
150
140
130
120
110
100
90
80
70
60
50
40
30
20
10
0
f1 (ppm)

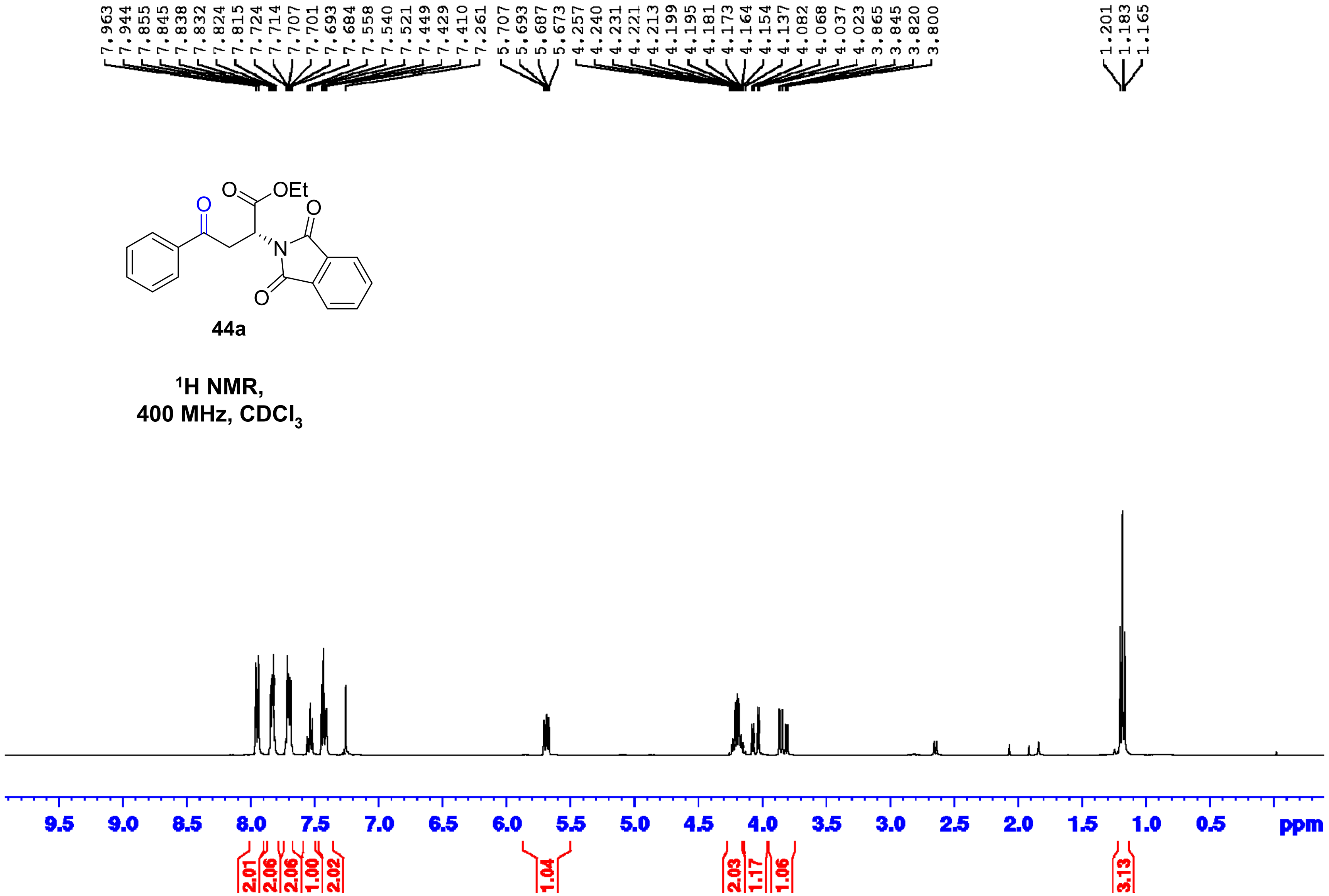

7.963
7.944
7.855
7.845
7.838
7.832
7.824
7.815
7.724
7.714
7.707
7.701
7.693
7.684
7.558
7.540
7.521
7.449
7.429
7.410
7.261
5.707
5.693
5.687
5.673
4.257
4.240
4.231
4.221
4.213
4.199
4.195
4.181
4.173
4.164
4.154
4.137
4.082
4.068
4.037
4.023
3.865
3.845
3.820
3.800
1.201
1.183
1.165
O
OEt
N
44a
^{1}H NMR,
400 MHz, $CDCl_3$
9.5
9.0
8.5
8.0
7.5
7.0
6.5
6.0
5.5
5.0
4.5
4.0
3.5
3.0
2.5
2.0
1.5
1.0
0.5
ppm
2.01
2.06
2.06
1.00
2.02
1.04
2.03
1.17
1.06
3.13

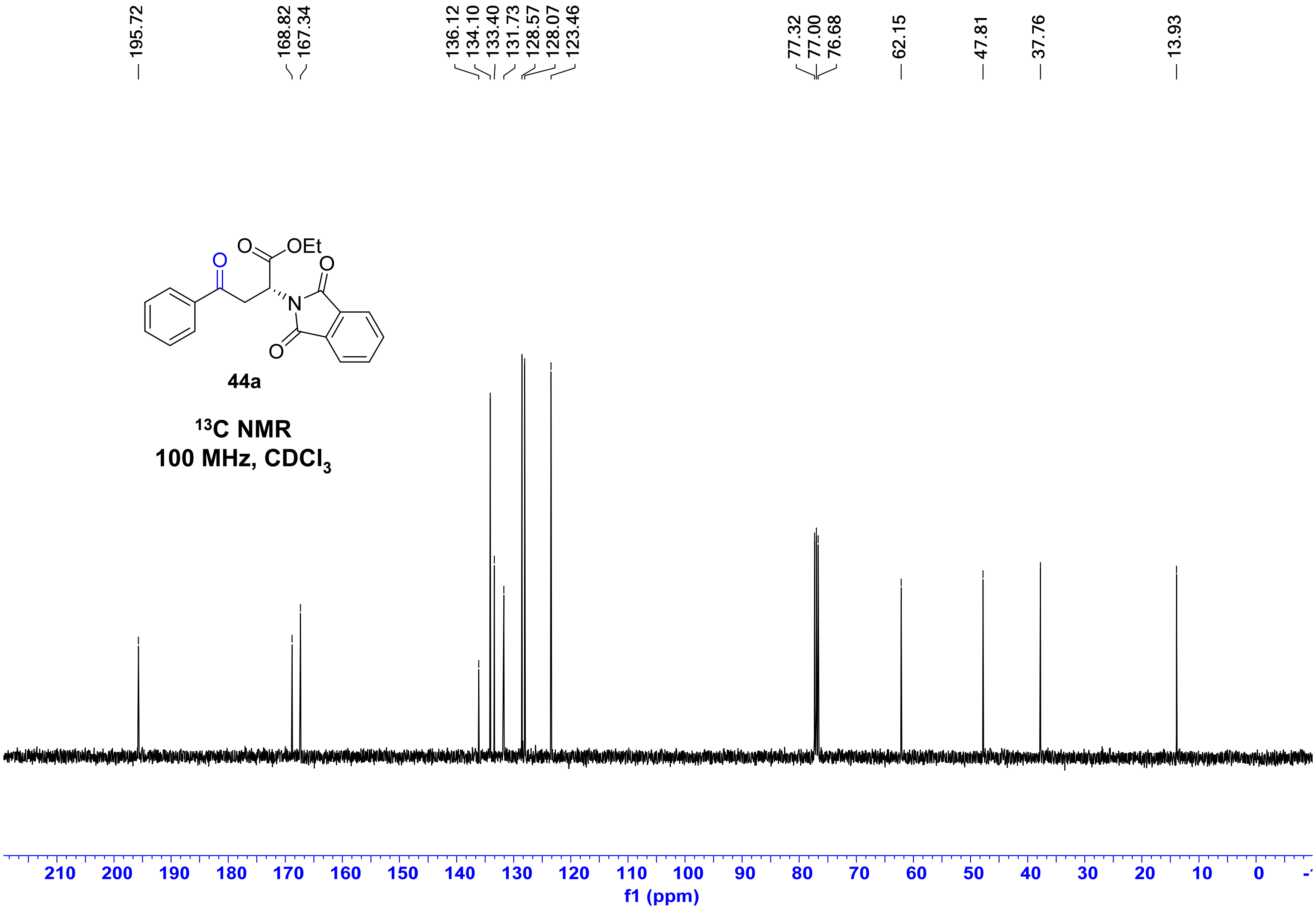

195.72
168.82
167.34
136.12
134.10
133.40
131.73
128.57
128.07
123.46
77.32
77.00
76.68
62.15
47.81
37.76
13.93
O
OEt
O
O
N
O
44a
13C NMR
100 MHz, CDCl3
210 200 190 180 170 160 150 140 130 120 110 100 90 80 70 60 50 40 30 20 10 0
f1 (ppm)

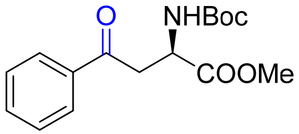

45a

^{1}H NMR,
400 MHz, $CDCl_3$

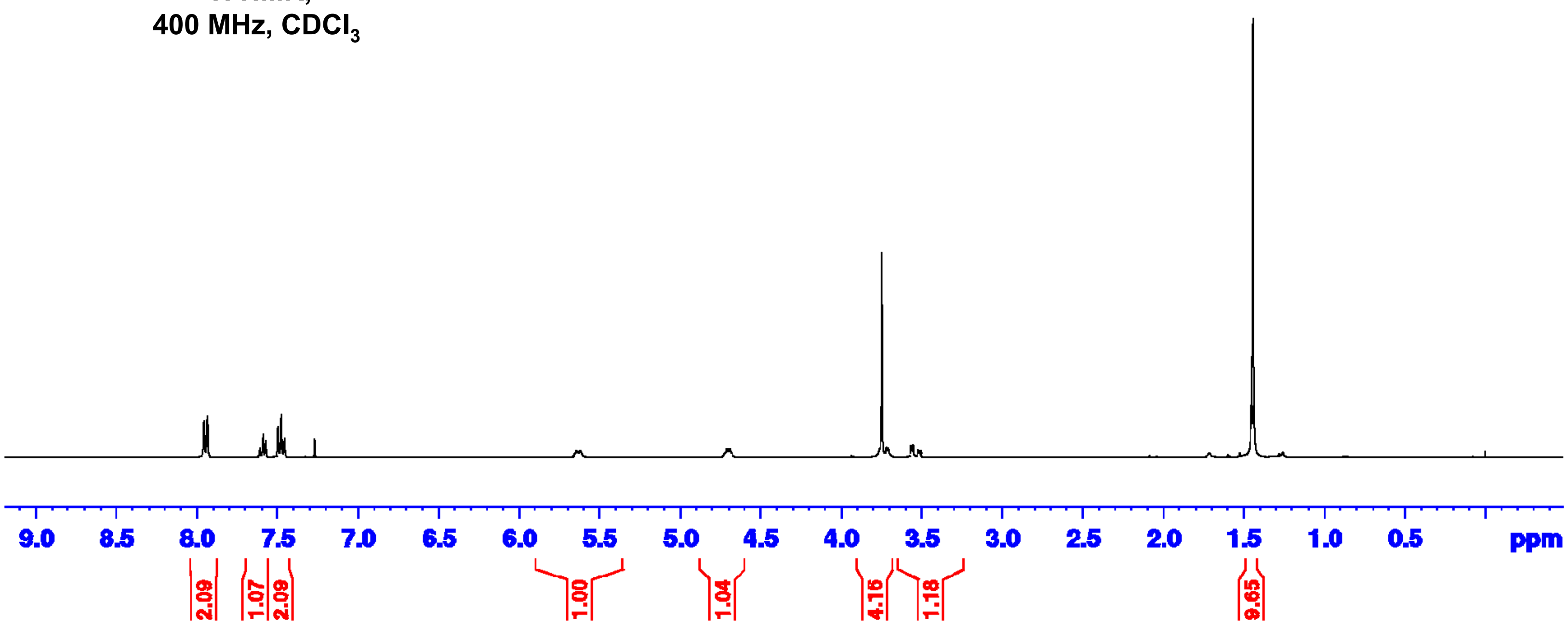

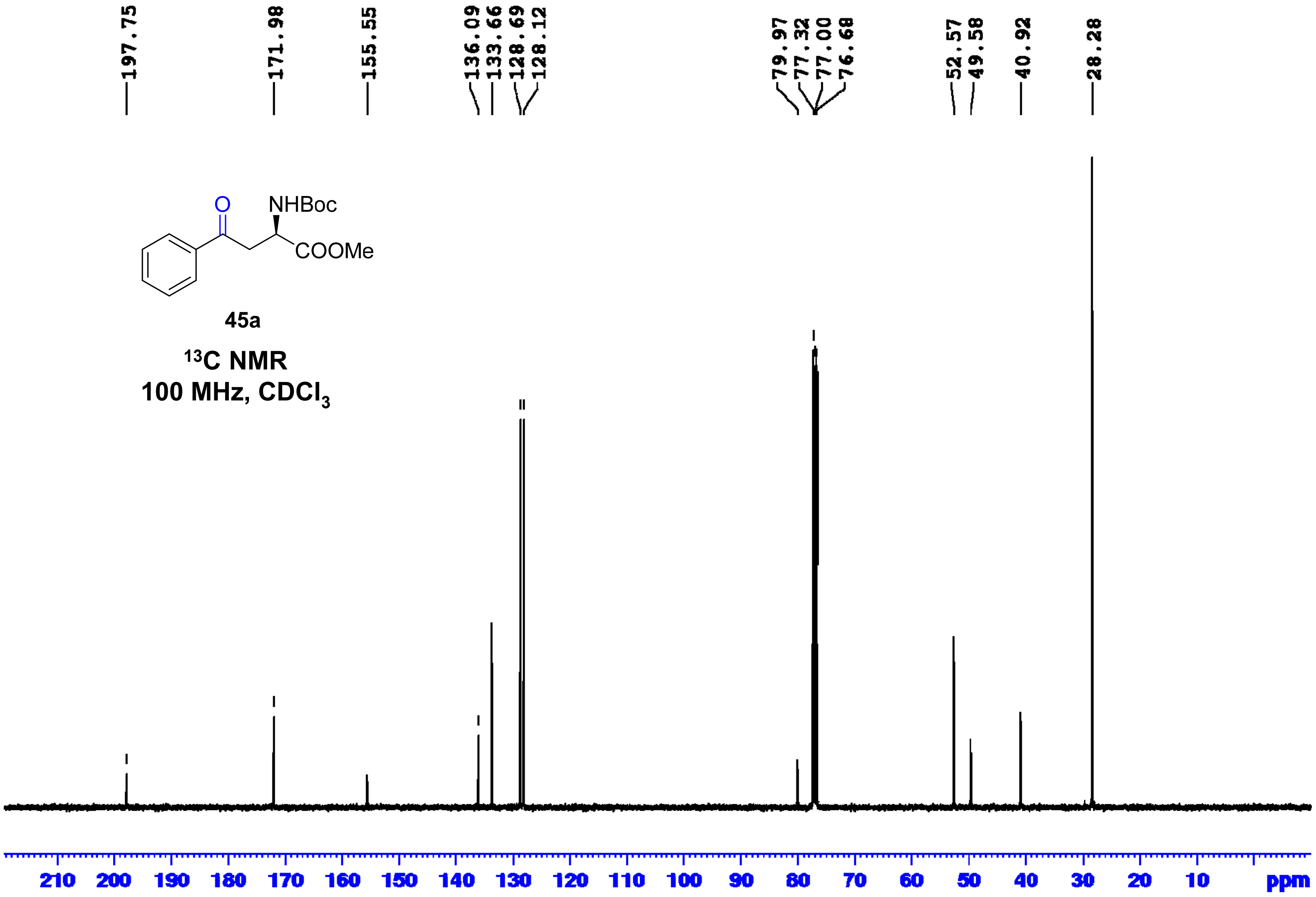
197.75
171.98
155.55
136.09
133.66
128.69
128.12
79.97
77.32
77.00
76.68
52.57
49.58
40.92
28.28
O
NHBoc
COOMe
45a
13C NMR
100 MHz, CDCl3
210 200 190 180 170 160 150 140 130 120 110 100 90 80 70 60 50 40 30 20 10 ppm

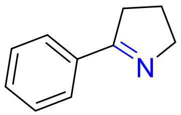

46a

^{1}H NMR,
400 MHz, $CDCl_3$

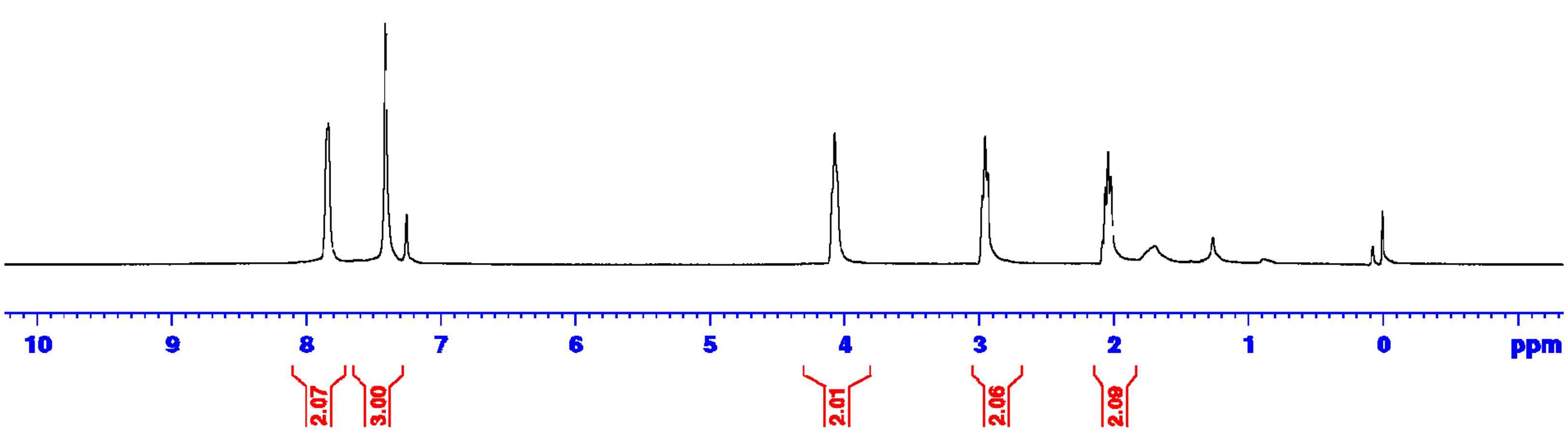

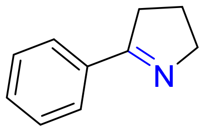

46a

^{13}C NMR
100 MHz, $CDCl_3$

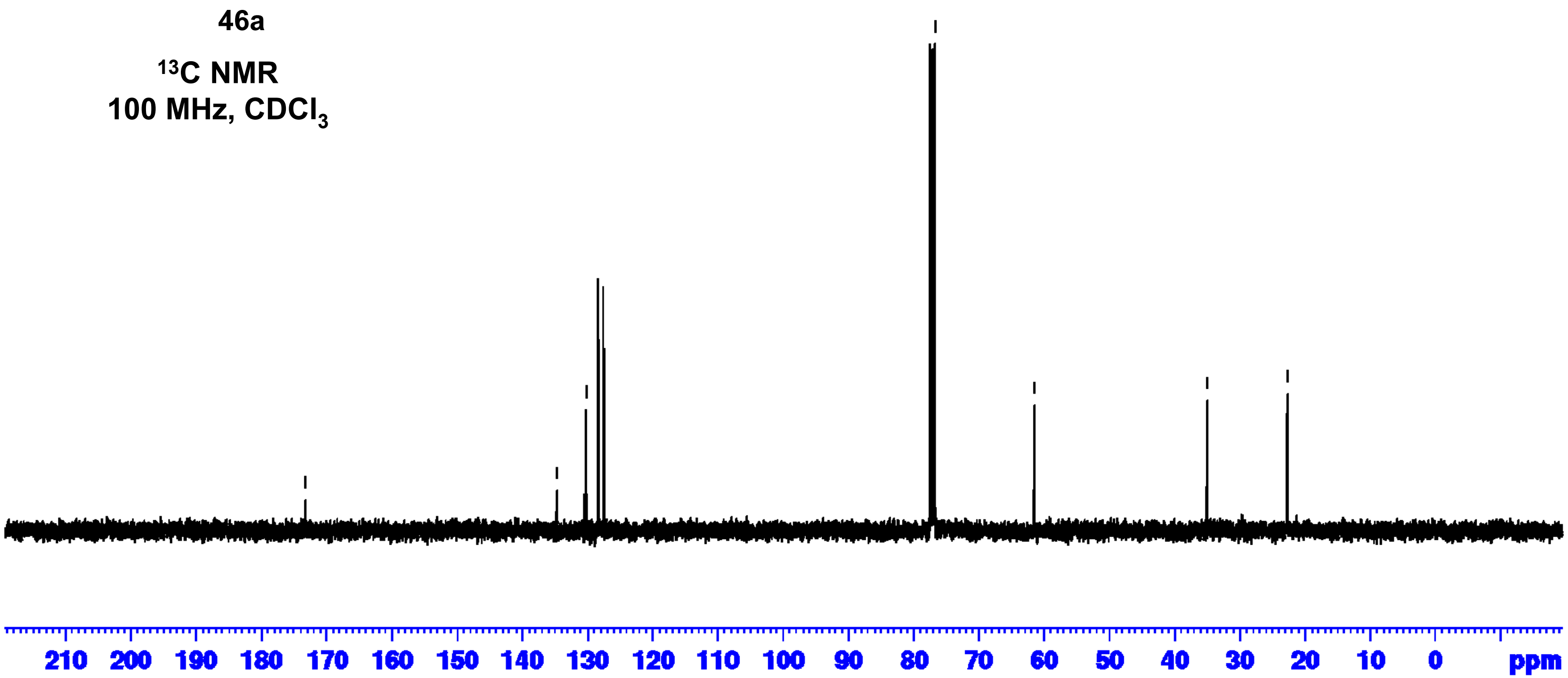

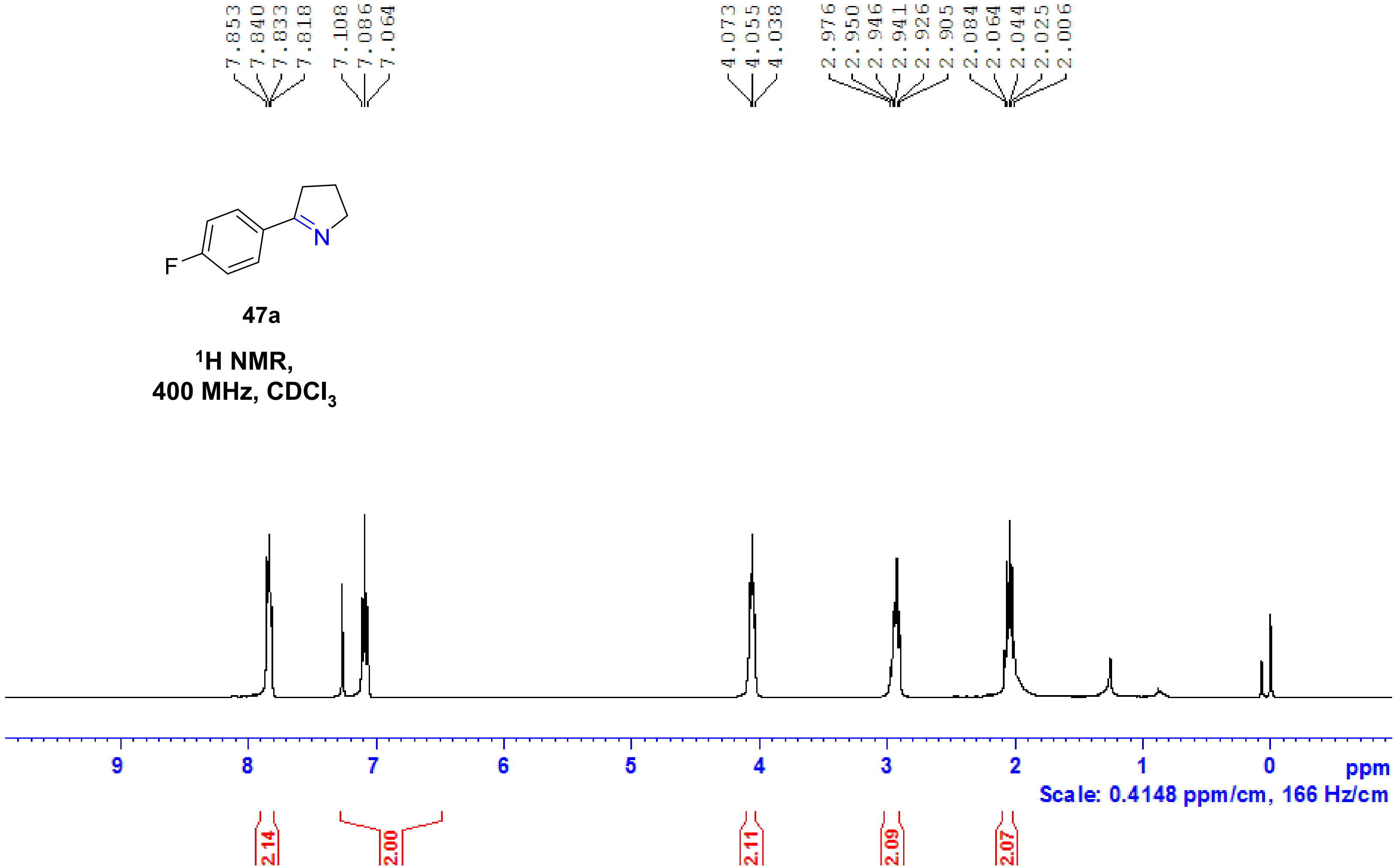

47a

^{1}H NMR, 400 MHz, $CDCl_3$

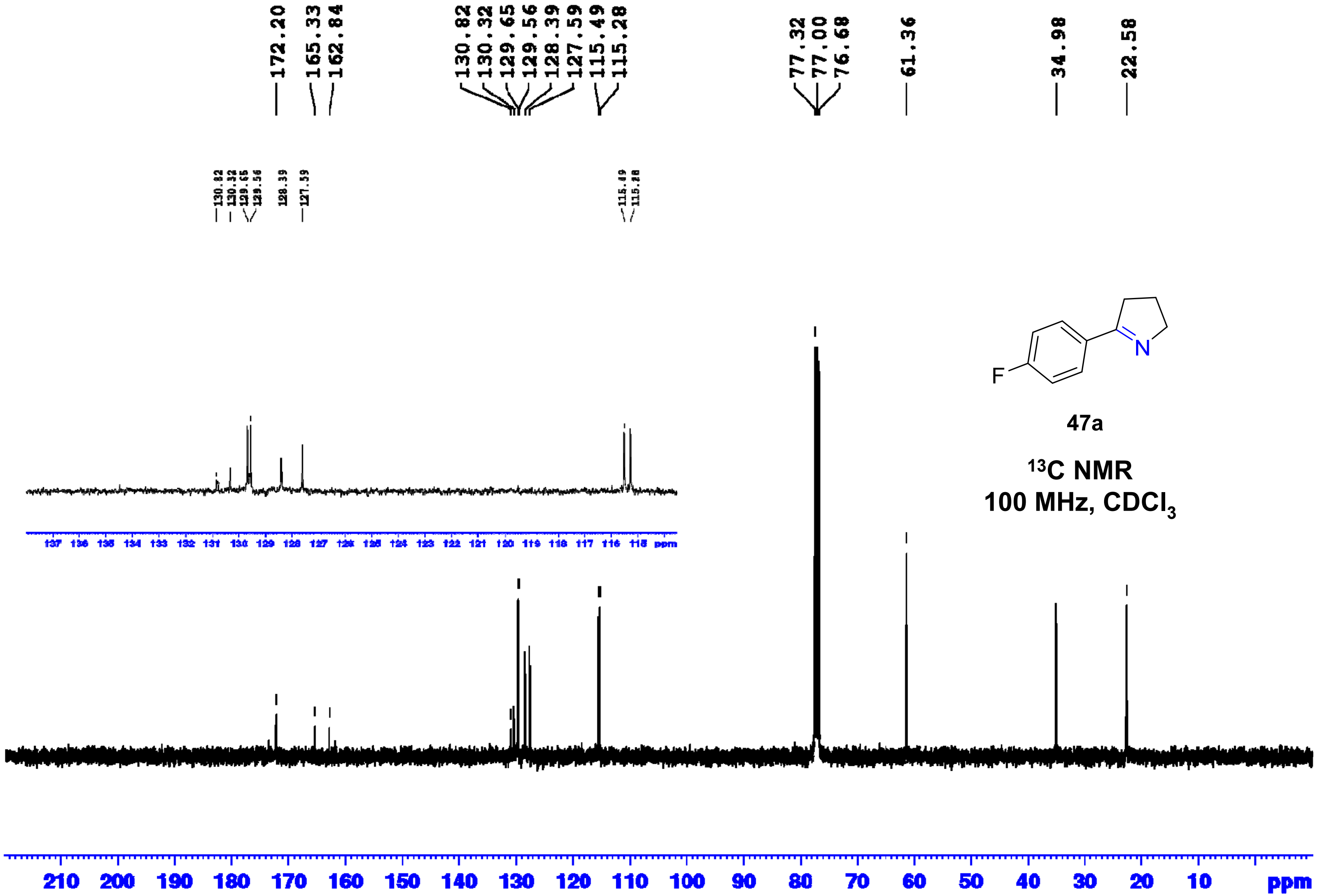
172.20
165.33
162.84
130.82
130.32
129.65
129.56
128.39
127.59
115.49
115.28
77.32
77.00
76.68
61.36
34.98
22.58
F
N
47a
^{13}C NMR
100 MHz, $CDCl_3$
210 200 190 180 170 160 150 140 130 120 110 100 90 80 70 60 50 40 30 20 10 ppm

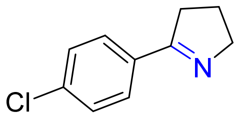

48a

**^{1}H NMR,
400 MHz, $CDCl_3$**

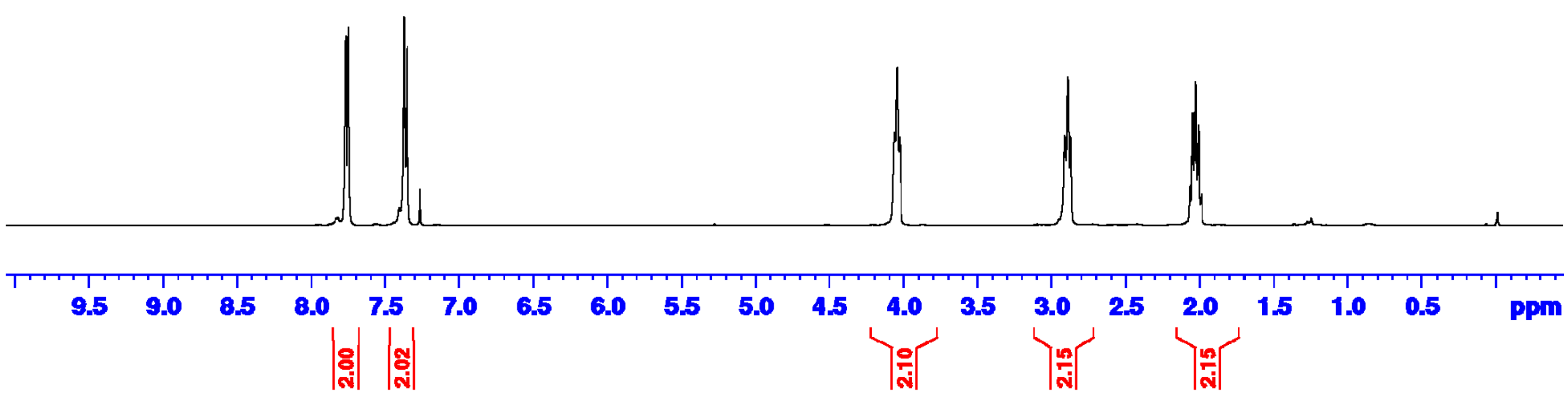

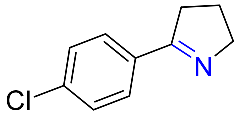

48a

^{13}C NMR
100 MHz, $CDCl_3$

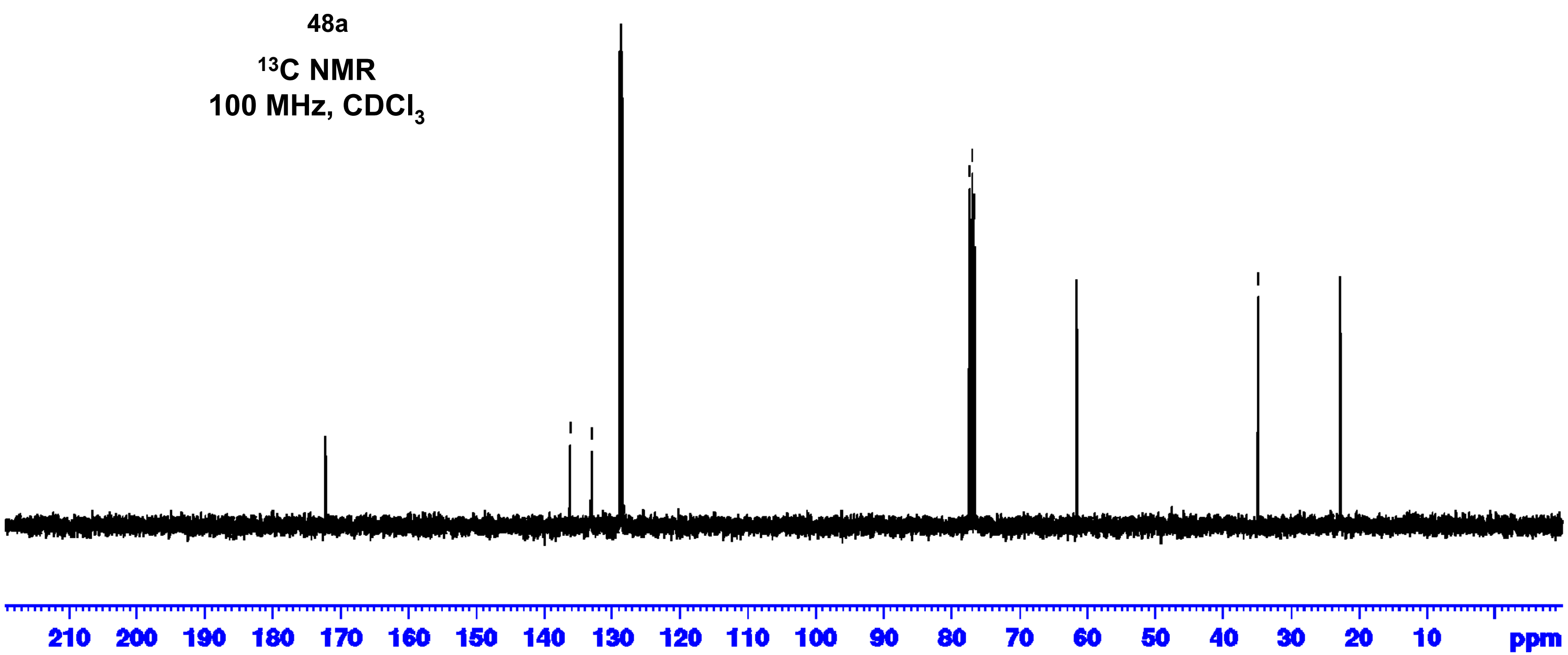

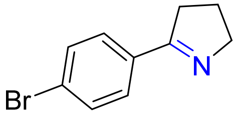

49a

^{1}H NMR,
400 MHz, $CDCl_3$

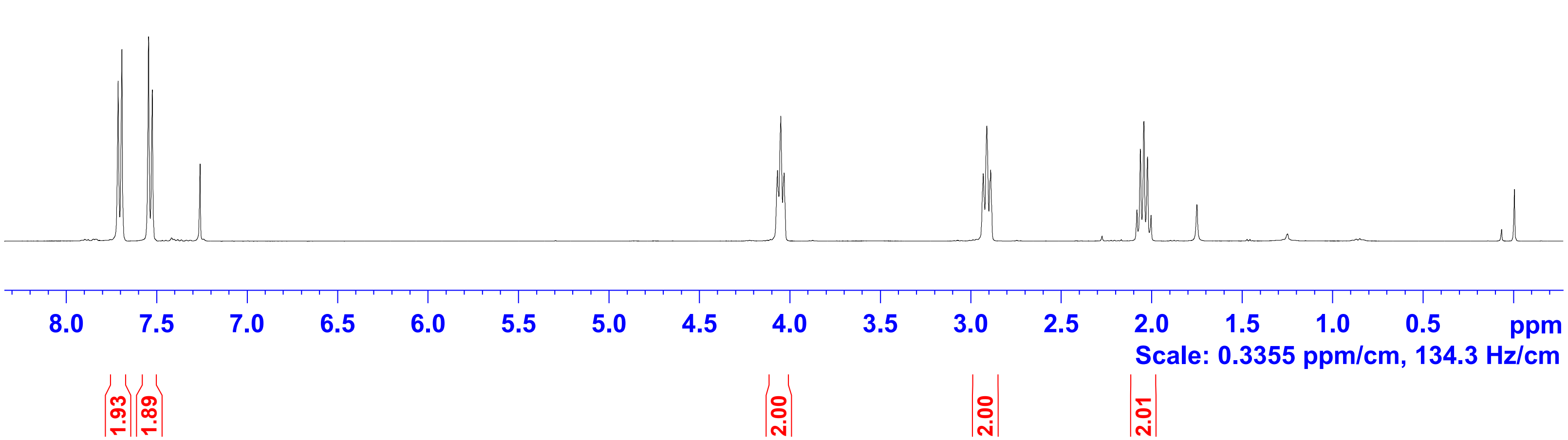

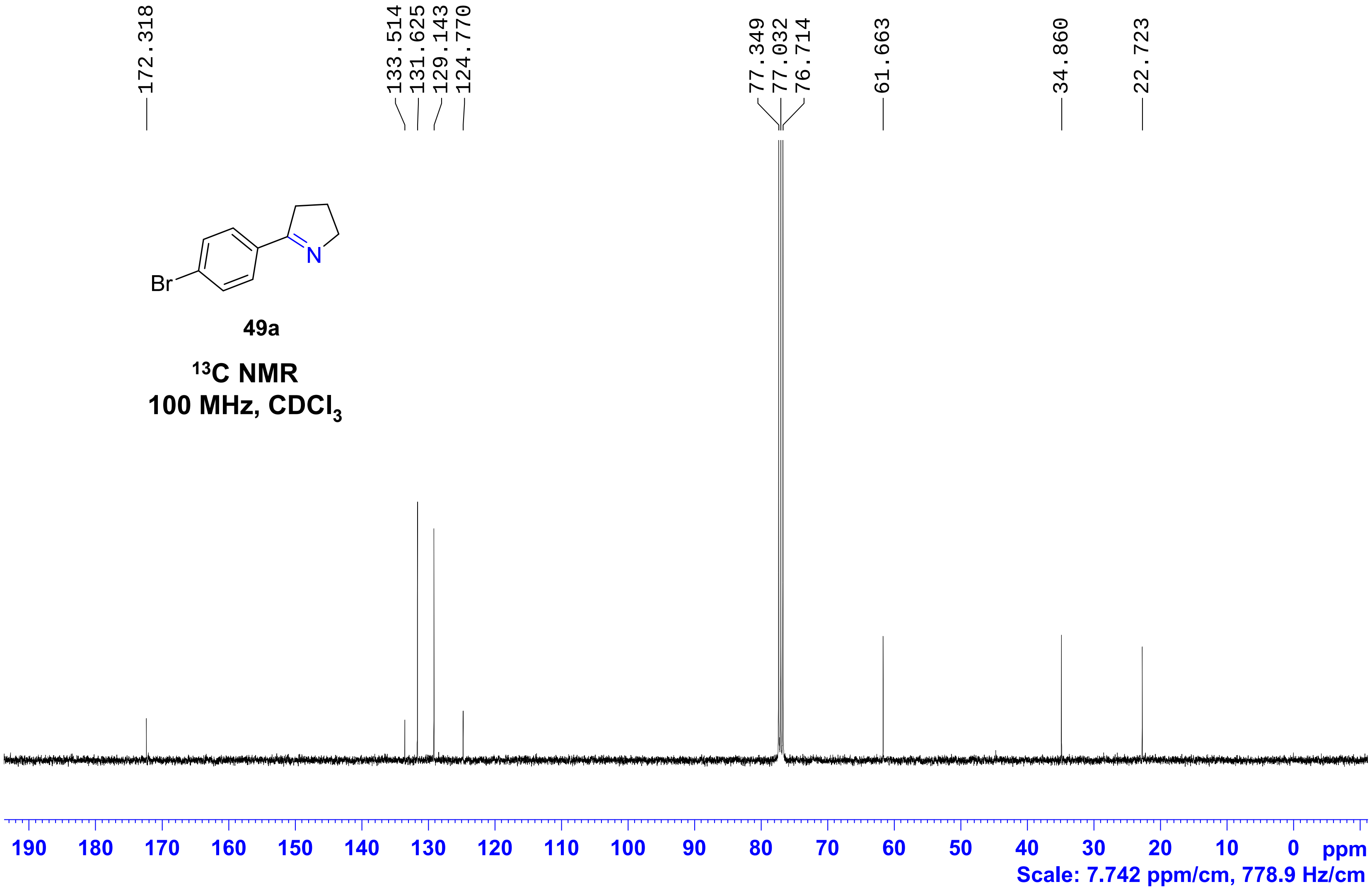

172.318
133.514
131.625
129.143
124.770
77.349
77.032
76.714
61.663
34.860
22.723
Br
N
49a
^{13}C NMR
100 MHz, CDCl$_3$
190 180 170 160 150 140 130 120 110 100 90 80 70 60 50 40 30 20 10 0 ppm
Scale: 7.742 ppm/cm, 778.9 Hz/cm

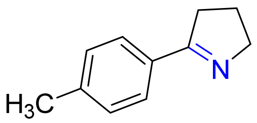

50a

^{1}H NMR,
400 MHz, CDCl$_3$

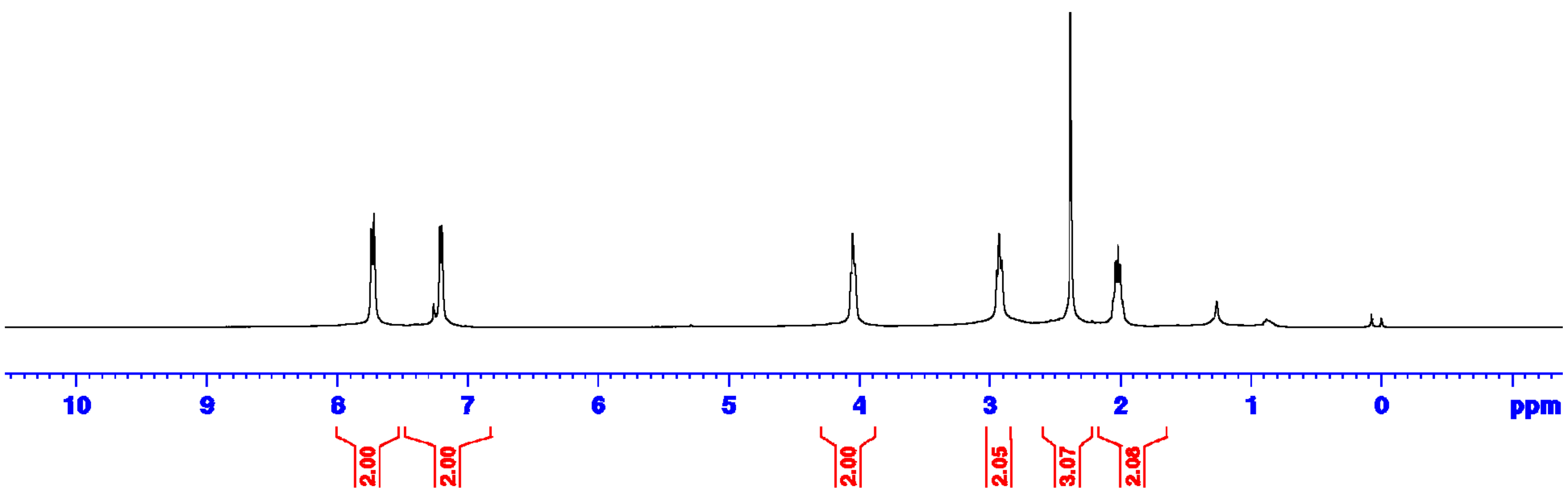

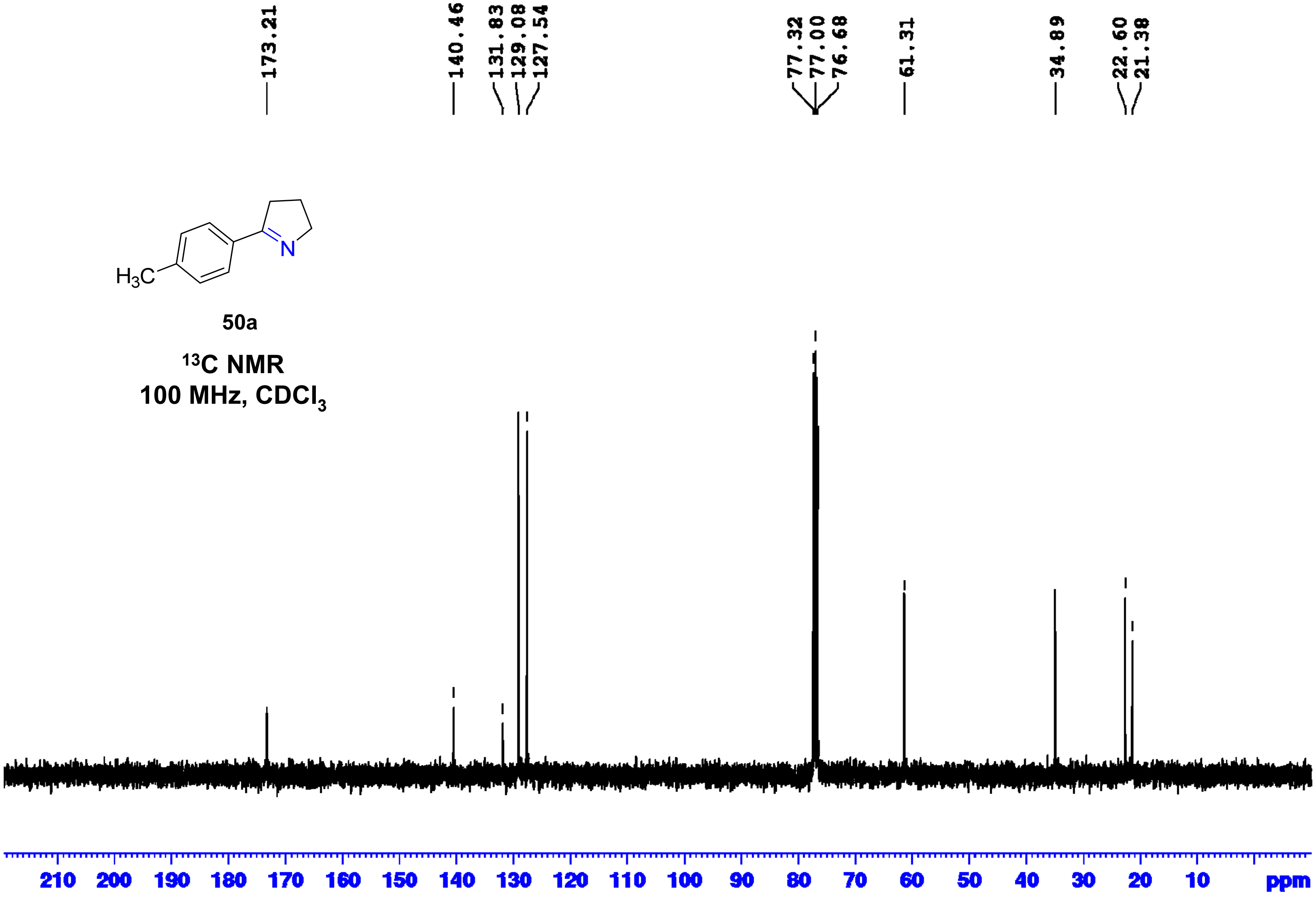

50a

^{13}C NMR
100 MHz, $CDCl_3$

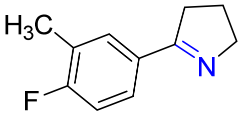

51a

^{1}H NMR,
400 MHz, CDCl$_3$

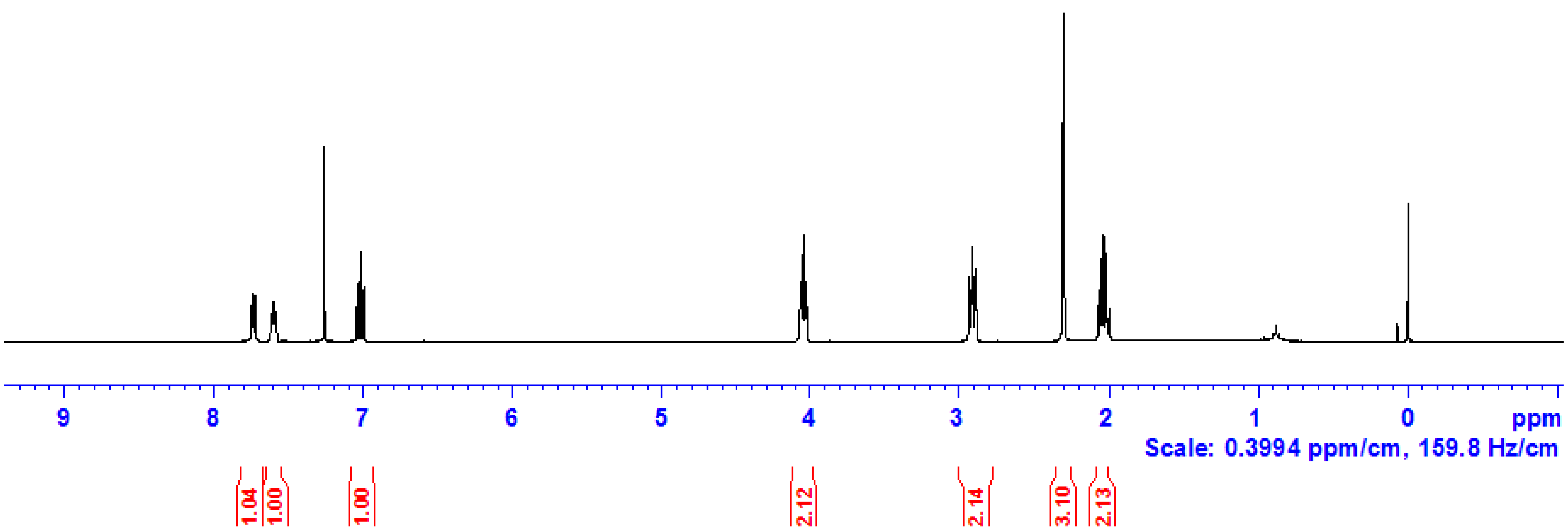

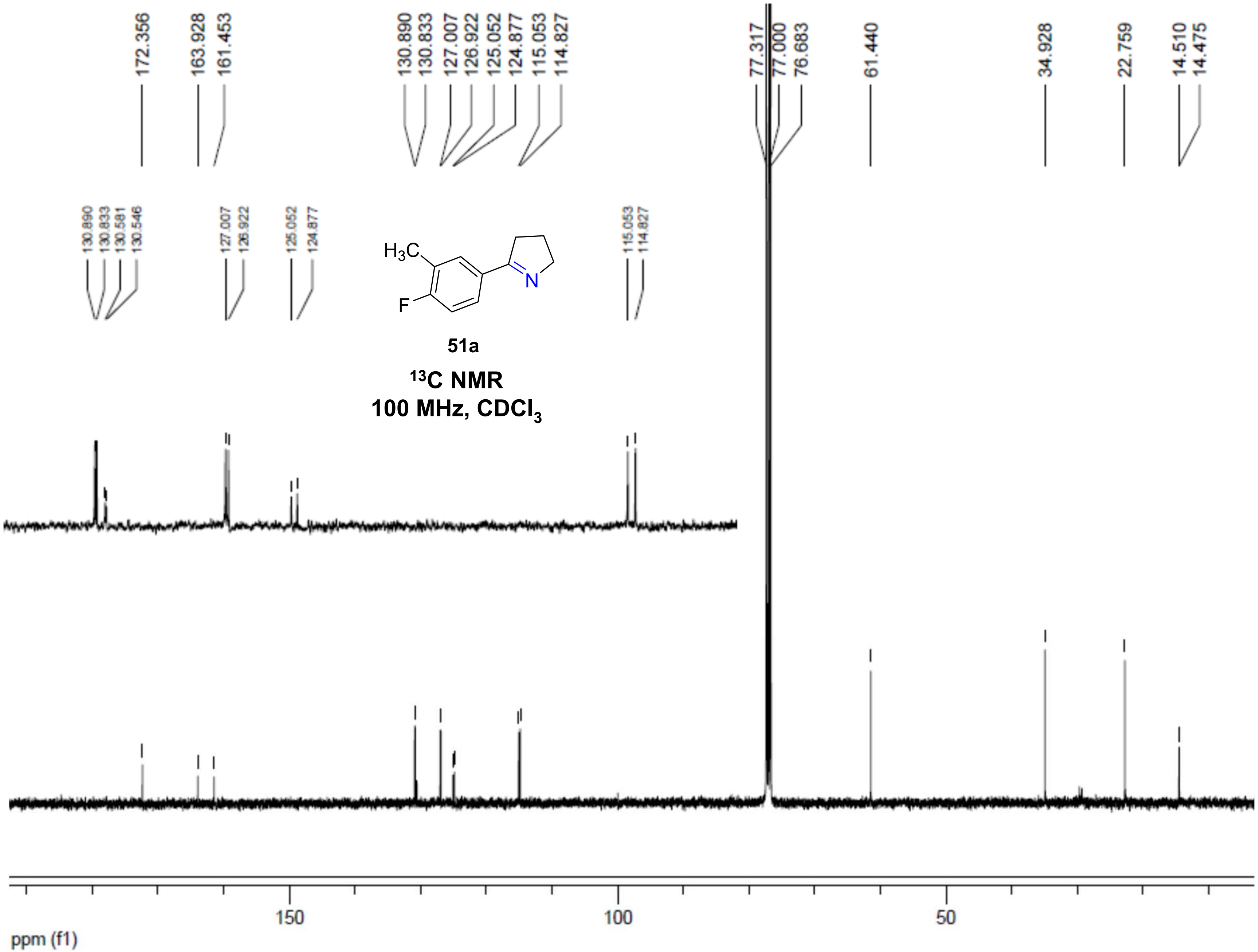
172.356
163.928
161.453
130.890
130.833
127.007
126.922
125.052
124.877
115.053
114.827
77.317
77.000
76.683
61.440
34.928
22.759
14.510
14.475
130.890
130.833
130.581
130.546
127.007
126.922
125.052
124.877
115.053
114.827
H3C
F
N
51a
13C NMR
100 MHz, CDCl3
150
100
50
ppm (f1)

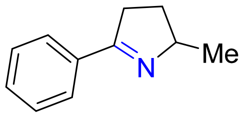

52a

^{1}H NMR,
400 MHz, $CDCl_3$

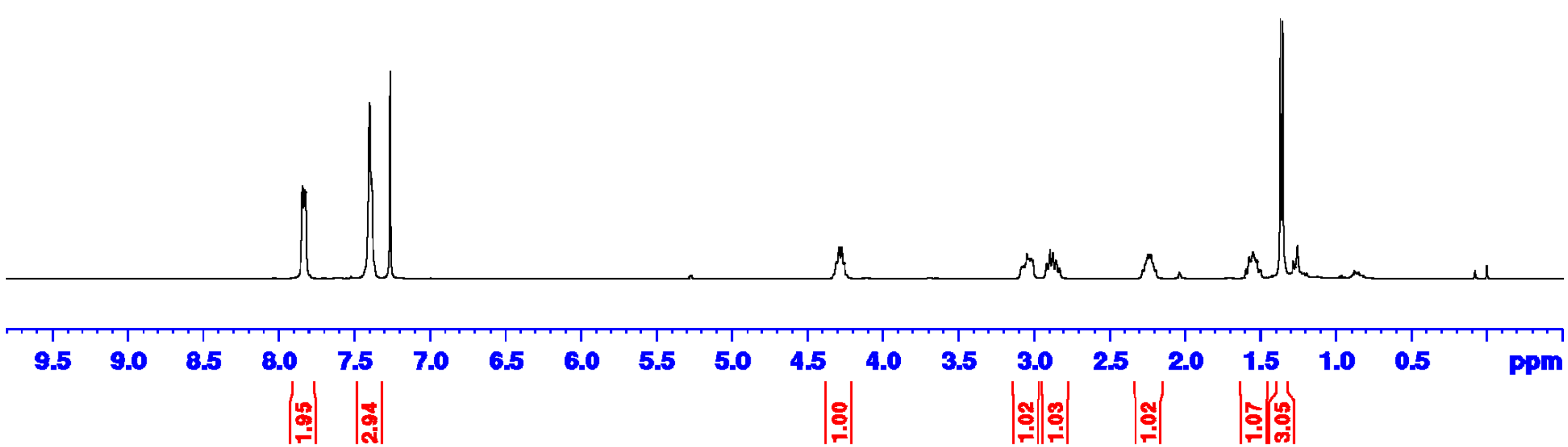

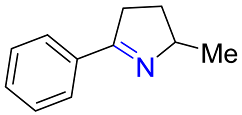

52a

^{13}C NMR
100 MHz, $CDCl_3$

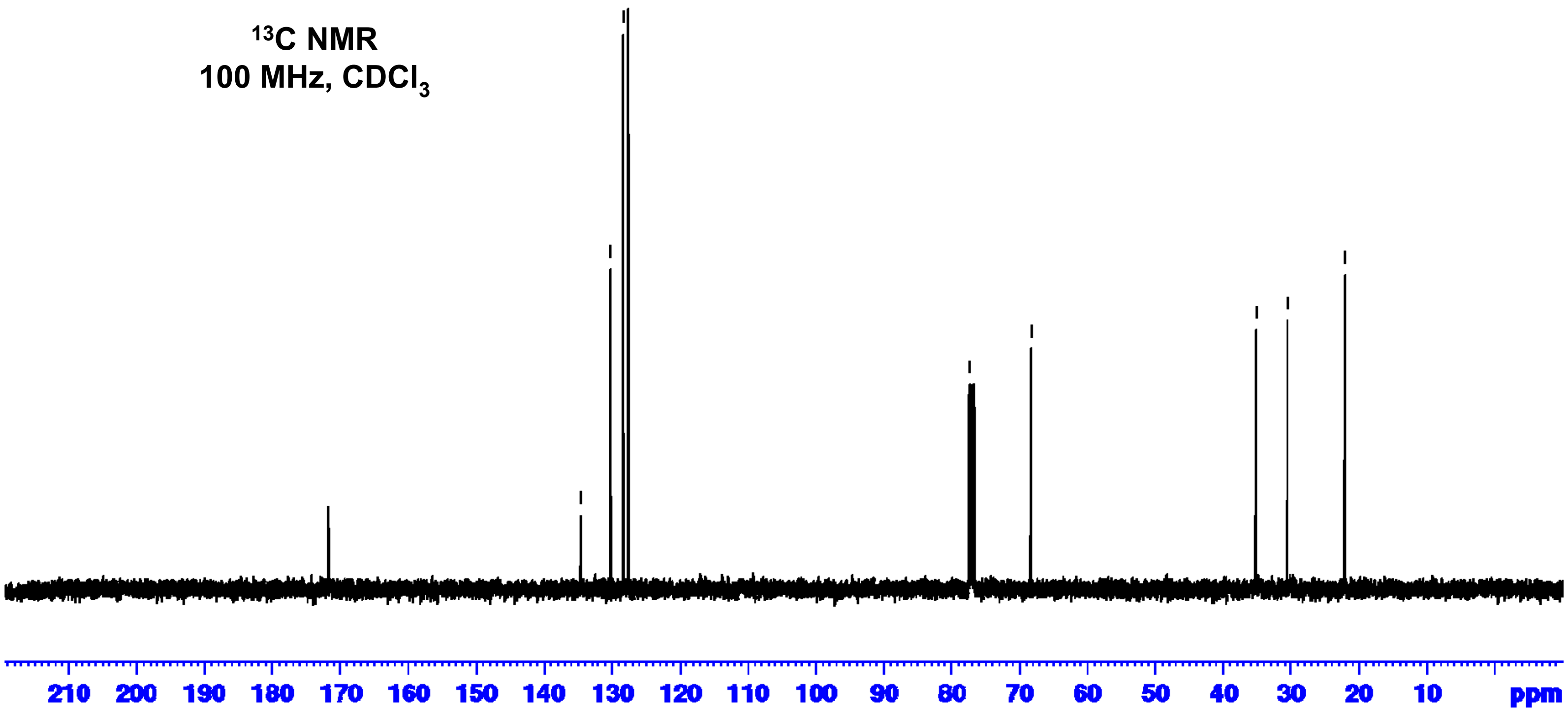

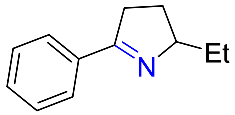

53a

^{1}H NMR,
400 MHz, $CDCl_3$

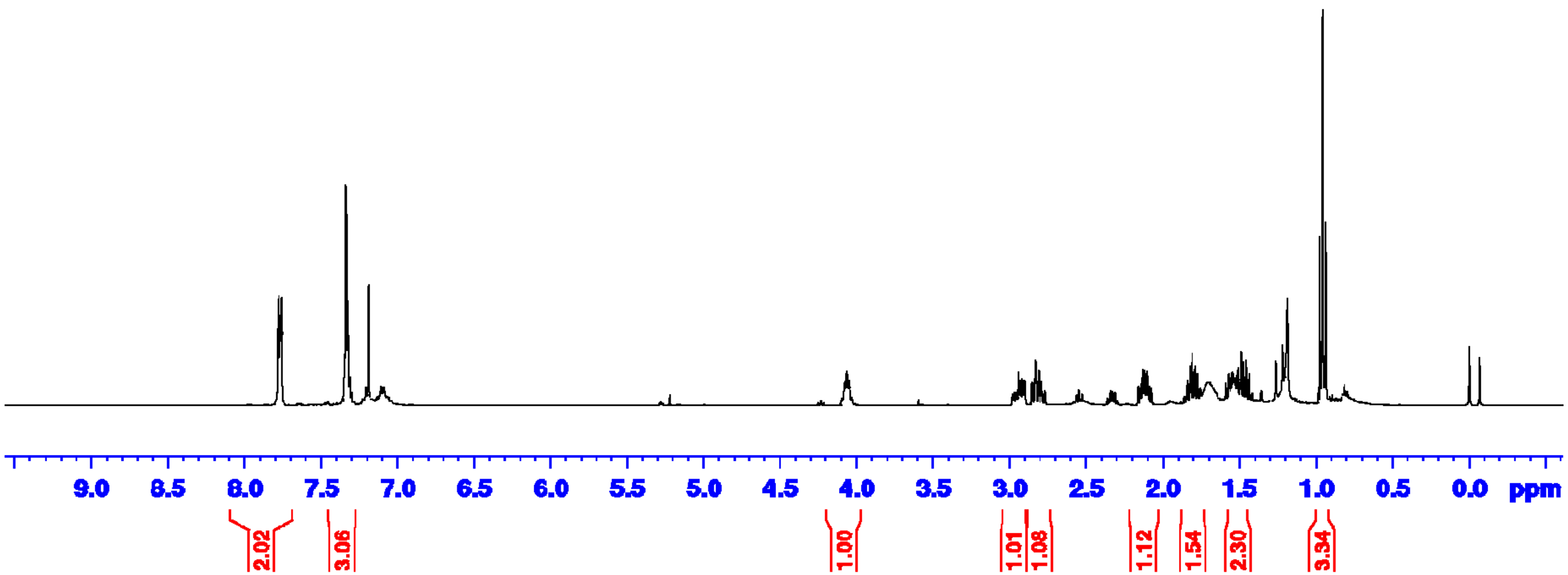

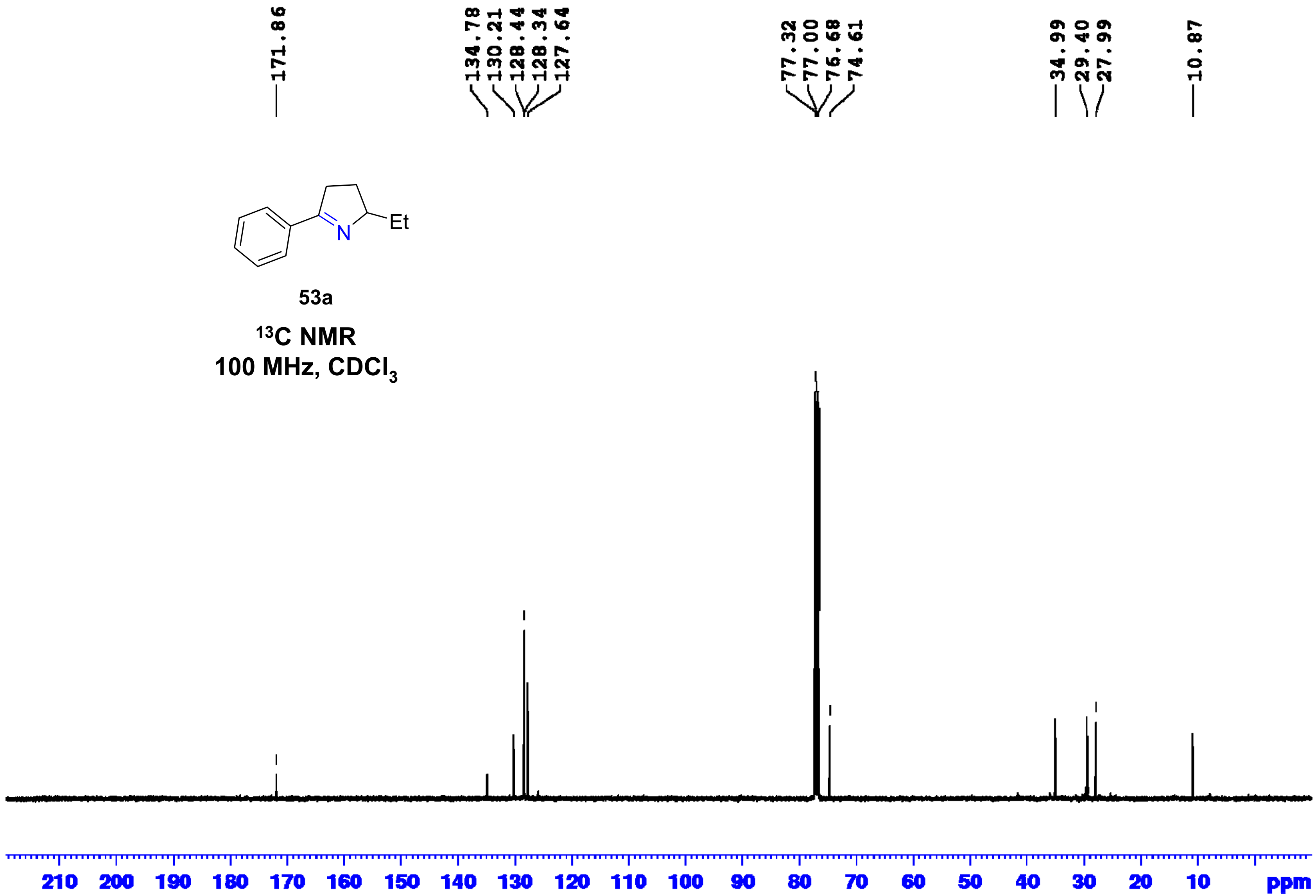
171.86
134.78
130.21
128.44
128.34
127.64
77.32
77.00
76.68
74.61
34.99
29.40
27.99
10.87
Et
N
53a
13C NMR
100 MHz, CDCl3
210
200
190
180
170
160
150
140
130
120
110
100
90
80
70
60
50
40
30
20
10
ppm

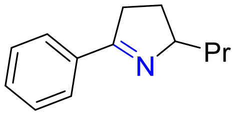

54a

^{1}H NMR,
400 MHz, $CDCl_3$

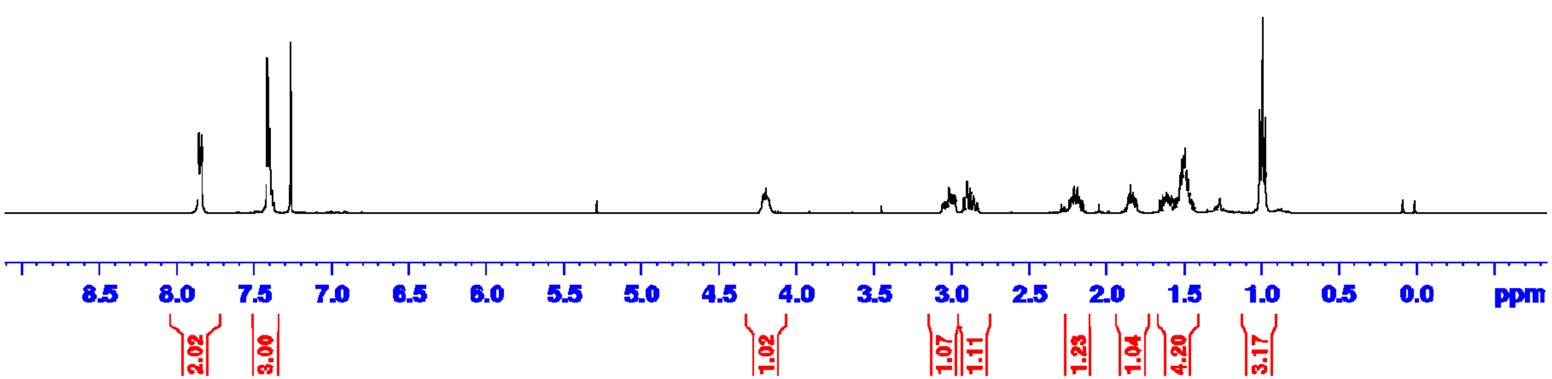

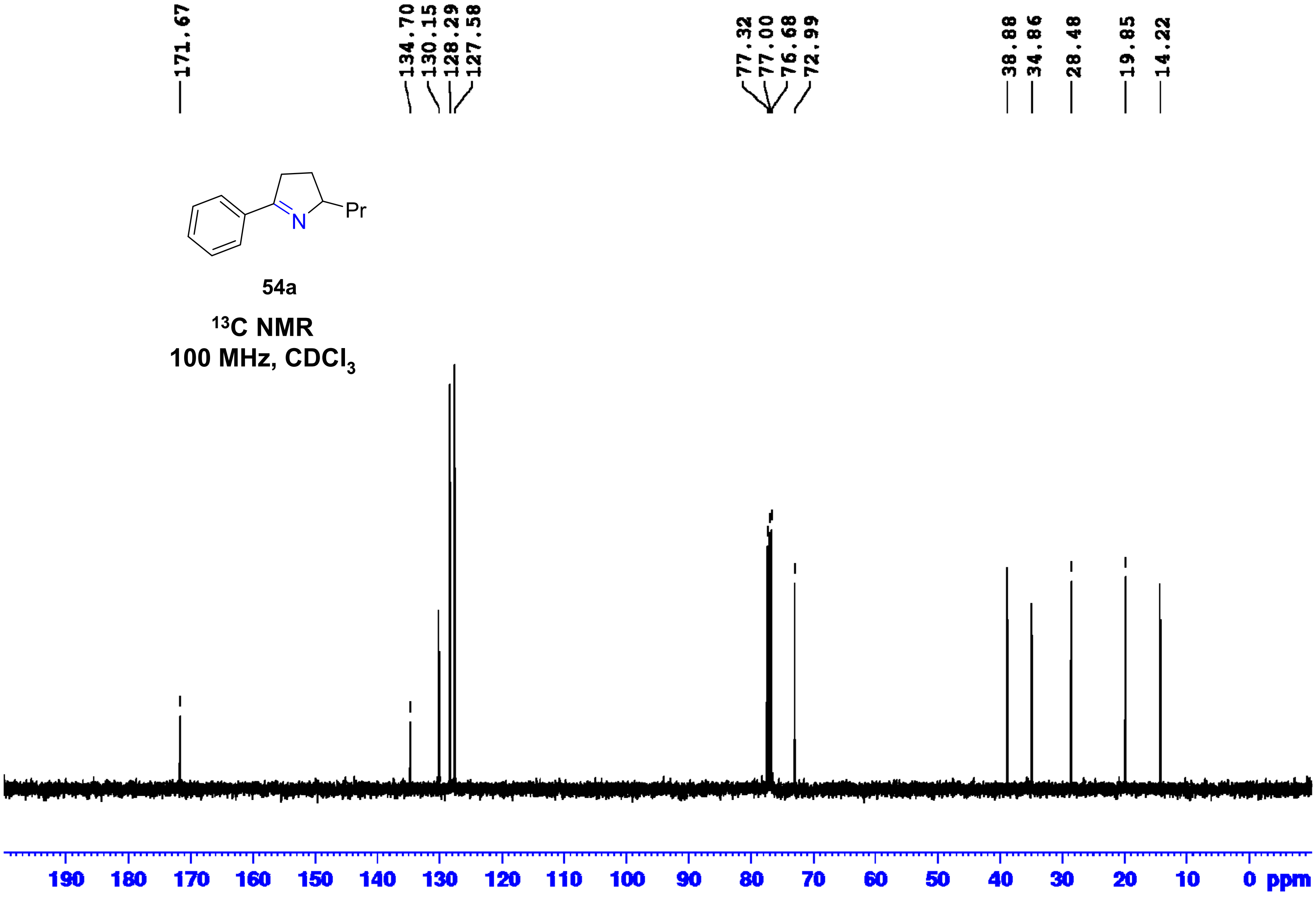
171.67
134.70
130.15
128.29
127.58
77.32
77.00
76.68
72.99
38.88
34.86
28.48
19.85
14.22
Pr
N
54a
13C NMR
100 MHz, CDCl3
190 180 170 160 150 140 130 120 110 100 90 80 70 60 50 40 30 20 10 0 ppm

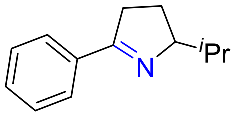

55a

^{1}H NMR,
400 MHz, $CDCl_3$

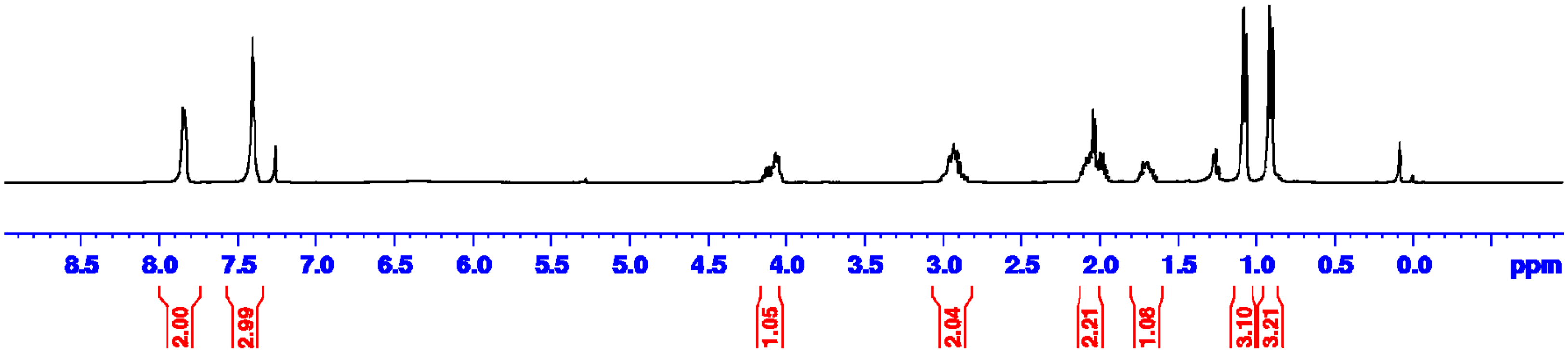

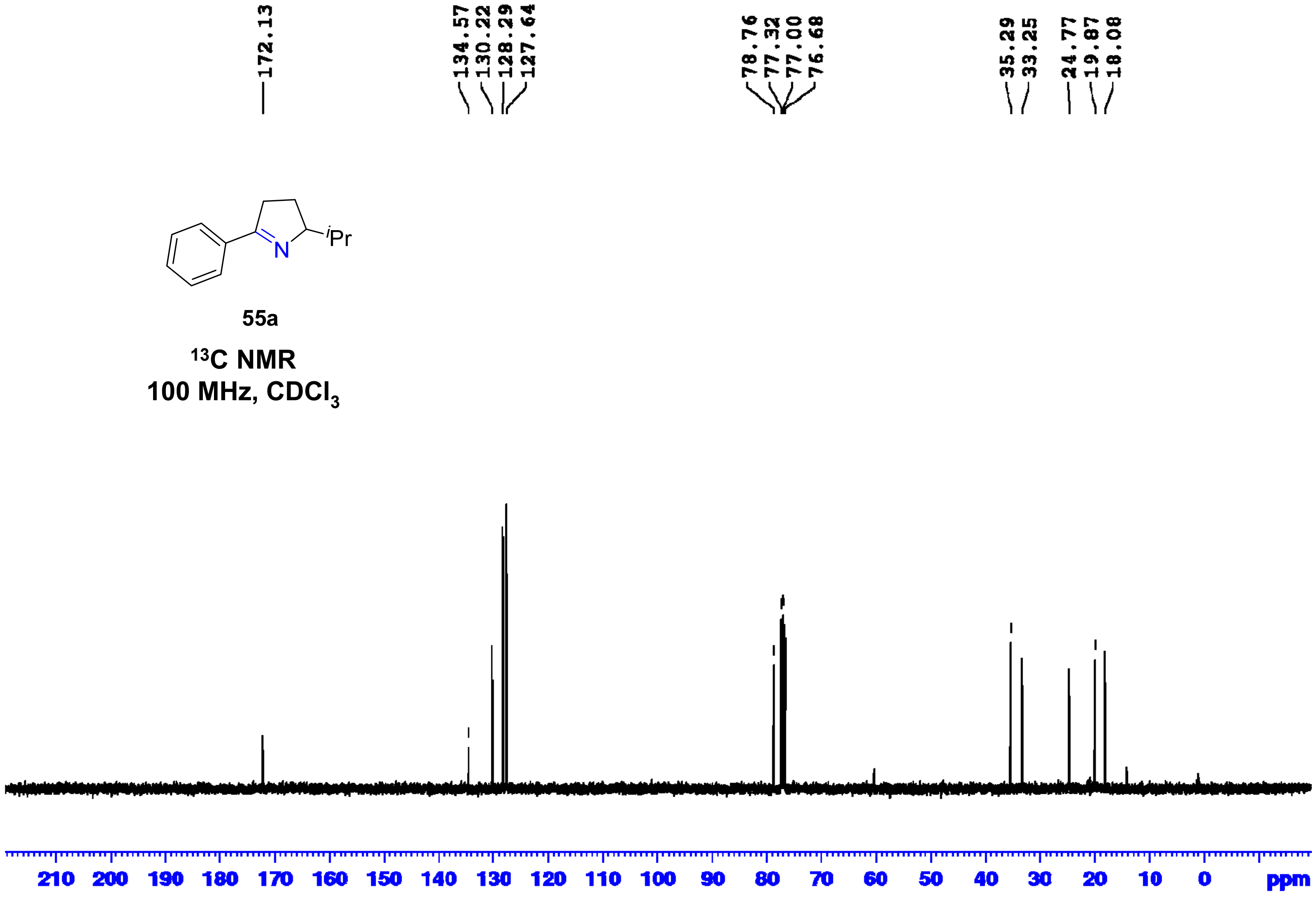
172.13
134.57
130.22
128.29
127.64
78.76
77.32
77.00
76.68
35.29
33.25
24.77
19.87
18.08
iPr
N
55a
13C NMR
100 MHz, CDCl3
210 200 190 180 170 160 150 140 130 120 110 100 90 80 70 60 50 40 30 20 10 0 ppm

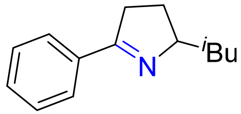

56a

^{1}H NMR,
400 MHz, $CDCl_3$

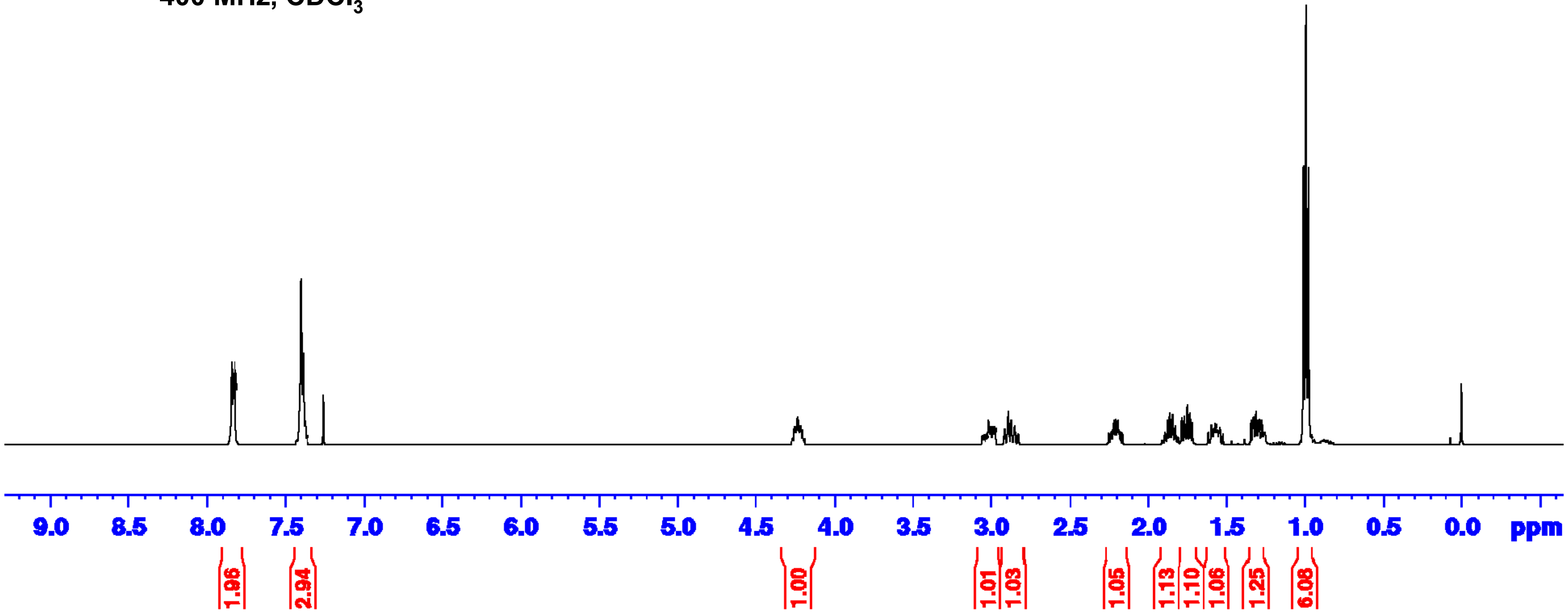

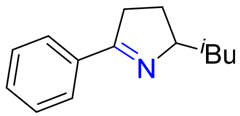

56a

^{13}C NMR
100 MHz, $CDCl_3$

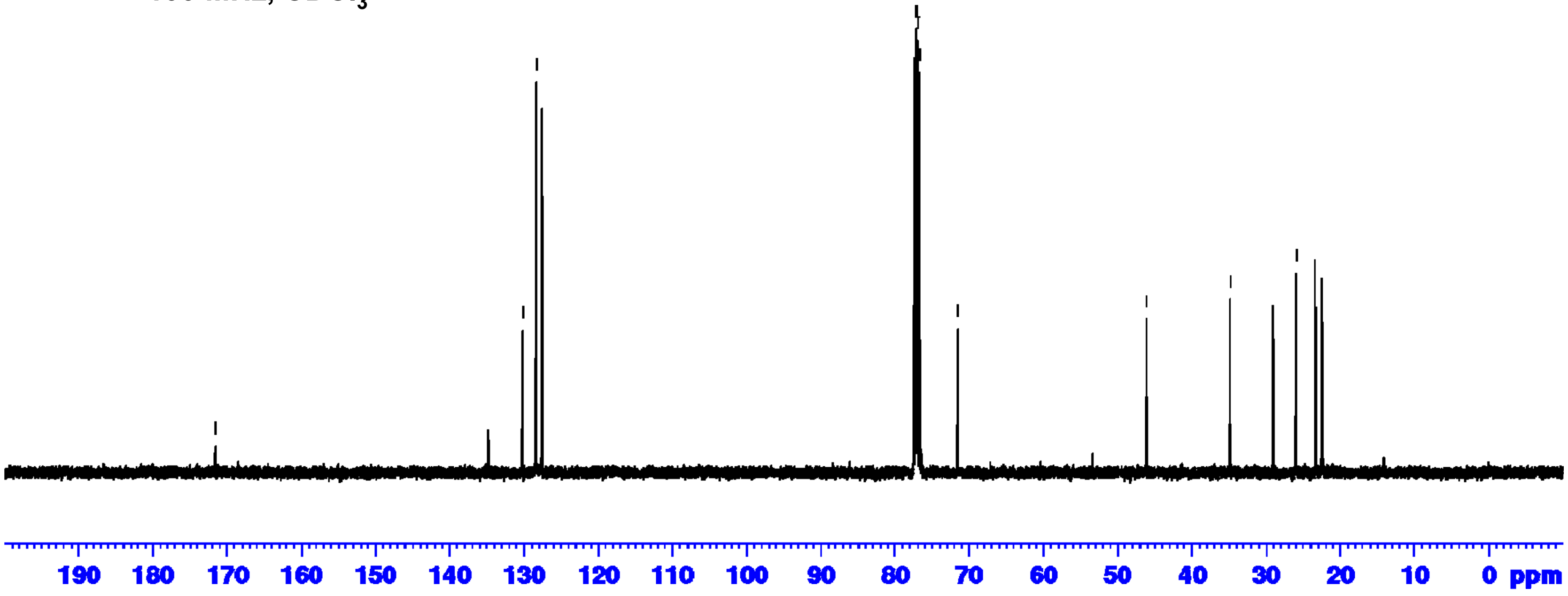

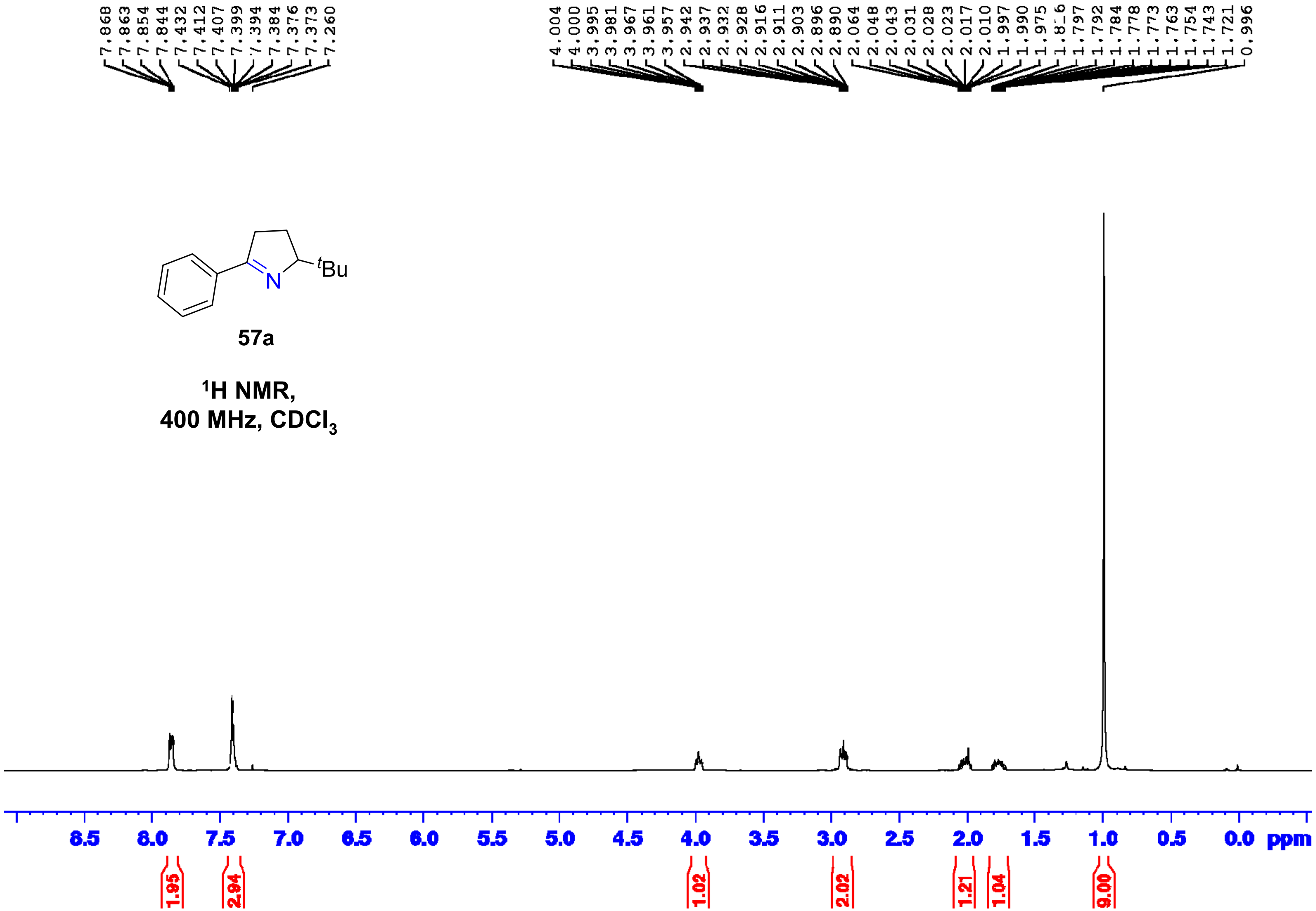
7.868
7.863
7.854
7.844
7.432
7.412
7.407
7.399
7.394
7.384
7.376
7.373
7.260
4.004
4.000
3.995
3.981
3.967
3.961
3.957
2.942
2.937
2.932
2.928
2.916
2.911
2.903
2.896
2.890
2.064
2.048
2.043
2.031
2.028
2.023
2.017
2.010
1.997
1.990
1.975
1.797
1.792
1.784
1.778
1.773
1.763
1.754
1.743
1.721
0.996
N
tBu
57a
1H NMR,
400 MHz, CDCl3
8.5
8.0
7.5
7.0
6.5
6.0
5.5
5.0
4.5
4.0
3.5
3.0
2.5
2.0
1.5
1.0
0.5
0.0
ppm
1.95
2.94
1.02
2.02
1.21
1.04
9.00

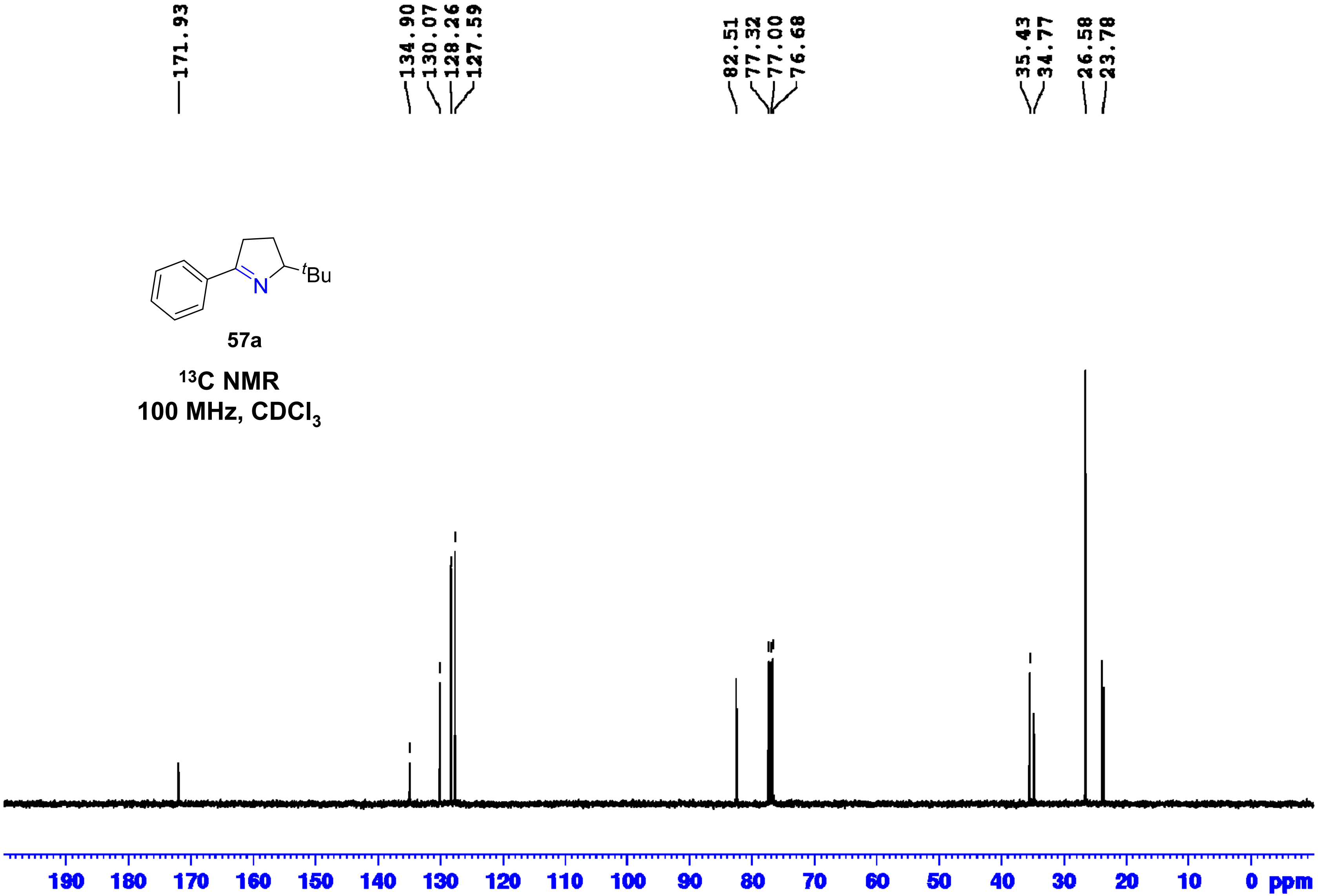

57a

^{13}C NMR
100 MHz, $CDCl_3$

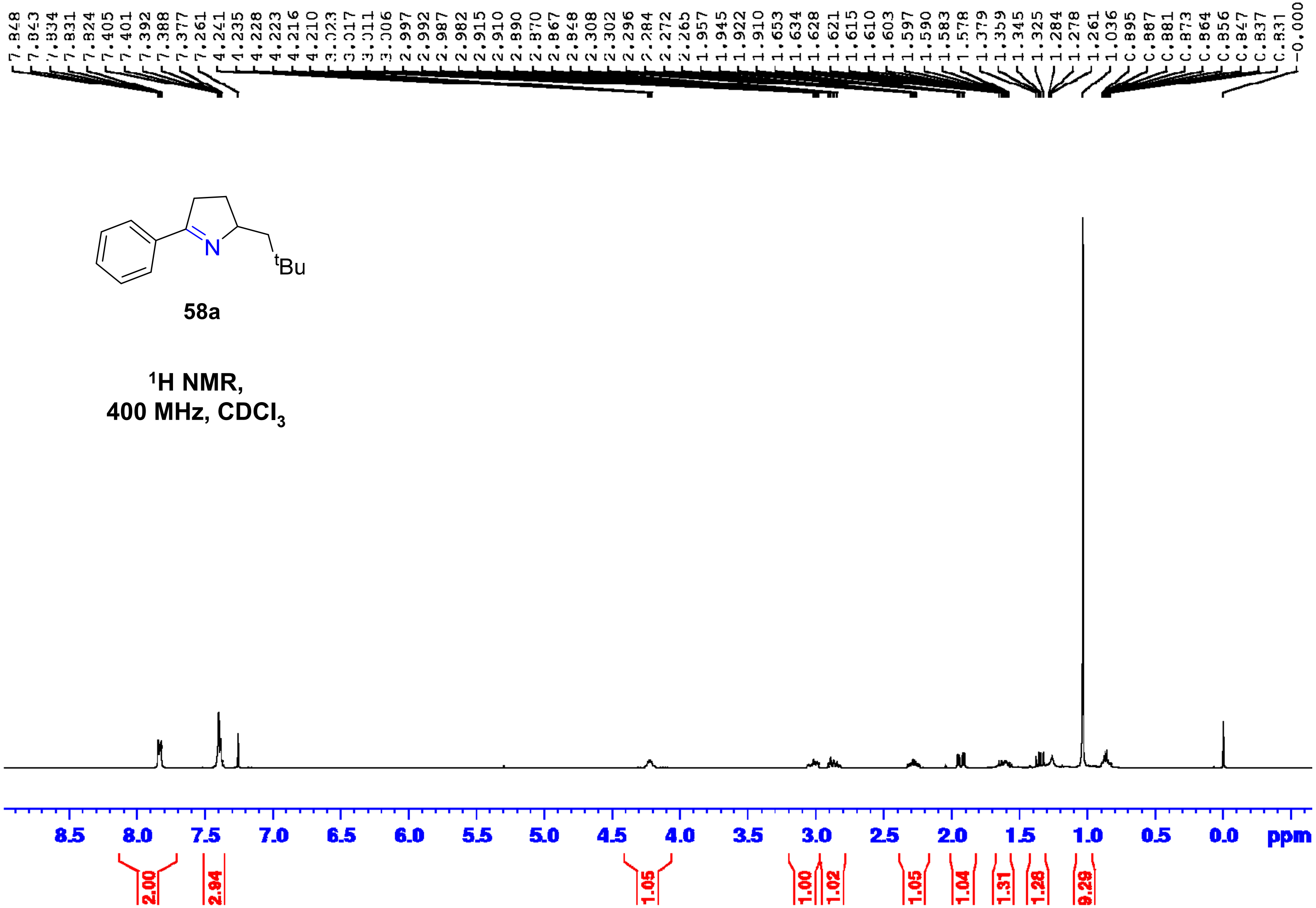

58a

^{1}H NMR, 400 MHz, $CDCl_3$

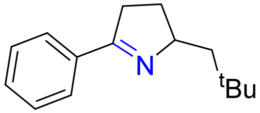

58a

^{13}C NMR
100 MHz, $CDCl_3$

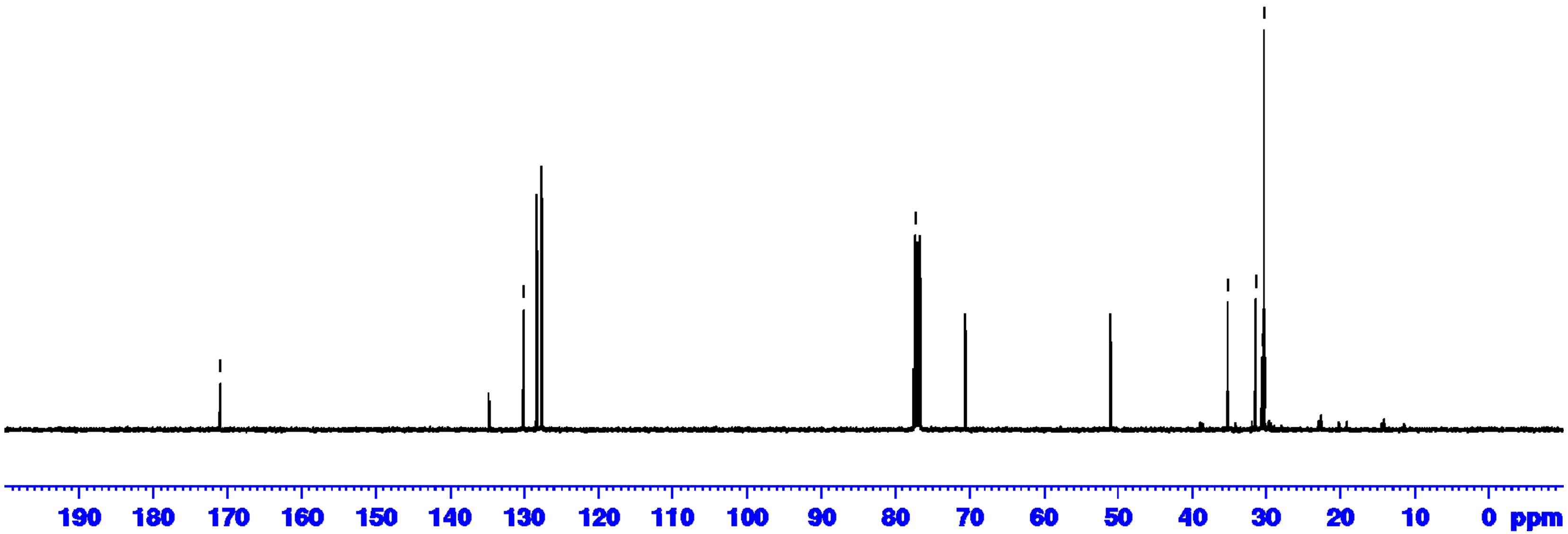

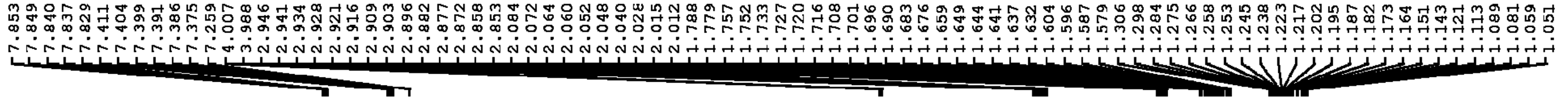

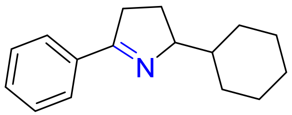

59a

^{1}H NMR,
400 MHz, $CDCl_3$

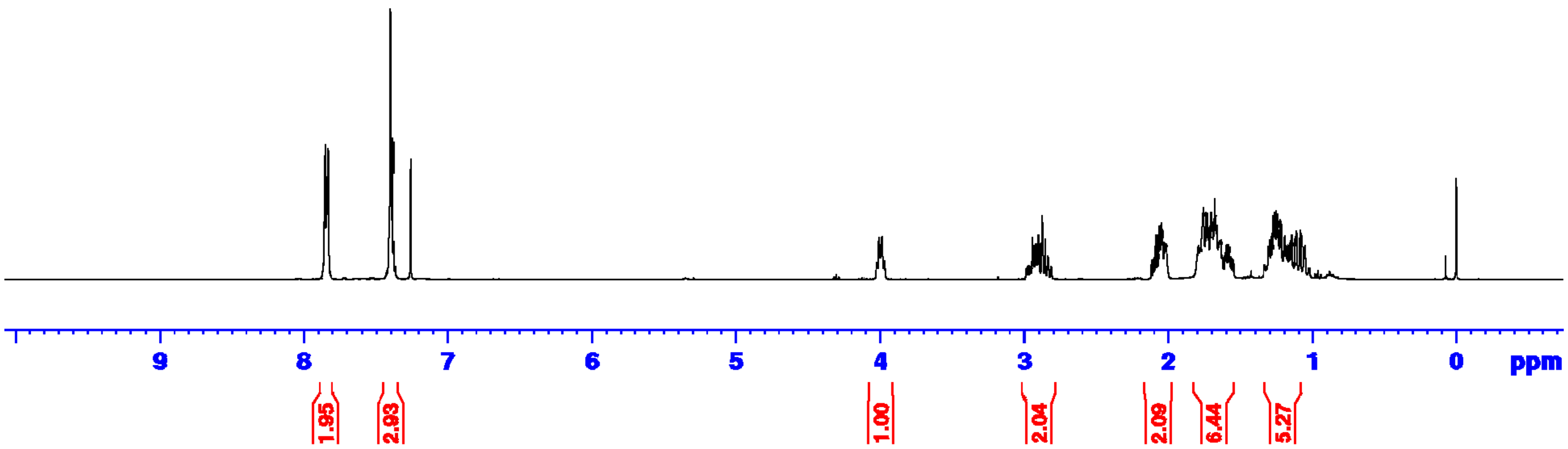

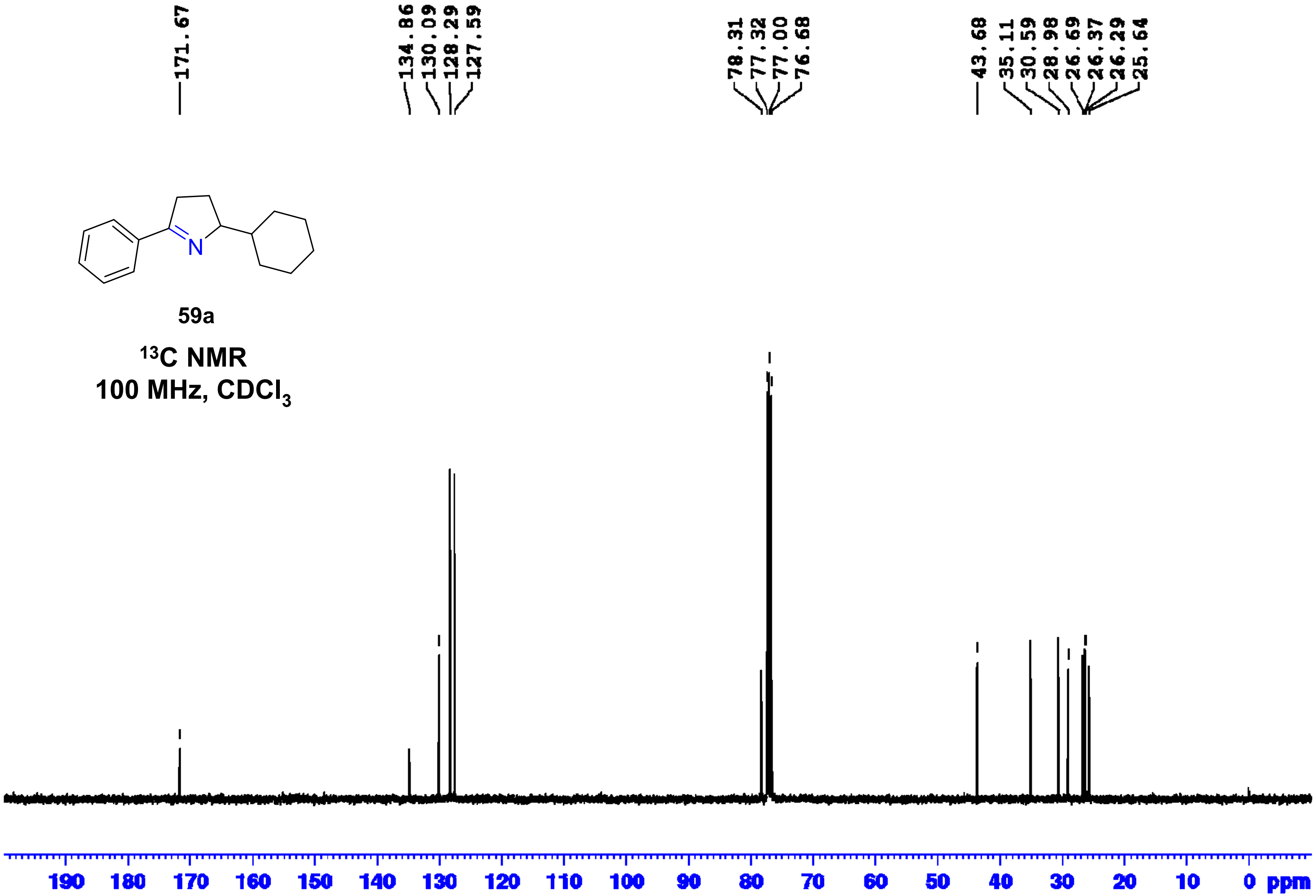
171.67
134.86
130.09
128.29
127.59
78.31
77.32
77.00
76.68
43.68
35.11
30.59
28.98
26.69
26.37
26.29
25.64
N
59a
13C NMR
100 MHz, CDCl3
190 180 170 160 150 140 130 120 110 100 90 80 70 60 50 40 30 20 10 0 ppm

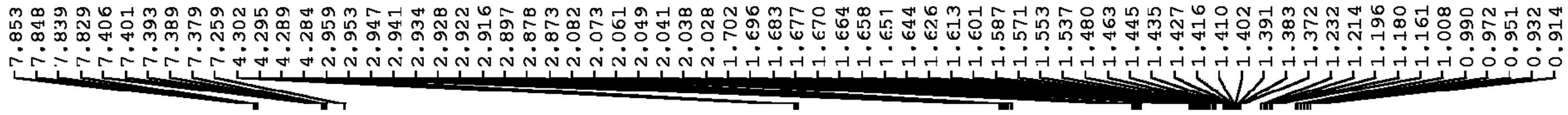

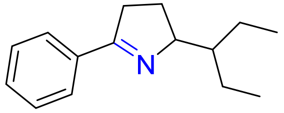

60a

^{1}H NMR,
400 MHz, $CDCl_3$

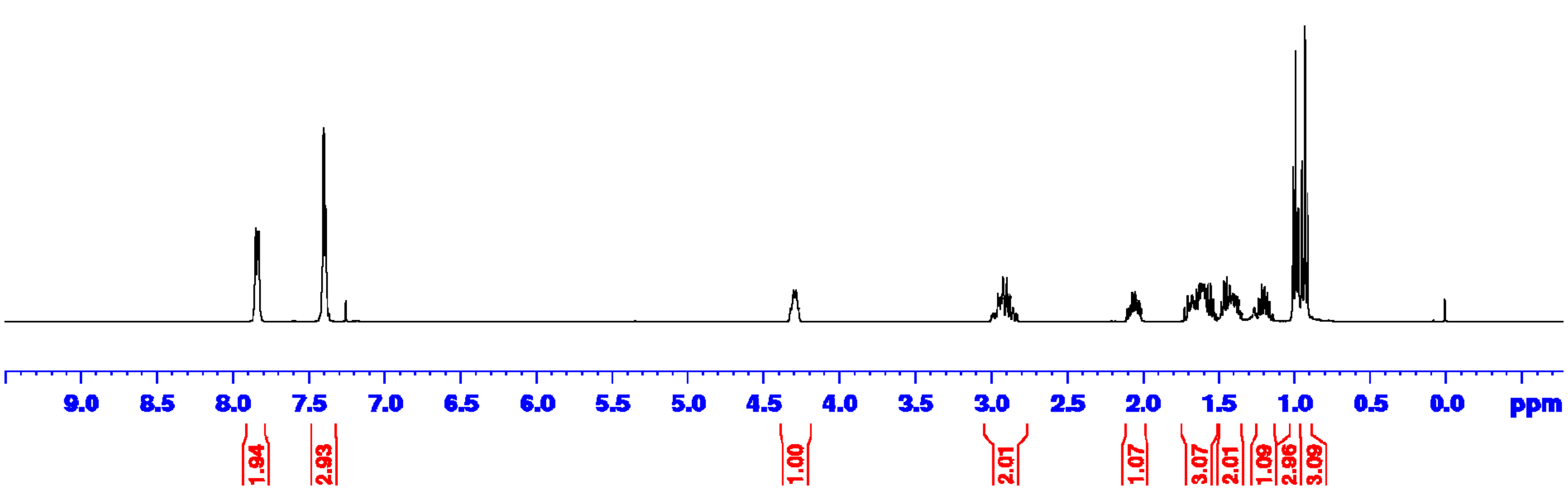

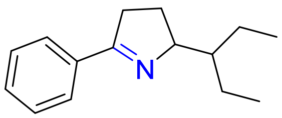

60a

^{13}C NMR
100 MHz, $CDCl_3$

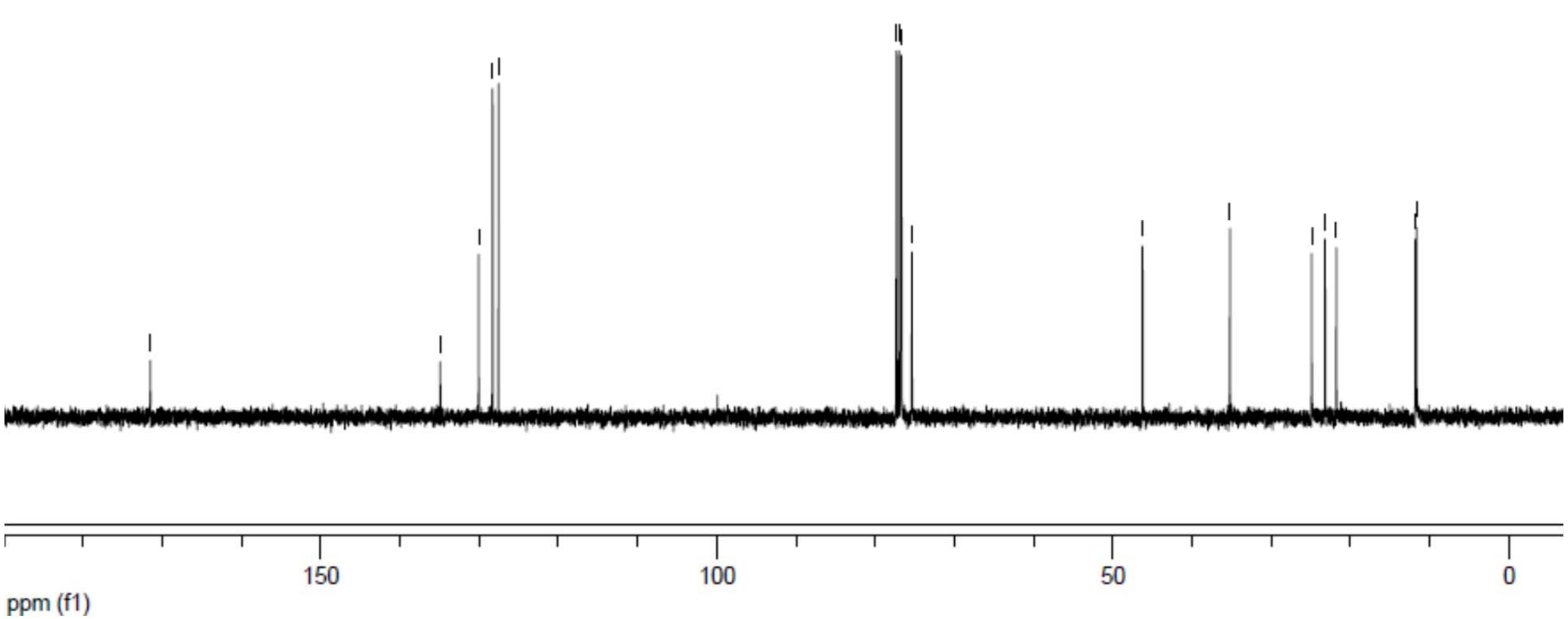

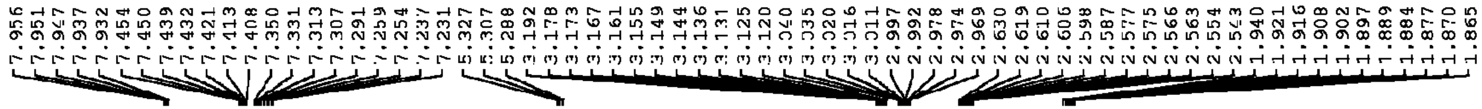

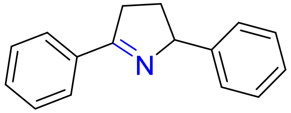

61a

^{1}H NMR,
400 MHz, $CDCl_3$

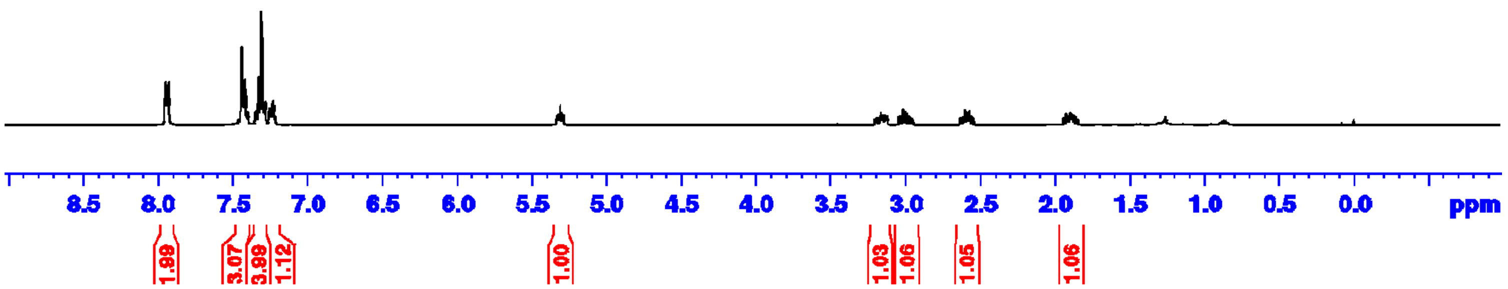

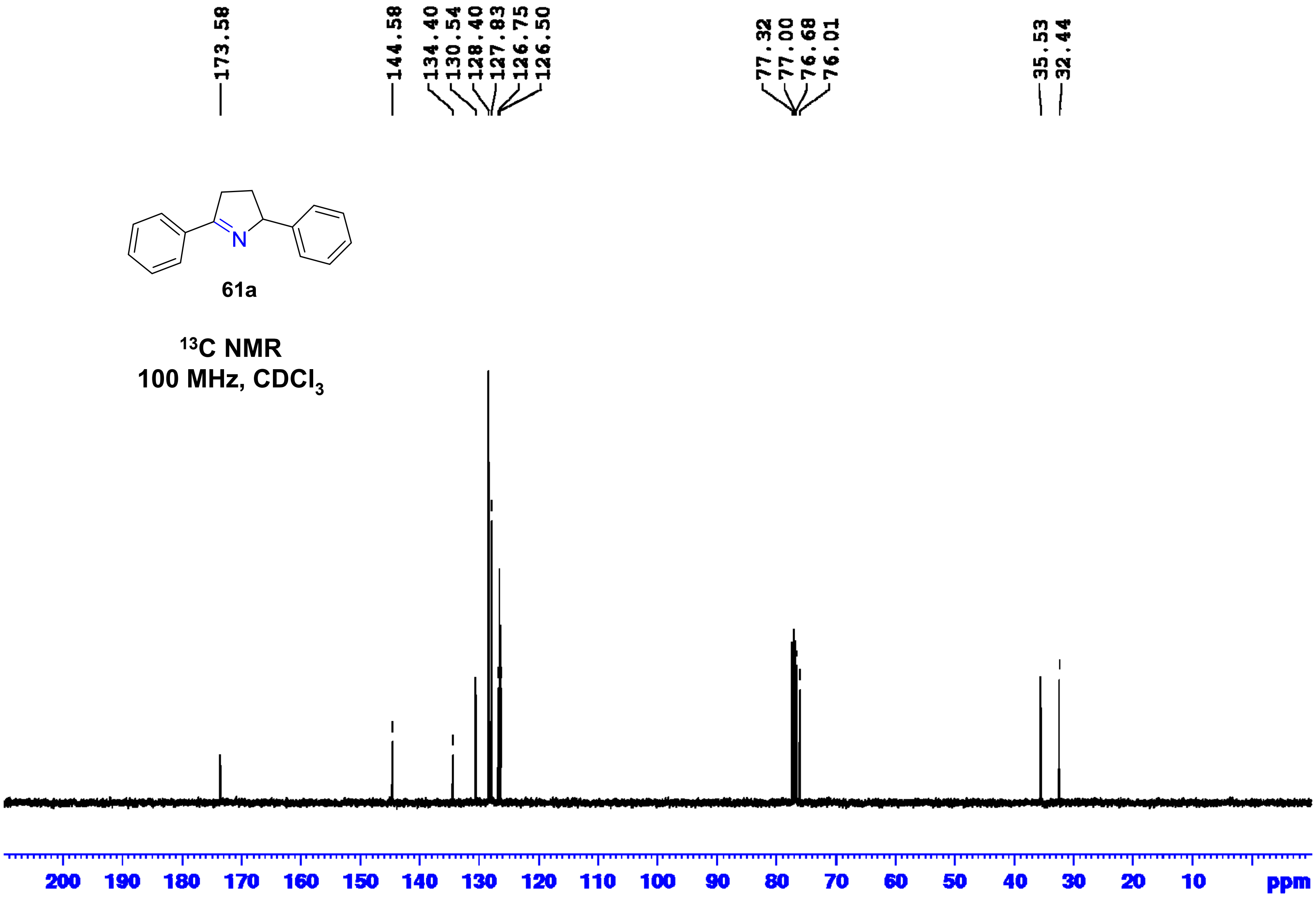
173.58
144.58
134.40
130.54
128.40
127.83
126.75
126.50
77.32
77.00
76.68
76.01
35.53
32.44
N
61a
13C NMR
100 MHz, CDCl3
200
190
180
170
160
150
140
130
120
110
100
90
80
70
60
50
40
30
20
10
ppm

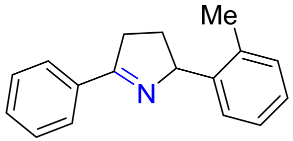

62a

^{1}H NMR,
400 MHz, $CDCl_3$

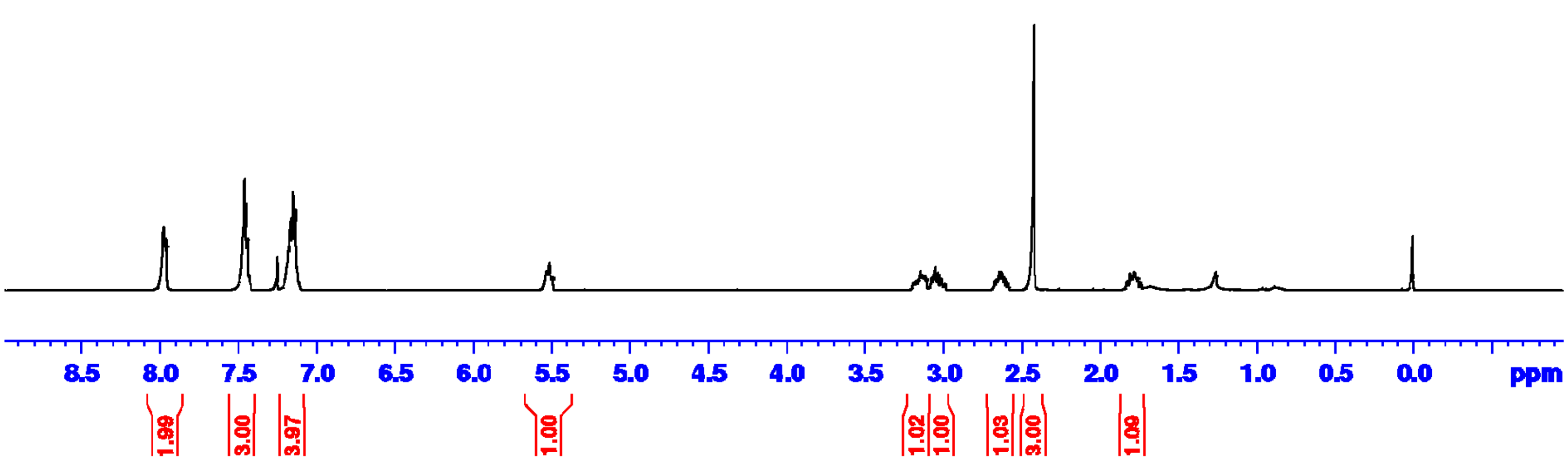

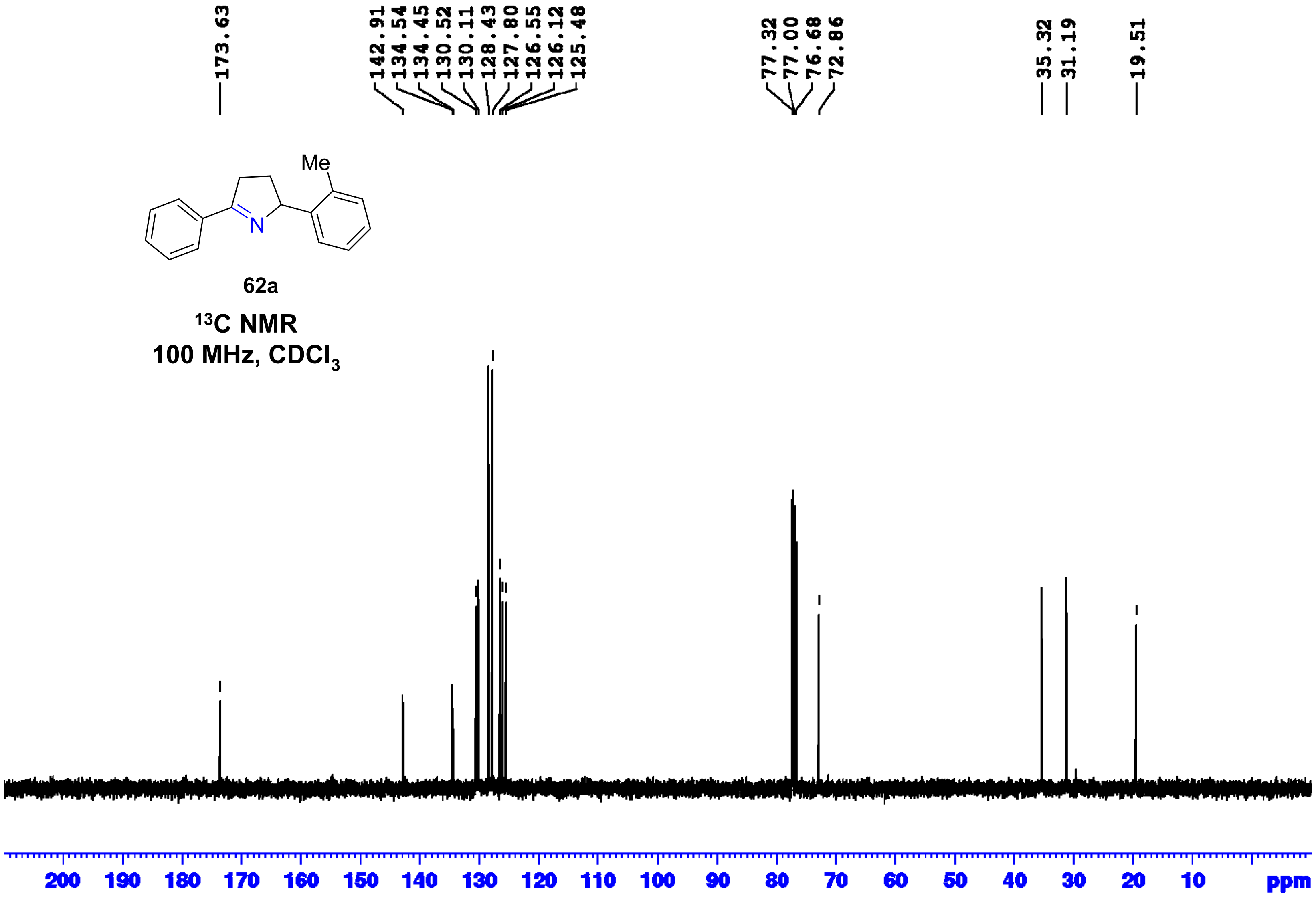
173.63
142.91
134.54
134.45
130.52
130.11
128.43
127.80
126.55
126.12
125.48
77.32
77.00
76.68
72.86
35.32
31.19
19.51
Me
N
62a
^{13}C NMR
100 MHz, $CDCl_3$
200 190 180 170 160 150 140 130 120 110 100 90 80 70 60 50 40 30 20 10 ppm

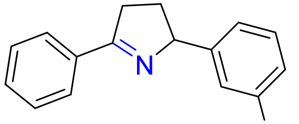

63a

^{1}H NMR,
400 MHz, $CDCl_3$

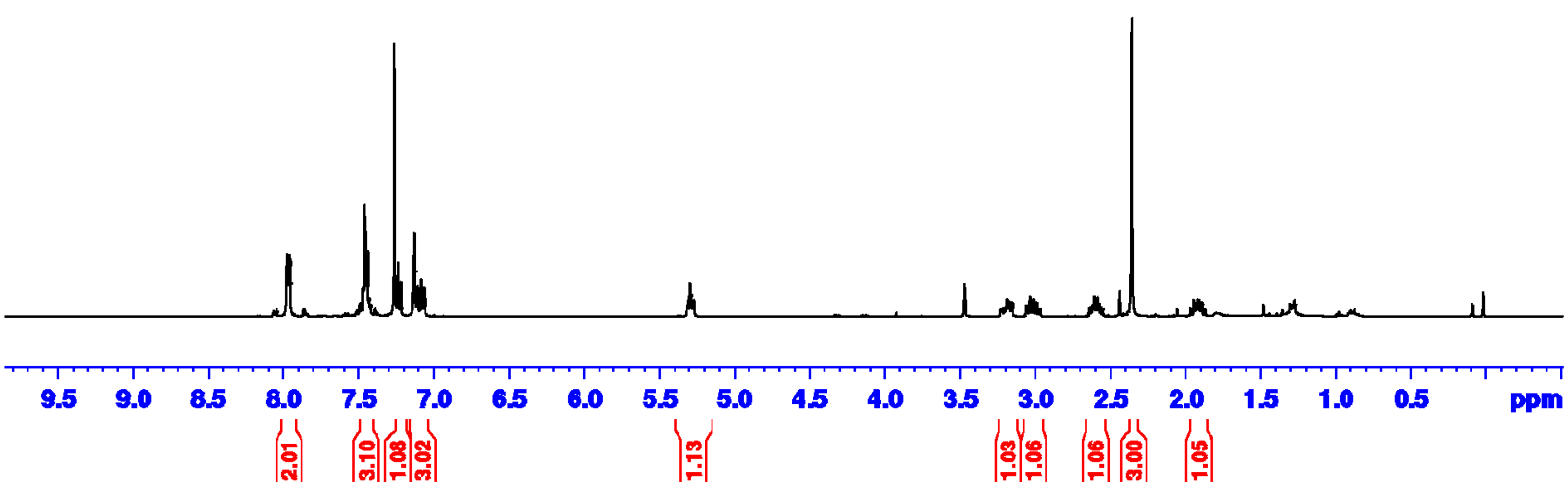

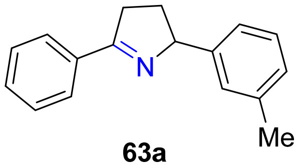

63a

^{13}C NMR
100 MHz, $CDCl_3$

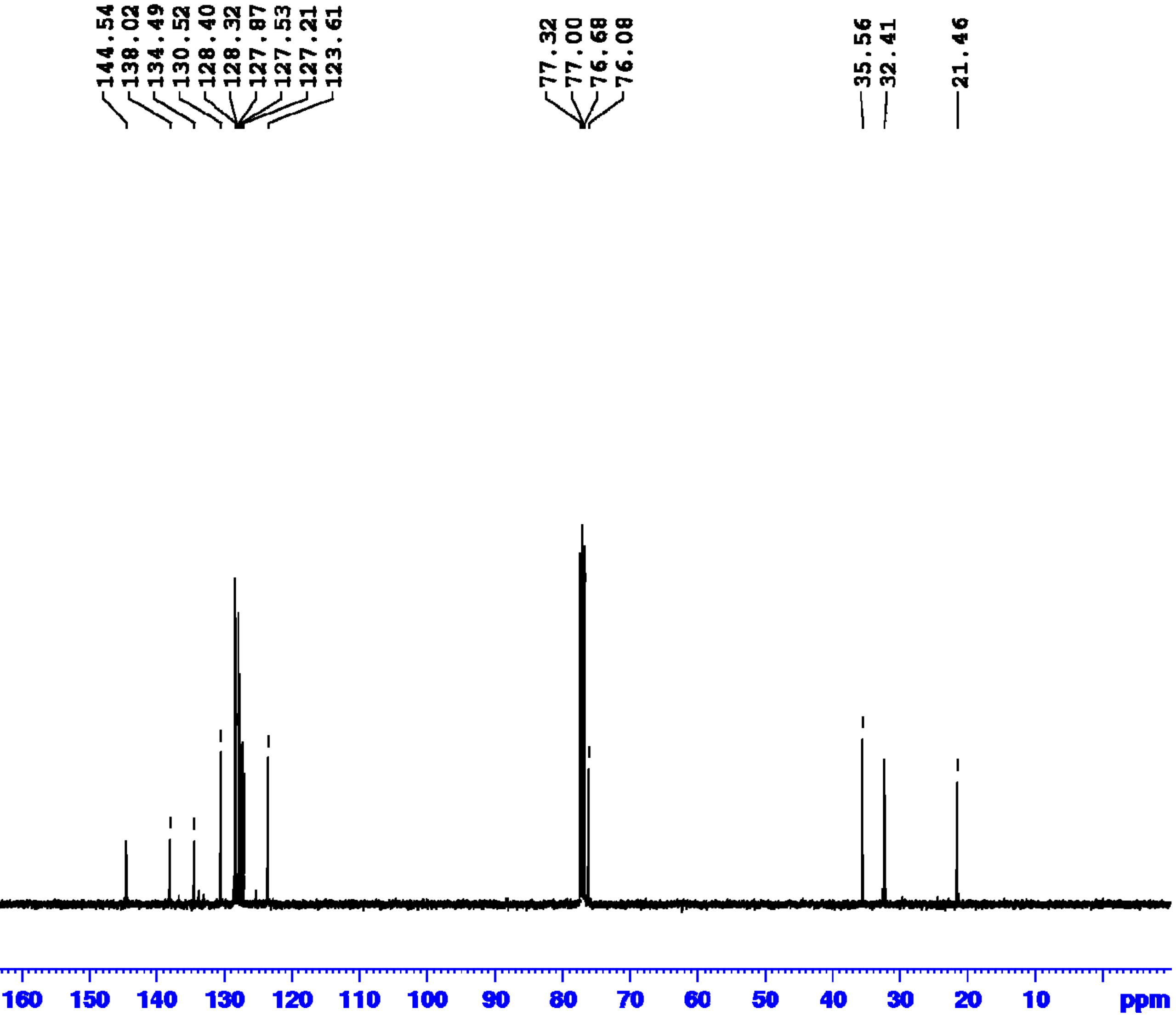

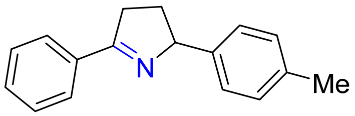

64a

^{1}H NMR,
400 MHz, $CDCl_3$

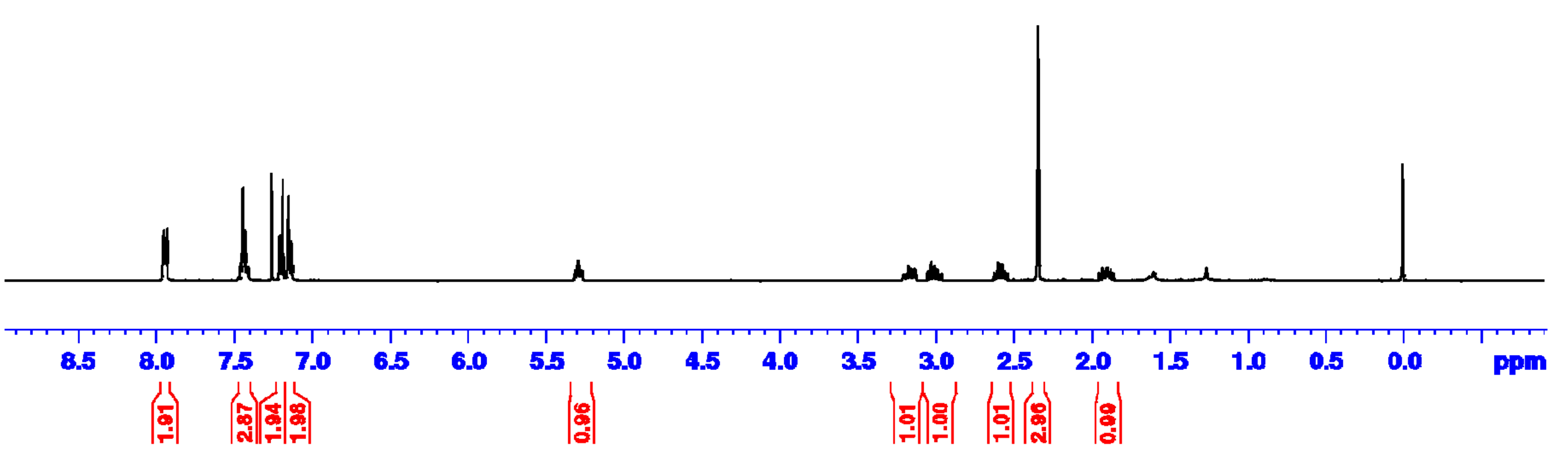

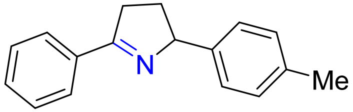

64a

^{13}C NMR
100 MHz, $CDCl_3$

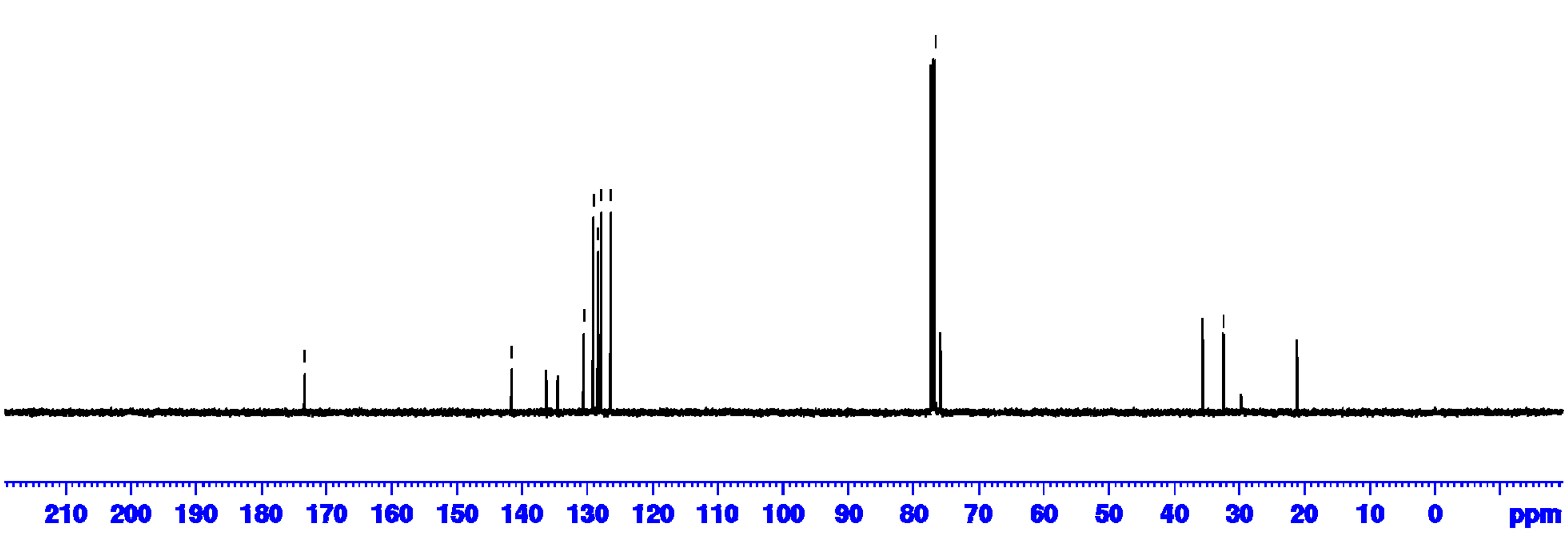

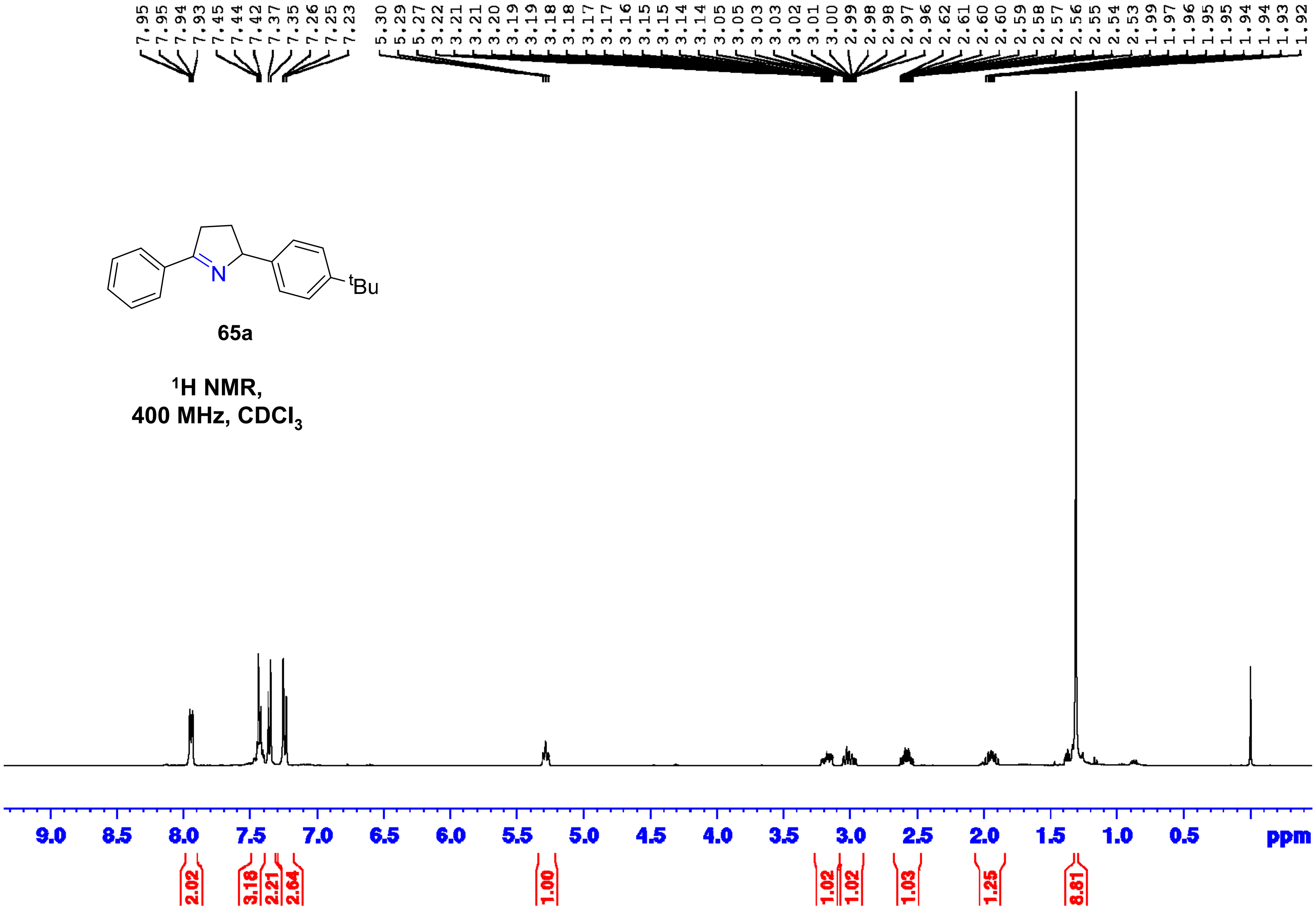

65a

^{1}H NMR,
400 MHz, CDCl$_3$

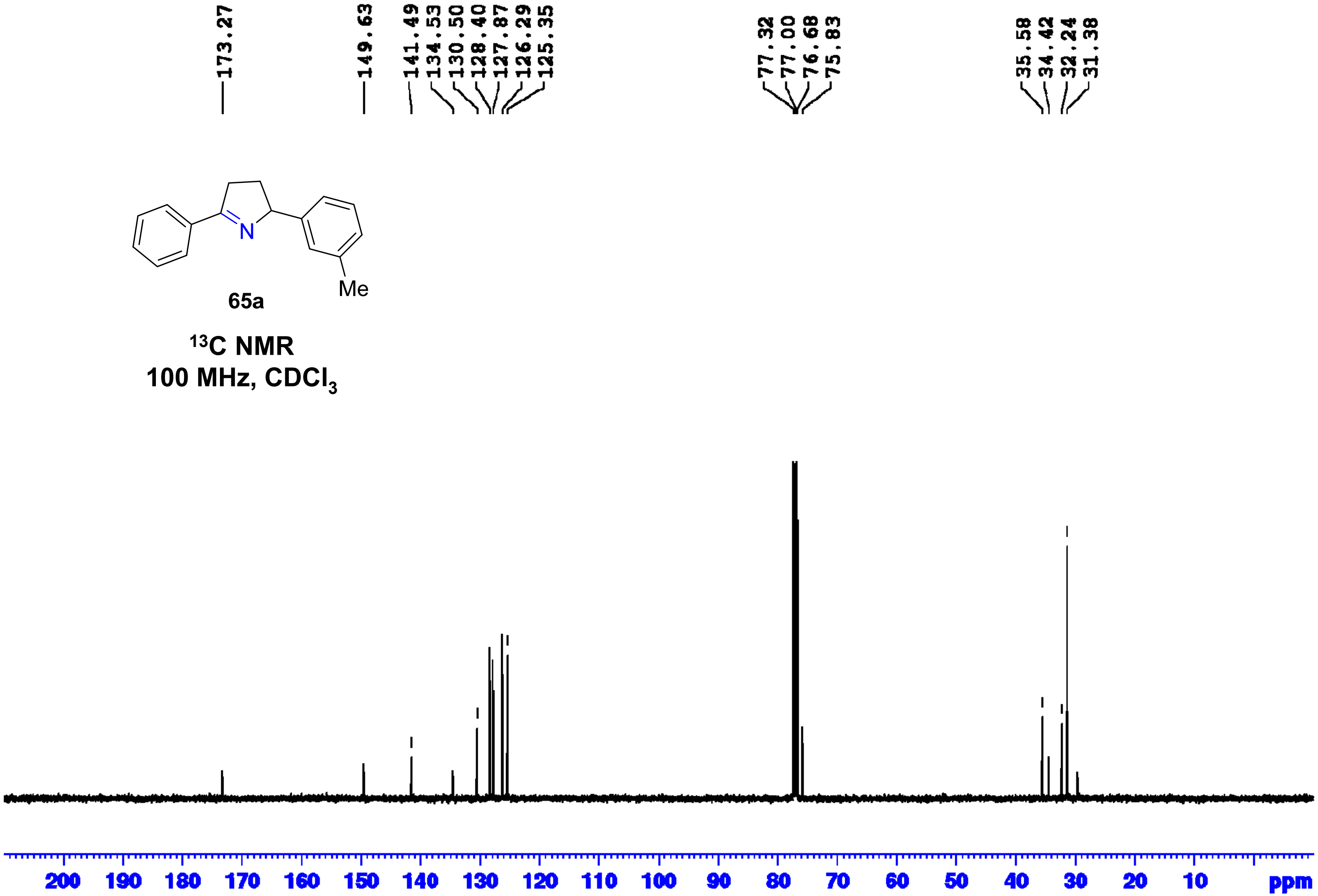
173.27
149.63
141.49
134.53
130.50
128.40
127.87
126.29
125.35
77.32
77.00
76.68
75.83
35.58
34.42
32.24
31.38
N
Me
65a
^{13}C NMR
100 MHz, $CDCl_3$
200 190 180 170 160 150 140 130 120 110 100 90 80 70 60 50 40 30 20 10 ppm

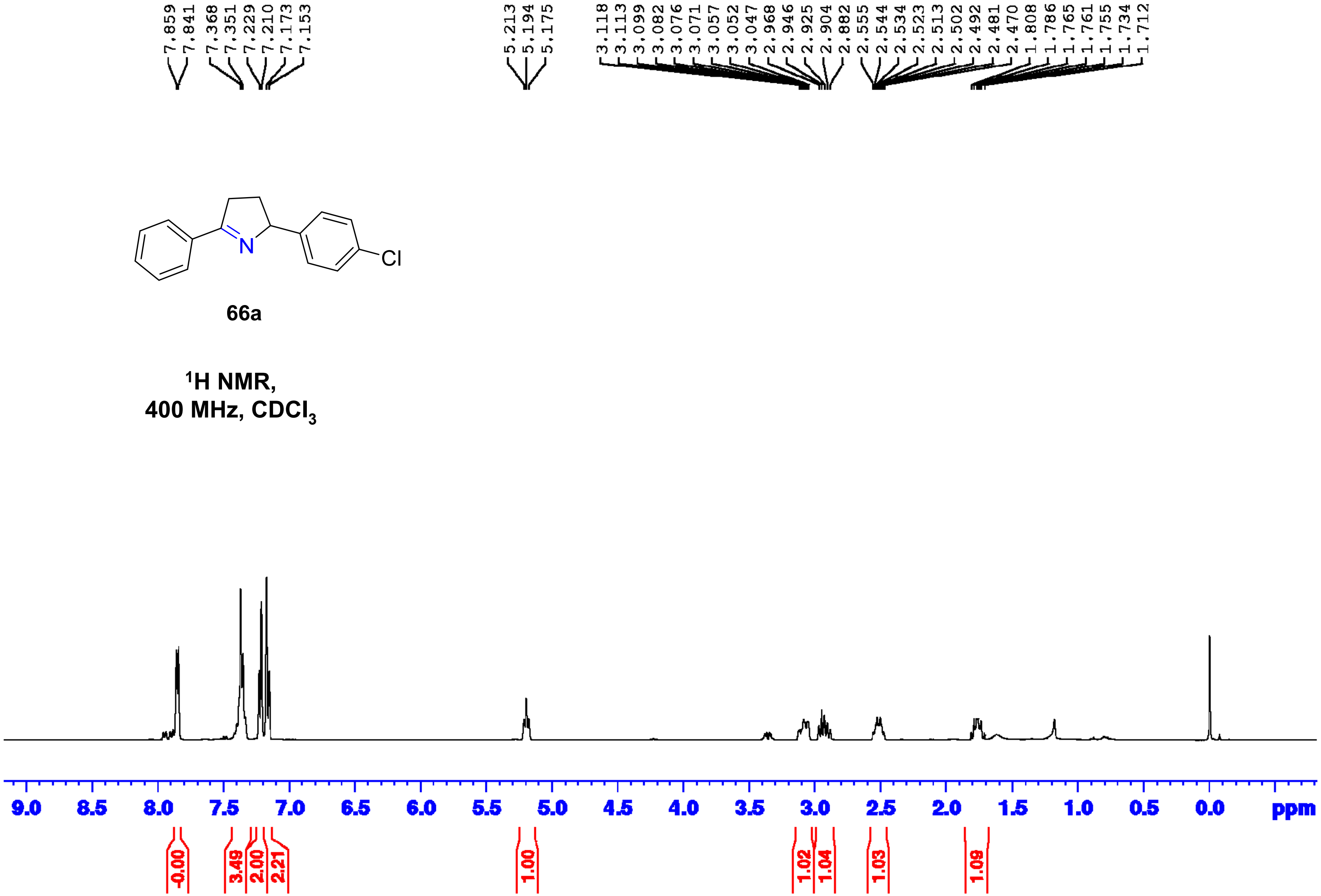
7.859
7.841
7.368
7.351
7.229
7.210
7.173
7.153
5.213
5.194
5.175
3.118
3.113
3.099
3.082
3.076
3.071
3.057
3.052
3.047
2.968
2.946
2.925
2.904
2.882
2.555
2.544
2.534
2.523
2.513
2.502
2.492
2.481
2.470
1.808
1.786
1.765
1.761
1.755
1.734
1.712
N
Cl
66a
1H NMR,
400 MHz, CDCl3
9.0 8.5 8.0 7.5 7.0 6.5 6.0 5.5 5.0 4.5 4.0 3.5 3.0 2.5 2.0 1.5 1.0 0.5 0.0 ppm
-0.00
3.49
2.00
2.21
1.00
1.02
1.04
1.03
1.09

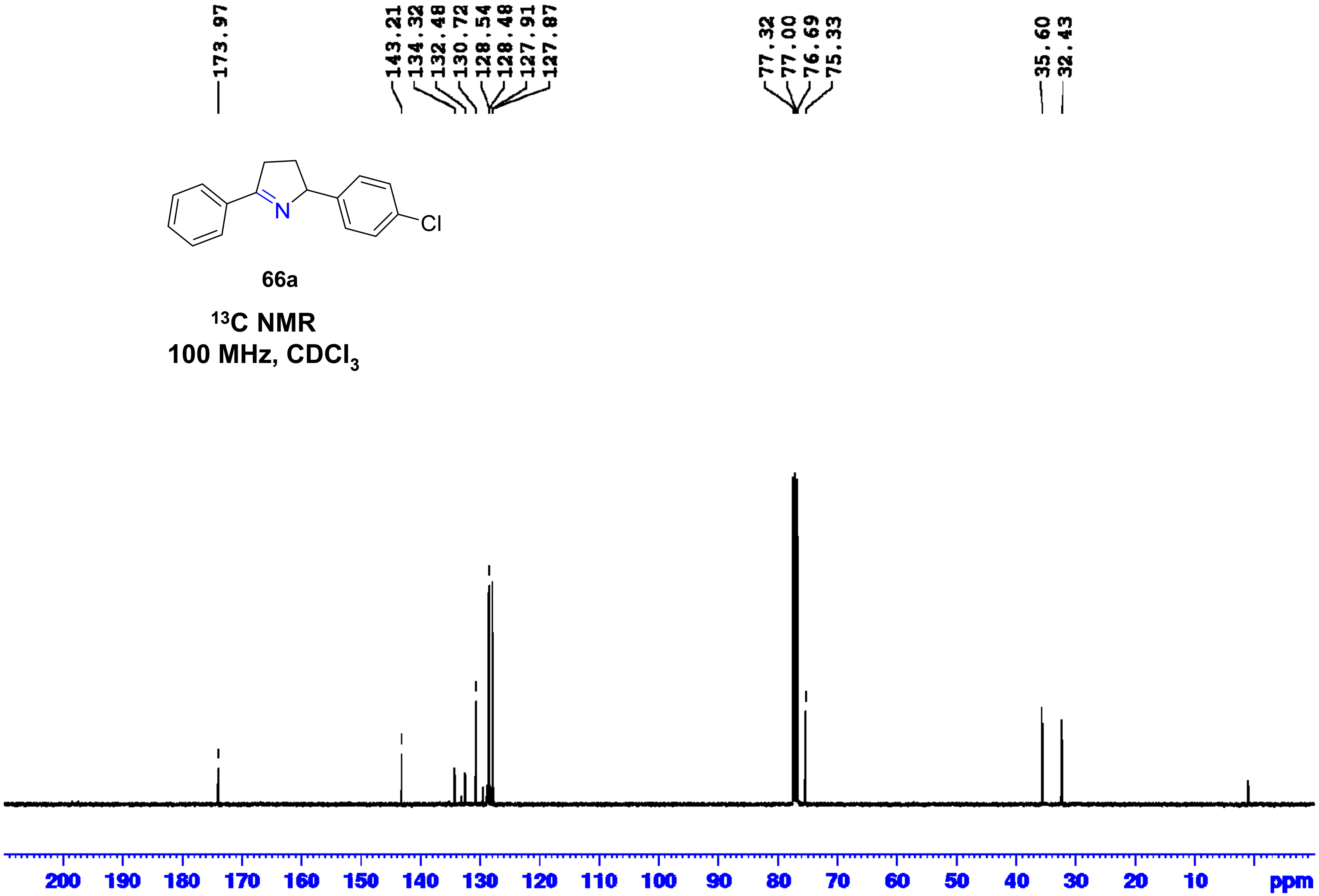
173.97
143.21
134.32
132.48
130.72
128.54
128.48
127.91
127.87
77.32
77.00
76.69
75.33
35.60
32.43
N
Cl
66a
^{13}C NMR
100 MHz, $CDCl_3$
200 190 180 170 160 150 140 130 120 110 100 90 80 70 60 50 40 30 20 10 ppm

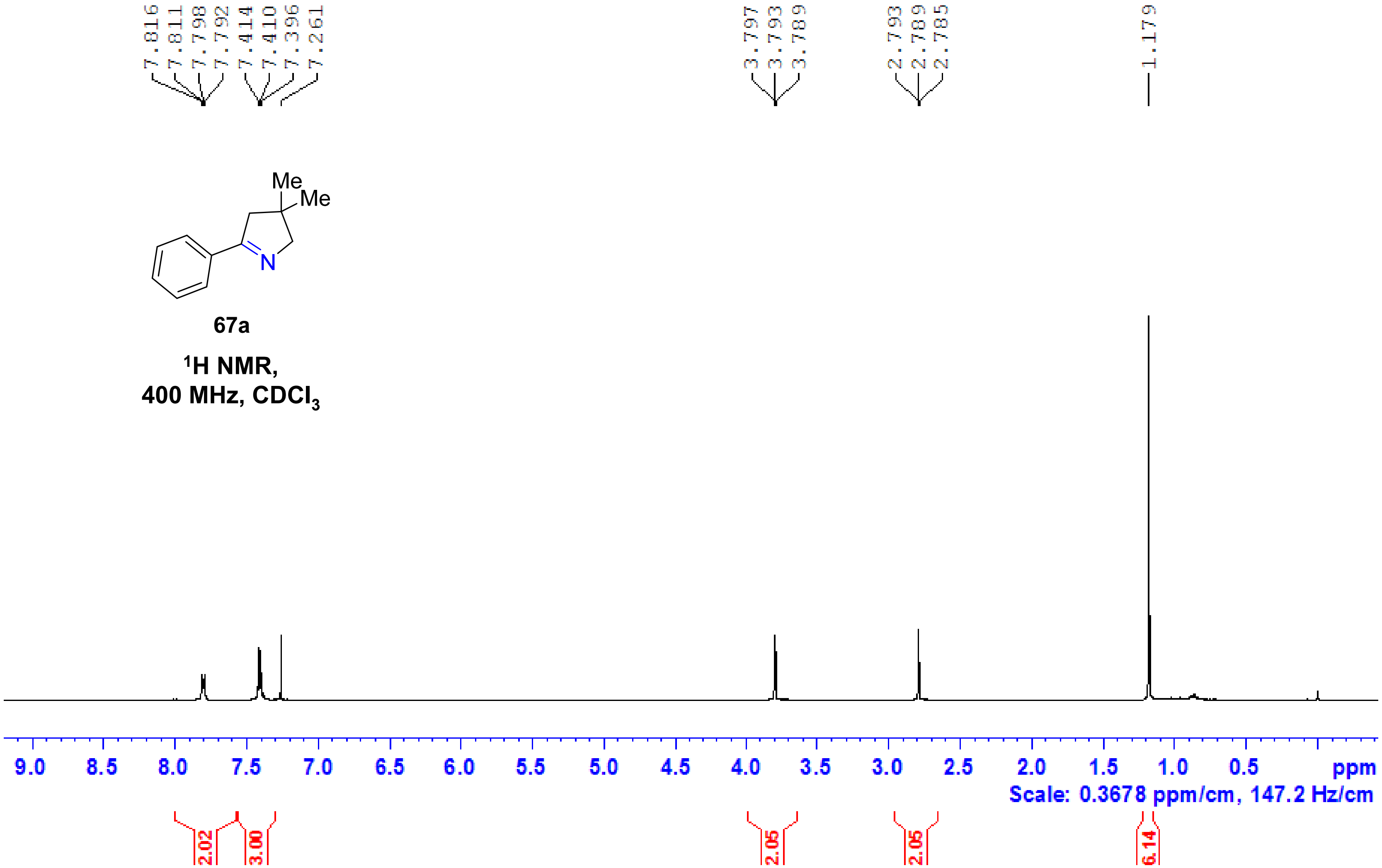
7.816
7.811
7.798
7.792
7.414
7.410
7.396
7.261
3.797
3.793
3.789
2.793
2.789
2.785
1.179
Me
Me
N
67a
^{1}H NMR,
400 MHz, $CDCl_3$
9.0
8.5
8.0
7.5
7.0
6.5
6.0
5.5
5.0
4.5
4.0
3.5
3.0
2.5
2.0
1.5
1.0
0.5
ppm
Scale: 0.3678 ppm/cm, 147.2 Hz/cm
2.02
3.00
2.05
2.05
6.14

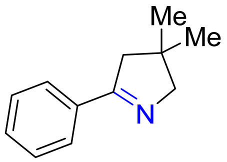

67a

^{13}C NMR
100 MHz, $CDCl_3$

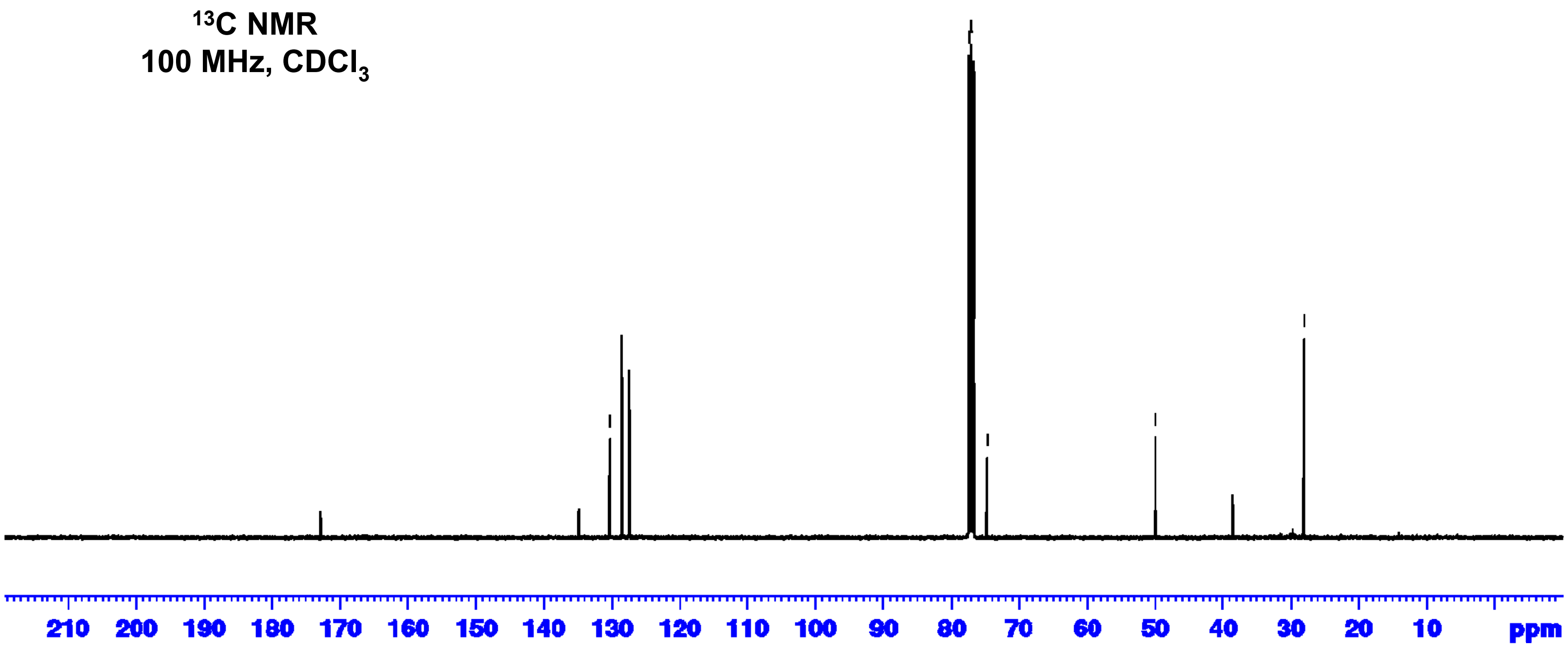

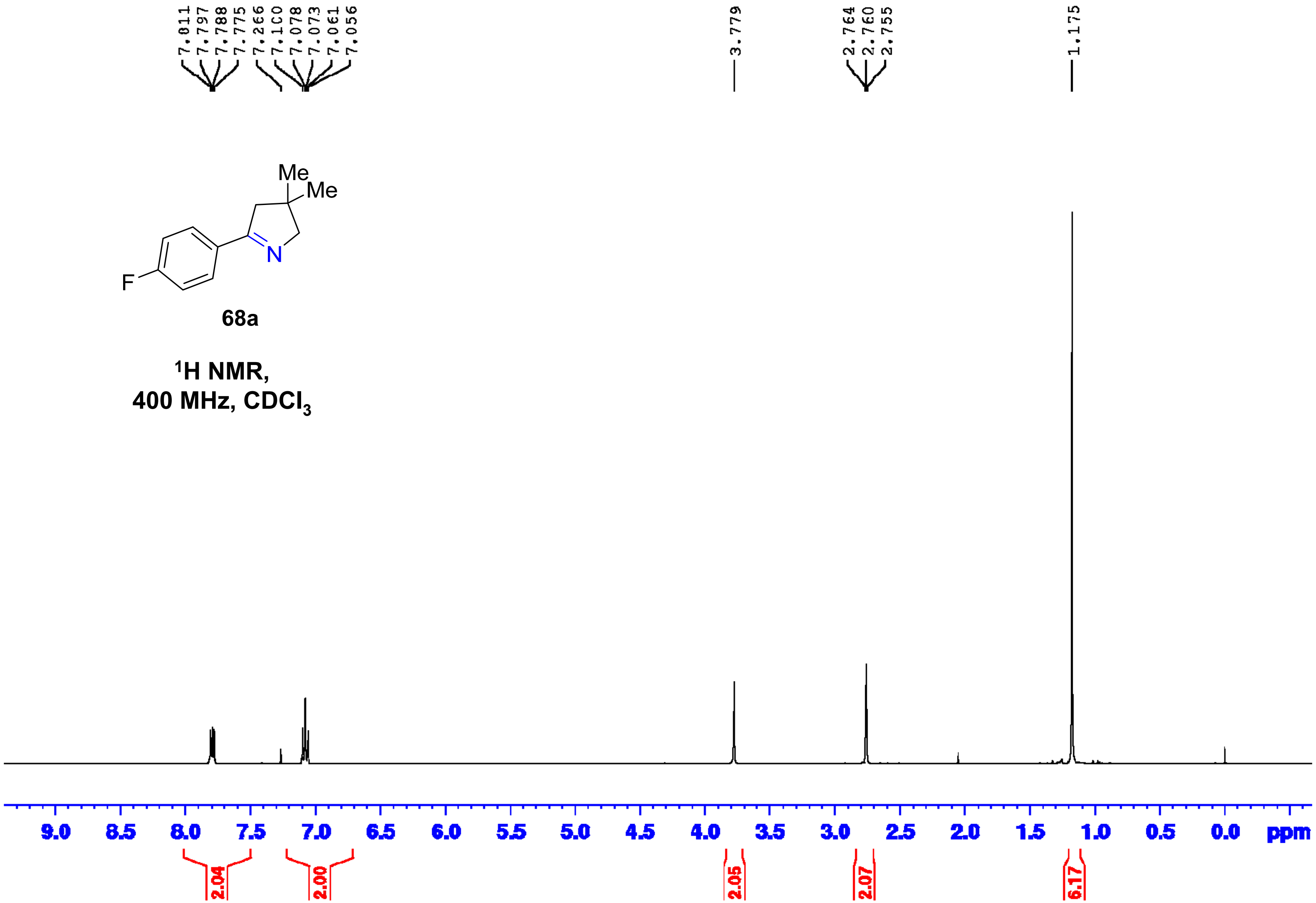
7.811
7.797
7.788
7.775
7.266
7.100
7.078
7.073
7.061
7.056
3.779
2.764
2.760
2.755
1.175
Me
Me
F
N
68a
1H NMR,
400 MHz, CDCl3
9.0
8.5
8.0
7.5
7.0
6.5
6.0
5.5
5.0
4.5
4.0
3.5
3.0
2.5
2.0
1.5
1.0
0.5
0.0
ppm
2.04
2.00
2.05
2.07
6.17

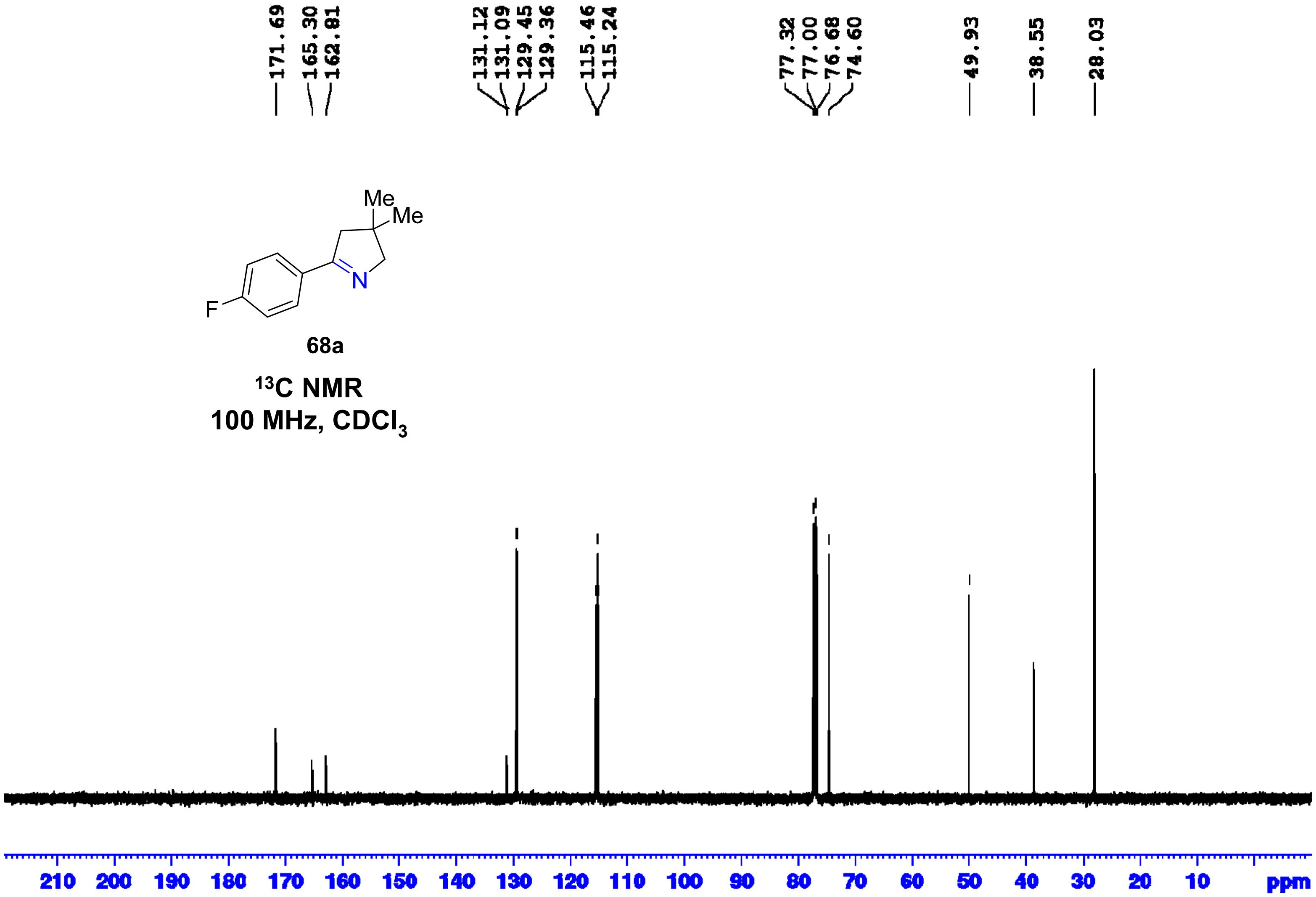
171.69
165.30
162.81
131.12
131.09
129.45
129.36
115.46
115.24
77.32
77.00
76.68
74.60
49.93
38.55
28.03
Me
Me
F
N
68a
13C NMR
100 MHz, CDCl3
210 200 190 180 170 160 150 140 130 120 110 100 90 80 70 60 50 40 30 20 10 ppm

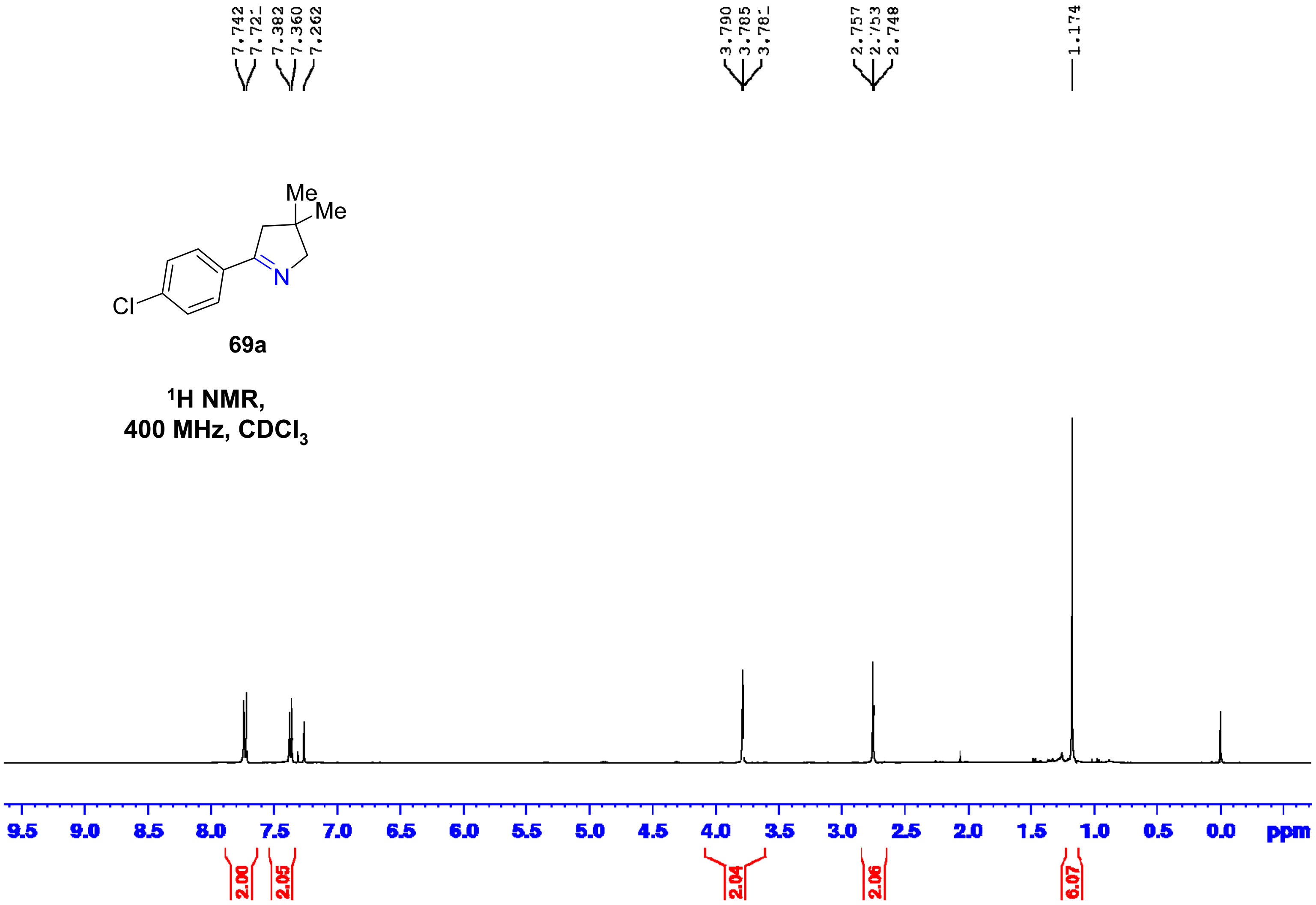

69a

^{1}H NMR,
400 MHz, $CDCl_3$

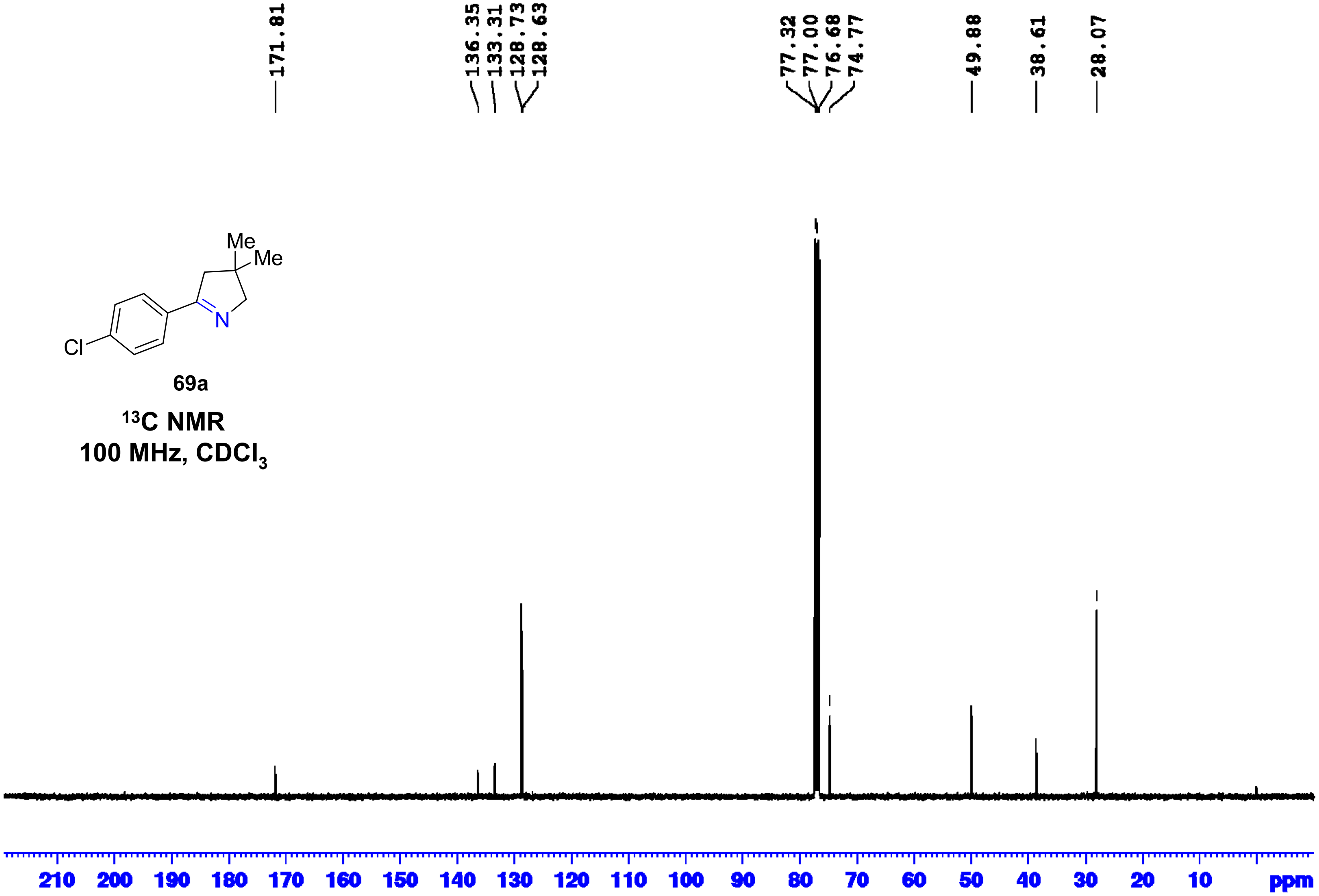
69a
13C NMR
100 MHz, CDCl3
171.81
136.35
133.31
128.73
128.63
77.32
77.00
76.68
74.77
49.88
38.61
28.07
210
200
190
180
170
160
150
140
130
120
110
100
90
80
70
60
50
40
30
20
10
ppm

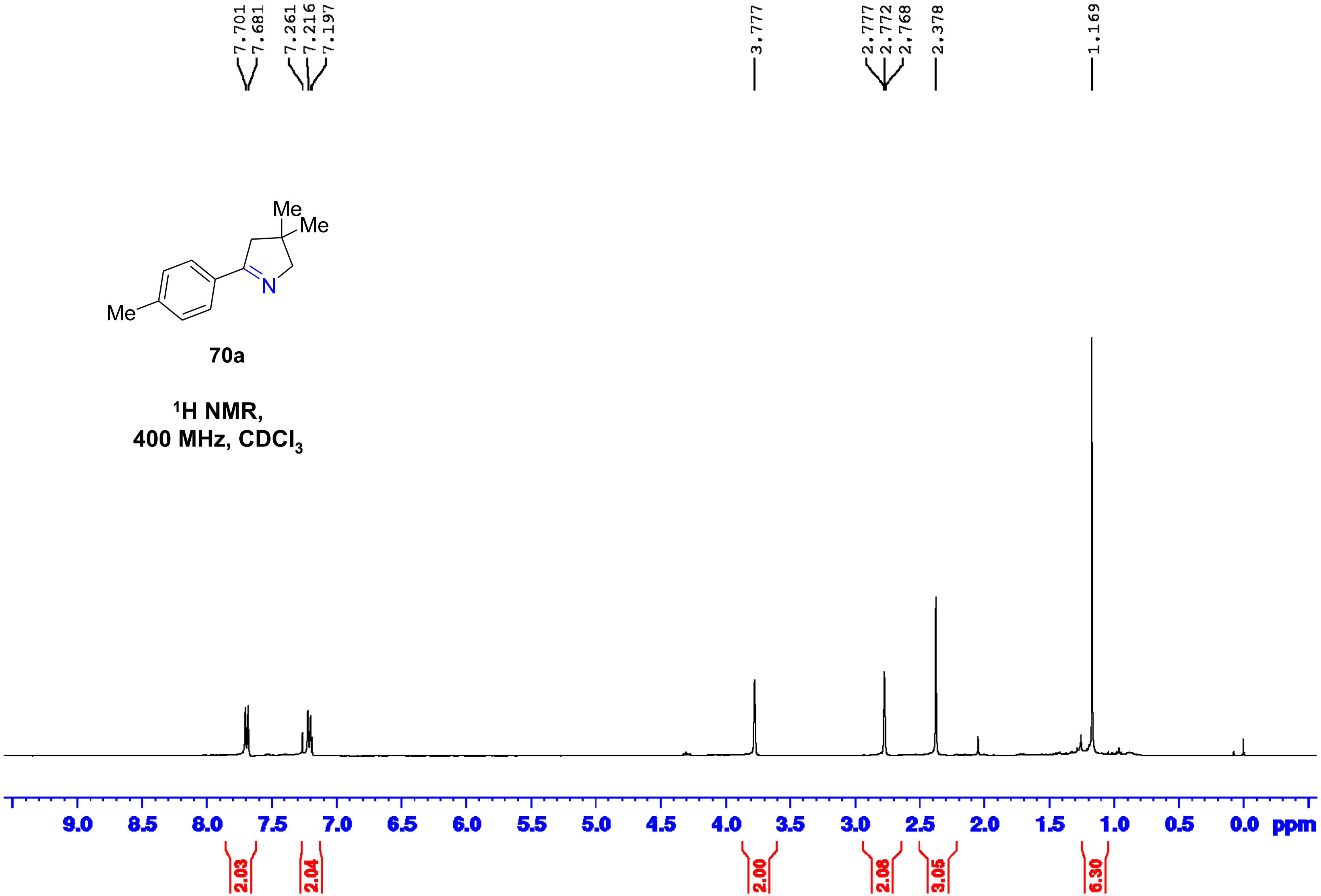

70a

^{1}H NMR,
400 MHz, $CDCl_3$

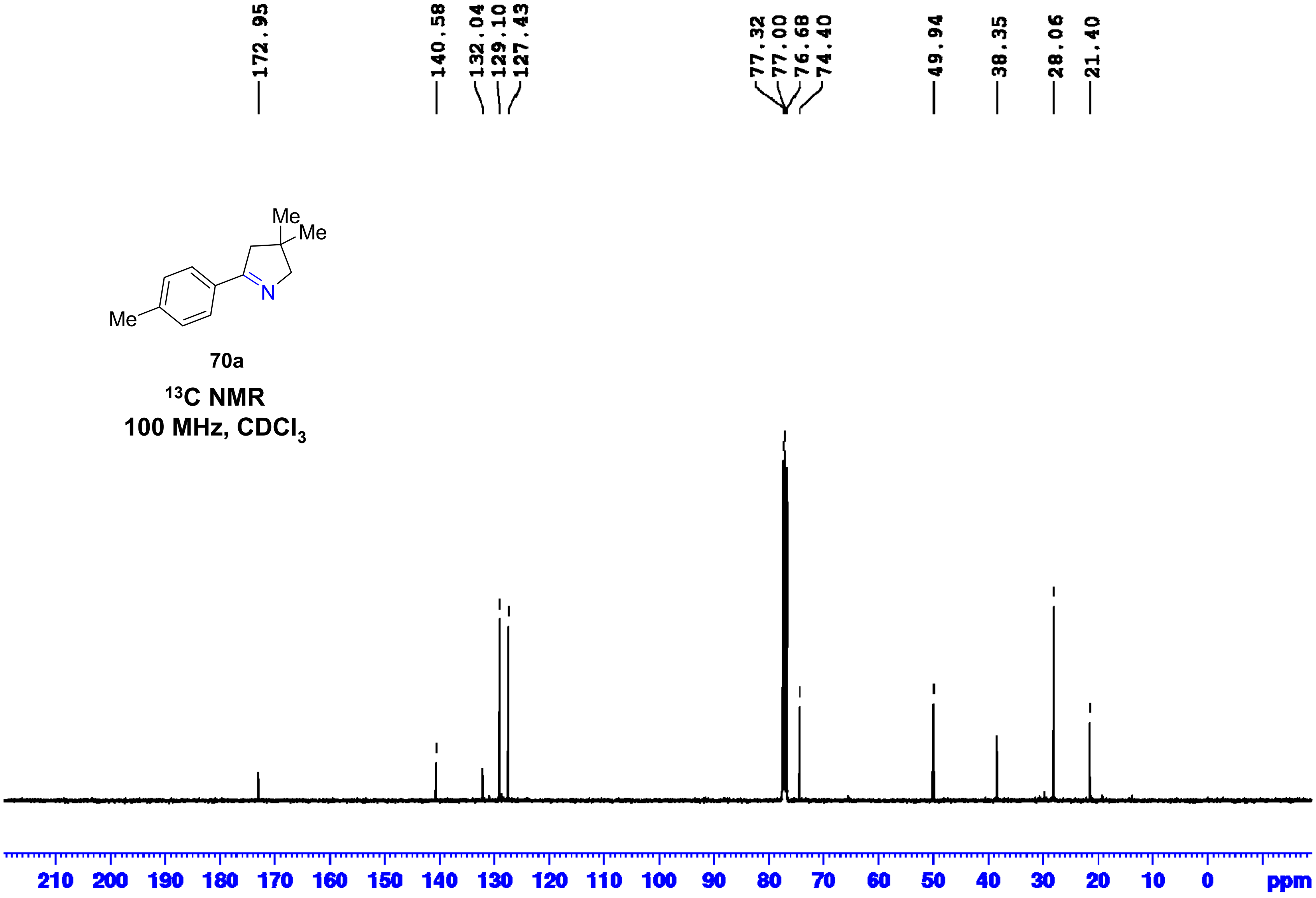

172.95
140.58
132.04
129.10
127.43
77.32
77.00
76.68
74.40
49.94
38.35
28.06
21.40
Me
Me
N
Me
70a
^{13}C NMR
100 MHz, $CDCl_3$
210 200 190 180 170 160 150 140 130 120 110 100 90 80 70 60 50 40 30 20 10 0 ppm

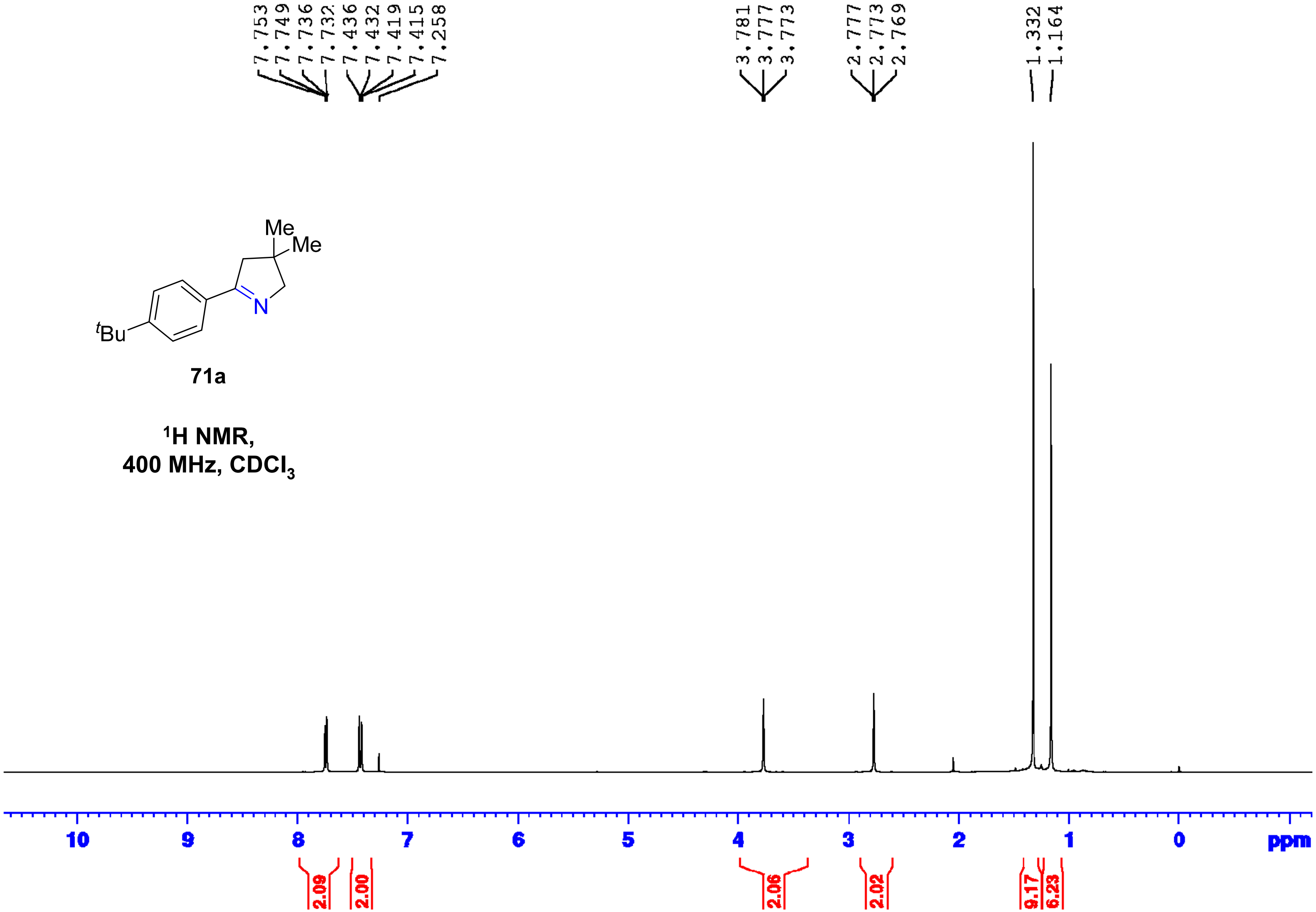

71a

^{1}H NMR,
400 MHz, CDCl$_3$

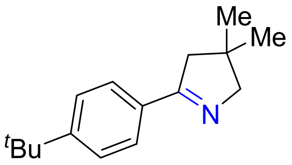

71a

^{13}C NMR
100 MHz, $CDCl_3$

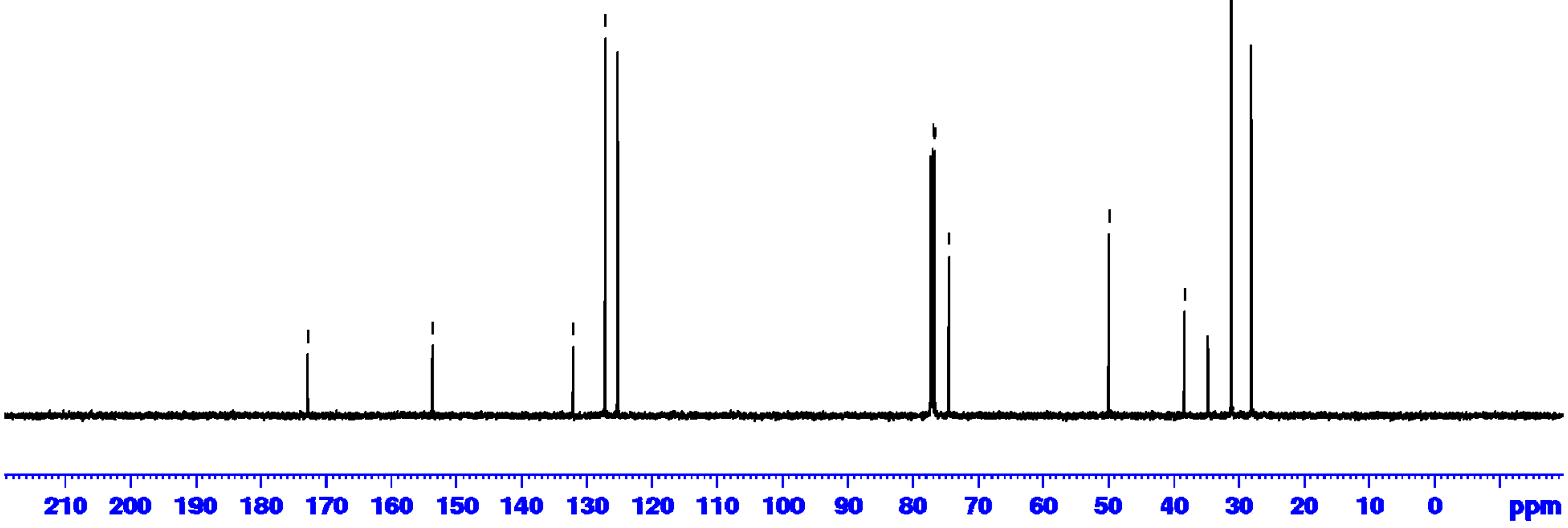

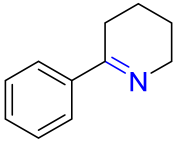

72a

^{1}H NMR,
400 MHz, $CDCl_3$

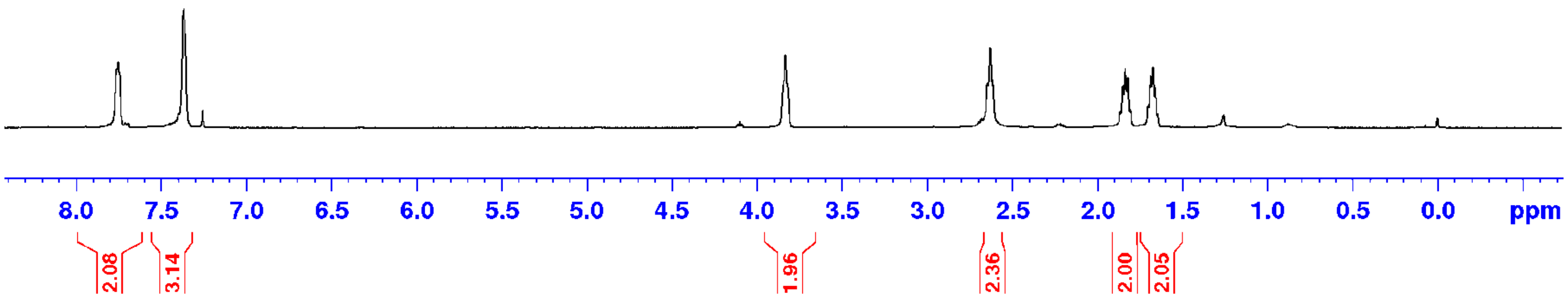

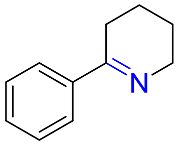

72a

^{13}C NMR
100 MHz, $CDCl_3$

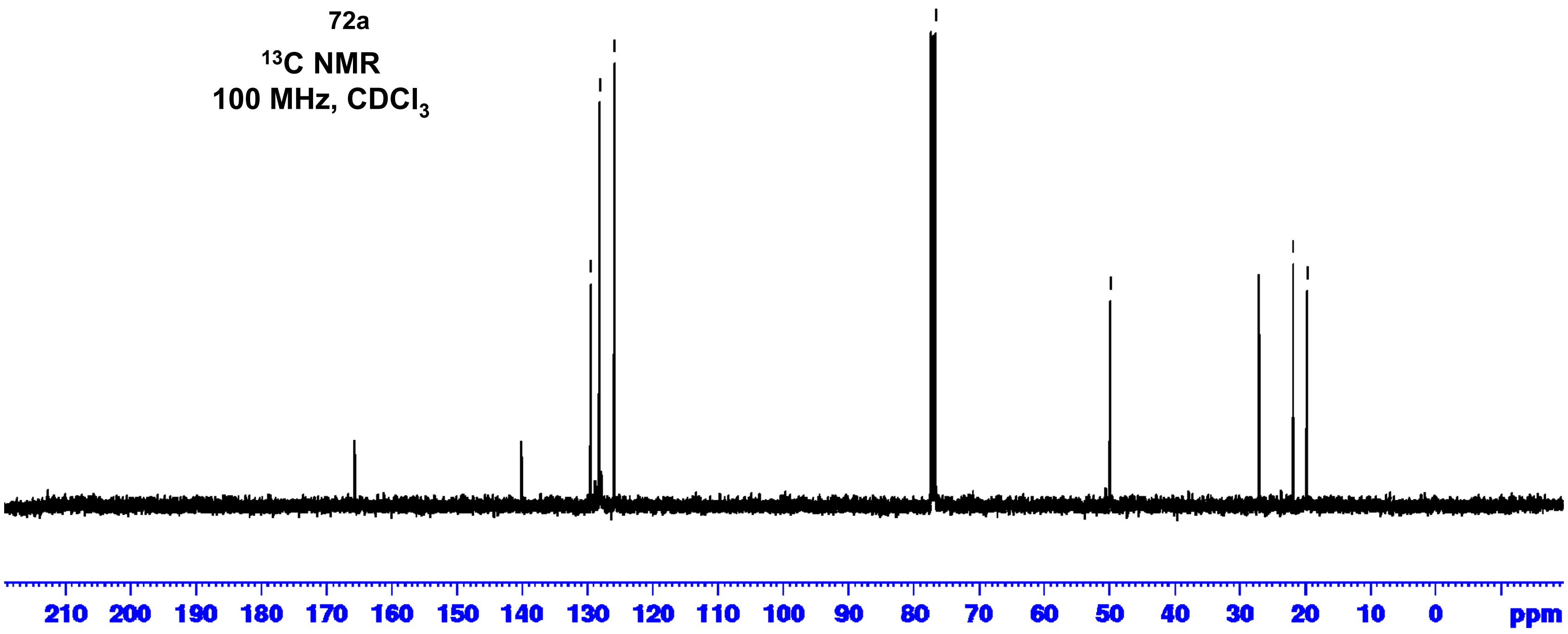

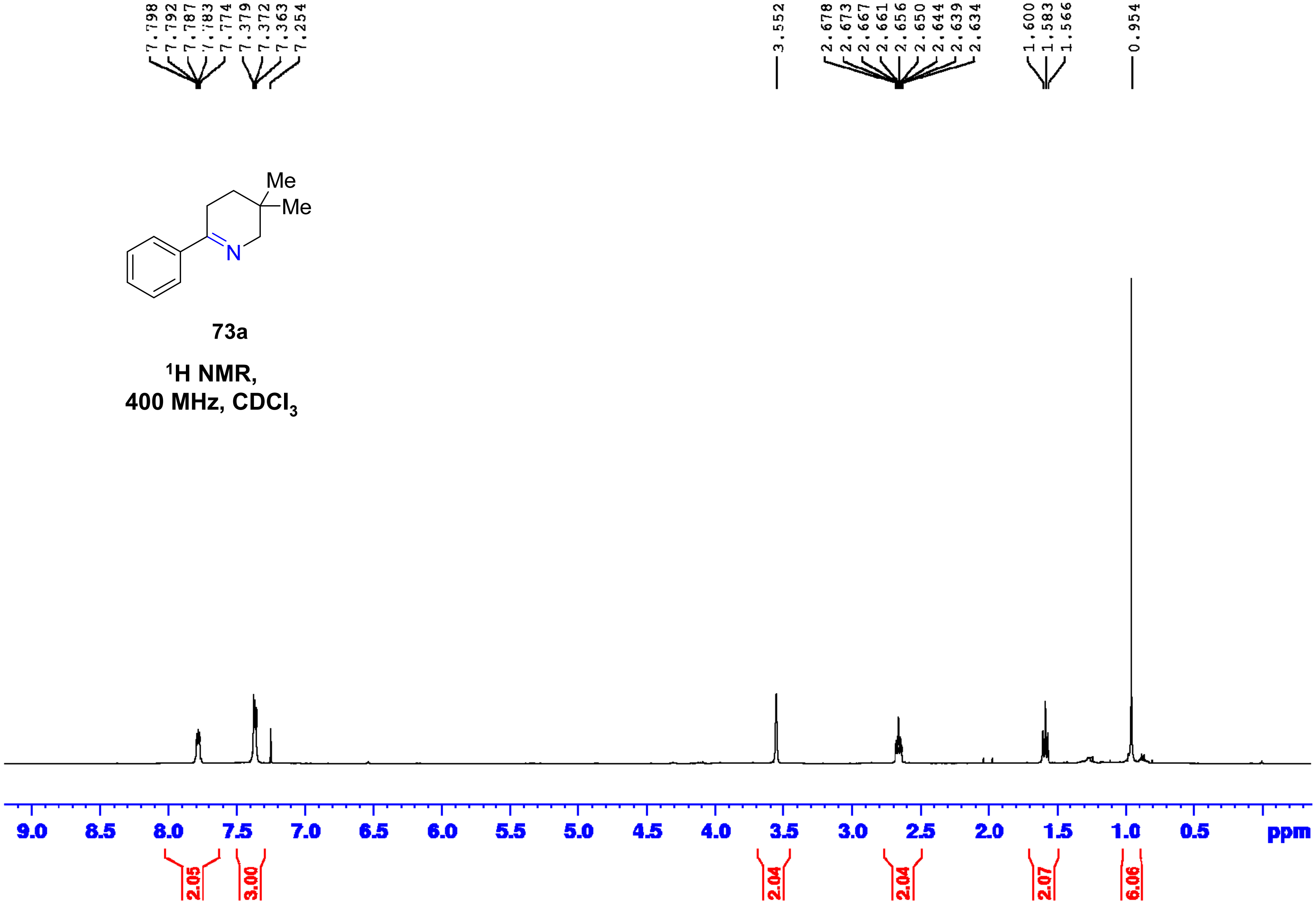

73a

^{1}H NMR,
400 MHz, $CDCl_3$

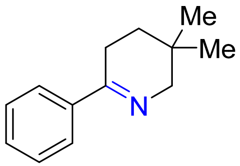

73a

^{13}C NMR
100 MHz, $CDCl_3$

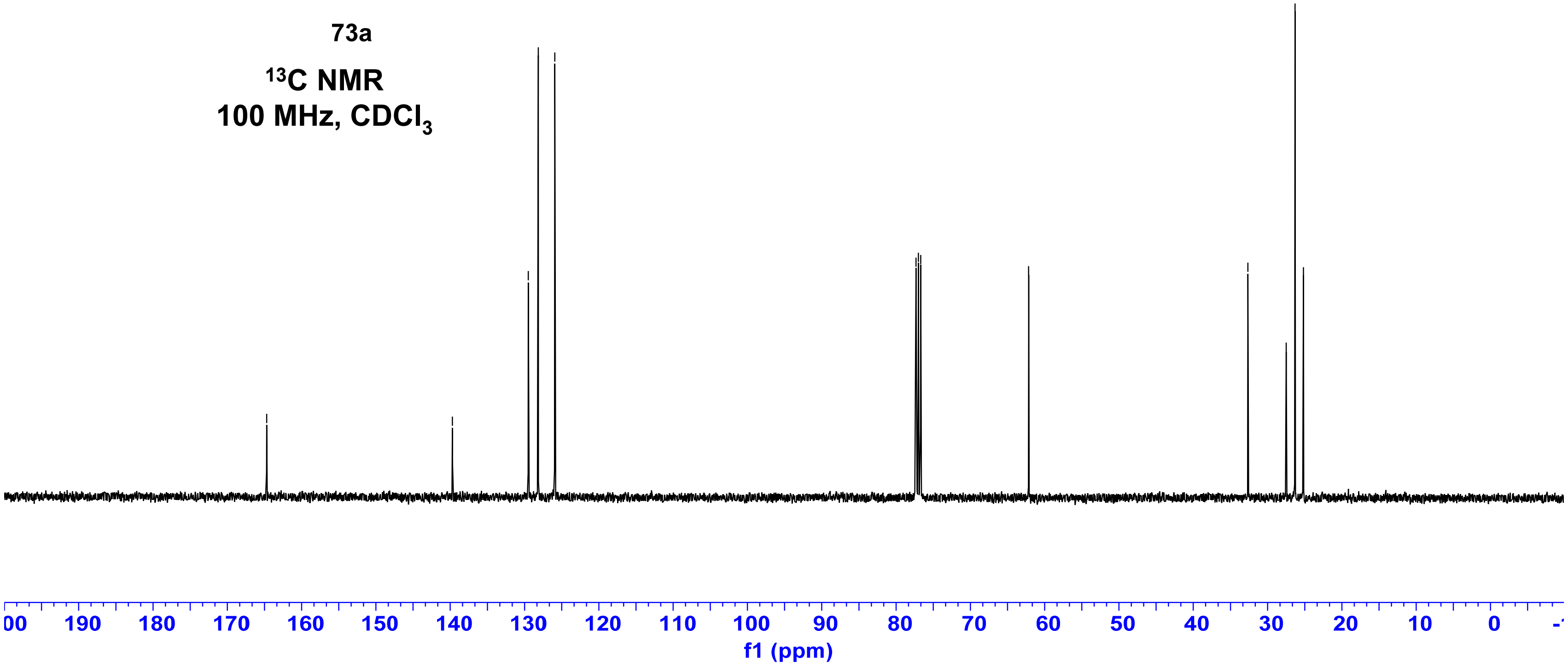

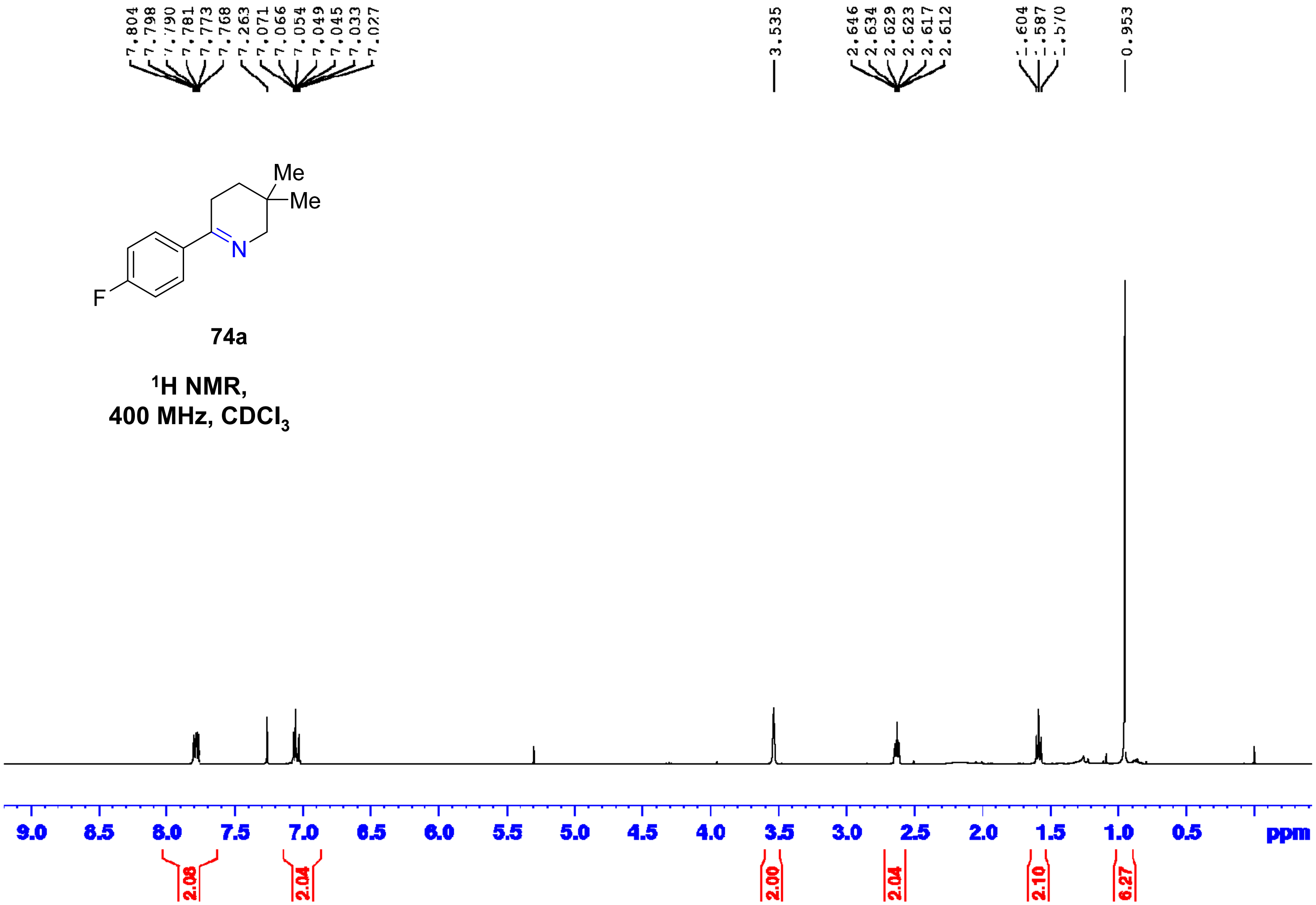

7.804
7.798
7.790
7.781
7.773
7.768
7.263
7.071
7.066
7.054
7.049
7.045
7.033
7.027
3.535
2.646
2.634
2.629
2.623
2.617
2.612
0.953
Me
Me
N
F
74a
¹H NMR,
400 MHz, CDCl3
9.0
8.5
8.0
7.5
7.0
6.5
6.0
5.5
5.0
4.5
4.0
3.5
3.0
2.5
2.0
1.5
1.0
0.5
ppm
2.08
2.04
2.00
2.04
2.10
6.27

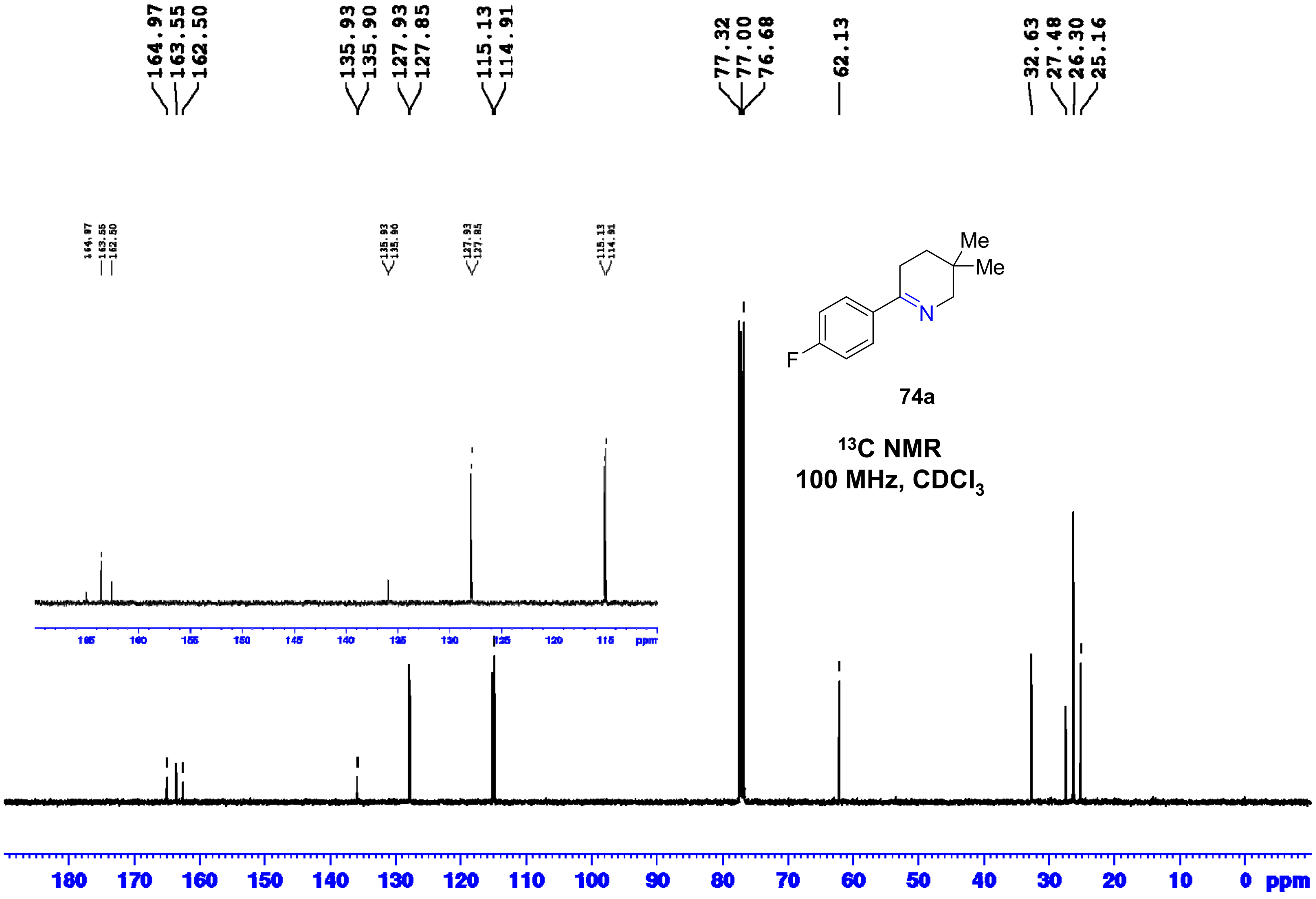
164.97
163.55
162.50
135.93
135.90
127.93
127.85
115.13
114.91
77.32
77.00
76.68
62.13
32.63
27.48
26.30
25.16
Me
Me
N
F
74a
^{13}C NMR
100 MHz, $CDCl_3$
180 170 160 150 140 130 120 110 100 90 80 70 60 50 40 30 20 10 0 ppm

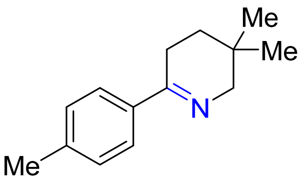

75a

^{1}H NMR,
400 MHz, $CDCl_3$

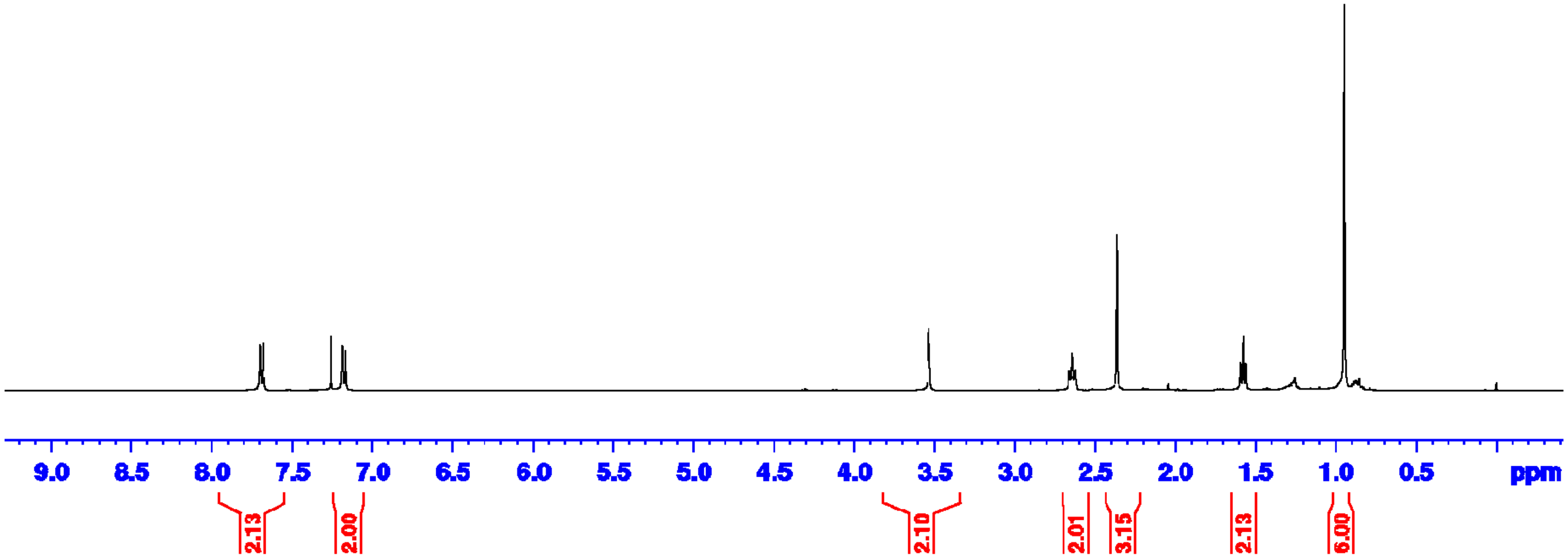

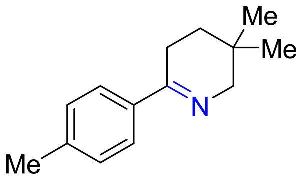

75a

^{13}C NMR
100 MHz, $CDCl_3$

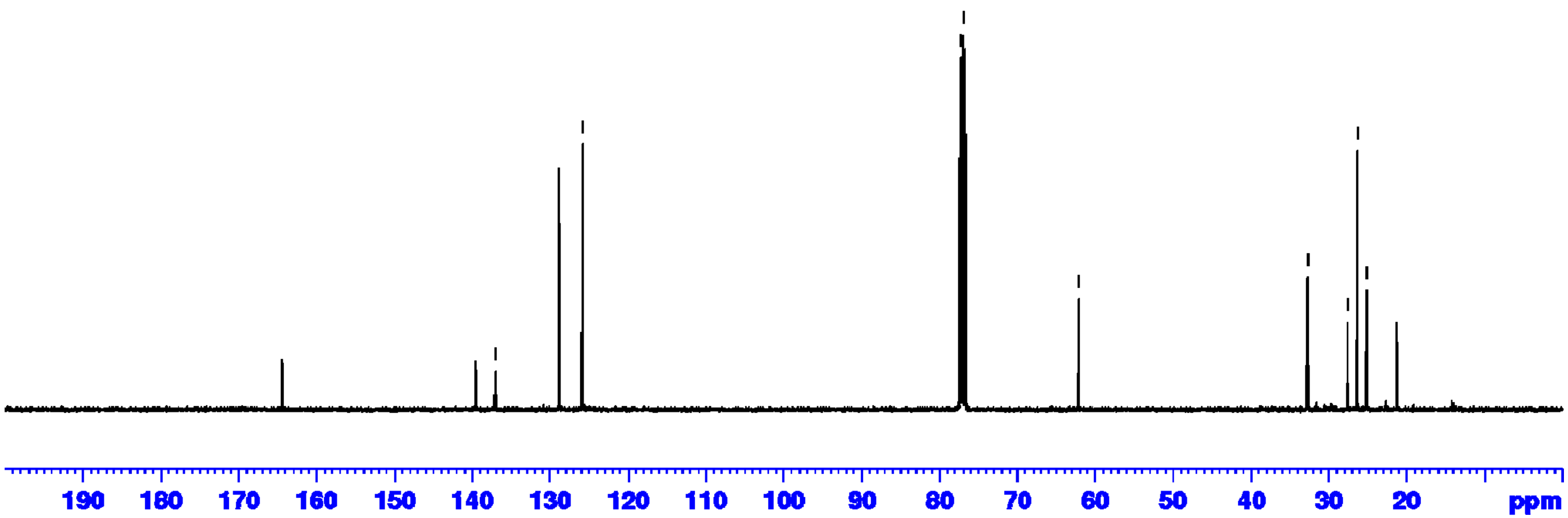

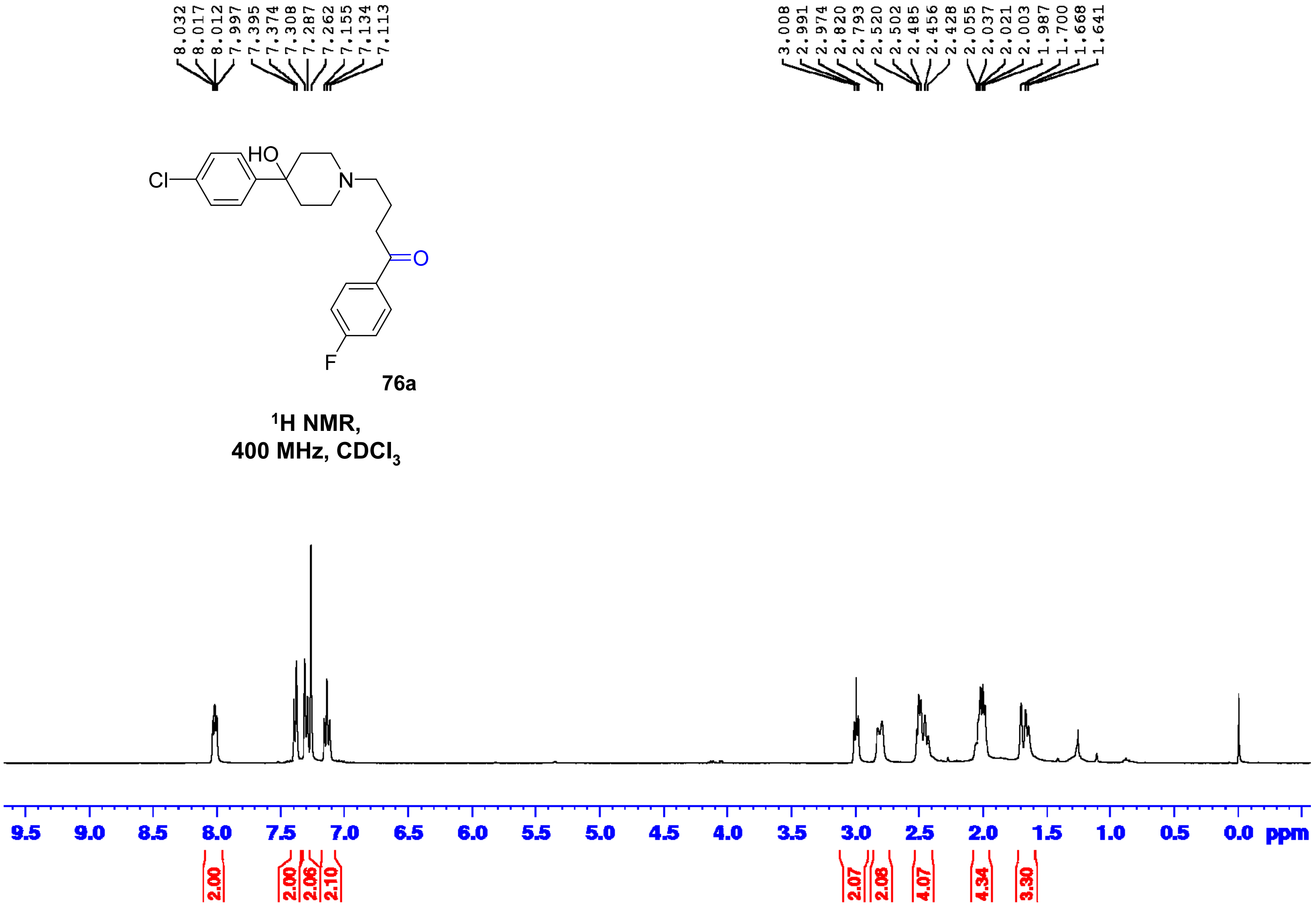
8.032
8.017
8.012
7.997
7.395
7.374
7.308
7.287
7.262
7.155
7.134
7.113
3.008
2.991
2.974
2.820
2.793
2.520
2.502
2.485
2.456
2.428
2.055
2.037
2.021
2.003
1.987
1.700
1.668
1.641
HO
Cl
N
O
F
76a
^{1}H NMR,
400 MHz, $CDCl_3$
9.5 9.0 8.5 8.0 7.5 7.0 6.5 6.0 5.5 5.0 4.5 4.0 3.5 3.0 2.5 2.0 1.5 1.0 0.5 0.0 ppm
2.00
2.00
2.06
2.10
2.07
2.08
4.07
4.34
3.30

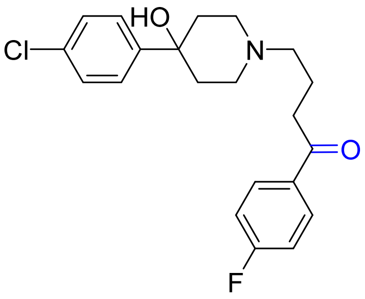

76a

^{13}C NMR
100 MHz, $CDCl_3$

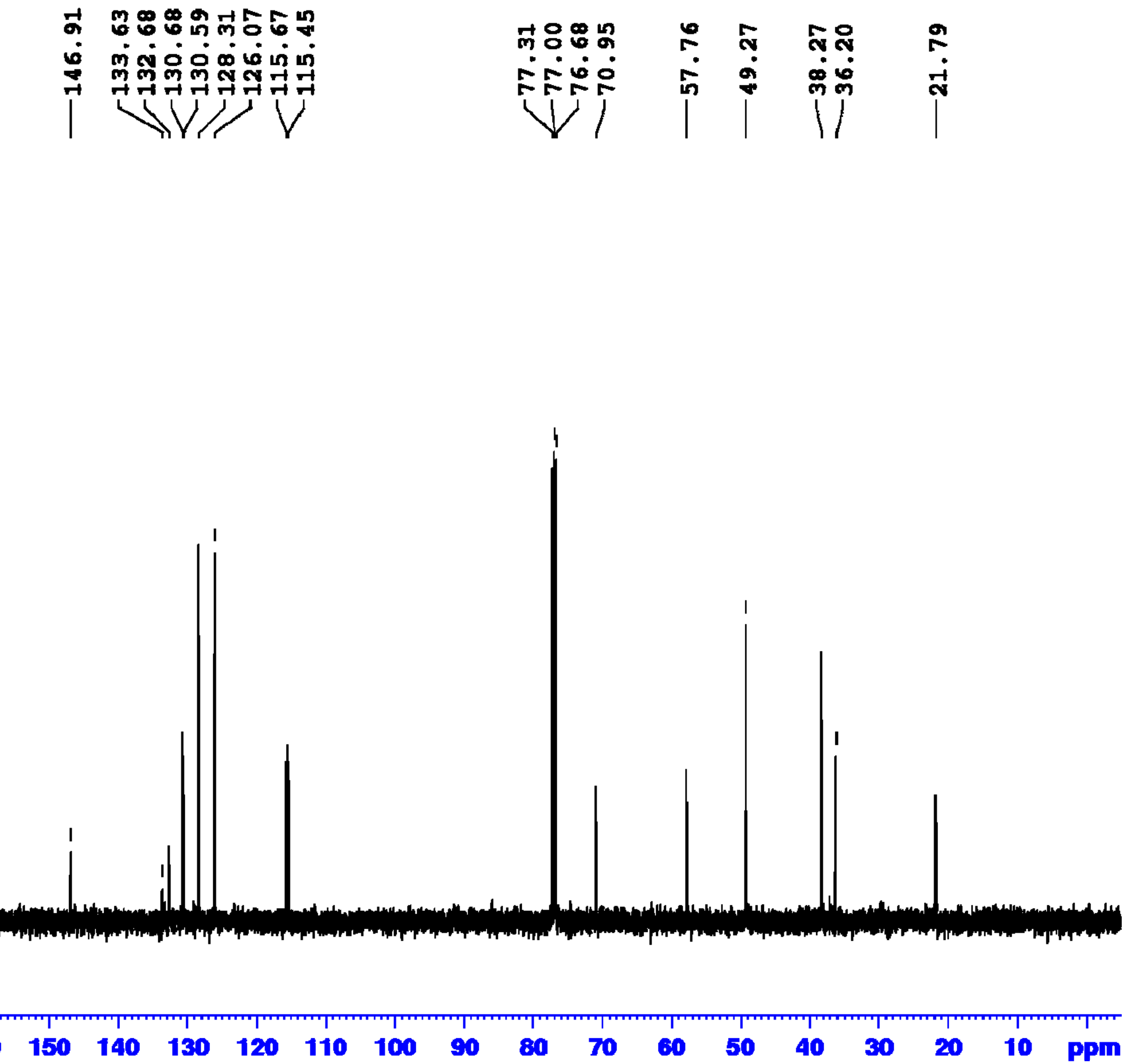

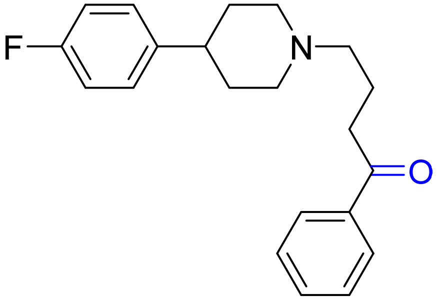

77a

^{1}H NMR,
400 MHz, $CDCl_3$

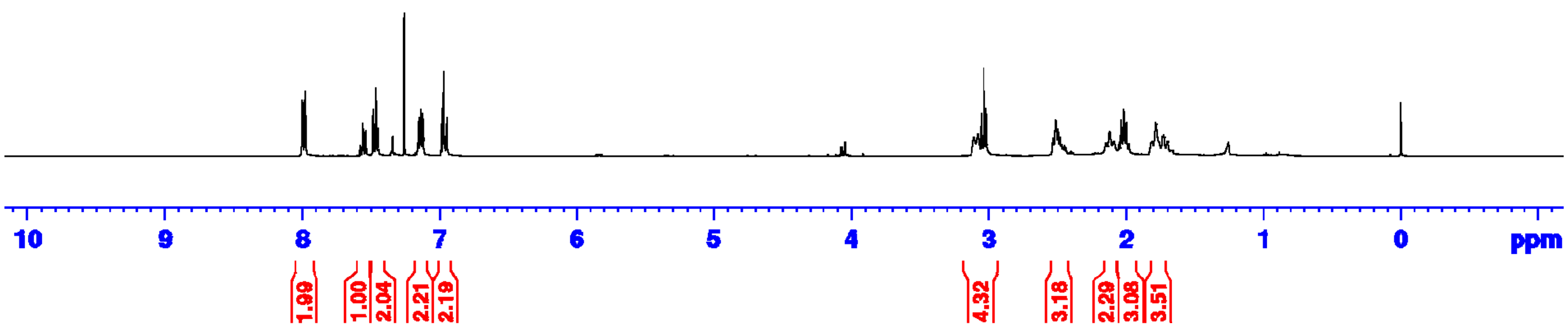

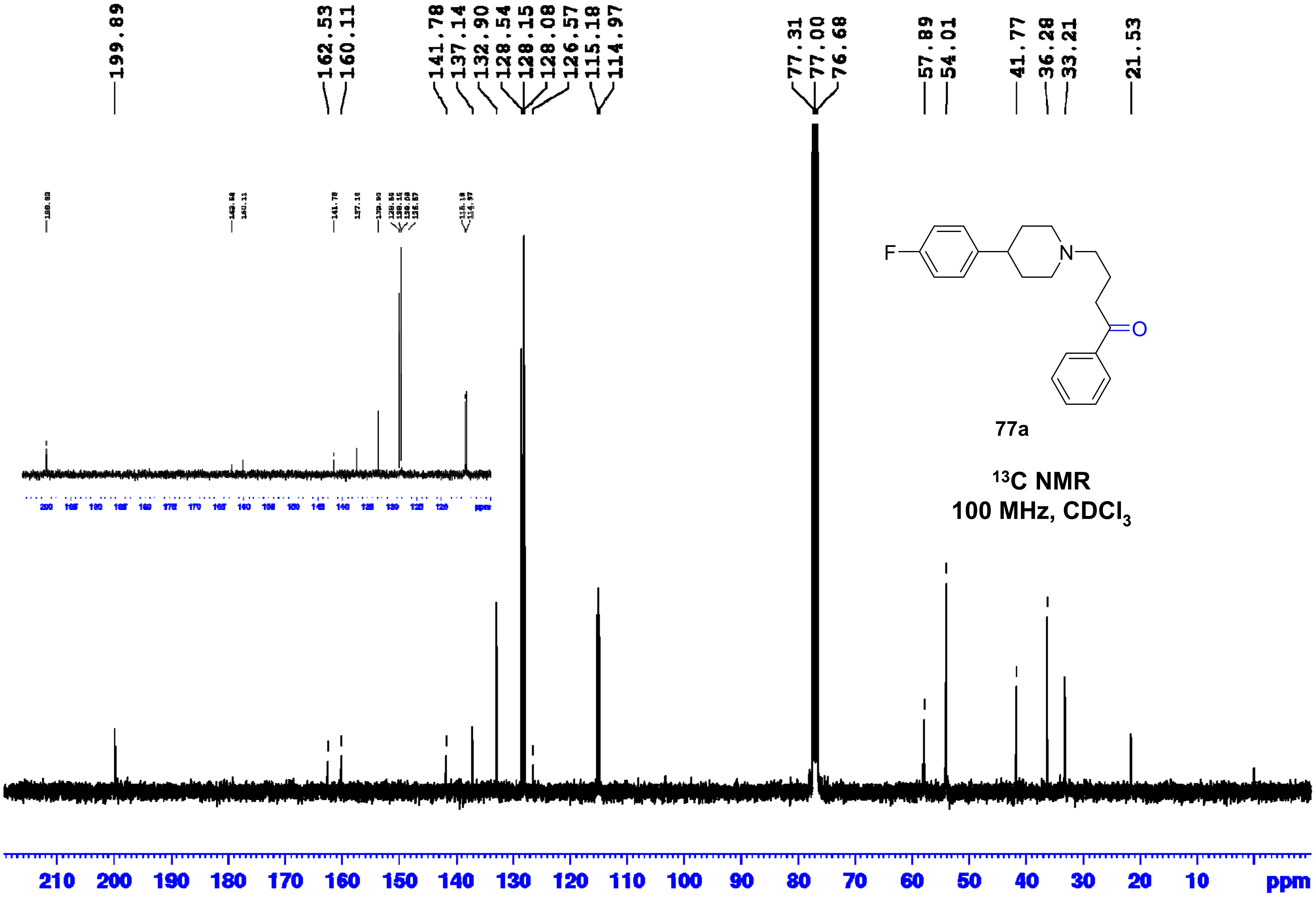
199.89
162.53
160.11
141.78
137.14
132.90
128.54
128.15
128.08
126.57
115.18
114.97
77.31
77.00
76.68
57.89
54.01
41.77
36.28
33.21
21.53
F
N
O
77a
^{13}C NMR
100 MHz, $CDCl_3$
210 200 190 180 170 160 150 140 130 120 110 100 90 80 70 60 50 40 30 20 10 ppm

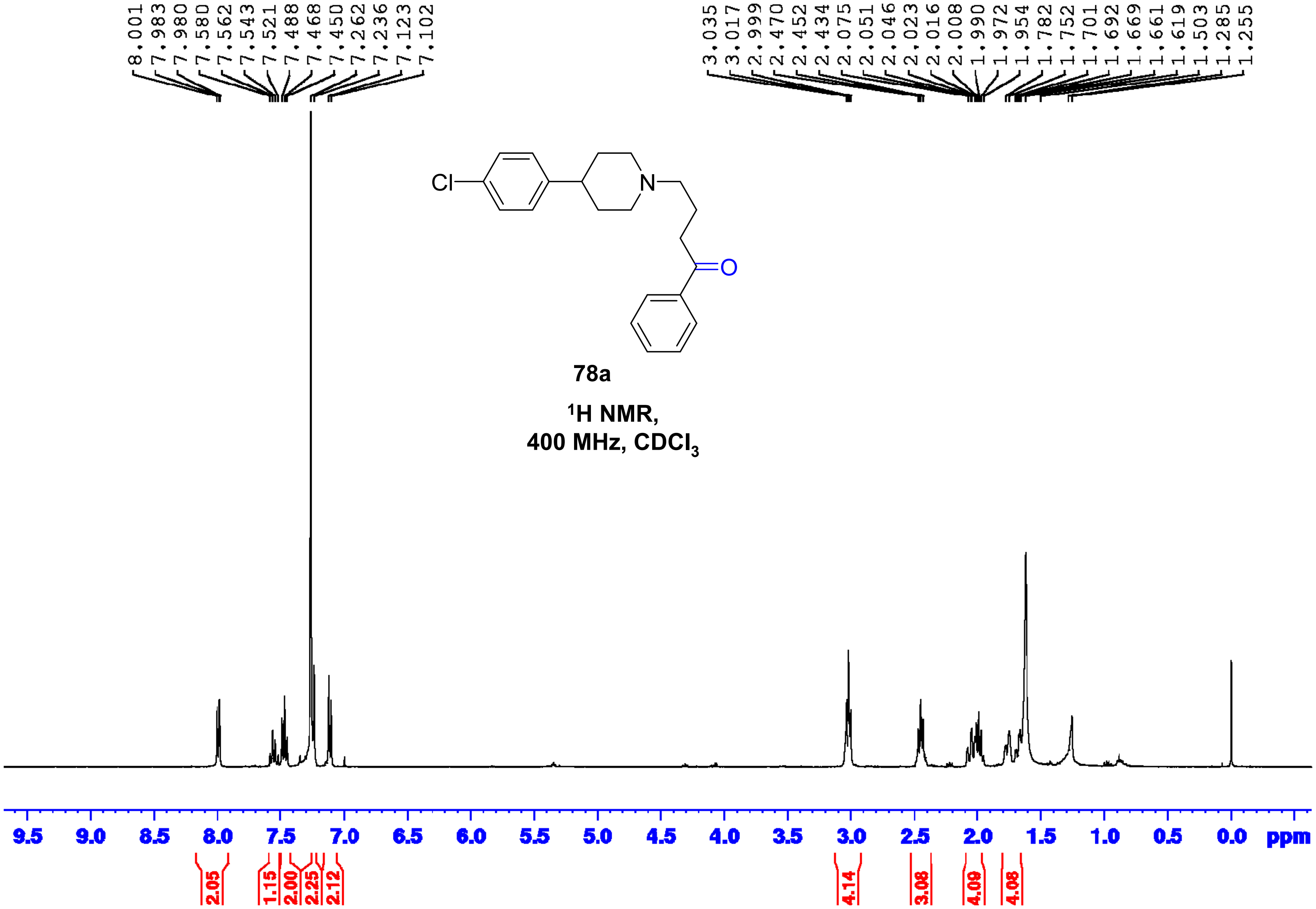
8.001
7.983
7.980
7.580
7.562
7.543
7.521
7.488
7.468
7.450
7.262
7.236
7.123
7.102
3.035
3.017
2.999
2.470
2.452
2.434
2.075
2.051
2.046
2.023
2.016
2.008
1.990
1.972
1.954
1.782
1.752
1.701
1.692
1.669
1.661
1.619
1.503
1.285
1.255
Cl
N
O
78a
1H NMR,
400 MHz, CDCl3
9.5 9.0 8.5 8.0 7.5 7.0 6.5 6.0 5.5 5.0 4.5 4.0 3.5 3.0 2.5 2.0 1.5 1.0 0.5 0.0 ppm
2.05
1.15
2.00
2.25
2.12
4.14
3.08
4.09
4.08

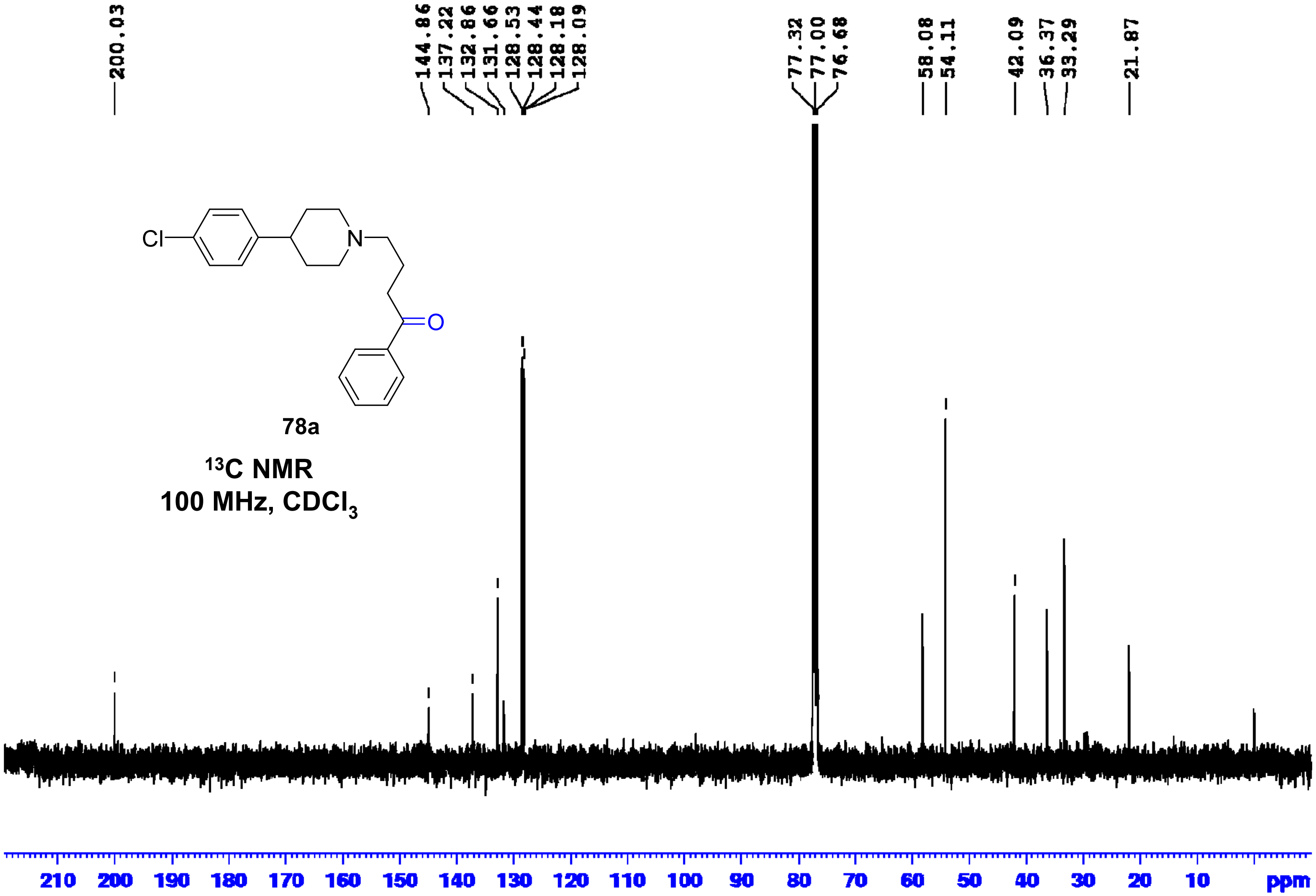
200.03
144.86
137.22
132.86
131.66
128.53
128.44
128.18
128.09
77.32
77.00
76.68
58.08
54.11
42.09
36.37
33.29
21.87
Cl
N
O
78a
^{13}C NMR
100 MHz, $CDCl_3$
210 200 190 180 170 160 150 140 130 120 110 100 90 80 70 60 50 40 30 20 10 ppm

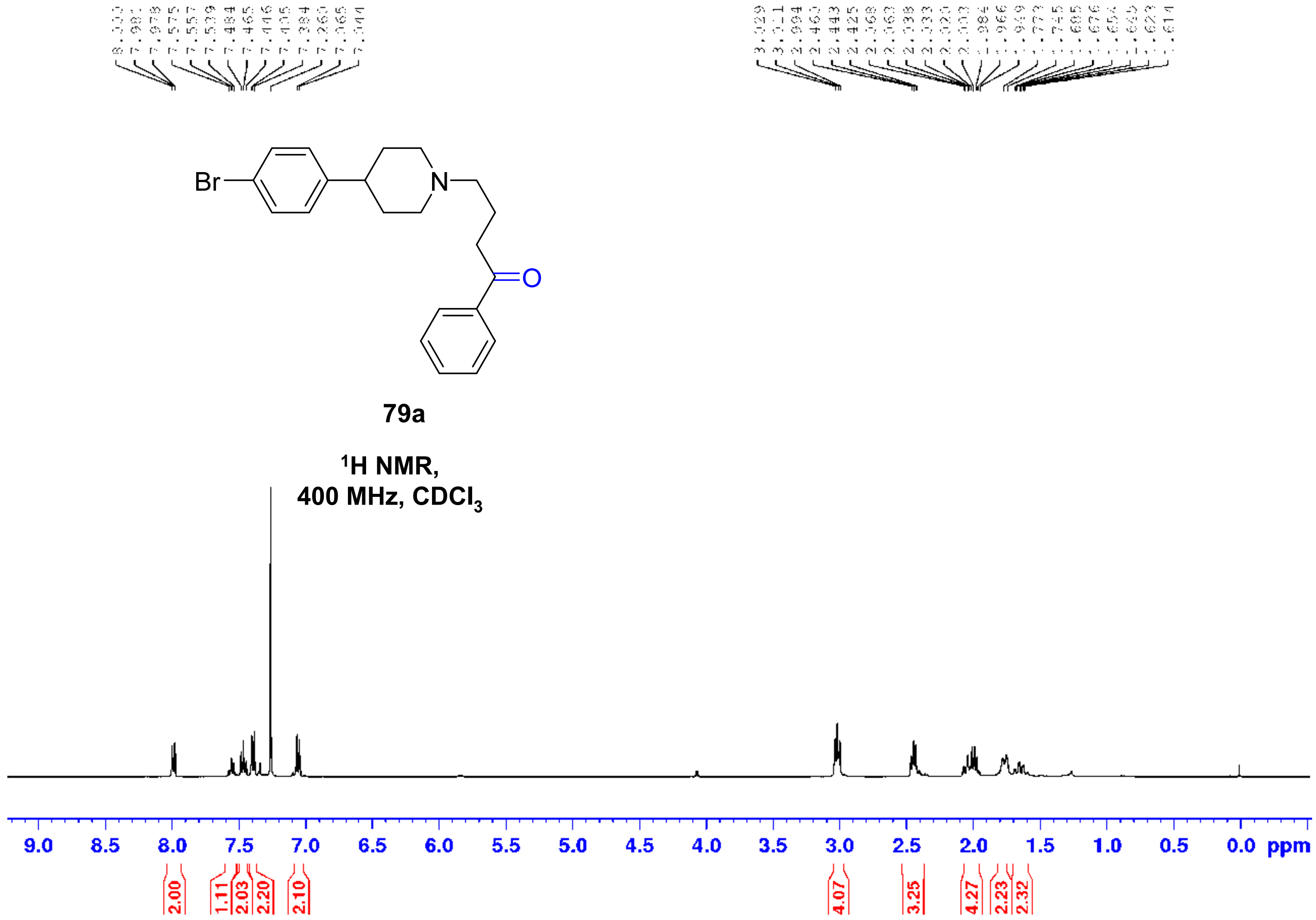
Br
N
O
79a
1H NMR,
400 MHz, CDCl3
9.0 8.5 8.0 7.5 7.0 6.5 6.0 5.5 5.0 4.5 4.0 3.5 3.0 2.5 2.0 1.5 1.0 0.5 0.0 ppm
2.00 1.11 2.03 2.20 2.10 4.07 3.25 4.27 2.23 2.32

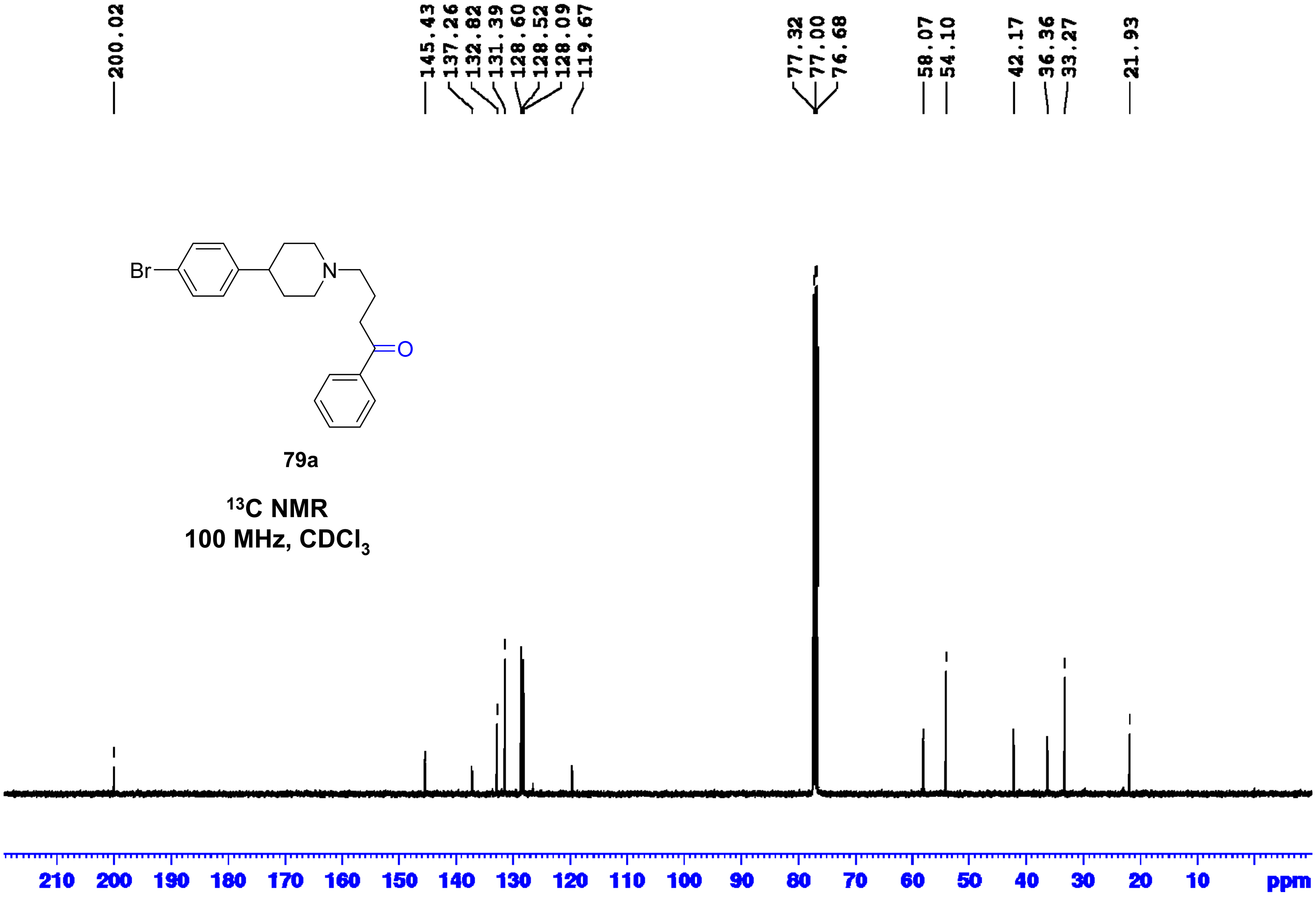
200.02
145.43
137.26
132.82
131.39
128.60
128.52
128.09
119.67
77.32
77.00
76.68
58.07
54.10
42.17
36.36
33.27
21.93
Br
N
O
79a
^{13}C NMR
100 MHz, $CDCl_3$
210 200 190 180 170 160 150 140 130 120 110 100 90 80 70 60 50 40 30 20 10 ppm

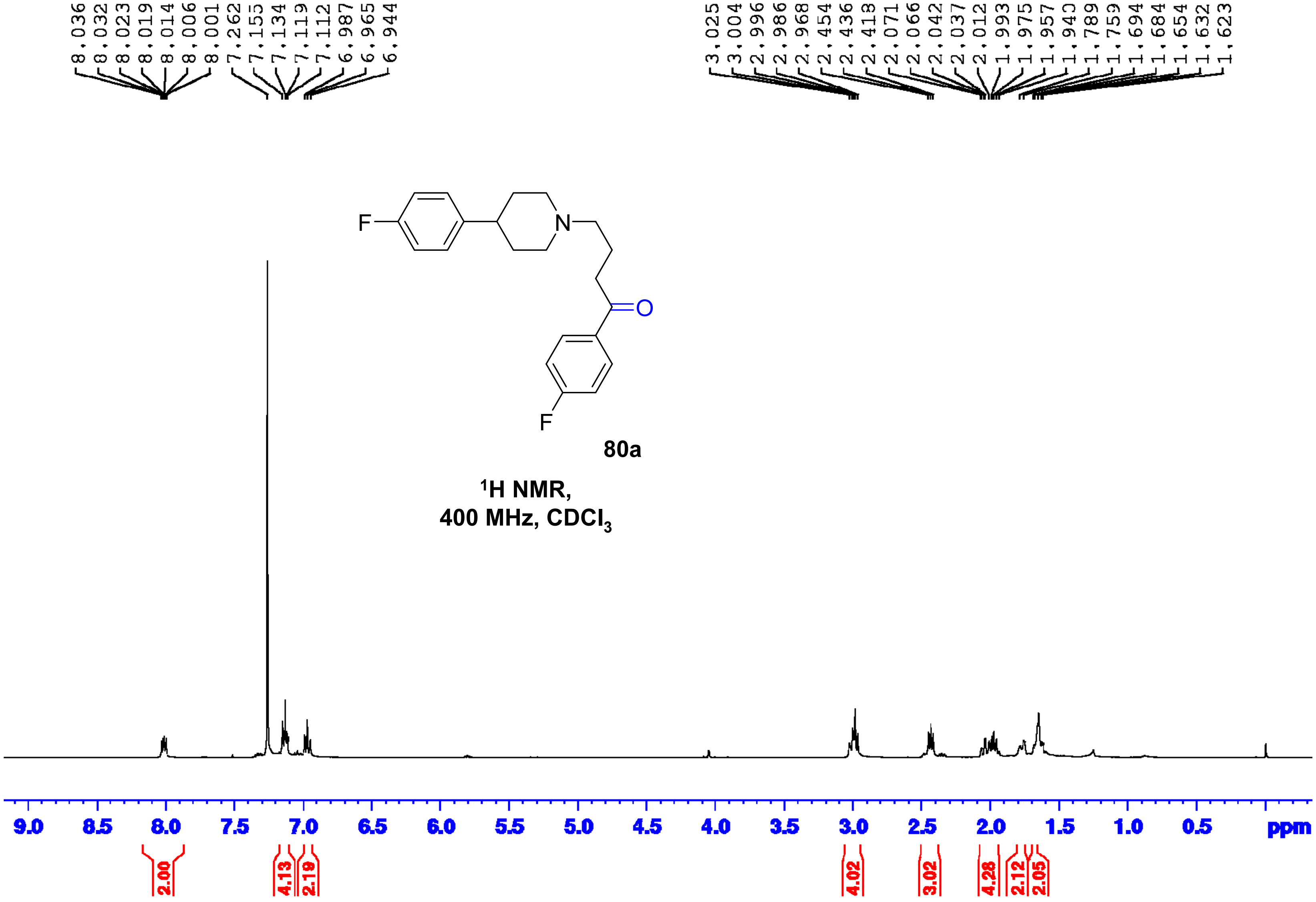

80a

^{1}H NMR,
400 MHz, $CDCl_3$

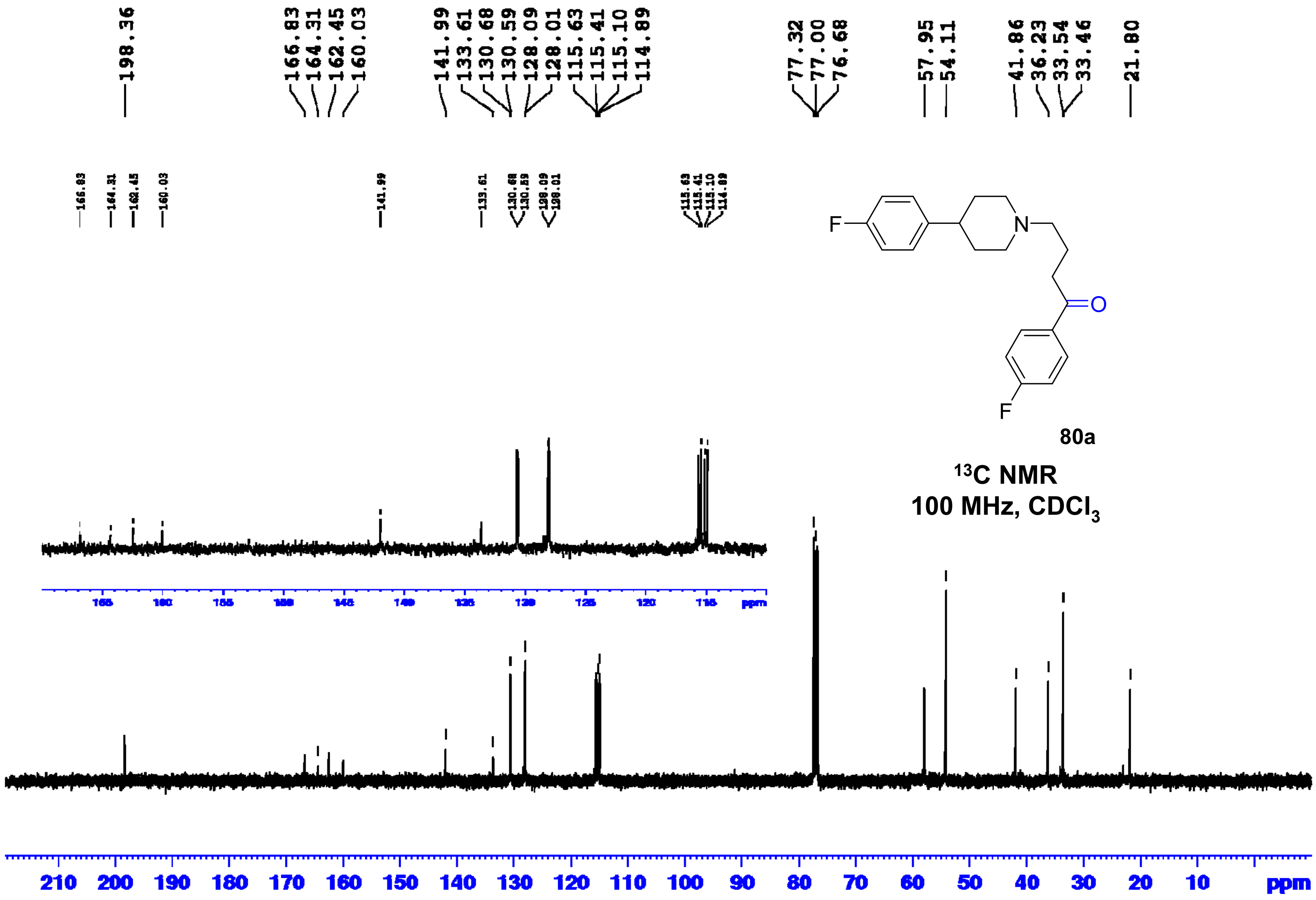
198.36
166.83
164.31
162.45
160.03
141.99
133.61
130.68
130.59
128.09
128.01
115.63
115.41
115.10
114.89
77.32
77.00
76.68
57.95
54.11
41.86
36.23
33.54
33.46
21.80
F
N
O
F
80a
13C NMR
100 MHz, CDCl3
ppm

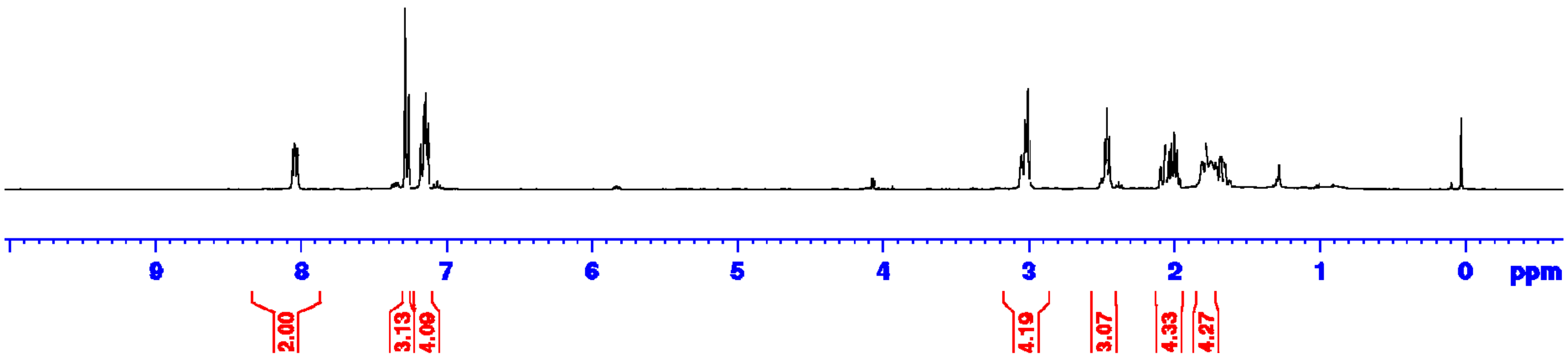

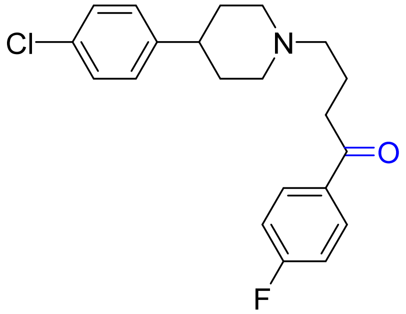

81a

^{1}H NMR,
400 MHz, $CDCl_3$

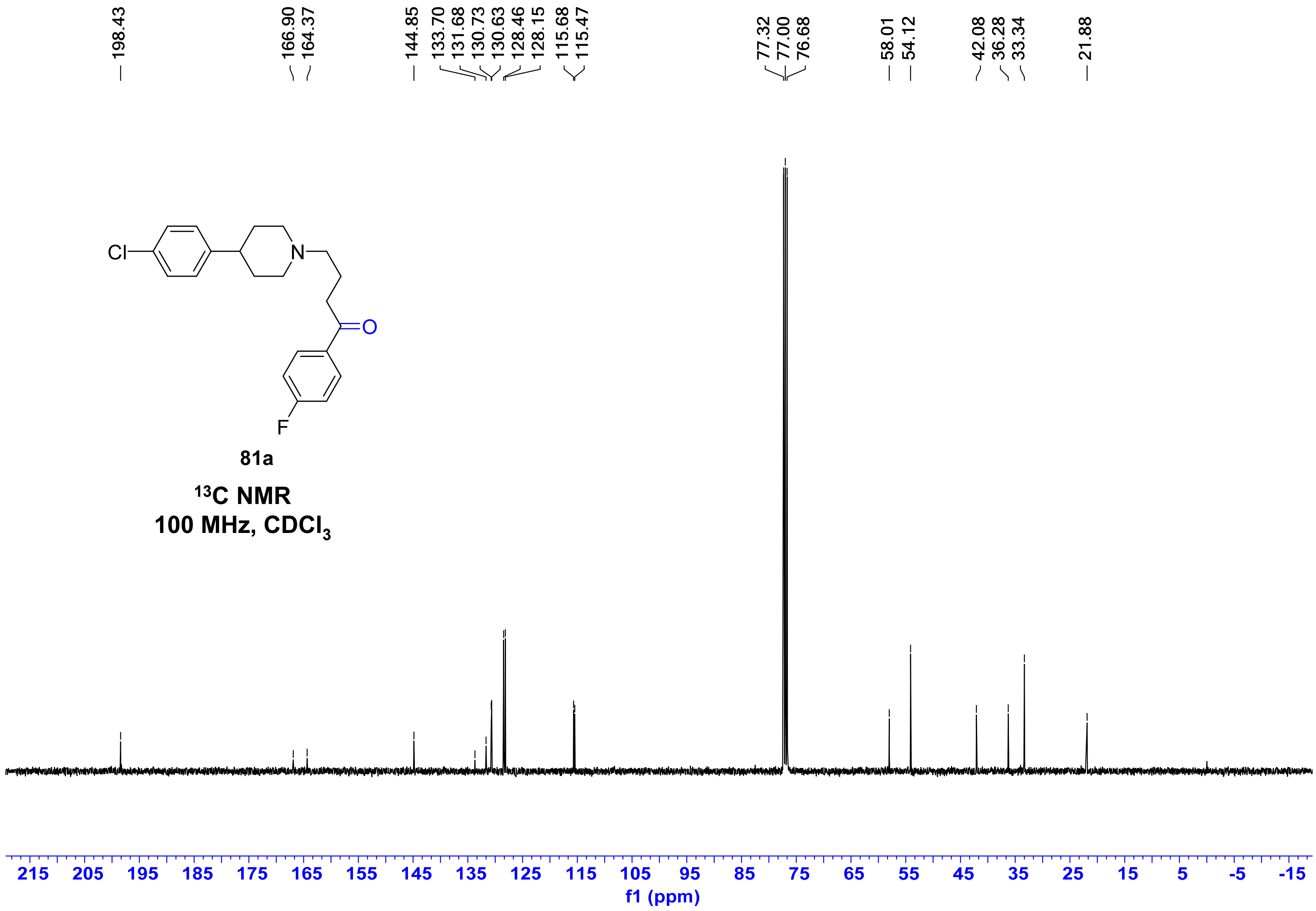
198.43
166.90
164.37
144.85
133.70
131.68
130.73
130.63
128.46
128.15
115.68
115.47
77.32
77.00
76.68
58.01
54.12
42.08
36.28
33.34
21.88
Cl
N
O
F
81a
13C NMR
100 MHz, CDCl3
215 205 195 185 175 165 155 145 135 125 115 105 95 85 75 65 55 45 35 25 15 5 -5 -15
f1 (ppm)

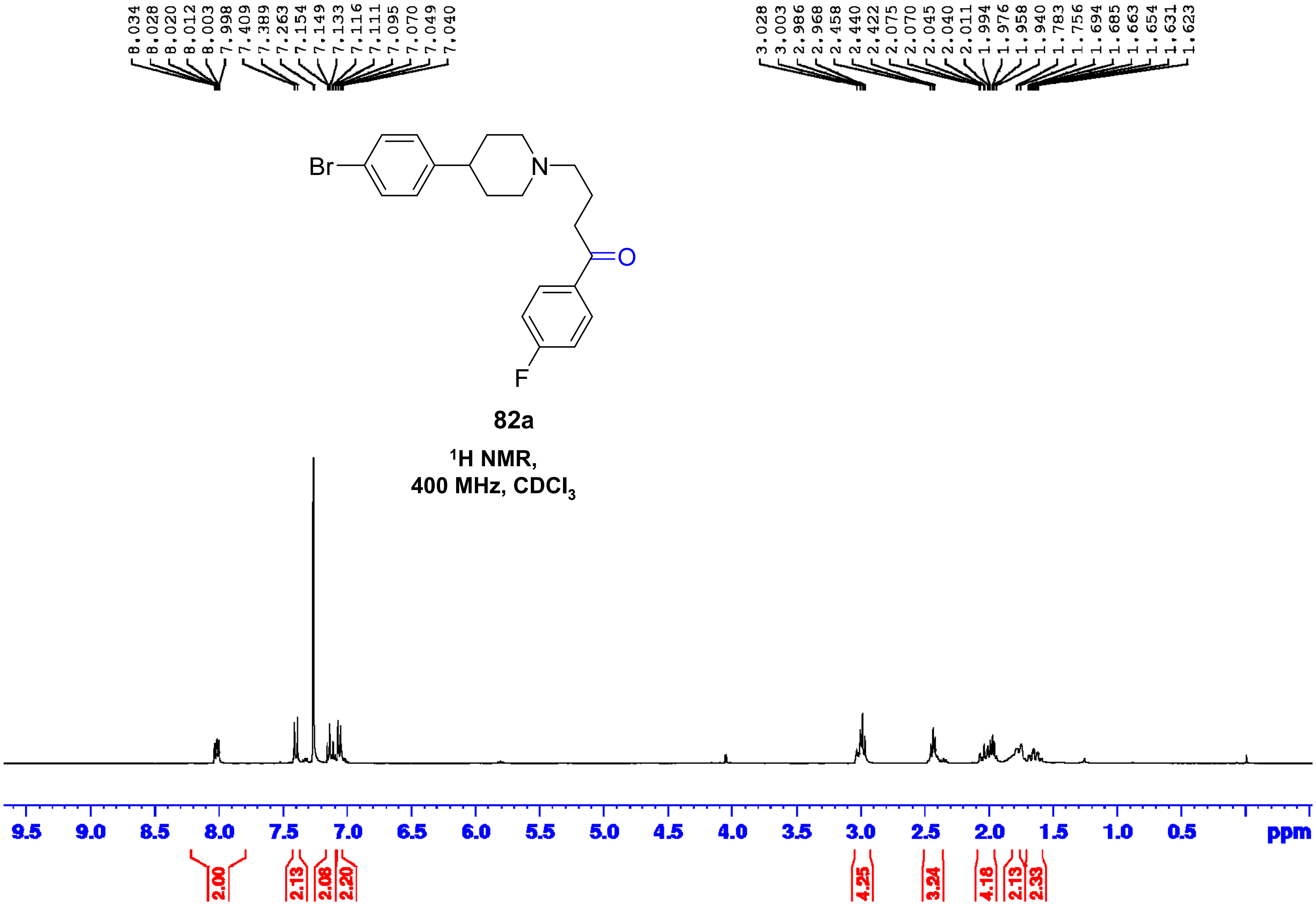
8.034
8.028
8.020
8.012
8.003
7.998
7.409
7.389
7.263
7.154
7.149
7.133
7.116
7.111
7.095
7.070
7.049
7.040
3.028
3.003
2.986
2.968
2.458
2.440
2.422
2.075
2.070
2.045
2.040
2.011
1.994
1.976
1.958
1.940
1.783
1.756
1.694
1.685
1.663
1.654
1.631
1.623
Br
N
O
F
82a
^{1}H NMR,
400 MHz, $CDCl_3$
9.5
9.0
8.5
8.0
7.5
7.0
6.5
6.0
5.5
5.0
4.5
4.0
3.5
3.0
2.5
2.0
1.5
1.0
0.5
ppm
2.00
2.13
2.08
2.20
4.25
3.24
4.18
2.13
2.33

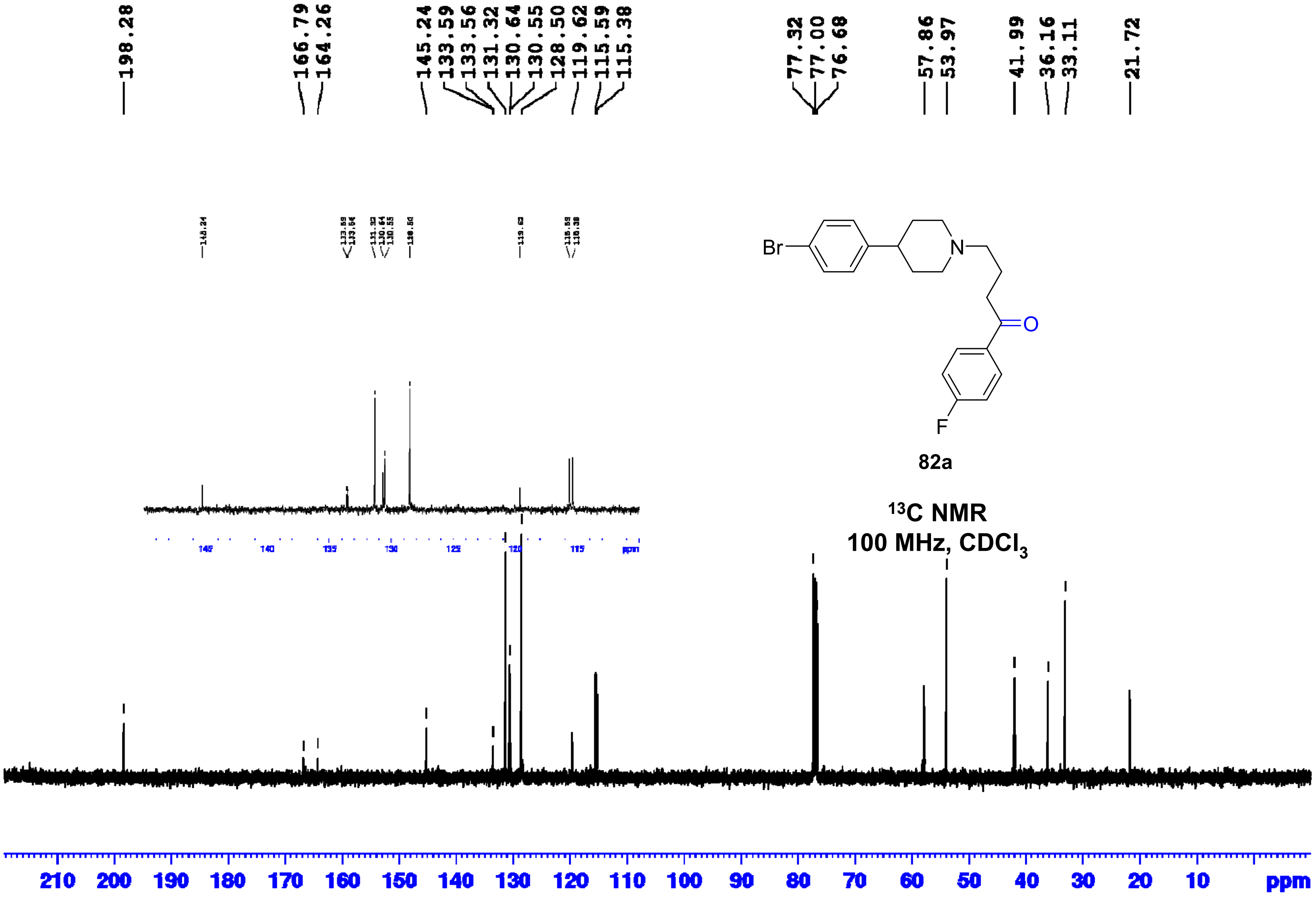
198.28
166.79
164.26
145.24
133.59
133.56
131.32
130.64
130.55
128.50
119.62
115.59
115.38
77.32
77.00
76.68
57.86
53.97
41.99
36.16
33.11
21.72
Br
N
O
F
82a
^{13}C NMR
100 MHz, CDCl$_3$
210
200
190
180
170
160
150
140
130
120
110
100
90
80
70
60
50
40
30
20
10
ppm

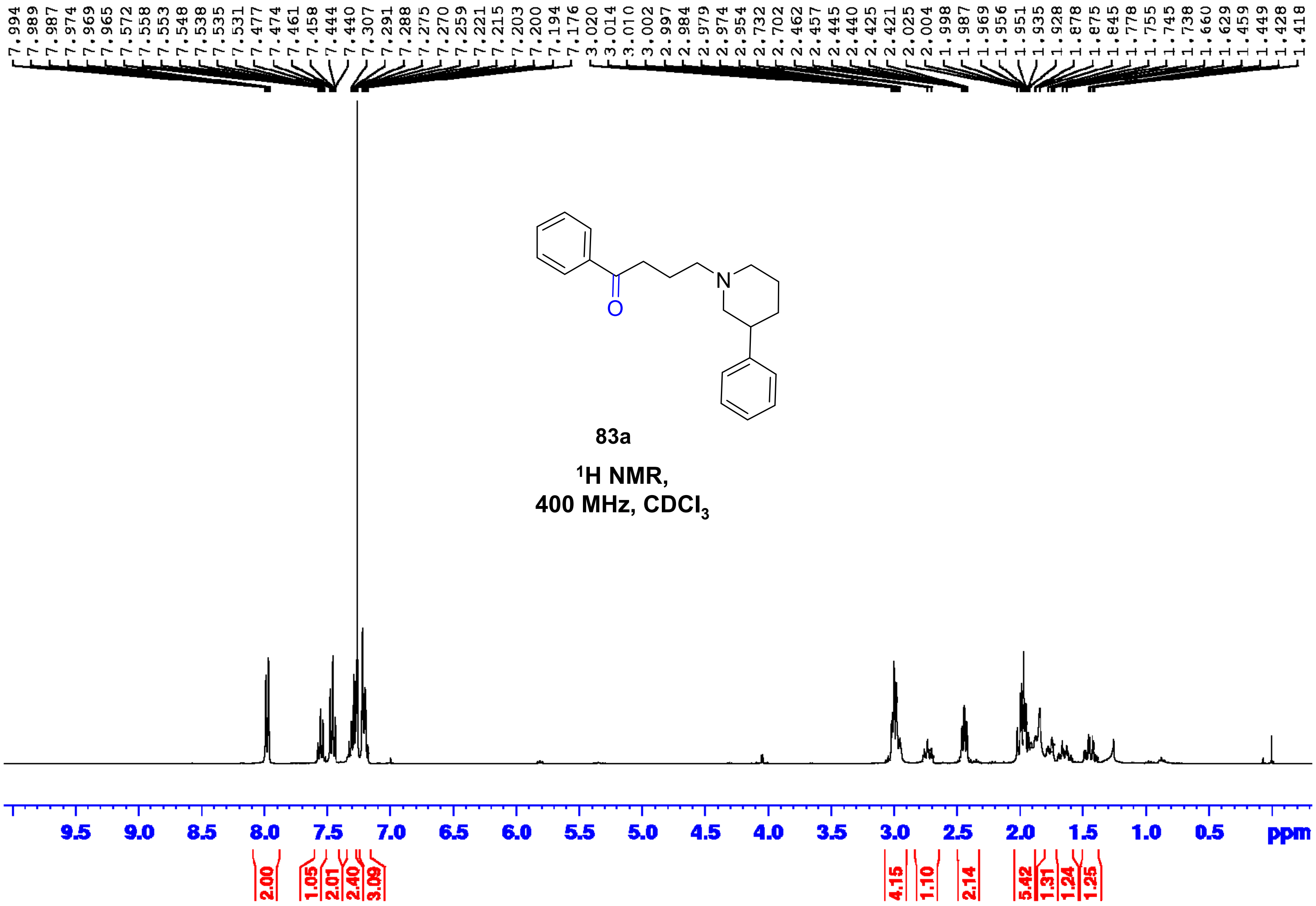
83a
1H NMR,
400 MHz, CDCl3
ppm

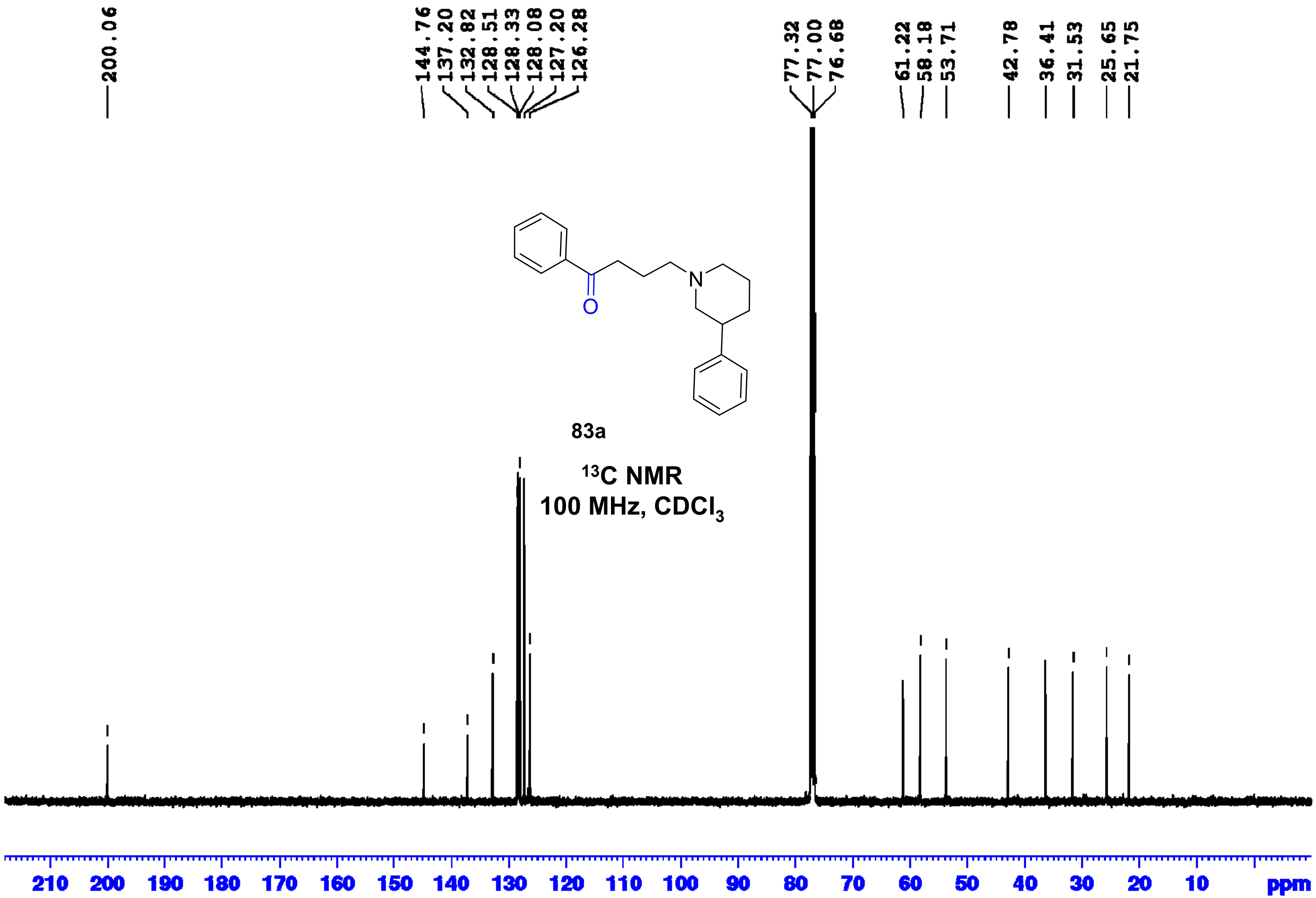
200.06
144.76
137.20
132.82
128.51
128.33
128.08
127.20
126.28
77.32
77.00
76.68
61.22
58.18
53.71
42.78
36.41
31.53
25.65
21.75
N
O
83a
^{13}C NMR
100 MHz, $CDCl_3$
210 200 190 180 170 160 150 140 130 120 110 100 90 80 70 60 50 40 30 20 10 ppm

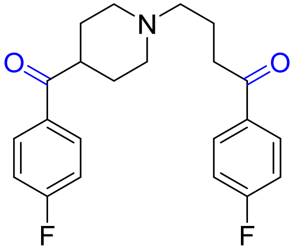

84a

^{1}H NMR,
400 MHz, $CDCl_3$

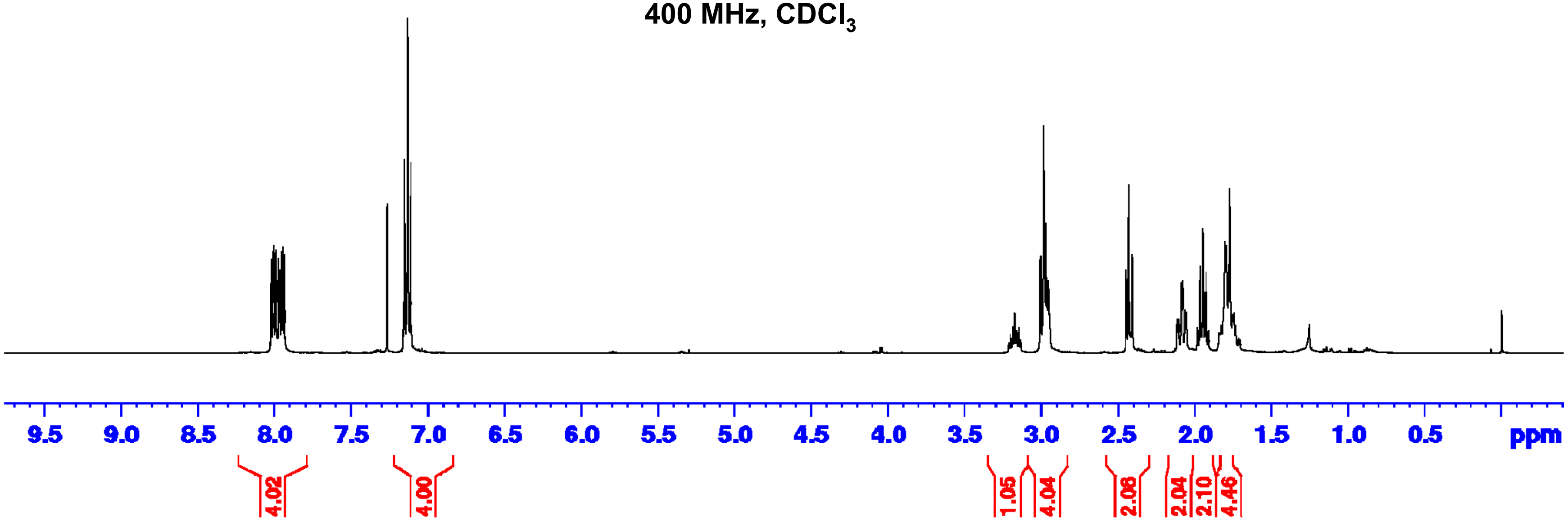

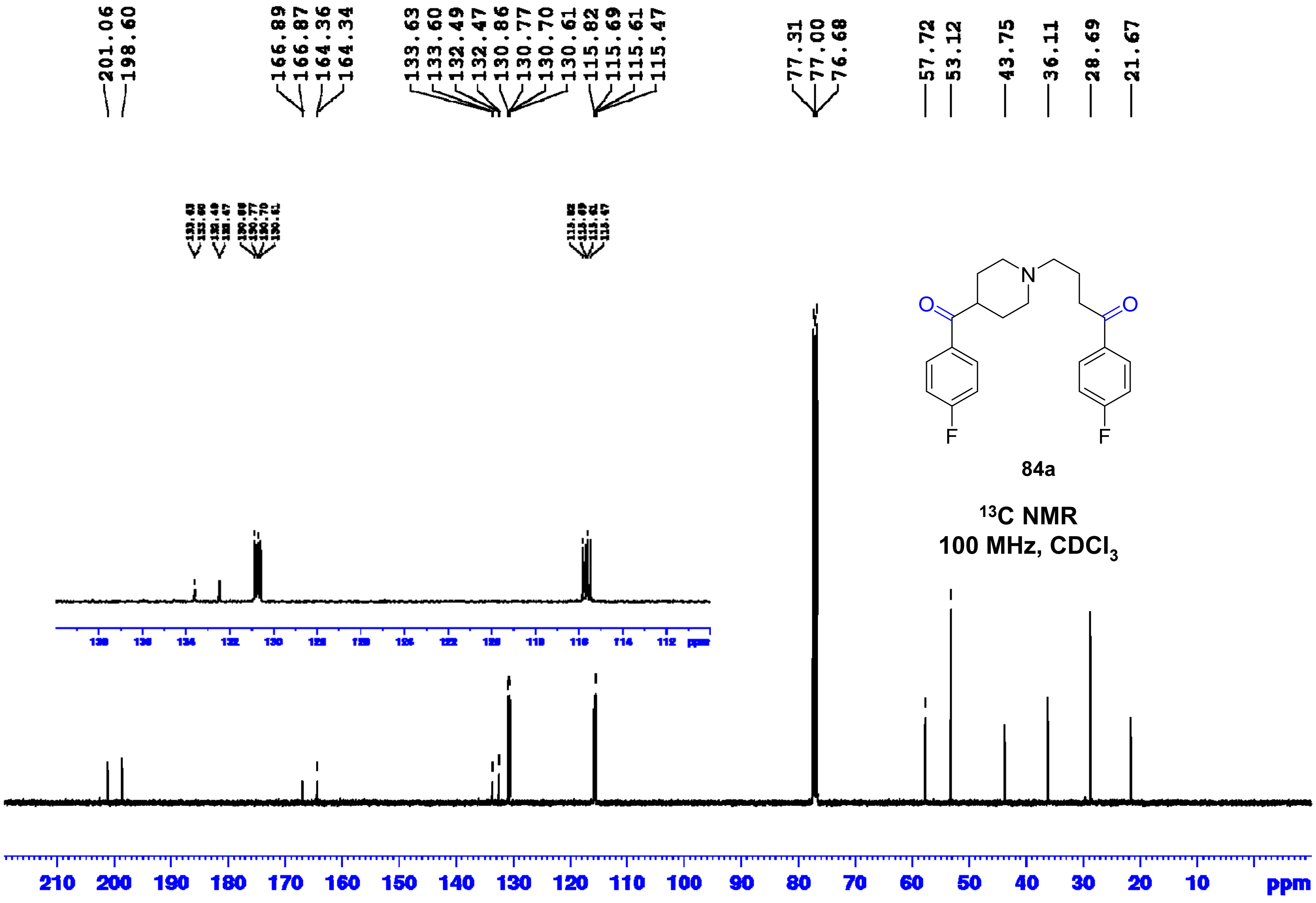

201.06
198.60
166.89
166.87
164.36
164.34
133.63
133.60
132.49
132.47
130.86
130.77
130.70
130.61
115.82
115.69
115.61
115.47
77.31
77.00
76.68
57.72
53.12
43.75
36.11
28.69
21.67
N
O
O
F
F
84a
^{13}C NMR
100 MHz, $CDCl_3$
210 200 190 180 170 160 150 140 130 120 110 100 90 80 70 60 50 40 30 20 10 ppm

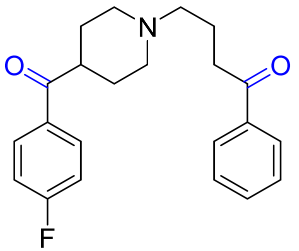

85a

^{1}H NMR,
400 MHz, $CDCl_3$

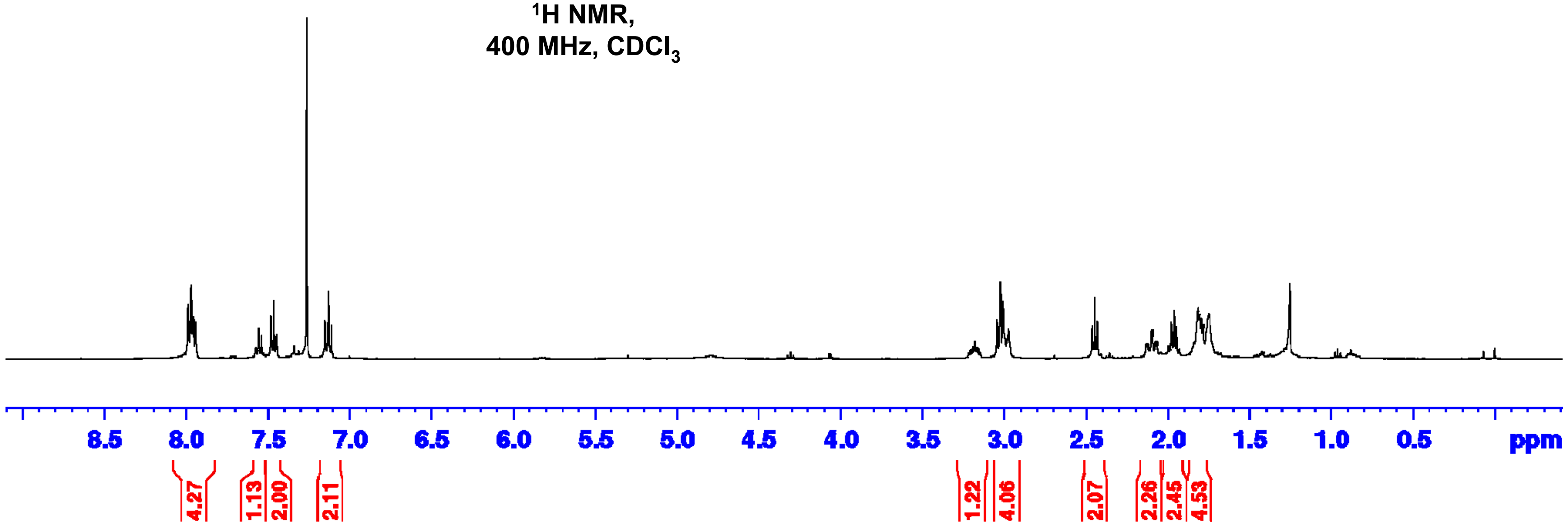

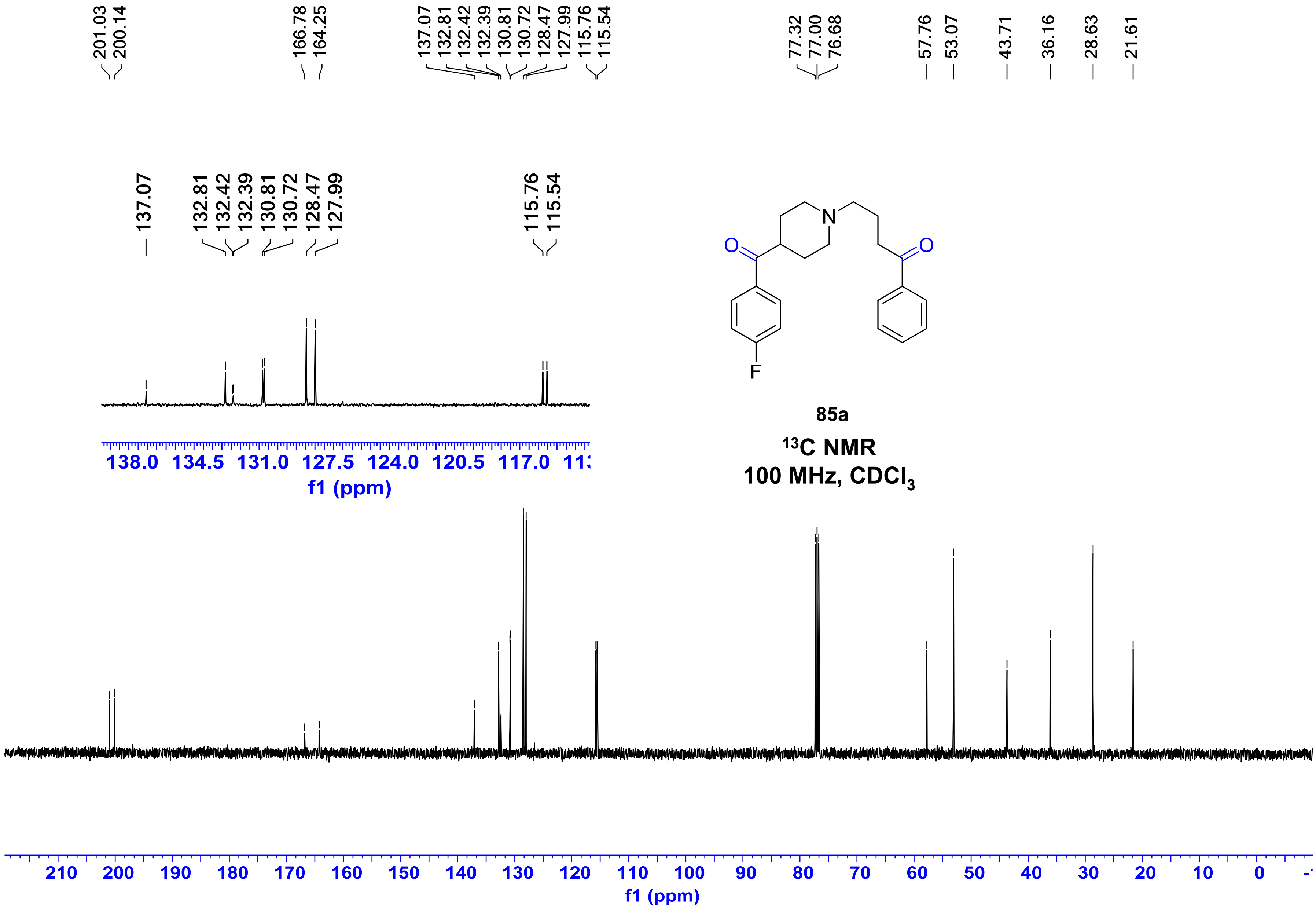
201.03
200.14
166.78
164.25
137.07
132.81
132.42
132.39
130.81
130.72
128.47
127.99
115.76
115.54
77.32
77.00
76.68
57.76
53.07
43.71
36.16
28.63
21.61
138.0 134.5 131.0 127.5 124.0 120.5 117.0
f1 (ppm)
O
N
O
F
85a
^{13}C NMR
100 MHz, $CDCl_3$
210 200 190 180 170 160 150 140 130 120 110 100 90 80 70 60 50 40 30 20 10 0
f1 (ppm)

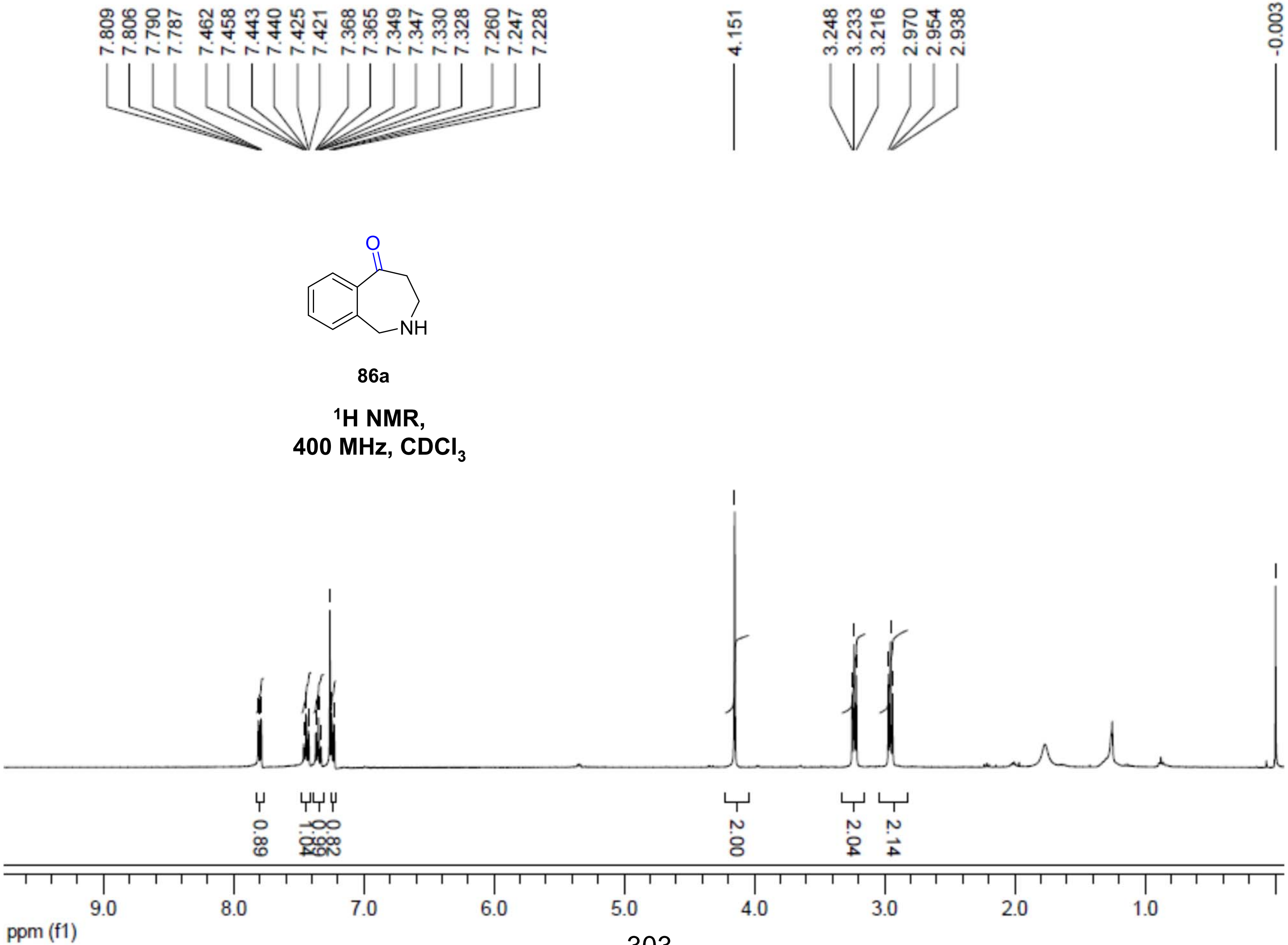
7.809
7.806
7.790
7.787
7.462
7.458
7.443
7.440
7.425
7.421
7.368
7.365
7.349
7.347
7.330
7.328
7.260
7.247
7.228
4.151
3.248
3.233
3.216
2.970
2.954
2.938
-0.003
O
NH
86a
1H NMR,
400 MHz, CDCl3
0.89
1.04
0.96
0.82
2.00
2.04
2.14
9.0
8.0
7.0
6.0
5.0
4.0
3.0
2.0
1.0
ppm (f1)

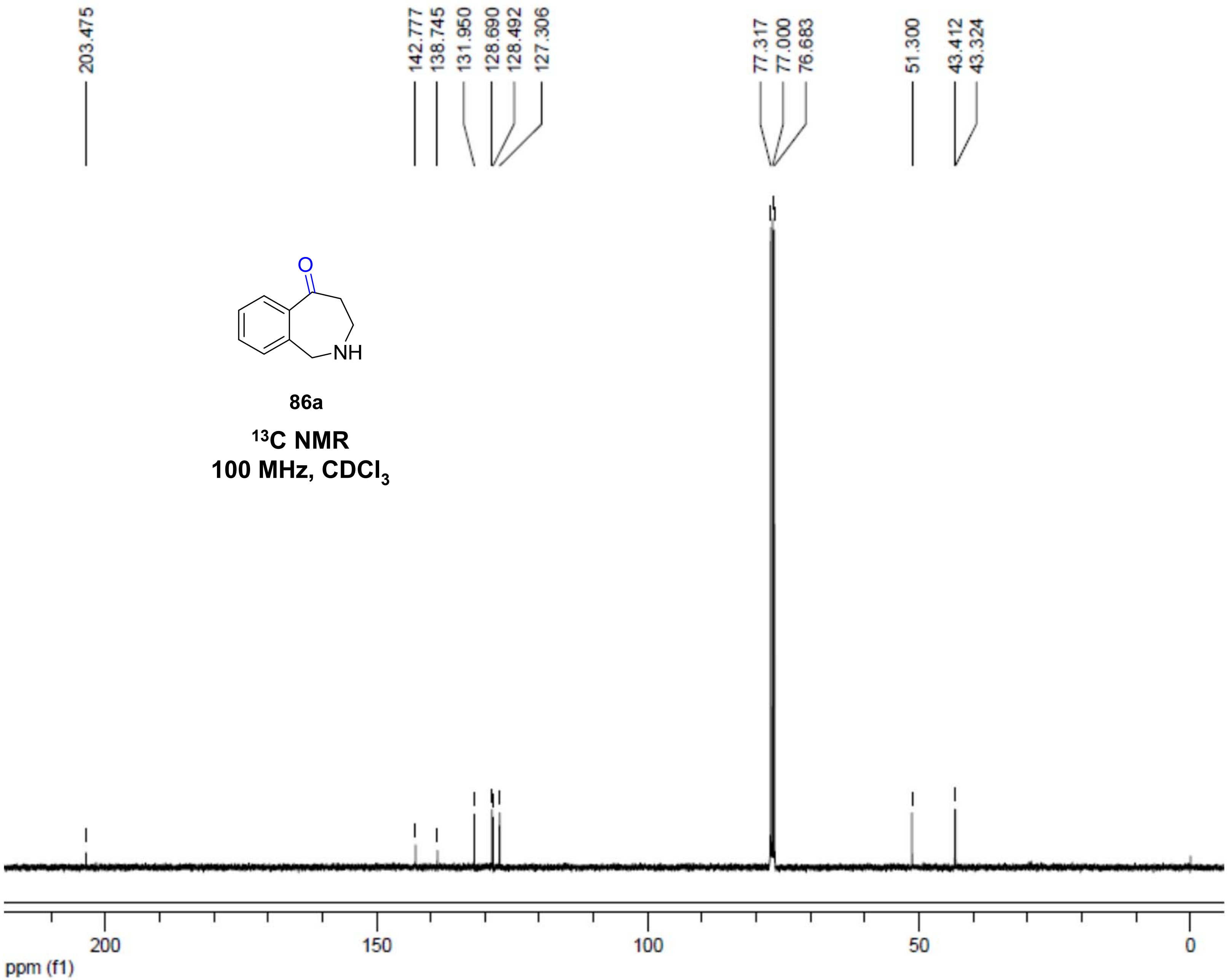
203.475
142.777
138.745
131.950
128.690
128.492
127.306
77.317
77.000
76.683
51.300
43.412
43.324
O
NH
86a
^{13}C NMR
100 MHz, $CDCl_3$
200
150
100
50
0
ppm (f1)

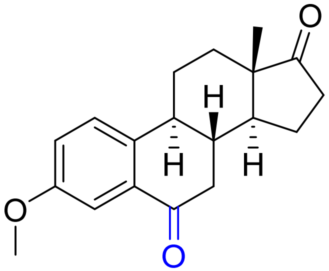

87a

^{1}H NMR, 400 MHz, $CDCl_3$

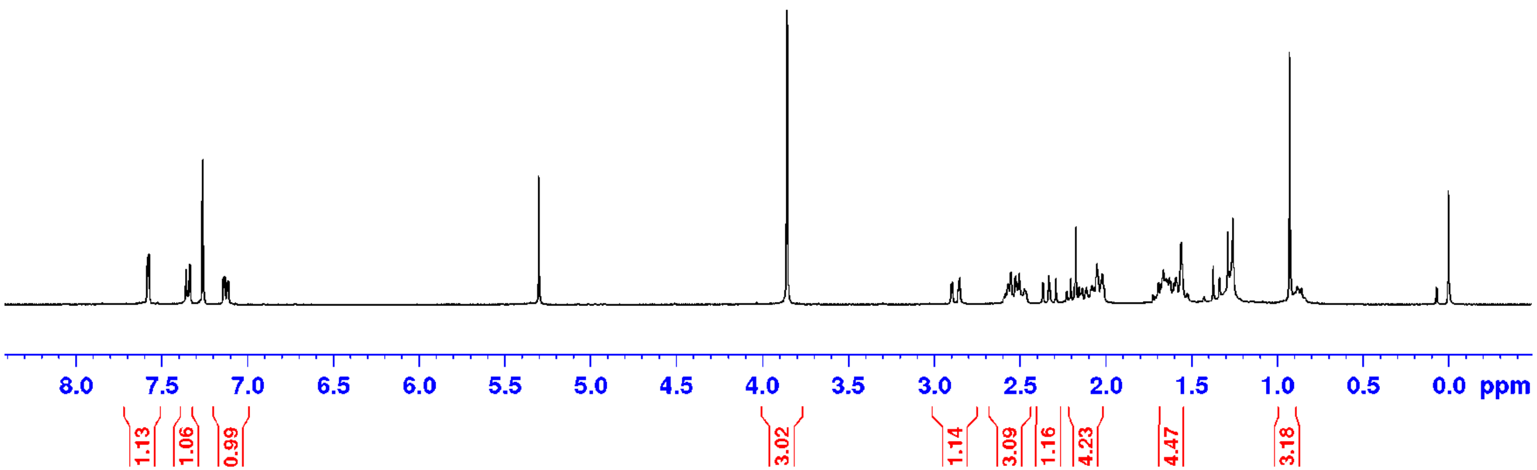

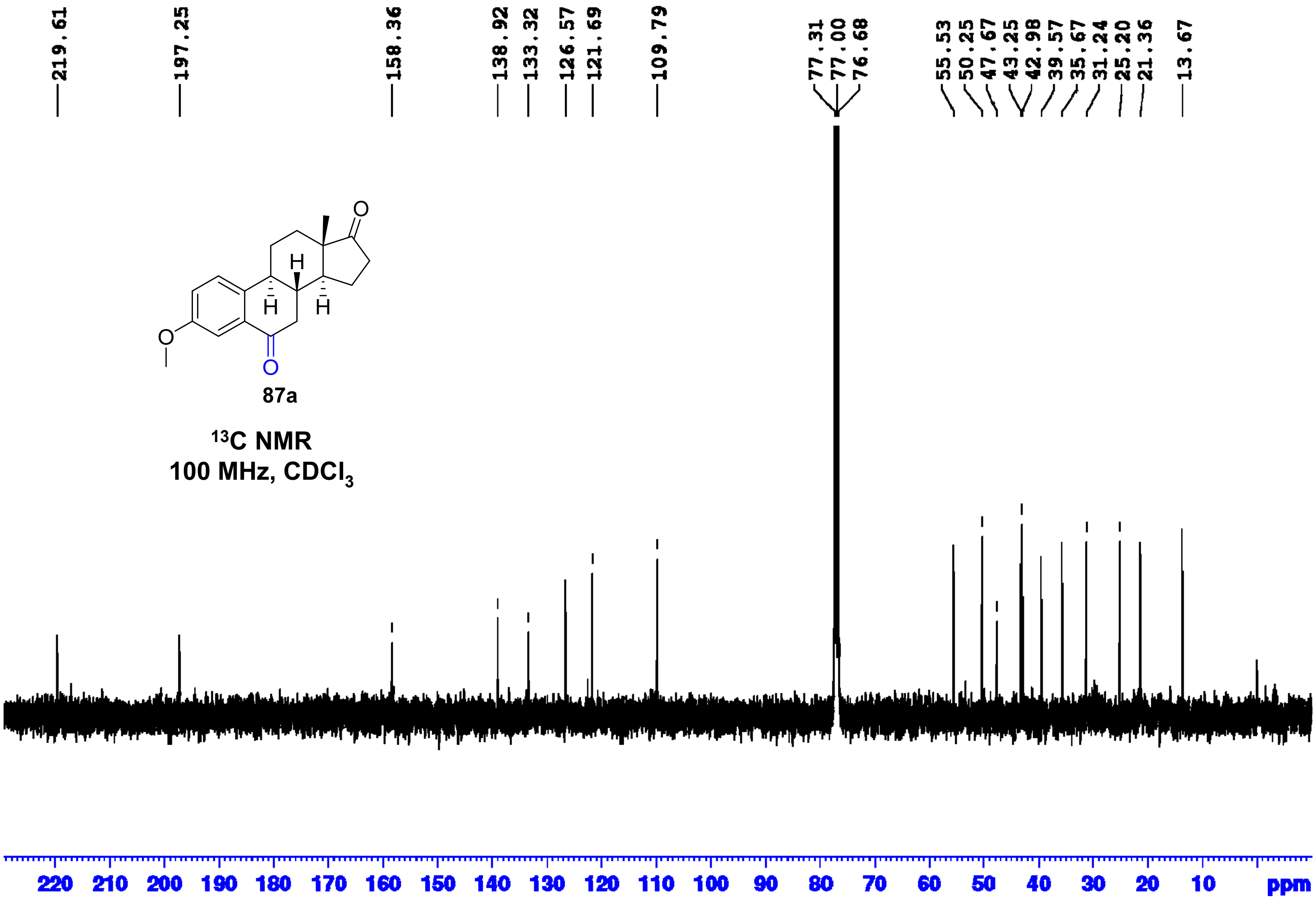

219.61
197.25
158.36
138.92
133.32
126.57
121.69
109.79
77.31
77.00
76.68
55.53
50.25
47.67
43.25
42.98
39.57
35.67
31.24
25.20
21.36
13.67
87a
^{13}C NMR
100 MHz, $CDCl_3$
220 210 200 190 180 170 160 150 140 130 120 110 100 90 80 70 60 50 40 30 20 10 ppm

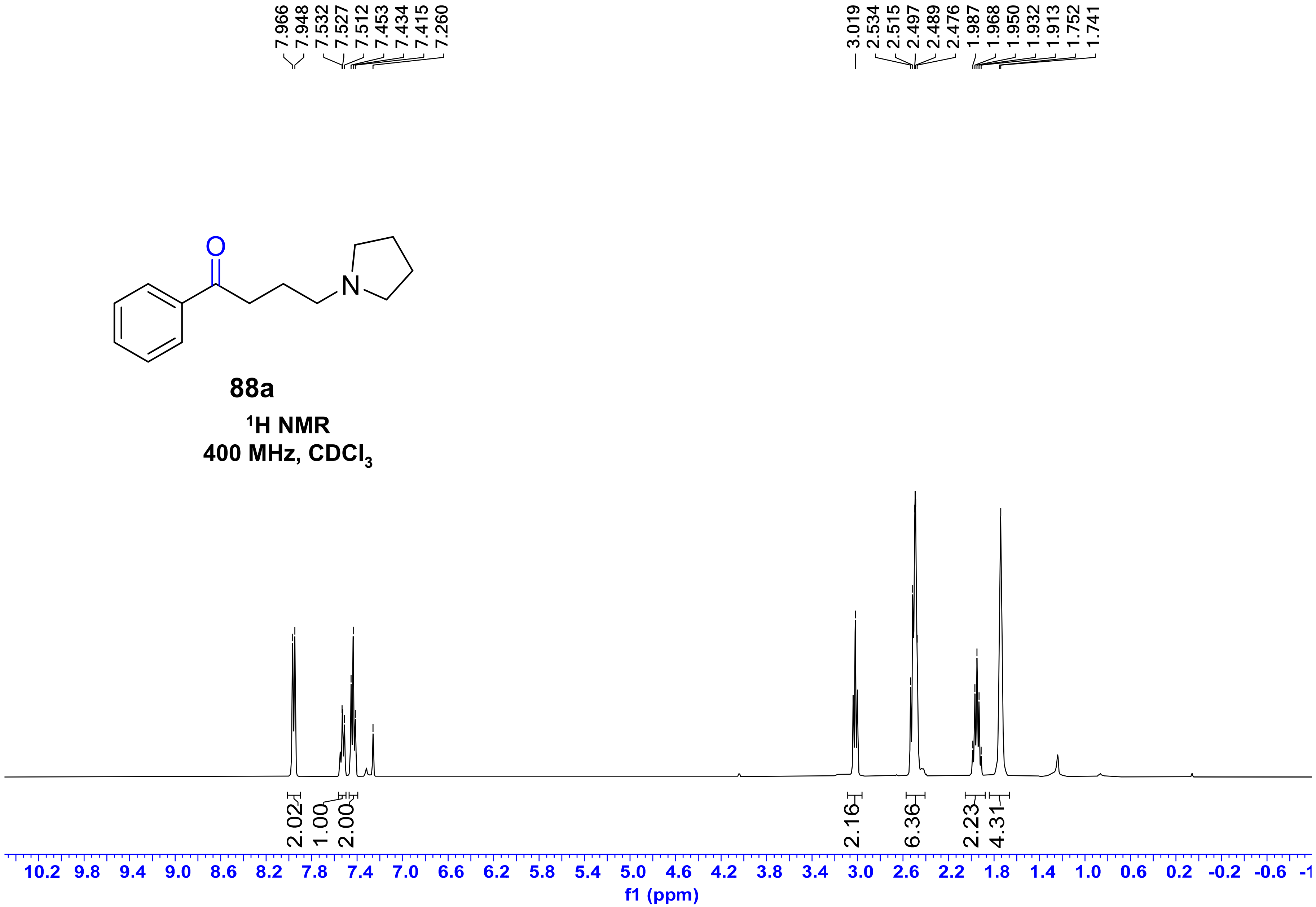
7.966
7.948
7.532
7.527
7.512
7.453
7.434
7.415
7.260
3.019
2.534
2.515
2.497
2.489
2.476
1.987
1.968
1.950
1.932
1.913
1.752
1.741
O
N
88a
1H NMR
400 MHz, CDCl3
2.02
1.00
2.00
2.16
6.36
2.23
4.31
10.2 9.8 9.4 9.0 8.6 8.2 7.8 7.4 7.0 6.6 6.2 5.8 5.4 5.0 4.6 4.2 3.8 3.4 3.0 2.6 2.2 1.8 1.4 1.0 0.6 0.2 -0.2 -0.6
f1 (ppm)

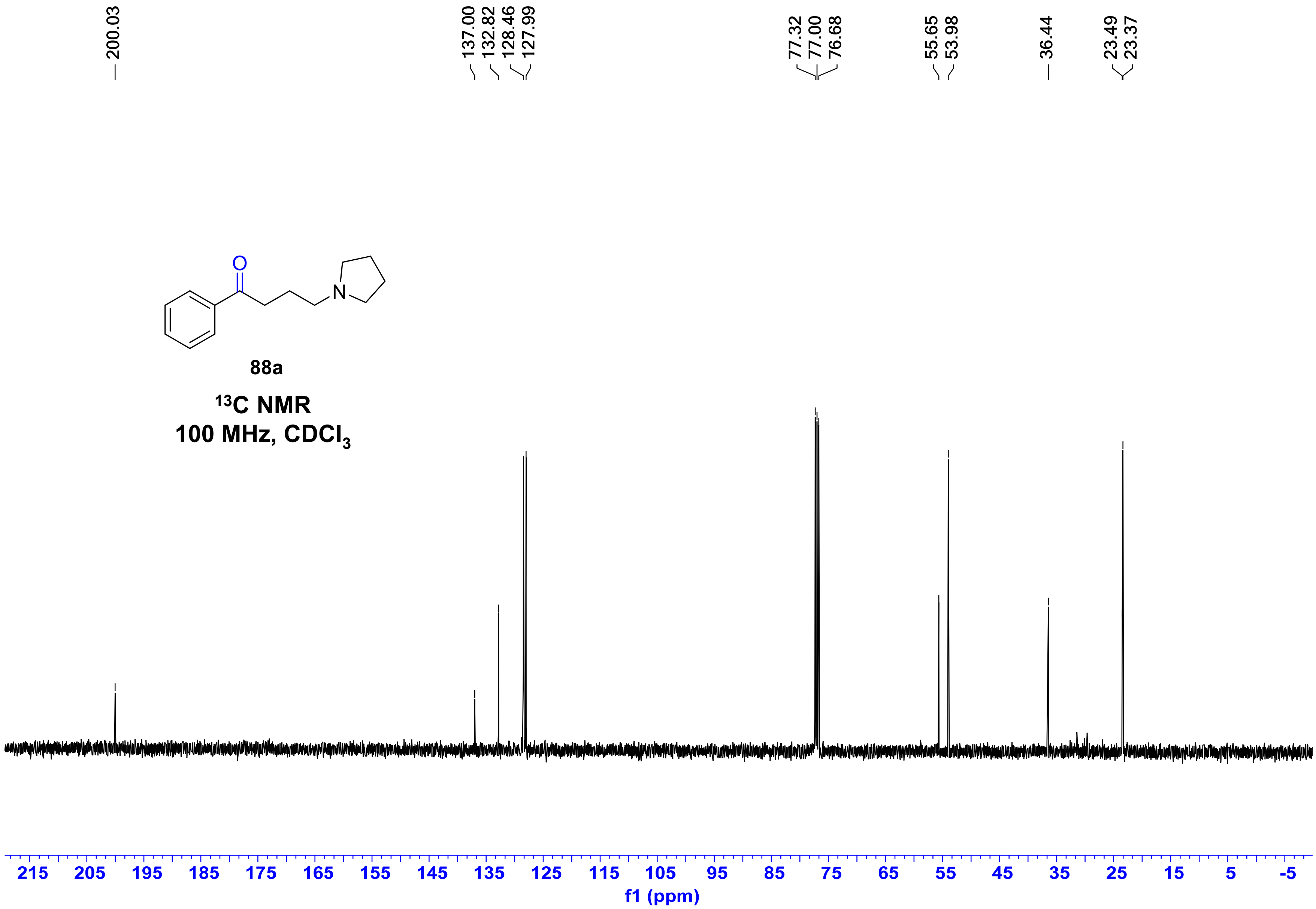
200.03
137.00
132.82
128.46
127.99
77.32
77.00
76.68
55.65
53.98
36.44
23.49
23.37
O
N
88a
13C NMR
100 MHz, CDCl3
215 205 195 185 175 165 155 145 135 125 115 105 95 85 75 65 55 45 35 25 15 5 -5
f1 (ppm)

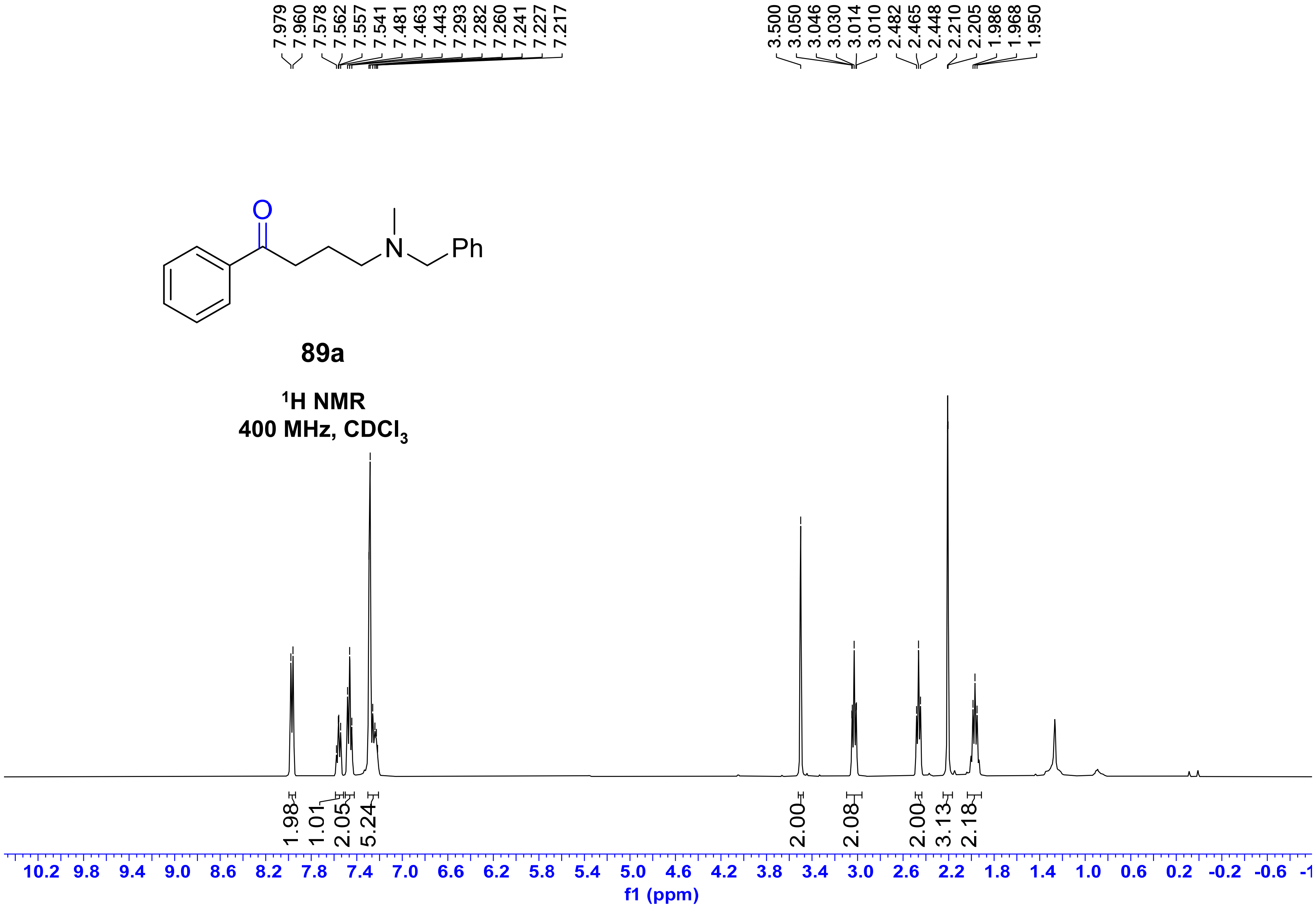
7.979
7.960
7.578
7.562
7.557
7.541
7.481
7.463
7.443
7.293
7.282
7.260
7.241
7.227
7.217
3.500
3.050
3.046
3.030
3.014
3.010
2.482
2.465
2.448
2.210
2.205
1.986
1.968
1.950
O
N
Ph
89a
1H NMR
400 MHz, CDCl3
1.98
1.01
2.05
5.24
2.00
2.08
2.00
3.13
2.18
10.2 9.8 9.4 9.0 8.6 8.2 7.8 7.4 7.0 6.6 6.2 5.8 5.4 5.0 4.6 4.2 3.8 3.4 3.0 2.6 2.2 1.8 1.4 1.0 0.6 0.2 -0.2 -0.6
f1 (ppm)

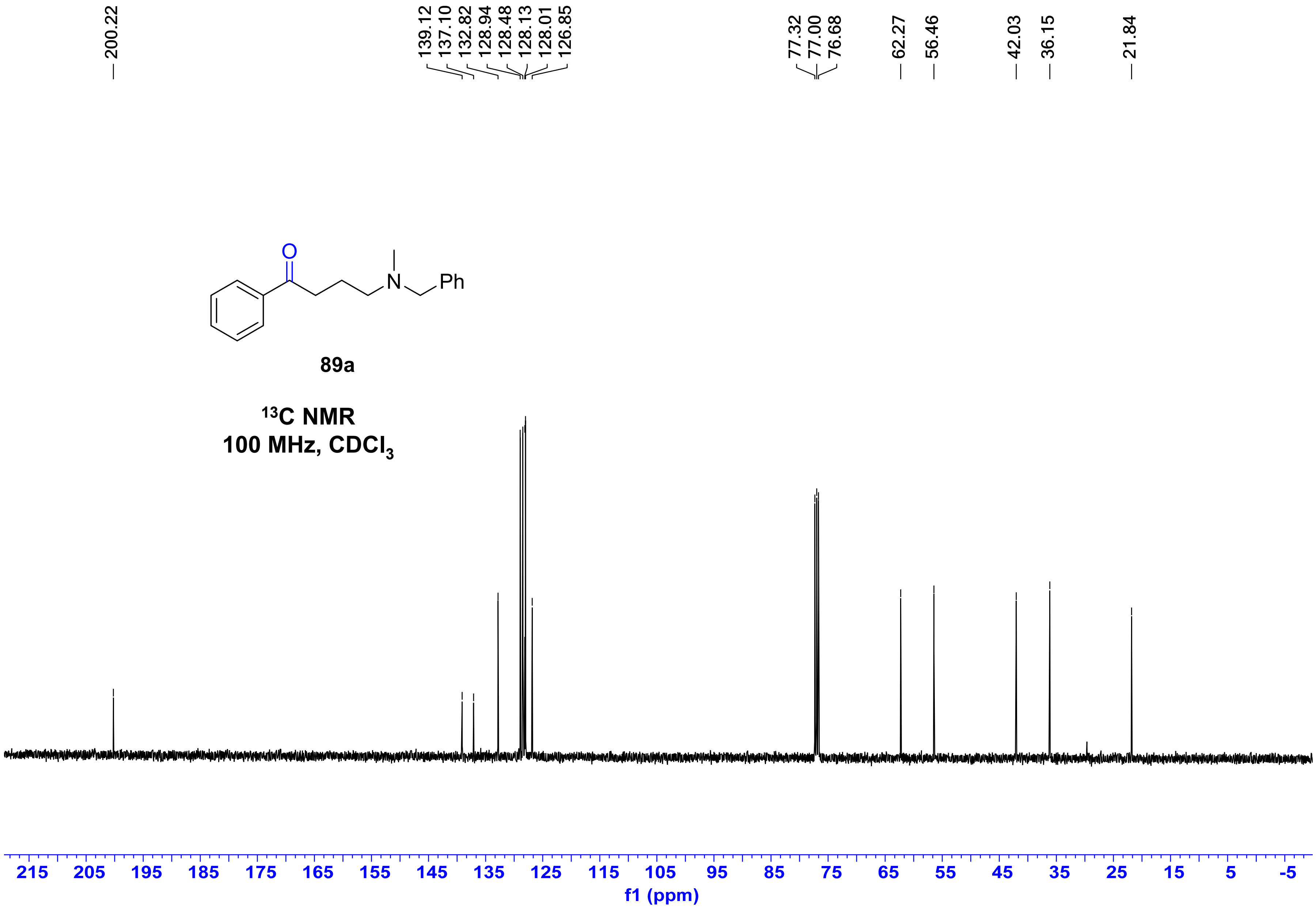
200.22
139.12
137.10
132.82
128.94
128.48
128.13
128.01
126.85
77.32
77.00
76.68
62.27
56.46
42.03
36.15
21.84
O
N
Ph
89a
13C NMR
100 MHz, CDCl3
215 205 195 185 175 165 155 145 135 125 115 105 95 85 75 65 55 45 35 25 15 5 -5
f1 (ppm)

14. HPLC Traces

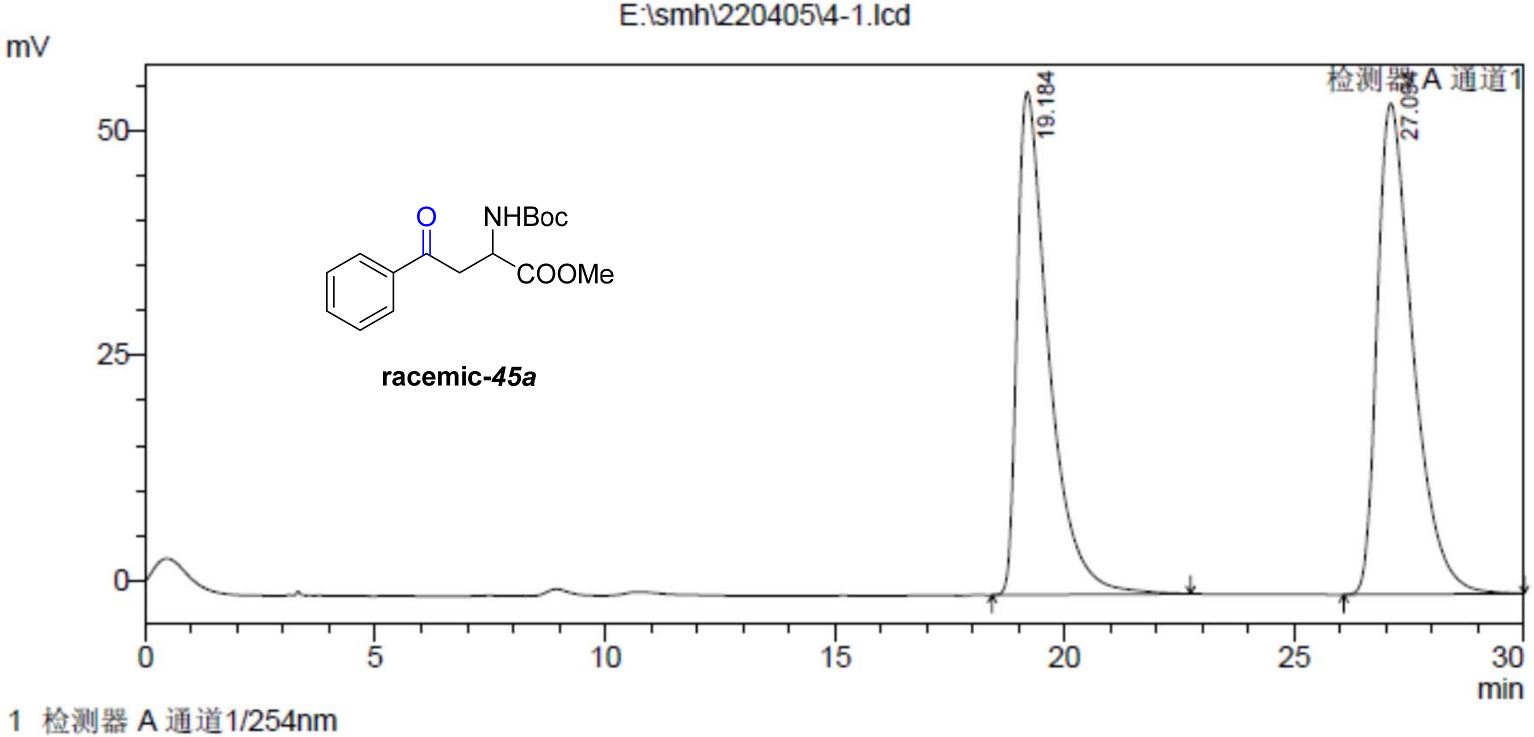

检测器 A Ch1 254nm

Peak#	Ret. Time	Area	Area %
1	19.184	2665078	47.524
2	27.094	2942726	52.476
总计		5607805	100.000

E:\smh\220405\4-3.lcd

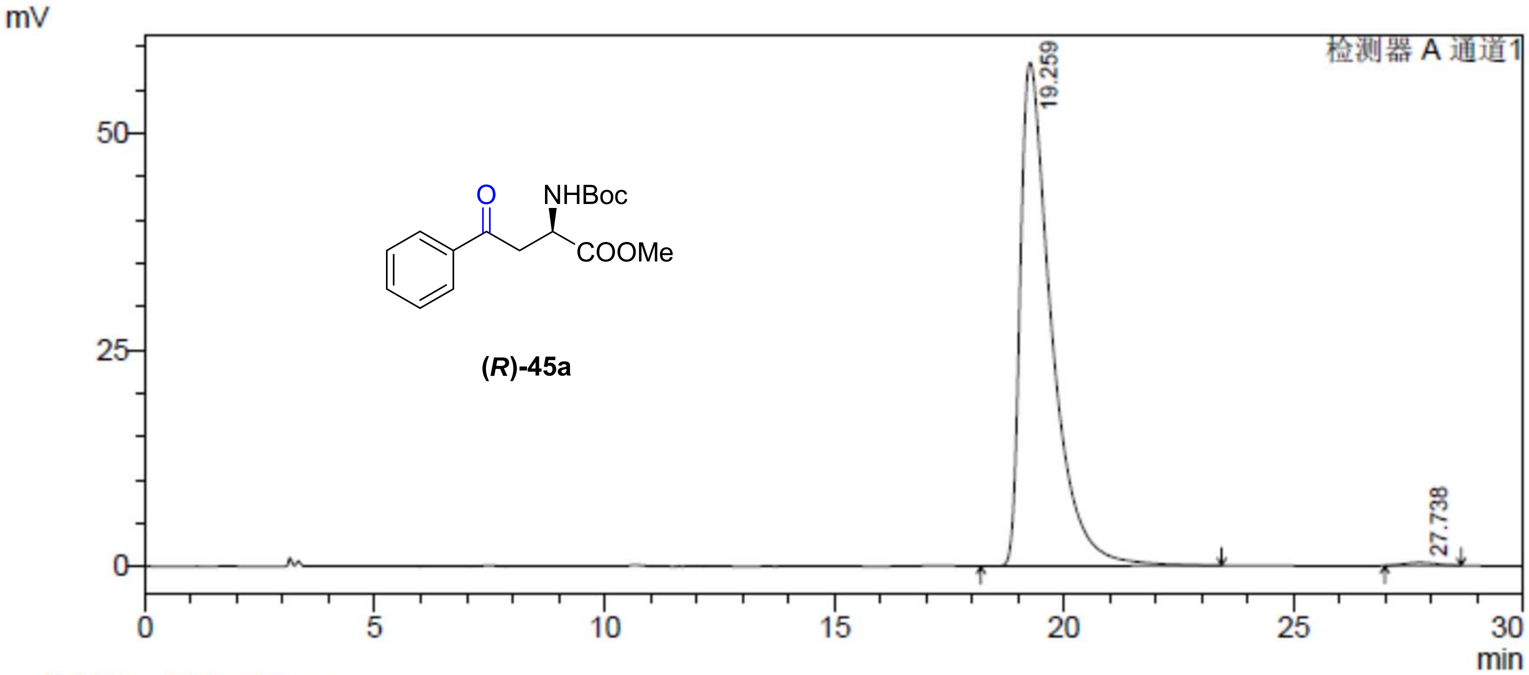

1 检测器 A 通道1/254nm

检测器 A Ch1 254nm

Peak#	Ret. Time	Area	Area %
1	19.259	2736533	99.367
2	27.738	17430	0.633
总计		2753963	100.000
